# Late-stage peptide C–H alkylation for bioorthogonal C–H activation featuring solid phase peptide synthesis

**DOI:** 10.1038/s41467-019-11395-3

**Published:** 2019-08-07

**Authors:** Alexandra Schischko, Nikolaos Kaplaneris, Torben Rogge, Giedre Sirvinskaite, Jongwoo Son, Lutz Ackermann

**Affiliations:** Institut für Organische und Biomolekulare Chemie, Georg-August-Universität Göttingen, Tammannstr. 2, 37077 Göttingen, Germany

**Keywords:** Homogeneous catalysis, Synthetic chemistry methodology, Solid-phase synthesis

## Abstract

Methods for the late-stage diversification of structurally complex peptides hold enormous potential for advances in drug discovery, agrochemistry and pharmaceutical industries. While C–H arylations emerged for peptide modifications, they are largely limited to highly reactive, expensive and/or toxic reagents, such as silver(I) salts, in superstoichiometric quantities. In sharp contrast, we herein establish the ruthenium(II)-catalyzed C–H alkylation on structurally complex peptides. The additive-free ruthenium(II)carboxylate C–H activation manifold is characterized by ample substrate scope, racemization-free conditions and the chemo-selective tolerance of otherwise reactive functional groups, such as electrophilic ketone, bromo, ester, amide and nitro substituents. Mechanistic studies by experiment and computation feature an acid-enabled C–H ruthenation, along with a notable protodemetalation step. The transformative peptide C–H activation regime sets the stage for peptide ligation in solution and proves viable in a bioorthogonal fashion for C–H alkylations on user-friendly supports by means of solid phase peptide syntheses.

## Abstract (web summary)

Palladium-catalyzed late-stage diversification of structurally complex peptides has major shortcomings. Here, the authors developed a ruthenium-catalyzed C–H alkylations of peptides allowing for fluorescence labeling, late-stage diversifications and peptide ligations by solid phase peptide synthesis.

## Introduction

**Fig. 1:**
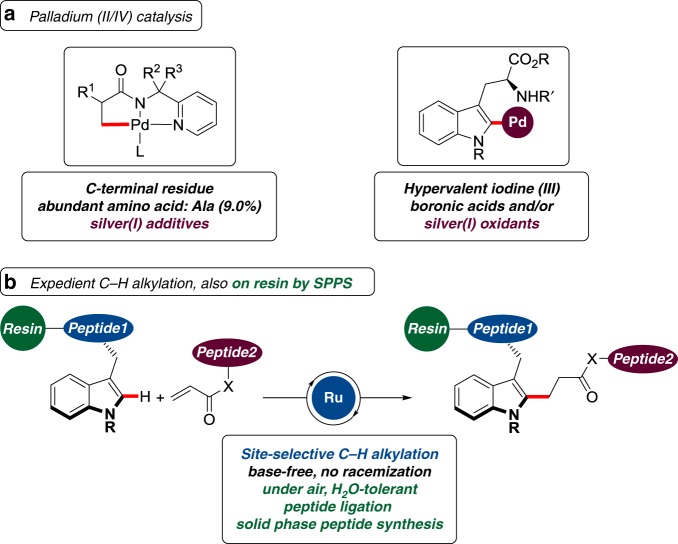
C–H alkylation for transformative peptide diversification. **a** Palladium(II/IV) arylation with reactive, expensive, or toxic reagents; **b** versatile peptide C–H alkylation/ligation in solution and on solid support

The chemo-selective diversification and ligation of structurally complex peptides^1–3^ is of prime importance in biomolecular chemistry for the modification and assembly of peptides and proteins^4^. Thus, non-natural peptides are paramount to numerous applied areas, ranging from proteomics, diagnostics, and asymmetric syntheses to drug delivery^5^. Likewise, decorated peptides have been identified as increasingly potent therapeutics. Particularly, unnatural amino acids and peptides populate a unique conformational space and feature distinct bioactivities, whereas being stable toward proteolytic enzymes^6–9^. Thus, the chemo-selective diversification of peptides bears great potential for inter alia medicinal chemistry, synthetic biology, and pharmaceutical industries^2^. As a direct consequence, strategies for the preparation of unnatural amino acids have been devised, for instance, involving asymmetric syntheses^10^. Despite the indisputable advances, such approaches largely require prefunctionalized substrates that translate into labor-intensive, multi-step operations, and thereby generate undesired waste products^11^. Further, cross-couplings between two prefunctionalized substrates are viable under basic reaction conditions. However, also these conventional cross-couplings can lead to the racemization of the peptidic scaffold, and yield stoichiometric amount of undesired byproducts. In response to these limitations, metal-catalyzed C‒H activation^12–18^ has emerged as a more atom- and step-economical tool toward decorated peptides^19–21^, with considerable potential for the late-stage diversification of peptides of relevance to agrochemical and pharmaceutical industries^22–30^. Thus, peptide C–H arylations have recently witnessed a significant momentum by means of ruthenium^31,32^, and oxidative bidentate^33,34^ chelation-assisted palladium(II) catalysis, among others^35–44^. Although these C–H arylation protocols have enabled the synthesis of substituted peptides, they are inherently limited to the introduction of the aryl motif. Furthermore, such arylative approaches require reactive aryliodonium and aryldiazonium salts, are operative under basic conditions or employ stoichiometric quantities of costly and toxic silver(I) salts, thus significantly compromising the step- and atom-economical nature of the C–H activation approach (Fig. 1a).

In sharp contrast, we have now addressed these major shortcomings of C–H functionalization by developing peptide C–H alkylation^45–48^ on structurally complex peptides (Fig. 1b). Notable features of our findings include (i) ruthenium(II)carboxylate-catalyzed^49–51^ peptide C–H alkylation under base-free conditions, (ii) racemization-free amino acid and peptide late-stage diversification, (iii) detailed mechanistic insights into acid-enabled C–H alkylation by experiment and computation, (iv) versatile peptide fluorescence labeling, and (v) chemo-selective peptide ligation in a bioorthogonal fashion. A key asset of our strategy is represented by the peptide C–H alkylation by solid phase peptide synthesis (SPPS)^52–54^, setting the stage for the operationally simple assembly of structurally complex peptides in an iterative manner on user-friendly resin support.

## Results

### Establishing C–H activation

**Fig. 2:**
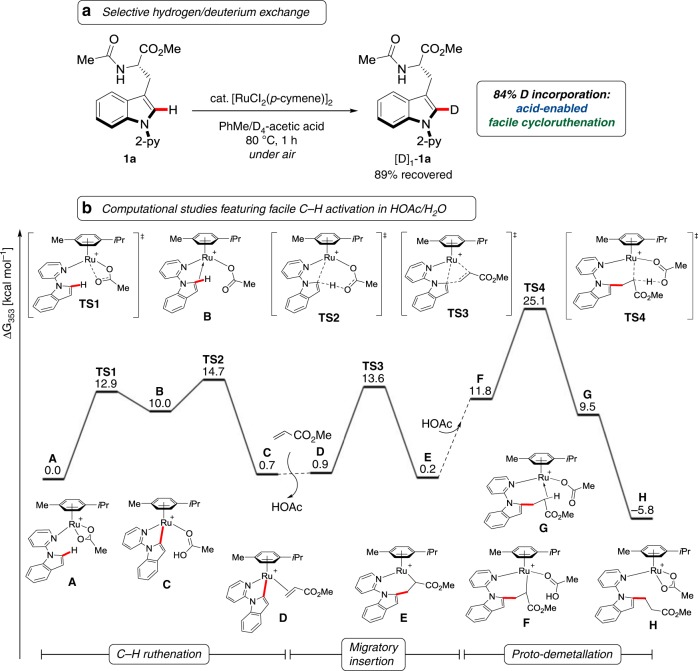
Ruthenium-catalyzed C–H alkylation for peptide diversification. **a** Experimental evidence for facile C–H cleavage by selective H/D scrambling; **b** Calculated Gibbs free energy profile for the C–H alkylation at the PW6B95-D3(BJ)/def2-QZVP* + SMD(AcOH)//M06-D3/def2-SVP level of theory

We initiated our studies by probing various reaction conditions for the envisioned C–H alkylation of tryptophan **1a** (Table 1). Detailed experimentation indicated aqueous acetic acid to be the solvent of choice (Table 1, entries 1–4). Thereby, basic reaction conditions could be prevented toward a racemization-free hydroarylation regime. The powerful ruthenium(II) catalyst allowed for the site-selective C–H alkylation under mild conditions (Table 1, entries 4–8), which even set the stage C–H functionalization at physiological temperature of only 37 °C (Table 1, entry 9). The robustness of the ruthenium catalysis was reflected by being fully tolerant of H_2_O (Table 1, entry 11). It is noteworthy that experimental mechanistic studies unraveled that an acid-enabled facile hydrogen/deuterium exchange with excellent levels of position-selectivity (Fig. 2a), being indicative of an organometallic C–H activation manifold. These features were mirrored by density functional theory (DFT) calculations at the PW6B95-D3(BJ)/def2-QZVP* + SMD(AcOH)//M06-D3/def2-SVP level of theory (Fig. 2b and Supplementary Data set 1). Our computational studies provided strong support for a facile C–H ruthenation, along with a rate-determining protodemetalation by the solvent acetic acid. Furthermore, our detailed DFT analysis highlighted the role of the various η^6^-arene ligands, and the key importance of mono-cationic ruthenium(II) complexes^55^ as the crucial intermediates for a facile base-assisted internal electrophilic substitution^56,57^ C–H activation, the results of which are described in the Supplementary Fig. 16.

**Table 1:** Initial optimization of the C–H alkylation of tryptophan **1a**


Entry	Solvent	[1a]	*T*/°C	Yield/%
1	GVL/HOAc (1/1)	0.3 m	120	58
2	HO_2_CEt	0.3 m	120	35
3	HOPiv	0.3 m	120	23
4	HOAc	0.3 m	120	81
5	HOAc	0.3 m	100	88
6	HOAc	0.3 m	80	73
7	HOAc	1.0 m	80	90
8	HOAc	1.0 m	60	80
9	HOAc	1.0 m	37	54
10	HOAc	1.0 m	80	83^a^
11	HOAc/H_2_O (3/1)	1.0 m	80	75

Key aspects of C–H alkylation optimization, highlighting acid-enabled C–H activation. Reaction conditions: **1a** (0.15 mmol), **2a** (0.45 mmol), [RuCl_2_(*p*-cymene)]_2_ (10 mol %), solvent, 15 h

^a^[RuCl_2_(*p*-cymene)]_2_ (5.0 mol %). 2-py: 2-pyridyl

### Versatility of peptide C–H activation

**Fig. 3:**
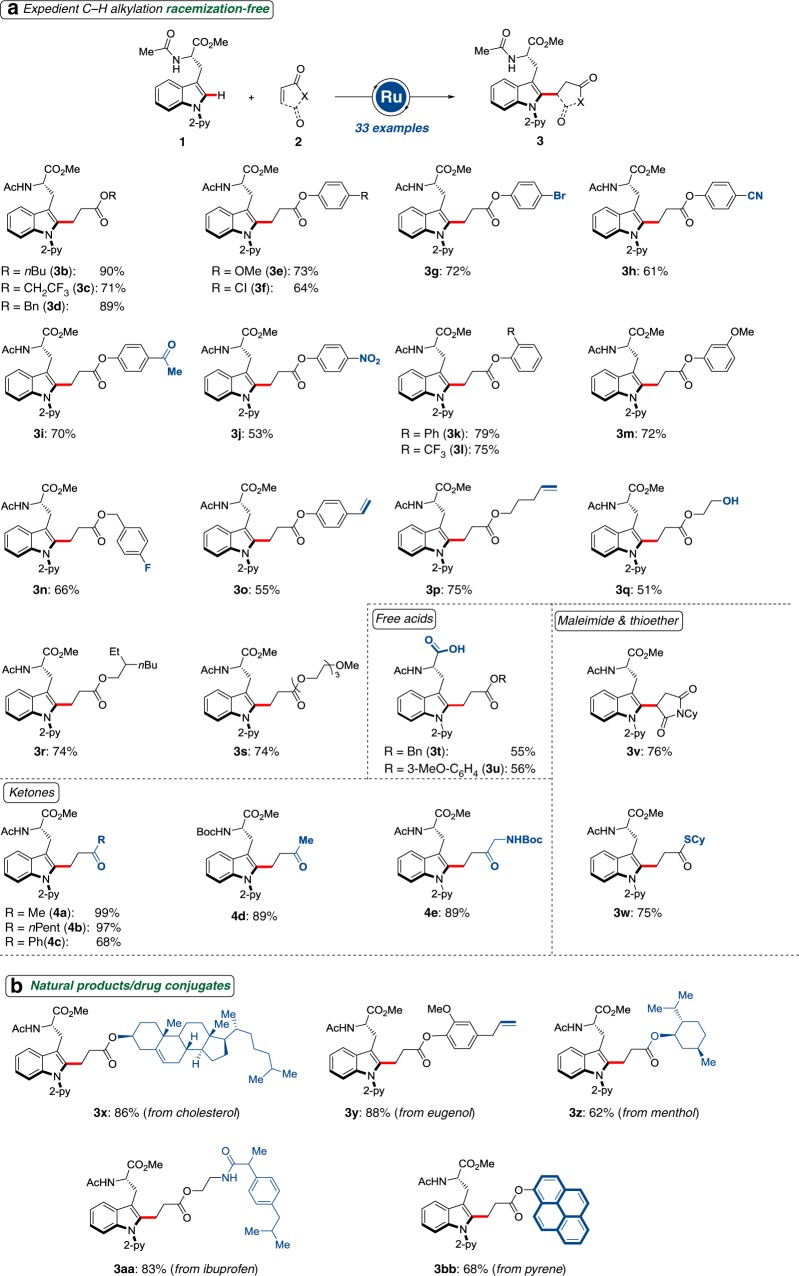
Expedient ruthenium(II)-catalyzed amino-acid C–H alkylation. **a** Ruthenium(II)-catalyzed C–H alkylation with alkenes **2**; **b** ruthenium(II)-catalyzed C–H alkylation for amino-acid conjugates featuring natural products and drugs

With the optimized conditions in hand, we probed the robustness of the ruthenium(II)-catalyzed C–H alkylation regime employing alkenes **2** (Fig. 3a). The C–H alkylation was widely applicable, and the ruthenium(II) catalyst proved tolerant of otherwise reactive functional groups, including fluoro, chloro, bromo, cyano, ester, styrene, thioesters, ketone, and nitro substituents, which are invaluable for post-synthetic manipulation by inter alia olefin metathesis. Likewise, the ruthenium-catalyzed C–H alkylation set the stage for the chemo-selective ligation with PEGylated motifs, and enabled position-specific fluorescent labeling. The PEGylation strategy is highly attractive to enhance the solubility and pharmacological properties for drug delivery, and to improve the self-organization and photoluminescent properties of supramolecular nanostructures. The robustness of the developed C–H alkylation was reflected by the tolerance of free carboxylic acids (**3t** and **3u**), giving rise to useful building blocks. The versatile ruthenium(II) catalysis manifold proved also amenable to sensitive ketones under aqueous conditions. The mild nature of the C–H alkylation allowed here for the use of synthetically-useful Boc-protected amino acids and amino ketones without cleavage of the sensitive Boc group (**4d** and **4e**, respectively). Moreover, our C–H activation strategy set the stage for the chemo-selective preparation of amino-acid conjugates bearing drugs and natural products, including cholesterol, eugenol, menthol, and ibuprofen (Fig. 3b).

**Fig. 4:**
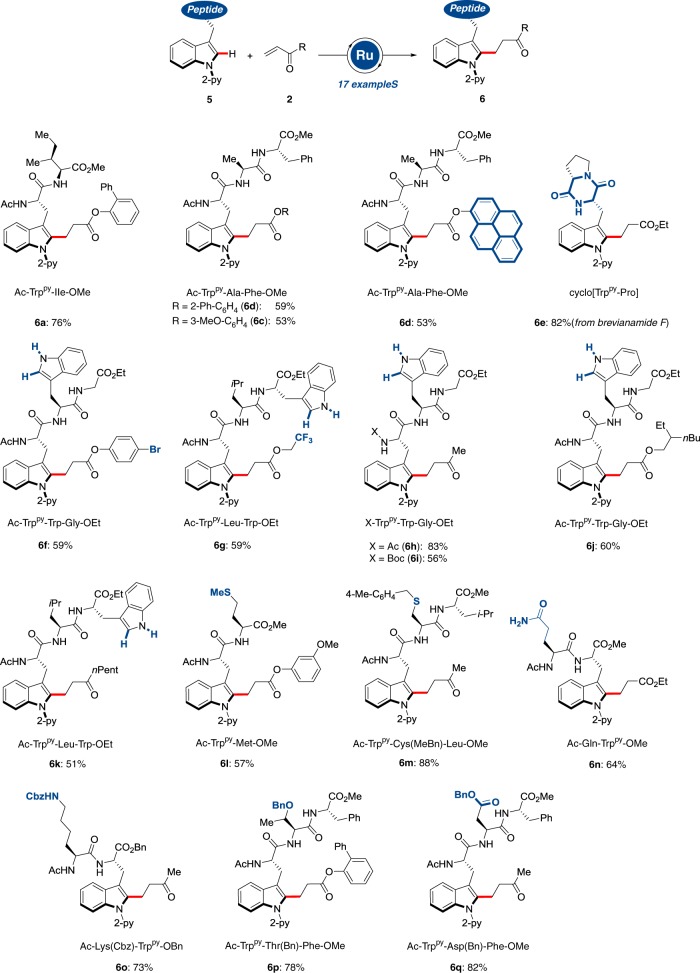
Late-stage functionalization of peptides. Ruthenium(II)-catalyzed C–H alkylation of structurally complex peptides **5** with sensitive functional groups

With a robust method for the base-free C–H manipulation of amino acids being established, we next explored the challenging diversification of structurally complex peptides **5** (Fig. 4). Thus, the exceedingly mild modification of peptides proved viable with excellent levels of position- and chemo-selectivities, even on NH-free tryptophan-containing peptides, albeit owing to the peptide solubilities at lower concentrations in select cases. Moreover, various peptides with amino acids bearing coordinating side chains were well tolerated in the ruthenium-catalyszed C–H alkylation. Likewise, the presence of reactive bromoarene moieties did not affect the outcome of the C–H alkylation, being indicative of its complementary nature as compared with typical arylation regimes. Generally, the bioorthogonal, acid-enabled C–H alkylation proceeded without any signs of racemization on the peptidic backbone.

**Fig. 5:**
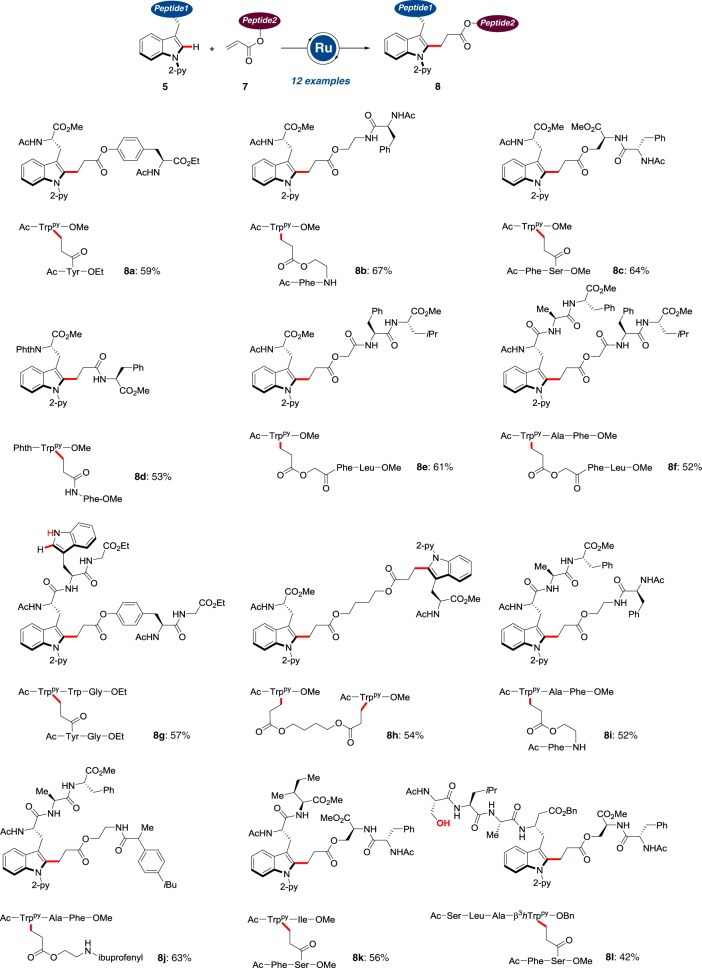
Peptide ligation via C–H functionalization. Ruthenium(II)-catalyzed C–H alkylation, occurring without racemization including β-peptides and twofold C–H activations

The power of the ruthenium(II) C–H activation manifold was further reflected by the smooth ligation of peptides **5** with engineered peptides **7** (Fig. 5). Hence, substrates **7** derived from the natural amino acids serine and tyrosine were selectively ligated with the tryptophan-derived peptides **5** to furnish the products **8**. Here, the amide and ester moieties in the peptidic backbone were fully tolerated without racemization of the stereogenic centers. In addition, 2-aminoethanol and hydroxyacetic acid were used as enabling linker motifs to provide atom-economical access to the ligated products **8b**, **8e**, **8f**, **8i**, and **8j**. These findings highlight the unique flexibility of the C–H alkylation-based ligation method in that it can be easily adapted to alcohol- or thiol-containing substrates. The judicious choice of an appropriately designed diene linker set the stage for a twofold C–H activation, delivering the double C–H activation/ligation product **8h**, whereas the ibuprofen-derived hybrid **8j** was likewise efficiently accessed by the chemo-selective C–H alkylation strategy. The versatile C–H activation strategy was not limited to α-amino acids. Indeed, our approach enabled the C–H alkylation of β-amino acid-derived peptides as well, thereby providing step-economical access to hexapeptide **8l** with, among others, unprotected serine.

**Fig. 6:**
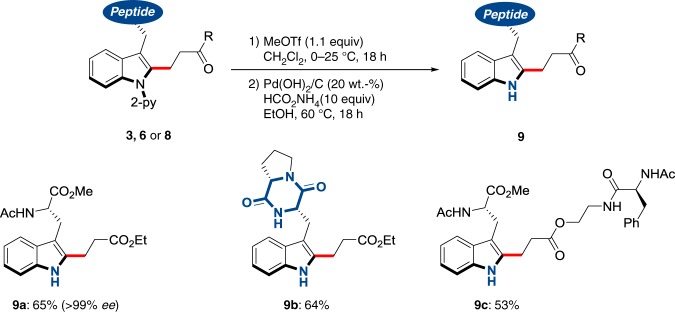
Expedient access to functionalized NH-free tryptophan-containing peptides. Traceless removal of the pyridine group under racemization-free conditions

The synthetic utility of our C–H alkylation regime was further demonstrated by the traceless removal of the pyridyl motif to ensure expedient access to NH-free tryptophan-containing peptides (Fig. 6).

### Solid phase peptide synthesis peptide C–H alkylation

**Fig. 7:**
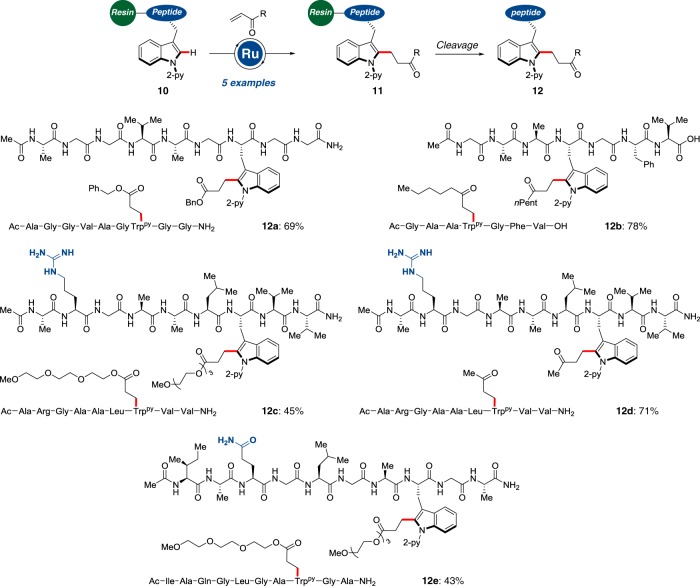
C–H functionalization on solid support. On-resin C–H alkylation by user-friendly solid phase peptide synthesis providing access to diversely decorated nona- and decapeptides

Encouraged by the outstanding robustness and chemoselectivity of the bioorthogonal C–H alkylation regime, we probed the peptide C–H alkylation on solid-supported peptides. To this end, we designed the amino acid Fmoc-Trp^py^-OH ready for SPPS protocols, which was conveniently incorporated into peptides **10** (Fig. 7 and the Supplementary Information). With the resin-supported peptides in hand, we explored the challenging C–H alkylation to afford the desired functionalized peptides with excellent levels of chemoselectivity, delivering inter alia arginine and glutamine-containing peptides **12c**–**e**. Notably, the powerful ruthenium(II)-catalyzed C–H alkylation occurred efficiently on the solid support despite of limited diffusion control. Our approach clearly showcased the key advantages of the on-resin C–H alkylation as to achieving high conversions without compromising the ease of purification. In addition, the challenging C–H activation of poorly soluble peptides and peptide ligation are accomplished by the on-resin approach, whereas jellification and aggregation of peptides during the C–H activation were circumvented through the peptide late-stage diversification strategy on-resin.

## Discussion

We have realized a metal-catalyzed peptide C–H alkylation strategy for the racemization-free diversification of peptides in a bioorthogonal manner. Thus, an air- and water-tolerant ruthenium(II) catalyst set the stage for the chemo-selective late-stage diversification of amino acids and peptides. The robustness of the expedient ruthenium(II) catalysis was reflected by C–H modification and ligation of structurally complex peptides. The operationally simple late-stage modification provided direct access to structurally complex molecular architectures and enabled peptide C–H alkylations on resin by solid phase peptide synthesis. These findings should prove invaluable for applications to medicinal chemistry, and biochemistry, illustrating the robust nature of the ruthenium(II)carboxylate-catalyzed C–H activation for the assembly of engineered molecular architecture. Our report also illustrates the outstanding synthetic utility of ruthenium(II)-catalyzed C–H activation towards complexity-increasing peptide syntheses, which should prove instrumental for fluorescence labeling, late-stage diversifications and peptide ligations in academia as well as in applied areas, including, but not limited to, agrochemical and pharmaceutical industries.

## Methods

### Ruthenium(II)-catalyzed C–H alkylation of peptides

A 10 mL conical-bottom test tube with a stir bar was charged with py-peptide (30–150 µmol) and [RuCl_2_(*p*-cymene)]_2_ (10 mol %). Glacial acetic acid (100–300 µL) and alkene (3.0 equiv) were added. The tube was fitted with a septum and the mixture was heated to 80 °C for 15 h. After cooling to ambient temperature, the reaction mixture was diluted with toluene (1.0–5.0 mL) and purified by column chromatography or preparative thin layer chromatography.

### Ruthenium(II)-catalyzed on-resin C–H alkylation of peptides

A 10 mL conical-bottom test tube without a stir bar was charged with a peptide loaded resin (30 µmol) and [RuCl_2_(*p*-cymene)]_2_ (10.0 mol %). Glacial acetic acid (30–150 µL) and alkene (3.0–10.0 equiv) were added. The test tube was fitted with a septum and the mixture was heated to 80 °C for 15 h with a slight shaking of the reaction vial. After cooling to ambient temperature, the mixture was transferred with methanol to a 10 mL syringe equipped with a frit. The resin was washed with HOAc (5 × 5 mL), MeOH (3 × 5 mL), HOAc (3 × 5 mL), MeOH (3 × 5 mL), and CH_2_Cl_2_ (5 × 5 mL). After drying overnight at 40 °C, the resin was treated with TFA (3 mL) or TFA/TIS/H_2_O/DCM (3 mL, 95:2.5:1.25:1.25) at ambient temperature for 4 h. The cleavage solution was collected and the resin was washed with TFA (3 mL) and HOAc (3 × 5 mL). The combined phases were concentrated under reduced pressure and the peptide was three times precipitated from cold diethyl ether (– 20 °C, 5 mL), centrifuged and decanted. The obtained solid was dried under vacuum.

## Supplementary information

Supplementary Information

Description of Additional Supplementary Files

Supplementary Data 1

## Data Availability

All data are also available from the authors upon reasonable request.
