# Supplementary material for: Late-stage peptide C–H alkylation for bioorthogonal C–H activation featuring solid phase peptide synthesis
Source: Nat Commun. 2019 Aug 7;10:3553. doi: 10.1038/s41467-019-11395-3 (PMC6685959; doi:10.1038/s41467-019-11395-3)
Supplement: Supplementary file 1 — Supplementary Information [file 41467_2019_11395_MOESM1_ESM.pdf]

Late-Stage Peptide C–H Alkylation for Bioorthogonal C–H Activation Featuring Solid Phase Peptide Synthesis

Schischko et al.

Supplementary Information

Late-Stage Peptide C–H Alkylation for Bioorthogonal C–H Activation Featuring Solid Phase Peptide Synthesis

Alexandra Schischko,† Nikolaos Kaplaneris,† Torben Rogge, Giedre Sirvinskaite, Jongwoo Son, and Lutz Ackermann*

Institut für Organische und Biomolekulare Chemie

Georg-August-Universität, Tammannstrasse 2, D-37077 Göttingen (Germany)

Fax: +49/551-39-6777

E-mail: Lutz.Ackermann@chemie.uni-goettingen.de

Supplementary Tables

Supplementary Table 1. Optimization studies.

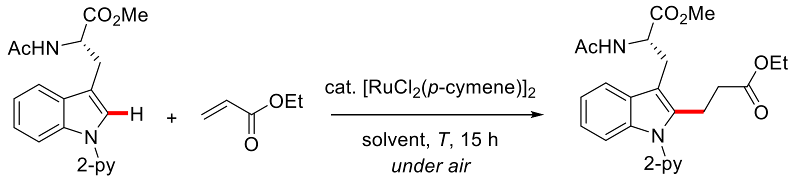

Entry	*T*/ °C	$[Ru]_2$/ mol %	solvent	Trp conc./ M	yield/ %[b]
1	120	10	HOAc	0.3	81[c]
2	120	10	GVL/HOAc 1:1	0.3	58[c]
3	120	10	GVL/NH_4Cl (5 M in H_2O) 1:1	0.3	13[c]
4	120	10	NH_4Cl (5 M in H_2O)	0.3	traces[c]
5	120	10	HOAc/KOAc in H_2O pH 4.0	0.3	traces[c]
6	120	10	HOAc/KOAc in H_2O pH 3.7	0.3	traces[c]
7	120	10	HOAc/*t*-amyl alcohol 1:9	0.3	traces[c]
8	120	10	HOAc/H_2O 1:9	0.3	traces[c]
9	120	10	HOAc/toluene 1:9	0.3	traces[c]
10	120	10	HOAc/*m*-xylene 1:9	0.3	traces[c]
11	120	10	HOAc/octane 1:9	0.3	47[c]
12	120	10	HOAc/DME 1:9	0.3	traces[c]
13	120	10	propionic acid	0.3	35[c]
14	120	10	pivalic acid	0.3	23[c]
15	120	10	citric acid/NaOH in H_2O pH 3.5	0.3	0[c]
16	120	10	$KHSO_4$ in H_2O pH 1.6	0.3	0[c]
17	100	10	HOAc	0.3	88
18	80	10	HOAc	0.3	73
**19**	**80**	**10**	**HOAc**	**1.0**	**90**
20	60	10	HOAc	1.0	80
21	38	10	HOAc	1.0	54
22	80	5.0	HOAc	1.0	83
23	80	2.5	HOAc	1.0	28
24	80	-	HOAc	1.0	0
25	80	10	HOAc/H_2O 3:1	1.0	75
26	80	10	HOAc/H_2O 1:1	1.0	60

[a] Reaction conditions: **1a** (150 µmol), **2a** (450 µmol), $[RuCl_2(p\text{-cymene})]_2$ (x mol %), solvent (450 µL or 150 µL), 15 h. [b] Yields refer to isolated compounds. [c] The reaction was carried out under N_2. GVL: γ-valerolactone; DME: dimethoxyethane.

Supplementary Methods

General Remarks: Catalytic reactions were carried out under air using ambient-temperature-air-dried glassware. *N*-Pyridyl tryptophans were prepared by modified literature procedures.[1-4] Di- to tetrapeptides were prepared by standard peptide-coupling methods in solution.[5-7] Peptides for on-resin reactions were synthesized by microwave assisted solid phase peptide synthesis (SPPS) using hydroxymethyl polystyrene resin (Novabiochem®), which was loaded with Fmoc-Val (0.63 mmol/g) according to standard literature procedures,[8] or Rink-Amide-ChemMatrix® resin (Biotage, 0.50 mmol/g) or Fmoc-Rink-Amide AM resin (Iris Biotech, 0.55 mmol/g). SPPS was carried out in a CEM Liberty Blue™. Precipitated peptides were centrifuged with a Heraeus Megafuge 16R from Thermo Scientific™ for 3 min at 9800 rounds/min. Chemicals were obtained from commercial sources, and were used without further purification. Yields refer to isolated compounds, estimated to be >95 % pure as determined by ^{1}H NMR and LC-MS. TLC and PTLC: Macherey-Nagel, TLC plates Alugram® Xtra Sil G/UV$_{254}$; detection under UV light at 254 nm. Chromatography: Separations were carried out on Merck Geduran Silica 60 (0.063–0.200 mm, 70–230 mesh ASTM). NMR: Spectra were recorded on a Varian Inova 600, Varian Inova 500, Bruker Avance III HD 500, Bruker Avance III HD 400, Bruker Avance III 400, Varian VNMRS 300, Bruker Avance 300, Varian Mercury VX 300, Varian Mercury 300, or Bruker Avance III 300 in the solvent indicated; chemical shifts (δ) refer to non-deuterated solvent resonances and are provided in ppm. All IR spectra were taken on a Bruker FT-IR Alpha device. MS: EI-MS: Jeol AccuTOF at 70 eV; ESI-MS: Bruker Daltonic maXis or microTOF. Melting points: Stuart® melting point apparatus SMP3, Barlworld Scientific, values are uncorrected. HPLC chromatograms were recorded on an Agilent 1260/1290 Infinity using the DAICEL CHIRALPAK® IC-3 column and hexanes/ethyl acetate (1:1, 1 mL/min, detection at 274 nm). LC-MS chromatograms were recorded on an Agilent 1260/1290 Infinity system with an Agilent 6100s Series Single Quad using the ZORBAX SB-C18 column, 5 µm. The flow rate was set to 0.5 mL/min, detection at 270 and 290 nm. The methods used are depicted in Supplementary Figure 1:

method length	t/min	acetonitrile (0.1% TFA)	water (0.1% TFA)
15 min	0	60	40
	5	100	0
	13	100	0
	15	60	40
22 min	0	60	40
	5	100	0
	20	100	0
	22	60	40

Supplementary Figure 1. LC-MS methods.

General procedure A: Ruthenium(II)-catalyzed C–H alkylation of tryptophan: A 10 mL conical-bottom test tube with a stir bar was charged with pyridylated tryptophan (150 µmol) and [$RuCl_2$(*p*-cymene)]$_2$ (9.2 mg, 10.0 mol %). Glacial acetic acid (150 µL) and acrylate or vinyl ketone (1.1–3.0 equiv.) were added. The tube was fitted with a septum and the mixture was heated to 80 °C for 15 h. After cooling to ambient temperature, the reaction mixture was diluted with toluene (5.0 mL) and purified by column chromatography.

General procedure B: Ruthenium(II)-catalyzed C–H alkylation of peptides: A 10 mL conical-bottom test tube with a stir bar was charged with pyridylated peptide (30–150 µmol) and [$RuCl_2$(*p*-cymene)]$_2$ (10 mol %). Glacial acetic acid (100–300 µL) and acrylate or vinyl ketone (3.0 equiv.) were added. The tube was fitted with a septum and the mixture was heated to 80 °C for 15 h. After cooling to ambient temperature, the reaction mixture was diluted with toluene (1.0–5.0 mL) and purified by column chromatography or PTLC.

General procedure C: Ruthenium(II)-catalyzed C–H ligation of peptides: A 10 mL conical-bottom test tube with a stir bar was charged with pyridylated peptide (30–150 µmol), acryloyl peptide (1.1 equiv.), [$RuCl_2$(*p*-cymene)]$_2$ (10 mol %) and glacial acetic acid (100–300 µL). The tube was fitted with a septum and the mixture was heated to 80 °C for 15 h. After cooling to ambient temperature, the reaction mixture was diluted with toluene (1.0–5.0 mL) and purified by column chromatography or PTLC.

General Procedure D: Traceless Removal of Directing Group: To a stirred solution of peptide (1.0 equiv) in CH_2Cl_2 (0.25 M) was added MeOTf (1.0 equiv) at 0 ˚C. After 30 min, the mixture was allowed to warm up to 25 °C and stirred for 18 h. The crude mixture was concentrated under reduced pressure to afford a bright yellow solid. In a sealed-tube, the crude product, $Pd(OH)_2$/C (20 wt%, 0.1 equiv) and ammonium formate (10.0 equiv) were dissolved in ethanol (0.5 M) and stirred at 60 ˚C for 16 hours. The mixture was filtered through a short plug of celite, concentrated under reduced pressure and purified by column chromatography.

General procedure E: Ruthenium(II)-catalyzed on-resin C–H alkylation of peptides: A 10 mL conical-bottom test tube without a stir bar was charged with a peptide loaded resin (30 µmol) and $[RuCl_2(p\text{-cymene})]_2$ (10.0 mol %). Glacial acetic acid (30–150 µL) and acrylate or vinyl ketone (3.0–10.0 equiv.) were added. The test tube was fitted with a septum and the mixture was heated to 80 °C for 15 h with a slight shaking of the reaction vial. After cooling to ambient temperature, the mixture was transferred with methanol to a 10 mL syringe equipped with a frit. The resin was washed with acetic acid (5 × 5 mL), methanol (3 × 5 mL), acetic acid (3 × 5 mL), methanol (3 × 5 mL), and CH_2Cl_2 (5 × 5 mL). After drying overnight at 40 °C, the resin was treated with TFA (3 mL) or TFA/TIS/H_2O/DCM (3 mL, 95:2.5:1.25:1.25) at ambient temperature for 4 h. The cleavage solution was collected and the resin was washed with TFA (3 mL) and acetic acid (3 × 5 mL). The combined phases were concentrated under reduced pressure and the peptide was three times precipitated from cold diethyl ether (–20 °C, 5 mL), centrifuged and decanted. The obtained solid was dried under vacuum.

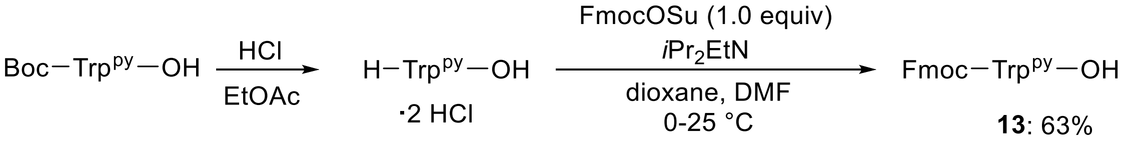

Supplementary Figure 2. Synthesis and Characterization Data for Fmoc-Trp^{py}-OH (**13**)

Boc-Trp^{py}-OH was synthesized according to literature procedures.[9] HCl (aq., 12 M, 3.0 equiv.) was added dropwise to a solution of Boc-Trp^{py}-OH (2.14 g, 5.6 mmol) in EtOAc (100 mL). After the product had precipitated, the solid was filtered off and dried in vacuum. The completion of the reaction was monitored by TLC.

A solution of FmocOSu (9-Fluorenylmethyl *N*-succinimidyl carbonate, 1.89 g, 5.6 mmol) in dioxane (20 mL) was added dropwise to a solution of H-Trp^{py}-OH·2HCl (1.98 g, 5.6 mmol) and *N*,*N*-diisopropylethylamine (9.7 mL, 56 mmol) in dioxane/DMF (1:1, 500 mL). The mixture was stirred at ambient temperature for 10 h. Then, the solvent was removed in vacuo and the product was purified by column chromatography (hexanes/EtOAc/HOAc 1:1:0.02, R_f=0.13). The product **13** was obtained as a white solid (1.78 g, 3.5 mmol, 63%). **m. p.** 93 °C. ^{1}**H NMR** (300 MHz, DMSO-d_6): δ 12.79 (br s, 1H), 8.54 (dd, *J* = 5.0, 1.8 Hz, 1H), 8.35 (d, *J* = 8.2 Hz, 1H), 7.94 (ddd, *J* = 9.0, 7.5, 1.9 Hz, 1H), 7.88 (s, 1H), 7.84 (d, *J* = 7.6 Hz, 2H), 7.77 (d, *J* = 8.3 Hz, 1H), 7.72–7.58 (m, 4H), 7.40–7.30 (m, 2H), 7.31–7.12 (m, 5H), 4.37 (ddd, *J* = 8.9, 8.9, 4.6 Hz, 1H), 4.25–4.11 (m, 3H), 3.30 (dd, *J* = 14.7, 4.7 Hz, 1H), 3.14 (dd, *J* = 14.7, 9.6 Hz, 1H). ^{13}**C NMR** (126 MHz, DMSO-d_6): δ 173.1 (C_q), 155.7 (C_q), 151.7 (C_q), 148.3 (CH), 143.5 (C_q), 140.4 (C_q), 138.8 (CH), 134.6 (C_q), 129.5 (C_q), 128.6 (CH), 127.9 (CH), 127.3 (CH), 126.7 (CH), 125.1 (CH), 122.8 (CH), 120.8 (CH), 119.8 (CH), 118.6 (CH), 114.4 (C_q), 113.7 (CH), 113.6 (CH), 65.6 (CH_2), 54.3 (CH), 46.5 (CH), 26.6 (CH_2). **IR** (ATR): 1713, 1592, 1472, 1453, 1437, 1318, 1219, 1050, 758, 736 cm^{-1}. **MS** (ESI) *m/z* (relative intensity) 1029 (30) $[2M+Na]^+$, 1007 (24) $[2M+H]^+$, 526 (100) $[M+Na]^+$, 504 (79) $[M+H]^+$. **HR-MS** (ESI) *m/z* calcd for $C_{31}H_{26}N_3O_4^+$ 504.1918 $[M+H]^+$, found 504.1906.

Studies on a potential Racenization: Tryptophan **1a** was partially racemized using a modified literature pyridylation procedure.[1] The ruthenium(II)-catalyzed C–H alkylation of *rac*-**1a** yielded partially racemic products *rac*-**3a**, *rac*-**3e** and *rac*-**3f**. HPLC analysis showed that no racemization occured during the ruthenium(II)-catalyzed C–H alkylation.

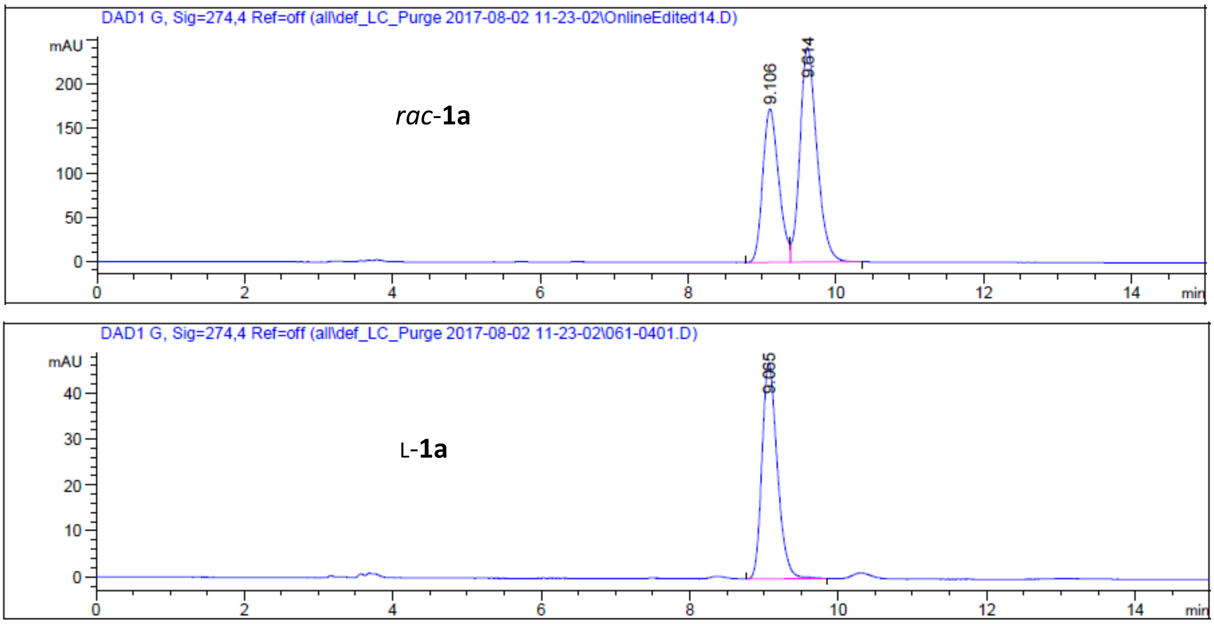

Supplementary Figure 3. HPLC-Chromatograms of the partially racemic mixture of the compound *rac*-**1a** and of starting material L-**1a**.

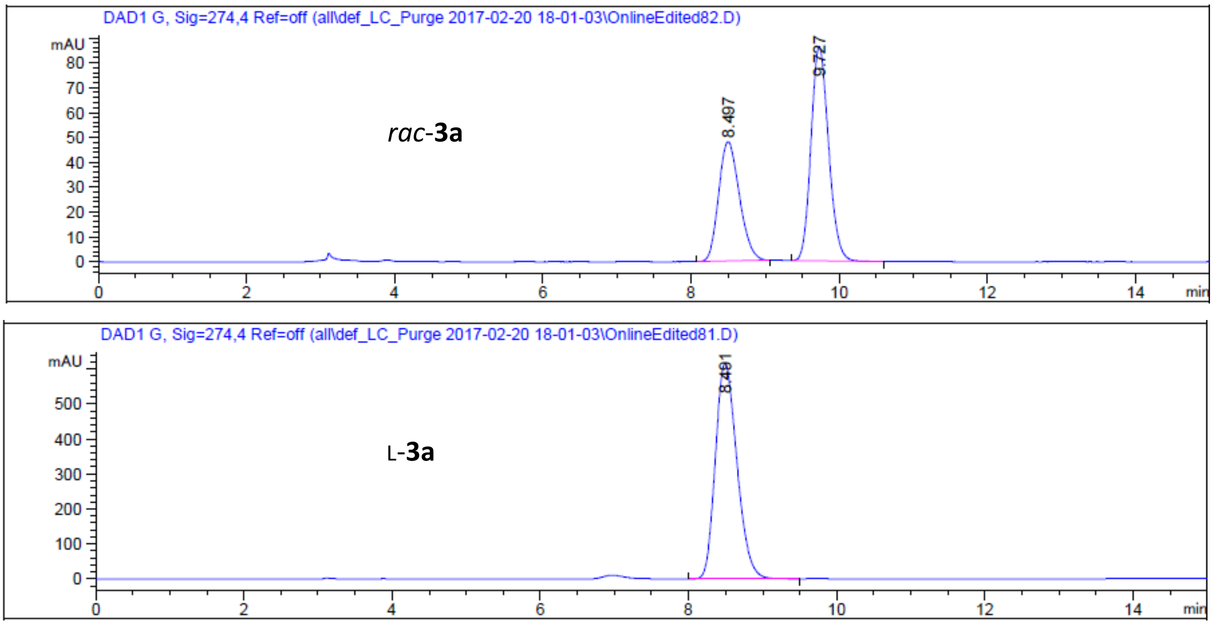

Supplementary Figure 4. HPLC-Chromatograms of the partially racemic mixture of the compound *rac*-**3a** and of isolated product L-**3a**.

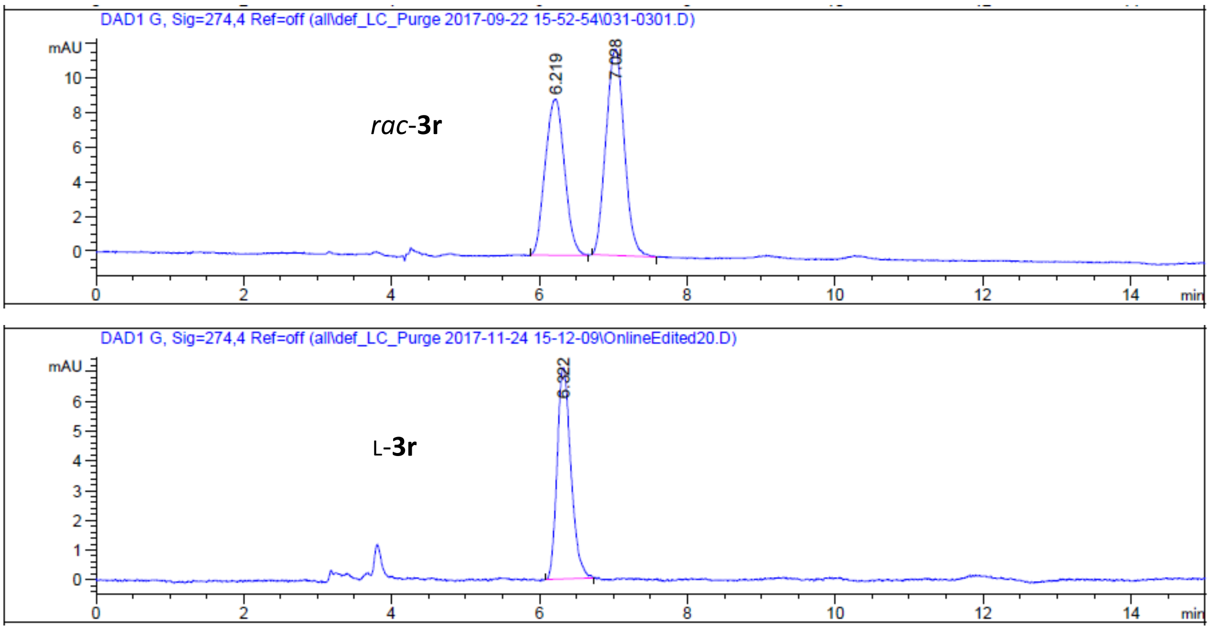

Supplementary Figure 5.HPLC-Chromatograms of the partially racemic mixture of the compound *rac*-**3r** and of isolated product L-**3r**.

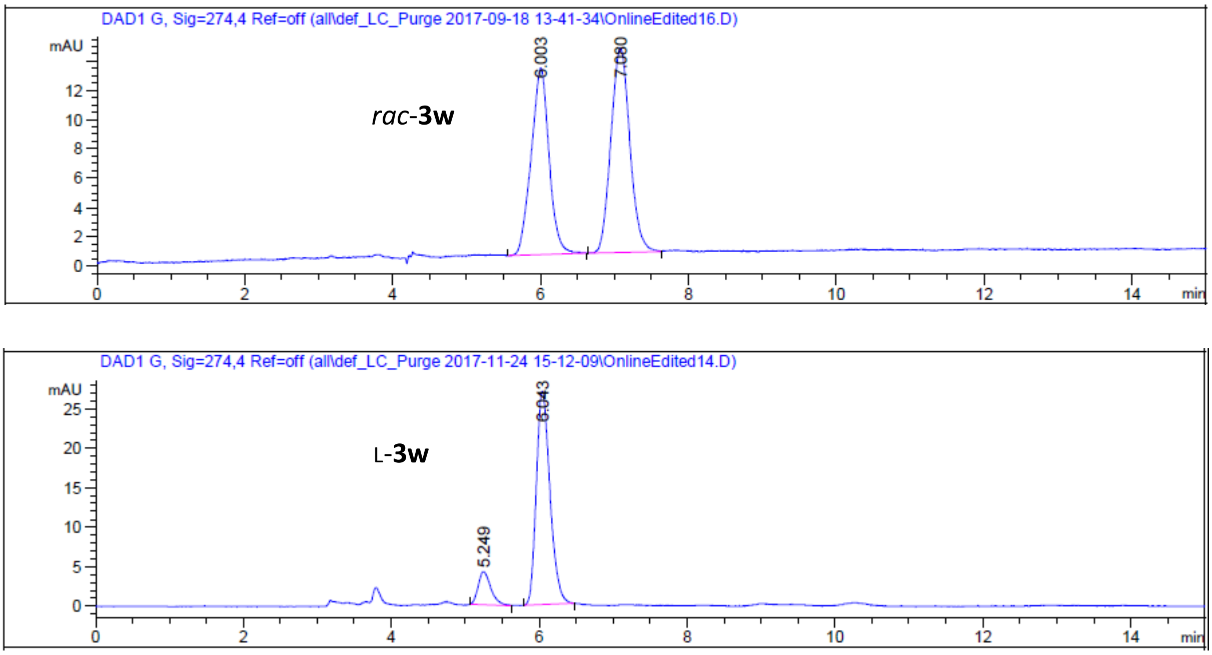

Supplementary Figure 6.HPLC-Chromatograms of the partially racemic mixture of the compound *rac*-**3w** and of isolated product L-**3w**.

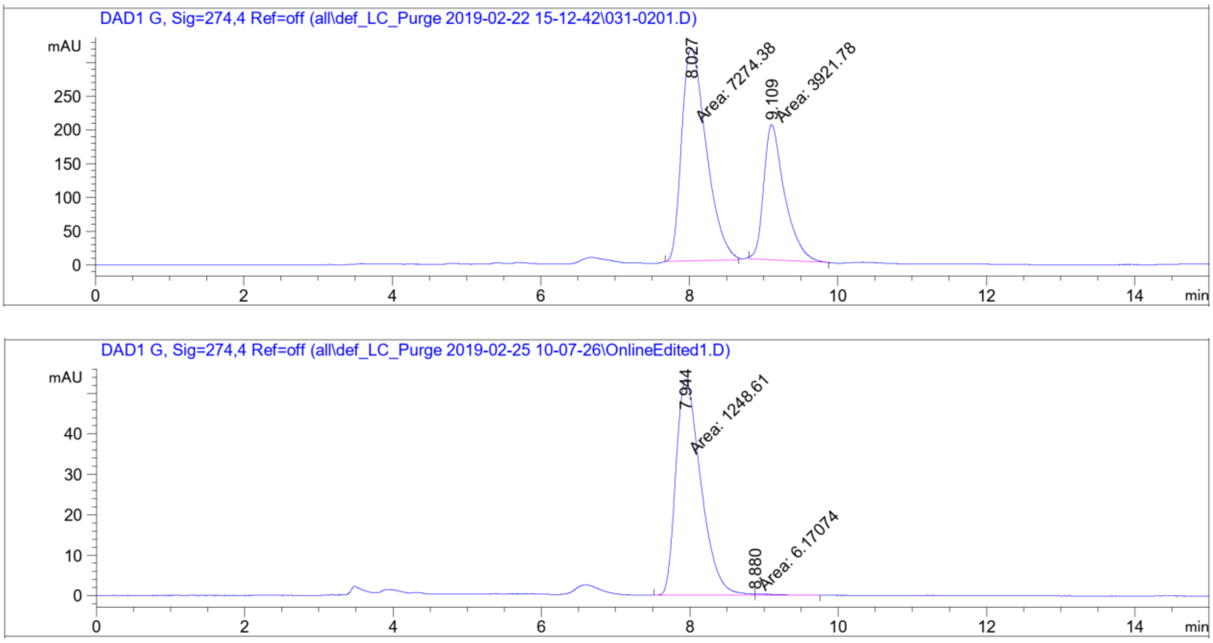

Supplementary Figure 7: HPLC-Chromatograms of the partially racemic mixture of the compound *rac*-**9a** and of isolated product L-**9a**.

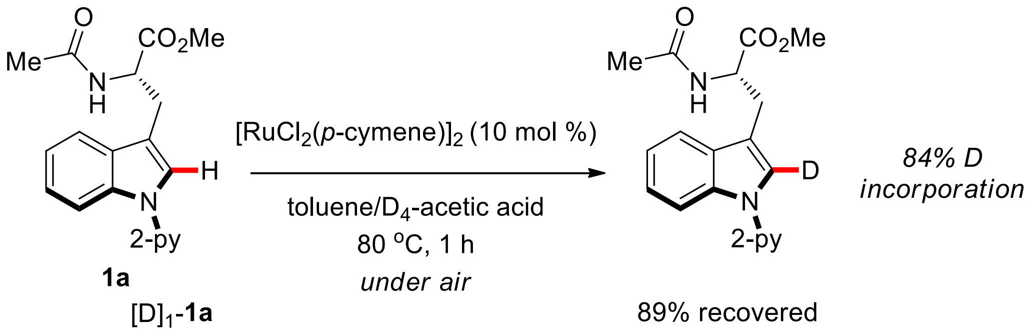

Supplementary Figure 8: H/D Exchange Studies.

A 10 mL conical-bottom test tube with a stir bar was charged with pyridylated tryptophan (150 µmol) and $[RuCl_2(p\text{-cymene})]_2$ (9.2 mg, 10.0 mol %). Toluene/D_4-acetic acid (1:1, 150 µL) were added. The tube was fitted with a septum and the mixture was heated to 80 °C for 1 h. After cooling to ambient temperature, the reaction mixture was diluted with toluene (5.0 mL). Purification by column chromatography (hexanes/EtOAc 1:1→1:2) yielded $[D]_1$-**1a** (45 mg, 134 µmol, 89%) as a white solid. The H/D exchange result was determined by ^{1}H-NMR spectroscopy.

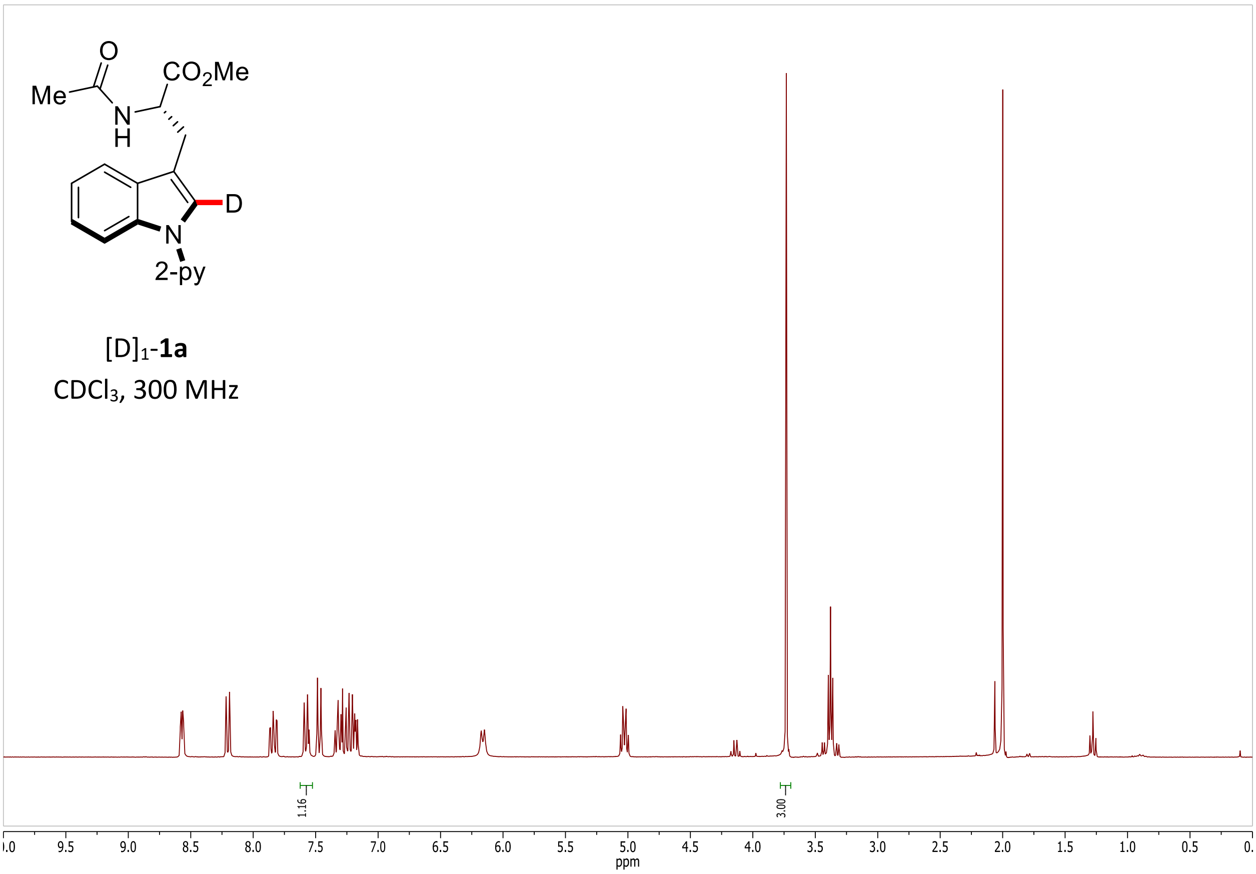

Supplementary Figure 9: ^{1}H-NMR of compound $[D]_1$-**1a**.

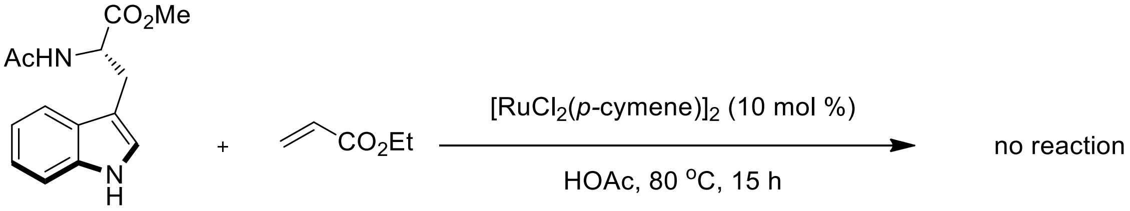

Supplementary Figure 10. Examination of *NH*-free tryptophan as a substrate.

A 10 mL conical-bottom test tube with a stir bar was charged with (*S*)-methyl 2-acetamido-3-(1*H*-indol-3-yl)propanoate (39 mg, 150 µmol) and $[RuCl_2(p\text{-cymene})]_2$ (9.2 mg, 10.0 mol %). Glacial acetic acid (150 µL) and ethyl acrylate (**2a**, 45 mg, 0.45 mmol) were added. The tube was fitted with a septum and the mixture was heated to 80 °C for 15 h. After cooling to ambient temperature, the reaction mixture was analyzed by LC-MS.

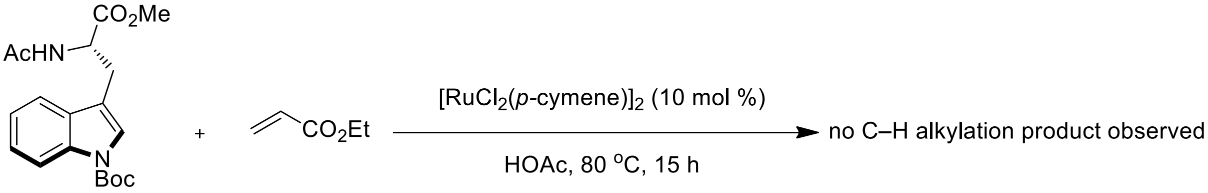

Supplementary Figure 11. Examination of *NH*-Boc tryptophan as a substrate.

A 10 mL conical-bottom test tube with a stir bar was charged with (*S*)-*tert*-butyl 3-(2-acetamido-3-methoxy-3-oxopropyl)-1*H*-indole-1-carboxylate (54 mg, 150 µmol) and $[RuCl_2(p\text{-cymene})]_2$ (9.2 mg, 10.0 mol %). Glacial acetic acid (150 µL) and ethyl acrylate (**2a**, 45 mg, 0.45 mmol) were added. The tube was fitted with a septum and the mixture was heated to 80 °C for 15 h. After cooling to ambient temperature, the reaction mixture was analyzed by LC-MS.

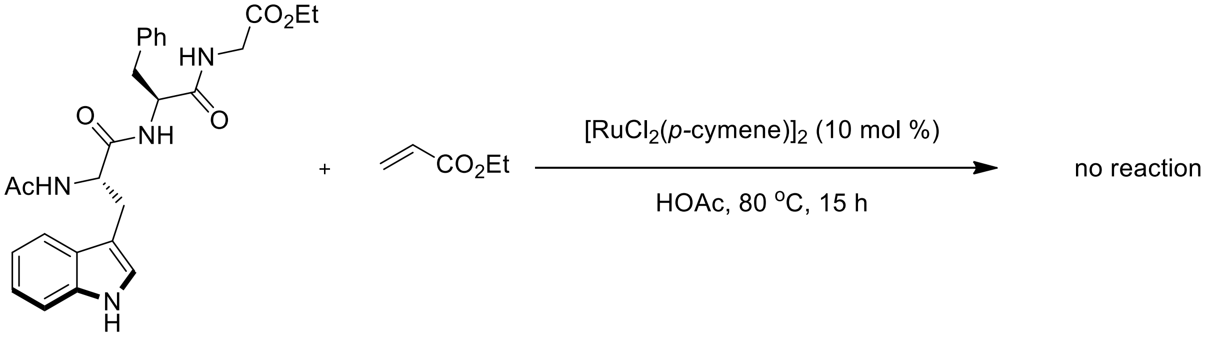

Supplementary Figure 12. Examination of petidic backbone as the directing group for the C–H alkylation

A 10 mL conical-bottom test tube with a stir bar was charged with ethyl 2-{[*S*]-2-[(*S*)-2-acetamido-3-(1*H*-indol-3-yl)propanamido]-3-phenylpropanamido}acetate (72 mg, 150 µmol) and $[RuCl_2(p\text{-cymene})]_2$ (9.2 mg, 10.0 mol %). Glacial acetic acid (300 µL) and ethyl acrylate (**2a**, 45 mg, 0.45 mmol) were added. The tube was fitted with a septum and the mixture was heated to 80 °C for 15 h. After cooling to ambient temperature, the reaction mixture was analyzed by LC-MS.

Characterization Data for Alkylated Amino Acids and Peptides

Methyl acetyl-1-(2-pyridyl)-2-(ethyl propionate-3-yl)-L-tryptophanate (3a):

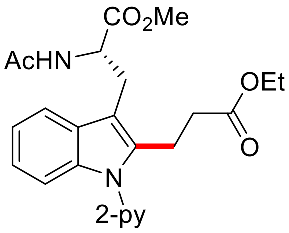

The general procedure A was followed using methyl acetyl-1-(2-pyridyl)-L-tryptophanate (**1a**, 50.6 mg, 150 µmol) and ethyl acrylate (**2a**, 45 mg, 0.45 mmol). Purification by column chromatography (EtOAc) yielded **3a** (59 mg, 0.14 mmol, 90%) as a colorless oil. **^{1}H NMR** (300 MHz, $CDCl_3$): δ 8.63 (dd, *J* = 4.9, 1.8 Hz, 1H), 7.91 (ddd, *J* = 7.7, 7.7, 1.9 Hz, 1H), 7.59–7.51 (m, 1H), 7.46 (d, *J* = 7.8 Hz, 1H), 7.34 (dd, *J* = 7.2, 5.1 Hz, 1H), 7.30–7.24 (m, 1H), 7.18–7.09 (m, 2H), 6.36 (d, *J* = 7.6 Hz, 1H), 4.96 (ddd, *J* = 7.7, 6.0, 6.0 Hz, 1H), 4.02 (q, *J* = 7.2 Hz, 2H), 3.70 (s, 3H), 3.38 (dd, *J* = 14.7, 6.0 Hz, 1H), 3.31 (dd, *J* = 14.7, 6.0 Hz, 1H), 3.27–3.13 (m, 2H), 2.29 (ddd, *J* = 9.0, 7.1, 2.5 Hz, 2H), 1.95 (s, 3H), 1.16 (t, *J* = 7.2 Hz, 3H). **^{13}C NMR** (126 MHz, $CDCl_3$): δ 172.4 (C_q), 172.2 (C_q), 169.7 (C_q), 150.9 (C_q), 149.4 (CH), 138.7 (CH), 136.8 (C_q), 136.6 (C_q), 128.5 (C_q), 122.4 (CH), 122.2 (CH), 120.9 (CH), 120.7 (CH), 118.5 (CH), 109.8 (CH), 109.4 (C_q), 60.5 (CH_2), 52.8 (CH), 52.3 (CH_3), 33.7 (CH_2), 27.1 8 (CH_2), 23.1 (CH_3), 20.3 (CH_2), 14.1 (CH_3). **IR** (ATR): 2954, 2927, 1731, 1655, 1470, 1459, 1436, 1369, 1176, 742 cm^{-1}. **MS** (ESI) *m/z* (relative intensity) 897 (24) $[2M+Na]^+$, 460 (79) $[M+Na]^+$, 438 (100) $[M+H]^+$. **HR-MS** (ESI) *m/z* calcd for $C_{24}H_{28}N_3O_5^+$ 438.2023 $[M+H]^+$, found 438.2026.

Methyl acetyl-1-(2-pyridyl)-2-(*n*-butyl propionate-3-yl)-L-tryptophanate (3b):

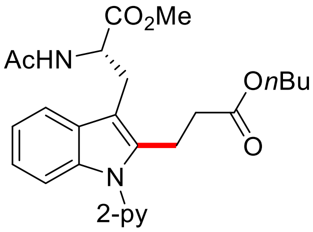

The general procedure A was followed using methyl acetyl-1-(2-pyridyl)-L-tryptophanate (**1a**, 50.6 mg, 150 µmol) and *n*-butyl acrylate (57 mg, 0.45 mmol). Purification by column chromatography (EtOAc) yielded **3b** (62 mg, 0.13 mmol, 90%) as a colorless oil. **^{1}H NMR** (300 MHz, $CDCl_3$): δ 8.64 (ddd, *J* = 4.9, 2.0, 0.8 Hz, 1H), 7.94 (ddd, *J* = 8.0, 7.4, 2.0 Hz, 1H), 7.59–7.52 (m, 1H), 7.48 (ddd, *J* = 8.0, 0.9, 0.9 Hz, 1H), 7.36 (ddd, *J* = 7.5, 4.9, 1.1 Hz, 1H), 7.30–7.26 (m, 1H), 7.18–7.10 (m, 2H), 6.32 (d, *J* = 7.8 Hz, 1H), 4.98 (ddd, *J* = 7.8, 6.0, 6.0 Hz, 1H), 3.97 (t, *J* = 6.7 Hz, 2H), 3.72 (s, 3H), 3.44–3.27 (m, 2H), 3.21 (td, *J* = 7.3, 4.5 Hz, 2H), 2.29 (ddd, *J* = 8.9, 7.0, 2.1 Hz, 2H), 1.96 (s, 3H), 1.58–1.43 (m, 2H), 1.35–1.19 (m, 2H), 0.87 (t, *J* = 7.3 Hz, 3H). **^{13}C NMR** (126 MHz, $CDCl_3$): δ 172.5 (C_q), 172.4 (C_q), 169.8 (C_q), 151.0 (C_q), 149.5 (CH), 138.6 (CH), 136.9 (C_q), 136.7 (C_q), 128.6 (C_q), 122.5 (CH), 122.3 (CH), 121.0 (CH), 120.8 (CH), 118.5 (CH), 109.9 (CH), 109.4 (C_q), 64.5 (CH_2), 52.8 (CH), 52.4 (CH_3),

33.8 (CH_2), 30.6 (CH_2), 27.1 (CH_2), 23.2 (CH_3), 20.4 (CH_2), 19.1 (CH_2), 13.7 (CH_3). **IR** (ATR): 2957, 2935, 1731, 1655, 1585, 1470, 1459, 1436, 1369, 1174, 1148, 742 cm^{-1}. **MS** (ESI) *m/z* (relative intensity) 953 (24) $[2M+Na]^+$, 488 (45) $[M+Na]^+$, 466 (100) $[M+H]^+$. **HR-MS** (ESI) *m/z* calcd for $C_{26}H_{32}N_3O_5^+$ 466.2336 $[M+H]^+$, found 466.2338.

Methyl acetyl-1-(2-pyridyl)-2-(2,2,2-trifluoroethyl propionate-3-yl)-L-tryptophanate (3c):

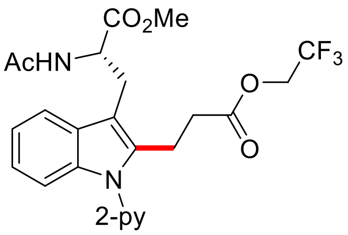

The general procedure A was followed using methyl acetyl-1-(2-pyridyl)-L-tryptophanate (**1a**, 50.6 mg, 150 µmol) and 2,2,2-trifluoroethyl acrylate (46 mg, 0.30 mmol). Purification by column chromatography (EtOAc) yielded **3c** (52 mg, 0.11 mmol, 71%) as a colorless oil. **^{1}H NMR** (300 MHz, $CDCl_3$): δ 8.63 (ddd, *J* = 4.9, 2.0, 0.8 Hz, 1H), 7.92 (ddd, *J* = 7.8, 7.4, 1.9 Hz, 1H), 7.59–7.50 (m, 1H), 7.48 (d, *J* = 8.0 Hz, 1H), 7.35 (ddd, *J* = 7.5, 4.9, 1.0 Hz, 1H), 7.30 (ddd, *J* = 6.0, 3.2, 0.7 Hz, 1H), 7.19–7.12 (m, 2H), 6.19 (d, *J* = 7.8 Hz, 1H), 4.97 (ddd, *J* = 8.0, 5.9, 5.9 Hz, 1H), 4.38 (q, *J* = 8.4 Hz, 2H), 3.69 (s, 3H), 3.38 (dd, *J* = 14.7, 6.3 Hz, 1H), 3.32 (dd, *J* = 14.7. 6.3 Hz, 1H), 3.28–3.11 (m, 2H), 2.55–2.45 (m, 2H), 1.96 (s, 3H). **^{13}C NMR** (126 MHz, $CDCl_3$): δ 172.4 (C_q), 170.7 (C_q), 169.6 (C_q), 151.0 (C_q), 149.6 (CH), 138.5 (CH), 136.6 (C_q), 136.2 (C_q), 128.6 (C_q), 122.7 (q, $^1J_{C\text{-}F}$ = 277.5 Hz, C_q, CF_3), 122.6 (CH), 122.3 (CH), 120.8 (CH), 120.7 (CH), 118.5 (CH), 110.0 (CH), 109.5 (C_q), 60.3 (q, $^2J_{C\text{-}F}$ = 36.6 Hz, CH_2), 52.9 (CH), 52.4 (CH_3), 33.3 (CH_2), 27.2 (CH_2), 23.2 (CH_3), 20.2 (CH_2). **^{19}F NMR** (282 MHz, $CDCl_3$): δ –73.77–(–77.85) (m). **IR** (ATR): 3292, 1749, 1652, 1541, 1472, 1459, 1437, 1368, 1267, 1139, 729 cm^{-1}. **MS** (ESI) *m/z* (relative intensity) 1005 (18) $[2M+Na]^+$, 514 (100) $[M+Na]^+$, 492 (33) $[M+H]^+$. **HR-MS** (ESI) *m/z* calcd for $C_{24}H_{25}F_3N_3O_5^+$ 492.1741 $[M+H]^+$, found 492.1734.

Methyl acetyl-1-(2-pyridyl)-2-(benzyl propionate-3-yl)-L-tryptophanate (3d):

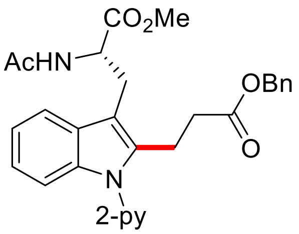

The general procedure A was followed using methyl acetyl-1-(2-pyridyl)-L-tryptophanate (**1a**, 50.6 mg, 150 µmol) and benzyl acrylate (36 mg, 0.23 mmol). Purification by column chromatography (EtOAc) yielded **3d** (66 mg, 0.13 mmol, 89%) as a colorless oil. **^{1}H NMR** (300 MHz, $CDCl_3$): δ 8.60 (ddd, *J* = 4.9, 2.0, 0.8 Hz, 1H), 7.90 (ddd, *J* = 8.0, 7.4, 1.9 Hz, 1H), 7.57–7.51 (m, 1H), 7.41 (ddd, *J* = 8.0, 1.0, 1.0 Hz, 1H), 7.33 (ddd, *J* = 7.5, 4.9, 1.0 Hz, 1H), 7.30–7.23 (m, 4H), 7.21–7.12 (m, 4H), 6.27 (d, *J* =

7.8 Hz, 1H), 5.01 (s, 2H), 4.96 (ddd, *J* = 7.8, 6.0, 6.0 Hz, 1H), 3.67 (s, 3H), 3.35 (dd, *J* = 14.7, 6.0 Hz, 1H), 3.32–3.29 (m, 1H), 3.29–3.21 (m, 2H), 2.36 (ddd, *J* = 8.8, 7.0, 2.0 Hz, 2H), 1.94 (s, 3H). **^{13}C NMR** (76 MHz, $CDCl_3$): δ 172.5 (C_q), 172.2 (C_q), 169.9 (C_q), 150.9 (C_q), 149.5 (CH), 138.7 (CH), 136.7 (C_q), 136.6 (C_q), 135.6 (C_q), 128.5 (C_q), 128.4 (CH), 128.1 (CH), 128.0 (CH), 122.5 (CH), 122.3 (CH), 120.9 (CH), 120.8 (CH), 118.5 (CH), 109.9 (CH), 109.5 (C_q), 66.3 (CH_2), 52.7 (CH), 52.3 (CH_3), 33.6 (CH_2), 26.9 (CH_2), 23.0 (CH_3), 20.2 (CH_2). **IR** (ATR): 3060, 2951, 1732, 1655, 1470, 1458, 1436, 1212, 1161, 740 cm^{-1}. **MS** (ESI) *m/z* (relative intensity) 1021 (27) $[2M+Na]^+$, 522 (71) $[M+Na]^+$, 500 (100) $[M+H]^+$. **HR-MS** (ESI) *m/z* calcd for $C_{29}H_{30}N_3O_5^+$ 500.2180 $[M+H]^+$, found 500.2178.

Methyl acetyl-1-(2-pyridyl)-2-(4-methoxyphenyl propionate-3-yl)-L-tryptophanate (3e):

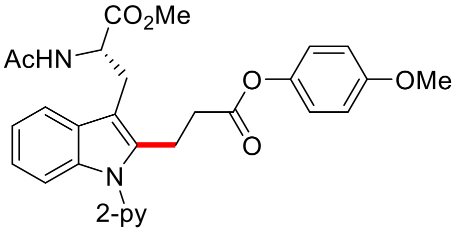

The general procedure A was followed using methyl acetyl-1-(2-pyridyl)-L-tryptophanate (**1a**, 50.6 mg, 150 µmol) and 4-methoxyphenyl acrylate (40 mg, 0.23 mmol). Purification by column chromatography (EtOAc) yielded **3e** (56 mg, 0.11 mmol, 73%) as a colorless oil. **^{1}H NMR** (600 MHz, $CDCl_3$): δ 8.66 (ddd, *J* = 4.9, 1.9, 0.9 Hz, 1H), 7.94 (ddd, *J* = 7.2, 7.2, 1.8 Hz, 1H), 7.60–7.54 (m, 1H), 7.51 (d, *J* = 8.0 Hz, 1H), 7.37 (dd, *J* = 7.5, 4.9 Hz, 1H), 7.33–7.29 (m, 1H), 7.19–7.15 (m, 2H), 6.87–6.78 (m, 4H), 6.28 (d, *J* = 7.8 Hz, 1H), 4.98 (ddd, *J* = 8.0, 5.9, 5.9 Hz, 1H), 3.76 (s, 3H), 3.70 (s, 3H), 3.40 (dd, *J* = 15.1, 6.2 Hz, 1H), 3.37–3.31 (m, 2H), 3.31–3.26 (m, 1H), 2.62–2.51 (m, 2H), 1.90 (s, 3H). **^{13}C NMR** (126 MHz, $CDCl_3$): δ 172.4 (C_q), 171.3 (C_q), 169.8 (C_q), 157.1 (C_q), 151.0 (C_q), 149.6 (CH), 143.8 (C_q), 138.6 (CH), 136.7 (C_q), 136.4 (C_q), 128.6 (C_q), 122.5 (CH), 122.3 (CH), 122.1 (CH), 120.9 (CH), 120.8 (CH), 118.5 (CH), 114.3 (CH), 109.9 (CH), 109.6 (C_q), 55.6 (CH_3), 52.8 (CH), 52.4 (CH_3), 33.8 (CH_2), 27.2 (CH_2), 23.1 (CH_3), 20.4 (CH_2). **IR** (ATR): 2952, 2927, 1742, 1654, 1505, 1470, 1459, 1436, 1369, 1190, 1135, 741 cm^{-1}. **MS** (ESI) *m/z* (relative intensity) 1053 (42) $[2M+Na]^+$, 538 (100) $[M+Na]^+$, 516 (71) $[M+H]^+$. **HR-MS** (ESI) *m/z* calcd for $C_{29}H_{30}N_3O_6^+$ 516.2129 $[M+H]^+$, found 516.2126.

Methyl acetyl-1-(2-pyridyl)-2-(4-chlorophenyl propionate-3-yl)-L-tryptophanate (3f):

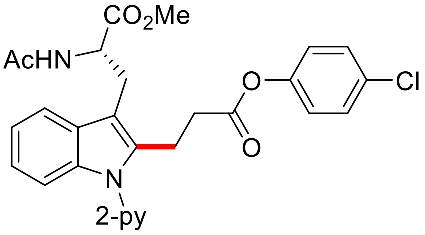

The general procedure A was followed using methyl acetyl-1-(2-pyridyl)-L-tryptophanate (**1a**, 50.6 mg, 150 µmol) and 4-chlorophenyl acrylate (41 mg, 0.23 mmol). Purification by column chromatography (EtOAc) yielded **3f** (50 mg, 96 µmol, 64%) as a white solid. **m. p.** 84 °C. **^{1}H NMR** (600 MHz, $CDCl_3$): δ 8.66 (ddd, *J* = 4.9, 2.0, 0.9 Hz, 1H), 7.94 (ddd, *J* = 7.7, 7.7, 2.0 Hz, 1H), 7.58–7.55 (m, 1H), 7.51 (ddd, *J* = 8.0, 0.9, 0.9 Hz, 1H), 7.36 (ddd, *J* = 7.5, 4.9, 1.0 Hz, 1H), 7.33–7.31 (m, 1H), 7.28 (dm, *J* = 8.8 Hz, 2H), 7.19–7.15 (m, 2H), 6.89 (dm, *J* = 8.8 Hz, 2H), 6.21 (d, *J* = 7.8 Hz, 1H), 4.97 (ddd, *J* = 7.9, 6.0, 6.0 Hz, 1H), 3.69 (s, 3H), 3.39 (dd, *J* = 14.8, 6.4 Hz, 1H), 3.37–3.31 (m, 2H), 3.31–3.26 (m, 1H), 2.67–2.55 (m, 2H), 1.92 (s, 3H). **^{13}C NMR** (126 MHz, $CDCl_3$): δ 172.4 (C_q), 170.7 (C_q), 169.7 (C_q), 151.1 (C_q), 149.7 (CH), 148.8 (C_q), 138.5 (CH), 136.6 (C_q), 136.3 (C_q), 131.1 (C_q), 129.3 (CH), 128.6 (C_q), 122.7 (CH), 122.6 (CH), 122.3 (CH), 120.8 (CH), 120.8 (CH), 118.5 (CH), 110.0 (CH), 109.6 (C_q), 52.9 (CH), 52.4 (CH_3), 33.9 (CH_2), 27.2 (CH_2), 23.2 (CH_3), 20.4 (CH_2). **IR** (ATR): 2953, 2925, 1741, 1654, 1468, 1471, 1459, 1436, 1197, 1133, 1086, 741 cm^{-1}. **MS** (ESI) *m/z* (relative intensity) 1061 (5) $[2M+Na]^+$, 542 (100) $[M+Na]^+$, 520 (69) $[M+H]^+$. **HR-MS** (ESI) *m/z* calcd for $C_{28}H_{27}{}^{35}ClN_3O_5^+$ 520.1634 $[M+H]^+$, found 520.1639.

Methyl acetyl-1-(2-pyridyl)-2-(4-bromophenyl propionate-3-yl)-L-tryptophanate (3g):

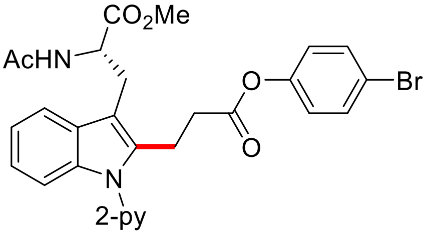

The general procedure A was followed using methyl acetyl-1-(2-pyridyl)-L-tryptophanate (**1a**, 50.6 mg, 150 µmol) and 4-bromophenyl acrylate (41 mg, 0.23 mmol). Purification by column chromatography (hexanes/EtOAc 1:1→1:3) yielded **3g** (61 mg, 96 µmol, 72%) as a white solid. **m. p.** 126 °C. **^{1}H NMR** (300 MHz, $CDCl_3$): δ 8.65 (ddd, *J* = 4.9, 2.0, 0.8 Hz, 1H), 7.93 (ddd, *J* = 8.0, 7.5, 2.0 Hz, 1H), 7.60–7.53 (m, 1H), 7.50 (ddd, *J* = 8.0, 1.0, 1.0 Hz, 1H), 7.43 (d, *J* = 8.9 Hz, 2H), 7.38–7.28 (m, 2H), 7.22–7.13 (m, 2H), 6.83 (d, *J* = 8.9 Hz, 2H), 6.25 (d, *J* = 7.8 Hz, 1H), 4.97 (ddd, *J* = 7.8, 6.0, 6.0 Hz, 1H), 3.69 (s, 3H), 3.55–3.18 (m, 4H), 2.65–2.57 (m, 2H), 1.92 (s, 3H). **^{13}C NMR** (126 MHz, $CDCl_3$): δ 172.4 (C_q), 170.5 (C_q), 169.6 (C_q), 151.1 (C_q), 149.6 (CH), 149.4 (C_q), 138.5 (CH), 136.6 (C_q), 136.3 (C_q), 132.3 (CH), 128.5 (C_q), 123.1 (CH), 122.6 (CH), 122.3 (CH), 120.8 (CH), 120.8 (CH), 118.8 (C_q), 118.5 (CH), 110.0 (CH), 109.6 (C_q), 52.9 (CH), 52.4 (CH_3), 33.9 (CH_2), 27.2 (CH_2), 23.1 (CH_3), 20.3 (CH_2). **IR** (ATR): 2928, 1745, 1654, 1585, 1471, 1437, 1199, 1134, 744 cm^{-1}. **MS** (ESI) *m/z* (relative intensity)

588/586 (100) [M+Na]$^+$, 566/564 (50) [M+H]$^+$. **HR-MS** (ESI) *m/z* calcd for $C_{28}H_{27}{}^{79}BrN_3O_5^+$ 564.1129 [M+H]$^+$, found 564.1130.

Methyl acetyl-1-(2-pyridyl)-2-(4-cyanophenyl propionate-3-yl)-L-tryptophanate (3h):

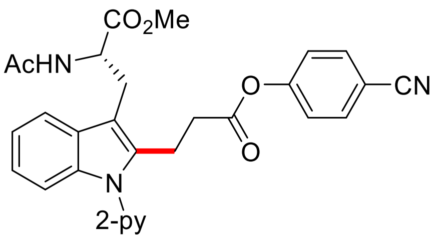

The general procedure A was followed using methyl acetyl-1-(2-pyridyl)-L-tryptophanate (**1a**, 50.6 mg, 150 µmol) and 4-cyanophenyl acrylate (40 mg, 0.23 mmol). Purification by column chromatography (hexanes/EtOAc 1:1→1:3) yielded **3h** (47 mg, 92 µmol, 61%) as a colorless oil. **^{1}H NMR** (300 MHz, $CDCl_3$): δ 8.66 (ddd, *J* = 4.9, 2.0, 0.8 Hz, 1H), 7.94 (ddd, *J* = 8.0, 7.5, 2.0 Hz, 1H), 7.63 (d, *J* = 8.8 Hz, 2H), 7.58–7.48 (m, 2H), 7.42–7.28 (m, 2H), 7.23–7.13 (m, 2H), 7.10 (d, *J* = 8.8 Hz, 2H), 6.20 (d, *J* = 7.9 Hz, 1H), 4.96 (ddd, *J* = 7.9, 6.0, 6.0 Hz, 1H), 3.68 (s, 3H), 3.40–3.25 (m, 4H), 2.69 (dd, *J* = 8.0, 7.0 Hz, 2H), 1.94 (s, 3H). **^{13}C NMR** (126 MHz, $CDCl_3$): δ 172.4 (C_q), 170.0 (C_q), 169.6 (C_q), 153.6 (C_q), 151.0 (C_q), 149.6 (CH), 138.6 (CH), 136.6 (C_q), 136.1 (C_q), 133.5 (CH), 128.6 (C_q), 122.7 (CH), 122.5 (CH), 122.3 (CH), 120.9 (CH), 120.8 (CH), 118.5 (CH), 118.1 (C_q), 110.0 (CH), 109.7 (C_q), 109.7 (C_q), 52.9 (CH), 52.4 (CH_3), 34.0 (CH_2), 27.3 (CH_2), 23.2 (CH_3), 20.3 (CH_2). **IR** (ATR): 2930, 2228, 1743, 1653, 1586, 1472, 1438, 1209, 1121, 744 cm^{-1}. **MS** (ESI) *m/z* (relative intensity) 533 (100) [M+Na]$^+$, 511 (69) [M+H]$^+$. **HR-MS** (ESI) *m/z* calcd for $C_{29}H_{27}N_4O_5^+$ 511.1976 [M+H]$^+$, found 511.1982.

Methyl acetyl-1-(2-pyridyl)-2-(4-acetylphenyl propionate-3-yl)-L-tryptophanate (3i):

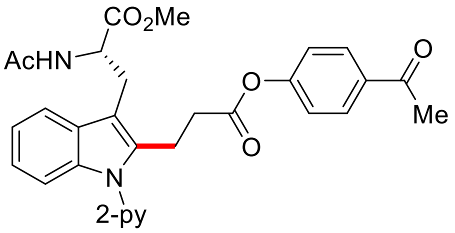

The general procedure A was followed using methyl acetyl-1-(2-pyridyl)-L-tryptophanate (**1a**, 50.6 mg, 150 µmol) and 4-acetylphenyl acrylate (42 mg, 0.23 mmol). Purification by column chromatography (EtOAc) yielded **3i** (55 mg, 0.11 mmol, 70%) as a colorless oil. **^{1}H NMR** (300 MHz, $CDCl_3$): δ 8.66 (ddd, *J* = 4.9, 2.0, 0.8 Hz, 1H), 7.93 (dm, *J* = 8.7 Hz, 3H), 7.59–7.53 (m, 1H), 7.50 (dd, *J* = 8.0, 1.0 Hz, 1H), 7.40–7.28 (m, 2H), 7.21–7.13 (m, 2H), 7.05 (dm, *J* = 8.7 Hz, 2H), 6.25 (d, *J* = 7.8 Hz, 1H), 4.97 (ddd, *J* = 7.9, 6.0, 6.0 Hz, 1H), 3.68 (s, 3H), 3.48–3.21 (m, 4H), 2.75–2.61 (m, 2H), 2.57 (s, 3H), 1.92 (s, 3H). **^{13}C NMR** (126 MHz, $CDCl_3$): δ 196.6 (C_q), 172.4 (C_q), 170.3 (C_q), 169.7 (C_q), 154.0 (C_q), 151.0 (C_q), 149.7 (CH), 138.5 (CH), 136.6 (C_q), 136.2 (C_q), 134.6 (C_q), 129.8 (CH), 128.5 (C_q), 122.6 (CH), 122.3 (CH), 121.5 (CH), 120.8 (CH), 120.8 (CH), 118.5 (CH), 110.0 (CH), 109.6 (C_q), 52.9 (CH), 52.4

(CH_3), 34.0 (CH_2), 27.2 (CH_2), 26.6 (CH_3), 23.1 (CH_3), 20.3 (CH_2). **IR** (ATR): 2952, 2927, 1745, 1680, 1585, 1471, 1459, 1436, 1265, 1201, 1119, 739 cm^{-1}. **MS** (ESI) *m/z* (relative intensity) 550 (100) [M+Na]$^+$, 528 (72) [M+H]$^+$. **HR-MS** (ESI) *m/z* calcd for $C_{30}H_{30}N_3O_6^+$ 528.2129 [M+H]$^+$, found 528.2136.

Methyl acetyl-1-(2-pyridyl)-2-(4-nitrophenyl propionate-3-yl)-L-tryptophanate (3j):

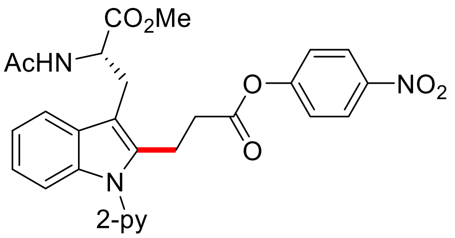

The general procedure A was followed using methyl acetyl-1-(2-pyridyl)-L-tryptophanate (**1a**, 50.6 mg, 150 µmol) and 4-nitrophenyl acrylate (44 mg, 0.23 mmol). Purification by column chromatography (hexanes/EtOAc 1:1→1:3) yielded **3j** (42 mg, 80 µmol, 53%) as a white solid. **m. p.** 102 °C. **^{1}H NMR** (300 MHz, $CDCl_3$): δ 8.66 (ddd, *J* = 4.9, 2.0, 0.8 Hz, 1H), 8.21 (d, *J* = 9.3 Hz, 2H), 7.94 (ddd, *J* = 8.0, 7.5, 2.0 Hz, 1H), 7.57–7.48 (m, 2H), 7.39–7.30 (m, 2H), 7.23–7.12 (m, 4H), 6.20 (d, *J* = 7.9 Hz, 1H), 4.96 (ddd, *J* = 7.9, 6.0, 6.0 Hz, 1H), 3.68 (s, 3H), 3.44–3.24 (m, 4H), 2.72 (dd, *J* = 8.2, 7.0 Hz, 2H), 1.95 (s, 3H). **^{13}C NMR** (126 MHz, $CDCl_3$): δ 172.4 (C_q), 170.0 (C_q), 169.6 (C_q), 155.1 (C_q), 151.1 (C_q), 149.7 (CH), 145.2 (C_q), 138.5 (CH), 136.6 (C_q), 136.1 (C_q), 128.6 (C_q), 125.0 (CH), 122.7 (CH), 122.3 (CH), 122.2 (CH), 120.9 (CH), 120.8 (CH), 118.5 (CH), 110.1 (CH), 109.7 (C_q), 53.0 (CH), 52.4 (CH_3), 34.1 (CH_2), 27.3 (CH_2), 23.2 (CH_3), 20.3 (CH_2). **IR** (ATR): 2923, 1743, 1653, 1589, 1522, 1458, 1437, 1370, 1347, 1156, 745 cm^{-1}. **MS** (ESI) *m/z* (relative intensity) 553 (100) [M+Na]$^+$, 531 (68) [M+H]$^+$. **HR-MS** (ESI) *m/z* calcd for $C_{28}H_{27}N_4O_7^+$ 531.1874 [M+H]$^+$, found 531.1881.

Methyl acetyl-1-(2-pyridyl)-2-(2-phenylphenyl propionate-3-yl)-L-tryptophanate (3k):

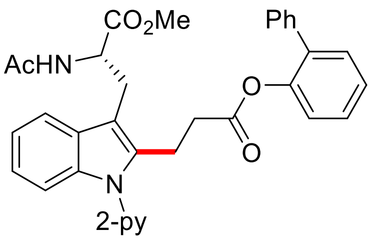

The general procedure A was followed using methyl acetyl-1-(2-pyridyl)-L-tryptophanate (**1a**, 50.6 mg, 150 µmol) and 2-phenylphenyl acrylate (52 mg, 0.23 mmol). Purification by column chromatography (hexanes/EtOAc 1:1→1:2) yielded **3k** (66 mg, 118 µmol, 79%) as a colorless oil. **^{1}H NMR** (300 MHz, $CDCl_3$): δ 8.57–8.52 (m, 1H), 7.87 (ddd, *J* = 7.8, 7.8, 1.9 Hz, 1H), 7.61–7.48 (m, 1H), 7.41–7.20 (m, 11H), 7.18–7.11 (m, 2H), 7.01–6.95 (m, 1H), 6.15 (d, *J* = 7.8 Hz, 1H), 4.93 (ddd, *J* = 7.8, 5.9, 5.9 Hz, 1H), 3.65 (s, 3H), 3.33 (dd, *J* = 14.8, 6.1 Hz, 1H), 3.23 (dd, *J* = 14.8, 5.9 Hz, 1H), 3.15–3.02 (m, 2H), 2.39 (t, *J* = 7.7 Hz, 2H), 1.85 (s, 3H). **^{13}C NMR** (126 MHz, $CDCl_3$): δ 172.4 (C_q), 170.8 (C_q), 169.7

(C_q), 151.0 (C_q), 149.6 (CH), 147.5 (C_q), 138.4 (CH), 137.4 (C_q), 136.6 (C_q), 136.4 (C_q), 134.7 (C_q), 130.7 (CH), 128.7 (CH), 128.6, (C_q) 128.4 (CH), 128.1 (CH), 127.3 (CH), 126.3 (CH), 122.6 (CH), 122.5 (CH), 122.2 (CH), 120.8 (CH), 120.7 (CH), 118.5 (CH), 110.0 (CH), 109.4 (C_q), 52.8 (CH), 52.4 (CH_3), 33.7 (CH_2), 27.1 (CH_2), 23.1 (CH_3), 20.2 (CH_2). **IR** (ATR): 3056, 2925, 1749, 1654, 1584, 1541, 1136, 701 cm^{-1}. **MS** (ESI) *m/z* (relative intensity) 584 (49) $[M+Na]^+$, 562 (100) $[M+H]^+$. **HR-MS** (ESI) *m/z* calcd for $C_{34}H_{32}N_3O_5^+$ 562.2336 $[M+H]^+$, found 562.2334.

Methyl acetyl-1-(2-pyridyl)-2-(2-trifluoromethylphenyl propionate-3-yl)-L-tryptophanate (3l):

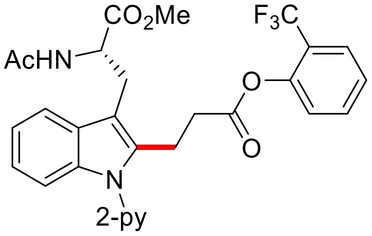

The general procedure A was followed using methyl acetyl-1-(2-pyridyl)-L-tryptophanate (**1a**, 50.6 mg, 150 µmol) and 2-(trifluoromethyl)phenyl acrylate (50 mg, 0.23 mmol). Purification by column chromatography (hexanes/EtOAc 1:1→1:2) yielded **3l** (62 mg, 112 µmol, 75%) as a colorless oil. **^{1}H NMR** (300 MHz, $CDCl_3$): δ 8.66 (ddd, *J* = 4.9, 2.0, 0.8 Hz, 1H), 7.93 (ddd, *J* = 8.0, 7.5, 2.0 Hz, 1H), 7.68–7.46 (m, 4H), 7.39–7.27 (m, 3H), 7.20–7.14 (m, 2H), 7.11 (d, *J* = 8.2 Hz, 1H), 6.24 (d, *J* = 7.9 Hz, 1H), 5.00 (ddd, *J* = 7.9, 5.9, 5.9 Hz, 1H), 3.70 (s, 3H), 3.49–3.21 (m, 4H), 2.70 (dd, *J* = 8.5, 7.3 Hz, 2H), 1.91 (s, 3H). **^{13}C NMR** (126 MHz, $CDCl_3$): δ 172.4 (C_q), 170.2 (C_q), 169.6 (C_q), 151.0 (C_q), 149.7 (CH), 147.8 (q, $^3J_{C\text{-}F}$ = 2.2 Hz, C_q), 138.5 (CH), 136.6 (C_q), 136.2 (C_q), 132.9 (CH), 128.6 (C_q), 126.7 (q, $^3J_{C\text{-}F}$ = 4.8 Hz, CH), 125.7 (CH), 124.3 (CH), 122.8 (q, $^1J_{C\text{-}F}$ = 272.3 Hz, C_q), 122.5 (CH), 122.4 (q, $^2J_{C\text{-}F}$ = 33.9 Hz, C_q), 122.3 (CH), 120.8 (CH), 120.7 (CH), 118.5 (CH), 110.0 (CH), 109.5 (C_q), 52.8 (CH_3), 52.4 (CH), 33.7 (CH_2), 27.2 (CH_2), 23.1 (CH_3), 20.3 (CH_2). **^{19}F NMR** (282 MHz, $CDCl_3$): δ –61.75 (s). **IR** (ATR): 3055, 2952, 1745, 1655, 1588, 1472, 1438 1371, 1131, 744 cm^{-1}. **MS** (ESI) *m/z* (relative intensity) 576 (78) $[M+Na]^+$, 554 (100) $[M+H]^+$. **HR-MS** (ESI) *m/z* calcd for $C_{29}H_{27}F_3N_3O_5^+$ 554.1897 $[M+H]^+$, found 554.1903.

Methyl acetyl-1-(2-pyridyl)-2-(3-methoxyphenyl propionate-3-yl)-L-tryptophanate (3m):

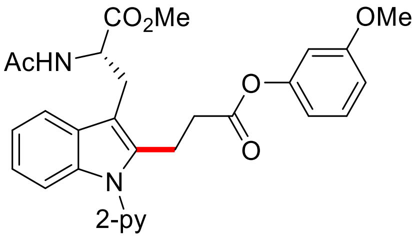

The general procedure A was followed using methyl acetyl-1-(2-pyridyl)-L-tryptophanate (**1a**, 50.6 mg, 150 µmol) and 3-methoxyphenyl acrylate (41 mg, 0.23 mmol). Purification by column chromatography (hexanes/EtOAc 1:1→1:3) yielded **3m** (56 mg, 108 µmol, 72%) as a colorless oil. **^{1}H NMR** (300 MHz,

$CDCl_3$): δ 8.67 (ddd, *J* = 4.9, 2.0, 0.9 Hz, 1H), 7.93 (ddd, *J* = 8.0, 7.5, 2.0 Hz, 1H), 7.61–7.54 (m, 1H), 7.51 (ddd, *J* = 8.0, 0.9, 0.9 Hz, 1H), 7.40–7.29 (m, 2H), 7.24–7.13 (m, 3H), 6.73 (ddd, *J* = 8.2, 2.4, 0.9 Hz, 1H), 6.53 (ddd, *J* = 8.2, 2.4, 0.9 Hz, 1H), 6.42 (dd, *J* = 2.4, 2.4 Hz, 1H), 6.24 (d, *J* = 7.7 Hz, 1H), 4.98 (ddd, *J* = 7.7, 6.0, 6.0 Hz, 1H), 3.71 (s, 3H), 3.70 (s, 3H), 3.48–3.21 (m, 4H), 2.70–2.45 (m, 2H), 1.89 (s, 3H). **^{13}C NMR** (126 MHz, $CDCl_3$): δ 172.5 (C_q), 170.8 (C_q), 169.7 (C_q), 160.3 (C_q), 151.3 (C_q), 151.1 (C_q), 149.7 (CH), 138.5 (CH), 136.7 (C_q), 136.3 (C_q), 129.6 (CH), 128.6 (C_q), 122.6 (CH), 122.3 (CH), 120.8 (CH), 120.8 (CH), 118.6 (CH), 113.5 (CH), 111.8 (CH), 110.0 (CH), 109.6 (C_q), 107.2 (CH), 55.4 (CH_3), 52.8 (CH), 52.4 (CH_3), 33.8 (CH_2), 27.2 (CH_2), 23.1 (CH_3), 20.4 (CH_2). **IR** (ATR): 2950, 1745, 1655, 1588, 1489, 1471, 1458, 1437, 1139, 744 cm^{-1}. **MS** (ESI) *m/z* (relative intensity) 538 (65) $[M+Na]^+$, 516 (100) $[M+H]^+$. **HR-MS** (ESI) *m/z* calcd for $C_{29}H_{30}N_3O_6^+$ 516.2129 $[M+H]^+$, found 516.2134.

Methyl acetyl-1-(2-pyridyl)-2-(4-fluorobenzyl propionate-3-yl)-L-tryptophanate (3n):

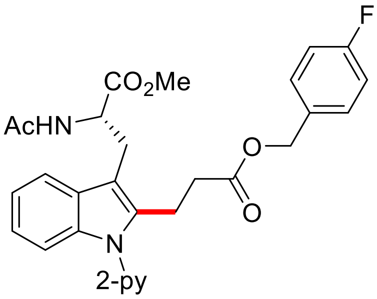

The general procedure A was followed using methyl acetyl-1-(2-pyridyl)-L-tryptophanate (**1a**, 50.6 mg, 150 µmol) and 4-fluorobenzyl acrylate (37 mg, 0.23 mmol). Purification by column chromatography (EtOAc) yielded **3n** (51 mg, 99 µmol, 66%) as a colorless oil. **^{1}H NMR** (500 MHz, $CDCl_3$): δ 8.60 (ddd, *J* = 4.9, 2.0, 0.9 Hz, 1H), 7.90 (ddd, *J* = 7.7, 7.7, 2.0 Hz, 1H), 7.56–7.52 (m, 1H), 7.40 (ddd, *J* = 7.9, 1.0, 1.0 Hz, 1H), 7.33 (ddd, *J* = 7.5, 4.9, 1.0 Hz, 1H), 7.29–7.26 (m, 1H), 7.20–7.13 (m, 4H), 6.98–6.90 (m, 2H), 6.25 (d, *J* = 7.8 Hz, 1H), 4.99–4.92 (m, 3H), 3.67 (s, 3H), 3.38–3.28 (m, 2H), 3.28–3.17 (m, 2H), 2.41–2.28 (m, 2H), 1.95 (s, 3H). **^{13}C NMR** (126 MHz, $CDCl_3$): δ 172.6 (C_q), 172.2 (C_q), 169.9 (C_q), 162.6 (d, $^1J_{C\text{-}F}$ = 247.2 Hz, C_q), 151.0 (C_q), 149.6 (CH), 138.7 (CH), 136.7 (C_q), 136.6 (C_q), 131.5 (d, $^4J_{C\text{-}F}$ = 3,2 Hz, C_q), 130.1 (d, $^3J_{C\text{-}F}$ = 8.3 Hz, CH), 128.6 (C_q), 122.6 (CH), 122.3 (CH), 121.0 (CH), 120.9 (CH), 118.6 (CH), 115.4 (d, $^2J_{C\text{-}F}$ = 21.6 Hz, CH), 110.0 (CH), 109.6 (C_q), 65.6 (CH_2), 52.8 (CH), 52.4 (CH_3), 33.6 (CH_2), 27.0 (CH_2), 23.1 (CH_3), 20.3 (CH_2). **^{19}F NMR** (471 MHz, $CDCl_3$): δ –113.48–(–113.56) (m). **IR** (ATR): 2953, 1732, 1656, 1586, 1511, 1471, 1459, 1436, 1370, 1221, 742 cm^{-1}. **MS** (ESI) *m/z* (relative intensity) 1057 (52) $[2M+Na]^+$, 540 (100) $[M+Na]^+$, 518 (17) $[M+H]^+$. **HR-MS** (ESI) *m/z* calcd for $C_{29}H_{29}FN_3O_5^+$ 518.2086 $[M+H]^+$, found 518.2081.

Methyl acetyl-1-(2-pyridyl)-2-(4-styryl propionate-3-yl)-L-tryptophanate (3o):

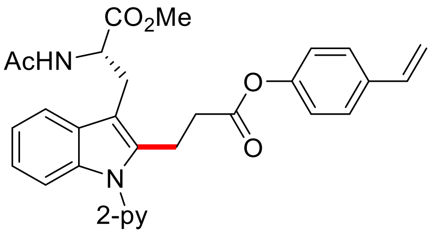

The general procedure A was followed using methyl acetyl-1-(2-pyridyl)-L-tryptophanate (**1a**, 50.6 mg, 150 µmol) and 4-styryl acrylate (34 mg, 0.20 mmol). Purification by column chromatography (EtOAc) yielded **3o** (42 mg, 83 µmol, 55%) as a white solid. **m. p.** 108 °C. **^{1}H NMR** (600 MHz, $CDCl_3$): δ 8.67 (ddd, *J* = 5.0, 2.0, 0.8 Hz, 1H), 7.94 (ddd, *J* = 7.7, 7.7, 2.0 Hz, 1H), 7.60–7.54 (m, 1H), 7.51 (ddd, *J* = 8.0, 1.0, 1.0 Hz, 1H), 7.37 (ddd, *J* = 7.5, 4.9, 1.1 Hz, 1H), 7.35 (dm, *J* = 8.6 Hz, 2H), 7.32–7.29 (m, 1H), 7.19–7.15 (m, 2H), 6.89 (dm, *J* = 8.6 Hz, 2H), 6.66 (dd, *J* = 17.6, 10.9 Hz, 1H), 6.29 (d, *J* = 7.8 Hz, 1H), 5.67 (dd, *J* = 17.6, 0.8 Hz, 1H), 5.22 (dd, *J* = 10.8, 0.8 Hz, 1H), 4.99 (ddd, *J* = 7.8, 5.9, 5.9 Hz, 1H), 3.70 (s, 3H), 3.43–3.26 (m, 4H), 2.64–2.53 (m, 2H), 1.90 (s, 3H). **^{13}C NMR** (126 MHz, $CDCl_3$): δ 172.4 (C_q), 170.9 (C_q), 169.9 (C_q), 151.1 (C_q), 149.9 (C_q), 149.7 (CH), 138.6 (CH), 136.7 (C_q), 136.4 (C_q), 135.7 (CH), 135.3 (C_q), 128.6 (C_q), 127.0 (CH), 122.6 (CH), 122.3 (CH), 121.4 (CH), 120.9 (CH), 120.8 (CH), 118.5 (CH), 114.0 (CH_2), 110.0 (CH), 109.6 (C_q), 52.9 (CH), 52.5 (CH_3), 33.9 (CH_2), 27.2 (CH_2), 23.1 (CH_3), 20.4 (CH_2). **IR** (ATR): 3265, 2934, 1743, 1655, 1586, 1505, 1470, 1459, 1436, 1368, 1197, 1166, 1135, 782, 742 cm^{-1}. **MS** (ESI) *m/z* (relative intensity) 1045 (100) $[2M+Na]^+$, 534 (99) $[M+Na]^+$, 512 (15) $[M+H]^+$. **HR-MS** (ESI) *m/z* calcd for $C_{30}H_{30}N_3O_5^+$ 512.2180 $[M+H]^+$, found 512.2172.

Methyl acetyl-1-(2-pyridyl)-2-(5-pent-1-ene propionate-3-yl)-L-tryptophanate (3p):

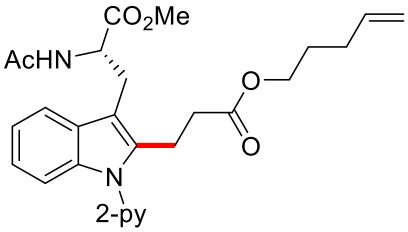

The general procedure A was followed using methyl acetyl-1-(2-pyridyl)-L-tryptophanate (**1a**, 50.6 mg, 150 µmol) and pent-4-en-1-yl acrylate (42 mg, 0.30 mmol). Purification by column chromatography (EtOAc) yielded **3p** (54 mg, 0.11 mmol, 75%) as a colorless oil. **^{1}H NMR** (600 MHz, $CDCl_3$): δ 8.62–8.66 (m, 1H), 7.92 (ddd, *J* = 8.0, 7.4, 2.0 Hz, 1H), 7.58–7.52 (m, 1H), 7.47 (d, *J* = 8.0 Hz, 1H), 7.35 (ddd, *J* = 7.5, 4.9, 1.0 Hz, 1H), 7.31–7.27 (m, 1H), 7.19–7.11 (m, 2H), 6.27 (d, *J* = 7.9 Hz, 1H), 5.74 (dddd, *J* = 16.9, 10.2, 6.6, 6.6 Hz, 1H), 5.00–4.91 (m, 3H), 4.02–3.95 (m, 2H), 3.71 (s, 3H), 3.37 (dd, *J* = 14.9, 6.2 Hz, 1H), 3.32 (dd, *J* = 14.9, 6.2 Hz, 1H), 3.26–3.15 (m, 2H), 2.36–2.26 (m, 2H), 2.05–2.00 (m, 2H), 1.96 (s, 3H), 1.66–1.60 (m, 2H). **^{13}C NMR** (126 MHz, $CDCl_3$): δ 172.4 (C_q), 172.3 (C_q), 169.7 (C_q), 151.0 (C_q), 149.5 (CH), 138.5 (CH), 137.2 (CH), 136.8 (C_q), 136.6 (C_q), 128.5 (C_q), 122.4 (CH), 122.2 (CH), 120.9 (CH), 120.7 (CH), 118.5 (CH), 115.2 (CH_2), 109.9 (CH), 109.4 (C_q), 64.0

(CH_2), 52.8 (CH), 52.4 (CH_3), 33.7 (CH_2), 29.9 (CH_2), 27.7 (CH_2), 27.1 (CH_2), 23.1 (CH_3), 20.3 (CH_2). **IR** (ATR): 3274, 2952, 1732, 1655, 1470, 1459, 1436, 1368, 1167, 742 cm^{-1}. **MS** (ESI) *m/z* (relative intensity) 977 (6) $[2M+Na]^+$, 500 (91) $[M+Na]^+$, 478 (100) $[M+H]^+$. **HR-MS** (ESI) *m/z* calcd for $C_{27}H_{32}N_3O_5^+$ 478.2336 $[M+H]^+$, found 478.2342.

Methyl acetyl-1-(2-pyridyl)-2-(2-hydroxyethyl propionate-3-yl)-L-tryptophanate (3q):

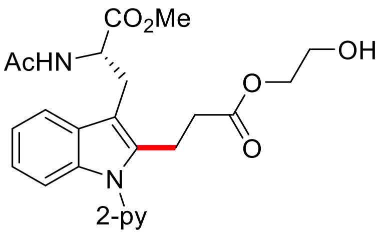

The general procedure A was followed using methyl acetyl-1-(2-pyridyl)-L-tryptophanate (**1a**, 50.6 mg, 150 µmol) and 2-hydroxyethyl acrylate (34 mg, 0.30 mmol). Purification by column chromatography (EtOAc) yielded **3q** (34 mg, 77 µmol, 51%) as a colorless oil. ^{1}**H NMR** (300 MHz, $CDCl_3$): δ 7.88 (ddd, *J* = 7.7, 7.7, 2.0 Hz, 1H), 7.47–7.37 (m, 2H), 7.31 (ddd, *J* = 7.5, 4.9, 1.0 Hz, 1H), 7.26–7.18 (m, 1H), 7.13–7.05 (m, 2H), 6.67 (d, *J* = 6.9 Hz, 1H), 4.84 (ddd, *J* = 7.7, 6.2, 6.2 Hz, 1H), 4.03 (ddd, *J* = 5.8, 2.6, 1.5 Hz, 2H), 3.66–3.53 (m, 2H), 3.59 (s, 3H), 3.34–3.25 (m, 2H), 3.25–3.15 (m, 2H), 2.31 (dd, *J* = 7.8, 1.9 Hz, 2H), 1.93 (s, 3H). ^{13}**C NMR** (126 MHz, $CDCl_3$): δ 172.4 (C_q), 172.2 (C_q), 170.3 (C_q), 150.8 (C_q), 149.5 (CH), 138.6 (CH), 136.5 (C_q), 136.5 (C_q), 128.2 (C_q), 122.3 (CH), 122.3 (CH), 120.9 (CH), 120.6 (CH), 118.1 (CH), 109.9 (CH), 109.4 (C_q), 66.3 (CH_2), 60.4 (CH_2), 53.1 (CH), 52.4 (CH_3), 33.9 (CH_2), 27.2 (CH_2), 22.9 (CH_3), 20.5 (CH_2). **IR** (ATR): 3292, 2953, 1733, 1654, 1586, 1471, 1458, 1436, 1370, 1223, 1167, 743 cm^{-1}. **MS** (ESI) *m/z* (relative intensity) 929 (12) $[2M+Na]^+$, 476 (100) $[M+Na]^+$, 454 (75) $[M+H]^+$. **HR-MS** (ESI) *m/z* calcd for $C_{24}H_{28}N_3O_6^+$ 454.1973 $[M+H]^+$, found 454.1971.

Methyl acetyl-1-(2-pyridyl)-2-[(2-ethyl)*n*-hexyl propionate-3-yl]-L-tryptophanate (3r):

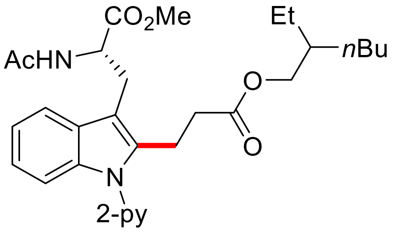

The general procedure A was followed using methyl acetyl-1-(2-pyridyl)-L-tryptophanate (**1a**, 50.6 mg, 150 µmol) and 2-ethylhexyl acrylate (55 mg, 0.30 mmol). Purification by column chromatography (EtOAc) yielded **3r** (58 mg, 0.11 mmol, 74%) as a colorless oil. ^{1}**H NMR** (600 MHz, $CDCl_3$): δ 8.65–8.61 (m, 1H), 7.93 (ddd, *J* = 7.7, 7.7, 1.9 Hz, 1H), 7.57–7.52 (m, 1H), 7.47 (dd, *J* = 7.9, 1.1 Hz, 1H), 7.37–7.33 (m, 1H), 7.29–7.25 (m, 1H), 7.18–7.11 (m, 2H), 6.34 (d, *J* = 7.8 Hz, 1H), 4.97 (ddd, *J* = 7.8, 6.0, 6.0 Hz, 1H), 3.93–3.84 (m, 2H), 3.71 (s, 3H), 3.37 (dd, *J* = 14.8, 6.0 Hz, 1H), 3.32 (dd, *J* = 14.8, 6.0 Hz, 1H), 3.27–3.15 (m, 2H), 2.35–2.25 (m, 2H), 1.96 (s, 3H), 1.50–1.43 (m, 1H), 1.29–1.21 (m, 4H),

1.21–1.17 (m, 4H), 0.86 (t, *J* = 7.0 Hz, 3H), 0.81 (t, *J* = 7.5 Hz, 3H). ^{13}**C NMR** (126 MHz, $CDCl_3$): δ 172.5 (C_q), 172.4 (C_q), 169.8 (C_q), 151.0 (C_q), 149.5 (CH), 138.5 (CH), 136.8 (C_q), 136.7 (C_q), 128.6 (C_q), 122.4 (CH), 122.2 (CH), 120.9 (CH), 120.7 (CH), 118.5 (CH), 109.9 (CH), 109.3 (C_q), 67.0 (CH_2), 52.8 (CH), 52.4 (CH_3), 38.6 (CH), 33.7 (CH_2), 30.3 (CH_2), 28.9 (CH_2), 27.1 (CH_2), 23.7 (CH_2), 23.1 (CH_3), 23.0 (CH_2), 20.4 (CH_2), 14.1 (CH_3), 11.0 (CH_3). **IR** (ATR): 2957, 2929, 2859, 1732, 1657, 1471, 1459, 1437, 1370, 1173, 1148, 732 cm^{-1}. **MS** (ESI) *m/z* (relative intensity) 1065 (53) $[2M+Na]^+$, 544 (62) $[M+Na]^+$, 522 (100) $[M+H]^+$. **HR-MS** (ESI) *m/z* calcd for $C_{30}H_{40}N_3O_5^+$ 522.2962 $[M+H]^+$, found 522.2960.

Methyl acetyl-1-(2-pyridyl)-2-{2-[2-(2-methoxyethoxy)ethoxy]ethyl propionate-3-yl}-L-tryptophanate (3s):

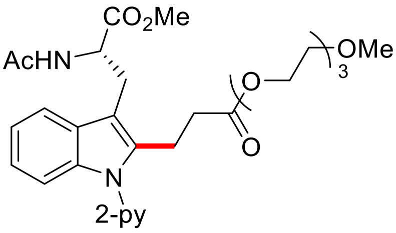

The general procedure A was followed using methyl acetyl-1-(2-pyridyl)-L-tryptophanate (**1a**, 50.6 mg, 150 µmol) and 2-[2-(2-methoxyethoxy)ethoxy]ethyl acrylate (49 mg, 0.23 mmol). Purification by column chromatography (EtOAc) yielded **3s** (62 mg, 0.11 mmol, 74%) as a colorless oil. ^{1}**H NMR** (300 MHz, $CDCl_3$): δ 8.63 (ddd, *J* = 4.9, 2.0, 0.8 Hz, 1H), 7.92 (ddd, *J* = 7.5, 7.5, 2.1 Hz 1H), 7.58–7.49 (m, 1H), 7.47 (ddd, *J* = 8.0, 1.0, 1.0 Hz, 1H), 7.34 (ddd, *J* = 7.5, 4.9, 1.0 Hz, 1H), 7.30–7.23 (m, 1H), 7.18–7.09 (m, 2H), 6.35 (d, *J* = 7.7 Hz, 1H), 4.96 (ddd, *J* = 7.8, 6.0, 6.0 Hz, 1H), 4.16–4.10 (m, 2H), 3.70 (s, 3H), 3.63–3.56 (m, 8H), 3.56–3.50 (m, 2H), 3.36 (s, 3H), 3.35–3.31 (m, 2H), 3.28–3.13 (m, 2H), 2.34 (ddd, *J* = 8.7, 6.9, 1.9 Hz, 2H), 1.95 (s, 3H). ^{13}**C NMR** (126 MHz, $CDCl_3$): δ 172.5 (C_q), 172.3 (C_q), 169.8 (C_q), 151.1 (C_q), 149.7 (CH), 138.5 (CH), 136.7 (C_q), 136.7 (C_q), 128.5 (C_q), 122.4 (CH), 122.2 (CH), 120.9 (CH), 120.7 (CH), 118.5 (CH), 109.9 (CH), 109.3 (C_q), 71.9 (CH_2), 70.5 (CH_2), 70.5 (CH_2), 70.5 (CH_2), 69.0 (CH_2), 63.7 (CH_2), 59.0 (CH_3), 52.8 (CH), 52.4 (CH_3), 33.6 (CH_2), 27.1 (CH_2), 23.1 (CH_3), 20.3 (CH_2). **IR** (ATR): 3289, 2947, 2876, 1733, 1658, 1584, 1568, 1532, 1474, 1459, 1437, 1369, 1177, 1104, 744 cm^{-1}. **MS** (ESI) *m/z* (relative intensity) 1133 (3) $[2M+Na]^+$, 578 (100) $[M+Na]^+$, 556 (55) $[M+H]^+$. **HR-MS** (ESI) *m/z* calcd for $C_{29}H_{38}N_3O_8^+$ 556.2653 $[M+H]^+$, found 556.2646.

(*S*)-2-Acetamido-3-{2-[3-(benzyloxy)-3-oxopropyl]-1-[pyridin-2-yl]-1*H*-indol-3-yl}propanoic acid (3t):

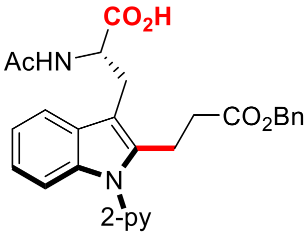

The general procedure A was followed using methyl N^{α}-acetyl-1-(pyridin-2-yl)-*L*-tryptophan (48.5 mg, 150 µmol) and benzyl acrylate (37 mg, 0.23 mmol). Purification by column chromatography (CH_2Cl_2/MeOH 30:1→20:1) yielded **3t** (40 mg, 83 µmol, 55%) as a white solid. **m.p.** 110 °C. **^{1}H NMR** (400 MHz, $CDCl_3$): δ 8.67 (dd, J = 5.0, 1.7 Hz, 1H), 8.03–7.90 (m, 1H), 7.56 (dd, J = 6.9, 1.8 Hz, 1H), 7.46–7.33 (m, 2H), 7.31–7.21 (m, 3H), 7.20–7.11 (m, 2H), 7.13–6.95 (m, 4H), 5.11–4.84 (m, 3H), 3.27–2.85 (m, 4H), 2.34–2.21 (m, 2H), 1.85 (s, 3H). **^{13}C NMR** (101 MHz, $CDCl_3$): δ 174.2 (C_q), 173.2 (C_q), 170.8 (C_q), 150.9 (C_q), 149.4 (CH), 139.7 (CH), 137.0 (C_q), 136.9 (C_q), 135.5 (C_q), 128.7 (CH), 128.6 (C_q), 128.5 (CH), 128.3 (CH), 123.1 (CH), 122.6 (CH), 122.2 (CH), 120.8 (CH), 119.0 (CH), 109.9 (C_q), 109.8 (CH), 66.8 (CH_2), 52.1 (CH), 33.8 (CH_2), 26.5 (CH_2), 23.0 (CH_3), 20.4 (CH_2). **IR** (ATR): 3366, 3056, 2929, 1752, 1608, 1589, 1471, 1438, 1138, 742 cm^{-1}. **MS** (ESI) *m/z* (relative intensity): 993 (16) [2M+Na]$^+$, 971 (20) [2M+H]$^+$, 508 (33) [M+Na]$^+$, 486 (100) [M+H]$^+$. **HR-MS** (ESI) *m/z* calcd for $C_{28}H_{28}N_3O_5$ [M+H]$^+$: 486.2023; found: 486.2024.

(*S*)-2-acetamido-3-{2-[3-(3-methoxyphenoxy)-3-oxopropyl]-1-[pyridin-2-yl]-1*H*-indol-3-yl}propanoic acid (**3u**):

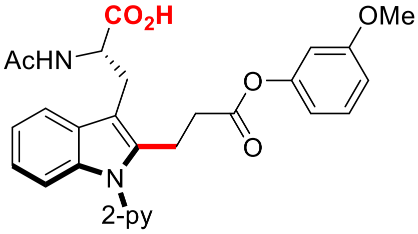

The general procedure A was followed using methyl N^{α}-acetyl-1-(pyridin-2-yl)-*L*-tryptophan (48.5 mg, 150 µmol) and 3-methoxyphenyl acrylate (41 mg, 0.23 mmol). Purification by column chromatography (CH_2Cl_2/MeOH 30:1→20:1) yielded **3u** (42 mg, 84 µmol, 56%) as a white solid. **m.p.** 116 °C. **^{1}H NMR** (400 MHz, $CDCl_3$): δ 8.73 (d, J = 4.9 Hz, 1H), 7.99 (dd, J = 7.7, 7.7 Hz, 1H), 7.59 (d, J = 7.4 Hz, 1H), 7.51 (d, J = 7.7 Hz, 1H), 7.45 (dd, J = 7.4, 4.9 Hz, 1H), 7.22–7.11 (m, 2H), 7.11–6.98 (m, 2H), 6.93–6.83 (m, 1H), 6.70 (dd, J = 8.1, 2.2 Hz, 1H), 6.43 (dd, J = 8.1, 2.2 Hz, 1H), 6.31 (d, J = 2.2 Hz, 1H), 5.08–4.99 (m, 1H), 3.64 (s, 3H), 3.39–3.16 (m, 3H), 3.16 – 2.96 (m, 1H), 2.45-2.28 (m, 2H), 1.81 (s, 3H). **^{13}C NMR** (101 MHz, $CDCl_3$): δ 174.0 (C_q), 171.3 (C_q), 170.7 (C_q), 160.4 (C_q), 151.2 (C_q), 150.6 (C_q), 149.2 (CH) , 139.7 (CH), 136.9 (C_q), 136.4 (C_q), 129.7 (CH), 128.6 (C_q), 123.1 (CH), 122.6 (CH), 122.1 (CH), 120.8 (CH), 119.0 (CH), 113.4 (CH), 112.0 (CH), 110.1 (C_q), 109.6 (CH), 107.1 (CH), 55.3 (CH_3), 52.2 (CH), 33.5 (CH_2), 26.5 (CH_2), 22.7 (CH_3), 20.2 (CH_2). **IR** (ATR): 3341, 3053, 2932, 1731,

1586, 1471, 1437, 1163, 742 cm^{-1}. **MS** (ESI) *m/z* (relative intensity): 1003 (26) [2M+H]$^+$, 524 (34) [M+Na]$^+$, 502 (100) [M+H]$^+$. **HR-MS** (ESI) *m/z* calcd for $C_{28}H_{28}N_3O_6$ [M+H]$^+$: 502.1973; found: 502.1974.

(*S*)-methyl 2-acetamido-3-[2-(1-cyclohexyl-2,5-dioxopyrrolidin-3-yl)-1-(pyridin-2-yl)-1H-indol-3-yl]propanoate (**3v**)

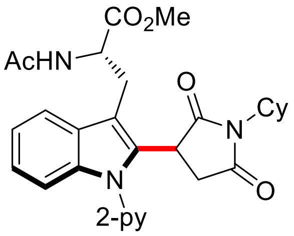

The general procedure A was followed using methyl acetyl-1-(2-pyridyl)-L-tryptophanate (**1a**, 25.0 mg, 74 µmol) and 1-cyclohexyl-1*H*-pyrrole-2,5-dione (**185**, 20 mg, 0.11 mmol, 1.5 equiv) in HOAc (74 µL) at 100 °C. Purification by column chromatography (EtOAc) yielded **3v** (29 mg, 56 µmol, 76%, dr = 1:1) as a colorless oil. Due to formation of two diastereomers, the NMR spectra show a double set of signals. **^{1}H NMR** (600 MHz, $CDCl_3$): δ 8.45 (ddd, *J* = 5.0, 2.0, 0.9 Hz, 0.5H), 8.42 (ddd, *J* = 5.0, 2.0, 0.9 Hz, 0.5H), 7.91 (ddd, *J* = 7.2, 7.2, 2.0 Hz, 0.5H), 7.89 (ddd, *J* = 7.2, 7.2, 2.0 Hz, 0.5H), 7.69–7.65 (m, 0.5H), 7.56–7.51 (m, 1.5H), 7.41–7.38 (m, 0.5H), 7.34–7.31 (m, 0.5H), 7.29–7.25 (m, 1H), 7.23–7.20 (m, 1H), 7.21–7.13 (m, 1.5H), 6.87 (d, *J* = 8.4 Hz, 1H), 5.02 (ddd, *J* = 8.5, 6.0, 4.4 Hz, 0.5H), 4.76 (ddd, *J* = 9.8, 4.8, 4.8 Hz, 0.5H), 4.46–4.38 (m, 1H), 3.99 (dddd, *J* = 12.3, 12.3, 3.9, 3.9 Hz, 0.5H), 3.90 (dddd, *J* = 12.3, 12.3, 3.9, 3.9 Hz, 0.5H), 3.79 (s, 1.5H), 3.62 (s, 1.5H), 3.57–3.50 (m, 0.5H), 3.46 (dd, *J* = 14.8, 4.8 Hz, 0.5H), 3.20 (dd, *J* = 15.0, 6.0 Hz, 0.5H), 3.11 (dd, *J* = 14.8, 9.9 Hz, 0.5H), 3.03 (dd, *J* = 18.0, 6.7 Hz, 0.5H), 2.95 (dd, *J* = 18.0, 9.7 Hz, 0.5H), 2.83 (dd, *J* = 18.2, 10.0 Hz, 0.5H), 2.54 (dd, *J* = 18.2, 6.3 Hz, 0.5H), 2.20–2.09 (m, 1H), 2.09–1.95 (m, 1H), 2.01 (s, 1.5H), 1.98 (s, 1.5H), 1.89–1.77 (m, 2H), 1.68–1.60 (m, 2H), 1.52–1.42 (m, 1H), 1.39–1.23 (m, 2H), 1.23–1.14 (m, 1H). **^{13}C NMR** (126 MHz, $CDCl_3$): δ 177.9 (C_q), 177.9 (C_q), 175.7 (C_q), 175.7 (C_q), 173.0 (C_q), 172.1 (C_q), 171.1 (C_q), 170.6 (C_q), 151.0 (C_q), 150.9 (C_q), 149.5 (CH), 149.4 (CH), 139.0 (CH), 138.9 (CH), 136.6 (C_q), 136.5 (C_q), 131.6 (C_q), 130.2 (C_q), 128.0 (C_q), 127.6 (C_q), 123.9 (CH), 123.7 (CH), 122.3 (CH), 122.3 (CH), 121.5 (CH), 121.2 (CH), 120.6 (CH), 119.9 (CH), 119.1 (CH), 119.1 (CH), 114.4 (C_q), 114.4 (C_q) 110.4 (CH), 110.3 (CH), 53.0 (CH), 52.5 (CH_3), 52.5 (CH_3), 52.2 (CH), 52.1 (CH), 52.0 (CH), 38.3 (CH), 37.8 (CH), 35.8 (CH_2), 34.3 (CH_2), 28.9 (CH_2), 28.7 (CH_2), 28.6 (CH_2), 28.6 (CH_2), 27.0 (CH_2), 26.9 (CH_2), 25.9 (CH_2), 25.8 (CH_2), 25.8 (CH_2), 25.8 (CH_2), 25.0 (CH_2), 25.0 (CH_2), 23.0 (CH_3), 22.6 (CH_3). **IR** (ATR): 3282, 2935, 2856, 1740, 1693, 1583, 1472, 1436, 1186, 1141, 735 cm^{-1}. **MS** (ESI) *m/z* (relative intensity) 1055 (64) [2M+Na]$^+$, 539 (100) [M+Na]$^+$, 517 (7) [M+H]$^+$. **HR-MS** (ESI) *m/z* calcd for $C_{29}H_{33}N_4O_5^+$ 517.2445 [M+H]$^+$, found 517.2440.

Methyl acetyl-1-(2-pyridyl)-2-(*S*-cyclohexyl propanethioate-3-yl)-L-tryptophanate (3w):

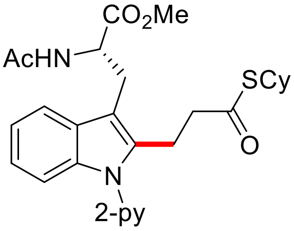

The general procedure A was followed using methyl acetyl-1-(2-pyridyl)-L-tryptophanate (**1a**, 50.6 mg, 150 µmol) and *S*-cyclohexyl prop-2-enethioate (77 mg, 0.45 mmol). Purification by column chromatography (EtOAc) yielded **3w** (57 mg, 0.11 mmol, 75%) as a white solid. **m. p.** 185 (decomp.). **^{1}H NMR** (600 MHz, $CDCl_3$): δ 8.66 (ddd, *J* = 4.9, 2.0, 0.8 Hz, 1H), 7.94 (ddd, *J* = 7.7, 7.7, 2.0 Hz, 1H), 7.57–7.52 (m, 1H), 7.49 (ddd, *J* = 8.0, 0.9, 0.9 Hz, 1H), 7.36 (ddd, *J* = 7.5, 4.8, 1.0 Hz, 1H), 7.31–7.27 (m, 1H), 7.17–7.12 (m, 2H), 6.24 (d, *J* = 7.8 Hz, 1H), 4.97 (ddd, *J* = 7.8, 5.9, 5.9 Hz, 1H), 3.71 (s, 3H), 3.45–3.37 (m, 1H), 3.35 (dd, *J* = 14.8, 5.9 Hz, 1H), 3.30 (dd, *J* = 14.8, 5.9 Hz, 1H), 3.27–3.15 (m, 2H), 2.51 (ddd, *J* = 8.6, 6.8, 2.7 Hz, 2H), 1.97 (s, 3H), 1.84–1.78 (m, 2H), 1.67–1.61 (m, 2H), 1.58–1.51 (m, 1H), 1.41–1.18 (m, 5H). **^{13}C NMR** (126 MHz, $CDCl_3$): δ 197.8 (C_q), 172.4 (C_q), 169.7 (C_q), 150.9 (C_q), 149.5 (CH), 138.7 (CH), 136.7 (C_q), 136.5 (C_q), 128.6 (C_q), 122.5 (CH), 122.3 (CH), 120.9 (CH), 120.8 (CH), 118.5 (CH), 109.9 (CH), 109.5 (C_q), 52.8 (CH), 52.5 (CH_3), 43.3 (CH_2), 42.4 (CH), 33.0 (CH_2), 27.1 (CH_2), 25.9 (CH_2), 25.6 (CH_2), 23.2 (CH_3), 21.0 (CH_2). **IR** (ATR): 2927, 2852, 1742, 1675, 1470, 1459, 1436, 1369, 740 cm^{-1}. **MS** (ESI) *m/z* (relative intensity) 1037 (36) $[2M+Na]^+$, 530 (100) $[M+Na]^+$, 508 (11) $[M+H]^+$. **HR-MS** (ESI) *m/z* calcd for $C_{28}H_{34}N_3O_4S^+$ 508.2265 $[M+H]^+$, found 508.2252.

Methyl acetyl-1-(2-pyridyl)-2-(cholesteryl propionate-3-yl)-L-tryptophanate (3x):

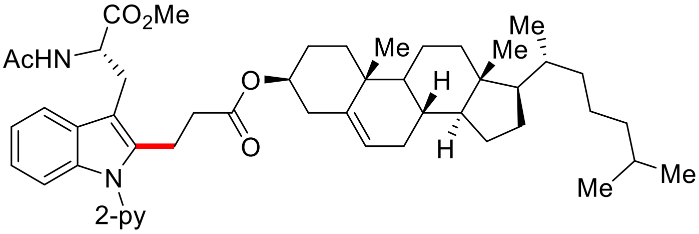

The general procedure A was followed using methyl acetyl-1-(2-pyridyl)-L-tryptophanate (**1a**, 50.6 mg, 150 µmol), cholesteryl acrylate (98 mg, 0.23 mmol) and a mixture of 150 µL HOAc and 150 µL toluene as the solvent. The reaction mixture was heated to 100 °C for 15 h. Purification by column chromatography (EtOAc) yielded **3x** (100 mg, 0.13 mmol, 86%) as a white solid. **m. p.** 92 °C. **^{1}H NMR** (600 MHz, $CDCl_3$): δ 8.64 (dd, *J* = 4.8, 2.0 Hz, 1H), 7.92 (ddd, *J* = 7.7, 7.7, 2.0 Hz, 1H), 7.55–7.49 (m, 1H), 7.46 (d, *J* = 7.9 Hz, 1H), 7.34 (ddd, *J* = 7.5, 4.9, 1.1 Hz, 1H), 7.27–7.24 (m, 1H), 7.15–7.10 (m, 2H), 6.34 (d, *J* = 7.8 Hz, 1H), 5.31–5.26 (m, 1H), 4.95 (ddd, *J* = 7.8, 6.0, 6.0 Hz, 1H), 4.46 (dddd, *J* = 10.9, 10.9, 6.5, 4.4 Hz, 1H), 3.70 (s, 3H), 3.36 (dd, *J* = 14.8, 6.0 Hz, 1H), 3.30 (dd, *J* = 14.8, 6.0 Hz, 1H), 3.23–3.12 (m, 2H), 2.29–2.17 (m, 2H), 2.17–2.12 (m, 2H), 2.00–1.95 (m, 1H), 1.95–1.89 (m, 1H), 1.93 (s, 3H), 1.84–1.76 (m, 2H), 1.75–1.69 (m, 1H), 1.58–0.95 (m, 21H), 0.94 (s, 3H), 0.89 (d, *J* = 6.6 Hz, 3H), 0.85 (d, *J* = 6.6 Hz, 3H), 0.84 (d, *J* = 6.6 Hz, 3H), 0.64 (s, 3H). **^{13}C NMR** (126 MHz,

CDCl$_3$): δ 172.4 (C$_q$), 171.7 (C$_q$), 170.0 (C$_q$), 151.1 (C$_q$), 149.7 (CH), 139.4 (C$_q$), 138.6 (CH), 137.0 (C$_q$), 136.7 (C$_q$), 128.6 (C$_q$), 122.6 (CH), 122.4 (CH), 122.4 (CH), 121.1 (CH), 120.7 (CH), 118.4 (CH), 109.9 (CH), 109.3 (C$_q$), 74.2 (CH), 56.7 (CH), 56.2 (CH), 52.9 (CH), 52.5 (CH$_3$), 50.0 (CH), 42.4 (C$_q$), 39.8 (CH$_2$), 39.6 (CH$_2$), 38.0 (CH$_2$), 37.0 (CH$_2$), 36.6 (C$_q$), 36.2 (CH$_2$), 35.8 (CH), 34.0 (CH$_2$), 31.9 (CH$_2$), 31.9 (CH), 28.3 (CH$_2$), 28.1 (CH), 27.7 (CH$_2$), 27.1 (CH$_2$), 24.3 (CH$_2$), 23.9 (CH$_2$), 23.2 (CH$_3$), 22.9 (CH$_3$), 22.6 (CH$_3$), 21.1 (CH$_2$), 20.4 (CH$_2$), 19.3 (CH$_3$), 18.8 (CH$_3$), 11.9 (CH$_3$). **IR** (ATR): 3294, 2952, 1733, 1647, 1557, 1471, 1458, 1436, 1369, 1202, 1169, 1141, 741 cm^{-1}. **MS** (ESI) *m/z* (relative intensity) 800 (100) [M+Na]$^+$, 778 (26) [M+H]$^+$. **HR-MS** (ESI) *m/z* calcd for $C_{49}H_{68}N_3O_5^+$ 778.5153 [M+H]$^+$, found 778.5133.

Methyl acetyl-1-(2-pyridyl)-2-(4-allyl-2-methoxyphenyl propionate-3-yl)-L-tryptophanate (3y):

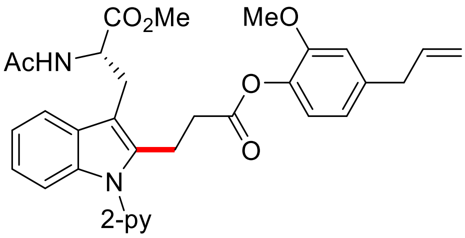

The general procedure A was followed using methyl acetyl-1-(2-pyridyl)-L-tryptophanate (**1a**, 50.6 mg, 150 µmol) and 4-allyl-2-methoxyphenyl acrylate (50 mg, 0.23 mmol) in toluene/HOAc (1:1, 150 µL). Purification by column chromatography (hexanes/EtOAc 1:1→1:2) yielded **3y** (73 mg, 132 µmol, 88%) as a pale yellow solid. **m. p.** 77 °C. **^{1}H NMR** (600 MHz, CDCl$_3$): δ 8.66 (ddd, *J* = 5.0, 1.9, 0.9 Hz, 1H), 7.93 (ddd, *J* = 7.8, 7.8, 2.0 Hz, 1H), 7.62–7.56 (m, 1H), 7.52 (dd, *J* = 8.0, 0.9 Hz, 1H), 7.35 (dd, *J* = 7.5, 4.9 Hz, 1H), 7.33–7.29 (m, 1H), 7.20–7.14 (m, 2H), 6.78 (dd, *J* = 8.0, 0.7 Hz, 1H), 6.73–6.63 (m, 2H), 6.32 (d, *J* = 7.6 Hz, 1H), 5.92 (ddtd, *J* = 16.9, 10.2, 6.7, 0.7 Hz, 1H), 5.08 (ddd, *J* = 9.2, 1.2, 0.8 Hz, 1H), 5.06 (q, *J* = 1.2 Hz, 1H), 4.99 (ddd, *J* = 7.6, 6.1, 6.1 Hz, 1H) 3.72 (s, 3H), 3.63 (s, 3H), 3.46–3.26 (m, 6H), 2.60 (ddd, *J* = 16.7, 8.6, 6.0 Hz, 1H), 2.54 (ddd, *J* = 16.3, 8.7, 7.3 Hz, 1H), 1.85 (s, 3H). **^{13}C NMR** (126 MHz, CDCl$_3$): δ 172.5 (C$_q$), 170.7 (C$_q$), 169.9 (C$_q$), 151.1 (C$_q$), 150.5 (C$_q$), 149.7 (CH), 138.9 (C$_q$), 138.5 (CH), 137.6 (C$_q$), 136.8 (CH), 136.7 (C$_q$), 136.5 (C$_q$), 128.5 (C$_q$), 122.5 (CH), 122.2 (CH), 122.2 (CH), 120.9 (CH), 120.7 (CH), 120.5 (CH), 118.6 (CH), 116.0 (CH$_2$), 112.6 (CH), 109.9 (CH), 109.6 (C$_q$), 55.6 (CH$_3$), 52.8 (CH), 52.4 (CH$_3$), 40.0 (CH$_2$), 33.3 (CH$_2$), 27.1 (CH$_2$), 23.0 (CH$_3$), 20.4 (CH$_2$). **IR** (ATR): 2951, 1745, 1656, 1507, 1437, 1137, 1123, 742 cm^{-1}. **MS** (ESI) *m/z* (relative intensity) 578 (62) [M+Na]$^+$, 556 (100) [M+H]$^+$. **HR-MS** (ESI) *m/z* calcd for $C_{32}H_{34}N_3O_6^+$ 556.2442 [M+H]$^+$, found 556.2438.

Methyl acetyl-1-(2-pyridyl)-2-((–)-menthyl propionate-3-yl)-L-tryptophanate (3z):

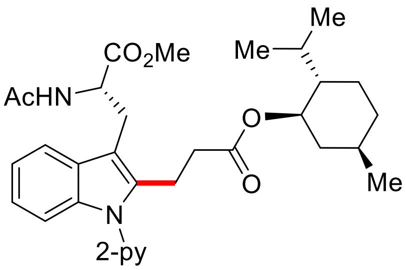

The general procedure A was followed using methyl acetyl-1-(2-pyridyl)-L-tryptophanate (**1a**, 50.6 mg, 150 µmol) and (–)-menthyl acrylate (47 mg, 0.23 mmol). Purification by column chromatography (EtOAc) yielded **3z** (51 mg, 93 µmol, 62%) as a white solid. **m. p.** 84 °C. **^{1}H NMR** (400 MHz, $CDCl_3$): δ 8.63 (ddd, *J* = 4.9, 2.0, 0.8 Hz, 1H), 7.93 (ddd, *J* = 8.0, 7.4, 2.0 Hz, 1H), 7.57–7.52 (m, 1H), 7.46 (ddd, *J* = 8.0, 0.9, 0.9 Hz, 1H), 7.35 (ddd, *J* = 7.5, 4.9, 1.0 Hz, 1H), 7.30–7.27 (m, 1H), 7.17–7.11 (m, 2H), 6.30 (d, *J* = 7.7 Hz, 1H), 4.97 (ddd, *J* = 7.8, 5.9, 5.9 Hz, 1H), 4.56 (ddd, *J* = 10.9, 10.9, 4.4 Hz, 1H), 3.72 (s, 3H), 3.37 (dd, *J* = 14.8, 6.0 Hz, 1H), 3.32 (dd, *J* = 14.8, 6.0 Hz, 1H), 3.28–3.15 (m, 2H), 2.24 (dd, *J* = 7.6, 7.6 Hz, 2H), 1.96 (s, 3H), 1.79 (dddd, *J* = 12.0, 3.9, 3.9, 1.9 Hz, 1H), 1.67–1.52 (m, 3H), 1.39 (ddddd, *J* = 15.2, 8.5, 3.2, 3.2, 3.2 Hz, 1H), 1.29–1.17 (m, 1H), 1.04–0.92 (m, 1H), 0.83 (d, *J* = 6.5 Hz, 3H), 0.82–0.79 (m, 1H), 0.79–0.77 (m, 1H), 0.77 (d, *J* = 7.0 Hz, 3H), 0.62 (d, *J* = 6.9 Hz, 3H). **^{13}C NMR** (126 MHz, $CDCl_3$): δ 172.4 (C_q), 171.9 (C_q), 151.1 (C_q), 149.5 (CH), 138.4 (CH), 136.8 (C_q), 136.7 (C_q), 130.0 (C_q), 128.6 (C_q), 122.4 (CH), 122.1 (CH), 120.8 (CH), 120.7 (CH), 118.5 (CH), 109.8 (CH), 109.4 (C_q), 74.3 (CH), 52.8 (CH), 52.4 (CH_3), 47.0 (CH), 40.9 (CH_2), 34.2 (CH_2), 33.9 (CH_2), 31.4 (CH), 27.1 (CH_2), 26.4 (CH), 23.7 (CH_2), 23.1 (CH_3), 22.1 (CH_3), 20.8 (CH_3), 20.5 (CH_2), 16.5 (CH_3). **IR** (ATR): 2953, 2927, 2868, 1729, 1656, 1471, 1459, 1436, 1369, 1176, 1148, 739 cm^{-1}. **MS** (ESI) *m/z* (relative intensity) 1117 (49) $[2M+Na]^+$, 570 (69) $[M+Na]^+$, 548 (100) $[M+H]^+$, 410 (11) $[M–menthol+H]^+$. **HR-MS** (ESI) *m/z* calcd for $C_{32}H_{42}N_3O_5^+$ 548.3119 $[M+H]^+$, found 548.3109.

Methyl acetyl-1-(2-pyridyl)-2-{2-[2-(4-isobutylphenyl)propanamido]ethyl propionate-3-yl}-L-tryptophanate (3aa):

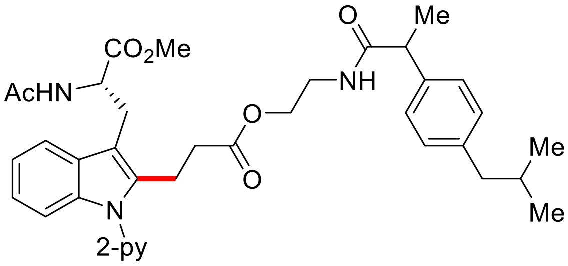

The general procedure A was followed using methyl acetyl-1-(2-pyridyl)-L-tryptophanate (**1a**, 50.6 mg, 150 µmol) and 2-[2-(4-isobutylphenyl)propanamido]ethyl acrylate (69 mg, 0.23 mmol). Purification by column chromatography (hexanes/EtOAc 1:1→1:2) yielded **3aa** (80 mg, 125 µmol, 83%) as a white solid. **m. p.** 123–126 °C. **^{1}H NMR** (600 MHz, $CDCl_3$, mixture of diasteromers 1.0/1.0): δ 8.60 (dd, *J* = 4.7, 2.9 Hz, 1H), 7.94–7.85 (m, 1H), 7.49 (ddd, *J* = 5.8, 1.8, 1.8 Hz, 1H), 7.41 (d, *J* = 7.7 Hz, 1H), 7.33 (dd, *J* = 7.3, 4.8 Hz, 1H), 7.17–7.09 (m, 4H), 7.04 (d, *J* = 7.6 Hz, 2H), 6.41–6.35 (m, 2H), 4.91–4.81

(m, 1H), 4.15–3.90 (m, 2H), 3.64 (s, 1.5H), 3.63 (s, 1.5H), 3.57–3.44 (m, 1H), 3.44–3.23 (m, 2H), 3.22–3.10 (m, 2H), 2.38 (d, J = 7.2 Hz, 3H), 2.32–2.23 (m, 2H), 1.94 (s, 1.5H), 1.91 (s, 1.5H), 1.83–1.74 (m, 1H), 1.43 (d, J = 7.1 Hz, 1.5H), 1.42 (d, J = 6.9 Hz, 1.5H), 0.84 (d, J = 6.6 Hz, 6H). **^{13}C NMR** (126 MHz, $CDCl_3$): δ 174.5 (C_q), 172.4 (C_q), 172.4 (C_q), 171.9 (C_q), 169.8 (C_q), 151.0 (C_q), 149.6 (CH), 140.3 (C_q), 138.6 (C_q), 138.5 (C_q), 138.5 (CH), 136.6 (C_q), 136.6 (C_q), 136.5 (C_q), 129.3 (CH), 128.4 (C_q), 128.4 (C_q), 127.1 (CH), 122.4 (CH), 122.3 (CH), 120.8 (CH), 120.7 (CH), 118.2 (CH), 118.2 (CH), 110.0 (CH), 109.3 (C_q), 63.5 (CH_2), 53.1 (CH), 53.1 (CH), 52.4 (CH_3), 46.3 (CH), 46.3 (CH), 45.0 (CH_2), 38.6 (CH_2), 34.0 (CH_2), 33.9 (CH_2), 30.1 (CH), 27.2 (CH_2), 27.2 (CH_2), 23.2 (CH_3), 23.2 (CH_3), 22.4, 20.5 (CH_2), 18.7 (CH_3), 18.6 (CH_3). **IR** (ATR): 3276, 3074, 2962, 1742, 1647, 1542, 1472, 1459, 1148, 743 cm^{-1}. **MS** (ESI) *m/z* (relative intensity) 1304 (22) $[2M+Na]^+$ 663 (63) $[M+Na]^+$, 641 (100) $[M+H]^+$. **HR-MS** (ESI) *m/z* calcd for $C_{37}H_{45}N_4O_6^+$ 641.3334 $[M+H]^+$, found 641.1331.

Methyl acetyl-1-(2-pyridyl)-2-(1-pyrenyl propionate-3-yl)-L-tryptophanate (3bb):

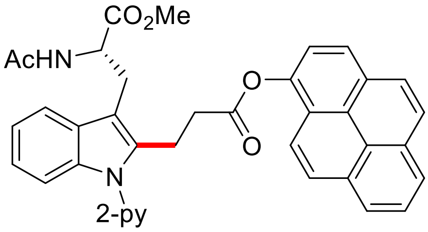

The general procedure A was followed using methyl acetyl-1-(2-pyridyl)-L-tryptophanate (**1a**, 50.6 mg, 150 µmol) and pyren-1-yl acrylate (62 mg, 0.23 mmol). Purification by column chromatography (hexanes/EtOAc 1:1→1:2) yielded **3bb** (62 mg, 102 µmol, 68%) as a white solid. **m. p.** 205 °C. **^{1}H NMR** (300 MHz, $CDCl_3$): δ 8.76 (dd, J = 4.9, 1.2 Hz, 1H), 8.26–8.08 (m, 3H), 8.07–7.88 (m, 4H), 7.78 (d, J = 9.2 Hz, 1H), 7.70–7.57 (m, 4H), 7.48–7.35 (m, 2H), 7.33–7.24 (m, 2H), 6.29 (d, J = 7.7 Hz, 1H), 5.04 (ddd, J = 7.7, 6.1, 6.1 Hz, 1H), 3.72 (s, 3H), 3.64–3.33 (m, 4H), 2.98–2.84 (m, 2H), 1.81 (s, 3H). **^{13}C NMR** (126 MHz, $CDCl_3$): δ 172.6 (C_q), 171.6 (C_q), 170.0 (C_q), 151.2 (C_q), 149.7 (CH), 144.0 (C_q), 138.9 (CH), 136.9 (C_q), 136.3 (C_q), 131.0 (C_q), 130.8 (C_q), 129.3 (C_q), 128.8 (C_q), 128.1 (CH), 127.1 (CH), 127.0 (CH), 126.3 (CH), 125.5 (CH), 125.4 (C_q), 125.3 (CH), 124.9 (CH), 124.4 (C_q), 122.9 (C_q), 122.8 (CH), 122.5 (CH), 121.1 (CH), 121.0 (CH), 119.9 (CH), 119.5 (CH), 118.8 (CH), 110.2 (C_q), 110.1 (CH), 52.8 (CH), 52.4 (CH_3), 33.8 (CH_2), 27.1 (CH_2), 22.9 (CH_3), 20.6 (CH_2). **IR** (ATR): 3276, 3074, 2962, 2930, 1742, 1647, 1542, 1472, 1459, 1148, 743 cm^{-1}. **MS** (ESI) *m/z* (relative intensity) 1241 (100) $[2M+Na]^+$, 632 (59) $[M+Na]^+$, 610 (33) $[M+H]^+$. **HR-MS** (ESI) *m/z* calcd for $C_{38}H_{32}N_3O_5^+$ 610.2336 $[M+H]^+$, found 610.2336.

Methyl acetyl-1-(2-pyridyl)-2-(butane-3-one-1-yl)-L-tryptophanate (4a):

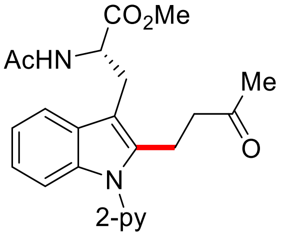

The general procedure A was followed using methyl acetyl-1-(2-pyridyl)-L-tryptophanate (**1a**, 50.6 mg, 150 µmol) and but-3-en-2-one (32 mg, 0.45 mmol). Purification by column chromatography (EtOAc) yielded **4a** (61 mg, 0.15 mmol, 99%) as a colorless oil. **^{1}H NMR** (300 MHz, $CDCl_3$): δ 8.60 (ddd, *J* = 4.9, 2.0, 0.8 Hz, 1H), 7.88 (ddd, *J* = 8.0, 7.5, 2.0 Hz, 1H), 7.54–7.48 (m, 1H), 7.41 (ddd, *J* = 8.0, 0.9, 0.9 Hz, 1H), 7.31 (ddd, *J* = 7.5, 4.9, 1.0 Hz, 1H), 7.27–7.20 (m, 1H), 7.13–7.07 (m, 2H), 6.33 (d, *J* = 7.8 Hz, 1H), 4.91 (ddd, *J* = 7.8, 6.1, 6.1 Hz, 1H), 3.66 (s, 3H), 3.38–3.20 (m, 2H), 3.07 (ddd, *J* = 7.8, 7.8, 2.0 Hz, 2H), 2.54–2.43 (m, 2H), 1.97 (s, 3H), 1.91 (s, 3H). **^{13}C NMR** (76 MHz, $CDCl_3$): δ 207.3 (C_q), 172.6 (C_q), 169.9 (C_q), 151.2 (C_q), 149.7 (CH), 138.5 (CH), 137.3 (C_q), 136.7 (C_q), 128.6 (C_q), 122.3 (CH), 122.3 (CH), 120.9 (CH), 120.7 (CH), 118.3 (CH), 109.9 (CH), 109.0 (C_q), 52.8 (CH), 52.3 (CH_3), 42.9 (CH_2), 29.8 (CH_3), 27.0 (CH_2), 23.0 (CH_3), 18.9 (CH_2). **IR** (ATR): 3277, 2952, 2926, 1740, 1713, 1655, 1585, 1470, 1459, 1436, 1367, 1218, 1165, 741 cm^{-1}. **MS** (ESI) *m/z* (relative intensity) 837 (38) $[2M+Na]^+$, 430 (90) $[M+Na]^+$, 408 (100) $[M+H]^+$. **HR-MS** (ESI) *m/z* calcd for $C_{23}H_{26}N_3O_4^+$ 408.1918 $[M+H]^+$, found 408.1916.

Methyl acetyl-1-(2-pyridyl)-2-(octane-3-one-1-yl)-L-tryptophanate (4b):

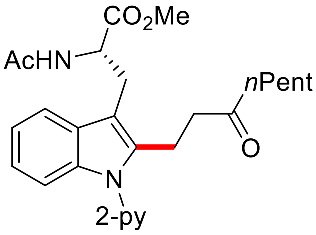

The general procedure A was followed using methyl acetyl-1-(2-pyridyl)-L-tryptophanate (**1a**, 50.6 mg, 150 µmol) and oct-1-en-3-one (37 mg, 0.30 mmol). Purification by column chromatography (EtOAc) yielded **4b** (67 mg, 0.14 mmol, 97%) as a colorless oil. **^{1}H NMR** (500 MHz, $CDCl_3$): δ 8.63 (ddd, *J* = 4.9, 2.0, 0.8 Hz, 1H), 7.92 (ddd, *J* = 8.0, 7.5, 2.0 Hz, 1H), 7.56–7.51 (m, 1H), 7.45 (ddd, *J* = 7.9, 0.9, 0.9 Hz, 1H), 7.35 (ddd, *J* = 7.5, 4.9, 1.0 Hz, 1H), 7.29–7.26 (m, 1H), 7.18–7.11 (m, 2H), 6.32 (d, *J* = 7.8 Hz, 1H), 4.95 (ddd, *J* = 7.7, 6.0, 6.0 Hz, 1H), 3.71 (s, 3H), 3.36 (dd, *J* = 14.8, 6.0 Hz, 1H), 3.30 (dd, *J* = 14.8, 6.0 Hz, 1H), 3.16–3.03 (m, 2H), 2.46 (dd, *J* = 7.6, 7.6 Hz, 2H), 2.21 (ddd, *J* = 7.2, 7.2, 1.2 Hz, 2H), 1.95 (s, 3H), 1.48–1.40 (m, 2H), 1.28–1.19 (m, 2H), 1.19–1.10 (m, 2H), 0.84 (dd, *J* = 7.2, 7.2 Hz, 3H). **^{13}C NMR** (126 MHz, $CDCl_3$): δ 209.9 (C_q), 172.6 (C_q), 169.9 (C_q), 151.2 (C_q), 149.6 (CH), 138.7 (CH), 137.5 (C_q), 136.7 (C_q), 128.7 (C_q), 122.4 (CH), 122.3 (CH), 121.0 (CH), 120.8 (CH), 118.4 (CH), 110.0 (CH), 109.1 (C_q), 52.8 (CH), 52.4 (CH_3), 42.7 (CH_2), 41.9 (CH_2), 31.2 (CH_2), 27.0 (CH_2), 23.4 (CH_2), 23.1 (CH_3), 22.4 (CH_2), 19.0 (CH_2), 13.9 (CH_3). **IR** (ATR): 3290, 2953, 2928, 1742, 1711, 1654,

1585, 1470, 1458, 1436, 1369, 1216, 1130, 741 cm^{-1}. **MS** (ESI) *m/z* (relative intensity) 949 (92) $[2M+Na]^+$, 486 (100) $[M+Na]^+$, 464 (26) $[M+H]^+$. **HR-MS** (ESI) *m/z* calcd for $C_{27}H_{34}N_3O_4^+$ 464.2544 $[M+H]^+$, found 464.2541.

Methyl acetyl-1-(2-pyridyl)-2-(2-benzoylethane-1-yl)-L-tryptophanate (4c):

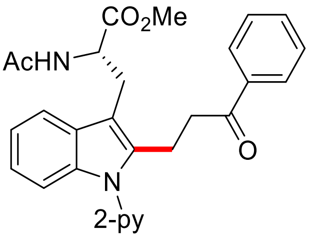

The general procedure A was followed using methyl acetyl-1-(2-pyridyl)-L-tryptophanate (**1a**, 50.6 mg, 150 µmol) and 1-phenylprop-2-en-1-one (59 mg, 0.45 mmol). Purification by column chromatography (EtOAc) yielded **4c** (48 mg, 0.10 mmol, 68%) as a white solid. **m. p.** 150 °C. **^{1}H NMR** (500 MHz, $CDCl_3$): δ 8.60 (ddd, *J* = 5.0, 2.0, 0.9 Hz, 1H), 7.90 (ddd, *J* = 7.7, 7.7, 2.0 Hz, 1H), 7.81–7.76 (m, 2H), 7.58–7.55 (m, 1H), 7.53 (dddd, *J* = 8.6, 7.1, 1.3, 1.3 Hz, 1H), 7.46 (ddd, *J* = 8.0, 1.0, 1.0 Hz, 1H), 7.40 (dd, *J* = 8.2, 7.3 Hz, 2H), 7.32 (ddd, *J* = 7.5, 4.9, 1.0 Hz, 1H), 7.30–7.27 (m, 1H), 7.18–7.14 (m, 2H), 6.31 (d, *J* = 7.8 Hz, 1H), 4.99 (ddd, *J* = 7.9, 5.9, 5.9 Hz, 1H), 3.71 (s, 3H), 3.40 (dd, *J* = 14.8, 6.1 Hz, 1H), 3.33 (dd, *J* = 14.8, 5.8 Hz, 1H), 3.30–3.20 (m, 2H), 3.16–3.02 (m, 2H), 1.95 (s, 3H). **^{13}C NMR** (126 MHz, $CDCl_3$): δ 198.8 (C_q), 172.7 (C_q), 169.9 (C_q), 151.3 (C_q), 149.8 (CH), 138.6 (CH), 137.6 (C_q), 136.8 (C_q), 136.4 (C_q), 133.2 (CH), 128.8 (C_q), 128.6 (CH), 127.9 (CH), 122.4 (CH), 122.4 (CH), 121.1 (CH), 120.8 (CH), 118.4 (CH), 110.0 (CH), 109.1 (C_q), 52.8 (CH), 52.4 (CH_3), 38.3 (CH_2), 27.1 (CH_2), 23.2 (CH_3), 19.5 (CH_2). **IR** (ATR): 3334, 3060, 1740, 1674, 1580, 1470, 1458, 1435, 1368, 1206, 740 cm^{-1}. **MS** (ESI) *m/z* (relative intensity) 961 (21) $[2M+Na]^+$, 939 (1) $[2M+H]^+$, 492 (100) $[M+Na]^+$, 470 (44) $[M+H]^+$. **HR-MS** (ESI) *m/z* calcd for $C_{28}H_{28}N_3O_4^+$ 470.2074 $[M+H]^+$, found 470.2074.

Methyl *tert*-butoxycarbonyl-1-(2-pyridyl)-2-(butane-3-one-1-yl)-L-tryptophanate (4d):

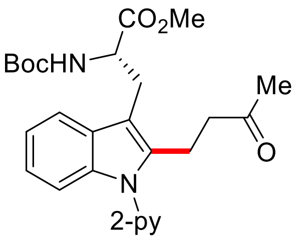

The general procedure A was followed using methyl *tert*-butoxycarbonyl-1-(2-pyridyl)-L-tryptophanate (59.3 mg, 150 µmol) and but-3-en-2-one (32 mg, 0.45 mmol) in H_2O/HOAc (9:1, 150 µL). Purification by column chromatography (hexanes/EtOAc 1:2) yielded **4d** (62 mg, 0.13 mmol, 89%) as a white solid. **m. p.** 87 °C. **^{1}H NMR** (300 MHz, $CDCl_3$): δ 8.63 (ddd, *J* = 4.9, 2.0, 0.8 Hz, 1H), 7.90 (ddd, *J* = 7.4, 2.0 Hz, 1H), 7.57–7.50 (m, 1H), 7.43 (ddd, *J* = 8.0, 1.0, 1.0 Hz, 1H), 7.33 (ddd, *J* = 7.5, 4.9, 1.0 Hz, 1H), 7.30–7.26 (m, 1H), 7.20–7.09 (m, 2H), 5.13 (d, *J* = 8.5 Hz, 1H), 4.64 (ddd, *J* = 6.7, 6.7, 6.7 Hz,

1H), 3.69 (s, 3H), 3.37–3.20 (m, 2H), 3.09 (dd, J = 7.6, 7.6 Hz, 2H), 2.62–2.52 (m, 2H), 2.03 (s, 3H), 1.40 (s, 9H). **^{13}C NMR** (126 MHz, $CDCl_3$): δ 207.0 (C_q), 172.7 (C_q), 155.0 (C_q), 151.3 (C_q), 149.6 (CH), 138.4 (CH), 137.4 (C_q), 136.7 (C_q), 128.7 (C_q), 122.2 (CH), 122.2 (CH), 121.0 (CH), 120.7 (CH), 118.5 (CH), 109.9 (CH), 109.1 (C_q), 79.8 (C_q), 54.1 (CH), 52.3 (CH_3), 43.4 (CH_2), 29.9 (CH_3), 28.4 (CH_3), 27.7 (CH_2), 19.2 (CH_2). **IR** (ATR): 3302, 2928, 1725, 1652, 1586, 1471, 1458, 1436, 1367, 1167, 1020, 741 cm^{-1}. **MS** (ESI) *m/z* (relative intensity) 953 (21) $[2M+Na]^+$, 488 (100) $[M+Na]^+$, 466 (24) $[M+H]^+$. **HR-MS** (ESI) *m/z* calcd for $C_{26}H_{32}N_3O_5^+$ 466.2336 $[M+H]^+$, found 466.2334.

Methyl acetyl-1-(2-pyridyl)-2-(*N*-*tert*-butoxycarbonyl-2-oxo-1-aminobutan-4-yl)-L-tryptophanate (4e):

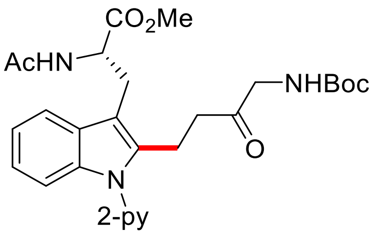

The general procedure A was followed using methyl acetyl-1-(2-pyridyl)-l-tryptophanate (**1a**, 50.6 mg, 150 µmol) and *tert*-butyl (2-oxobut-3-en-1-yl)carbamate (30 mg, 0.16 mmol) in H_2O/HOAc (9:1, 150 µL). Purification by column chromatography (EtOAc) yielded **4e** (62 mg, 0.13 mmol, 89%) as a white solid. **m. p.**: 96 °C. **^{1}H NMR** (500 MHz, $CDCl_3$): δ 8.65–8.60 (m, 1H), 7.92 (ddd, J = 8.0, 7.5, 2.0 Hz, 1H), 7.54–7.49 (m, 1H), 7.46 (ddd, J = 8.0, 1.0, 1.0 Hz, 1H), 7.35 (ddd, J = 7.5, 4.9, 1.0 Hz, 1H), 7.30–7.27 (m, 1H), 7.17–7.12 (m, 2H), 6.23 (d, J = 8.0 Hz, 1H), 5.19–5.14 (m, 1H), 4.93 (ddd, J = 8.1, 6.1, 6.1 Hz, 1H), 3.84 (d, J = 5.0 Hz, 2H), 3.68 (s, 3H), 3.34 (dd, J = 14.8, 6.5 Hz, 1H), 3.29 (dd, J = 14.8, 5.7 Hz, 1H), 3.20–2.08 (m, 2H), 2.54 (dd, J = 7.2, 7.2 Hz, 2H), 1.96 (s, 3H), 1.42 (s, 9H). **^{13}C NMR** (126 MHz, $CDCl_3$): δ 204.7 (C_q), 172.6 (C_q), 169.9 (C_q), 155.6 (C_q), 151.1 (C_q), 149.8 (CH), 138.7 (CH), 136.8 (C_q), 136.7 (C_q), 128.7 (C_q), 122.5 (CH), 122.4 (CH), 120.9 (CH), 120.9 (CH), 118.4 (CH), 110.1 (CH), 109.3 (C_q), 79.9 (C_q), 52.9 (CH), 52.5 (CH_3), 50.2 (CH_2), 39.4 (CH_2), 28.3 (CH_3), 27.2 (CH_2), 23.2 (CH_3), 19.0 (CH_2). **IR** (ATR): 3303, 2929, 1732, 1708, 1658, 1524, 1471, 1456, 1436, 1367, 1249, 1218, 1162, 742 cm^{-1}. **MS** (ESI) *m/z* (relative intensity) 1067 (17) $[2M+Na]^+$, 545 (100) $[M+Na]^+$, 523 (7) $[M+H]^+$. **HR-MS** (ESI) *m/z* calcd for $C_{28}H_{35}N_4O_6^+$ 523.2551 $[M+H]^+$, found 523.2545.

Methyl *N*-acetyl-1-(2-pyridyl)-2-(2-phenylphenyl propionate-3-yl)-L-tryptophyl-L-isoleucinate (6a):

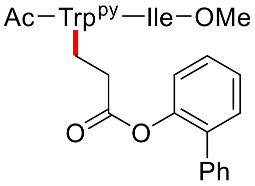

The general procedure A was followed using methyl *N*-acetyl-1-(2-pyridyl)-L-tryptophyl-L-isoleucinate (67.5 mg, 150 µmol) and 2-phenylphenyl acrylate (52 mg, 0.23 mmol). Purification by column chromatography (CH_2Cl_2/MeOH 40:1→30:1→20:1) yielded **6a** (77 mg, 114 µmol, 76%) as a white solid. **m. p.** 102 °C. **^{1}H NMR** (600 MHz, $CDCl_3$): δ 8.54 (ddd, *J* = 5.0, 2.0, 0.8 Hz, 1H), 7.87 (dd, *J* = 7.7, 7.7, 2.0 Hz, 1H), 7.70–7.66 (m, 1H), 7.46 (d, *J* = 8.0 Hz, 1H), 7.34 (dd, *J* = 7.3, 1.9 Hz, 1H), 7.32–7.28 (m, 6H), 7.29–7.26 (m, 2H), 7.26–7.24 (m, 1H), 7.18–7.11 (m, 2H), 7.04 (dd, *J* = 7.9, 1.5 Hz, 1H), 6.50 (d, *J* = 7.5 Hz, 1H), 6.29 (d, *J* = 8.1 Hz, 1H), 4.82 (ddd, *J* = 8.7, 7.5, 6.3 Hz, 1H), 4.34 (dd, *J* = 8.1, 4.9 Hz, 1H), 3.47 (s, 3H), 3.31 (dd, *J* = 14.5, 6.2 Hz, 1H), 3.16 (dd, *J* = 8.0, 6.7 Hz, 2H), 3.10 (dd, *J* = 14.5, 8.8 Hz, 1H), 2.57 (ddd, *J* = 17.1, 6.9, 6.9 Hz, 1H), 2.40 (ddd, *J* = 17.1, 7.8, 7.8 Hz, 1H), 1.93 (s, 3H), 1.71–1.66 (m, 1H), 1.27–1.19 (m, 1H), 1.02–0.93 (m, 1H), 0.80 (t, *J* = 7.4 Hz, 3H), 0.70 (d, *J* = 6.9 Hz, 3H). **^{13}C NMR** (126 MHz, $CDCl_3$): δ 171.2 (C_q), 170.9 (C_q), 170.9 (C_q), 169.7 (C_q), 151.2 (C_q), 149.5 (CH), 147.5 (C_q), 138.3 (CH), 137.4 (C_q), 136.7 (C_q), 136.4 (C_q), 134.6 (C_q), 130.6 (CH), 128.7 (CH), 128.3 (CH), 128.3 (C_q), 128.1 (CH), 127.3 (CH), 126.2 (CH), 122.8 (CH), 122.3 (CH), 122.1 (CH), 120.9 (CH), 120.8 (CH), 118.7 (CH), 110.1 (C_q), 109.9 (CH), 56.6 (CH), 53.6 (CH), 51.8 (CH_3), 38.1 (CH), 33.5 (CH_2), 28.1 (CH_2), 25.1 (CH_2), 23.2 (CH_3), 20.1 (CH_2), 15.2 (CH_3), 11.6 (CH_3). **IR** (ATR): 2961, 2923, 1747, 1646, 1472, 1436, 743 cm^{-1}. **MS** (ESI) *m/z* (relative intensity) 697 (100) $[M+Na]^+$, 675 (36) $[M+H]^+$. **HR-MS** (ESI) *m/z* calcd for $C_{40}H_{43}N_4O_6^+$ 675.3177 $[M+H]^+$, found 675.3165.

Methyl *N*-acetyl-1-(2-pyridyl)-2-(2-phenylphenyl propionate-3-yl)-L-tryptophyl-L-alaninyl-L-phenylalaninate (6b):

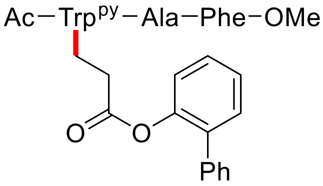

The general procedure B was followed using methyl *N*-acetyl-1-(2-pyridyl)-L-tryptophyl-L-alaninyl-L-phenylalaninate (84.6 mg, 150 µmol) and 2-phenylphenyl acrylate (52 mg, 0.23 mmol). Purification by column chromatography (CH_2Cl_2/MeOH 40:1→30:1→20:1) yielded **6b** (69 mg, 88 µmol, 59%) as a white solid. **m. p.** 204 °C. **^{1}H NMR** (300 MHz, $CDCl_3$): δ 8.52 (ddd, *J* = 4.9, 2.0, 0.8 Hz, 1H), 7.85 (ddd, *J* = 8.0, 7.5, 2.0 Hz, 1H), 7.71–7.60 (m, 1H), 7.43 (ddd, *J* = 8.0, 1.0, 1.0 Hz, 1H), 7.35–7.22 (m, 10H), 7.22–7.17 (m, 3H), 7.16–7.08 (m, 2H), 7.07–6.90 (m, 3H), 6.71–6.59 (m, 3H), 4.78 (ddd, *J* = 7.0, 7.0, 7.0 Hz, 1H), 4.64 (ddd, *J* = 7.7, 6.3, 6.3 Hz, 1H), 4.35 (dq, *J* = 7.0, 7.0 Hz, 1H), 3.62 (s, 3H), 3.41–

3.01 (m, 4H), 3.02–2.95 (m, 2H), 2.54–2.33 (m, 2H), 1.82 (s, 3H), 1.17 (d, *J* = 7.0 Hz, 3H). **^{13}C NMR** (126 MHz, $CDCl_3$): δ 171.5 (C_q), 171.3 (C_q), 171.1 (C_q), 171.0 (C_q), 170.2 (C_q), 151.1 (C_q), 149.5 (CH), 147.4 (C_q), 138.4 (CH), 137.3 (C_q), 136.8 (C_q), 136.3 (C_q), 135.8 (C_q), 134.6 (C_q), 130.7 (CH), 129.1 (CH), 128.6 (CH), 128.4 (CH), 128.4 (CH), 128.2 (C_q), 128.1 (CH), 127.3 (CH), 127.0 (CH), 126.3 (CH), 122.7 (CH), 122.5 (CH), 122.1 (CH), 121.0 (CH), 120.8 (CH), 118.8 (CH), 110.0 (C_q), 109.9 (CH), 53.6 (CH), 53.4 (CH), 52.2 (CH_3), 49.0 (CH), 37.8 (CH_2), 33.6 (CH_2), 27.6 (CH_2), 23.0 (CH_3), 20.1 (CH_2), 18.3 (CH_3). **IR** (ATR): 2923, 2853, 1748, 1683, 1637, 1507, 1473, 1188, 742 cm^{-1}. **MS** (ESI) *m/z* (relative intensity) 1582 (19) $[2M+Na]^+$, 802 (100) $[M+Na]^+$, 780 (66) $[M+H]^+$. **HR-MS** (ESI) *m/z* calcd for $C_{46}H_{46}N_5O_7^+$ 780.3392 $[M+H]^+$, found 780.3380.

Methyl *N*-acetyl-1-(2-pyridyl)-2-(3-methoxyphenyl propionate-3-yl)-L-tryptophyl-L-alaninyl-L-phenylalaninate (6c):

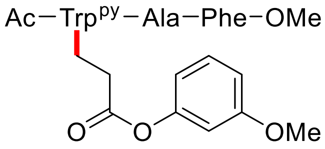

The general procedure B was followed using methyl *N*-acetyl-1-(2-pyridyl)-L-tryptophyl-L-alaninyl-L-phenylalaninate (84.6 mg, 150 µmol) and 3-methoxyphenyl acrylate (41 mg, 0.23 mmol). Purification by column chromatography (CH_2Cl_2/MeOH 40:1→30:1→20:1) yielded **6c** (58 mg, 80 µmol, 53%) as a white solid. **m. p.** 202 °C. **^{1}H NMR** (300 MHz, $CDCl_3$): δ 8.63 (ddd, *J* = 4.9, 2.0, 0.8 Hz, 1H), 7.90 (ddd, *J* = 8.0, 7.4, 2.0 Hz, 1H), 7.72–7.63 (m, 1H), 7.55 (d, *J* = 8.0 Hz, 1H), 7.37–7.08 (m, 8H), 7.07–7.00 (m, 2H), 6.70 (ddd, *J* = 8.2, 2.2, 0.9 Hz, 1H), 6.64–6.53 (m, 3H), 6.51 (ddd, *J* = 8.2, 2.2, 0.9 Hz, 1H), 6.41 (dd, *J* = 2.2, 2.2 Hz, 1H), 4.80 (ddd, *J* = 7.1, 7.1, 7.1 Hz, 1H), 4.65 (ddd, *J* = 7.8, 6.2, 6.2 Hz, 1H), 4.33 (dq, *J* = 7.0, 7.0 Hz, 1H), 3.65 (s, 3H), 3.64 (s, 3H), 3.42–3.12 (m, 4H), 2.98 (d, *J* = 6.2 Hz, 2H), 2.69 (ddd, *J* = 16.6, 7.2, 6.1 Hz, 1H), 2.56 (ddd, *J* = 16.6, 7.7, 7.7 Hz, 1H), 1.83 (s, 3H), 1.17 (d, *J* = 7.1 Hz, 3H). **^{13}C NMR** (126 MHz, $CDCl_3$): δ 171.5 (C_q), 171.4 (C_q), 171.0 (C_q), 170.2 (C_q), 160.3 (C_q), 151.3 (C_q), 151.2 (C_q), 149.6 (CH), 138.5 (CH), 136.8 (C_q), 136.3 (C_q), 135.8 (C_q), 129.6 (CH), 129.1 (CH), 128.5 (CH), 128.3 (C_q), 127.0 (CH), 122.6 (CH), 122.3 (CH), 121.1 (CH), 120.8 (CH), 118.8 (CH), 113.6 (CH), 111.9 (CH), 110.2 (C_q), 110.0 (CH), 107.3 (CH), 55.4 (CH_3), 53.7 (CH), 53.4 (CH), 52.3 (CH_3), 49.0 (CH), 37.8 (CH_2), 33.7 (CH_2), 27.6 (CH_2), 23.0 (CH_3), 20.4 (CH_2), 18.2 (CH_3). **IR** (ATR): 2919, 1748, 1628, 1541, 1521, 1472, 1141, 744 cm^{-1}. **MS** (ESI) *m/z* (relative intensity) 756 (100) $[M+Na]^+$, 734 (76) $[M+H]^+$. **HR-MS** (ESI) *m/z* calcd for $C_{41}H_{44}N_5O_8^+$ 734.3184 $[M+H]^+$, found 734.3178.

Methyl *N*-acetyl-1-(2-pyridyl)-2-(1-pyrenyl propionate-3-yl)-L-tryptophyl-L-alaninyl-L-phenylalaninate (6d):

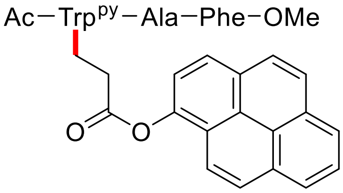

The general procedure B was followed using methyl *N*-acetyl-1-(2-pyridyl)-L-tryptophyl-L-alaninyl-L-phenylalaninate (84.6 mg, 150 µmol) and pyren-1-yl acrylate (62 mg, 0.23 mmol). Purification by column chromatography (hexanes/EtOAc 1:1→1:2) yielded **6d** (66 mg, 80 µmol, 53%) as a white solid. **m. p.** 237 °C (decomp.). **^{1}H NMR** (300 MHz, $CDCl_3$): δ 8.73 (dd, *J* = 5.2, 1.8 Hz, 1H), 8.23–7.92 (m, 7H), 7.84–7.71 (m, 2H), 7.71–7.58 (m, 3H), 7.45–7.35 (m, 2H), 7.27–7.15 (m, 5H), 7.06–6.91 (m, 2H), 6.69–6.53 (m, 3H), 4.87 (ddd, *J* = 7.0,7.0, 7.0 Hz, 1H), 4.66 (ddd, *J* = 6.7, 6.7, 6.7 Hz, 1H), 4.35 (dq, *J* = 7.0, 7.0 Hz, 1H), 3.64 (s, 3H), 3.58–3.45 (m, 2H), 3.43–3.18 (m, 2H), 3.09–2.83 (m, 4H), 1.66 (s, 3H), 1.14 (d, *J* = 6.9 Hz, 3H). **^{13}C NMR** (75 MHz, $CDCl_3$): δ 172.2 (C_q), 171.7 (C_q), 171.3 (C_q), 171.2 (C_q), 170.5 (C_q) 151.5 (C_q), 149.8 (CH), 144.1 (C_q), 138.7 (CH), 137.1 (C_q), 136.4 (C_q), 135.8 (C_q), 131.1 (C_q), 130.9 (C_q), 129.4 (C_q), 129.1 (CH), 128.5 (C_q), 128.5 (CH), 128.2 (CH), 127.2 (CH), 127.1 (CH), 127.0 (CH), 126.4 (CH), 125.5 (CH), 125.5 (C_q), 125.3 (CH), 124.9 (CH), 124.4 (C_q), 123.0 (C_q), 122.8 (CH), 122.5 (CH), 121.3 (CH), 121.1 (CH), 120.0 (CH), 119.6 (CH), 119.1 (CH), 110.7 (C_q), 110.2 (CH), 53.7 (CH), 53.3 (CH), 52.2 (CH_3), 49.0 (CH), 37.7 (CH_2), 33.7 (CH_2), 27.6 (CH_2), 22.8 (CH_3), 20.5 (CH_2), 18.1 (CH_3). **IR** (ATR): 3050, 2949, 2929, 1760, 1748, 1625, 1472, 1210, 848, 741 cm^{-1}. **MS** (ESI) *m/z* (relative intensity) 1677 (54) $[2M+Na]^+$, 850 (100) $[M+Na]^+$, 828 (86) $[M+H]^+$. **HR-MS** (ESI) *m/z* calcd for $C_{50}H_{46}N_5O_7^+$ 828.3392 $[M+H]^+$, found 828.3393.

Ethyl 3-(3-{[(3*S*,8*aS*)-1,4-dioxooctahydropyrrolo[1,2-*a*]pyrazin-3-yl]methyl}-1-[pyridin-2-yl]-1*H*-indol-2-yl)propanoate (6e):

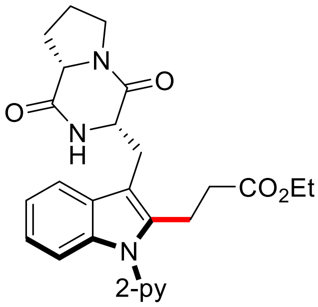

The general procedure B was followed using 3-{[1-(pyridin-2-yl)-1*H*-indol-3-yl]methyl}hexahydropyrrolo[1,2-*a*]pyrazine-1,4-dione (54 mg, 150 µmol) and ethyl acrylate (**2a**, 45 mg, 0.45 mmol) in 450 µL HOAc. Purification by column chromatography (EtOAc) yielded **6e** (57 mg, 123 µmol, 82%) as a pale yellow solid. **m.p.** 97 °C. **^{1}H NMR** (600 MHz, $CDCl_3$): δ 8.62 (ddd, *J* = 4.8, 2.0, 1.0 Hz, 1H), 7.91 (ddd, *J* = 7.5, 7.5, 1.8 Hz, 1H), 7.55 – 7.50 (m, 1H), 7.47 (dd, *J* = 7.9, 1.0 Hz, 1H), 7.37 – 7.31 (m, 1H), 7.29 (ddd, *J* = 7.5, 1.0, 1.0 Hz, 1H), 7.22 – 7.08 (m, 2H), 5.86 (s, 1H), 4.47 (dd, *J* = 11.5, 3.8 Hz, 1H), 4.10 – 3.97 (m, 3H), 3.74 (dd, *J* = 15.2, 3.8 Hz, 1H), 3.67 (ddd, *J* = 11.7, 7.9, 7.9 Hz, 1H), 3.57 (ddd, *J* = 11.7, 8.8, 3.1 Hz, 1H), 3.26 – 3.15 (m, 2H), 3.06 (ddd, *J* = 15.2, 11.7,

1.0 Hz, 1H), 2.43 (ddd, *J* = 15.9, 8.8, 7.1 Hz, 1H), 2.39 – 2.28 (m, 2H), 2.09 – 1.97 (m, 2H), 1.93 – 1.83 (m, 1H), 1.15 (t, *J* = 7.2 Hz, 3H). **^{13}C NMR** (126 MHz, $CDCl_3$): δ 172.1 (C_q), 169.3 (C_q), 165.5 (C_q), 150.8 (C_q), 149.7 (CH), 138.4 (CH), 137.3 (C_q), 136.8 (C_q), 127.6 (C_q), 122.8 (CH), 122.4 (CH), 121.0 (CH), 120.9 (CH), 118.1 (CH), 110.3 (CH), 108.5 (C_q), 60.6 (CH_2), 59.3 (CH), 54.5 (CH), 45.5 (CH_2), 34.0 (CH_2), 28.4 (CH_2), 25.7 (CH_2), 22.7 (CH_2), 20.6 (CH_2), 14.2 (CH_3). **IR** (ATR): 2979, 2930, 1718, 1585, 1469, 1436, 1164, 782, 732 cm^{-1}. **MS** (ESI) *m/z* (relative intensity): 943 (50) $[2M+Na]^+$,483 (100) $[M+Na]^+$, 461 (93) $[M+H]^+$. **HR-MS** (ESI) *m/z* calcd for $C_{26}H_{29}N_4O_4$ $[M+H]^+$: 461.2183; found: 461.2186.

Ethyl *N*-acetyl-1-(2-pyridyl)-2-(4-bromophenyl propionate-3-yl)-L-tryptophyl-L-tryptophyl-glycinate (6f):

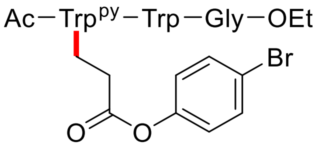

The general procedure B was followed using ethyl acetyl-1-(2-pyridyl)-L-tryptophyl-L-tryptophyl-glycinate (20.0 mg, 34 µmol) and 4-bromophenyl acrylate (11 mg, 100 µmol) in 100 µL HOAc. Purification by PTLC (EtOAc) yielded **6f** (16.4 mg, 20 µmol, 59%) as a white solid. **m. p.** 144 °C (decomp.). **^{1}H NMR** (400 MHz, DMSO-d_6): δ 9.01 (d, *J* = 6.7 Hz, 1H), 8.73 (ddd, *J* = 4.7, 2.0, 0.8 Hz, 1H), 8.71 (s, 1H), 8.44 (dd, *J* = 5.9, 5.9 Hz, 1H), 8.27–8.21 (m, 1H), 8.21–8.13 (m, 1H), 7.75 (ddd, *J* = 8.0, 1.0, 1.0 Hz, 1H), 7.65–7.53 (m, 4H), 7.38–7.30 (m, 3H), 7.28 (dm, *J* = 8.8 Hz, 2H), 7.21–7.12 (m, 2H), 6.73 (dm, *J* = 8.8 Hz, 2H), 4.89 (ddd, *J* = 12.0, 9.3, 2.3 Hz, 1H), 4.66 (ddd, *J* = 10.5, 6.8, 3.7 Hz, 1H), 4.11 (q, *J* = 7.1 Hz, 2H), 3.95–3.81 (m, 2H), 3.51–3.40 (m, 1H), 2.21–3.23 (m, 1H), 3.12–2.80 (m, 5H), 2.15–2.03 (m, 1H), 1.99 (s, 3H), 1.21 (t, *J* = 7.1 Hz, 3H). **^{13}C NMR** (126 MHz, DMSO-d_6): δ 175.2 (C_q), 172.5 (C_q), 172.0 (C_q), 171.3 (C_q), 170.8 (C_q), 170.0 (C_q), 157.7 (C_q), 151.1 (C_q), 150.3 (CH), 140.1 (CH), 137.0 (C_q), 135.6 (C_q), 135.4 (C_q), 132.4 (CH), 130.9 (C_q), 128.0 (CH), 125.6 (CH), 124.1 (CH), 123.5 (CH), 123.0 (CH), 121.3 (CH), 121.1 (CH), 119.8 (CH), 119.3 (C_q), 119.1 (CH), 118.2 (CH), 116.3 (CH), 111.4 (C_q), 110.5 (CH), 110.0 (C_q), 61.0 (CH_2), 54.9 (CH), 50.8 (CH), 41.4 (CH_2), 37.0 (CH_2), 29.0 (CH_2), 27.7 (CH_2), 23.3 (CH_3), 23.1 (CH_2), 14.6 (CH_3). **IR** (ATR): 3288, 1739, 1641, 1531, 1471, 1458, 1436, 1196, 1123, 741 cm^{-1}. **MS** (ESI) *m/z* (relative intensity) 1665 (14) $[2M+Na]^+$, 843 (100) $[M+Na]^+$, 821 (5) $[M+H]^+$. **HR-MS** (ESI) *m/z* calcd for $C_{42}H_{42}{}^{79}BrN_6O_7^+$ 821.2293 $[M+H]^+$, found 821.2283.

Ethyl *N*-acetyl-1-(2-pyridyl)-2-(2,2,2-trifluoroethyl propionate-3-yl)-L-tryptophyl-L-leucyl-L-tryptophanate (6g):

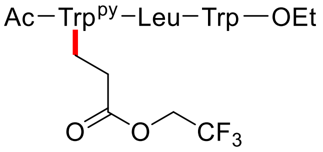

The general procedure B was followed using ethyl acetyl-1-(2-pyridyl)-L-tryptophyl-L-leucyl-L-tryptophanate (20.0 mg, 34 µmol) and 2,2,2-trifluoroethyl acrylate (24 mg, 155 µmol) in 120 µL HOAc. Purification by column chromatography (EtOAc) yielded peptide **6g** (14.1 mg, 18 µmol, 57%) as a white solid. **m. p.** 104 °C (decomp.). **^{1}H NMR** (600 MHz, CD_3OD): δ 8.60 (dd, *J* = 5.1, 1.9 Hz, 1H), 8.00 (ddd, *J* = 7.7, 7.7, 1.9 Hz, 1H), 7.65–7.61 (m, 2H), 7.48–7.43 (m, 2H), 7.29 (d, *J* = 8.3 Hz, 1H), 7.15–7.11 (m, 1H), 7.09–7.03 (m, 4H), 6.97 (ddd, *J* = 8.0, 7.0, 1.0 Hz, 1H), 4.73 (dd, *J* = 7.2, 7.2 Hz, 1H), 4.52 (dd, *J* = 7.0, 7.0 Hz, 1H), 4.44 (ddd, *J* = 8.8, 8.8, 8.8 Hz, 2H), 4.40 (dd, *J* = 9.5, 5.5 Hz, 1H), 4.00 (ddd, *J* = 7.1, 7.1, 7.1 Hz, 2H), 3.25 (dd, *J* = 14.4, 7.6 Hz, 1H), 3.22–3.15 (m, 2H), 3.15–3.07 (m, 3H), 2.48–2.33 (m, 2H), 1.91 (s, 3H), 1.61–1.54 (m, 1H), 1.50–1.38 (m, 2H), 1.06 (dd, *J* = 7.2, 7.2 Hz, 3H), 0.87 (d, *J* = 7.8 Hz, 3H), 0.86 (d, *J* = 7.8 Hz, 3H). **^{13}C NMR** (126 MHz, CD_3OD): δ 173.9 (C_q), 173.3 (C_q), 173.2 (C_q), 172.9 (C_q), 172.1 (C_q), 152.3 (C_q), 150.4 (CH), 140.6 (CH), 138.4 (C_q), 137.9 (C_q), 137.2 (C_q), 129.7 (C_q), 128.6 (C_q), 125.6 (C_q), 124.5 (CH), 124.1 (CH), 123.4 (CH), 123.3 (CH), 122.3 (CH), 121.6 (CH), 119.7 (CH), 119.7 (CH), 119.1 (CH), 112.2 (CH), 111.3 (C_q), 110.7 (CH), 110.4 (C_q), 62.2 (CH_2), 61.0 (CH_2), 55.4 (CH), 55.1 (CH), 52.9 (CH), 42.4 (CH_2), 33.9 (CH_2), 28.4 (CH_2), 28.1 (CH_2), 25.7 (CH), 23.4 (CH_3), 22.6 (CH_3), 22.3 (CH_3), 21.2 (CH_2), 14.3 (CH_3). **^{19}F NMR** (376 MHz, CD_3OD): δ –75.40–(–75.46) (m). **IR** (ATR): 3281, 2930, 1739, 1634, 1458, 1435, 1367, 1281, 1146, 740 cm^{-1}. **MS** (ESI) *m/z* (relative intensity) 827 (100) [M+Na]$^+$, 805 (30) [M+H]$^+$. **HR-MS** (ESI) *m/z* calcd for $C_{42}H_{47}F_3N_6O_7^+$ 805.3531 [M+H]$^+$, found 805.3550.

Ethyl acetyl-1-(2-pyridyl)-2-(butane-3-one-1-yl)-L-tryptophyl-L-tryptophyl-glycinate (6h):

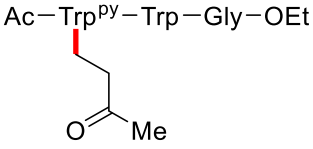

The general procedure B was followed using ethyl acetyl-1-(2-pyridyl)-L-tryptophyl-L-tryptophyl-glycinate (40.0 mg, 67 µmol) and but-3-en-2-one (14 mg, 0.20 mmol) in 200 µL HOAc. Purification by PTLC (EtOAc/THF 3:2, then MeCN) yielded **6h** (37 mg, 55 µmol, 83%) as a white solid. **m. p.** 105 °C (decomp.). **^{1}H NMR** (300 MHz, CD_3OD): δ 8.60 (ddd, *J* = 5.0, 1.9, 0.9 Hz, 1H), 8.03 (ddd, *J* = 7.7, 7.7, 2.0 Hz, 1H), 7.57 (ddd, *J* = 5.7, 3.2, 0.8 Hz, 1H), 7.54–7.44 (m, 3H), 7.29 (ddd, *J* = 8.1, 0.9. 0.9 Hz, 1H), 7.16–6.99 (m, 5H), 6.99–6.89 (m, 1H), 4.67 (ddd, *J* = 11.5, 5.4, 2.0 Hz, 2H), 4.16–4.04 (m, 2H), 3.77–3.70 (m, 2H), 3.27–3.14 (m, 2H), 3.15–2.99 (m, 4H), 2.58–2.38 (m, 2H), 1.94 (s, 3H), 1.82 (s, 3H), 1.25–1.17 (dd, *J* = 7.2, 7.2 Hz, 3H). **^{13}C NMR** (126 MHz, CD_3OD): δ 210.0 (C_q), 173.6 (C_q), 173.2 (C_q), 173.1 (C_q), 170.8 (C_q), 152.3 (C_q), 150.4 (CH), 140.6 (CH), 138.4 (C_q), 138.3 (C_q), 137.8 (C_q), 129.6 (C_q), 128.7 (C_q), 124.8 (CH), 124.1 (CH), 123.3 (CH), 123.2 (CH), 122.2 (CH), 121.6 (CH), 119.7

(CH), 119.5 (CH), 119.2 (CH), 112.2 (CH), 110.8 (CH), 110.7 (C_q), 110.4 (C_q), 62.3 (CH_2), 55.6 (CH), 55.4 (CH), 43.5 (CH_2), 42.1 (CH_2), 29.8 (CH_3), 28.8 (CH_2), 28.0 (CH_2), 22.6 (CH_3), 20.0 (CH_2), 14.5 (CH_3). **IR** (ATR): 3300, 2912, 1740, 1703, 1628, 1539, 1471, 1458, 1437, 1368, 1206, 742 cm^{-1}. **MS** (ESI) *m/z* (relative intensity) 687 (100) $[M+Na]^+$, 665 (26) $[M+H]^+$. **HR-MS** (ESI) *m/z* calcd for $C_{37}H_{41}N_6O_6^+$ 665.3082 $[M+H]^+$, found 665.3067.

Ethyl (*tert*-butoxycarbonyl)-1-(2-pyridyl)-2-(butane-3-one-1-yl)-L-tryptophyl-L-tryptophyl-glycinate (6i):

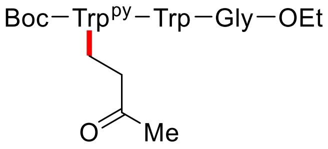

The general procedure B was followed using ethyl *tert*-butyloxycarbonyl-1-(2-pyridyl)-L-tryptophyl-L-tryptophylglycinate (50.0 mg, 77 µmol) and but-3-en-2-one (16 mg, 0.23 mmol) in 1.3 mL H_2O/HOAc (9:1). Purification by column chromatography (EtOAc) yielded **6i** (31 mg, 43 µmol, 56%) as a colorless oil. **^{1}H NMR** (600 MHz, $CDCl_3$): δ 8.72 (s, 1H), 8.65 (dd, *J* = 4.9, 2.0 Hz, 1H), 7.90 (ddd, *J* = 7.7, 7.7, 1.9 Hz, 1H), 7.61 (dd, *J* = 7.3, 3.4 Hz, 1H), 7.44 (d, *J* = 8.0 Hz, 1H), 7.36 (ddd, *J* = 7.8, 4.8, 1.0 Hz, 1H), 7.30–7.27 (m, 1H), 7.25 (d, *J* = 8.0 Hz, 1H), 7.19–7.13 (m, 2H), 7.12–7.05 (m, 2H), 6.91–6.85 (m, 1H), 6.82 (s, 1H), 6.70 (d, *J* = 6.4 Hz, 1H), 6.60–6.52 (m, 1H), 5.03 (d, *J* = 6.3 Hz, 1H), 4.71 (ddd, *J* = 8.1, 5.8, 5.8 Hz, 1H), 4.39 (ddd, *J* = 6.4, 6.4, 6.4 Hz, 1H), 4.10 (q, *J* = 7.1 Hz, 2H), 3.85 (dd, *J* = 17.6, 5.7 Hz, 1H), 3.73 (dd, *J* = 17.6, 5.7 Hz, 1H), 3.34–3.26 (m, 2H), 3.17 (dd, *J* = 14.7, 6.7 Hz, 1H), 3.14–3.05 (m, 2H), 2.96 (dd, *J* = 15.2, 6.1 Hz, 1H), 2.48 (dd, *J* = 7.6, 7.6 Hz, 2H), 1.96 (s, 3H), 1.22 (t, *J* = 7.2 Hz, 2H), 1.21 (s, 9H). **^{13}C NMR** (126 MHz, $CDCl_3$): δ 207.1 (C_q), 171.6 (C_q), 171.2 (C_q), 169.0 (C_q), 155.5 (C_q), 151.0 (C_q), 149.5 (CH), 138.6 (CH), 137.5 (C_q), 136.7 (C_q), 135.9 (C_q), 128.3 (C_q), 127.3 (C_q), 123.7 (CH), 122.5 (CH), 122.4 (CH), 121.8 (CH), 121.2 (CH), 120.9 (CH), 119.4 (CH), 118.7 (CH), 118.0 (CH), 111.2 (CH), 109.9 (CH), 109.2 (C_q), 109.0 (C_q), 80.2 (C_q), 61.1 (CH_2), 55.4 (CH), 53.7 (CH), 42.9 (CH_2), 41.3 (CH_2), 29.8 (CH_3), 28.0 (CH_3), 27.1 (CH_2), 26.7 (CH_2), 19.0 (CH_2), 14.1 (CH_3). **IR** (ATR): 3323, 2978, 2919, 1660, 1488, 1471, 1458, 1436, 1365, 1194, 1162, 1019, 739 cm^{-1}. **MS** (ESI) *m/z* (relative intensity) 1467 (10) $[2M+Na]^+$, 745 (100) $[M+Na]^+$, 723 (39) $[M+H]^+$. **HR-MS** (ESI) *m/z* calcd for $C_{40}H_{47}N_6O_7^+$ 723.3501 $[M+H]^+$, found 723.3493.

Ethyl acetyl-1-(2-pyridyl)-2-[(2-ethyl)*n*-hexyl propionate-3-yl]-L-tryptophyl-L-tryptophyl-glycinate (6j):

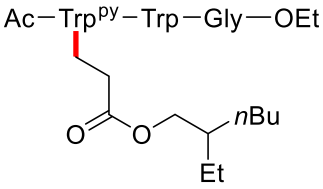

The general procedure B was followed using ethyl acetyl-1-(2-pyridyl)-L-tryptophyl-L-tryptophyl-glycinate (10.0 mg, 17 µmol) and 2-ethylhexyl acrylate (16 mg, 84 µmol) in 100 µL HOAc. Purification by PTLC (EtOAc) yielded **6j** (7.9 mg, 10 µmol, 60%) as a colorless oil. **^{1}H NMR** (500 MHz, CD_3OD): δ 8.61 (ddd, *J* = 4.9, 2.0, 0.9 Hz, 1H), 8.06 (ddd, *J* = 7.7, 7.7, 1.9 Hz, 1H), 7.60–7.57 (m, 1H), 7.55 (ddd, *J* = 7.9, 1.0, 1.0 Hz, 1H), 7.51–7.47 (m, 2H), 7.29 (ddd, *J* = 8.1, 0.9, 0.9 Hz, 1H), 7.18–7.15 (m, 1H), 7.10–7.07 (m, 2H), 7.06 (s, 1H), 7.04 (ddd, *J* = 8.1, 7.0, 1.2 Hz, 1H), 6.94 (ddd, *J* = 8.0, 7.0, 1.0 Hz, 1H), 4.73–4.66 (m, 1H), 4.63–4.59 (m, 1H), 4.11 (ddd, *J* = 7.1. 7.1, 7.1 Hz, 2H), 3.89–3.79 (m, 2H), 3.78–3.67 (m, 2H), 3.26–3.19 (m, 2H), 3.17 (dd, *J* = 7.6, 7.6 Hz, 2H), 3.08 (ddd, *J* = 14.7, 7.1, 3.9 Hz, 2H), 2.34–2.27 (m, 1H), 2.22 (ddd, *J* = 15.9, 7.8, 7.8 Hz, 1H), 1.82 (s, 3H), 1.38 (p, *J* = 6.0 Hz, 1H), 1.22 (dd, *J* = 7.2, 7.2 Hz, 3H), 1.20–1.15 (m, 8H), 0.85 (dd, *J* = 7.1, 7.1 Hz, 3H), 0.77 (dd, *J* = 7.4, 7.4 Hz, 3H). **^{13}C NMR** (126 MHz, CD_3OD): δ 174.1 (C_q), 173.5 (C_q), 173.2 (C_q), 173.0 (C_q), 170.7 (C_q), 152.4 (C_q), 150.5 (CH), 140.5 (CH), 138.4 (C_q), 137.8 (C_q), 137.7 (C_q), 129.6 (C_q), 128.8 (C_q), 124.8 (CH), 124.1 (CH), 123.4 (CH), 123.1 (CH), 122.2 (CH), 121.7 (CH), 119.7 (CH), 119.7 (CH), 119.2 (CH), 112.2 (CH), 111.2 (C_q), 110.7 (CH), 110.5 (C_q), 67.9 (CH_2), 62.2 (CH_2), 55.6 (CH), 55.4 (CH), 42.2 (CH_2), 40.1 (CH), 34.5 (CH_2), 31.4 (CH_2), 30.0 (CH_2), 28.7 (CH_2), 27.9 (CH_2), 24.8 (CH_2), 24.0 (CH_2), 22.6 (CH_3), 21.5 (CH_2), 14.5 (CH_3), 14.4 (CH_3), 11.3 (CH_3). **IR** (ATR): 2958, 2927, 2858, 1732, 1634, 1583, 1470, 1456, 1436, 1193, 740 cm^{-1}. **MS** (ESI) *m/z* (relative intensity) 1579 (4) $[2M+Na]^+$, 801 (100) $[M+Na]^+$, 779 (20) $[M+H]^+$. **HR-MS** (ESI) *m/z* calcd for $C_{44}H_{55}N_6O_7^+$ 779.4127 $[M+H]^+$, found 779.4122.

Ethyl acetyl-1-(2-pyridyl)-2-(octane-3-one-1-yl)-L-tryptophyl-L-leucyl-L-tryptophanate (6k):

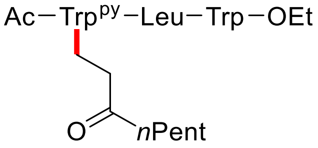

The general procedure B was followed using ethyl acetyl-1-(2-pyridyl)-L-tryptophyl-L-leucyl-L-tryptophanate (15.0 mg, 23 µmol) and oct-1-en-3-one (15 mg, 115 µmol) in 150 µL HOAc. Purification by PTLC (hexanes/EtOAc 1:4) yielded **6k** (9.2 mg, 12 µmol, 51%) as a colorless oil. **^{1}H NMR** (500 MHz, CD_3OD): δ 8.60 (ddd, *J* = 5.0, 2.0, 0.8 Hz, 1H), 8.01 (ddd, *J* = 7.7, 7.7, 1.9 Hz, 1H), 7.63 (ddd, *J* = 8.9, 8.0, 0.9 Hz, 2H), 7.49–7.43 (m, 2H), 7.29 (ddd, *J* = 8.2, 0.9, 0.9 Hz, 1H), 7.14–7.09 (m, 1H), 7.09–7.01 (m, 4H), 6.97 (ddd, *J* = 8.0, 7.0, 1.0 Hz, 1H), 4.71 (dd, *J* = 7.2, 7.2 Hz, 1H), 4.55 (dd, *J* = 6.9, 6.9 Hz, 1H), 4.42 (dd, *J* = 9.5, 5.6 Hz, 1H), 4.05–3.98 (m, 2H), 3.27–3.21 (m, 2H), 3.22–3.03 (m, 2H), 3.03–2.97 (m, 2H), 2.51–2.36 (m, 3H), 2.20 (ddd, *J* = 7.3, 7.3, 2.1 Hz, 1H), 1.91 (s, 3H), 1.62–1.15 (m, 9H), 1.08 (dd, *J* = 7.1, 7.1 Hz, 3H), 0.92–0.84 (m, 6H), 0.81 (dd, *J* = 7.3, 7.3 Hz, 3H). **^{13}C NMR** (126 MHz, CD_3OD): δ 212.5 (C_q), 174.2 (C_q), 173.5 (C_q), 173.5 (C_q), 173.1 (C_q), 152.6 (C_q), 150.5 (CH), 140.8 (CH), 138.5 (C_q), 138.4 (C_q), 138.0 (C_q), 129.8 (C_q), 128.7 (C_q), 124.6 (CH), 124.2 (CH), 123.6 (CH), 123.3 (CH), 122.4 (CH), 121.6 (CH), 119.8 (CH), 119.7 (CH), 119.2 (CH), 112.3 (CH), 110.9 (C_q), 110.8 (CH), 110.5 (C_q), 62.2 (CH_2), 55.4 (CH), 55.2 (CH), 52.9 (CH), 43.5 (CH_2), 42.5 (CH_2), 42.3

(CH_2), 32.6, (CH_2) 28.3 (CH_2), 28.2 (CH_2), 25.6 (CH), 24.5 (CH_2), 23.5 (CH_2), 23.4 (CH_3), 22.6 (CH_3), 22.2 (CH_3), 20.0 (CH_2), 14.3 (CH_3), 14.3 (CH_3). **IR** (ATR): 2955, 2926, 1736, 1711, 1636, 1583, 1566, 1458, 1436, 1369, 1206, 741 cm^{-1}. **MS** (ESI) *m/z* (relative intensity) 799 (100) $[M+Na]^+$, 777 (28) $[M+H]^+$. **HR-MS** (ESI) *m/z* calcd for $C_{45}H_{57}N_6O_6^+$ 777.4334 $[M+H]^+$, found 777.4303.

Methyl ((*S*)-2-acetamido-3-{2-[3-(3-methoxyphenoxy)-3-oxopropyl]-1-[pyridin-2-yl]-1*H*-indol-3-yl}propanoyl)-*L*-methioninate (6l):

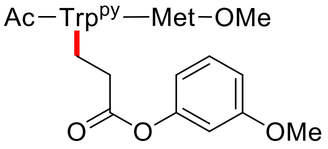

The general procedure B was followed using methyl N^a-acetyl-1-(pyridin-2-yl)-*L*-tryptophyl-*L*-methioninate (70.2 mg, 150 µmol) and 3-methoxyphenyl acrylate (40 mg, 0.23 mmol) in HOAc (450 µL). Purification by column chromatography (EtOAc) yielded **6l** (55.2 mg, 86 µmol, 57%) as a white solid. **m.p.** 88 °C. **^{1}H NMR** (600 MHz, $CDCl_3$): δ 8.66 (ddd, *J* = 4.9, 2.0, 0.9 Hz, 1H), 7.93 (ddd, *J* = 7.8, 7.8, 2.0 Hz, 1H), 7.68–7.62 (m, 1H), 7.58 (ddd, *J* = 7.8, 0.9, 0.9 Hz, 1H), 7.36 (ddd, *J* = 7.5, 4.9, 0.9 Hz, 1H), 7.32–7.27 (m, 1H), 7.18 (dd, *J* =8.2, 8.2 Hz, 1H), 7.16–7.12 (m, 2H), 6.72 (ddd, *J* = 8.2, 2.2, 0.8 Hz, 1H), 6.55 (ddd, *J* = 8.2, 2.2, 0.8 Hz, 1H), 6.52 (d, *J* = 7.4 Hz, 1H), 6.47 (dd, *J* = 2.2 Hz, 1H), 6.42 (d, *J* = 7.1 Hz, 1H), 4.84 (ddd, *J* = 9.1, 7.4, 6.0 Hz, 1H), 4.44 (ddd, *J* = 7.1, 7.1, 5.1 Hz, 1H), 3.70 (s, 3H), 3.51 (s, 3H), 3.43–3.34 (m, 3H), 3.15 (dd, *J* = 14.4, 9.1 Hz, 1H), 2.75 (ddd, *J* = 16.7, 6.7, 6.7 Hz, 1H), 2.56 (ddd, *J* = 16.7, 7.8, 7.8 Hz, 1H), 2.29 (ddd, *J* = 13.3, 9.1, 6.1 Hz, 1H), 2.23 (ddd, *J* = 13.3, 9.1, 6.1 Hz, 1H), 1.96 (s, 3H), 1.94 (s, 3H), 1.83–1.66 (m, 2H). **^{13}C NMR** (126 MHz, $CDCl_3$): δ 171.2 (C_q), 171.0 (C_q), 170.9 (C_q), 169.8 (C_q), 160.3 (C_q), 151.3 (C_q), 151.3 (C_q), 149.6 (CH), 138.5 (CH), 136.8 (C_q), 136.3 (C_q), 129.5 (CH), 128.3 (C_q), 122.4 (CH), 122.2 (CH), 121.0 (CH), 120.8 (CH), 118.6 (CH), 113.6 (CH), 111.7 (CH), 110.2 (C_q), 109.9 (CH), 107.4 (CH), 55.4 (CH_3), 53.7 (CH), 52.3 (CH_3), 51.6 (CH), 33.5 (CH_2), 31.9 (CH_2), 29.6 (CH_2), 28.1 (CH_2), 23.2 (CH_3), 20.4 (CH_2), 15.4 (CH_3). **IR** (ATR): 3277, 2921, 1744, 1645, 1459, 1436, 1137, 741 cm^{-1}. **MS** (ESI) *m/z* (relative intensity): 1316 (58) $[2M+Na]^+$, 669 (100) $[M+Na]^+$, 647 (82) $[M+H]^+$. **HR-MS** (ESI) *m/z* calcd for $C_{34}H_{39}N_4O_7S$ $[M+H]^+$: 647.2534; found: 647.2527.

Methyl *N*-{[*S*]-2-acetamido-3-[2-(3-oxobutyl)-1-(pyridin-2-yl)-1*H*-indol-3-yl]propanoyl}-*S*-(4-methylbenzyl)-*L*-cysteinyl-*L*-leucinate (6m):

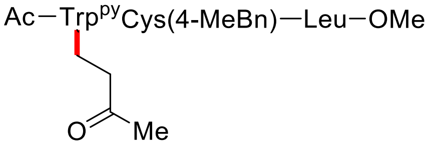

The general procedure B was followed using methyl methyl *N*-[$N^{α}$-acetyl-1-(pyridin-2-yl)-L-tryptophyl]-*S*-(4-methylbenzyl)-*L*-cysteinyl-*L*-leucinate (97 mg, 150 µmol) and but-3-en-2-one (32 mg, 0.45 mmol) in HOAc (300 µL). Purification by column chromatography (CH_2Cl_2/MeOH 40:1→30:1) yielded **6m** (96 mg, 132 µmol, 88%) as a white solid. **m. p.** 143 °C. **^{1}H NMR** (600 MHz, $CDCl_3$): δ 8.64–8.51 (m, 1H), 7.89 (ddd, *J* = 7.7, 7.7, 1.9 Hz, 1H), 7.69–7.60 (m, 1H), 7.49 (d, *J* = 7.9 Hz, 1H), 7.33 (ddd, *J* = 7.5, 4.8, 1.0 Hz, 1H), 7.23–7.19 (m, 1H), 7.17 (d, *J* = 7.8 Hz, 2H), 7.12–7.06 (m, 2H), 7.06 (d, *J* = 7.8 Hz, 2H), 6.94–6.89 (m, 2H), 6.79 (d, *J* = 6.5 Hz, 1H), 4.79 (ddd, *J* = 6.5, 6.5, 6.5 Hz, 1H), 4.49–4.35 (m, 2H), 3.68–3.65 (m, 5H), 3.30 (dd, *J* = 14.7, 7.0 Hz, 1H), 3.23 (dd, *J* = 14.7, 6.9 Hz, 1H), 3.10 (dd, *J* = 7.1, 7.1 Hz, 2H), 2.89 (dd, *J* = 14.1, 4.9 Hz, 1H), 2.63–2.43 (m, 3H), 2.28 (s, 3H), 1.98 (s, 3H), 1.94 (s, 3H), 1.71–1.47 (m, 3H), 0.90 (d, *J* = 5.9 Hz, 3H), 0.85 (d, *J* = 5.9 Hz, 3H). **^{13}C NMR** (126 MHz, $CDCl_3$): δ 207.9 (C_q), 172.5 (C_q), 171.2 (C_q), 170.5 (C_q), 169.5 (C_q), 151.3 (C_q), 149.5 (CH), 138.4 (CH), 137.1 (C_q), 136.8 (C_q), 136.6 (C_q), 134.9 (C_q), 129.1 (CH), 128.8 (CH), 128.2 (C_q), 122.3 (CH), 122.2 (CH), 121.3 (CH), 120.7 (CH), 118.5 (CH), 109.9 (CH), 109.4 (C_q), 54.1 (CH), 52.3 (CH), 52.2 (CH), 51.3 (CH_3), 42.7 (CH_2), 40.8 (CH_2), 36.4 (CH_2), 33.7 (CH_2), 30.0 (CH_3), 27.5 (CH_2), 24.8 (CH), 23.1 (CH_3), 22.8 (CH_3), 21.9 (CH_3), 21.1 (CH_3), 19.1 (CH_2). **IR** (ATR): 3283, 2955, 1743, 1713, 1640, 1542, 1460, 1437, 1204, 738 cm^{-1}. **MS** (ESI) *m/z* (relative intensity): 1477 (12) $[2M+Na]^+$, 750 (89) $[M+Na]^+$, 728 (100) $[M+H]^+$. **HR-MS** (ESI) *m/z* calcd for $C_{40}H_{50}N_5O_6S$ $[M+H]^+$: 728.3476; found: 728.3473.

Methyl (*S*)-2-[(*S*)-2-acetamido-5-amino-5-oxopentanamido]-3-(2-(3-ethoxy-3-oxopropyl)-1-(pyridin-2-yl)-1H-indol-3-yl)propanoate(6n):

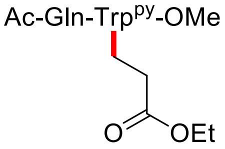

The general procedure B was followed using methyl $N^{α}$-(acetyl-*L*-glutaminyl)-1-(pyridin-2-yl)-*L*-tryptophanate (70 mg, 150 µmol) and ethyl acrylate (45 mg, 0.45 mmol). Purification by column chromatography (CH_2Cl_2/MeOH 20:1) yielded **6n** (54 mg, 96 µmol, 64%) as a white solid. **m. p.** 206 °C. **^{1}H NMR** (300 MHz, $CDCl_3$): δ 8.65 (d, *J* = 4.7 Hz, 1H), 7.95 (dd, *J* = 7.9, 7.4 Hz, 1H), 7.61–7.58 (m, 2H), 7.52 (d, *J* = 7.9 Hz, 1H), 7.39–7.35 (dd, *J* = 7.4, 4.7 Hz, 1H), 7.30–7.25 (m, 1H), 7.18–7.15 (m, 2H), 6.64 (d, *J* = 6.7 Hz, 1H), 6.32 (s, 1H), 5.61 (s, 1H), 4.95 (ddd, *J* = 6.4, 6.4, 6.4 Hz, 1H), 4.48 (ddd, *J* = 6.7, 6.7, 6.7 Hz, 1H), 4.07 (q, *J* = 7.0, 2H), 3.76 (s, 3H), 3.42 (dd, *J* = 6.4 Hz, 5.5 Hz, 1H), 3.33–3.18 (m, 3H), 2.40–2.22 (m, 4H), 2.11–2.04 (m, 1H), 2.01–1.96 (m, 1H), 1.94 (s, 3H), 1.18 (t, *J* = 7.0 Hz, 3H). **^{13}C NMR** (101 MHz, $CDCl_3$): δ 175.2 (C_q), 172.9 (C_q), 172.6 (C_q), 171.3 (C_q), 170.1 (C_q), 151.2 (C_q), 149.8 (CH), 138.7 (CH), 136.91 (C_q), 136.90 (C_q), 128.2 (C_q), 122.5 (CH), 122.4 (CH), 121.1 (CH), 120.8 (CH), 118.5 (CH), 110.0 (CH), 109.3 (C_q), 60.8 (CH_2), 52.9 (CH), 52.5 (CH_3), 52.2 (CH), 33.4 (CH_2), 31.6 (CH_2), 29.0 (CH_2), 26.8 (CH_2), 23.1 (CH_3), 20.1 (CH_2), 14.1 (CH_3). **IR** (ATR): 3394, 3280, 3226, 3064, 2952, 1734, 1687, 1633, 1585, 1539. **MS** (ESI): m/z (relative intensity): 588

(100) [M+Na]$^+$, 566 (20) [M+H]$^+$. **HR-MS** (ESI) m/z calcd for $C_{29}H_{35}N_5O_7Na$ [M+Na]$^+$: 588.2434; found: 588.2431.

Benzyl (*S*)-2-({*S*}-2-acetamido-6-{[(benzyloxy)carbonyl]amino}hexanamido)-3-[2-(3-oxobutyl)-1-(pyridin-2-yl)-1*H*-indol-3-yl]propanoate (6o):

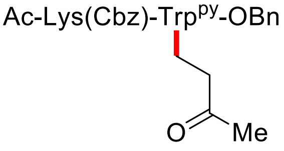

The general procedure B was followed using benzyl N^{α}-{N^2-acetyl-N^6-[(benzyloxy)carbonyl]-*L*-lysyl}1-(pyridin-2-yl)-*L*-tryptophanate (99 mg, 150 µmol) and but-3-en-2-one (32 mg, 0.45 mmol) in HOAc (450 µL). Purification by column chromatography (CH_2Cl_2/MeOH 40:1) yielded **6o** (82 mg, 110 µmol, 73%) as a white solid. **m. p.** 178 °C. **^{1}H NMR** (600 MHz, $CDCl_3$): δ 8.60 (dd, *J* = 5.0, 1.9 Hz, 1H), 7.89 (ddd, *J* = 7.7, 7.7, 1.9 Hz, 1H), 7.57–7.50 (m, 1H), 7.38 (d, *J* = 7.9 Hz, 1H), 7.35–7.16 (m, 12H), 7.15–7.07 (m, 2H), 7.02–6.96 (m, 1H), 6.21 (d, *J* = 7.1 Hz, 1H), 5.16–5.00 (m, 5H), 4.95 (ddd, *J* = 7.0, 7.0, 7.0 Hz, 1H), 4.39 (ddd, *J* = 7.1, 7.1, 7.1 Hz, 1H), 3.34 (dd, *J* = 14.8, 6.1 Hz, 1H), 3.27 (dd, *J* = 14.8, 7.6 Hz, 1H), 3.15–3.06 (m, 4H), 2.57–2.34 (m, 2H), 1.96 (s, 3H), 1.87 (s, 3H), 1.79–1.69 (m, 1H), 1.60–1.51 (m, 1H), 1.48–1.35 (m, 2H), 1.32–1.24 (m, 2H). **^{13}C NMR** (126 MHz, $CDCl_3$): δ 208.0 (C_q), 171.7 (C_q), 171.5 (C_q), 169.7 (C_q), 156.4 (C_q), 151.1 (C_q), 149.6 (CH), 138.5 (CH), 137.2 (C_q), 136.8 (C_q), 136.6 (C_q), 135.1 (C_q), 128.4 (CH), 128.4 (CH), 128.2 (CH), 128.2 (CH), 128.2 (C_q), 127.9 (CH), 127.9 (CH), 122.4 (CH), 122.3 (CH), 121.0 (CH), 120.8 (CH), 118.4 (CH), 109.9 (CH), 109.0 (C_q), 67.4 (CH_2), 66.5 (CH_2), 52.9 (CH), 52.5 (CH), 42.7 (CH_2), 40.4 (CH_2), 32.2 (CH_2), 30.0 (CH_3), 29.3 (CH_2), 27.1 (CH_2), 23.2 (CH_3), 22.0 (CH_2), 19.0 (CH_2). **IR** (ATR): 3297, 2934, 1712, 1649, 1530, 1471, 1438, 1264, 1246, 735 cm^{-1}. **MS** (ESI) *m/z* (relative intensity): 784 (65) [M+K]$^+$, 769 (96) [M+Na]$^+$, 746 (100) [M+H]$^+$. **HR-MS** (ESI) *m/z* calcd for $C_{43}H_{48}N_5O_7$ [M+H]$^+$: 746.3548; found: 746.3540.

Methyl *N*-({*S*}-3-{2-[3-([1,1'-biphenyl]-2-yloxy)-3-oxopropyl]-1-[pyridin-2-yl]-1*H*-indol-3-yl}-2-acetamidopropanoyl)-*O*-benzyl-*L*-threonyl-*L*-phenylalaninate (6p):

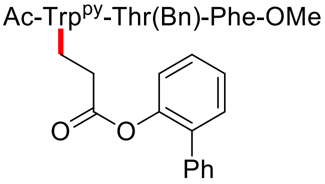

The general procedure B was followed using methyl methyl *N*-[N^{α}-acetyl-1-(pyridin-2-yl)-*L*-tryptophyl]-*O*-benzyl-*L*-threonyl-*L*-phenylalaninate (101 mg, 150 µmol) and 2-phenylphenyl acrylate (52 mg, 0.23 mmol) in HOAc (1.5 mL). Purification by column chromatography (CH_2Cl_2/MeOH 40:1→30:1) yielded **6p** (105 mg, 117 µmol, 78%) as a white solid. **m. p.** 102 °C. **^{1}H NMR** (600 MHz, $CDCl_3$): δ 8.54 (dd, *J* = 4.9, 1.9 Hz, 1H), 7.88 (ddd, *J* = 7.7, 7.7, 2.0 Hz, 1H), 7.70–7.60 (m, 1H), 7.52

(ddd, *J* = 7.7, 0.9, 0.9 Hz, 1H), 7.34 (dd, *J* = 7.3, 2.0 Hz, 1H), 7.33–7.22 (m, 14H), 7.18–7.15 (m, 3H), 7.10–7.05 (m, 2H), 7.02–7.00 (m, 2H), 6.97–6.92 (m, 2H), 6.64 (d, *J* = 6.0 Hz, 1H), 6.51 (d, *J* = 7.4 Hz, 1H), 4.79 (ddd, *J* = 7.4, 7.4, 7.4 Hz, 1H), 4.56–4.51 (m, 2H), 4.47 (d, *J* = 11.5 Hz, 1H), 4.30 (dd, *J* = 6.0, 3.4 Hz, 1H), 4.01 (qd, *J* = 6.4, 3.4 Hz, 1H), 3.62 (s, 3H), 3.33 (dd, *J* = 14.6, 6.3 Hz, 1H), 3.20–3.05 (m, 3H), 2.96 (dd, *J* = 13.9, 5.6 Hz, 1H), 2.86 (dd, *J* = 13.9, 7.4 Hz, 1H), 2.53 (ddd, *J* = 17.1, 7.9, 5.9 Hz, 1H), 2.43 (ddd, *J* = 17.1, 7.9, 7.9 Hz, 1H), 1.88 (s, 3H), 0.95 (d, *J* = 6.4 Hz, 3H). **^{13}C NMR** (126 MHz, $CDCl_3$): δ 171.3 (C_q), 171.2 (C_q), 171.1 (C_q), 169.9 (C_q), 168.1 (C_q), 151.2 (C_q), 149.4 (CH), 147.5 (C_q), 138.3 (CH), 137.8 (C_q), 137.3 (C_q), 136.8 (C_q), 136.2 (C_q), 135.7 (C_q), 134.6 (C_q), 130.6 (CH), 128.9 (CH), 128.7 (CH), 128.4 (CH), 128.3 (CH), 128.3 (CH), 128.2 (C_q), 128.1 (CH), 127.6 (CH), 127.6 (CH), 127.3 (CH), 126.9 (CH), 126.2 (CH), 122.8 (CH), 122.4 (CH), 122.1 (CH), 121.2 (CH), 120.7 (CH), 118.6 (CH), 109.9 (C_q), 109.7 (CH), 73.8 (CH), 71.2 (CH_2), 55.7 (CH), 53.9 (CH), 53.6 (CH), 52.1 (CH_3), 37.6 (CH_2), 33.5 (CH_2), 27.8 (CH_2), 23.1 (CH_3), 20.1 (CH_2), 14.4 (CH_3). **IR** (ATR): 3284, 3063, 1746, 1637, 1472, 1368, 1189, 733 cm^{-1}. **MS** (ESI) *m/z* (relative intensity): 1822 (33) $[2M+Na]^+$, 922 (7021) $[M+Na]^+$, 900 (100) $[M+H]^+$, **HR-MS** (ESI) *m/z* calcd for $C_{54}H_{54}N_5O_8$ $[M+H]^+$: 900.3967; found: 900.3964.

Benzyl (*S*)-3-{[*S*]-2-acetamido-3-[2-(3-oxobutyl)-1-(pyridin-2-yl)-1*H*-indol-3-yl]propanamido}-4-{[(*S*)-1-methoxy-1-oxo-3-phenylpropan-2-yl]amino}-4-oxobutanoate (6q):

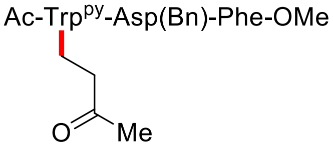

The general procedure B was followed using benzyl (*S*)-3-{[*S*]-2-acetamido-3-[1-(pyridin-2-yl)-1*H*-indol-3-yl]propanamido}-4-{[(*S*)-1-methoxy-1-oxo-3-phenylpropan-2-yl]amino}-4-oxobutanoate (99 mg, 150 µmol) and but-3-en-2-one (32 mg, 0.45 mmol) in HOAc (600 µL). Purification by column chromatography (CH_2Cl_2/MeOH 40:1) yielded **6q** (93 mg, 123 µmol, 82%) as a white solid. **m. p.** 96 °C. **^{1}H NMR** (600 MHz, $CDCl_3$): δ 8.60 (d, *J* = 3.2 Hz, 1H), 7.88 (ddd, *J* = 7.7, 7.7, 1.8 Hz, 1H), 7.61 (dd, *J* = 7.2, 1.6 Hz, 1H), 7.47 (d, *J* = 7.9 Hz, 1H), 7.35–7.25 (m, 8H), 7.23–7.18 (m, 2H), 7.13–7.05 (m, 5H), 7.03 (d, *J* = 7.5 Hz, 1H), 6.63 (d, *J* = 6.5 Hz, 1H), 5.03 (d, *J* = 12.3 Hz, 1H), 4.99 (d, *J* = 12.3 Hz, 1H), 4.76–4.66 (m, 2H), 4.61 (ddd, *J* = 7.5, 7.5, 5.9 Hz, 1H), 3.61 (s, 3H), 3.27 (dd, *J* = 14.7, 7.1 Hz, 1H), 3.18 (dd, *J* = 14.7, 6.9 Hz, 1H), 3.11 (t, *J* = 7.2 Hz, 2H), 3.04 (dd, *J* = 14.0, 5.9 Hz, 1H), 2.98 (dd, *J* = 14.0, 7.5 Hz, 1H), 2.90 (dd, *J* = 17.3, 4.1 Hz, 1H), 2.61–2.42 (m, 3H), 1.98 (s, 3H), 1.89 (s, 3H). **^{13}C NMR** (126 MHz, $CDCl_3$): δ 207.7 (C_q), 171.4 (C_q), 171.2 (C_q), 171.2 (C_q), 170.5 (C_q), 169.4 (C_q), 151.2 (C_q), 149.5 (CH), 138.4 (CH), 137.1 (C_q), 136.8 (C_q), 136.0 (C_q), 135.2 (C_q), 129.1 (CH), 128.4 (CH), 128.4 (CH), 128.2 (C_q), 128.2 (CH), 128.0 (CH), 126.9 (CH), 122.4 (CH), 122.3 (CH), 121.2 (CH), 120.8 (CH), 118.5 (CH), 109.9 (CH), 109.4 (C_q), 66.7 (CH_2), 54.1 (CH), 53.8 (CH), 52.2

(CH_3), 49.1 (CH), 42.7 (CH_2), 37.5 (CH_2), 35.8 (CH_2), 30.0 (CH_3), 27.2 (CH_2), 23.0 (CH_3), 19.0 (CH_2). **IR** (ATR): 3296, 3061, 2950, 1738, 1715, 1643, 1526, 1471, 1169, 742 cm^{-1}. **MS** (ESI) *m/z* (relative intensity): 1542 (29) $[2M+Na]^+$, 782 (100) $[M+Na]^+$, 760 (82) $[M+H]^+$. **HR-MS** (ESI) *m/z* calcd for $C_{43}H_{46}N_5O_8$ $[M+H]^+$: 760.3341; found: 760.3338.

Methyl acetyl-1-(2-pyridyl)-2-[*O*-(ethyl acetyl L-phenylalaninate-4-yl) propionate-3-yl]-L-tryptophanate (8a)

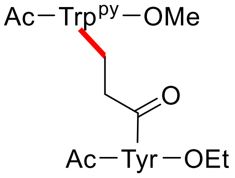

The general procedure C was followed using methyl acetyl-1-(2-pyridyl)-L-tryptophanate (30.0 mg, 89 µmol) and ethyl acetyl-L-tyrosinate acrylate (29.9 mg, 99 µmol) in 270 µL HOAc. Purification by column chromatography (EtOAc/MeOH 25:1) yielded peptide **8a** (34 mg, 53 µmol, 59%) as a white solid. **m. p.**: 111 °C. **^{1}H NMR** (300 MHz, $CDCl_3$): δ 8.66 (ddd, *J* = 5.0, 1.9, 0.8 Hz, 1H), 7.94 (td, *J* = 7.7, 2.0 Hz, 1H), 7.60–7.53 (m, 1H), 7.51 (ddd, *J* = 8.0, 1.0, 1.0 Hz, 1H), 7.37 (ddd, *J* = 7.5, 4.9, 1.0 Hz, 1H), 7.34–7.27 (m, 1H), 7.21–7.13 (m, 2H), 7.06 (dm, *J* = 8.4 Hz, 2H), 6.86 (dm, *J* = 8.4 Hz, 2H), 6.22 (d, *J* = 7.8 Hz, 1H), 5.94 (d, *J* = 7.7 Hz, 1H), 4.97 (ddd, *J* = 7.9, 5.9, 5.9 Hz, 1H), 4.83 (ddd, *J* = 7.8, 5.8, 5.8 Hz, 1H), 4.16 (q, *J* = 7.2 Hz, 2H), 3.70 (s, 3H), 3.45–3.21 (m, 4H), 3.16–3.02 (m, 2H), 2.64–2.54 (m, 2H), 1.99 (s, 3H), 1.91 (s, 3H), 1.24 (t, *J* = 7.1 Hz, 3H). **^{13}C NMR** (126 MHz, $CDCl_3$): δ 172.6 (C_q), 171.5 (C_q), 171.0 (C_q), 169.9 (C_q), 169.6 (C_q), 151.3 (C_q), 149.8 (CH), 149.6 (C_q), 138.7 (CH), 136.8 (C_q), 136.5 (C_q), 133.7 (C_q), 130.3 (CH), 128.8 (C_q), 122.7 (CH), 122.5 (CH), 121.5 (CH), 121.0 (CH), 121.0 (CH), 118.7 (CH), 110.2 (CH), 109.8 (C_q), 61.8 (CH_2), 53.3 (CH), 53.0 (CH), 52.6 (CH_3), 37.5 (CH_2), 34.1 (CH_2), 27.4 (CH_2), 23.4 (CH_3), 23.3 (CH_3), 20.6 (CH_2), 14.4 (CH_3). **IR** (ATR): 3315, 2927, 1750, 1726, 1652, 1544, 1441, 1369, 1202, 1138, 1138, 740 cm^{-1}. **MS** (ESI) *m/z* (relative intensity) 665 (100) $[M+Na]^+$, 643 (4) $[M+H]^+$, 528 (47). **HR-MS** (ESI) *m/z* calcd for $C_{35}H_{39}N_4O_8^+$ 643.2762 $[M+H]^+$, found 643.2766.

Methyl *N*-acetyl-1-(2-pyridyl)-2-{[(*N*-acetylphenylalaninyl)-2-aminoethane-1-yl] propionate-3-yl}-L-tryptophanate (8b):

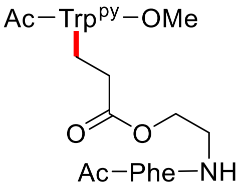

The general procedure C was followed using methyl acetyl-1-(2-pyridyl)-L-tryptophanate (**1a**, 50.6 mg, 150 µmol) and *N*-acetyl-L-phenylalaninyl-2-aminoethane-1-yl acrylate (46 mg, 165 µmol). Purification by column chromatography (CH_2Cl_2/MeOH 40:1→30:1) yielded **8b** (64 mg, 101 µmol, 67%) as a white

solid. **m. p.** 145 °C.· ^{1}**H NMR** (400 MHz, $CDCl_3$): δ 8.64 (ddd, *J* = 4.9, 2.0, 0.8 Hz, 1H), 7.94–7.84 (m, 1H), 7.53–7.45 (m, 1H), 7.43 (dd, *J* = 8.0, 0.9 Hz, 1H), 7.33 (ddd, *J* = 7.5, 4.9, 1.0 Hz, 1H), 7.30–7.26 (m, 1H), 7.24–7.18 (m, 2H), 7.18–7.09 (m, 6H), 6.70 (d, *J* = 8.0 Hz, 1H), 6.57 (d, *J* = 8.1 Hz, 1H), 4.84 (ddd, *J* = 7.8, 6.4, 6.4 Hz, 1H), 4.69 (ddd, *J* = 8.0, 6.9, 6.9 Hz, 1H), 4.11–3.82 (m, 2H), 3.62 (s, 3H), 3.40 (dddd, *J* = 14.4, 6.2, 6.2, 3.2 Hz, 1H), 3.34–3.26 (m, 2H), 3.25–3.10 (m, 3H), 3.09–2.93 (m, 2H), 2.43–2.21 (m, 2H), 1.97 (s, 3H), 1.94 (s, 3H). ^{13}**C NMR** (126 MHz, $CDCl_3$): δ 172.7 (C_q), 171.9 (C_q), 171.2 (C_q), 170.1 (C_q), 169.7 (C_q), 151.0 (C_q), 149.7 (CH), 138.5 (CH), 136.6 (C_q), 136.6 (C_q), 136.5 (C_q), 129.1 (CH), 128.3 (C_q), 128.3 (CH), 126.7 (CH), 122.4 (CH), 122.3 (CH), 120.7 (CH), 118.2 (CH), 110.0 (CH), 109.4 (C_q), 63.4 (CH_2), 54.4 (CH), 53.3 (CH), 52.5 (CH_3), 38.8 (CH_2), 38.5 (CH_2), 34.3 (CH_2), 27.5 (CH_2), 23.1 (CH_3), 23.1 (CH_3), 20.7 (CH_2). **IR** (ATR): 3062, 2951, 1735, 1650, 1541, 1471, 1458, 1225, 1174, 745 cm^{-1}. **MS** (ESI) *m/z* (relative intensity) 664 (74) $[M+Na]^+$, 642 (100) $[M+H]^+$, 341 (31). **HR-MS** (ESI) *m/z* calcd for $C_{35}H_{40}N_5O_7^+$ 642.2922 $[M+H]^+$, found 642.2917.

Methyl (*N*-acetyl-1-(2-pyridyl)-2-{[methyl (acetyl-L-phenylalanyl)-L-alanine-3-yl] propionate-3-yl}-L-tryptophanate) (8c):

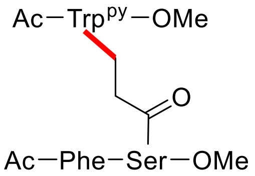

The general procedure C was followed using methyl acetyl-1-(2-pyridyl)-L-tryptophanate (**1a**, 50.6 mg, 150 µmol) and [methyl (*N*-acetyl-L-phenylalaninyl)-L-alaninate-3-yl] acrylate (60 mg, 165 µmol). Purification by column chromatography (CH_2Cl_2/MeOH 40:1→30:1→20:1) yielded **8c** (67 mg, 96 µmol, 64%) as a white solid. **m. p.** 116 °C. ^{1}**H NMR** (600 MHz, $CDCl_3$): δ 8.67–8.56 (m, 1H), 7.89 (ddd, *J* = 7.7, 7.7, 1.9 Hz, 1H), 7.53–7.47 (m, 1H), 7.43 (ddd, *J* = 8.0, 0.9, 0.9 Hz, 1H), 7.39 (d, *J* = 7.5 Hz, 1H), 7.33 (ddd, *J* = 7.4, 4.9, 0.9 Hz, 1H), 7.28–7.25 (m, 1H), 7.26–7.21 (m, 2H), 7.20–7.10 (m, 5H), 6.59 (d, *J* = 7.9 Hz, 1H), 6.49 (d, *J* = 8.0 Hz, 1H), 4.89 (ddd, *J* = 7.9, 6.5, 6.5 Hz, 1H), 4.80 (ddd, *J* = 8.0, 6.7, 6.7 Hz, 1H), 4.75 (ddd, *J* = 7.5, 3.6, 3.6 Hz, 1H), 4.35 (dd, *J* = 8.2, 3.7 Hz, 1H) 4.33 (dd, *J* = 8.2, 3.7 Hz, 1H), 3.69 (s, 3H), 3.62 (s, 3H), 3.36–3.25 (m, 2H), 3.19–3.11 (m, 3H), 2.99 (dd, *J* = 14.0, 7.1 Hz, 1H), 2.50–2.28 (m, 2H), 1.97 (s, 3H), 1.92 (s, 3H). ^{13}**C NMR** (126 MHz, $CDCl_3$): δ 172.8 (C_q), 171.6 (C_q), 171.0 (C_q), 170.1 (C_q), 170.0 (C_q), 169.2 (C_q), 150.9 (C_q), 149.6 (CH), 138.5 (CH), 136.7 (C_q), 136.5 (C_q), 136.4 (C_q), 129.2 (CH), 128.4 (C_q), 128.4 (CH), 126.7 (CH), 122.5 (CH), 122.3 (CH), 120.8 (CH), 120.8 (CH), 118.3 (CH), 110.0 (CH), 109.3 (C_q), 63.7 (CH_2), 54.0 (CH), 53.0 (CH), 52.8 (CH_3), 52.5 (CH_3), 51.8 (CH), 38.1 (CH_2), 34.3 (CH_2), 27.4 (CH_2), 23.2 (CH_3), 23.1 (CH_3), 20.6 (CH_2). **IR** (ATR): 3054, 2953, 1742, 1653, 1541, 1458, 1222, 744 cm^{-1}. **MS** (ESI) *m/z* (relative intensity) 722 (100) $[M+Na]^+$, 700 (49) $[M+H]^+$, 426 (30). **HR-MS** (ESI) *m/z* calcd for $C_{37}H_{42}N_5O_9^+$ 700.2977 $[M+H]^+$, found 700.2971.

Methyl (S)-2-(1,3-dioxoisoindolin-2-yl)-3-[2-(3-{[(S)-1-methoxy-1-oxo-3-phenylpropan-2-yl]amino}-3-oxopropyl)-1-(pyridin-2-yl)-1H-indol-3-yl]propanoate (8d):

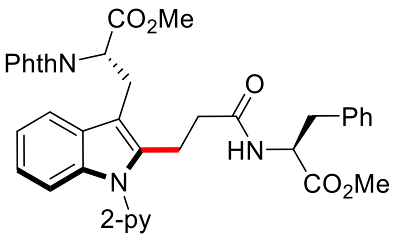

The general procedure C was followed using methyl (*S*)-2-(1,3-dioxoisoindolin-2-yl)-3-[1-(pyridin-2-yl)-1H-indol-3-yl]propanoate (64 mg, 150 µmol) and methyl acryloyl-*L*-phenylalaninate (70 mg, 300 µmol) in HOAc (450 µL). Purification by column chromatography (EtOAc) yielded **8d** (52.3 mg, 80 µmol, 53%) as a white solid. **m. p.** 178 °C. **^{1}H NMR** (400 MHz, $CDCl_3$): δ 8.55 (dd, *J* = 5.0, 1.8 Hz, 1H), 7.86 (ddd, *J* = 7.7, 7.7, 2.0 Hz, 1H), 7.76–7.68 (m, 2H), 7.69–7.62 (m, 2H), 7.61–7.55 (m, 1H), 7.35–7.30 (m, 2H), 7.26–7.18 (m, 4H), 7.10–7.02 (m, 4H), 6.29 (d, *J* = 7.8 Hz, 1H), 5.32 (dd, *J* = 9.7, 5.9 Hz, 1H), 4.84 (ddd, *J* = 7.8, 6.0, 6.0 Hz, 1H), 3.82 (s, 3H), 3.79–3.68 (m, 2H), 3.65 (s, 3H), 3.24–3.16 (m, 2H), 3.12 (dd, *J* = 13.9, 5.9 Hz, 1H), 3.05 (dd, *J* = 13.9, 6.1 Hz, 1H), 2.37–2.27 (m, 1H), 2.27–2.16 (m, 1H). **^{13}C NMR** (101 MHz, $CDCl_3$): δ 172.0 (C_q), 171.3 (C_q), 169.5 (C_q), 167.6 (C_q), 151.2 (C_q), 149.6 (CH), 138.5 (CH), 137.0 (C_q), 136.7 (C_q), 136.0 (C_q), 134.0 (CH), 131.7 (C_q), 129.2 (CH), 128.4 (CH), 128.2 (C_q), 126.9 (CH), 123.4 (CH), 122.3 (CH), 122.2 (CH), 121.0 (CH), 120.6 (CH), 118.3 (CH), 110.0 (CH), 109.7 (C_q), 53.0 (CH), 52.9 (CH_3), 52.4 (CH_3), 52.1 (CH), 37.9 (CH_2), 36.3 (CH_2), 24.5 (CH_2), 21.4 (CH_2). **IR** (ATR): 3057, 2952, 1742, 1713, 1672, 1436, 1388, 1368, 1204, 731 cm^{-1}. **MS** (ESI) *m/z* (relative intensity): 681 (100) $[M+Na]^+$, 659 (59) $[M+H]^+$. **HR-MS** (ESI) *m/z* calcd for $C_{38}H_{35}N_4O_7$ $[M+H]^+$: 659.2500; found: 659.2508.

Methyl ({[methyl *N*-acetyl-1-(2-pyridyl)-L-tryptophanate-2-yl]-3-propanoate-3-yl}methyl-carbonyl [L-phenylalaninyl-L-leucinate]) (8e):

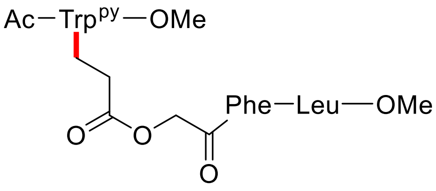

The general procedure C was followed using methyl acetyl-1-(2-pyridyl)-L-tryptophanate (**1a**, 50.6 mg, 150 µmol) and methyl [2-(acryloyloxy)acetyl]-L-phenylalanyl-L-leucinate (67 mg, 165 µmol). Purification by column chromatography (CH_2Cl_2/MeOH 40:1→20:1) yielded **8e** (68 mg, 92 µmol, 61%) as a white solid. **m. p.** 179 °C. **^{1}H NMR** (600 MHz, $CDCl_3$): δ 8.65–8.62 (m, 1H), 7.91 (ddd, *J* = 7.7, 7.7, 1.7 Hz, 1H), 7.54–7.49 (m, 1H), 7.46 (d, *J* = 7.9 Hz, 1H), 7.34 (dd, *J* = 7.4, 4.9 Hz, 1H), 7.28–7.25 (m, 1H), 7.25–7.20 (m, 2H), 7.19–7.11 (m, 5H), 7.08 (d, *J* = 7.6 Hz, 1H), 6.41 (d, *J* = 7.8 Hz, 1H), 6.31 (d, *J* = 7.8 Hz, 1H), 4.91 (ddd, *J* = 7.6, 7.6, 7.6 Hz, 1H), 4.63 (ddd, *J* = 7.8, 7.8, 7.8 Hz, 1H), 4.51–4.37 (m, 3H), 3.67 (s, 3H), 3.63 (s, 3H), 3.33 (dd, *J* = 14.7, 6.9 Hz, 1H), 3.28 (dd, *J* = 14.7, 5.6 Hz, 1H),

3.25–3.13 (m, 2H), 3.09–2.97 (m, 2H), 2.54–2.40 (m, 2H), 1.94 (s, 3H), 1.60–1.39 (m, 3H), 0.85 (d, *J* = 6.3 Hz, 6H). **^{13}C NMR** (126 MHz, $CDCl_3$): δ 172.5 (C_q), 172.4 (C_q), 170.9 (C_q), 170.0 (C_q), 169.9 (C_q), 166.8 (C_q), 150.9 (C_q), 149.7 (CH), 138.6 (CH), 136.6 (C_q), 136.3 (C_q), 136.3 (C_q), 129.2 (CH), 128.5 (C_q), 128.5 (CH), 126.9 (CH), 122.5 (CH), 122.4 (CH), 120.8 (CH), 120.8 (CH), 118.4 (CH), 110.1 (CH), 109.6 (C_q), 62.8 (CH_2), 54.2 (CH), 53.0 (CH), 52.4 (CH_3), 52.2 (CH_3), 51.0 (CH), 41.3 (CH_2), 37.7 (CH_2), 33.8 (CH_2), 27.5 (CH_2), 24.8 (CH), 23.2 (CH_3), 22.7 (CH_3), 22.0 (CH_3), 20.4 (CH_2). **IR** (ATR): 2956, 1743, 1654, 1541, 1472, 1478, 1159, 745 cm^{-1}. **MS** (ESI) *m/z* (relative intensity) 764 (100) $[M+Na]^+$, 742 (78) $[M+H]^+$. **HR-MS** (ESI) *m/z* calcd for $C_{40}H_{48}N_5O_9^+$ 742.3447 $[M+H]^+$, found 742.3437.

Methyl {[methyl ({[*N*-acetyl-1-(2-pyridyl)-L-tryptophyl-2-yl]-3-propanoate-3-yl}methyl-carbonyl [L-phenylalaninyl-L-leucinate])]-L-alaninyl-L-phenylalaninate} (8f):

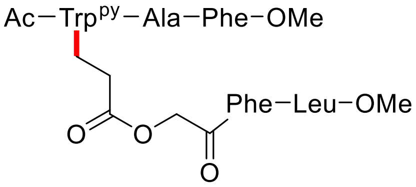

The general procedure C was followed using methyl *N*-acetyl-1-(2-pyridyl)-L-tryptophyl-L-alaninyl-L-phenylalaninate (84.6 mg, 150 µmol) and methyl (2-(acryloyloxy)acetyl)-L-phenylalanyl-L-leucinate (67 mg, 165 µmol) in 300 µL HOAc. Purification by column chromatography (CH_2Cl_2/MeOH 40:1→30:1→20:1) yielded **8f** (75 mg, 78 µmol, 52%) as a white solid. **m. p.** 153 °C. **^{1}H NMR** (600 MHz, $CDCl_3$): δ 8.60 (dd, *J* = 5.0, 1.9 Hz, 1H), 7.88 (ddd, *J* = 7.7, 7.7, 2.0 Hz, 1H), 7.64–7.61 (m, 1H), 7.54–7.49 (m, 1H), 7.35–7.29 (m, 2H), 7.27–7.23 (m, 3H), 7.23–7.16 (m, 7H), 7.12–7.08 (m, 2H), 7.04–6.97 (m, 2H), 6.73 (d, *J* = 7.3 Hz, 1H), 6.66-6.61 (m, 2H), 4.79 (ddd, *J* = 8.0, 8.0, 5.3 Hz, 1H), 4.63 (ddd, *J* = 7.4, 7.4, 7.4 Hz, 1H), 4.60 (ddd, *J* = 7.7, 7.7 Hz, 1H), 4.52–4.43 (m, 2H), 4.41–4.32 (m, 2H), 3.66 (s, 3H), 3.61 (s, 3H), 3.34–3.10 (m, 4H), 3.06 (d, *J* = 7.2 Hz, 2H), 2.94 (d, *J* = 6.2 Hz, 2H), 2.43 (t, *J* = 7.8 Hz, 2H), 1.93 (s, 3H), 1.59–1.48 (m, 2H), 1.45 (ddd, *J* = 12.5, 8.8, 5.1 Hz, 1H), 1.21 (d, *J* = 7.0 Hz, 3H), 0.85 (d, *J* = 6.4 Hz, 6H). **^{13}C NMR** (126 MHz, $CDCl_3$): δ 172.5 (C_q), 171.5 (C_q), 171.2 (C_q), 171.1 (C_q), 170.8 (C_q), 170.3 (C_q), 169.9 (C_q), 167.0 (C_q), 151.1 (C_q), 149.6 (CH), 138.4 (CH), 136.7 (C_q), 136.5 (C_q), 136.3 (C_q), 135.7 (C_q), 129.2 (CH), 129.0 (CH), 128.5 (CH), 128.5 (CH), 128.4 (C_q), 127.1 (CH), 126.9 (CH), 122.5 (CH), 122.2 (CH), 121.1 (CH), 120.8 (CH), 118.6 (CH), 110.1 (CH), 109.8 (C_q), 63.0 (CH_2), 54.5 (CH), 53.8 (CH), 53.4 (CH), 52.3 (CH_3), 52.2 (CH_3), 51.1 (CH), 48.8 (CH), 41.2 (CH_2), 37.8 (CH_2), 37.8 (CH_2), 33.9 (CH_2), 27.8 (CH_2), 24.8 (CH_3), 23.3 (CH_3), 22.7 (CH_3), 22.0 (CH), 20.4 (CH_2), 18.8 (CH_3). **IR** (ATR): 2921, 1745, 1652, 1541, 1473, 1457, 1158, 745 cm^{-1}. **MS** (ESI) *m/z* (relative intensity) 982 (100) $[M+Na]^+$, 961 (14) $[M+H]^+$. **HR-MS** (ESI) *m/z* calcd for $C_{52}H_{62}N_7O_{11}^+$ 961.4502 $[M+H]^+$, found 961.4502.

{*N*-acetyl [ethyl-1-(2-pyridyl)-2-{[*N*-acetyl (L-phenylalanineamide-4-yl) propionate-3-yl]}-L-tryptophyl-L-tryptophyl-glycinate]}[N^{ω}-(*O*-ethyl)acetate-2-yl] (8g):

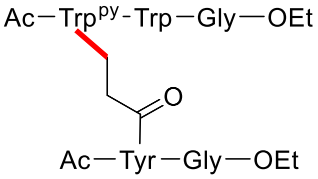

The general procedure C was followed using ethyl acetyl-1-(2-pyridyl)-L-tryptophyl-L-tryptophyl-glycinate (10.0 mg, 16.8 µmol) and ethyl acetyl-L-tyrosylglycinate acrylate (6.7 mg, 18.5 µmol). Purification by PTLC (EtOAc/MeOH 7:1) yielded peptide **8g** (9.2 mg, 9.6 µmol, 57%) as a white solid. **m. p.**: 93 °C. **^{1}H NMR** (500 MHz, CD_3OD): δ 8.65 (ddd, *J* = 5.0, 2.0, 0.8 Hz, 1H), 8.08 (ddd, *J* = 7.7, 7.7, 2.0 Hz, 1H), 7.67–7.58 (m, 2H), 7.52–7.46 (m, 2H), 7.28 (ddd, *J* = 8.1, 0.9, 0.9 Hz, 1H), 7.20 (dm, *J* = 8.6 Hz, 3H), 7.14–7.07 (m, 2H), 7.08–7.01 (m, 2H), 6.94 (ddd, *J* = 8.0, 7.0, 1.0 Hz, 1H), 6.82 (dm, *J* = 8.6 Hz, 2H), 4.69 (dd, *J* = 7.2, 7.2 Hz, 1H), 4.65–4.58 (m, 2H), 4.15 (ddd, *J* = 7.1, 7.1, 7.1 Hz, 2H), 4.10 (ddd, *J* = 7.1, 7.1, 7.1 Hz, 2H), 3.90–3.87 (m, 3H), 3.76–3.66 (m, 2H), 3.28–3.21 (m, 4H), 3.17–3.08 (m, 2H), 3.08–3.01 (m, 2H), 2.84 (dd, *J* = 14.1, 9.2 Hz, 1H), 2.61–2.53 (m, 1H), 2.53–2.44 (m, 1H), 1.87 (s, 3H), 1.78 (s, 3H), 1.24 (dd, *J* = 7.1, 7.1 Hz, 3H), 1.20 (dd, *J* = 7.1, 7.1 Hz, 3H). **^{13}C NMR** (126 MHz, CD_3OD): δ 175.1 (C_q), 174.8 (C_q), 174.4 (C_q), 174.3 (C_q), 174.2 (C_q), 173.8 (C_q), 172.0 (C_q), 171.9 (C_q), 153.5 (C_q), 151.8 (C_q), 151.7 (CH), 141.8 (CH), 139.5 (C_q), 138.9 (C_q), 138.6 (C_q), 137.3 (C_q), 132.2 (CH), 130.7 (C_q), 129.9 (C_q), 125.9 (CH), 125.3 (CH), 124.6 (CH), 124.3 (CH), 123.5 (CH), 123.3 (CH), 122.8 (CH), 120.8 (CH), 120.3 (CH), 117.2 (CH), 113.3 (CH), 112.5 (C_q), 111.9 (CH), 111.6 (C_q), 63.3 (CH_2), 63.3 (CH_2), 56.8 (CH), 56.6 (CH), 56.4 (CH), 43.1 (CH_2), 43.1 (CH_2), 39.1 (CH_2), 35.5 (CH_2), 29.7 (CH_2), 29.0 (CH_2), 23.5 (CH_3), 23.4 (CH_3), 22.3 (CH_2), 15.5 (CH_3), 15.5 (CH_3). **IR** (ATR): 3292, 1730, 1669, 1653, 1568, 1472, 1465, 1437, 1372, 1197, 744 cm^{-1}. **MS** (ESI) *m/z* (relative intensity) 979 (100) $[M+Na]^+$, 957 (2) $[M+H]^+$, 703 (14). **HR-MS** (ESI) *m/z* calcd for $C_{51}H_{56}N_8NaO_{11}^+$ 979.3961 $[M+Na]^+$, found 979.3960.

Butane-1,4-diyl bis[methyl acetyl-1-(2-pyridyl)-2-(propionate-3-yl)-L-tryptophanate] (8h):

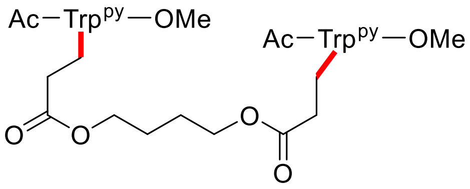

The general procedure A was followed using methyl acetyl-1-(2-pyridyl)-L-tryptophanate (**1a**, 50.6 mg, 150 µmol) and butane-1,4-diyl diacrylate (15 mg, 75 µmol). Purification by column chromatography (EtOAc) yielded **8h** (35 mg, 41 µmol, 54%) as a white solid. **m. p.** 86 °C (decomp.). **^{1}H NMR** (500 MHz, $CDCl_3$): δ 8.62 (ddd, *J* = 4.9, 2.0, 0.8 Hz, 2H), 7.91 (ddd, *J* = 8.0, 7.5, 2.0 Hz, 2H), 7.56–7.53 (m, 2H), 7.45 (ddd, *J* = 8.0, 1.0, 1.0 Hz, 2H), 7.33 (ddd, *J* = 7.4, 4.9, 1.0 Hz, 2H), 7.29–7.27 (m, 2H), 7.16–7.13 (m, 4H), 6.31 (d, *J* = 7.8 Hz, 2H), 4.95 (ddd, *J* = 7.8, 6.0, 6.0 Hz, 2H), 3.96–3.89 (m,

4H), 3.69 (s, 6H), 3.40–3.27 (m, 4H), 3.25–3.12 (m, 4H), 2.31 (ddd, *J* = 8.5, 7.0, 3.1 Hz, 4H), 1.94 (s, 6H), 1.61–1.66 (m, 4H), 1.46–1.51 (m, 4H). **^{13}C NMR** (126 MHz, $CDCl_3$): δ 172.5 (C_q), 172.3 (C_q), 169.8 (C_q), 162.5 (C_q), 151.1 (C_q), 149.6 (CH), 138.5 (CH), 136.7 (C_q), 128.5 (C_q), 122.4 (CH), 122.3 (CH), 120.9 (CH), 120.7 (CH), 118.4 (CH), 109.9 (CH), 109.3 (C_q), 63.9 (CH_2), 52.8 (CH), 52.4 (CH_3), 33.7 (CH_2), 27.1 (CH_2), 25.1 (CH_2), 23.1 (CH_3), 20.4 (CH_2). **IR** (ATR): 2953, 2924, 1731, 1653, 1470, 1459, 1436, 1369, 1167, 740 cm^{-1}. **MS** (ESI) *m/z* (relative intensity) 895 (100) $[M+Na]^+$, 873 (71) $[M+H]^+$, 456 (80). **HR-MS** (ESI) *m/z* calcd for $C_{48}H_{53}N_6O_{10}^+$ 873.3818 $[M+H]^+$, found 873.3799.

Methyl *N*-acetyl-1-(2-pyridyl)-2-{[(*N*-acetylphenylalaninyl)-2-aminoethane-1-yl] propionate-3-yl}-L-tryptophyl-L-alaninyl-L-phenylalaninate (8i):

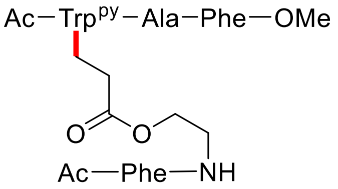

The general procedure C was followed using methyl *N*-acetyl-1-(2-pyridyl)-L-tryptophyl-L-alaninyl-L-phenylalaninate (84.6 mg, 150 µmol) and *N*-acetyl-L-phenylalaninyl-2-aminoethane-1-yl acrylate (46 mg, 165 µmol). Purification by column chromatography (CH_2Cl_2/MeOH 40:1→20:1) yielded **8i** (67 mg, 78 µmol, 52%) as a white solid. **m. p.** 192 °C (decomp.). **^{1}H NMR** (600 MHz, $CDCl_3$): δ 8.59 (dd, *J* = 4.8, 1.6 Hz, 1H), 7.85 (ddd, *J* = 7.7, 7.7, 2.0 Hz, 1H), 7.70–7.60 (m, 1H), 7.49 (dd, *J* = 8.0, 1.0 Hz, 1H), 7.39 (dd, *J* = 6.0, 6.0 Hz, 1H), 7.30 (ddd, *J* = 7.5, 4.8, 1.0 Hz, 1H), 7.26–7.23 (m, 2H), 7.22–7.19 (m, 4H), 7.18–7.16 (m, 1H), 7.15–7.10 (m, 5H), 7.04–7.01 (m, 2H), 6.92 (d, *J* = 7.3 Hz, 1H), 6.83–6.77 (m, 2H), 4.80–4.68 (m, 2H), 4.57 (ddd, *J* = 7.7, 6.4, 6.4 Hz, 1H), 4.50 (dq, *J* = 7.0, 7.0 Hz, 1H), 4.05 (ddd, *J* = 11.4, 6.8, 2.9 Hz, 1H), 3.95 (ddd, *J* = 11.4, 6.6, 2.9 Hz, 1H), 3.61 (s, 3H), 3.48–3.39 (m, 1H), 3.37–3.31 (m, 1H), 3.27 (dd, *J* = 14.5, 6.3 Hz, 1H), 3.16–3.00 (m, 4H), 2.99–2.92 (m, 3H), 2.45–2.24 (m, 2H), 1.96 (s, 3H), 1.88 (s, 3H), 1.23 (d, *J* = 7.0 Hz, 3H). **^{13}C NMR** (126 MHz, $CDCl_3$): δ 172.0 (C_q), 171.6 (C_q), 171.5 (C_q), 171.3 (C_q), 171.0 (C_q), 170.1 (C_q), 170.1 (C_q), 151.1 (C_q), 149.6 (CH), 138.4 (CH), 136.8 (C_q), 136.6 (C_q), 136.6 (C_q), 136.5 (C_q), 135.8 (C_q), 129.2 (CH), 129.0 (CH), 128.4 (CH), 128.3 (CH), 127.0 (CH), 126.7 (CH), 122.5 (CH), 122.2 (CH), 121.1 (CH), 120.9 (CH), 118.4 (CH), 110.1 (CH), 109.6 (C_q), 63.5 (CH_2), 54.5 (CH), 54.4 (CH), 53.6 (CH), 52.2 (CH_3), 48.8 (CH), 38.7 (CH_2), 38.5 (CH_2), 37.8 (CH_2), 34.7 (CH_2), 28.0 (CH_2), 23.3 (CH_3), 23.1 (CH_3), 20.7 (CH_2), 19.1 (CH_3). **IR** (ATR): 2957, 2921, 2851, 1734, 1647, 1541, 1457, 1438, 744 cm^{-1}. **MS** (ESI) *m/z* (relative intensity) 882 (100) $[M+Na]^+$, 860 (46) $[M+H]^+$. **HR-MS** (ESI) *m/z* calcd for $C_{47}H_{54}N_7O_9^+$ 860.3978 $[M+H]^+$, found 860.3968.

Methyl *N*-acetyl-1-(2-pyridyl)-2-{2-[2-(4-isobutylphenyl)propanamido]ethyl propionate-3-yl}-L-tryptophyl-L-alaninyl-L-phenylalaninate (8j):

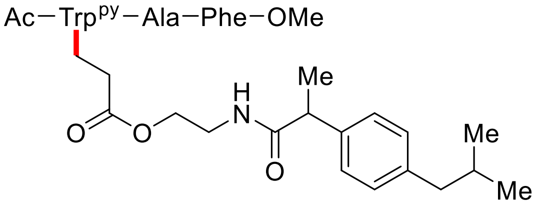

The general procedure B was followed using methyl *N*-acetyl-1-(2-pyridyl)-L-tryptophyl-L-alaninyl-L-phenylalaninate (84.6 mg, 150 µmol) and 2-[2-(4-isobutylphenyl)propanamido]ethyl acrylate (69 mg, 0.23 mmol). Purification by column chromatography (CH_2Cl_2/MeOH 40:1→20:1) yielded **8j** (82 mg, 95 µmol, 63%) as a white solid. **m. p.** 135 °C. **^{1}H NMR** (600 MHz, $CDCl_3$, mixture of diastereomers): δ 8.60 (ddd, *J* = 4.6, 2.1, 2.1 Hz, 1H), 7.95–7.82 (m, 1H), 7.67–7.60 (m, 1H), 7.50 (dd, *J* = 8.0, 3.4 Hz, 1H), 7.33 (ddd, *J* = 5.1, 5.1, 2.4 Hz, 1H), 7.26 (s, 3H), 7.25–7.18 (m, 1H), 7.19–7.14 (m, 2H), 7.12–7.07 (m, 4H), 7.05 (d, *J* = 6.6 Hz, 2H), 6.88–6.67 (m, 3H), 6.53–6.47 (m, 1H), 4.76 (ddd, *J* = 7.2,7.2, 7.2 Hz, 1H), 4.67 (dq, *J* = 6.7, 6.7 Hz, 1H), 4.42–4.32 (m, 1H), 4.12–3.92 (m, 2H), 3.65 (s, 3H), 3.54–3.35 (m, 2H), 3.31–3.08 (m, 5H), 3.02 (dd, *J* = 6.3, 4.7 Hz, 2H), 2.42–2.27 (m, 4H), 1.94-1.91 (m, 3H), 1.83–1.74 (m, 1H), 1.44 (d, *J* = 7.1 Hz, 1.5H), 1.41 (d, *J* = 7.1 Hz, 1.5H), 1.25 (t, *J* = 6.9 Hz, 3H), 0.86 (d, *J* = 6.6 Hz, 6H). **^{13}C NMR** (126 MHz, $CDCl_3$, mixture of diastereomers): δ 174.6 (C_q), 172.5 (C_q), 172.4 (C_q), 171.6 (C_q), 171.0 (C_q), 171.0 (C_q), 170.9 (C_q), 170.0 (C_q), 151.1 (C_q), 151.1 (C_q), 149.5 (CH), 140.3 (C_q), 140.2 (C_q), 138.6 (C_q), 138.6 (C_q), 138.5 (C_q), 136.7 (C_q), 136.7 (C_q), 136.5 (C_q), 136.4 (C_q), 135.7 (C_q), 129.3 (CH), 129.2 (CH), 129.1 (CH), 128.5 (CH), 128.3 (C_q), 128.3 (C_q), 127.1 (CH), 127.1 (CH), 122.4 (CH), 122.3 (CH), 122.3 (CH), 121.2 (CH), 121.1 (CH), 120.7 (CH), 118.5 (CH), 118.4 (CH), 110.0 (CH), 109.9 (C_q), 63.4 (CH_2), 54.1 (CH), 53.5 (CH), 52.2 (CH_3), 49.0 (CH), 46.3 (CH), 46.3 (CH), 45.0 (CH_2), 38.5 (CH_2), 37.9 (CH_2), 33.9 (CH_2), 33.8 (CH_2), 30.2 (CH_3), 28.0 (CH_2), 27.9 (CH_2), 23.2 (CH), 22.4 (CH_3), 20.5 (CH_2), 20.5 (CH_2), 18.7 (CH_3), 18.7 (CH_3), 18.6 (CH_3). **IR** (ATR): 2950, 2923, 1734, 1683, 1647, 1637, 1541, 1523, 1508 cm^{-1}. **MS** (ESI) *m/z* (relative intensity) 882 (100) $[M+Na]^+$, 859 (35) $[M+H]^+$. **HR-MS** (ESI) *m/z* calcd for $C_{49}H_{59}N_6O_8^+$ 859.4389 $[M+H]^+$, found 859.4385.

Methyl (*N*-acetyl-1-(2-pyridyl)-2-{[methyl (acetyl-L-phenylalanyl)-L-alanine-3-yl] propionate-3-yl}-L-tryptophyl-L-isoleucinate) (8k):

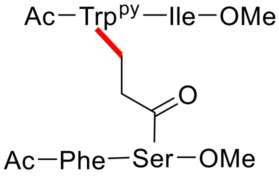

The general procedure C was followed using methyl *N*-acetyl-1-(2-pyridyl)-L-tryptophyl-L-isoleucinate (67.5 mg, 150 µmol) and [methyl (*N*-acetyl-L-phenylalaninyl)-L-alaninate-3-yl] acrylate (60 mg, 165 µmol) in 300 µL HOAc. Purification by column chromatography (CH_2Cl_2/MeOH 40:1→30:1→20:1) yielded **8k** (68 mg, 84 µmol, 56%) as a white solid. **m. p.** 135 °C. **^{1}H NMR** (600 MHz, $CDCl_3$): δ 8.63 (ddd, *J* = 4.9, 2.0, 0.8 Hz, 1H), 7.89 (ddd, *J* = 7.7, 7.7, 1.9 Hz, 1H), 7.82 (d,

J = 8.0 Hz, 1H), 7.58–7.55 (m, 1H), 7.49 (dd, *J* = 8.0, 1.0 Hz, 1H), 7.32 (ddd, *J* = 7.7, 4.8, 1.0 Hz, 1H), 7.30–7.27 (m, 1H), 7.25–7.17 (m, 3H), 7.17–7.10 (m, 4H), 6.88 (d, *J* = 8.0 Hz, 1H), 6.61–6.54 (m, 2H), 4.90 (ddd, *J* = 6.9, 6.9, 6.9 Hz, 1H), 4.88–4.79 (m, 2H), 4.42 (dd, *J* = 11.3, 4.4 Hz, 1H), 4.38 (dd, *J* = 8.4, 5.0 Hz, 1H), 4.34 (dd, *J* = 11.3, 3.2 Hz, 1H), 3.74 (s, 3H), 3.44 (s, 3H), 3.30–3.09 (m, 5H), 3.00 (dd, *J* = 14.0, 6.8 Hz, 1H), 2.48 (ddd, *J* = 15.6, 9.2, 6.0 Hz, 1H), 2.37 (ddd, *J* = 15.6, 9.1, 6.5 Hz, 1H), 1.97 (s, 3H), 1.94 (s, 3H), 1.77–1.69 (m, 1H), 1.37–1.24 (m, 1H), 1.05 (ddq, *J* = 14.4, 9.3, 7.4 Hz, 1H), 0.82 (t, *J* = 7.4 Hz, 3H), 0.77 (d, *J* = 6.8 Hz, 3H). **^{13}C NMR** (126 MHz, $CDCl_3$): δ 171.9 (C_q), 171.2 (C_q), 171.1 (C_q), 171.0 (C_q), 169.9 (C_q), 169.8 (C_q), 169.6 (C_q), 151.1 (C_q), 149.6 (CH), 138.4 (CH), 136.6 (C_q), 136.6 (C_q), 136.4 (C_q), 129.2 (CH), 128.5 (C_q), 128.3 (CH), 126.7 (CH), 122.3 (CH), 122.1 (CH), 120.9 (CH), 120.8 (CH), 118.2 (CH), 110.0 (CH), 109.7 (C_q), 63.7 (CH_2), 56.6 (CH), 53.9 (CH), 53.8 (CH), 52.8 (CH_3), 51.9 (CH), 51.8 (CH_3), 38.3 (CH), 38.2 (CH_2), 34.3 (CH_2), 28.0 (CH_2), 25.2 (CH_2), 23.3 (CH_3), 23.1 (CH_3), 20.6 (CH_2), 15.2 (CH_3), 11.6 (CH_3). **IR** (ATR): 3050, 2949, 2929, 1744, 1656, 1584, 1458, 1335, 1120, 736 cm^{-1}. **MS** (ESI) *m/z* (relative intensity) 835 (100) $[M+Na]^+$, 813 (29) $[M+H]^+$, 426 (30). **HR-MS** (ESI) *m/z* calcd for $C_{43}H_{53}N_6O_{10}^+$ 813.3818 $[M+H]^+$, found 813.3816.

Benzyl (acetyl-L-serinyl)-L-leucyl-L-alanyl-1-(2-pyridyl)-2-[(methyl (acetyl-L-phenylalanyl)-L-alaninate-3-yl) propionate-3-yl]-β^3-*homo*-L-tryptophanate (8l):

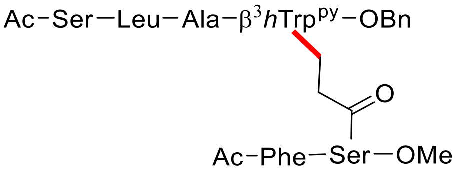

The general procedure C was followed using benzyl acetyl-L-serinyl-L-leucyl-L-alanyl-1-(2-pyridyl)-β^3-*homo*-L-tryptophanate (10.8 mg, 15.5 µmol) and methyl acetyl-L-phenylalanyl-L-serinate acrylate (11.2 mg, 30.9 µmol) in 50 µL HOAc. Purification by PTLC (first PTLC with EtOAc/MeOH 8:1, followed by a second PTLC with EtOAc/MeOH 10:1) yielded peptide **8l** (6.9 mg, 6.5 µmol, 42%) as a white solid. **m. p.**: 142 °C. **^{1}H NMR** (600 MHz, $CDCl_3$): δ 8.65 (ddd, *J* = 4.9, 2.0, 0.8 Hz, 1H), 8.15 (d, *J* = 8.0 Hz, 1H), 7.91 (ddd, *J* = 7.7, 7.7, 2.0 Hz, 1H), 7.60 (d, *J* = 8.6 Hz, 1H), 7.56 (dd, *J* = 7.2, 1.6 Hz, 1H), 7.46–7.40 (m, 2H), 7.34 (ddd, *J* = 7.5, 4.9, 1.0 Hz, 1H), 7.31 (d, *J* = 3.0 Hz, 5H), 7.28–7.21 (m, 2H), 7.21–7.14 (m, 4H), 7.14–7.07 (m, 2H), 6.90 (s, 1H), 6.81 (d, *J* = 7.7 Hz, 1H), 6.49 (d, *J* = 8.6 Hz, 1H), 5.12–5.06 (m, 2H), 5.04 (ddd, *J* = 8.3, 8.3, 6.0 Hz, 1H), 4.84 (ddd, *J* = 8.7, 4.7, 4.7 Hz, 1H), 4.71–4.65 (m, 1H), 4.56 (dd, *J* = 7.3, 7.3 Hz, 2H), 4.47 (dd, *J* = 11.5, 3.8 Hz, 1H), 4.40–4.34 (m, 1H), 4.26 (dd, *J* = 11.4, 5.4 Hz, 1H), 3.71–3.68 (m, 1H), 3.69 (s, 3H), 3.27–3.22 (m, 1H), 3.22–3.15 (m, 2H), 3.15–3.07 (m, 2H), 3.07–2.98 (m, 2H), 2.93 (dd, *J* = 14.0, 7.8 Hz, 1H), 2.66 (dd, *J* = 16.2, 5.8 Hz, 1H), 2.62–2.57 (dd, *J* = 16.2, 5.8 Hz, 1H), 2.35–2.24 (m, 2H), 1.98 (s, 3H), 1.83 (s, 3H), 1.69–1.64 (m, 1H), 1.22 (d, *J* = 7.0 Hz, 3H), 0.90 (d, *J* = 5.7 Hz, 3H), 0.88 (d, *J* = 5.7 Hz, 3H). **^{13}C NMR** (126 MHz, $CDCl_3$): δ 172.6 (C_q), 172.2 (C_q), 172.2 (C_q), 171.6 (C_q), 171.2 (C_q), 170.6 (C_q), 170.3 (C_q), 170.2 (C_q), 169.4 (C_q), 151.2 (C_q), 149.6 (CH), 138.7 (CH), 136.6 (C_q), 136.4 (C_q), 136.4 (C_q), 136.3 (C_q), 135.5

(C_q), 129.2 (CH), 128.6 (CH), 128.5 (CH), 128.4 (CH), 128.3 (CH), 126.7 (CH), 122.4 (CH), 122.3 (CH), 121.0 (CH), 120.8 (CH), 118.7 (CH), 110.7 (C_q), 109.9 (CH), 68.7 (CH_2), 66.6 (CH_2), 63.8 (CH_2), 54.9 (CH), 54.8 (CH), 53.9 (CH), 53.7 (CH), 52.7 (CH), 51.5 (CH_3), 49.0 (CH), 40.4 (CH_2), 38.9 (CH_2), 38.3 (CH_2), 34.0 (CH_2), 29.2 (CH_2), 25.0 (CH), 23.2 (CH_3), 23.2 (CH_3), 23.1 (CH_3), 21.4 (CH_3), 20.7 (CH_2), 18.9 (CH_3). **IR** (ATR): 3290, 2938, 1733, 1645, 1531, 1471, 1457, 1437, 1124, 742 cm^{-1}. **MS** (ESI) *m/z* (relative intensity) 1083 (100) $[M+Na]^+$, 1061 (26) $[M+H]^+$, 550 (60). **HR-MS** (ESI) *m/z* calcd for $C_{56}H_{69}N_8O_{13}^+$ 1061.4979 $[M+H]^+$, found 1061.4963.

(*S*)-Methyl 2-acetamido-3-[2-(3-ethoxy-3-oxopropyl)-1H-indol-3-yl]propanoate (9a):

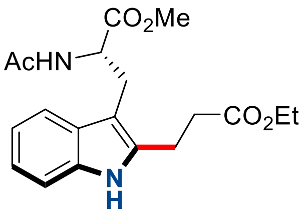

The general procedure D was followed using methyl (*S*)-2-acetamido-3-[2-(3-ethoxy-3-oxopropyl)-1-(pyridin-2-yl)-1*H*-indol-3-yl]propanoate **3a** (87.4 mg, 200 µmol). Purification by column chromatography on silica gel (EtOAc) yielded **9a** (46.8 mg, 130 µmol, 65%) as a colorless oil. **^{1}H NMR** (400 MHz, $CDCl_3$): δ 8.82 (s, 1H), 7.42 (dd, *J* = 7.5, 1.1 Hz, 1H), 7.28–7.22 (m, 1H), 7.10 (ddd, *J* = 8.1, 7.1, 1.3 Hz, 1H), 7.04 (ddd, *J* = 8.1, 7.1, 1.0 Hz, 1H), 6.18 (d, *J* = 7.8 Hz, 1H), 4.86 (ddd, *J* = 7.8, 5.7, 5.7 Hz, 1H), 4.15 (q, *J* = 7.1 Hz, 2H), 3.64 (s, 3H), 3.30–3.24 (m, 1H), 3.24–3.18 (m, 1H), 3.00–2.91 (m, 2H), 2.68–2.60 (m, 2H), 1.91 (s, 3H), 1.23 (t, *J* = 7.1 Hz, 3H). **^{13}C NMR** (101 MHz, $CDCl_3$) δ 174.1 (C_q), 172.6 (C_q), 169.8 (C_q), 135.8 (C_q), 135.3 (C_q), 128.4 (C_q), 121.5 (CH), 119.4 (CH), 118.0 (CH), 110.7 (CH), 105.6 (C_q), 61.0 (CH_2), 53.1 (CH), 52.3 (CH_3), 34.0 (CH_2), 26.7 (CH_2), 23.1 (CH_3), 20.3 (CH_2), 14.1 (CH_3). **IR** (ATR): 3333, 2969, 1734, 1655, 1528, 1438, 1374, 1202, 745 cm^{-1}. **MS** (ESI) *m/z* (relative intensity): 743 (17) $[2M+Na]^+$, 383 (100) $[M+Na]^+$, 361 (28) $[M+H]^+$. **HR-MS** (ESI) *m/z* calcd for $C_{19}H_{25}N_2O_5$ $[M+H]^+$: 361.1758; found: 361.1755.

Ethyl 3-{3-[(1,4-dioxooctahydropyrrolo[1,2-a]pyrazin-3-yl)methyl]-1*H*-indol-2-yl}propanoate (9b):

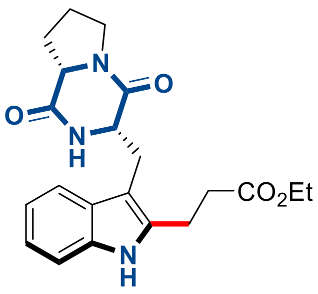

The general procedure D was followed using ethyl 3-{3-[(1,4-dioxooctahydropyrrolo[1,2-a]pyrazin-3-yl)methyl]-1-[pyridin-2-yl]-1*H*-indol-2-yl}propanoate **6e** (92.1 mg, 200 µmol). Purification by column chromatography on silica gel (EtOAc) yielded **9b** (48.9 mg, 128 µmol, 64%) as a pale yellow solid. **m. p.** 92 °C. **^{1}H NMR** (400 MHz, $CDCl_3$): δ 8.86 (s, 1H), 7.48–7.41 (m, 1H), 7.29 (dd, *J* = 8.1, 0.9 Hz, 1H), 7.14 (ddd, *J* = 8.0, 7.1, 0.9 Hz, 1H), 7.06 (ddd, *J* = 8.0, 7.1, 0.9 Hz, 1H), 5.73 (s, 1H), 4.36 (ddd, *J* = 11.3, 4.3, 1.5 Hz, 1H), 4.14 (q, *J* = 7.1 Hz, 2H), 4.03 (ddd, *J* = 8.0, 7.2, 1.6 Hz, 1H), 3.74 – 3.48 (m, 3H), 3.09–2.88 (m, 3H), 2.77–2.55 (m, 2H), 2.38–2.23 (m, 1H), 2.07–1.94 (m, 2H), 1.93–1.79 (m, 1H), 1.22 (t, *J* = 7.1 Hz, 3H). **^{13}C NMR** (101 MHz, $CDCl_3$): δ 173.8 (C_q), 169.4 (C_q), 165.7 (C_q), 136.2 (C_q), 135.6 (C_q), 127.4 (C_q), 122.1 (CH), 119.8 (CH), 117.8 (CH), 111.0 (CH), 105.2 (C_q), 61.1 (CH_2), 59.2 (CH), 54.6 (CH), 45.4 (CH_2), 34.0 (CH_2), 28.3 (CH_2), 25.6 (CH_2), 22.6 (CH_2), 20.5 (CH_2), 14.1 (CH_3). **IR** (ATR): 3282, 2979, 2925, 1726, 1655, 1460, 1419, 1188, 733 cm^{-1}. **MS** (ESI) *m/z* (relative intensity): 789 (46) $[2M+Na]^+$, 406 (100) $[M+Na]^+$, 384 (23) $[M+H]^+$. **HR-MS** (ESI) *m/z* calcd for $C_{21}H_{26}N_3O_4$ $[M+H]^+$: 384.1918; found: 384.1916.

(*S*)-Methyl 2-acetamido-3-[2-(3-{2-[(*S*)-2-acetamido-3-phenylpropanamido]ethoxy}-3-oxopropyl)-1*H*-indol-3-yl]propanoate (9c):

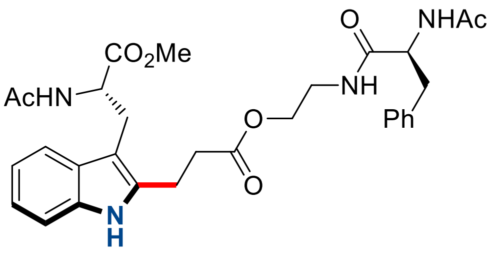

The general procedure D was followed using (s)-methyl 2-acetamido-3-[2-(3-{2-[(S)-2-acetamido-3-phenylpropanamido]ethoxy}-3-oxopropyl)-1-(pyridin-2-yl)-1H-indol-3-yl]propanoate **8b** (128.3 mg, 200 µmol). Purification by column chromatography on silica gel (CH_2Cl_2/MeOH 40:1→20:1) yielded **9c** (59.8 mg, 106 µmol, 53%) as a pale yellow solid. **m. p.** 107 °C. **^{1}H NMR** (400 MHz, $CDCl_3$) δ 9.19 (s, 1H), 7.45 (d, *J* = 7.6 Hz, 1H), 7.29–7.03 (m, 8H), 6.95–6.86 (m, 1H), 6.66 (d, *J* = 7.2 Hz, 1H), 6.51 (d, *J* = 7.7 Hz, 1H), 4.86 (ddd, *J* = 6.7, 6.7, 6.7 Hz, 1H), 4.74–4.64 (m, 1H), 4.21–3.94 (m, 2H), 3.67 (s, 3H), 3.45–3.35 (s, 2H), 3.33–3.17 (m, 2H), 3.10–2.95 (m, 4H), 2.74–2.60 (m, 2H), 1.95–1.90 (m, 6H). **^{13}C NMR** (126 MHz, $CDCl_3$) δ 173.3 (C_q), 172.8 (C_q), 171.6 (C_q), 170.5 (C_q), 170.1 (C_q), 136.5 (C_q), 135.5 (C_q), 135.4 (C_q), 129.1 (CH), 128.5 (CH), 128.4 (C_q), 126.9 (CH), 121.5 (CH), 119.4 (CH), 117.9

(CH), 110.7 (CH), 105.7 (C_q), 63.3 (CH_2), 54.5 (CH), 53.2 (CH), 52.4 (CH_3), 38.4 (CH_2), 38.3 (CH_2), 33.8 (CH_2), 26.9 (CH_2), 23.0 (CH_3), 22.9 (CH_3), 20.8 (CH_2). **IR** (ATR): 3296, 3062, 2952, 1731, 1648, 1531, 1178, 731, 699 cm^{-1}. **MS** (ESI) *m/z* (relative intensity): 587 (100) $[M+Na]^+$, 565 (26) $[M+H]^+$. **HR-MS** (ESI) *m/z* calcd for $C_{30}H_{37}N_4O_7$ $[M+H]^+$: 565.2657; found: 565.2651.

(Acetyl L-alanyl)-glycyl-glycyl-L-valinyl-L-alanyl-glycyl-1-(2-pyridyl)-2-(benzyl propionate-3-yl)-L-tryptophyl-glycyl-glycinamide (12a):

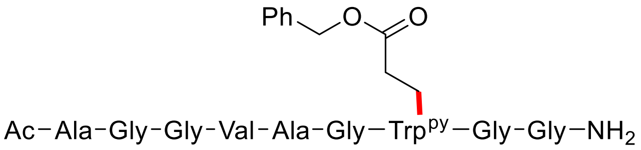

The general procedure E was followed using Ac-Ala-Gly-Gly-Val-Ala-Gly-Trp^{py}-Gly-Gly-Rink-Amide resin (85.9 mg, 0.35 mmol/g, 30.0 µmol) and benzyl acrylate (15 mg, 90 µmol) in 300 µL HOAc. Purification by crystallization (Et_2O, –20 °C) yielded **12a** as a white solid (21.0 mg, 20.8 µmol, 69%). **^{1}H NMR** (500 MHz, DMSO-d_6, 70 °C): δ 8.64–8.61 (m, 1H), 8.13–8.08 (m, 1H), 8.08–8.03 (m, 1H), 8.02–7.72 (m, 8H), 7.66–7.63 (m, 1H), 7.62–7.58 (d, J = 9.0 Hz, 1H), 7.55–7.52 (m, 1H), 7.49–7.46 (m, 1H), 7.19–7.16 (m, 1H), 7.12–7.06 (m, 4H), 6.95–6.80 (br s, 2H), 4.31–4.21 (m, 2H), 4.20–4.14 (m, 1H), 3.81–3.69 (m, 10H), 3.68–3.62 (m, 2H), 3.53 (s, 2H), 3.10–2.97 (m, 2H), 2.45–2.39 (m, 1H), 2.22–2.18 (m, 1H), 2.05–1.97 (m, 1H), 1.86 (s, 3H), 1.25 (d, J = 7.0 Hz, 3H), 1.22 (d, J = 7.0 Hz, 3H), 0.87 (d, J = 7.0 Hz, 3H), 0.84 (d, J = 7.0 Hz, 3H). **^{13}C NMR** (126 MHz, DMSO-d_6, 70 °C): δ 173.2 (C_q), 172.9 (C_q), 171.2 (C_q), 171.2 (C_q), 171.1 (C_q), 169.8 (C_q), 169.6 (C_q), 169.5 (C_q), 169.3 (C_q), 169.0 (C_q), 161.4 (CH), 149.3 (CH), 139.0 (CH), 128.7 (CH), 125.9 (CH), 122.6 (CH), 121.6 (CH), 121.2 (CH), 120.0 (CH), 118.5 (CH), 190.7 (CH), 70.2 (CH_2), 58.0 (CH), 48.9 (CH), 48.9 (CH), 42.7 (CH_2), 42.6 (CH_2), 42.4 (CH_2), 42.3 (CH_2), 42.3 (CH_2), 41.5 (CH_2), 40.8 (CH_2), 31.0 (CH), 30.6 (CH_2), 23.0 (CH_3), 19.0 (CH_3), 18.7 (CH_2), 17.9 (CH_3), 17.6 (CH_3), 17.6 (CH_3). **IR** (ATR): 3279, 2926, 1648, 1628, 1535, 1422, 1372, 1236, 1024, 672 cm^{-1}. **MS** (ESI) *m/z* (relative intensity) 1033 (9) $[M+Na]^+$, 1011 (100) $[M+H]^+$. **HR-MS** (ESI) *m/z* calcd for $C_{49}H_{62}N_{12}NaO_{12}^+$ 1033.6501 $[M+Na]^+$, found 1033.6487.

(Acetyl glycyl)-L-alanyl-L-alanyl-1-(2-pyridyl)-2-(octane-3-one-1-yl)-L-tryptophyl-glycyl-L-phenylalanyl-L-valin (12b):

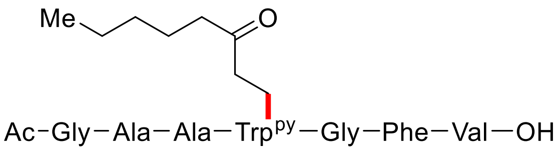

The general procedure E was followed using Ac-Gly-Ala-Ala-Trp^{py}-Gly-Phe-Val-Wang resin (60.0 mg, 0.63 mmol/g, 37.8 µmol) and oct-1-en-3-one (24 mg, 190 mol) in 300 µL HOAc. Purification by crystallization (Et_2O, –20 °C) yielded **12b** as a white solid (28.2 mg, 29.6 µmol, 78%). **^{1}H NMR** (500 MHz, CD_3OD): δ 8.62 (ddd, J = 5.0, 2.0, 0.8 Hz, 1H), 8.08 (ddd, J = 7.7, 7.7, 1.9 Hz, 1H), 7.89

(s, 1H), 7.62–7.59 (m, 1H), 7.55 (ddd, *J* = 7.9, 1.0, 1.0 Hz, 1H), 7.49 (ddd, *J* = 7.6, 5.0, 1.1 Hz, 1H), 7.28–7.20 (m, 4H), 7.20–7.14 (m, 2H), 7.13–7.06 (m, 2H), 5.18 (s, 2H), 4.64–4.59 (m, 1H), 4.55–4.50 (m, 1H), 4.32–4.23 (m, 2H), 4.19–4.15 (m, 1H), 3.97–3.82 (m, 3H), 3.45–3.36 (m, 1H), 3.26–3.21 (m, 1H), 3.23–3.16 (m, 1H), 3.15–3.06 (m, 1H), 3.01–2.93 (m, 1H), 2.54–2.41 (m, 2H), 2.24 (dd, *J* = 7.3, 7.3 Hz, 2H), 2.16–2.10 (m, 1H), 1.98 (s, 3H), 1.43–1.34 (m, 4H), 1.33 (d, *J* = 7.0 Hz, 3H), 1.24 (d, *J* = 7.0 Hz, 3H), 1.23–1.18 (m, 2H), 1.15–1.08 (m, 2H), 0.92 (d, *J* = 7.0 Hz, 3H), 0.91 (d, *J* = 7.0 Hz, 3H), 0.82 (t, *J* = 7.0 Hz, 3H). **^{13}C NMR** (126 MHz, CD_3OD): δ 212.2 (C_q), 175.6 (C_q), 175.0 (C_q), 174.1 (C_q), 174.0 (C_q), 172.7 (C_q), 172.4 (C_q), 171.4 (C_q), 152.6 (C_q), 150.6 (CH), 140.8 (CH), 138.9 (C_q), 138.5 (C_q), 138.4 (C_q), 131.0 (CH), 130.4 (C_q), 129.8 (CH), 127.6 (CH), 124.2 (CH), 123.4 (CH), 123.3 (CH), 121.8 (CH), 119.6 (CH), 111.1 (C_q), 110.9 (CH), 61.4 (CH), 56.6 (CH), 56.3 (CH), 51.4 (CH), 51.0 (CH), 44.0 (CH_2), 43.6 (CH_2), 43.5 (CH_2), 42.8 (CH_2), 38.5 (CH_2), 32.7 (CH), 32.4 (CH_2), 27.5 (CH_2), 24.6 (CH_2), 23.5 (CH_2), 22.6 (CH_3), 20.2 (CH_2), 20.2 (CH_3), 18.6 (CH_3), 17.6 (CH_3), 17.3 (CH_3), 14.8 (CH_3). **IR** (ATR): 3272, 2954, 2926, 1627, 1532, 1451, 1436, 1387, 1370, 1261, 1174, 873, 710 cm^{-1}. **MS** (ESI) *m/z* (relative intensity) 974 (100) $[M+Na]^+$, 952 (9) $[M+H]^+$. **HR-MS** (ESI) *m/z* calcd for $C_{50}H_{65}N_9NaO_{10}^+$ 974.4747 $[M+Na]^+$, found 974.4730.

(Acetyl L-alanyl)-L-arginyl-glycyl-L-alanyl-L-alanyl-L-leucyl-1-(2-pyridyl)-2-({2-[2-(2-methoxy-ethoxy)ethoxy]ethyl} propionate-3-yl)-L-tryptophyl-L-valinyl-L-valinamide (12c):

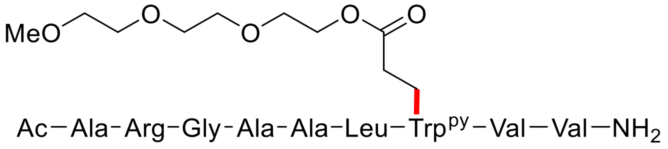

The general procedure E was followed using Ac-Ala-Arg(Pbf)-Gly-Ala-Ala-Leu-Trp^{py}-Val-Val-Rink-Amide resin (81.0 mg, 0.37 mmol/g, 30.0 µmol) and 2-[2-(2-methoxyethoxy)ethoxy]ethyl acrylate (65 mg, 300 µmol) in 300 µL HOAc. Purification by crystallization (Et_2O, –20 °C) yielded **12c** as a white solid (17.2 mg, 13.6 µmol, 45%). **^{1}H NMR** (500 MHz, DMSO-d_6): δ 8.6–8.54 (m, 1H), 8.10–7.91 (m, 3H), 7.91–7-78 (m, 3H), 7.78–7.60 (m, 2H), 7.60–7.52 (m, 1H), 7.52–7.42 (m, 3H), 7.42–7.34 (m, 3H), 7.20–7.05 (m, 2H), 7.05–6.88 (br s, 3H), 6.88–6.68 (br s, 1H), 4.35–4.15 (m, 7H), 4.15–4.08 (m, 1H), 3.76–3.68 (m, 2H), 3.58–3.32 (m, 12H), 3.24 (s, 3H), 3.16–3.07 (m, 4H), 2.54–2.48 (m, 2H), 2.38–2.32 (m, 2H), 2.08–1.92 (m, 2H), 1.86 (s, 3H), 1.81–1.69 (m, 1H), 1.68–1.56 (m, 2H), 1.56–1.45 (m, 3H), 1.45–1.32 (m, 1H), 1.32–1.16 (m, 9H), 0.92–0.77 (m, 18H). **^{13}C NMR** (126 MHz, DMSO-d_6): δ 172.6 (C_q), 172.3 (C_q), 172.3 (C_q), 171.6 (C_q), 171.6 (C_q), 171.5 (C_q), 171.5 (C_q), 171.4 (C_q), 171.3 (C_q), 170.3 (C_q), 169.2 (C_q), 164.6 (C_q), 156.7 (C_q), 150.5 (C_q), 149.2 (CH), 138.5 (CH), 136.2 (C_q), 128.5 (C_q), 125.7 (CH), 122.3 (CH), 121.0 (CH), 119.7 (CH), 113.6 (CH), 111.7 (C_q), 110.5 (CH), 71.1 (CH_2), 71.0 (CH_2), 69.6 (CH_2), 69.5 (CH_2), 69.5 (CH_2), 69.4 (CH_2), 57.7 (CH_3), 57.7 (CH), 57.3 (CH), 57.3 (CH), 52.1 (CH), 51.2 (CH), 48.4 (CH), 48.2 (CH), 48.1 (CH), 42.1 (CH_2), 41.9 (CH_2), 41.9 (CH_2), 30.9

(CH_2), 30.8 (CH_2), 30.0 (CH), 29.9 (CH), 28.6 (CH_2), 24.6 (CH_2), 24.6 (CH_2), 24.0 (CH), 22.6 (CH_3), 22.2 (CH_3), 22.1 (CH_3), 21.5 (CH_3), 21.5 (CH_3), 18.9 (CH_3), 17.7 (CH_3), 17.7 (CH_3), 17.6 (CH_3), 17.5 (CH_3). **IR** (ATR): 3278, 2954, 1618, 1509, 1472, 1094 cm^{-1}. **MS** (ESI) *m/z* (relative intensity) 1278 (5) $[M+H]^+$, 1066 (15), 746 (100) 641 (18). **HR-MS** (ESI) *m/z* calcd for $C_{61}H_{96}N_{15}O_{15}^+$ 1278.7205 $[M+H]^+$, found 1278.7169.

(Acetyl L-alanyl)-L-argininyl-glycyl-L-alanyl-L-alanyl-L-leucyl-1-(2-pyridyl)-2-(butane-3-one-1-yl)-L-tryptophyl-L-valinyl-L-valinamide (12d):

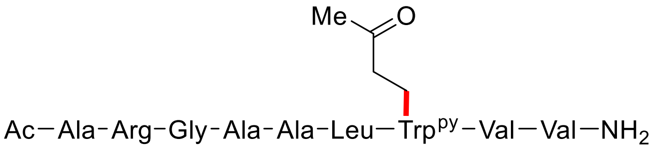

The general procedure E was followed using Ac-Ala-Arg(Pbf)-Gly-Ala-Ala-Leu-Trp^{py}-Val-Val-Rink-Amide resin (81.0 mg, 0.37 mmol/g, 30.0 µmol) and but-3-en-2-one (21 mg, 300 µmol) in 300 µL HOAc. Purification by crystallization (Et_2O, –20 °C) yielded **12d** as a white solid (24.0 mg, 21.2 µmol, 71%). ^{1}**H NMR** (500 MHz, DMSO-d_6): δ 8.66–8.60 (m, 1H), 8.25–7.82 (m, 11H), 7.81–7.60 (m, 4H), 7.56 (d, J = 7.5 Hz, 1H), 7.52–7.43 (m, 2H), 7.37–7.21 (m, 2H), 7.21–7.11 (m, 2H), 7.11–7.04 (m, 2H), 7.04–6.98 (m, 2H), 4.70–4.60 (m, 1H), 4.40–4.13 (m, 8H), 4.13–4.02 (m, 1H), 3.77–3.64 (m, 2H), 3.23–3.12 (m, 1H), 3.12–3.03 (m, 2H), 3.03–2.91 (m, 3H), 2.48–2.35 (m, 2H), 1.95 (s, 3H), 1.83 (s, 3H), 1.75–1.64 (m, 1H), 1.64–1.41 (m, 5H), 1.41–1.31 (m, 1H), 1.30–1.08 (m, 7H), 1.02–0.94 (m, 3H), 0.92–0.73 (m, 18H). ^{13}**C NMR** (126 MHz, DMSO-d_6): δ 207.7 (C_q), 173.2 (C_q), 172.4 (C_q), 172.3 (C_q), 172.2 (C_q), 172.1 (C_q), 171.6 (C_q), 171.1 (C_q), 170.9 (C_q), 169.8 (C_q), 168.7 (C_q), 157.1 (C_q), 151.1 (C_q), 150.0 (CH), 139.7 (CH), 137.7 (C_q), 136.7 (C_q), 135.2 (C_q), 128.7 (C_q), 123.2 (CH), 122.3 (CH), 121.8 (CH), 120.5 (CH), 119.3 (CH), 110.3 (CH), 58.1 (CH), 58.0 (CH), 57.9 (CH), 51.8 (CH), 49.0 (CH), 48.8 (CH), 48.7 (CH), 48.5 (CH), 43.2 (CH_2), 42.3 (CH_2), 41.2 (CH_2), 40.9 (CH_2), 31.0 (CH_3), 29.5 (CH_2), 27.6 (CH_2), 25.4 (CH_2), 24.7 (CH), 24.6 (CH), 23.6 (CH_3), 23.5 (CH_3), 23.0 (CH_3), 22.8 (CH_3), 22.2 (CH_3), 22.1 (CH_3), 19.8 (CH_3), 19.2 (CH_2), 18.6 (CH_3), 18.4 (CH_3), 18.3 (CH_3), 13.0 (CH). **IR** (ATR): 3277, 2960, 1622, 1519, 1470, 1165, 697 cm^{-1}. **MS** (ESI) *m/z* (relative intensity) 1152 (2) $[M+Na]^+$, 1130 (85) $[M+H]^+$, 1060 (35), 797 (85), 663 (100). **HR-MS** (ESI) *m/z* calcd for $C_{55}H_{84}N_{15}O_{11}^+$ 1130.6469 $[M+H]^+$, found 1130.6461.

(Acetyl L-isoleucyl)-L-alanyl-L-glutaminyl-glycyl-L-leucyl-glycyl-L-alanyl-(1-(2-pyridyl)-2-({2-[2-(2-methoxyethoxy)ethoxy]ethyl} propionate-3-yl)-L-tryptophyl)-glycyl-L-alaninamide (12e):

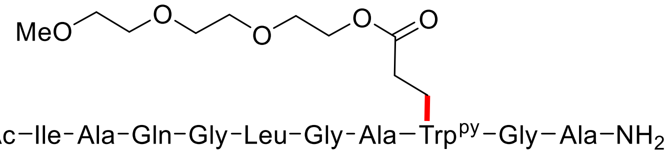

The general procedure E was followed using Ac-Ile-Ala-Gln-Gly-Leu-Gly-Ala-Trp^{py}-Gly-Ala-Rink-Amide resin (81.0 mg, 0.37 mmol/g, 30.0 µmol) and 2-[2-(2-methoxyethoxy)ethoxy]ethyl acrylate

(65 mg, 300 µmol) in 300 µL HOAc. Purification by crystallization (Et_2O, –20 °C) yielded **12e** as a white solid (16.5 mg, 12.9 µmol, 43%). **^{1}H NMR** (600 MHz, DMSO-d_6): δ 8.64–8.62 (m, 1H), 8.21 (dd, *J* = 5.7, 5.7 Hz, 1H), 8.18 (dd, *J* = 5.7, 5.7 Hz, 1H), 8.10–8.05 (m, 3H), 8.02 (d, *J* = 7.0 Hz, 1H), 7.96 (d, *J* = 7.9 Hz, 1H), 7.94–7.89 (m, 2H), 7.81 (d, *J* = 7.5 Hz, 1H), 7.66–7.64 (m, 1H), 7.55–7.52 (m, 1H), 7.49–7.45 (m, 1H), 7.26–7.23 (m, 1H), 7.22–7.19 (m, 1H), 7.19–7.17 (m, 1H), 7.10–7.07 (m, 2H), 7.01 (br s, 1H), 6.74 (br s, 1H), 4.34–4.09 (m, 7H), 3.80–3.64 (m, 6H), 3.55–3.11 (m, 15H), 2.15–2.05 (m, 2H), 1.92–1.82 (m, 1H), 1.85 (s, 3H), 1.77–1.64 (m, 2H), 1.62–1.53 (m, 1H), 1.52–1.36 (m, 3H), 1.29–1.12 (m, 10H), 1.12–1.03 (m, 1H), 0.92–0.76 (m, 12H). **^{13}C NMR** (126 MHz, DMSO-d_6): δ 174.4 (C_q), 174.4 (C_q), 174.0 (C_q), 172.9 (C_q), 172.6 (C_q), 172.3 (C_q), 171.7 (C_q), 171.3 (C_q), 169.7 (C_q), 169.0 (C_q), 169.0 (C_q), 168.5 (C_q), 161.8 (CH), 155.0 (C_q), 152.7 (C_q), 148.9 (CH), 139.7 (CH), 136.0 (C_q), 135.2 (C_q), 130.4 (C_q), 129.3 (CH), 126.6 (CH), 121.4 (CH), 120.4 (CH), 119.4 (CH), 114.8 (C_q), 114.3 (CH), 71.7 (CH_2), 71.6 (CH_2), 70.2 (CH_2), 70.1 (CH_2), 70.0 (CH_2), 69.9 (CH_2), 58.5 (CH_3), 57.4 (CH), 52.8 (CH), 51.6 (CH), 49.0 (CH), 48.7 (CH), 48.5 (CH), 48.4 (CH), 42.6 (CH_2), 42.4 (CH_2), 42.4 (CH_2), 41.2 (CH_2), 41.1 (CH_2), 36.9 (CH), 31.8 (CH_2), 28.4 (CH_2), 24.9 (CH_2), 24.6 (CH), 23.5 (CH_3), 23.0 (CH_3), 22.1 (CH_3), 18.7 (CH_3), 18.3 (CH_3), 18.2 (CH_3), 15.8 (CH_3), 11.5 (CH_3). **IR** (ATR): 3290, 2960, 2935, 1652, 1539, 1454, 1412, 1242, 1202, 1132, 1025, 751 cm^{-1}. **MS** (ESI) *m/z* (relative intensity) 1301 (1) $[M+Na]^+$, 820 (100). **HR-MS** (ESI) *m/z* calcd for $C_{60}H_{90}N_{14}NaO_{17}^+$ 1301.6501 $[M+Na]^+$, found 1301.6477.

Peptide Ligation on Resin: The general procedure E was followed using Ac-Gly-Trp^{py}-Gly-Gly-Rink-Amide resin (250 µmol, 0.55 mmol/g) and (*R*)-2-(2-acetamido-3-phenylpropanamido)ethyl acrylate (1.52 g, 5.0 mmol) in 3.0 mL HOAc. Purification by crystallization (Et_2O, –20 °C) yielded a mixture that was analyzed by LC-MS (flow rate was set at 0.25 mL/min), showing 12% conversion to a mixture of hydroarylated and alkenylated product.

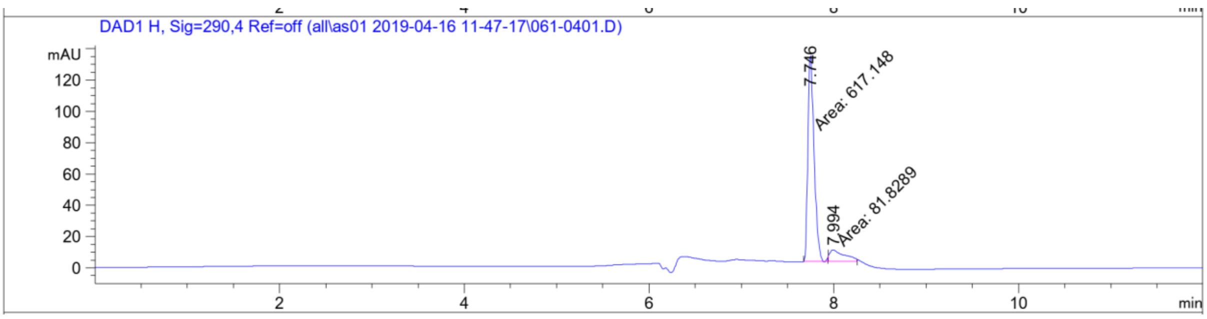

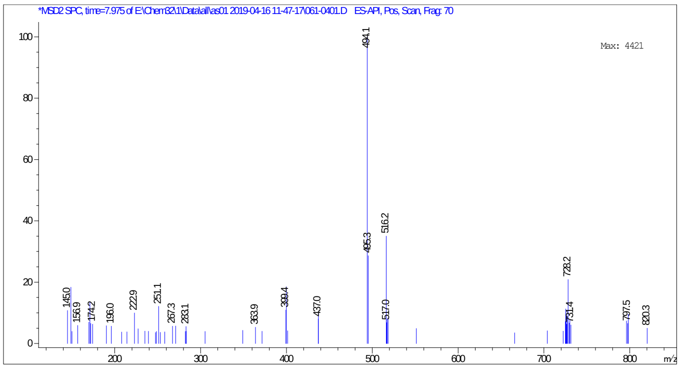

Signal 3: DAD1 H, Sig=290,4 Ref=off

Peak #	RetTime [min]	Type	Width [min]	Area [mAU*s]	Height [mAU]	Area %
1	7.746	MM	0.0781	617.14752	131.67651	88.2930
2	7.994	MM	0.1876	81.82895	7.27148	11.7070
Totals :				698.97647	138.94799	

Supplementary Figure 13. LC-MS trace of the C–H ligation on resin.

For reference the LC-MS trace of Ac-Gly-Trp^{py}-Gly-Gly-NH_2 is given (flow rate was set at 0.25 mL/min).

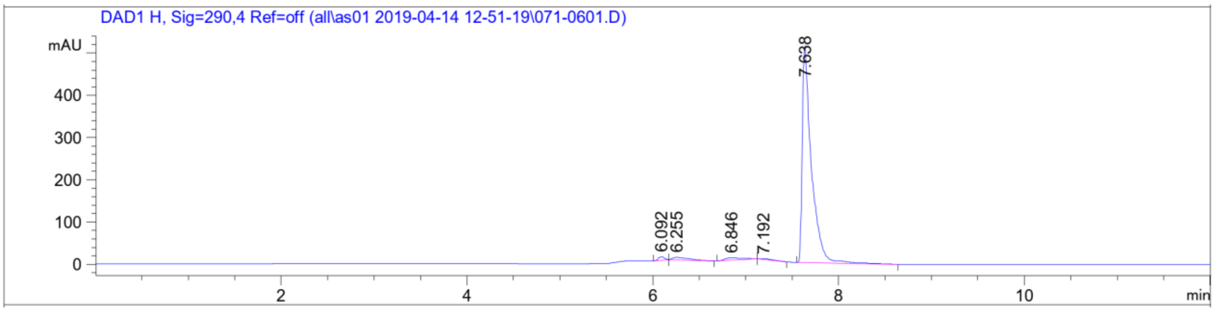

Supplementary Figure 14. LC-MS trace of Ac-Gly-Trp^{py}-Gly-Gly-NH_2.

Computation Data: All calculations were performed using Gaussian 16, Revision A.03 package.[10] Geometry optimizations were performed at the PBE0 level of theory[11] including D3(BJ) dispersion corrections or at the M06 level of theory[12] including D3 correction.[13-14] Ruthenium was described with def2-SVP basis set[15] in combination with SDD pseudopotential,[16] while on all other atoms def2-SVP basis set was used. All stationary points were fully characterized by analytical frequency calculations as either a minimum or a transition state (exactly one imaginary frequency). IRC calculations were used to confirm the intermediates linked by each transition state. Single point calculations were performed at the PBE0 level of theory or at the PW6B95 level of theory[17] including D3(BJ) dispersion corrections with def2-QZVP basis set[15] and SDD pseudopotential on ruthenium, def2-QZVP(-f) basis set on hydrogen and def2-QZVP(-g) basis set on all other atoms. This basis set is denoted as def2-QZVP* throughout the manuscript. Solvent effects were taken into consideration in the single point calculations through the use of the SMD continuum solvation model with acetic acid (ε = 6.2528) as implemented in Gaussian.[18] Unless otherwise stated all reported energies are Gibbs free energies in kcal mol^{-1}, which were calculated by adding the gas-phase Gibbs free energy correction (ΔΔG) at 353 K and 1 atm to the single point energies. Superscripts correspond to the coordinated arene-ligand. If no superscript is given, *p*-cymene is assumed.

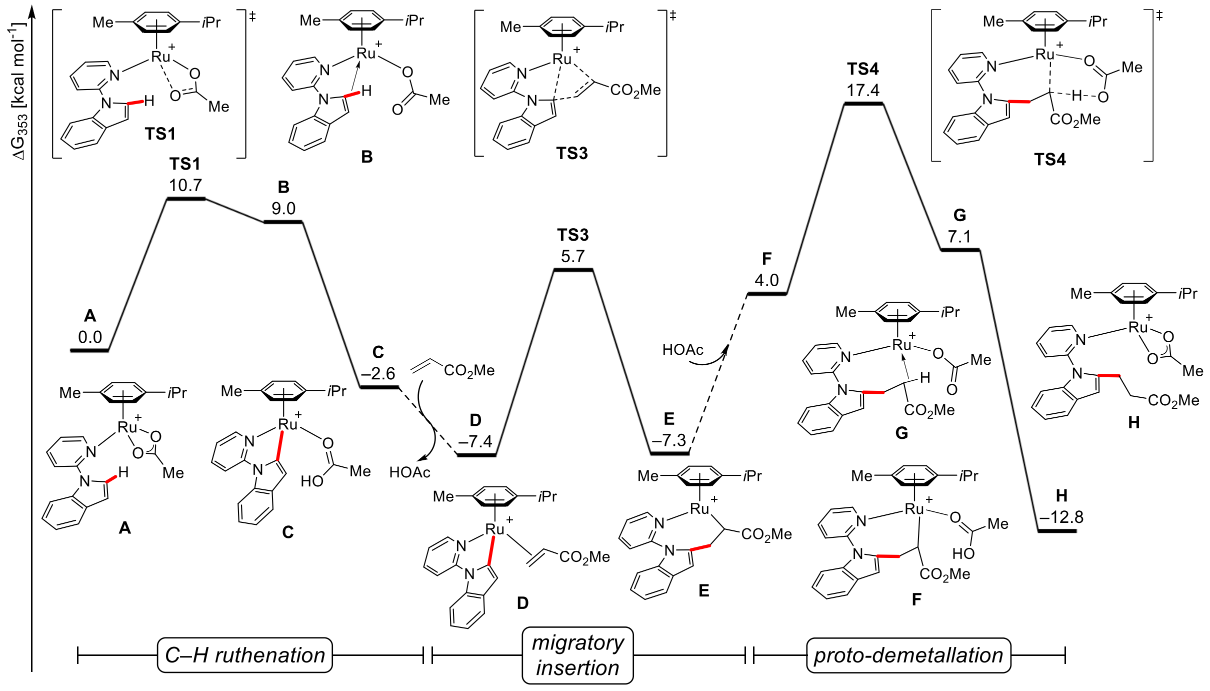

Supplementary Figure 15. Gibbs free energy profile at the PBE0-D3(BJ)/def2-QZVP*+SMD(AcOH)//PBE0-D3(BJ)/def2-SVP level of theory.

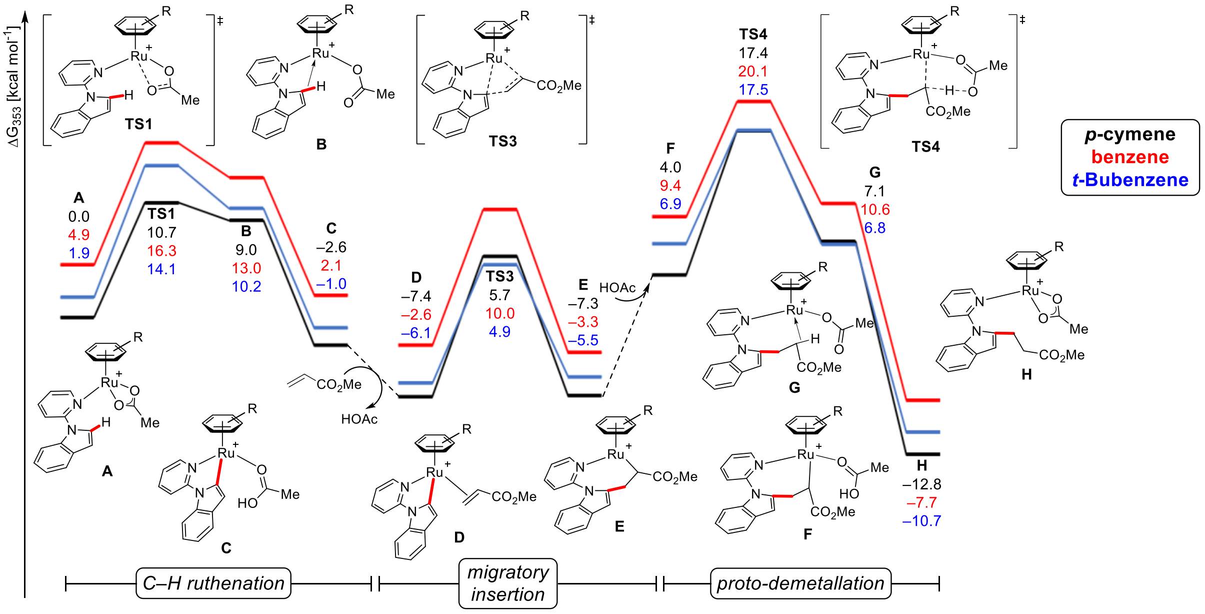

Supplementary Figure 16. Influence of η[6]–coordinated arene-ligand:Gibbs free energy profile at the PBE0-D3(BJ)/def2-QZVP*+SMD(AcOH)//PBE0-D3(BJ)/def2-SVP level of theory with various η[6]-arene-ligands on ruthenium: *p*-cymene (black line), benzene (red) and *t*-Bubenzene (blue).

Formation of catalytically active complex: The catalytically active complex [Ru(OAc)$_2$(*p*-cymene)] is genereated *in situ* under the reaction conditions from the pre-catalyst [RuCl$_2$(*p*-cymene)]$_2$ and HOAc. This salt metathesis/ligand exchange involves the formation of 2 molecules of HCl per ruthenium. The structures were optimized in the gas phase and the energies corrected to the condensed phase with the use of the SMD continuum solvation model. This model neglects specific solvent-solute interactions and hydrogen-bond formations, which are expected to significantly stabilize the dissolved HCl. Furthermore, a possible dissociation of HCl into Cl$^-$ and the formation of H·(HOAc)$_n^+$ clusters is not considered within this model. The calculated energies for the formation of [Ru(OAc)$_2$(*p*-cymene)] are therefore not directly related to the actual reaction conditions and should be considered with caution.

$$[\mathrm{RuCl_2}(p\text{-cymene})]_2 + 4\ \mathrm{HOAc} \rightleftharpoons 2\ [\mathrm{Ru(OAc)_2}(p\text{-cymene})] + 4\ \mathrm{HCl}$$

$$\Delta G = 46.3\ \mathrm{kcal\ mol^{-1}}$$

(PBE0/def2-QZVP*//PBE0/def2-SVP level of theory)

Supplementary Figure 17. Formation of catalytically active complex.

Formation of mono-cationic complex: Starting from the bis-carboxylate complex [Ru(OAc)$_2$(*p*-cymene)], mono-cationic complex **A** is formed by coordination of pyridyl indole *via* the pyridine

nitrogen and decoordination of one acetate ligand. Due to the separation into the cationic complex **A** and an anionic acetate, this process was calculated to be endergonic by 13.8 kcal mol^{-1}. However, the reaction is conducted in polar protic acetic acid as solvent, which should stabilize solvent-separated and contact ion-pairs. This effect can unfortunately not be described within the framework of the employed SMD continuum solvation model.

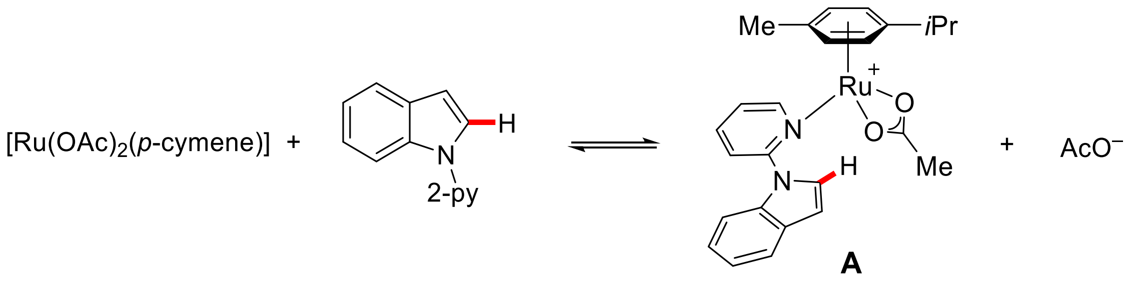

ΔG = 13.8 kcal mol^{-1}

(PBE0/def2-QZVP*//PBE0/def2-SVP level of theory)

Supplementary Figure 18. Formation of mono-cationic complex.

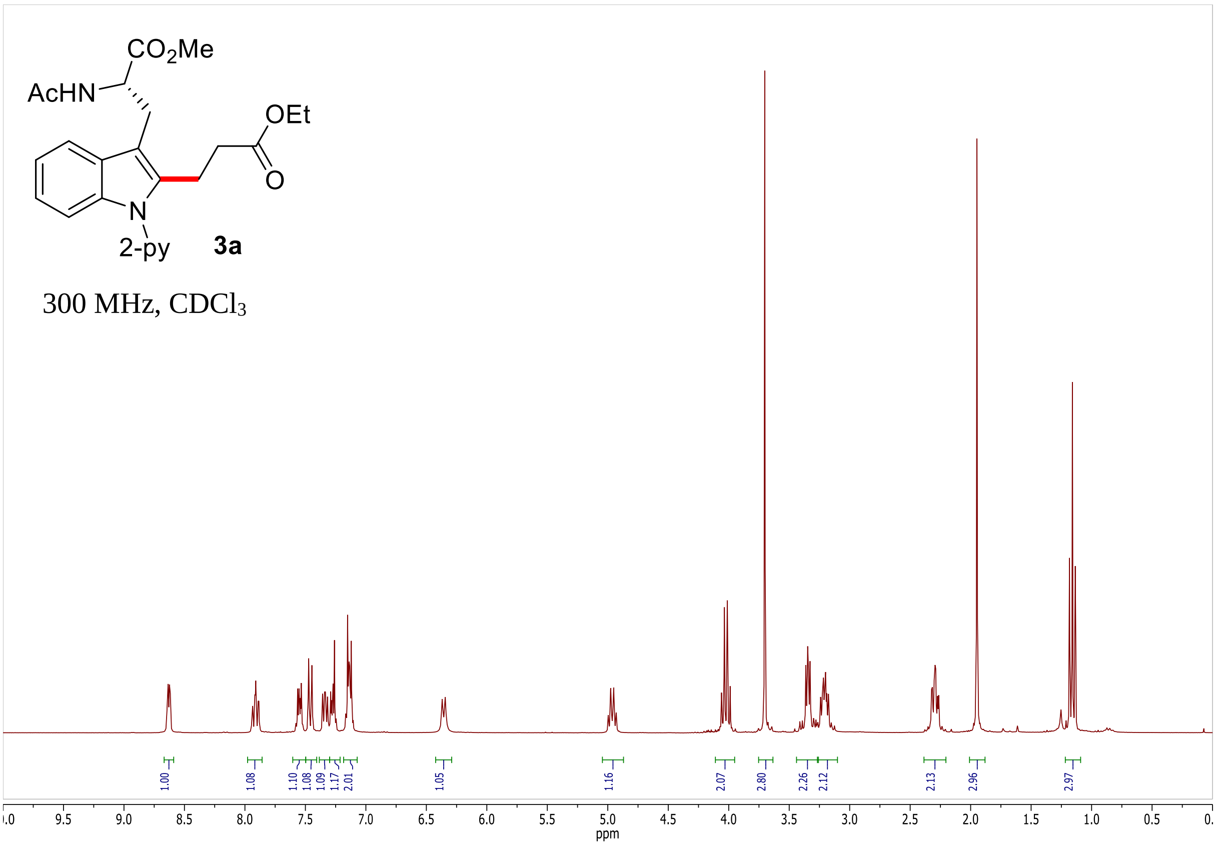

Supplementary Figure 19. ^{1}H-NMR spectrum of **3a**.

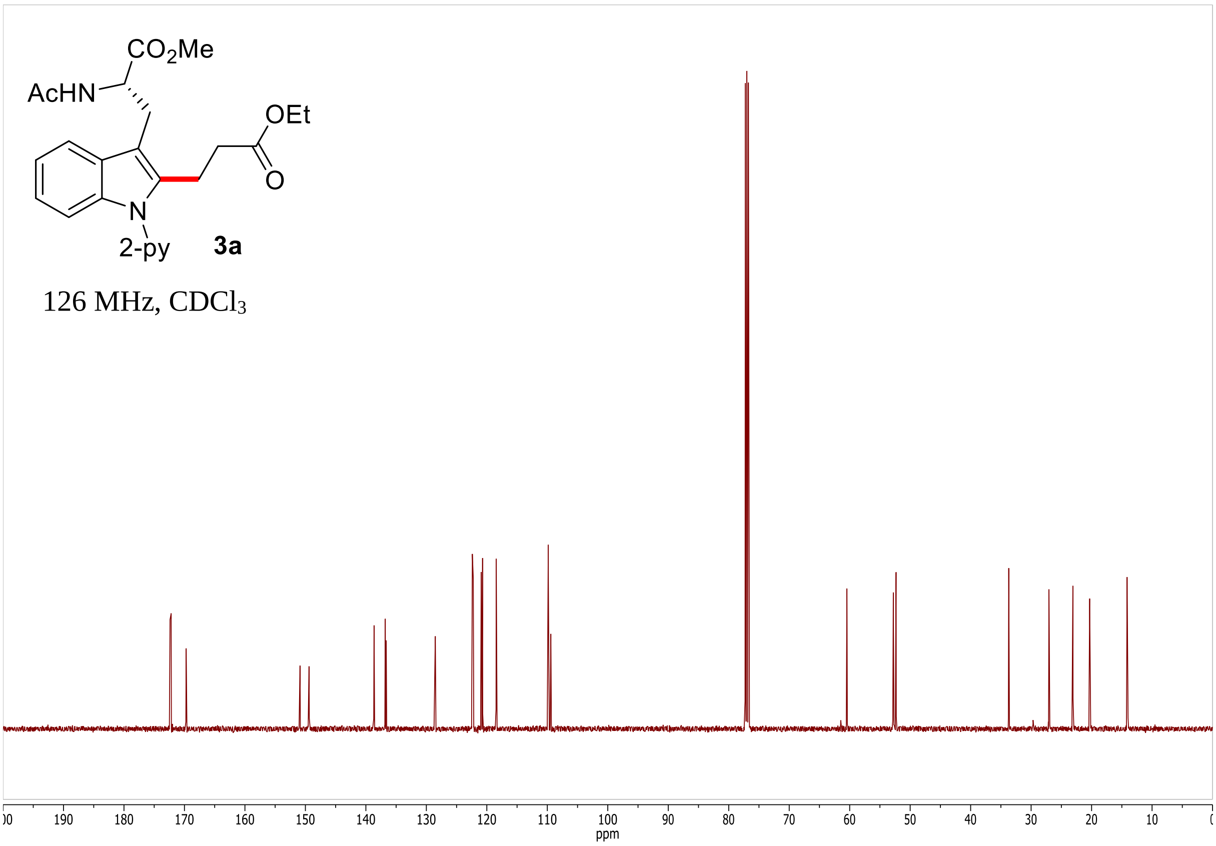

Supplementary Figure 20. ^{13}C-NMR spectrum of **3a**.

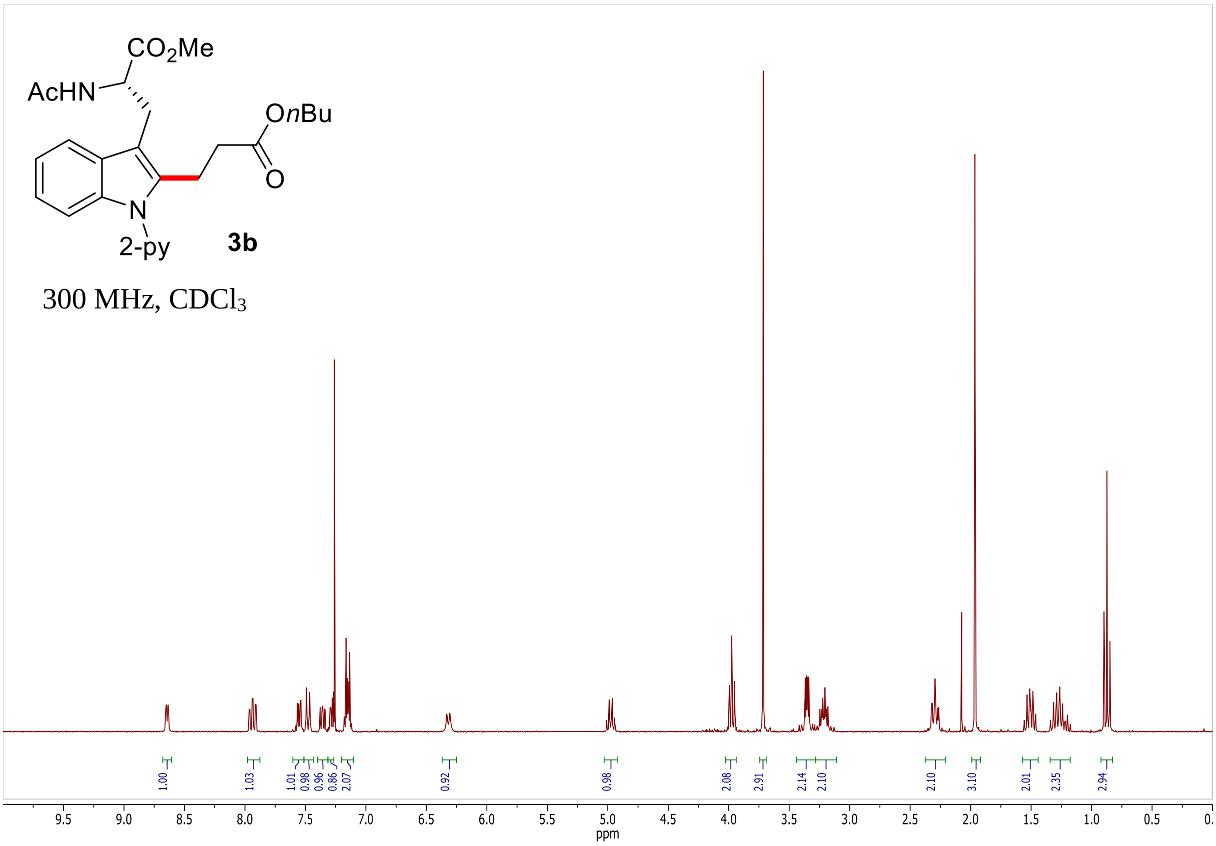

Supplementary Figure 21. ^{1}H-NMR spectrum of **3b**.

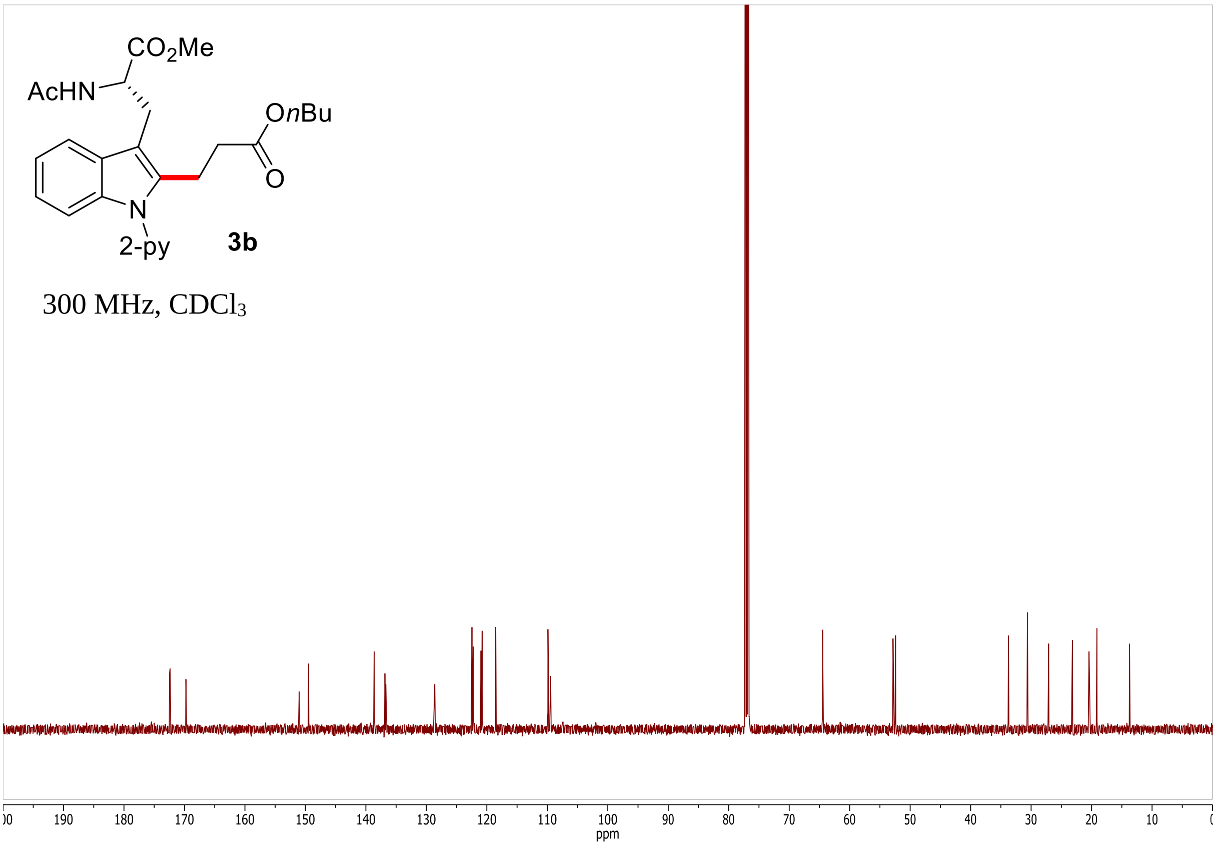

Supplementary Figure 22. ^{13}C-NMR spectrum of **3b**.

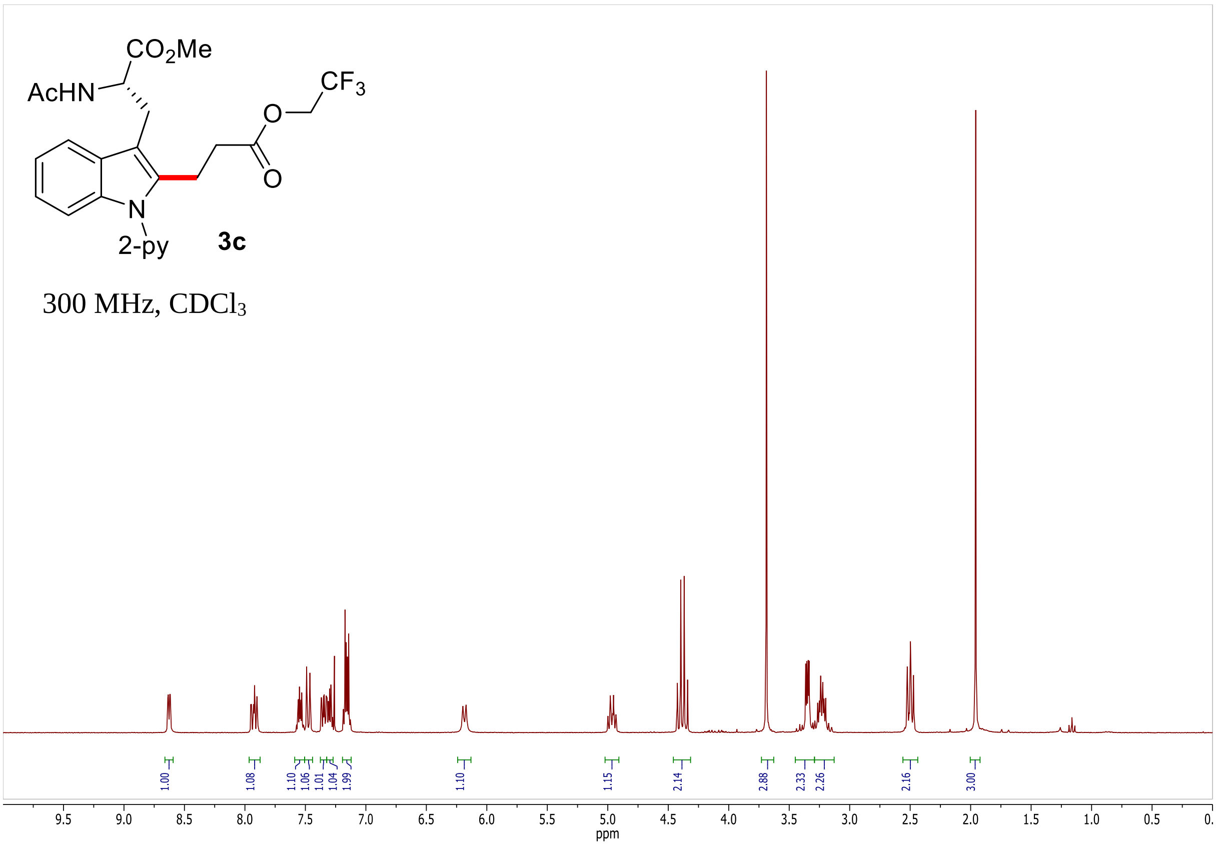

Supplementary Figure 23. ^{1}H-NMR spectrum of **3c**.

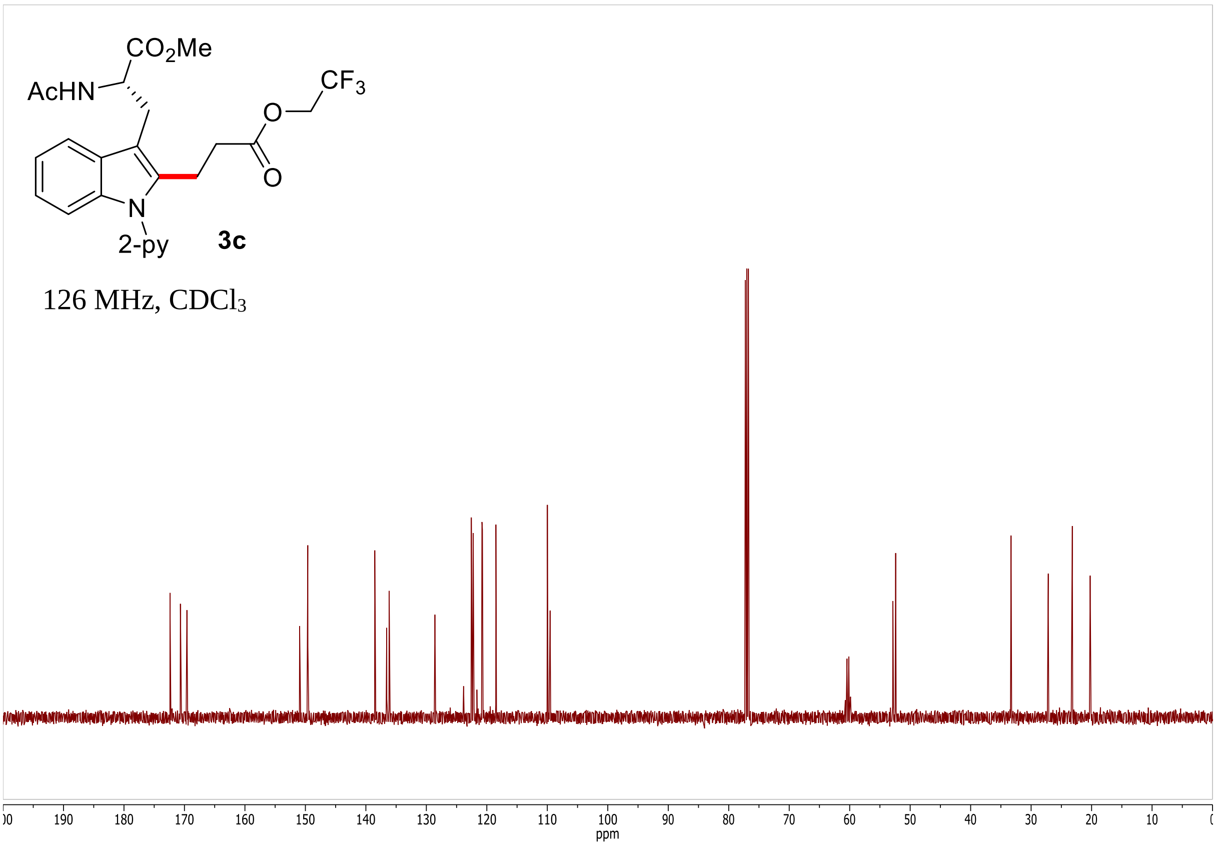

Supplementary Figure 24. ^{13}C-NMR spectrum of **3c**.

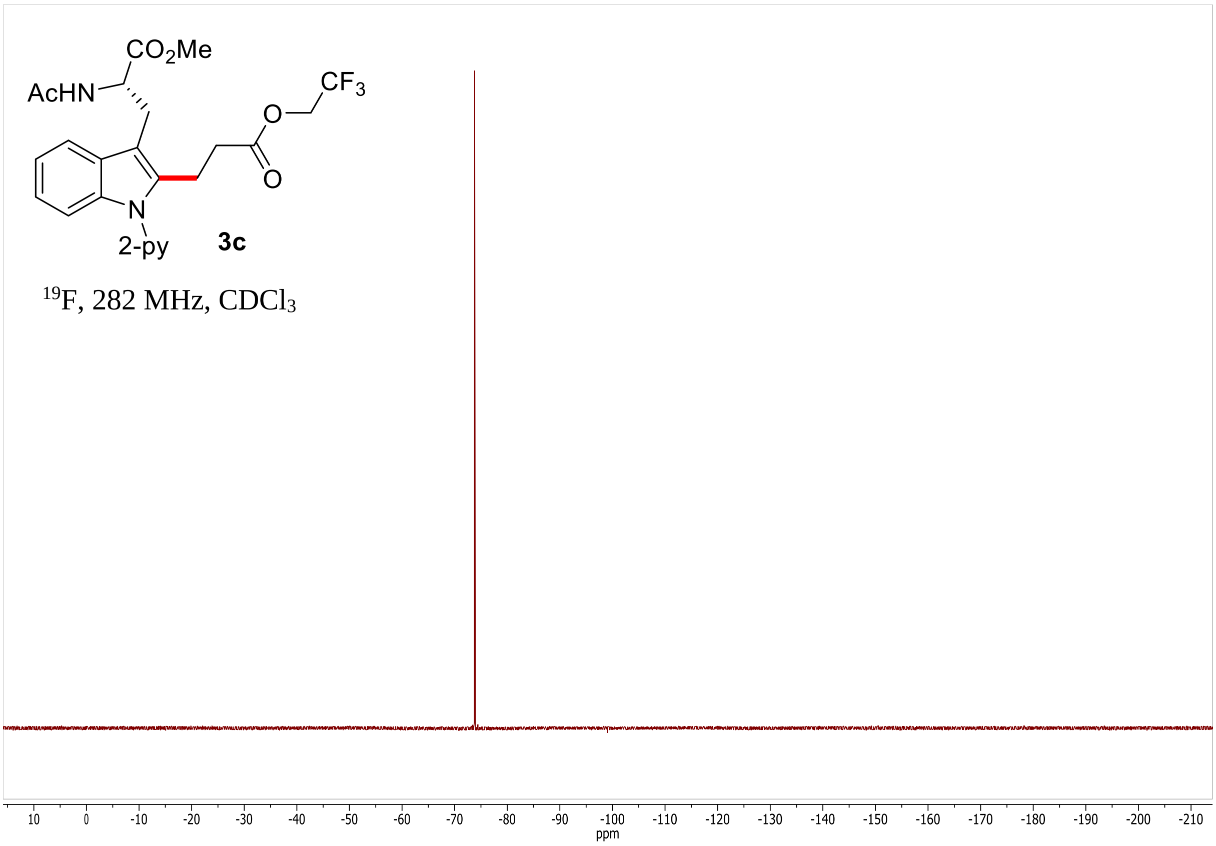

Supplementary Figure 25. ^{19}F-NMR spectrum of **3c**.

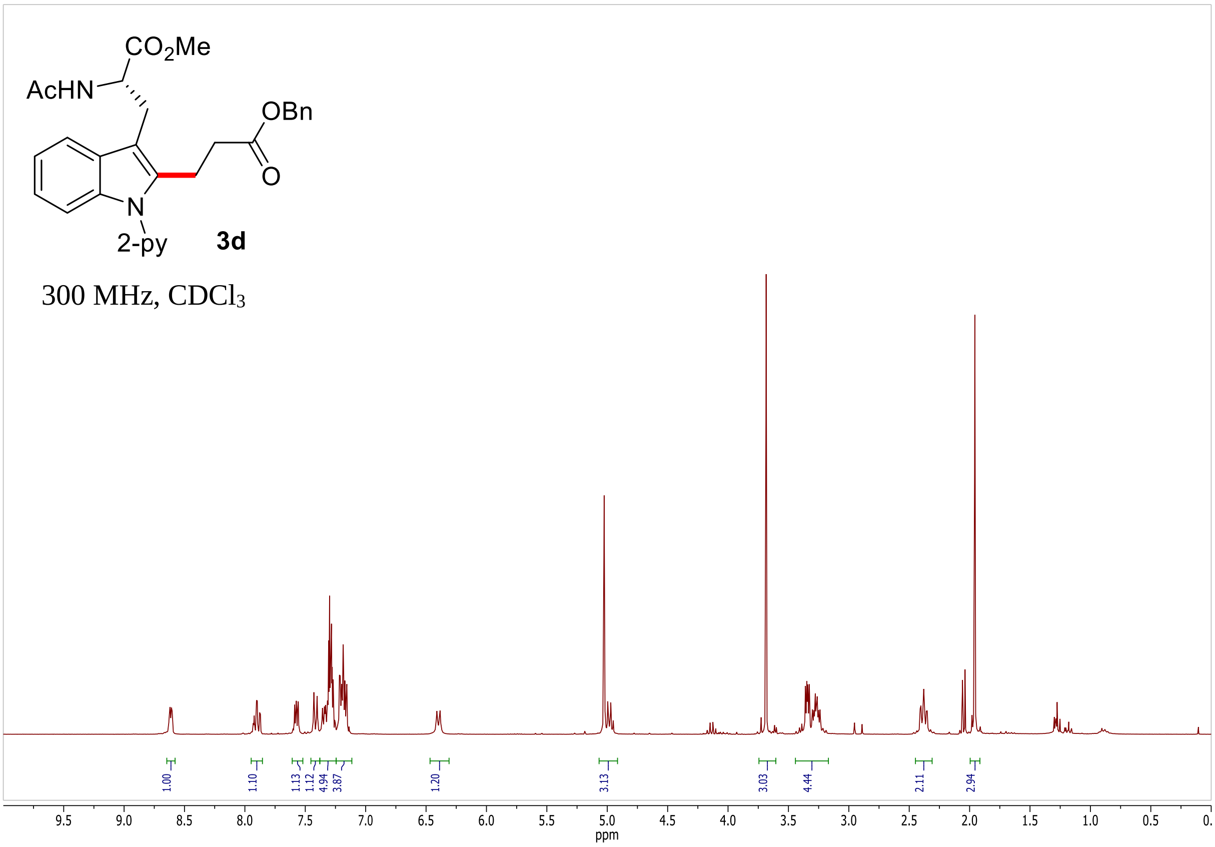

Supplementary Figure 26. ^{1}H-NMR spectrum of **3d**.

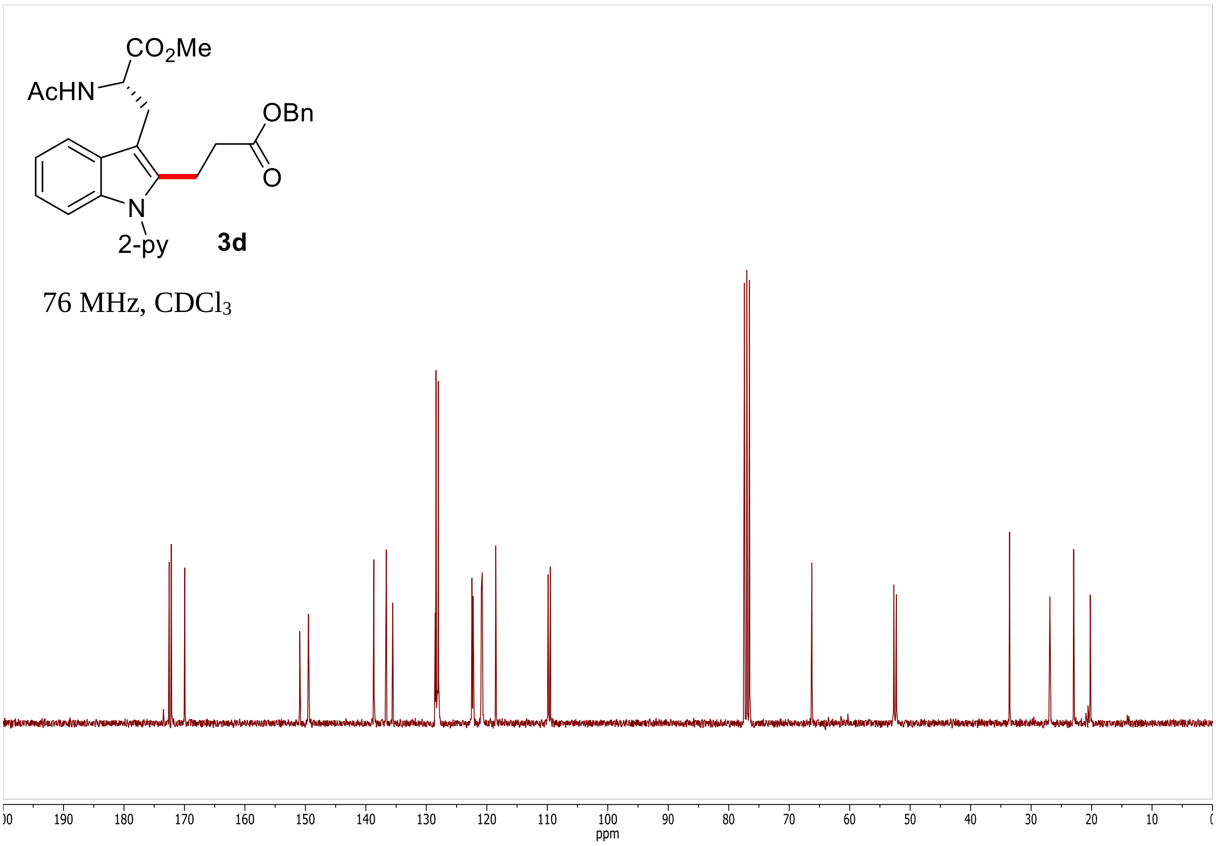

Supplementary Figure 27. ^{13}C-NMR spectrum of **3ad**.

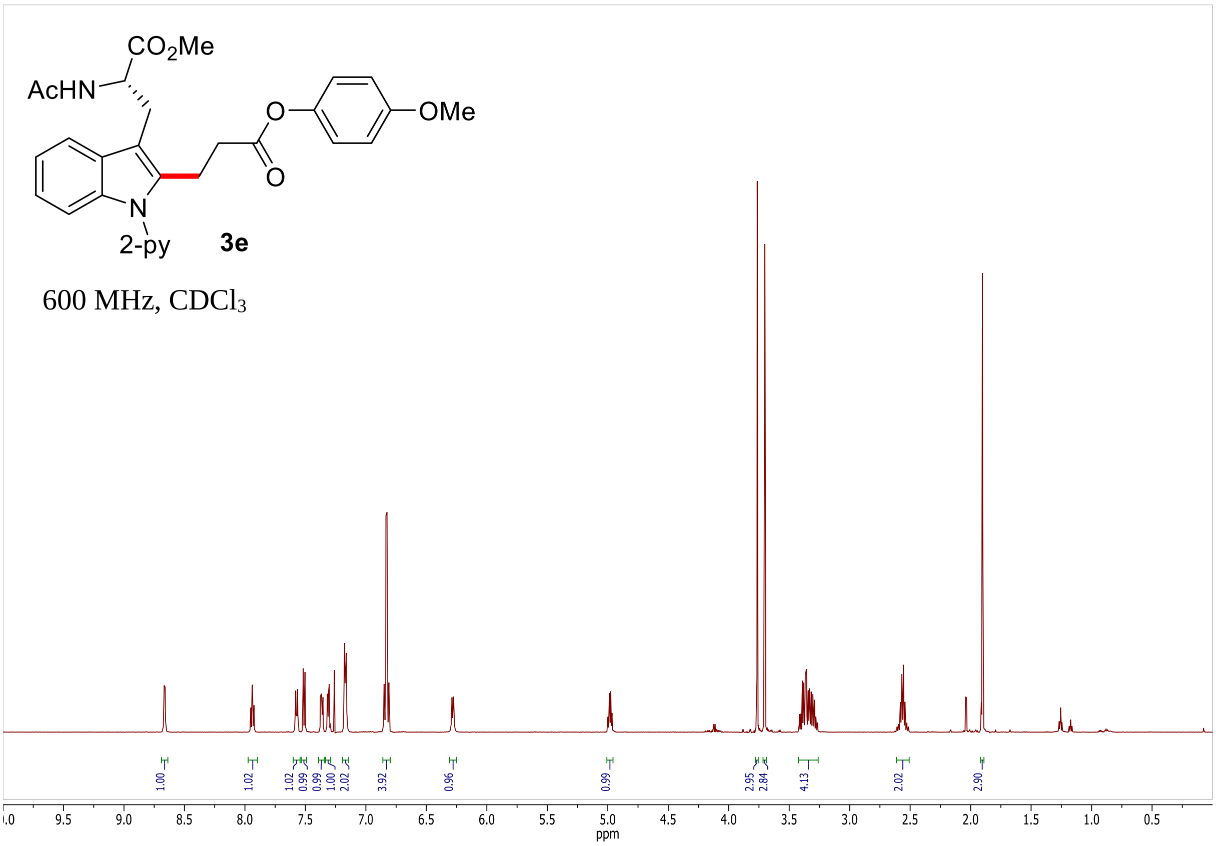

Supplementary Figure 28. ^{1}H-NMR spectrum of **3e**.

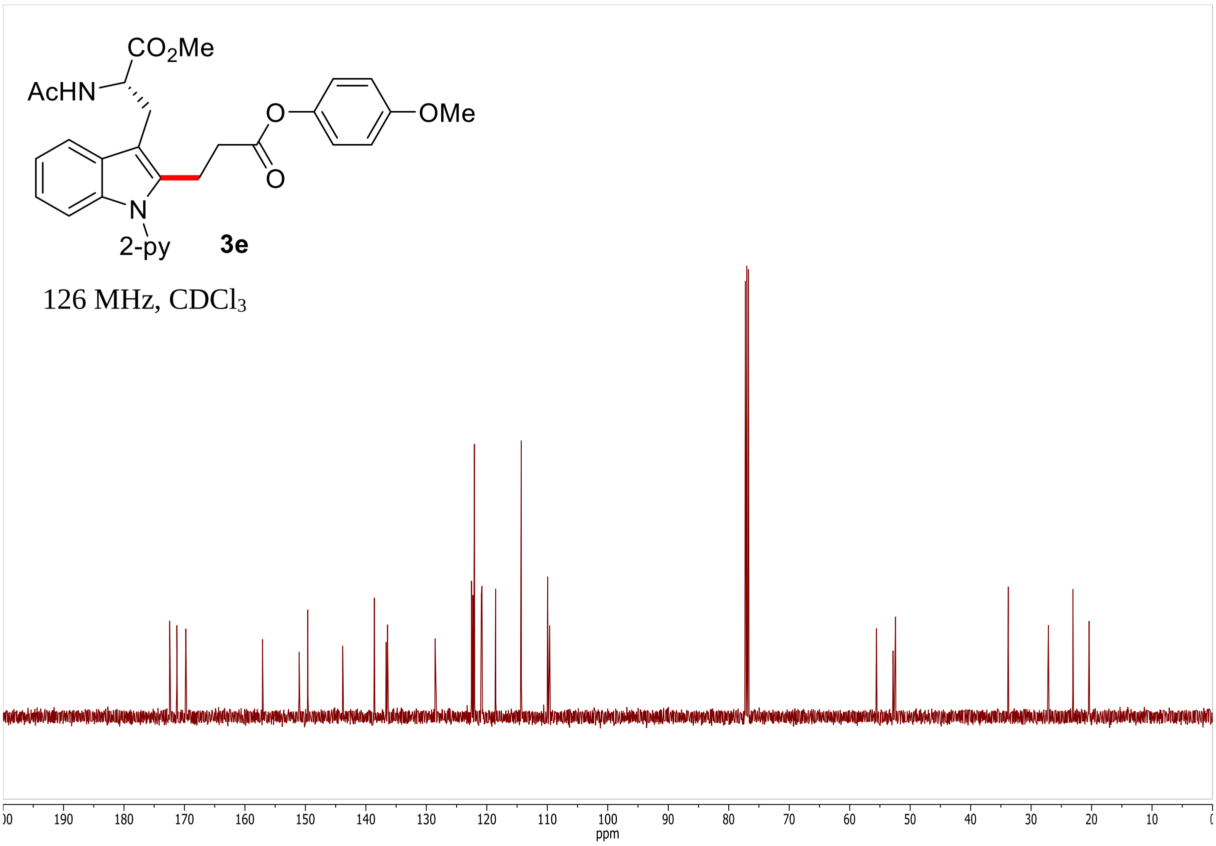

Supplementary Figure 29. ^{13}C-NMR spectrum of **3e**.

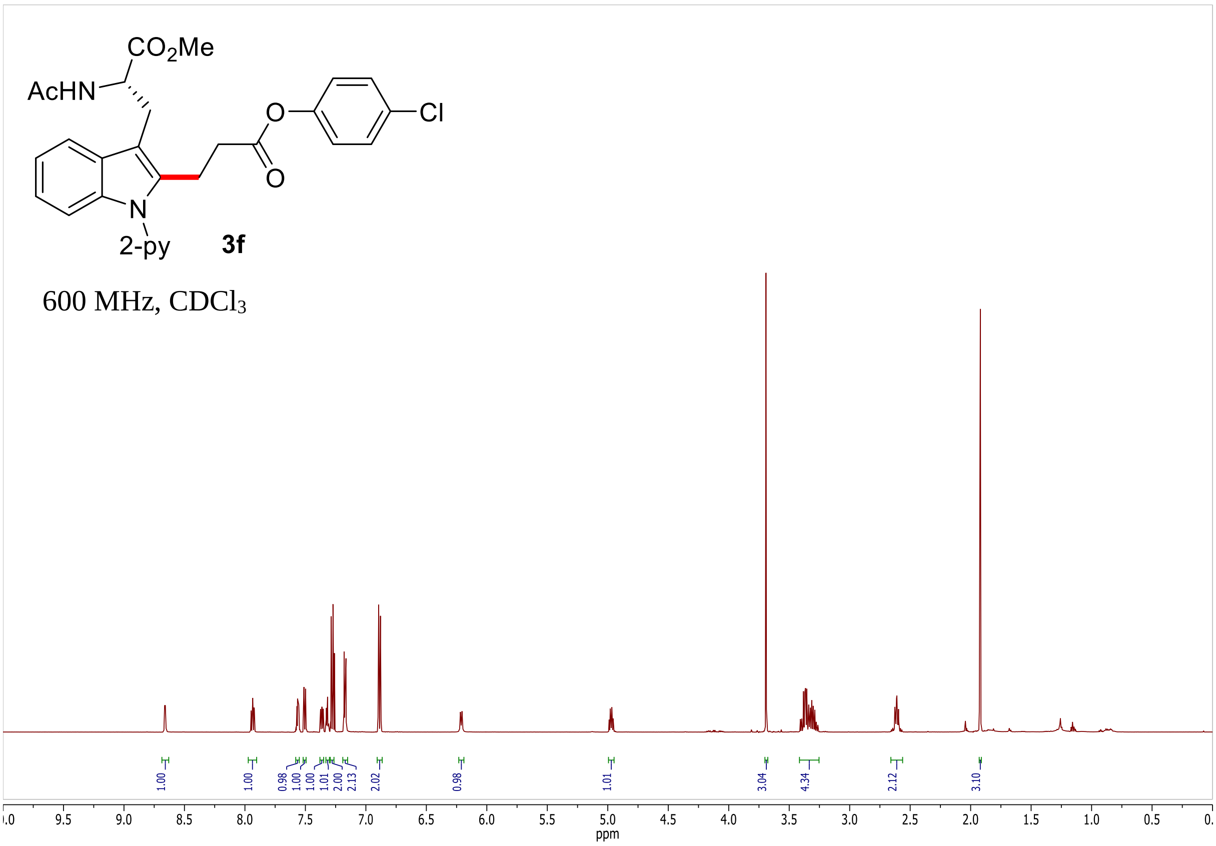

Supplementary Figure 30. ^{1}H-NMR spectrum of **3f**.

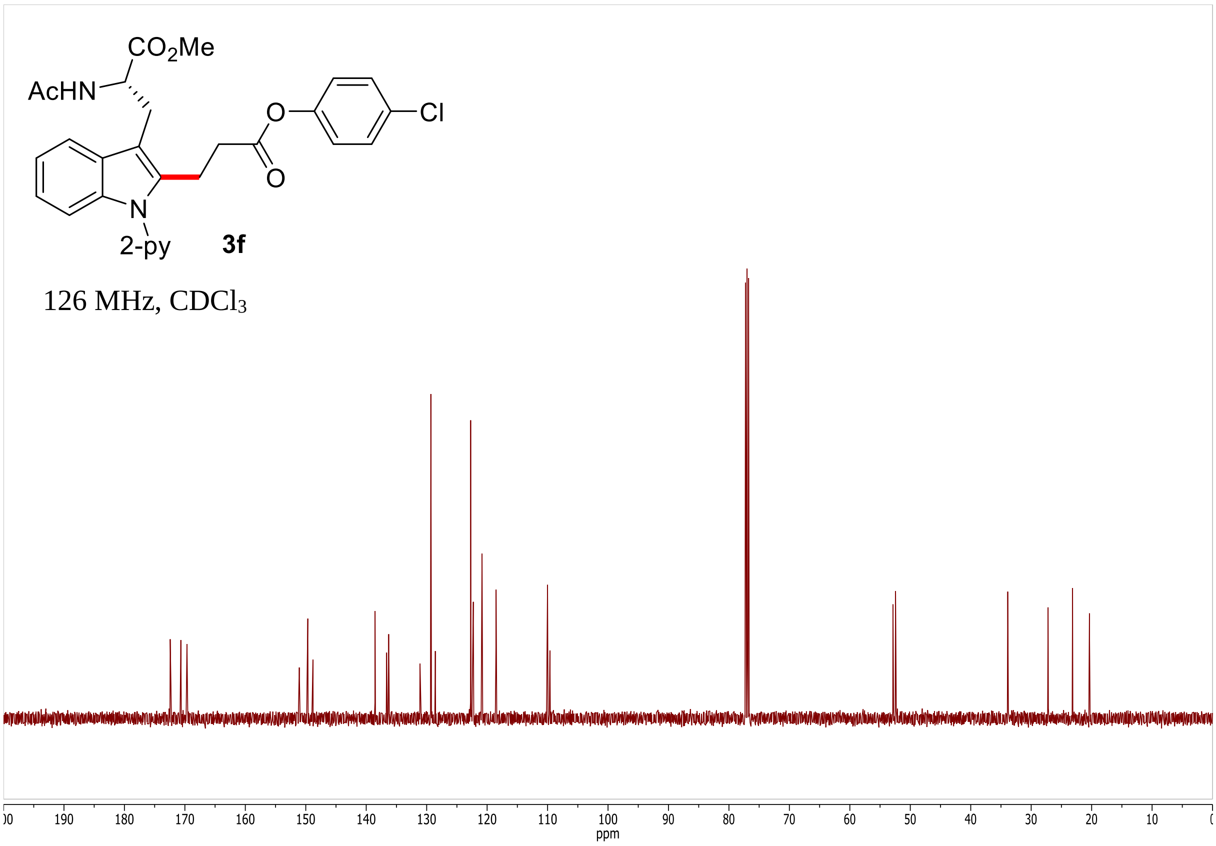

Supplementary Figure 31.^{13}C-NMR spectrum of **3f**.

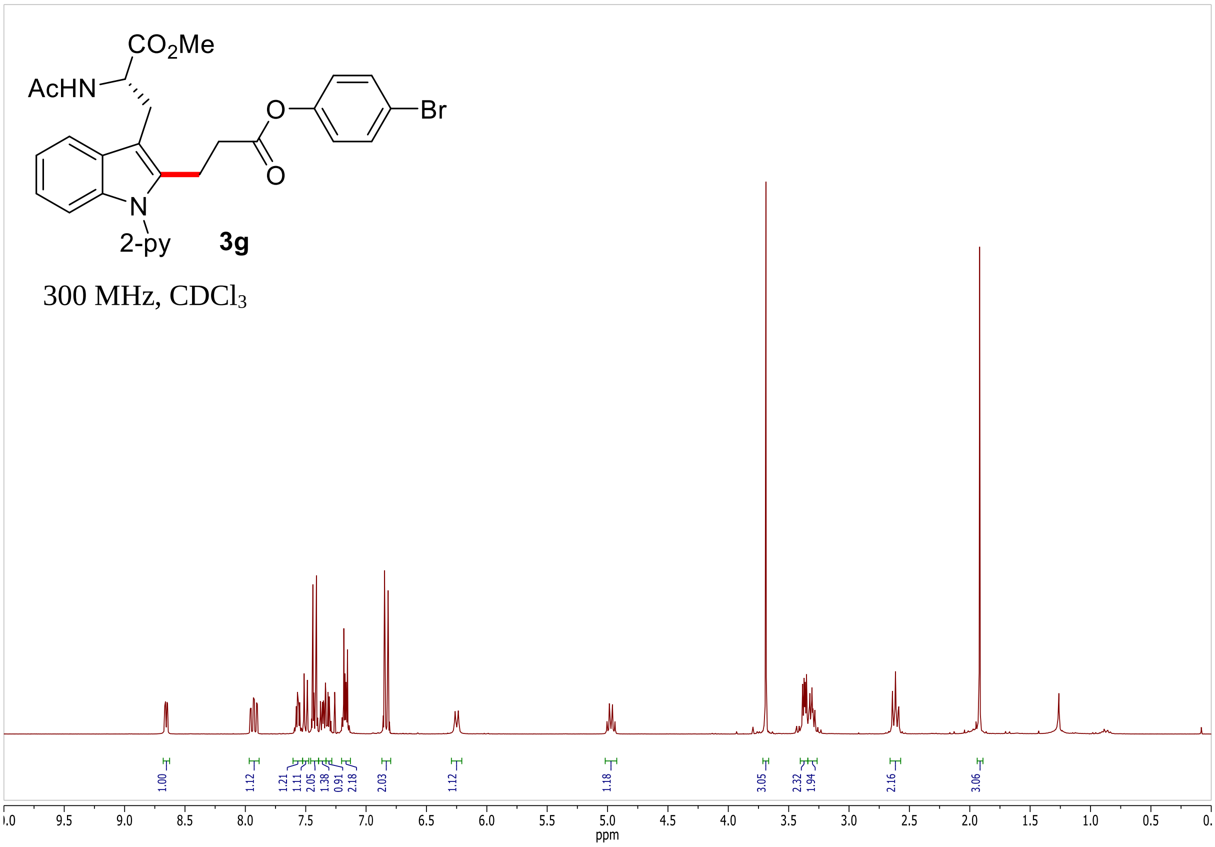

Supplementary Figure 32. ^{1}H-NMR spectrum of **3g**.

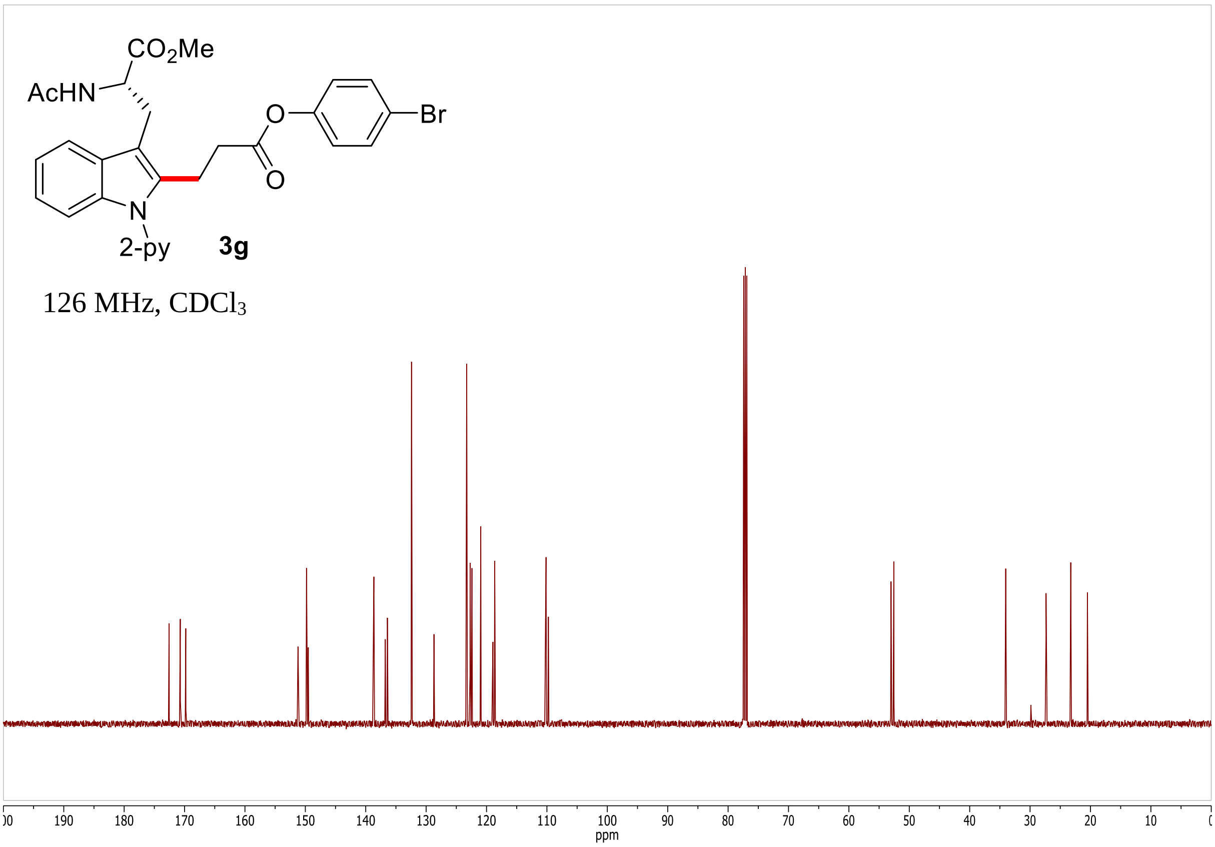

Supplementary Figure 33. ^{13}C-NMR spectrum of **3g**.

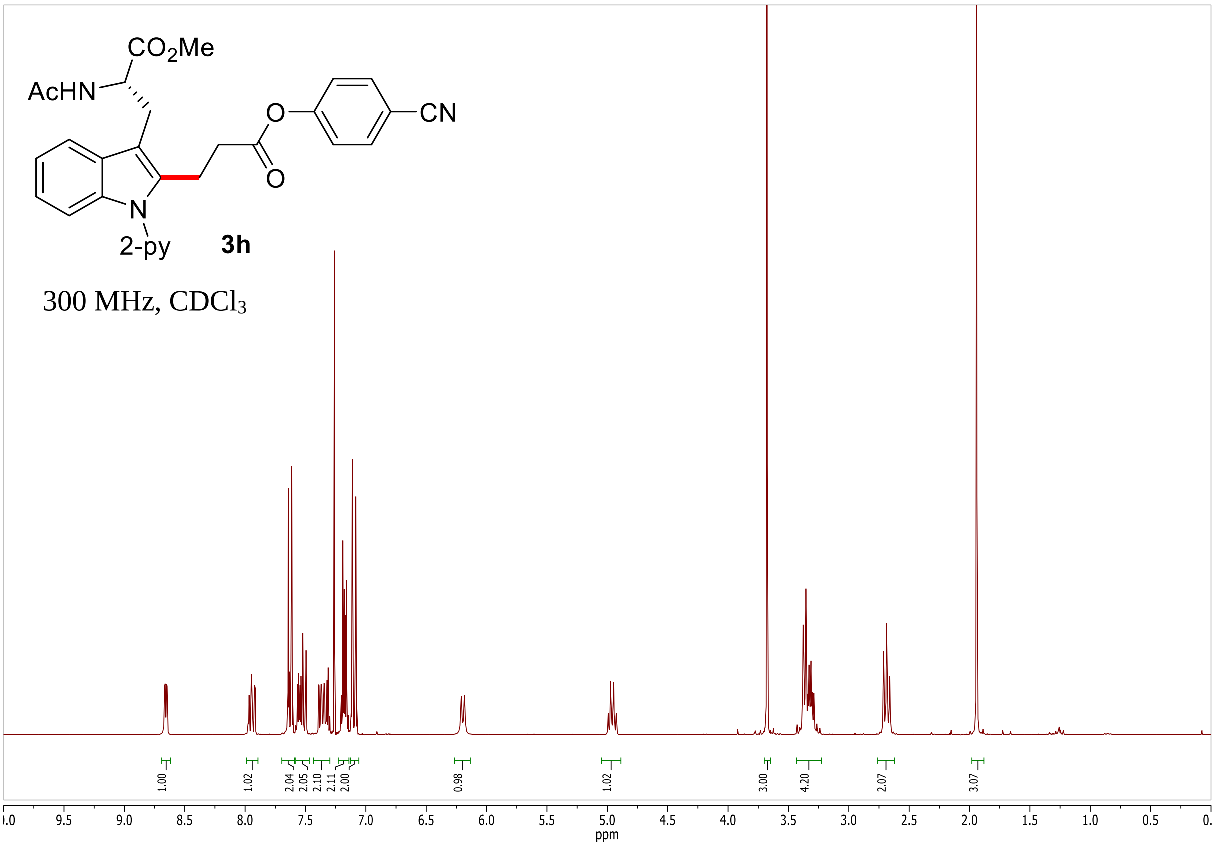

Supplementary Figure 34. ^{1}H-NMR spectrum of **3h**.

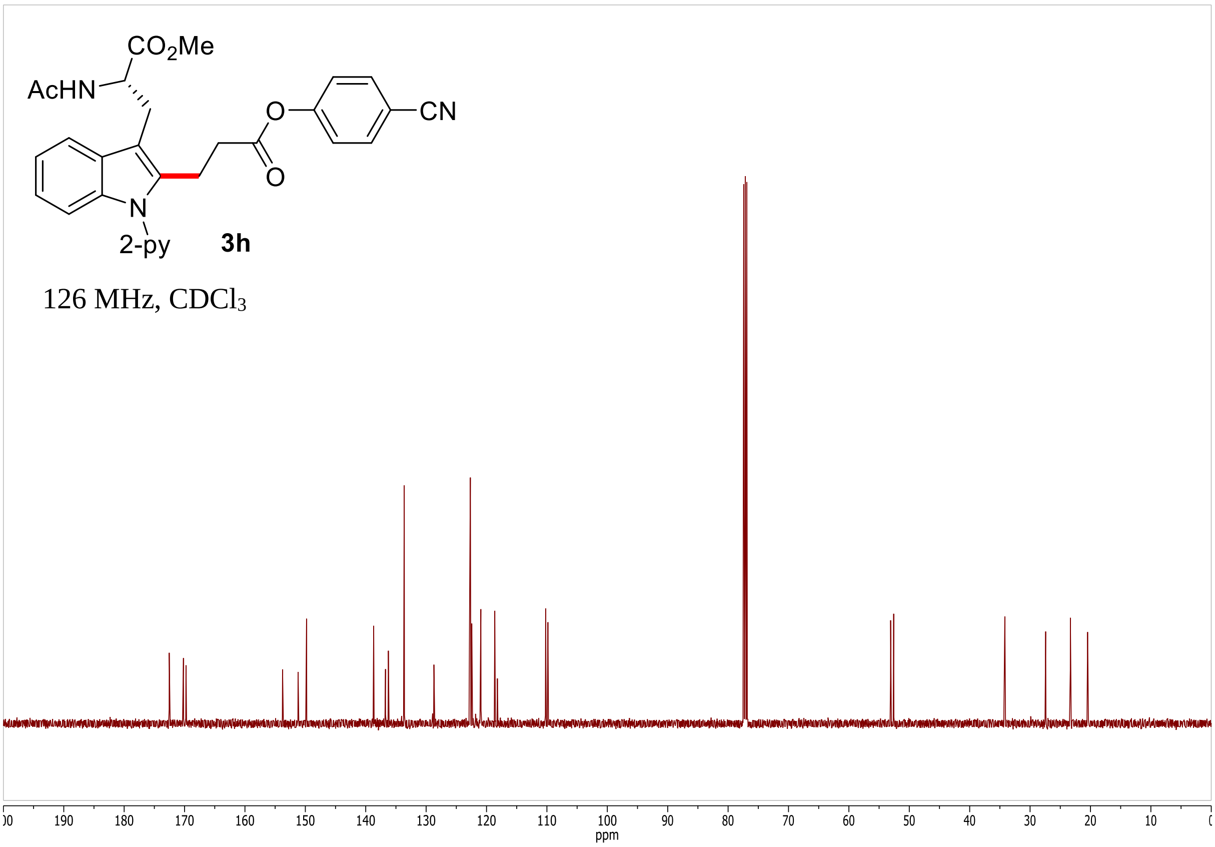

Supplementary Figure 35. ^{13}C-NMR spectrum of **3h**.

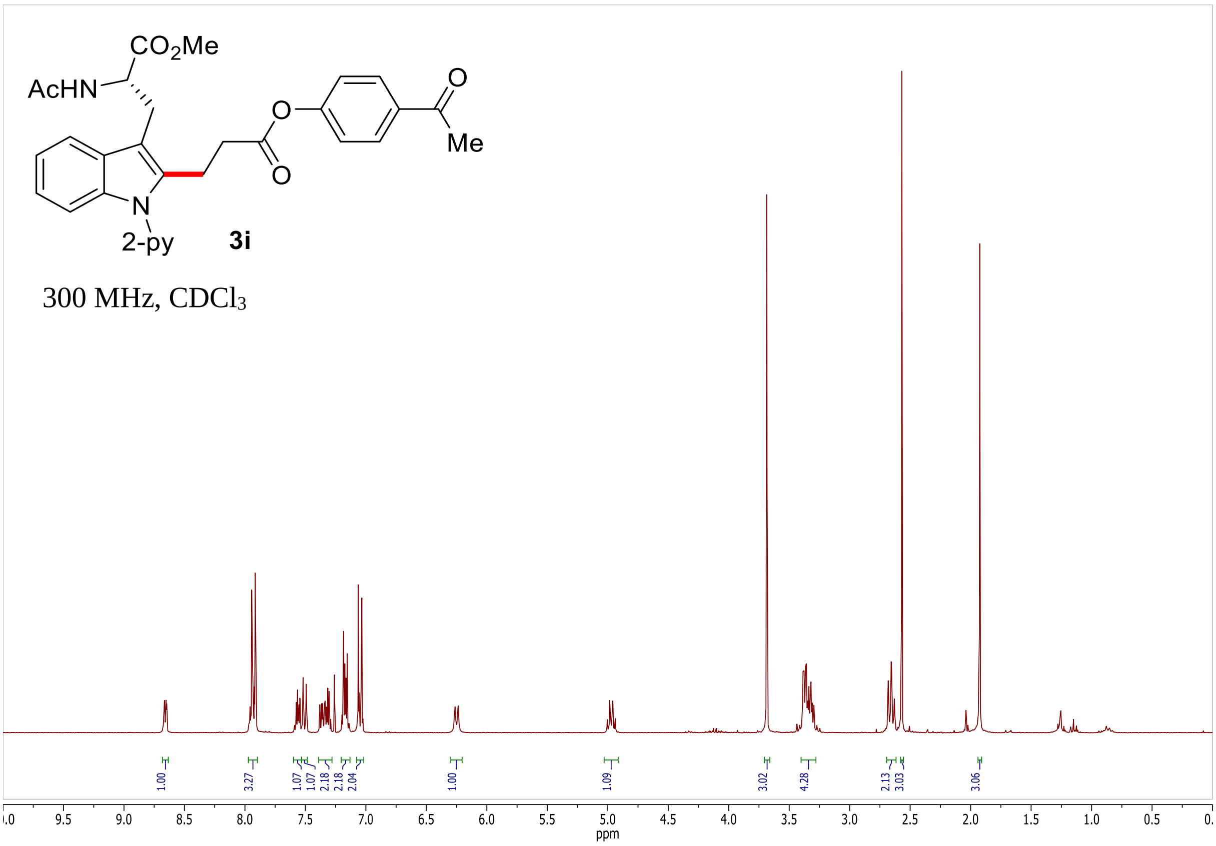

Supplementary Figure 36. ^{1}H-NMR spectrum of **3i**.

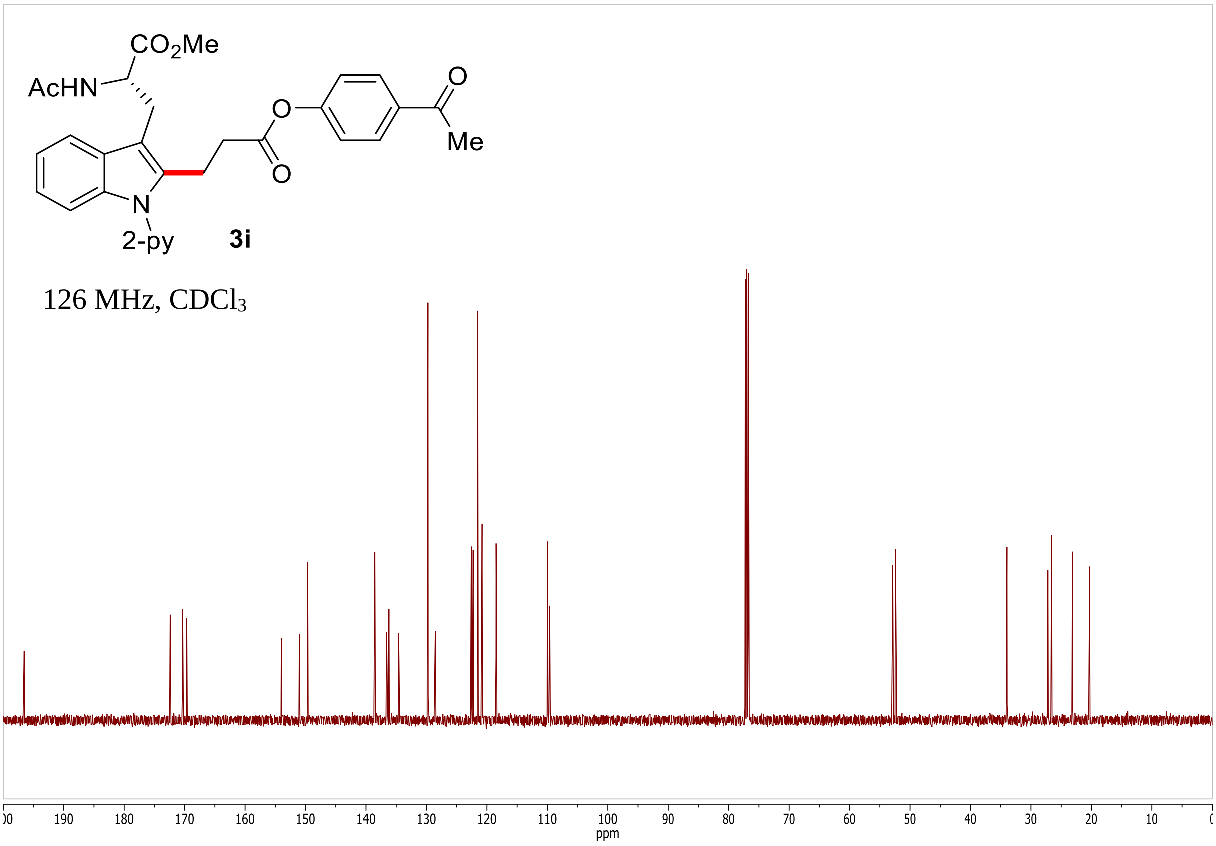

Supplementary Figure 37. ^{13}C-NMR spectrum of **3i**.

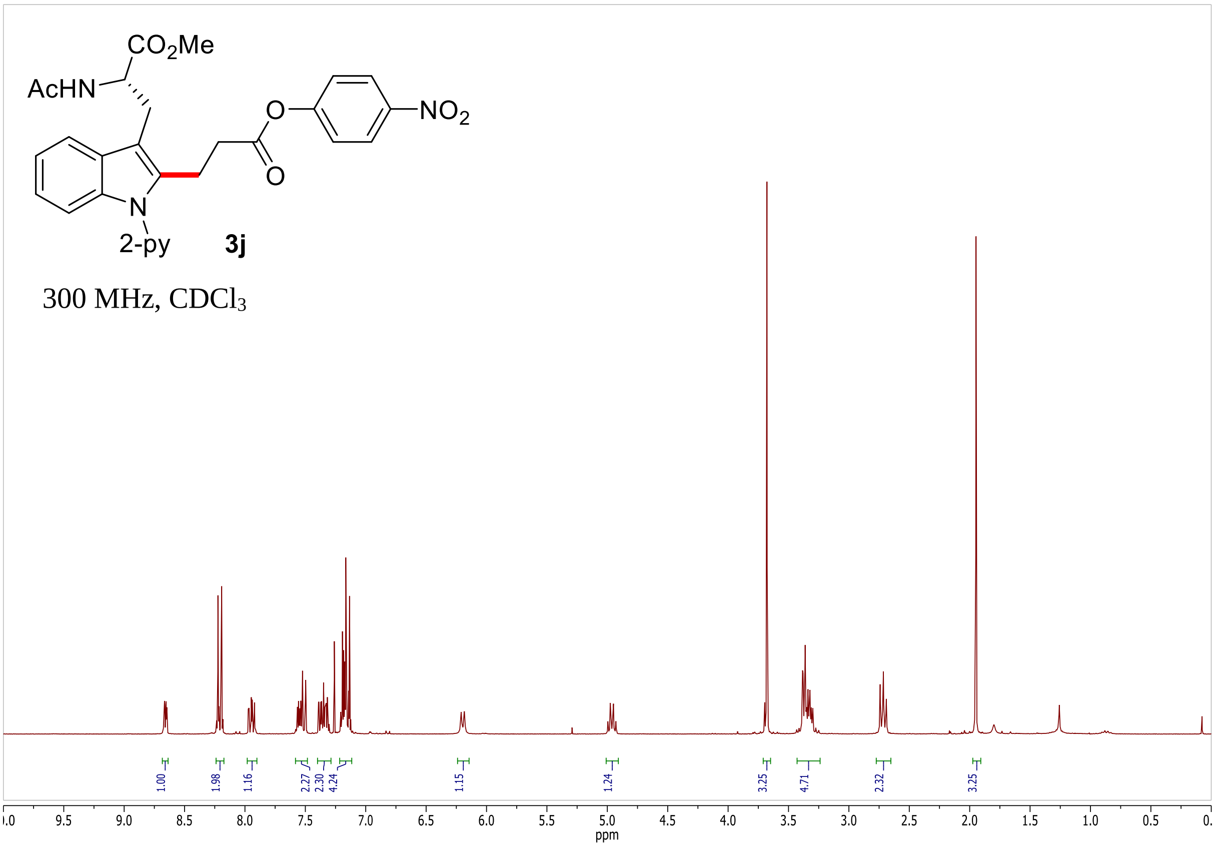

Supplementary Figure 38. ^{1}H-NMR spectrum of **3j**.

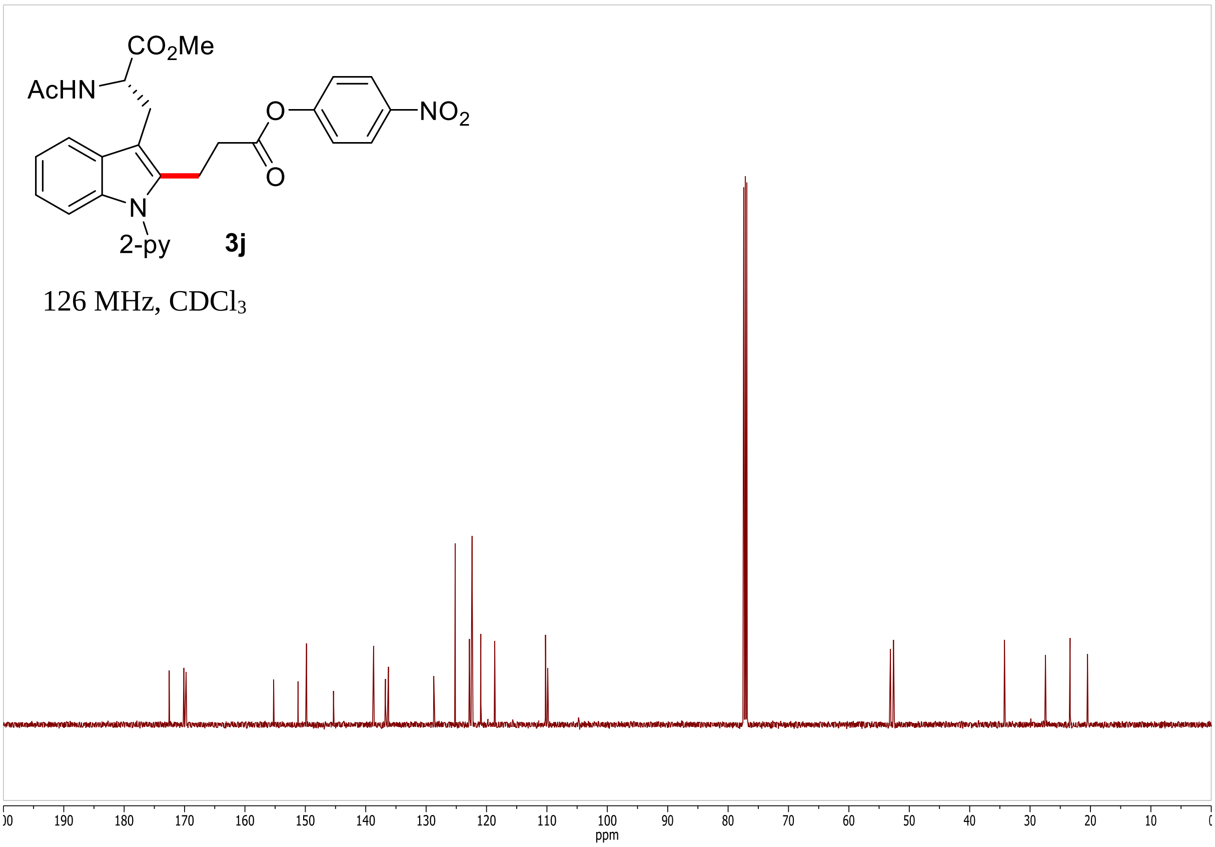

Supplementary Figure 39. ^{13}C-NMR spectrum of **3j**.

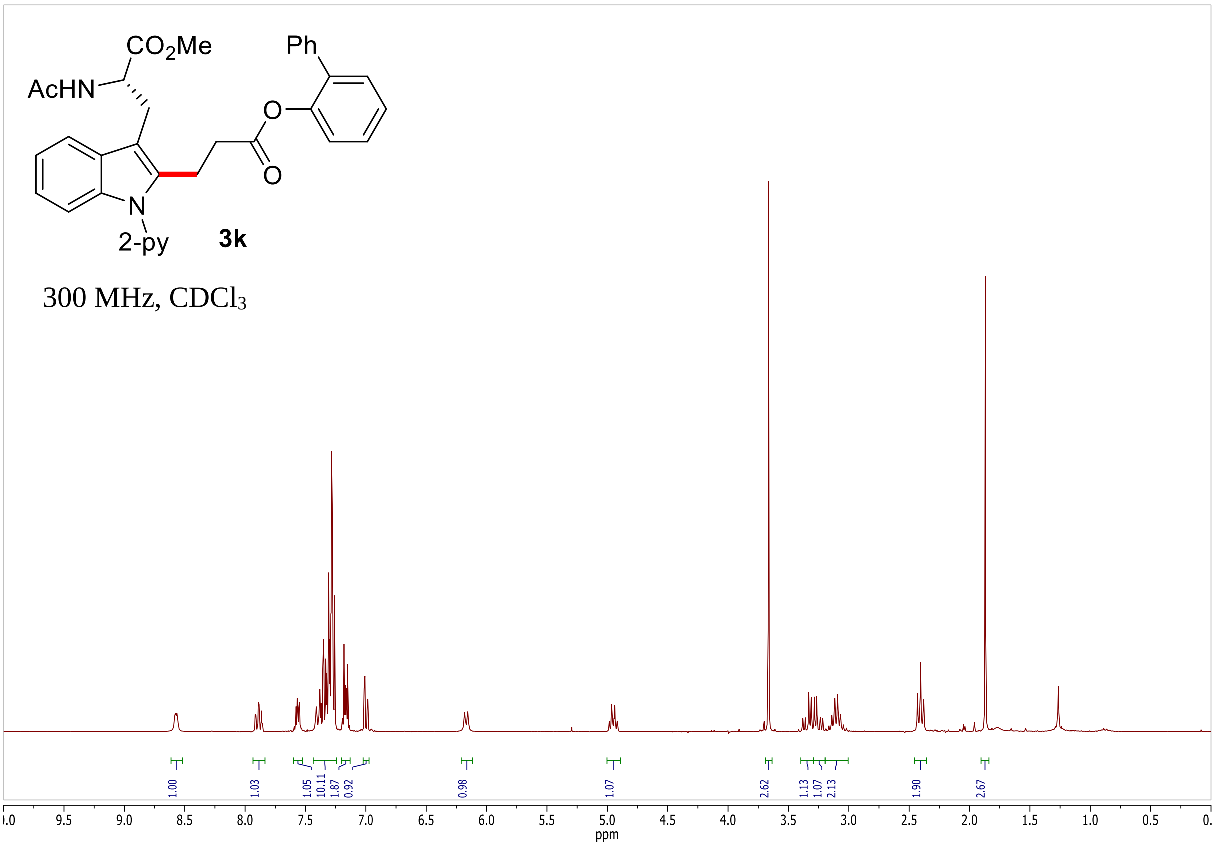

Supplementary Figure 40. ^{1}H-NMR spectrum of **3k**.

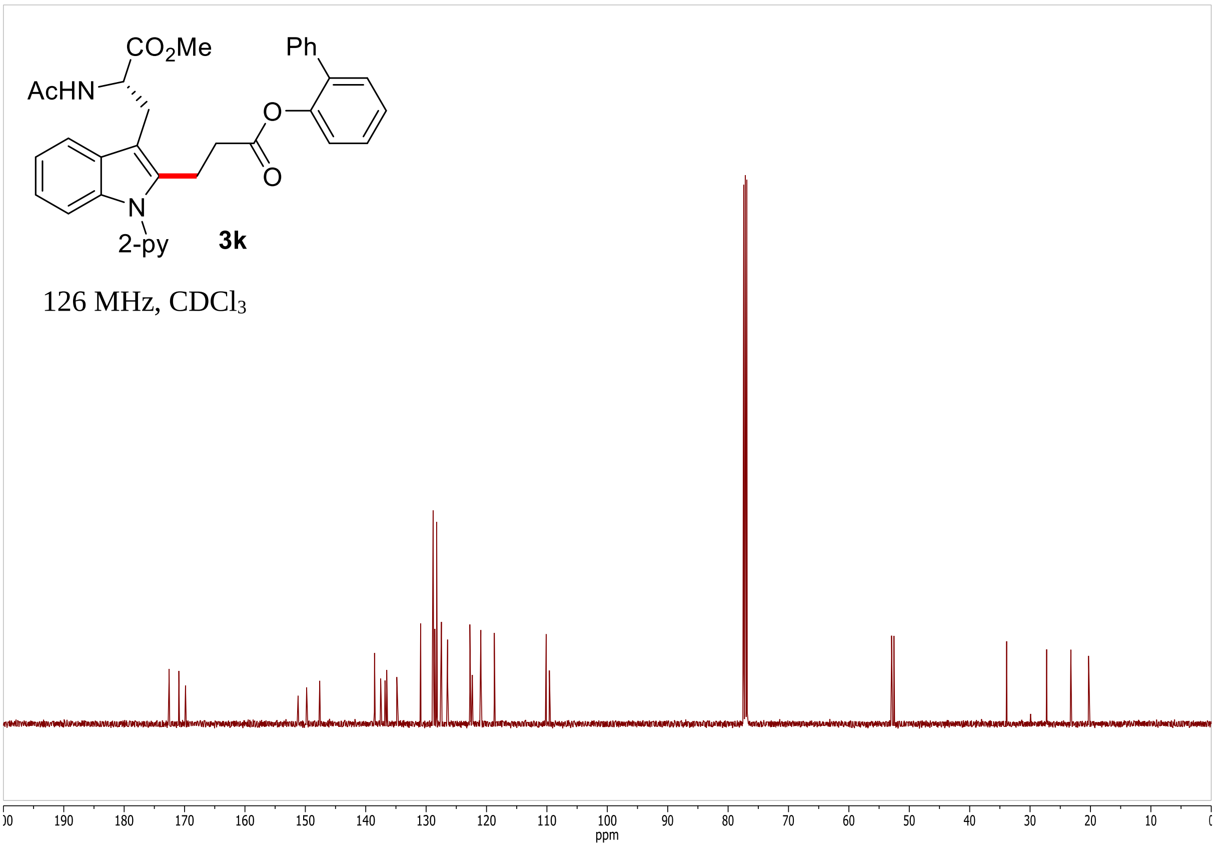

Supplementary Figure 41. ^{13}C-NMR spectrum of **3k**.

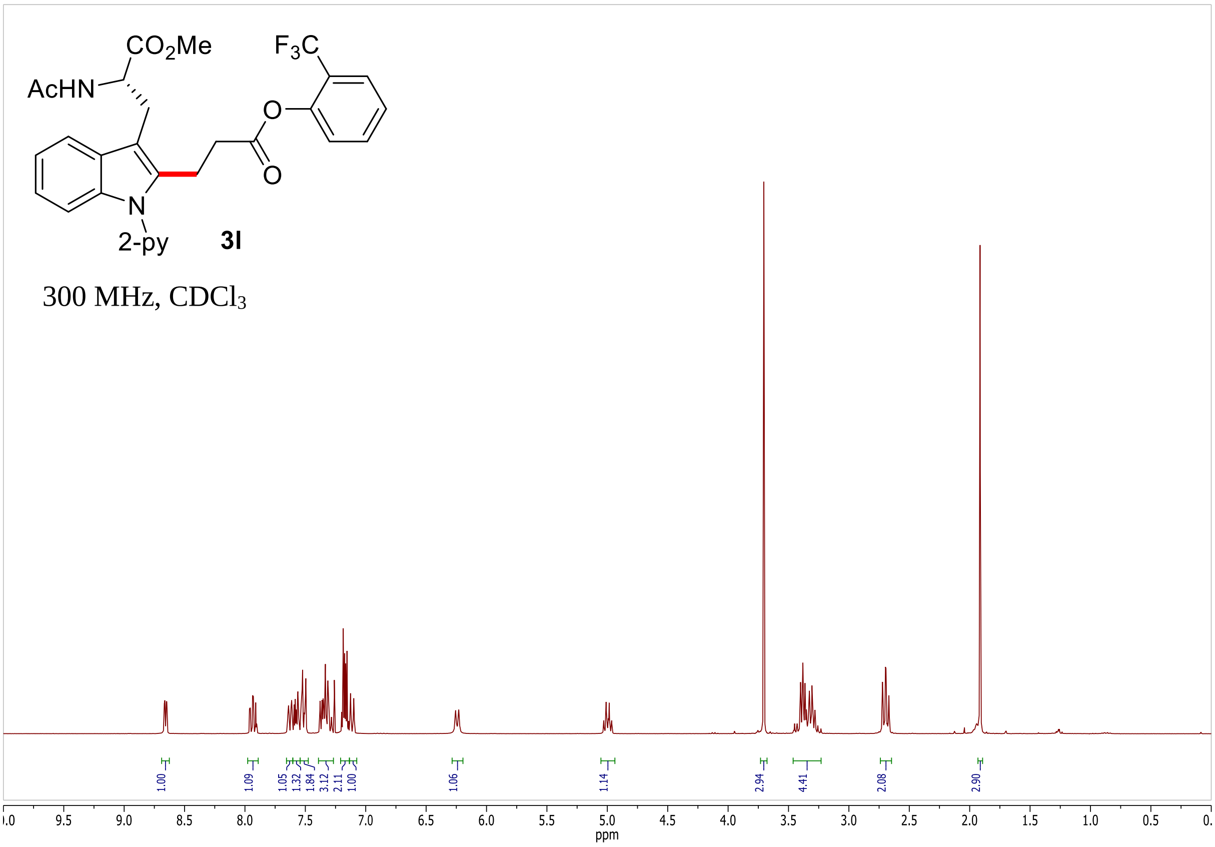

Supplementary Figure 42. ^{1}H-NMR spectrum of **3l**.

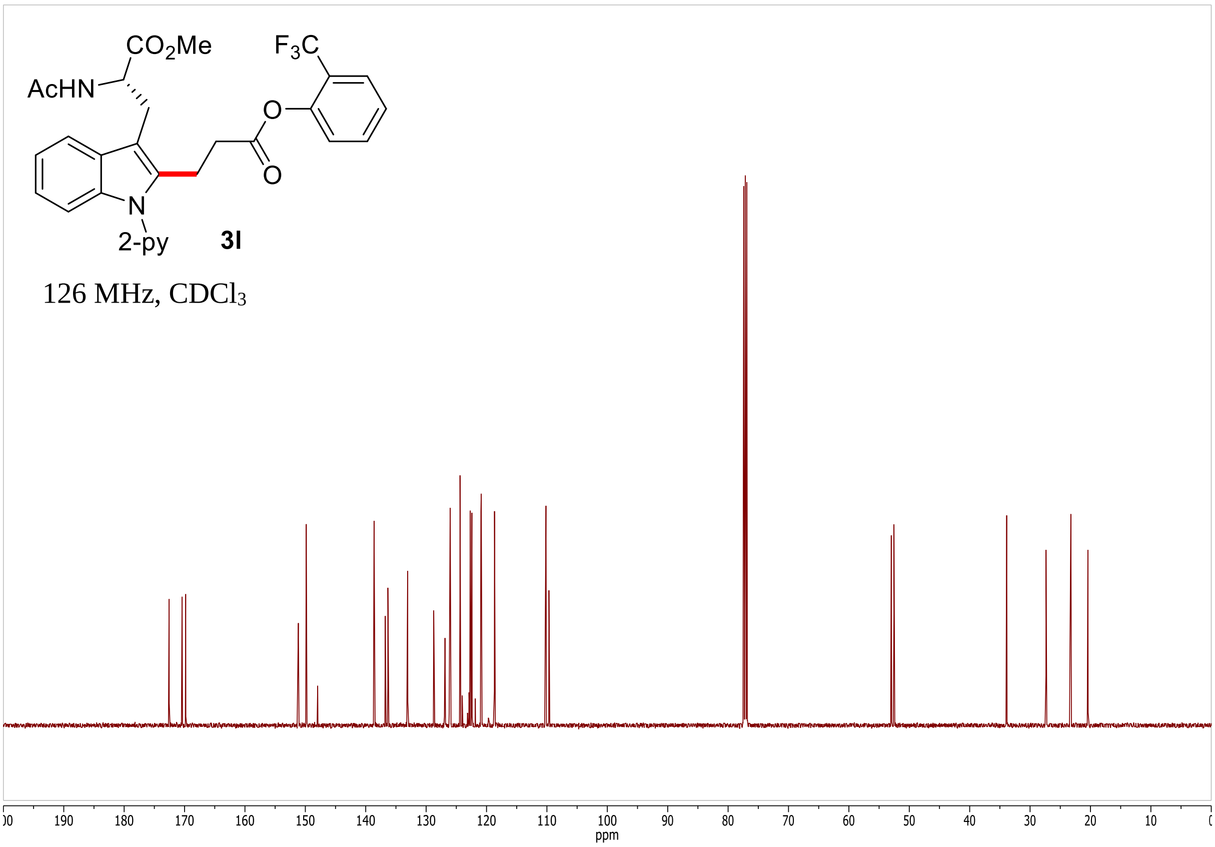

Supplementary Figure 43. ^{13}C-NMR spectrum of **3l**.

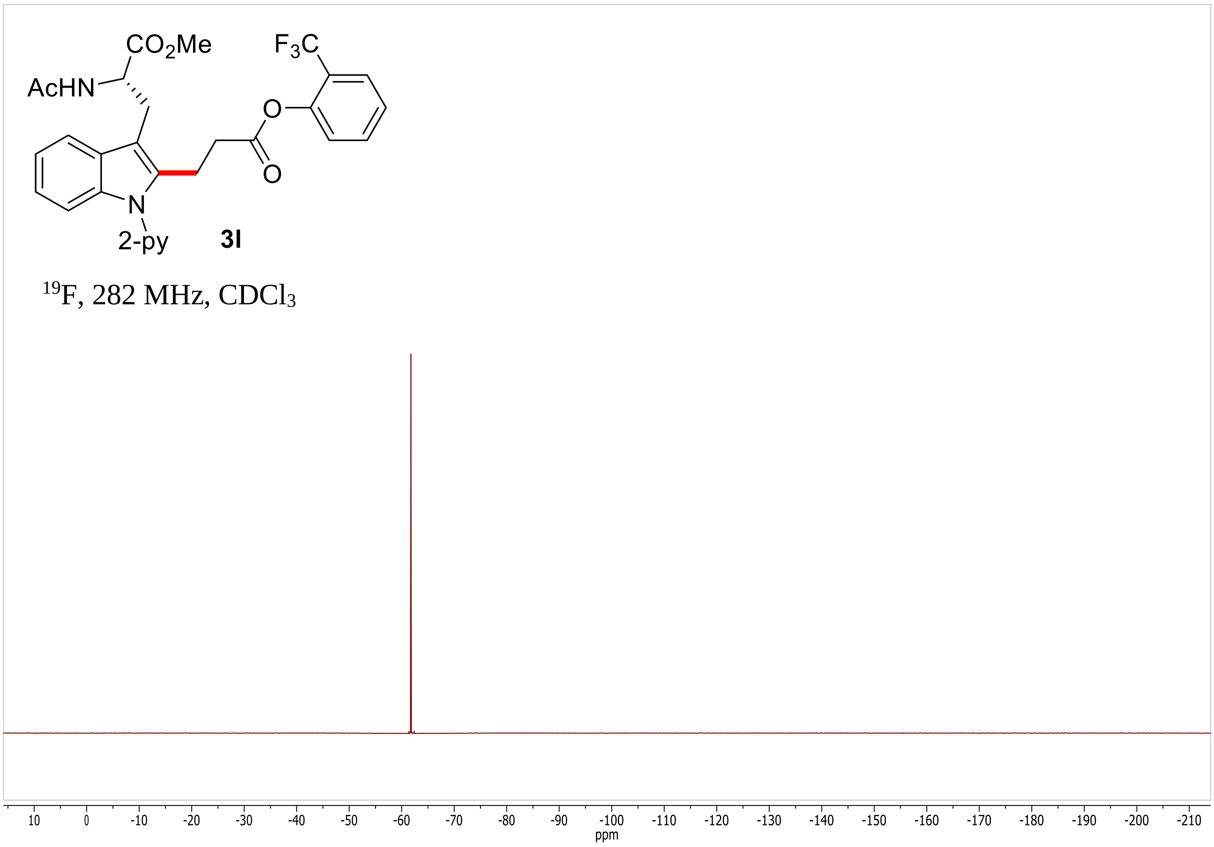

Supplementary Figure 44. ^{19}F-NMR spectrum of **3l**.

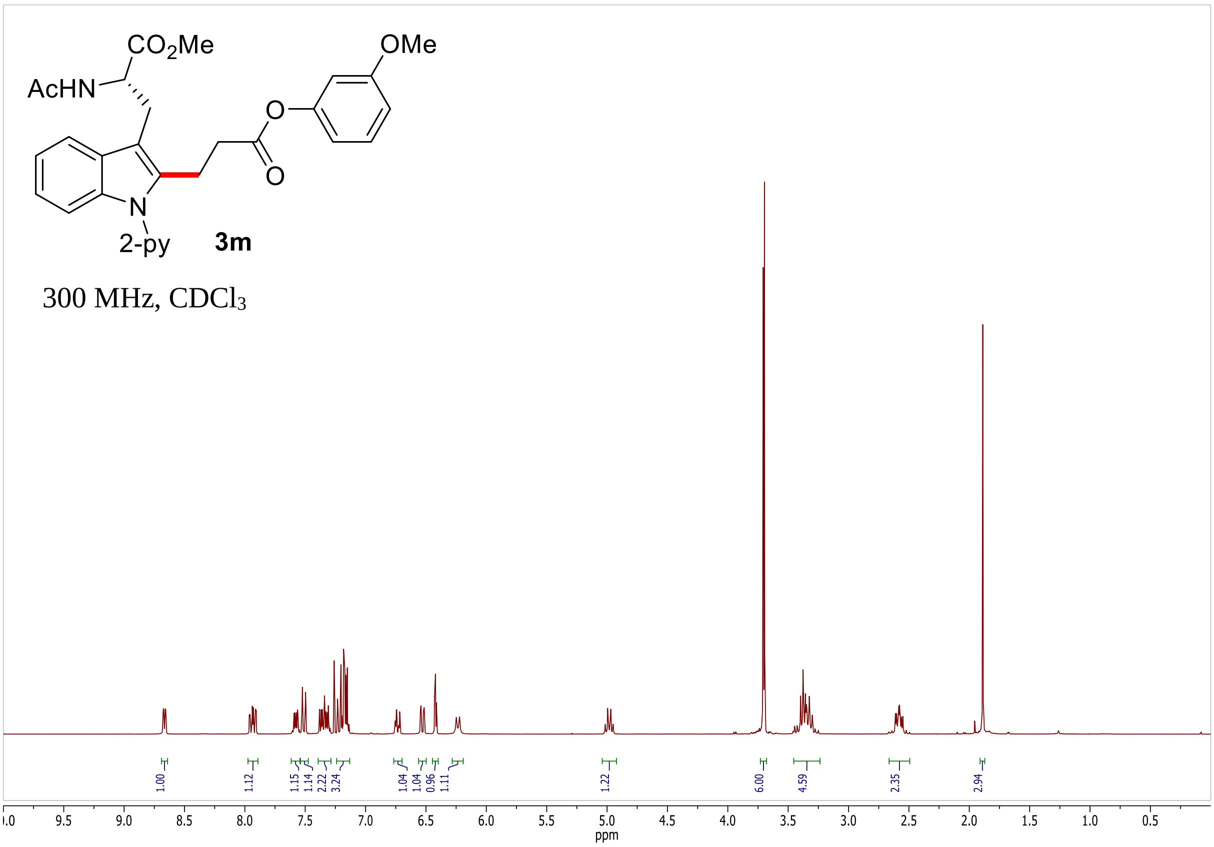

Supplementary Figure 45. ^{1}H-NMR spectrum of **3m**.

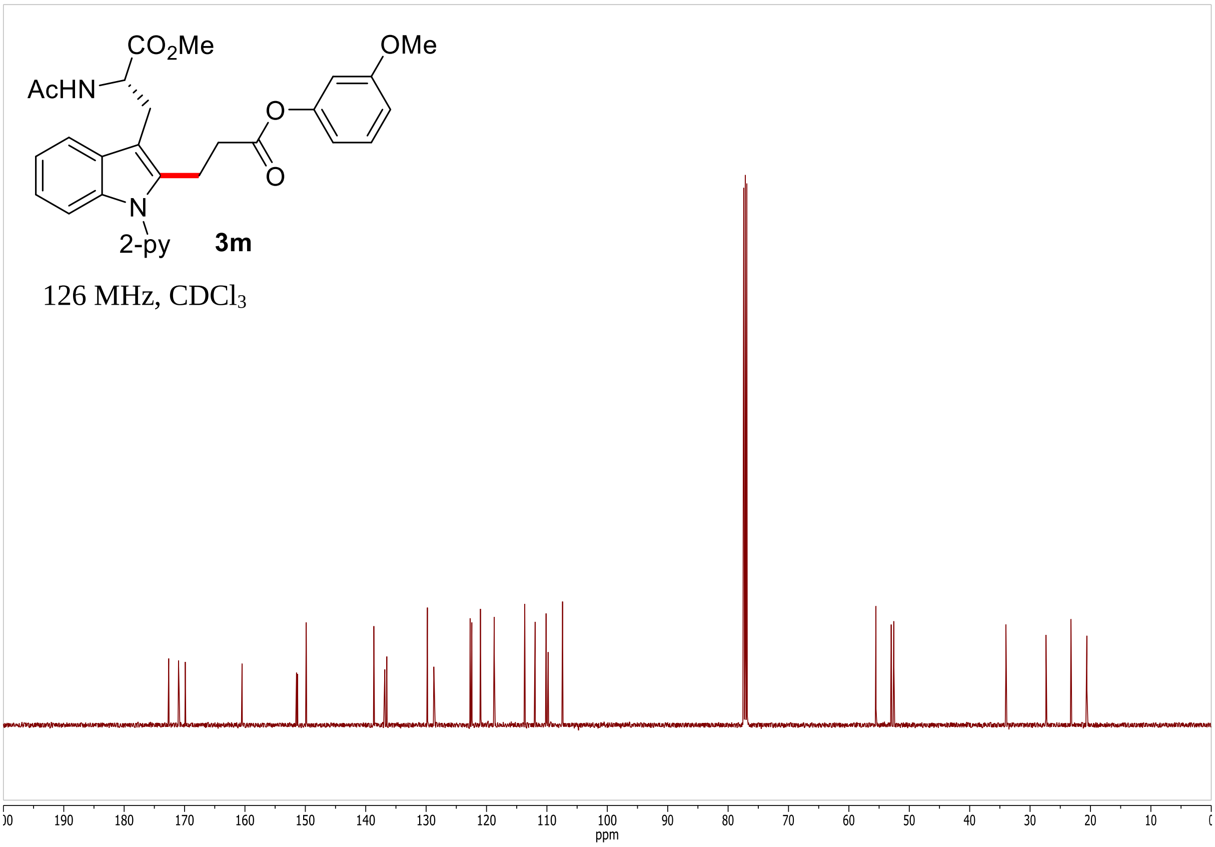

Supplementary Figure 46. ^{13}C-NMR spectrum of **3m**.

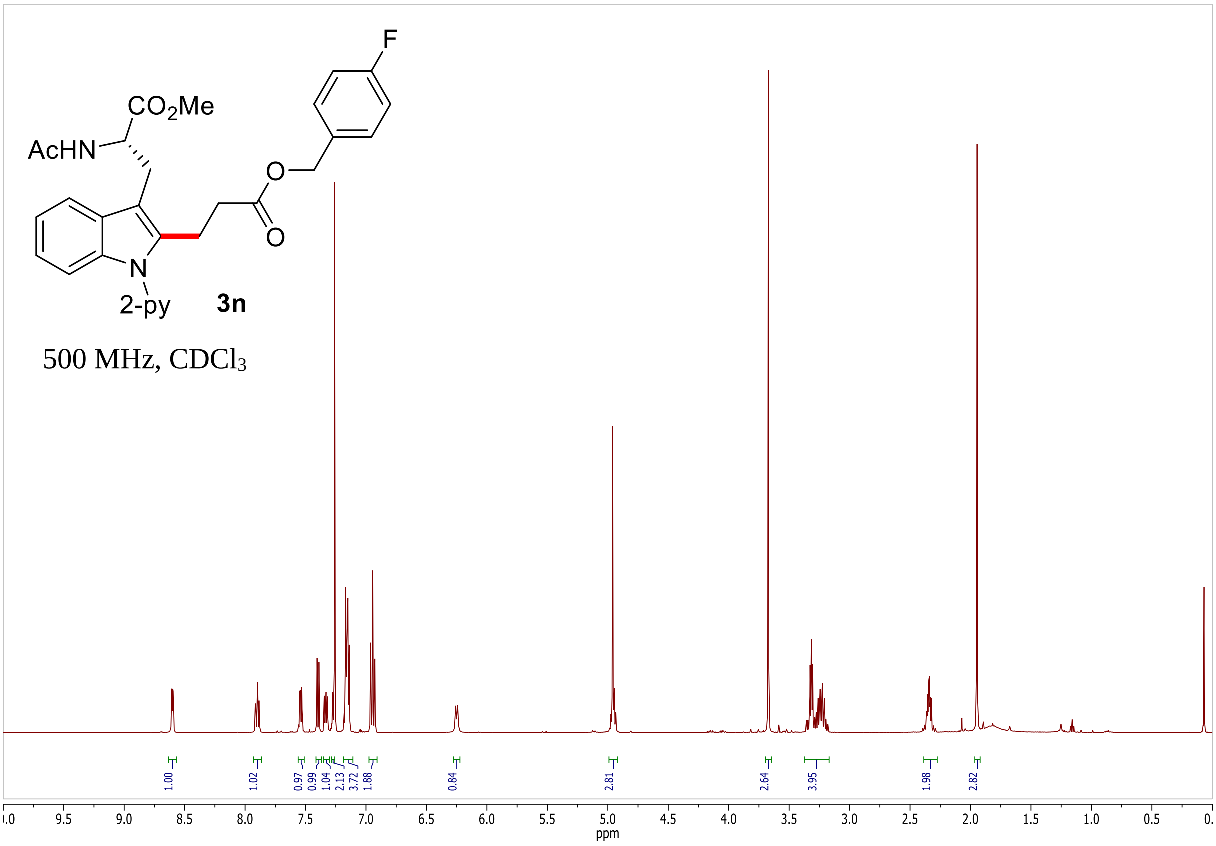

Supplementary Figure 47. ^{1}H-NMR spectrum of **3n**.

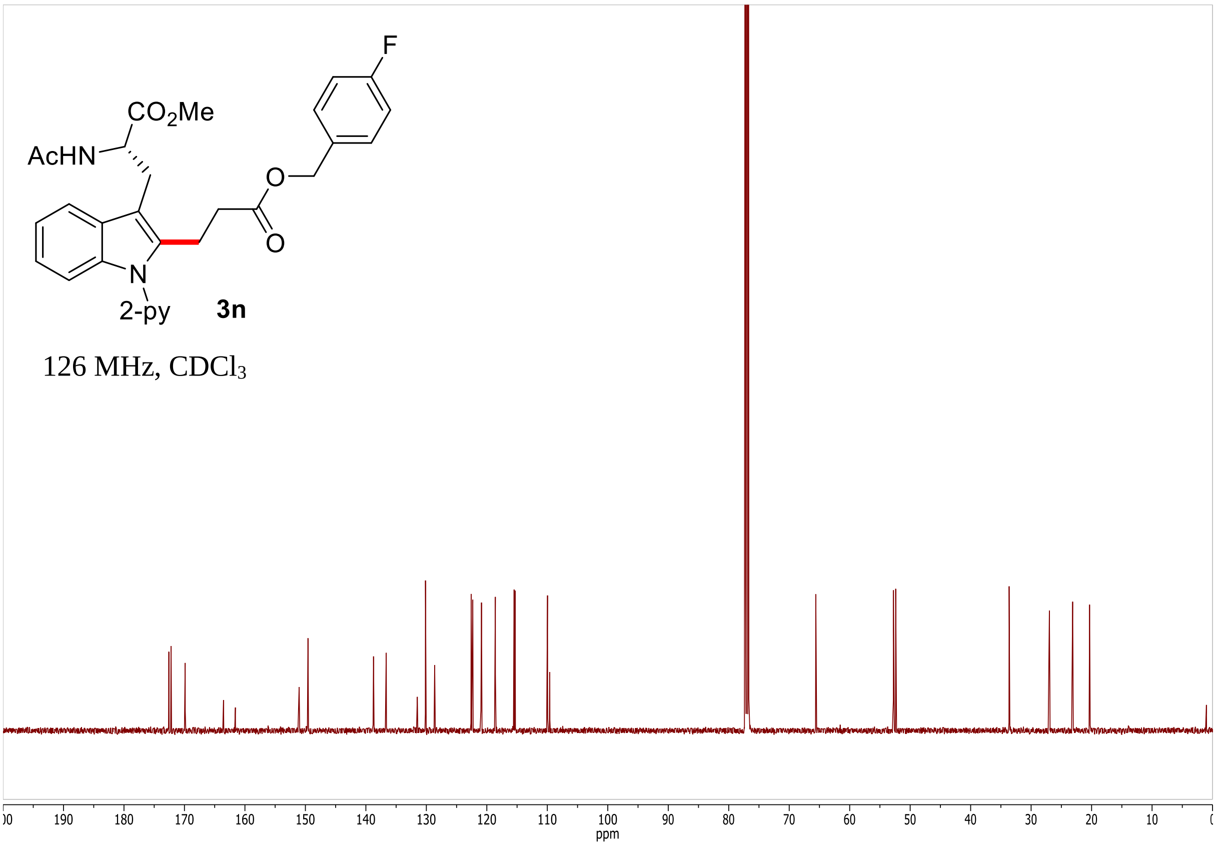

Supplementary Figure 48. ^{13}C-NMR spectrum of **3n**.

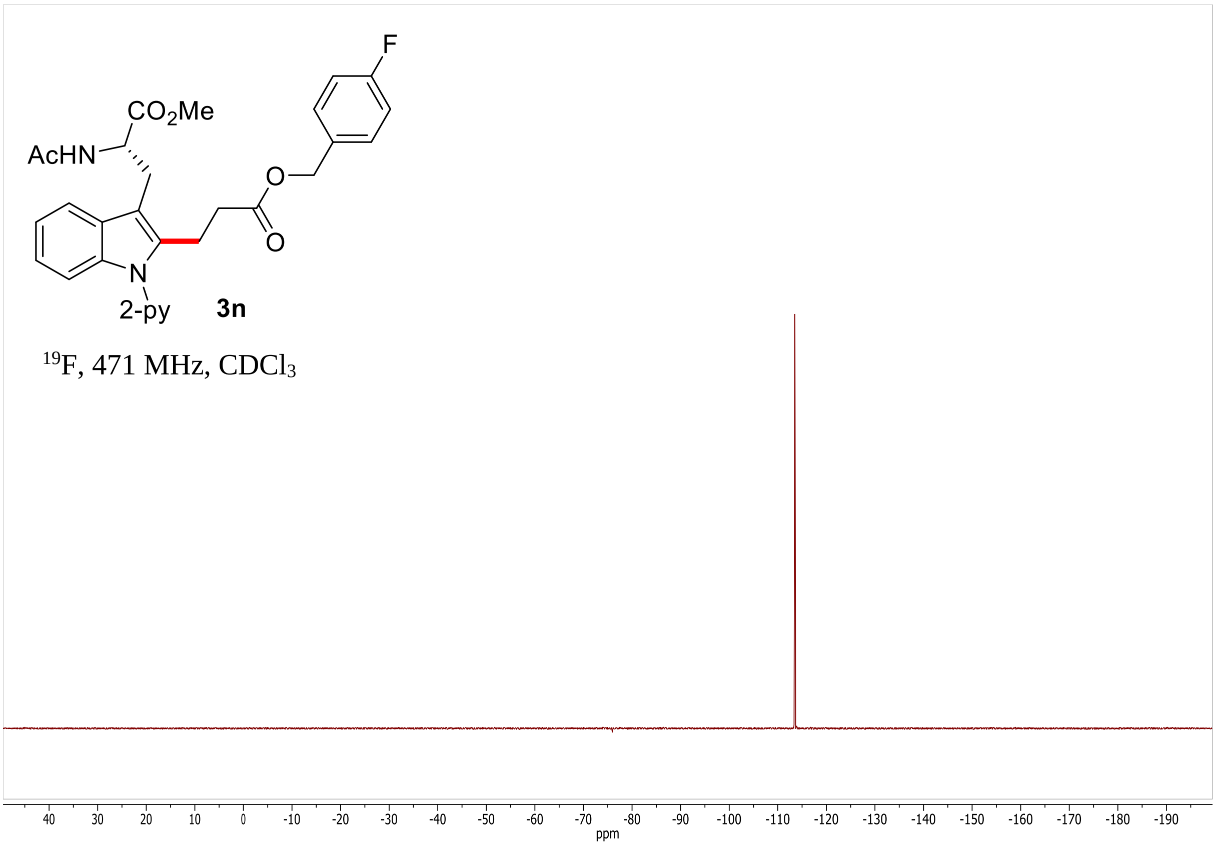

Supplementary Figure 49. ^{19}F-NMR spectrum of **3n**.

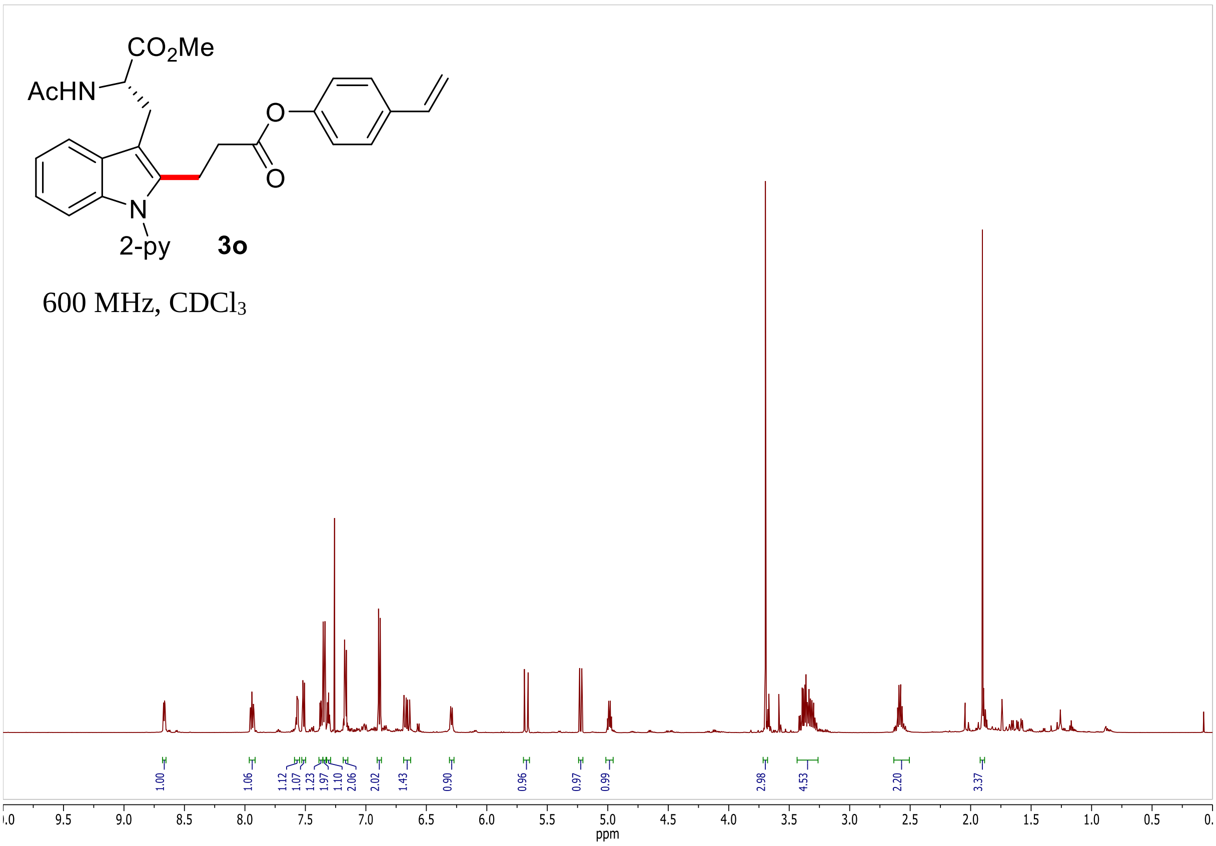

Supplementary Figure 50. ^{1}H-NMR spectrum of **3o**.

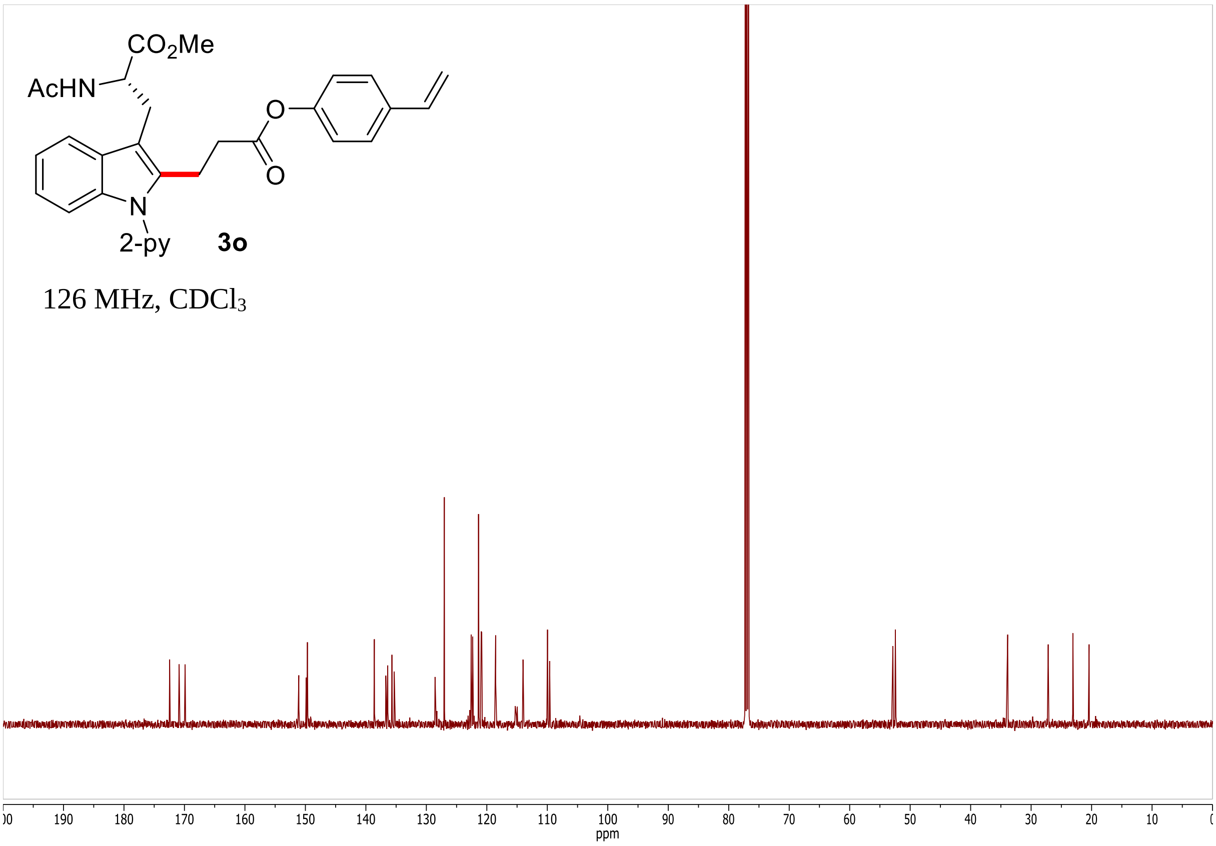

Supplementary Figure 51. ^{13}C-NMR spectrum of **3o**.

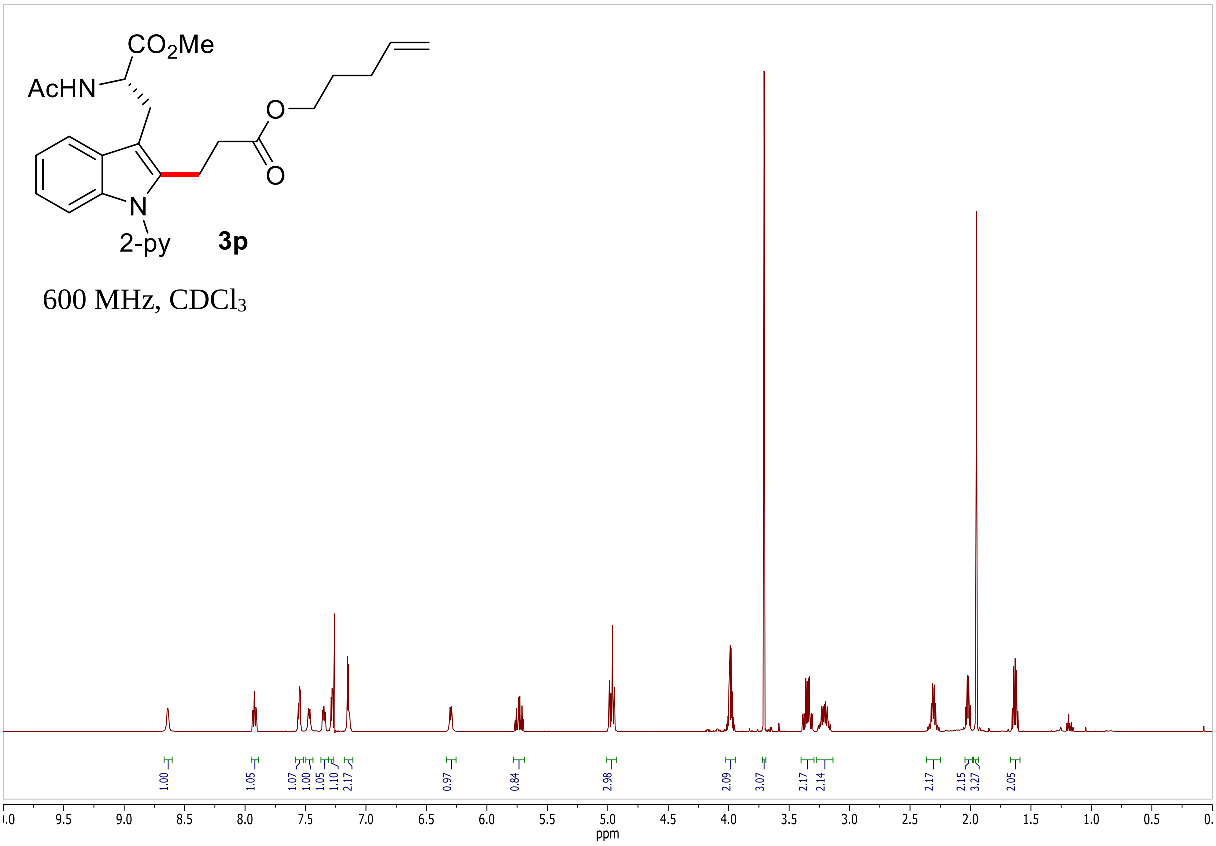

Supplementary Figure 52. ^{1}H-NMR spectrum of **3p**.

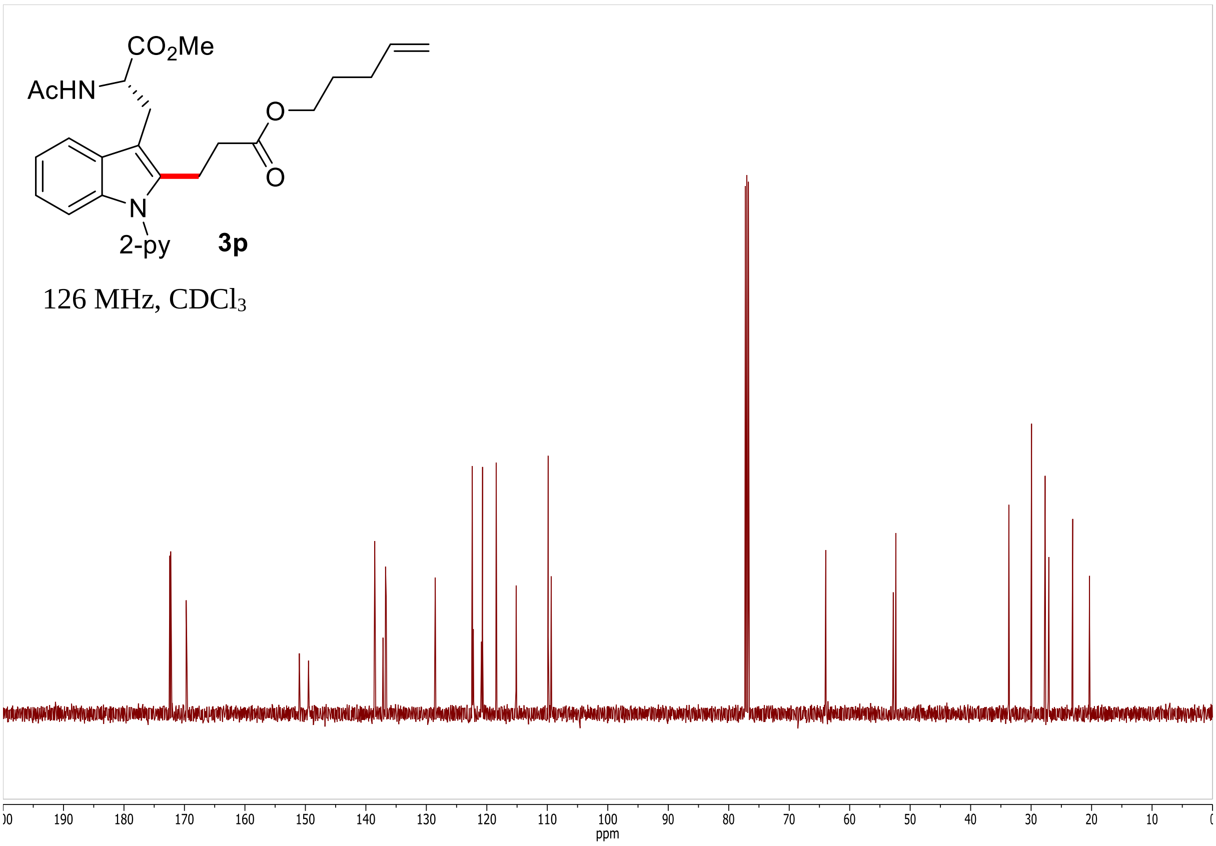

Supplementary Figure 53. ^{13}C-NMR spectrum of **3p**.

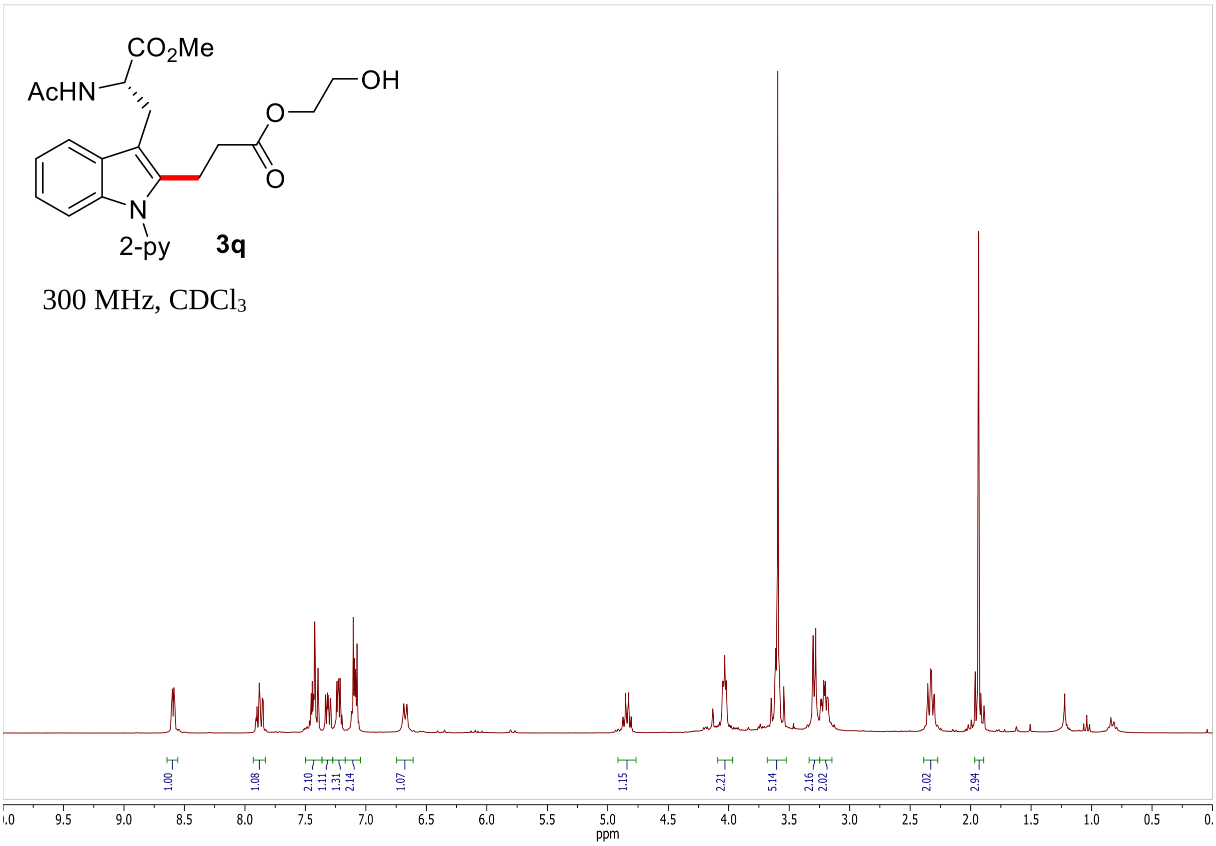

Supplementary Figure 54. ^{1}H-NMR spectrum of **3q**.

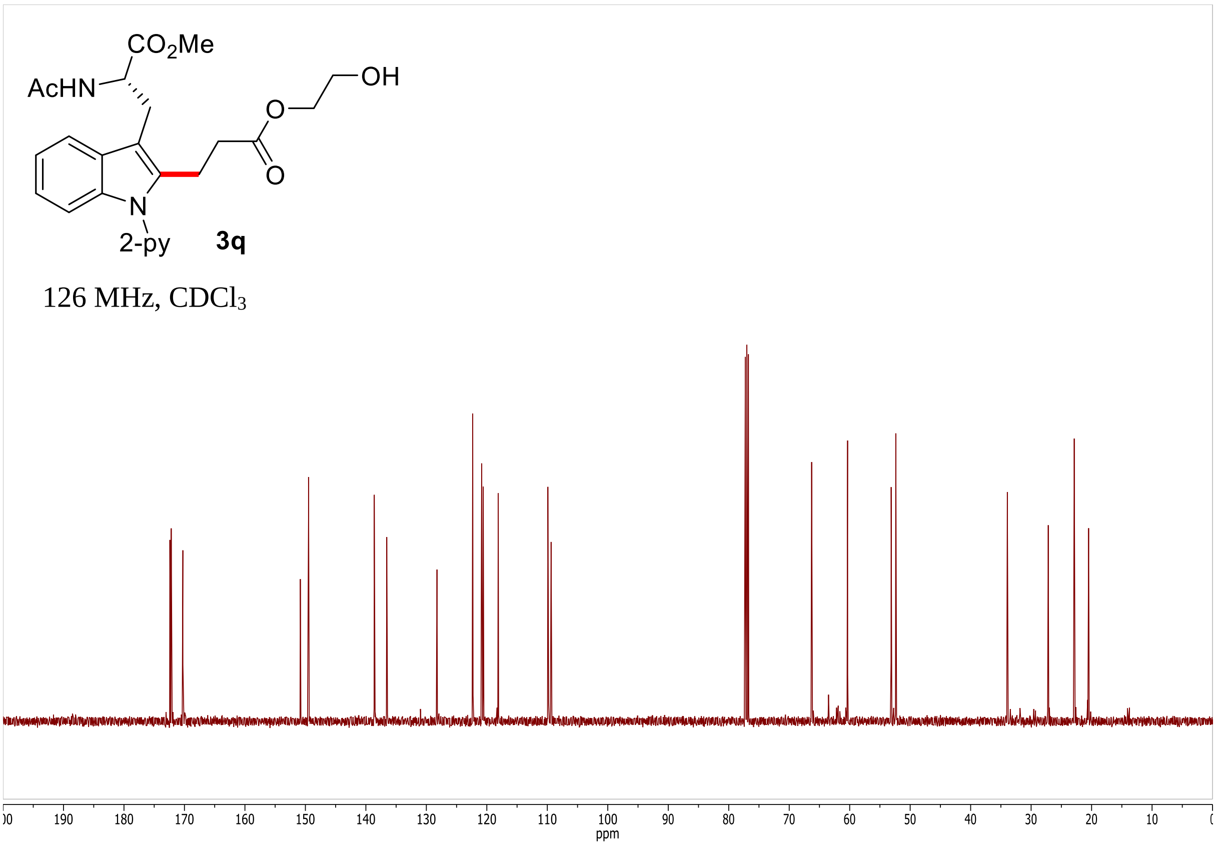

Supplementary Figure 55. ^{13}C-NMR spectrum of **3q**.

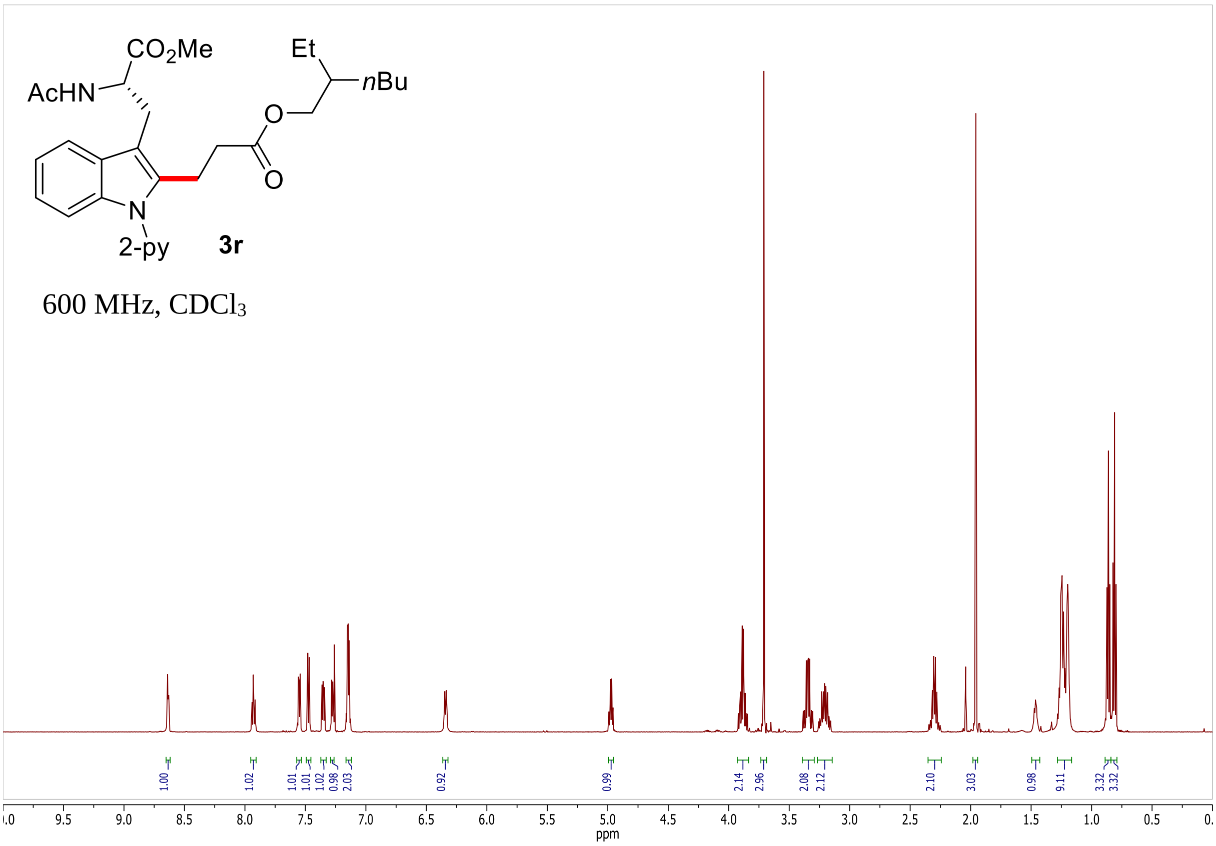

Supplementary Figure 56. ^{1}H-NMR spectrum of **3r**.

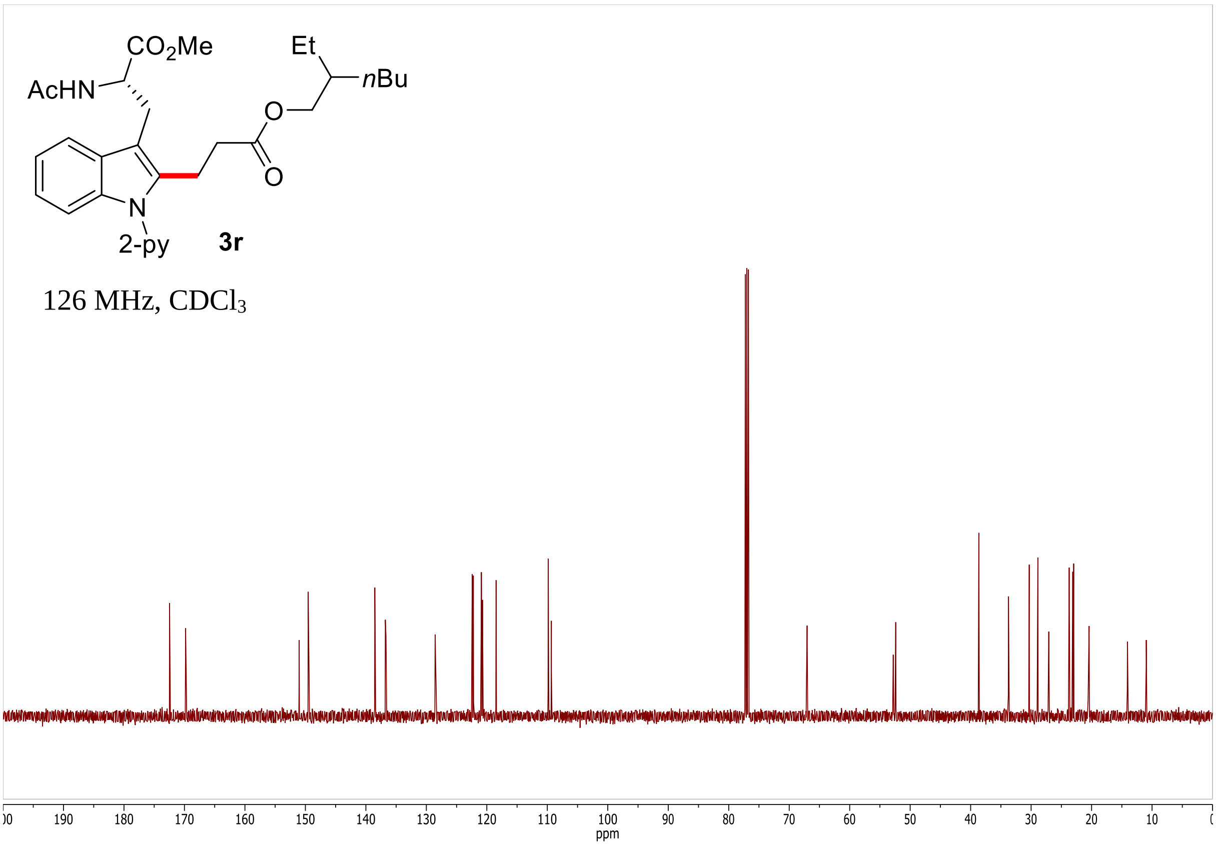

Supplementary Figure 57. ^{13}C-NMR spectrum of **3r**.

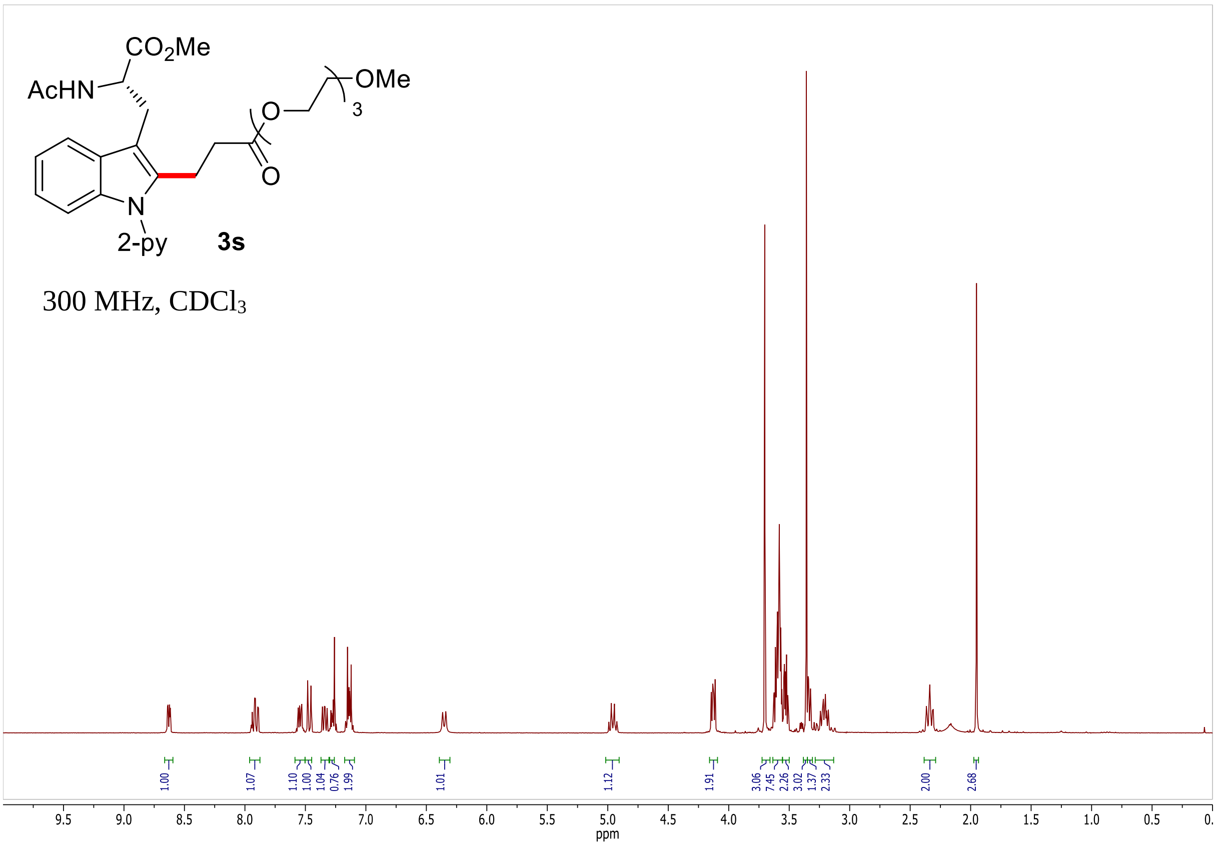

Supplementary Figure 58. ^{1}H-NMR spectrum of **3s**.

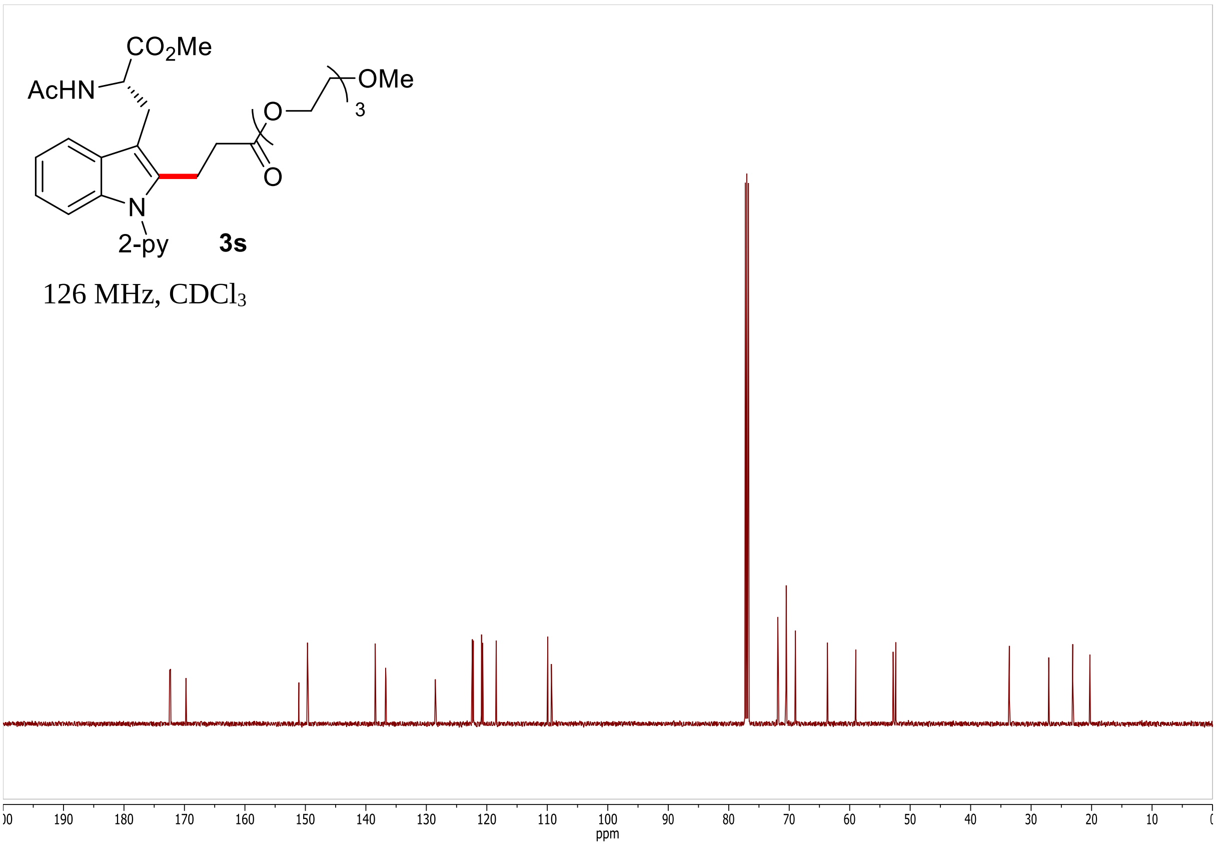

Supplementary Figure 59. ^{13}C-NMR spectrum of **3s**.

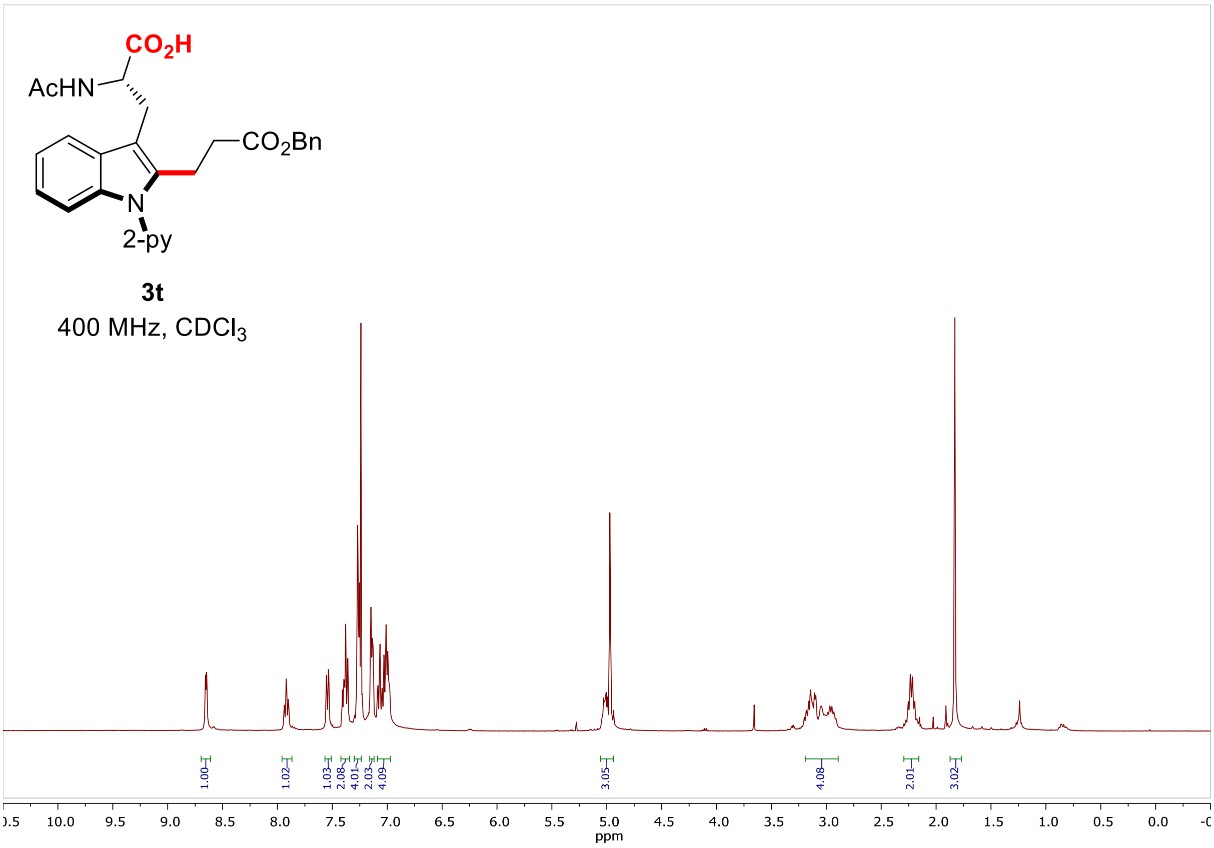

Supplementary Figure 60. ^{1}H-NMR spectrum of **3t**.

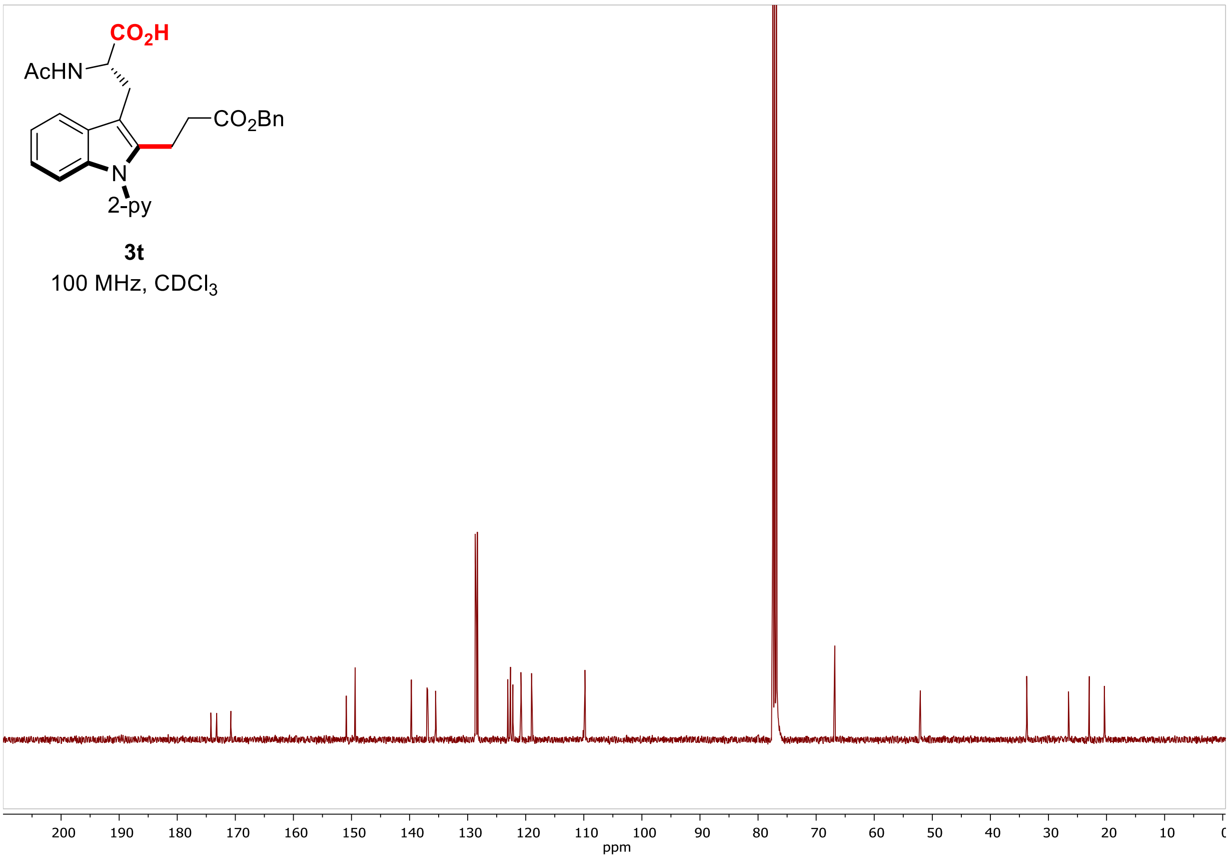

Supplementary Figure 61. ^{13}C-NMR spectrum of **3t**.

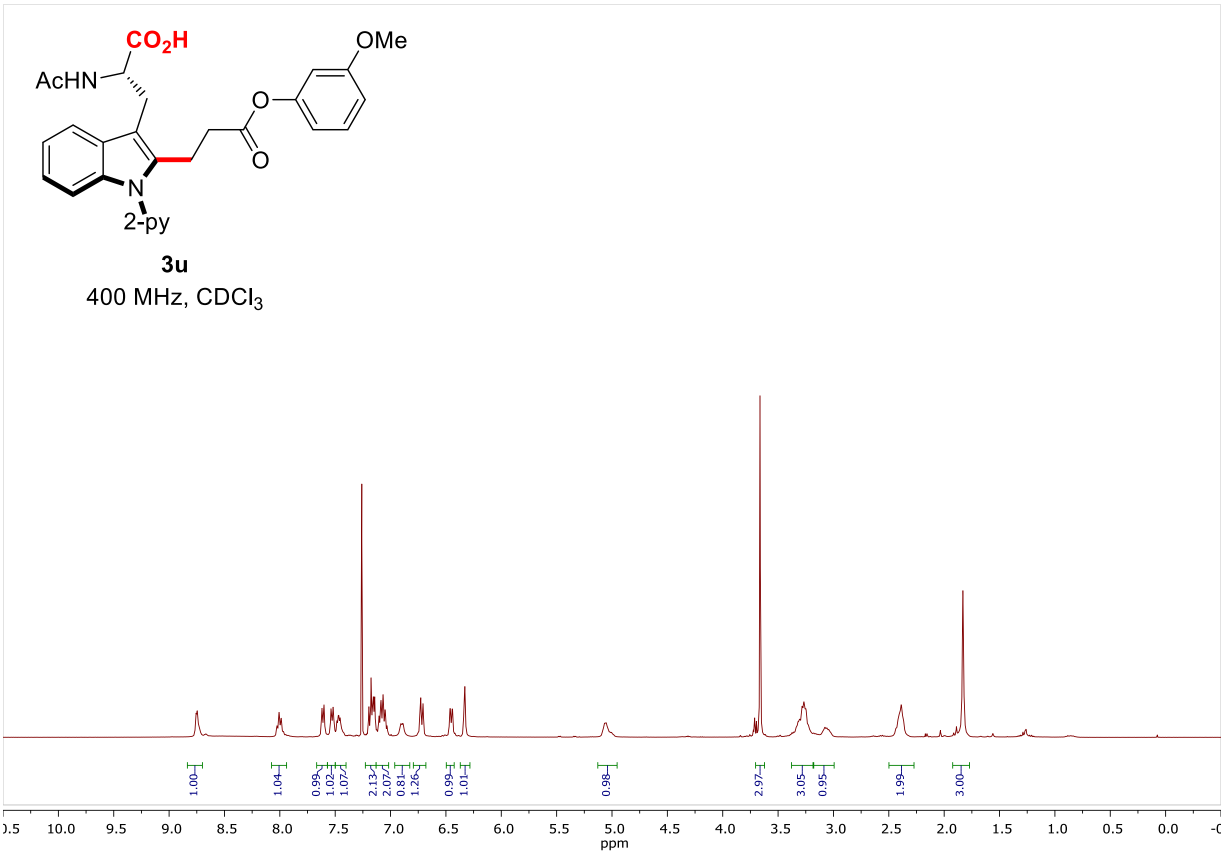

Supplementary Figure 62. ^{1}H-NMR spectrum of **3u**.

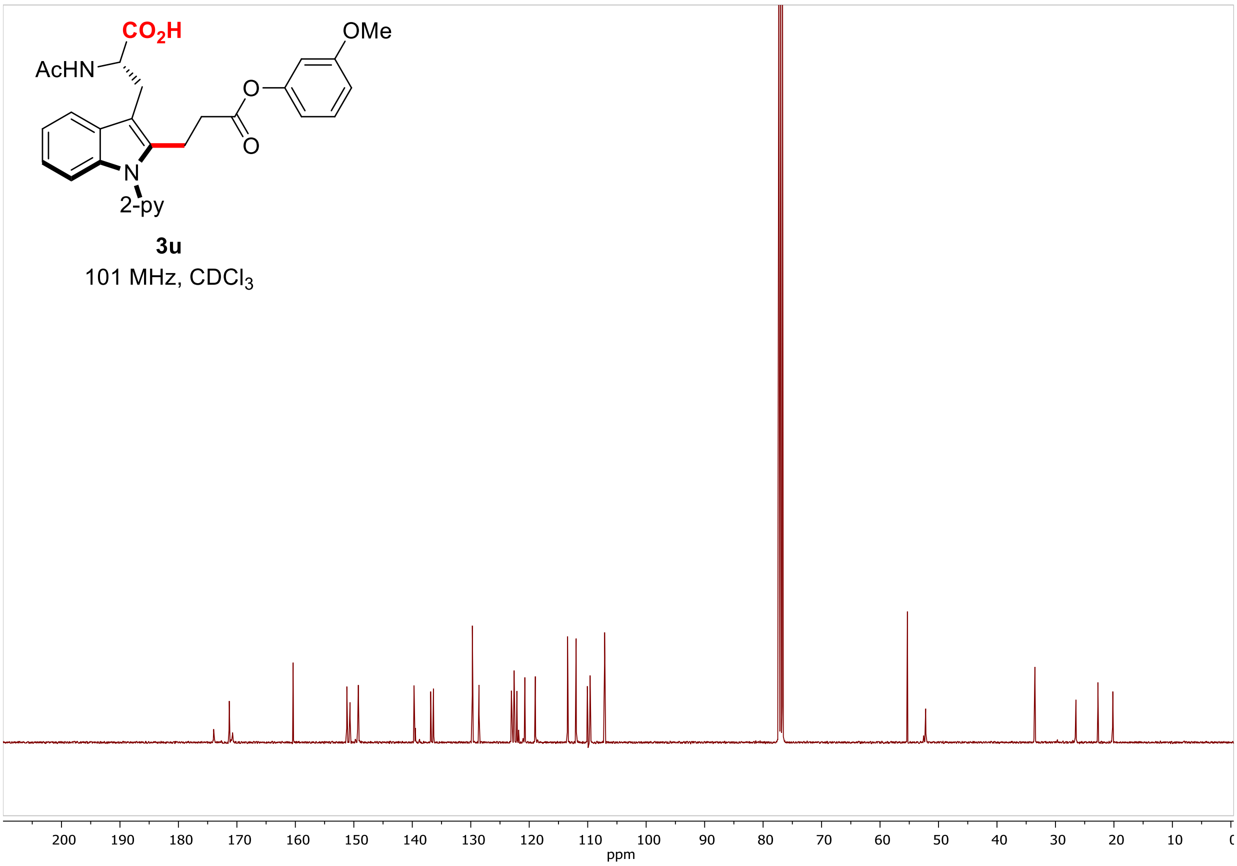

Supplementary Figure 63. ^{13}C-NMR spectrum of **3u**.

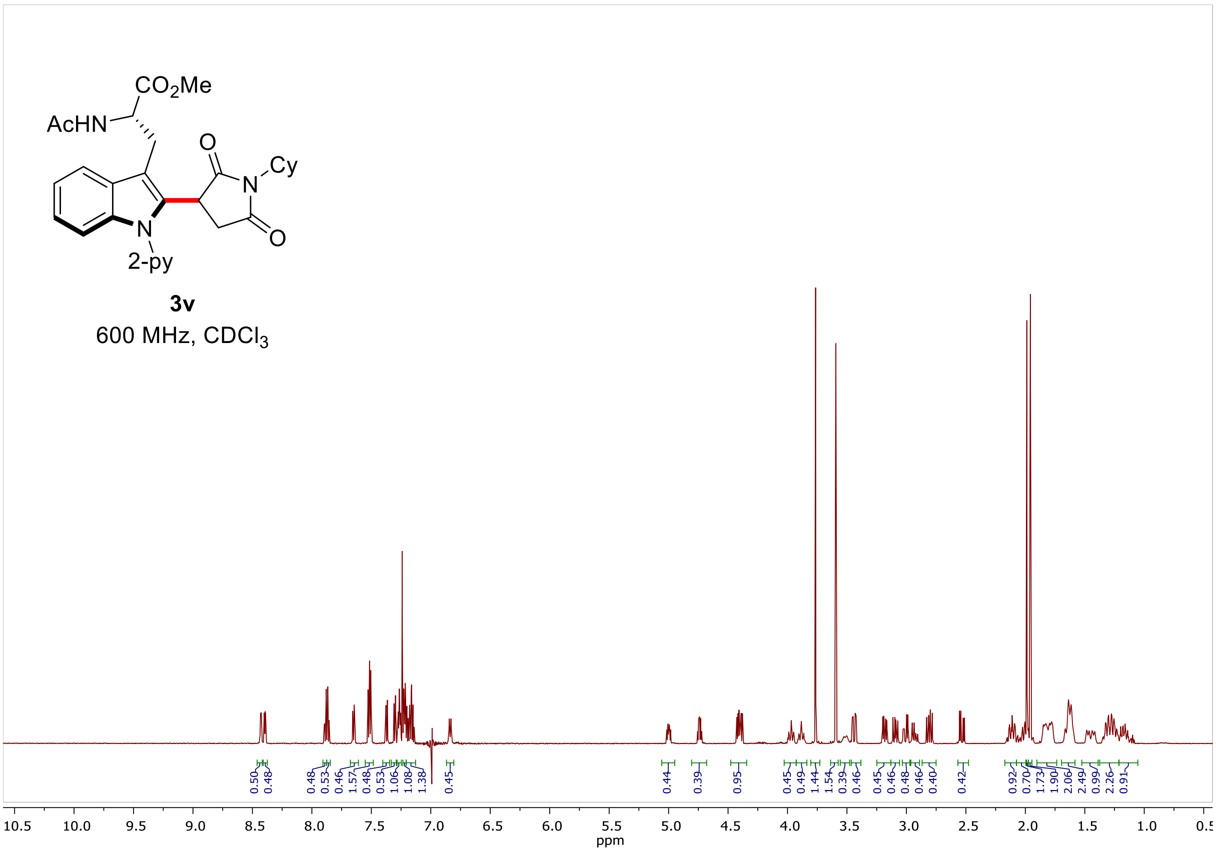

Supplementary Figure 64. ^{1}H-NMR spectrum of **3v**.

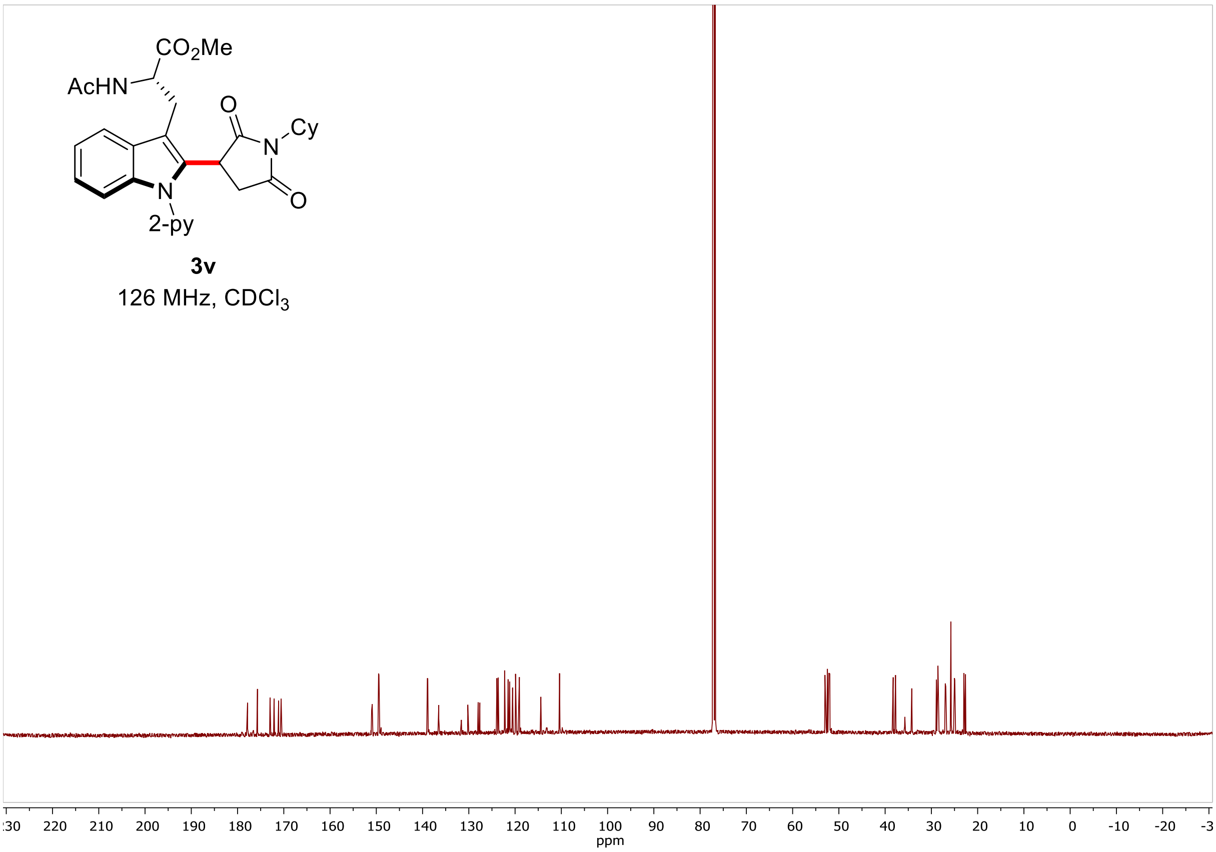

Supplementary Figure 65. ^{13}C-NMR spectrum of **3v**.

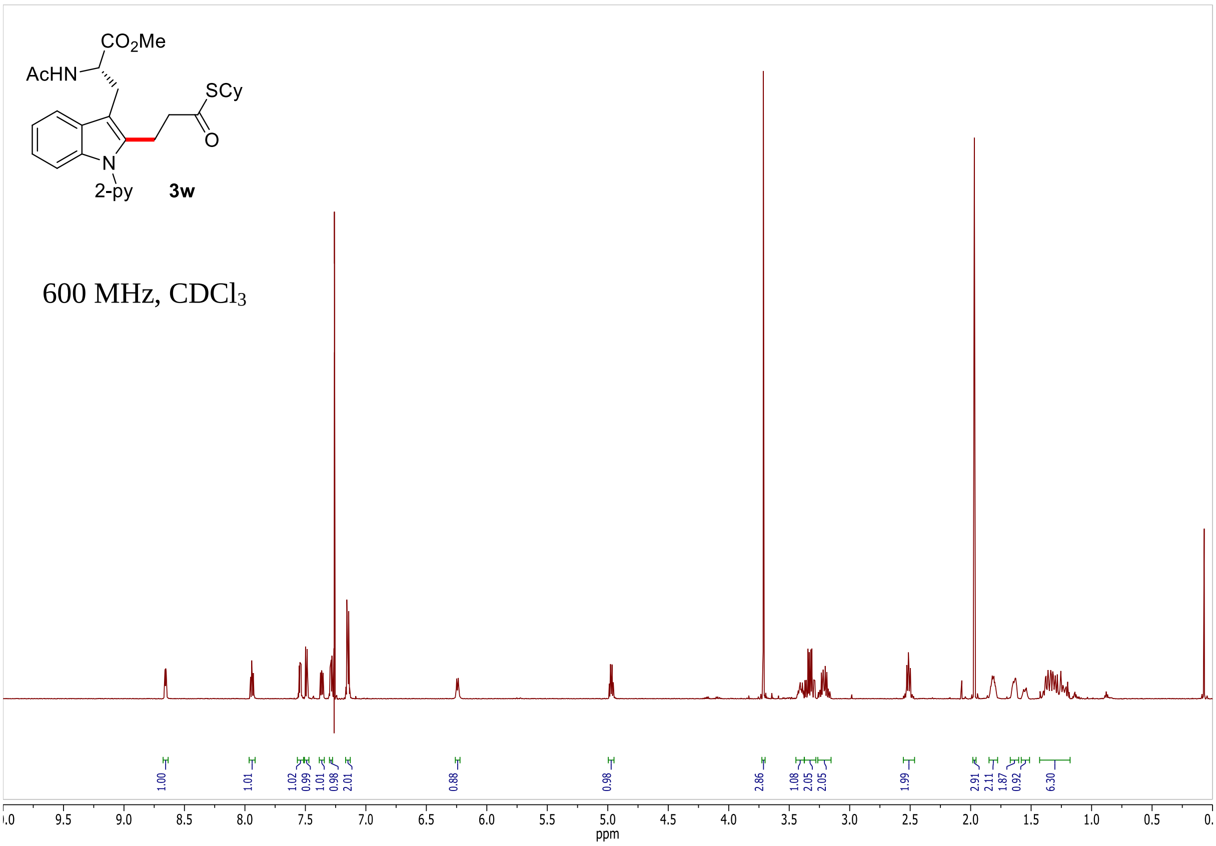

Supplementary Figure 66. ^{1}H-NMR spectrum of **3w**.

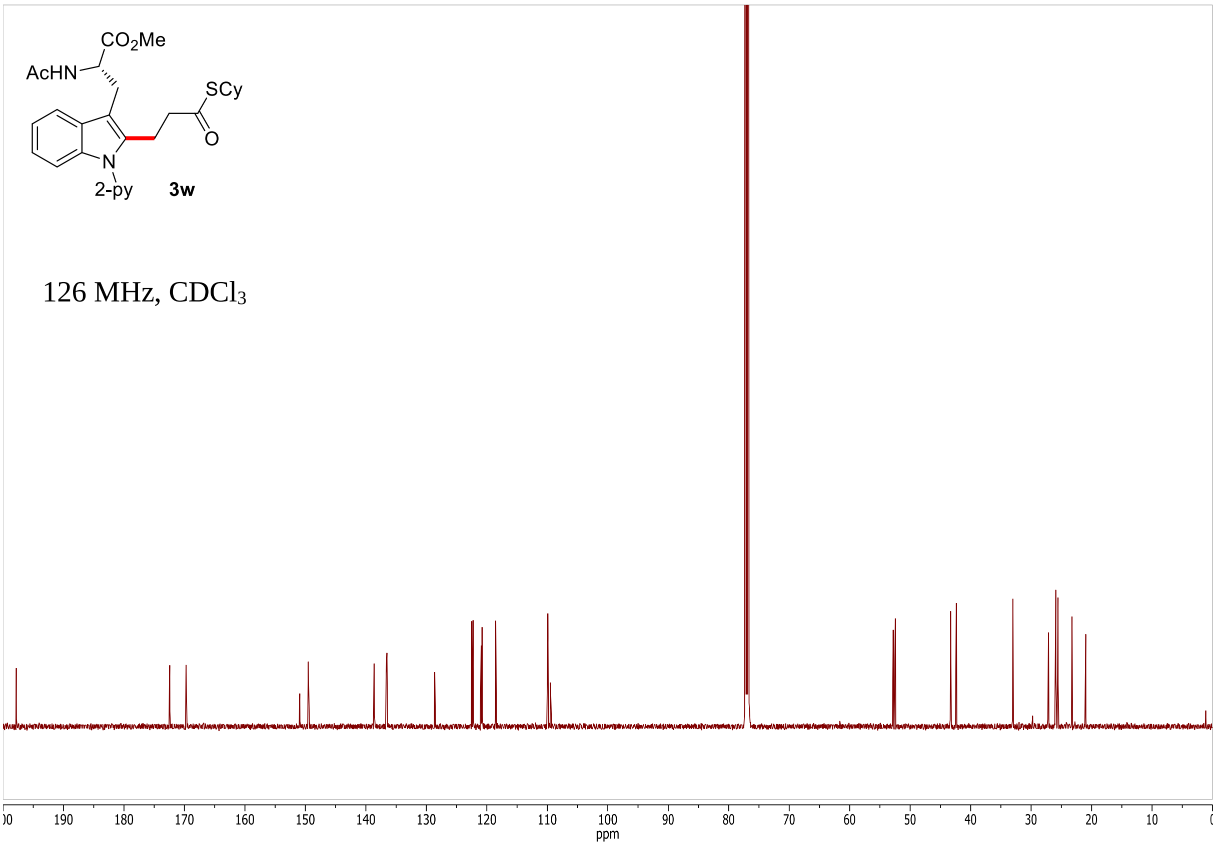

Supplementary Figure 67. ^{13}C-NMR spectrum of **3w**.

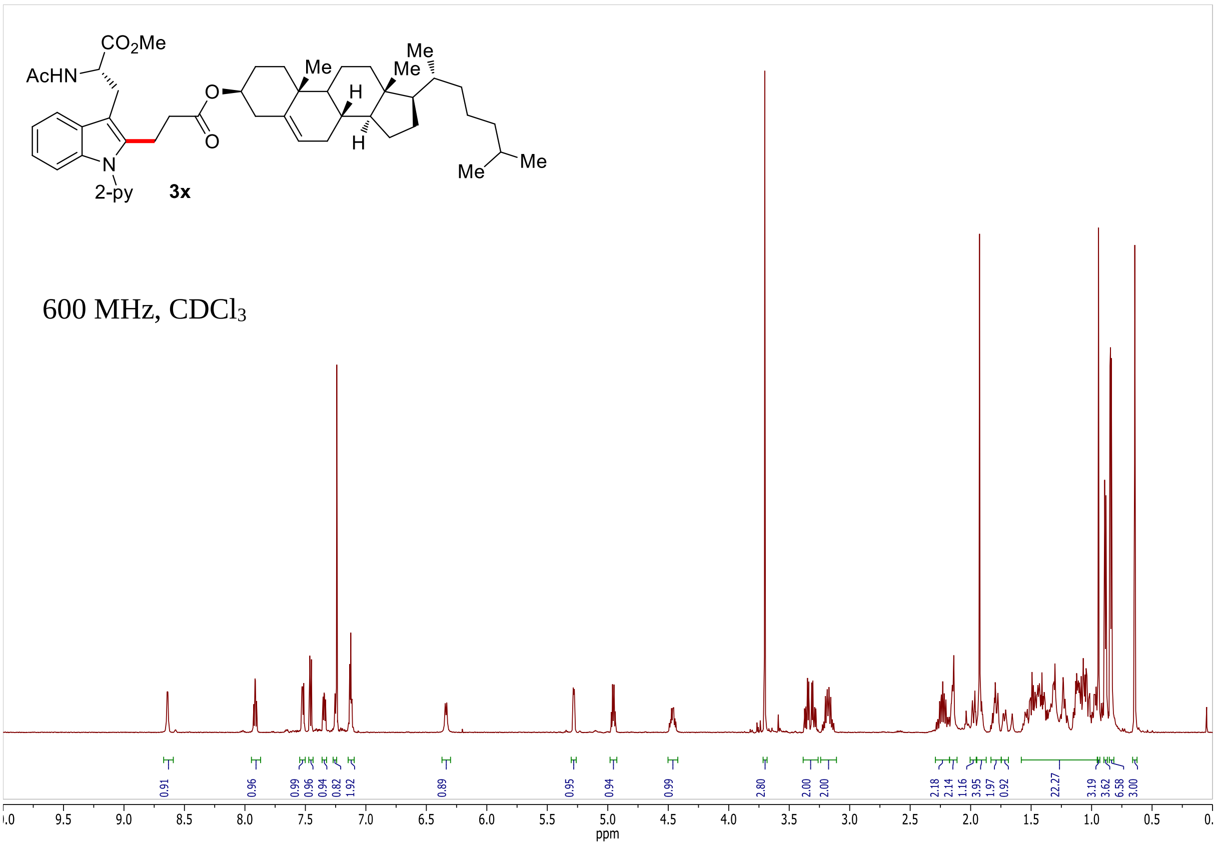

Supplementary Figure 68. ^{1}H-NMR spectrum of **3x**.

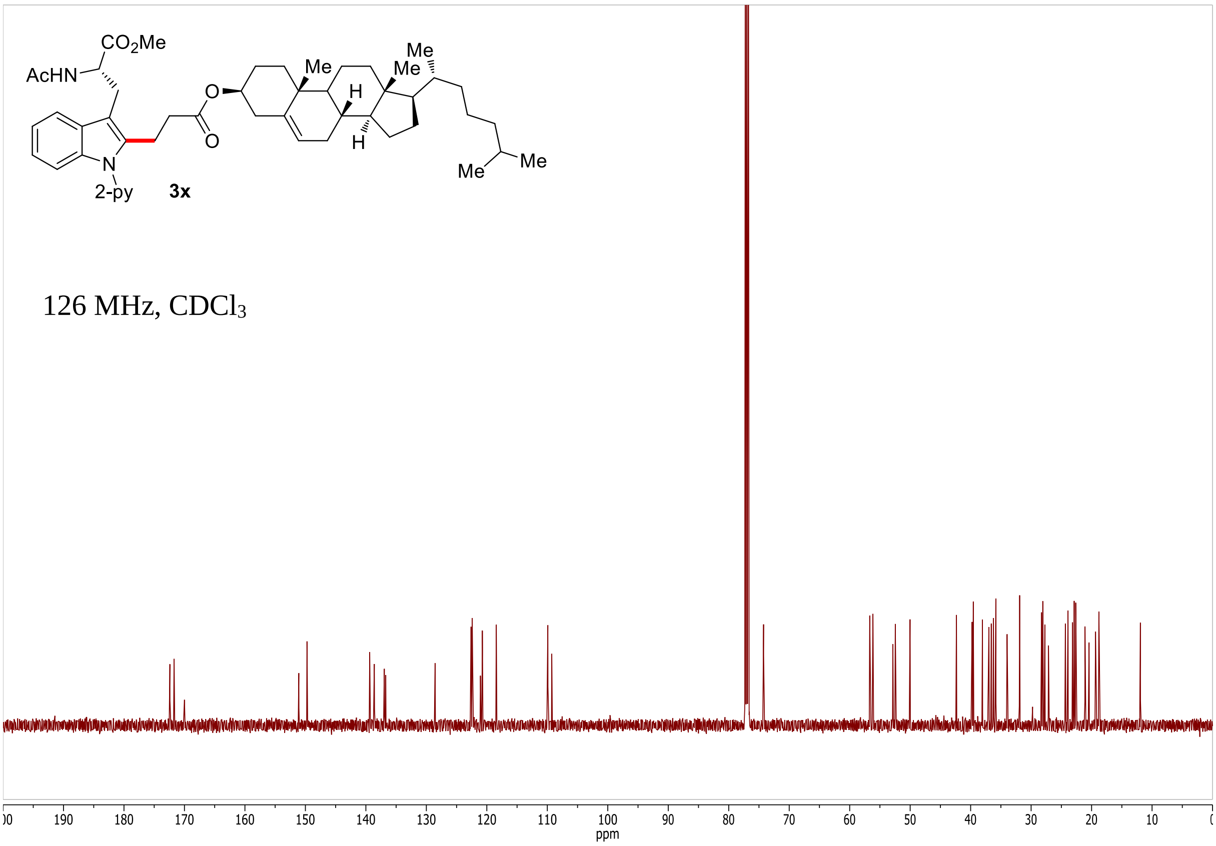

Supplementary Figure 69. ^{13}C-NMR spectrum of **3x**.

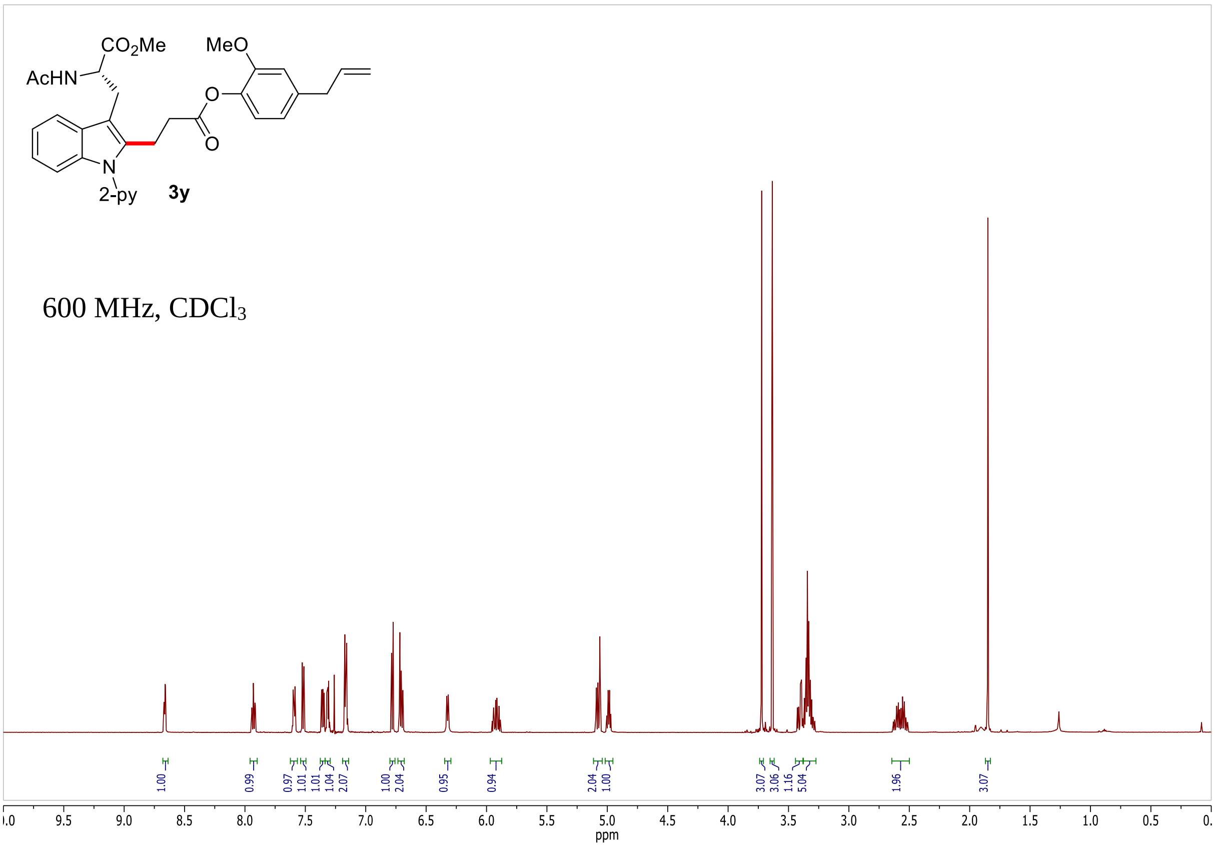

Supplementary Figure 70. ^{1}H-NMR spectrum of **3y**.

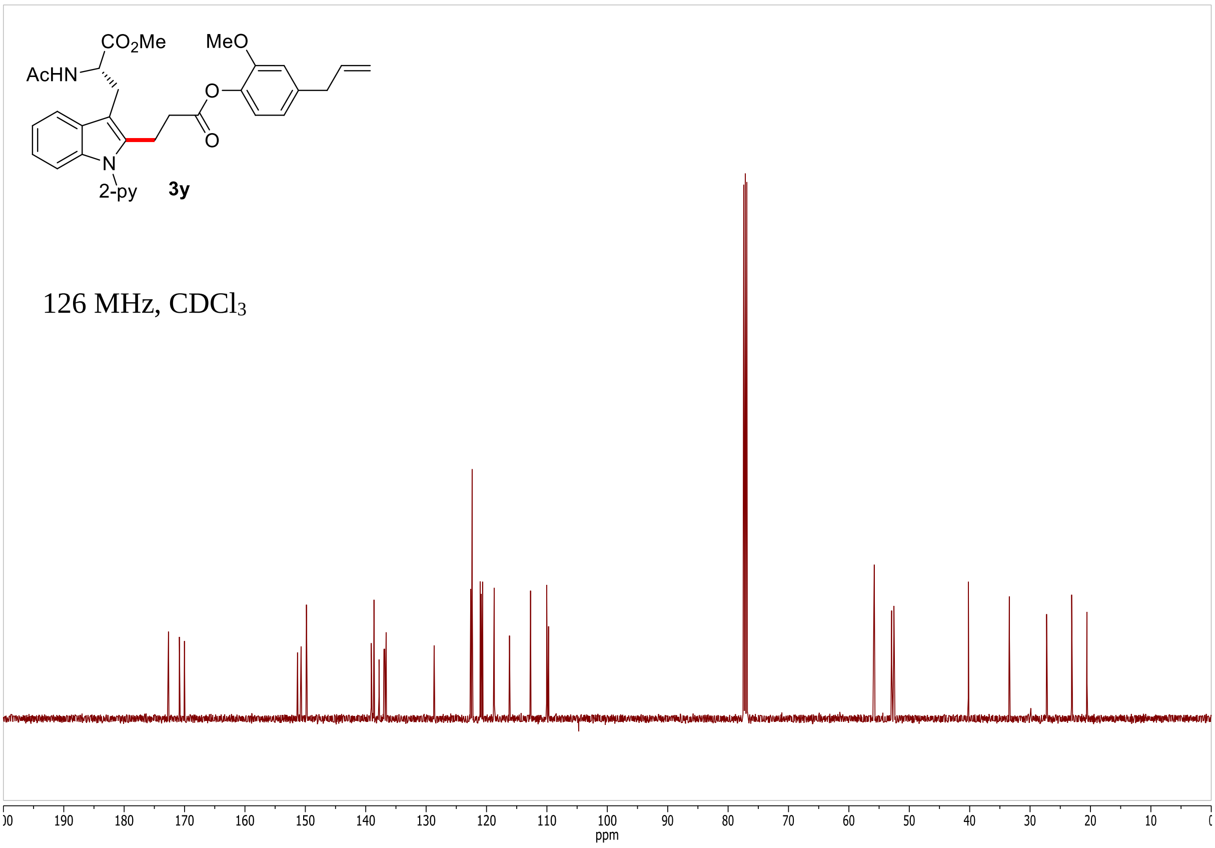

Supplementary Figure 71. ^{13}C-NMR spectrum of **3y**.

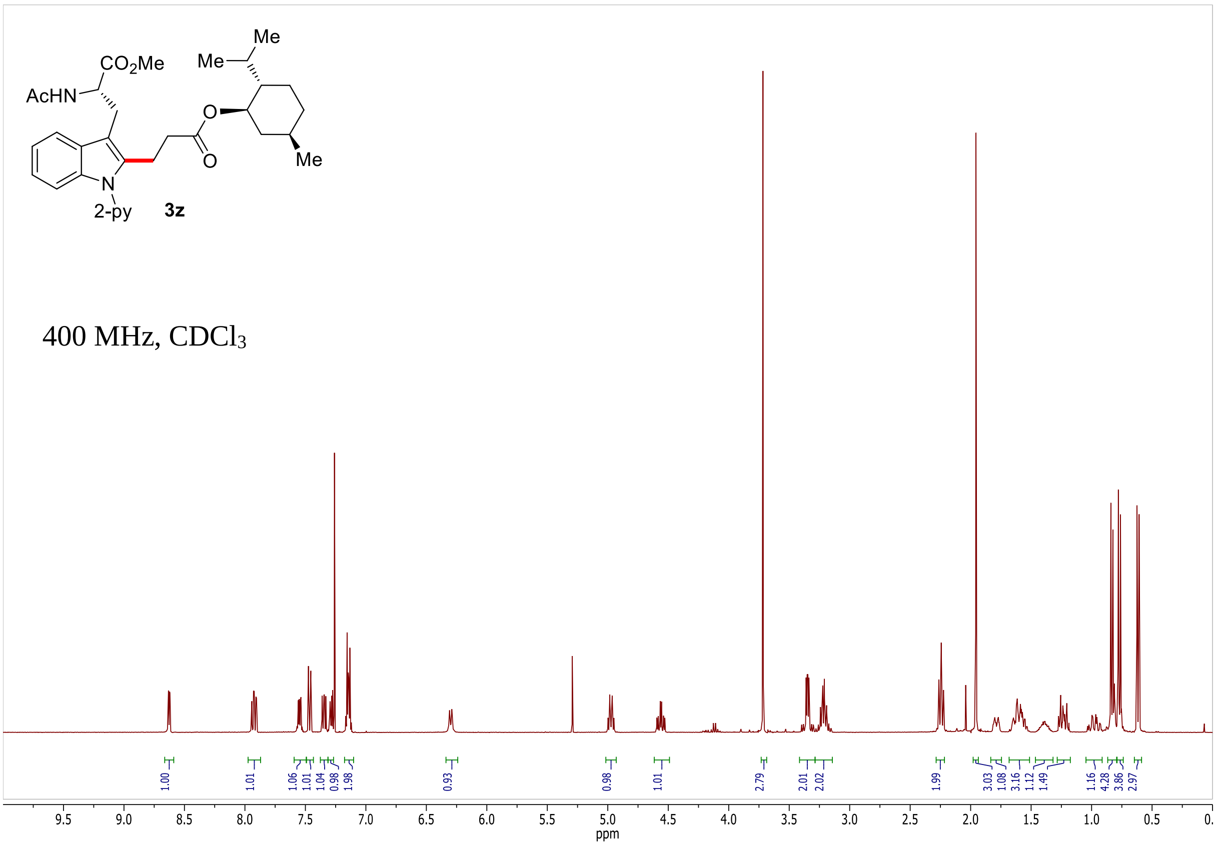

Supplementary Figure 72. ^{1}H-NMR spectrum of **3z**.

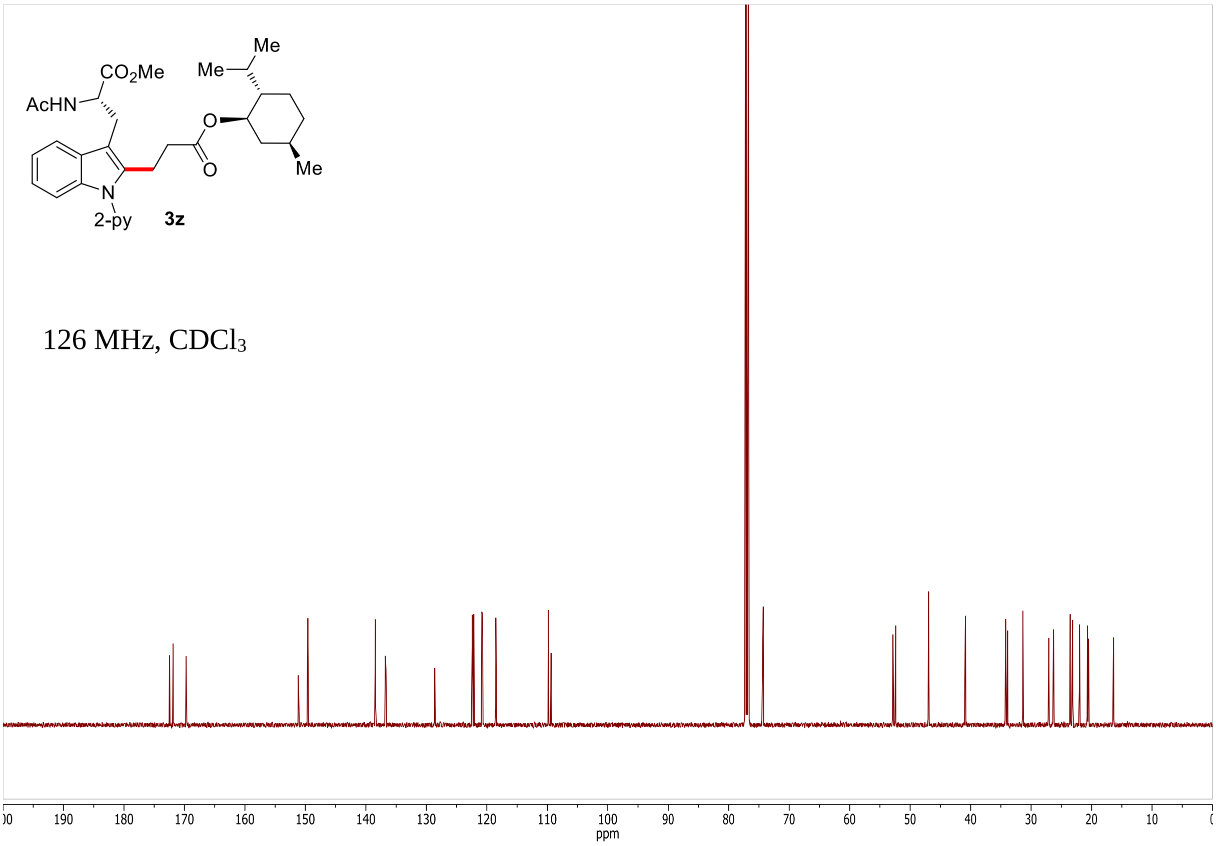

Supplementary Figure 73. ^{13}C-NMR spectrum of **3z**.

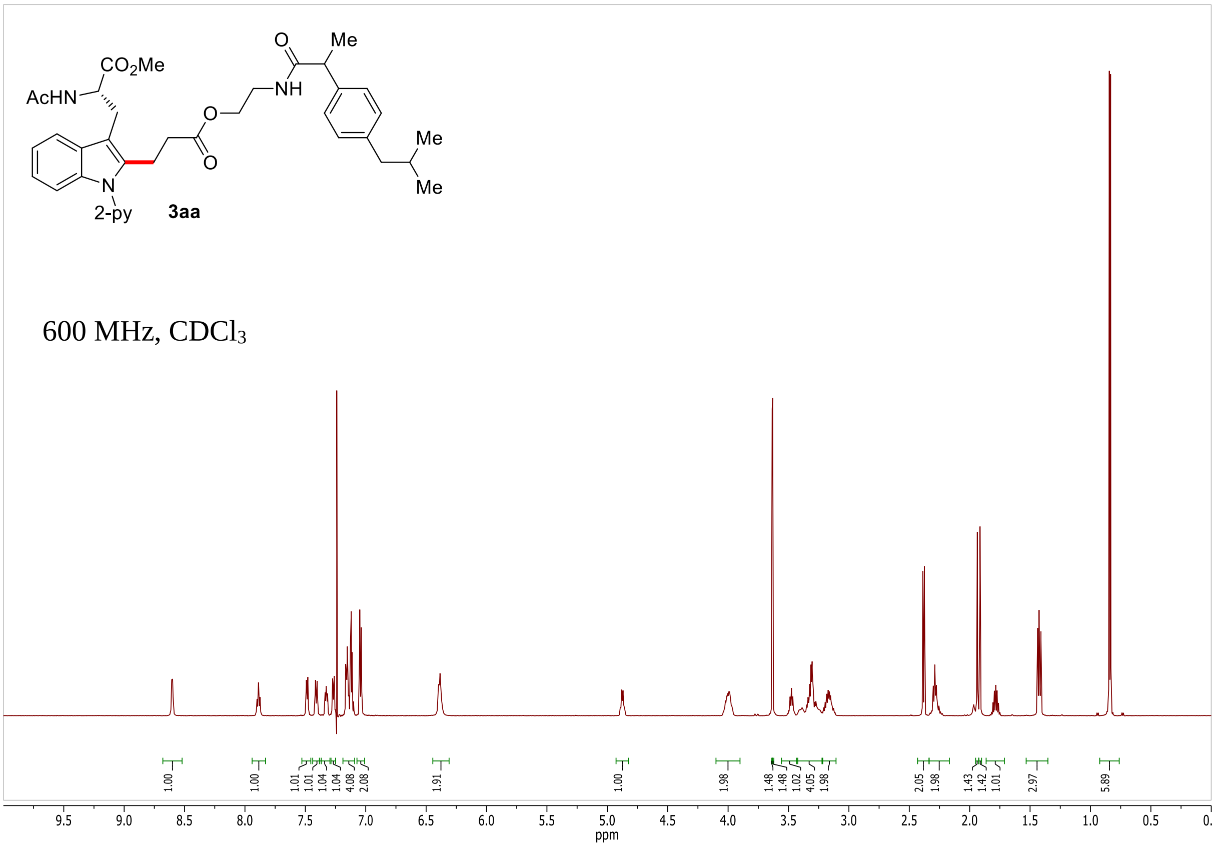

Supplementary Figure 74. ^{1}H-NMR spectrum of **3aa**.

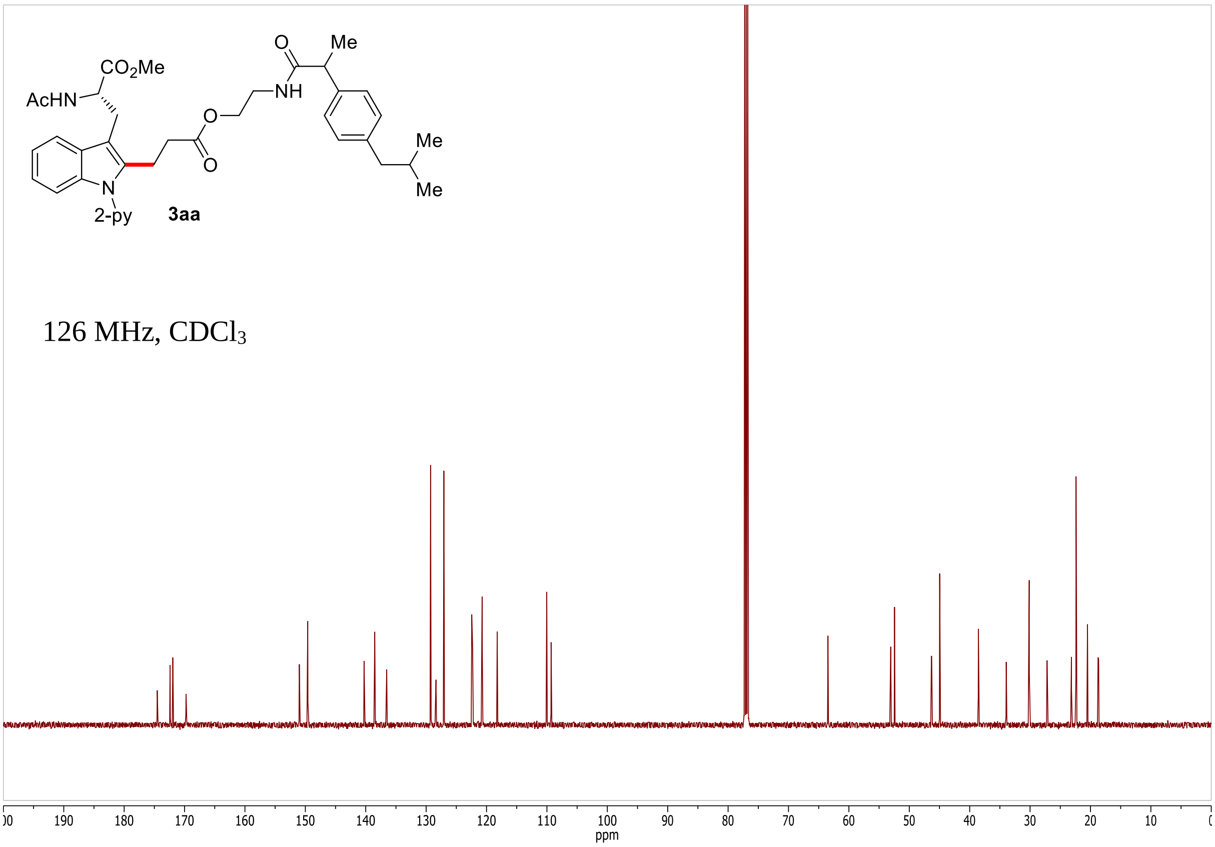

Supplementary Figure 75. ^{13}C-NMR spectrum of **3aa**.

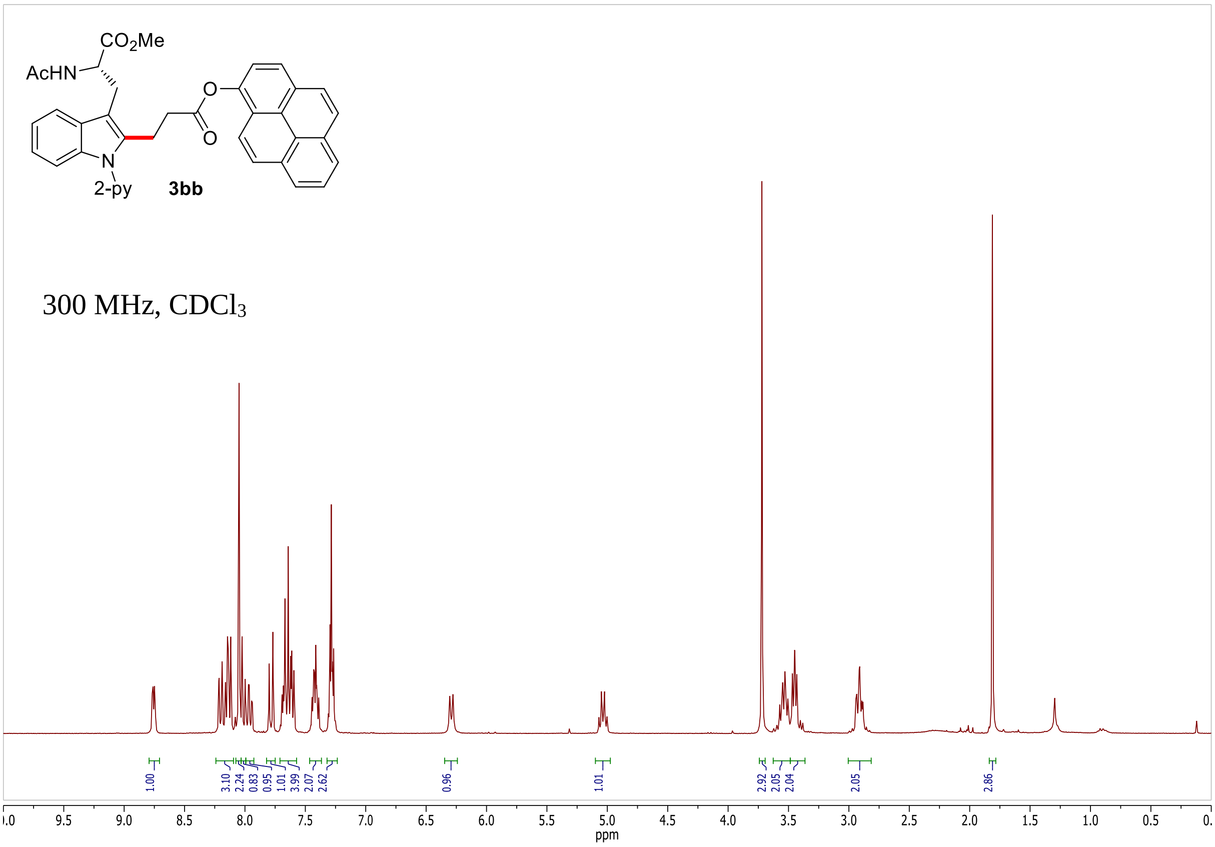

Supplementary Figure 76. ^{1}H-NMR spectrum of **3bb**.

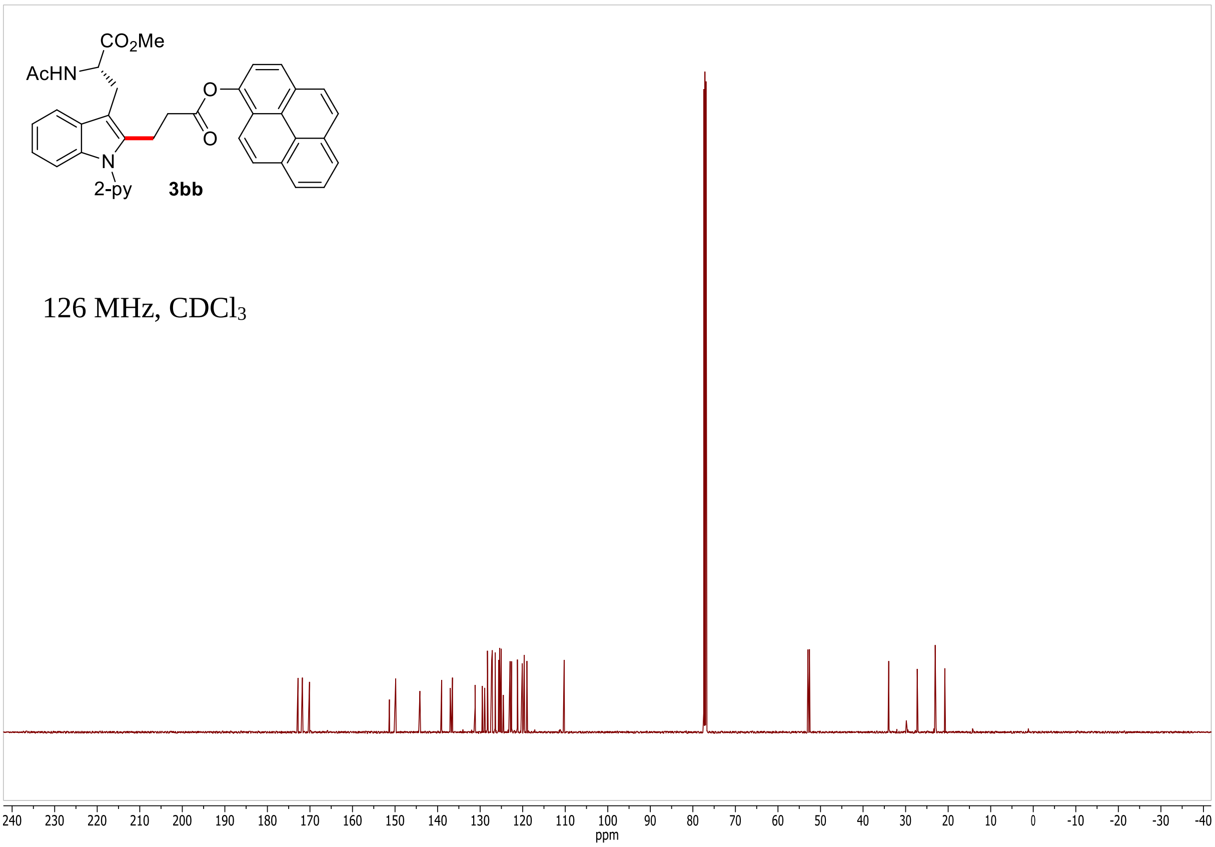

Supplementary Figure 77. ^{13}C-NMR spectrum of **3bb**.

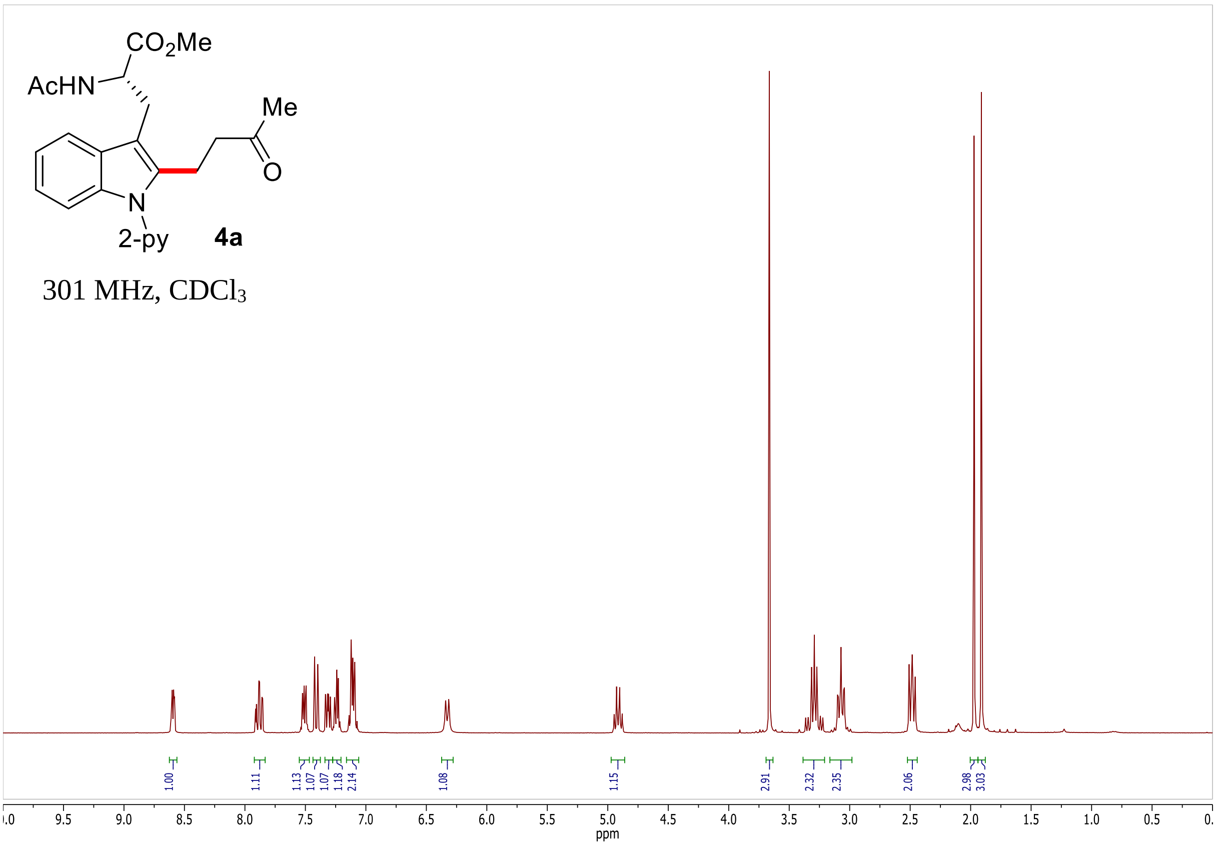

Supplementary Figure 78. ^{1}H-NMR spectrum of **4a**.

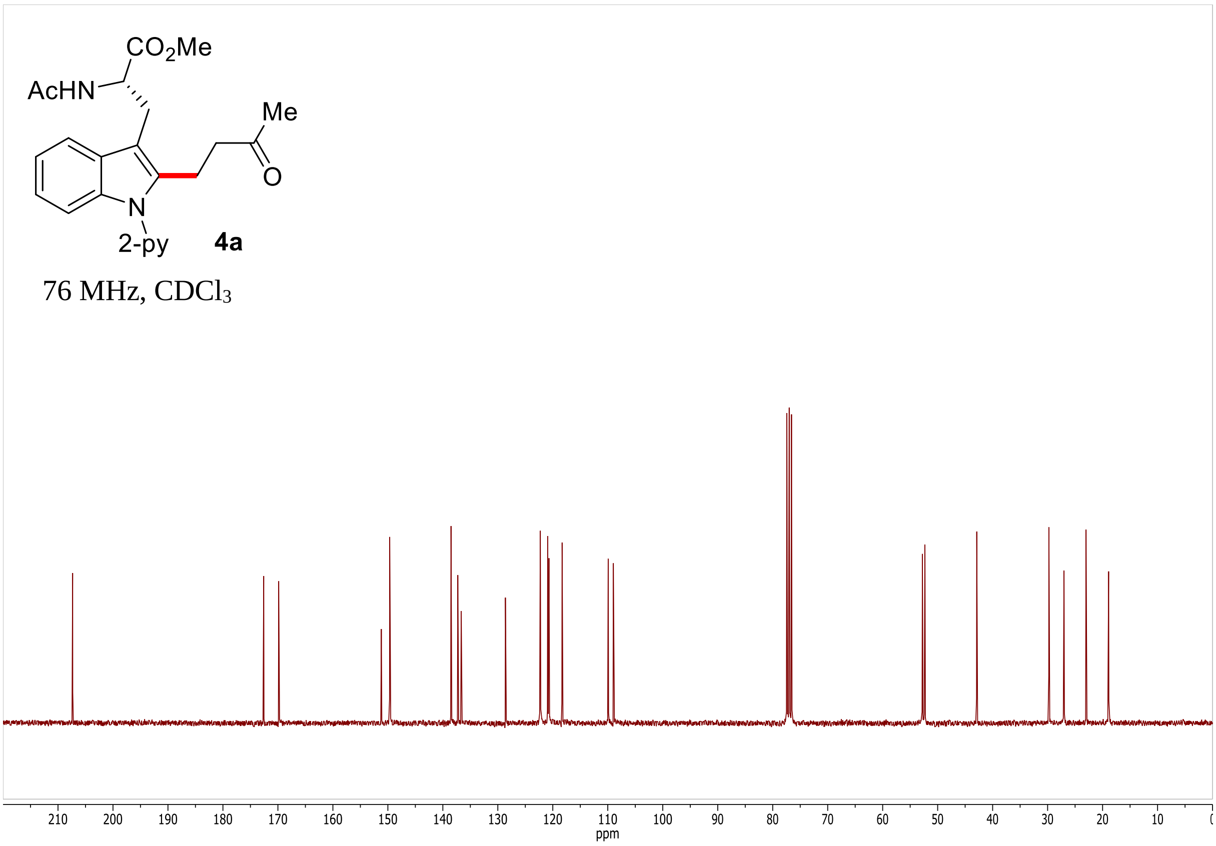

Supplementary Figure 79. ^{13}C-NMR spectrum of **4a**.

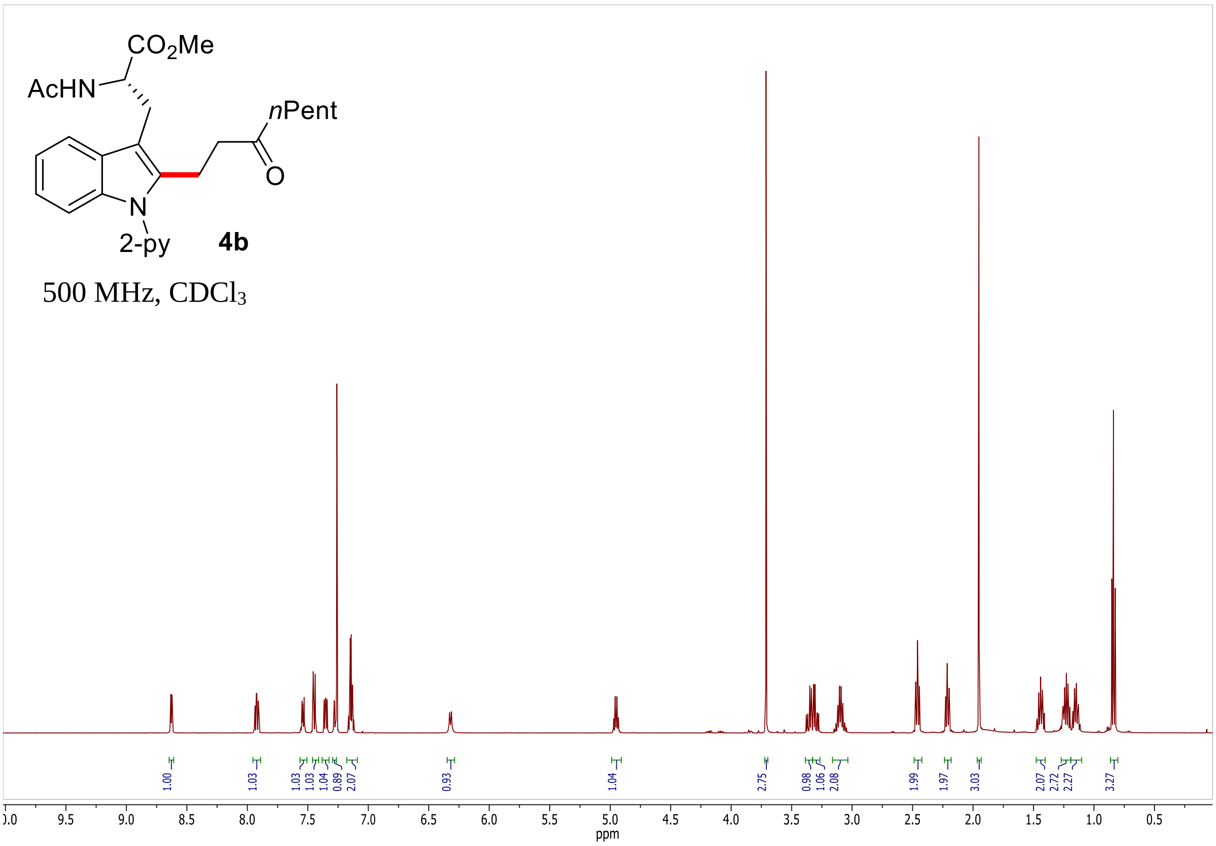

Supplementary Figure 80. ^{1}H-NMR spectrum of **4b**.

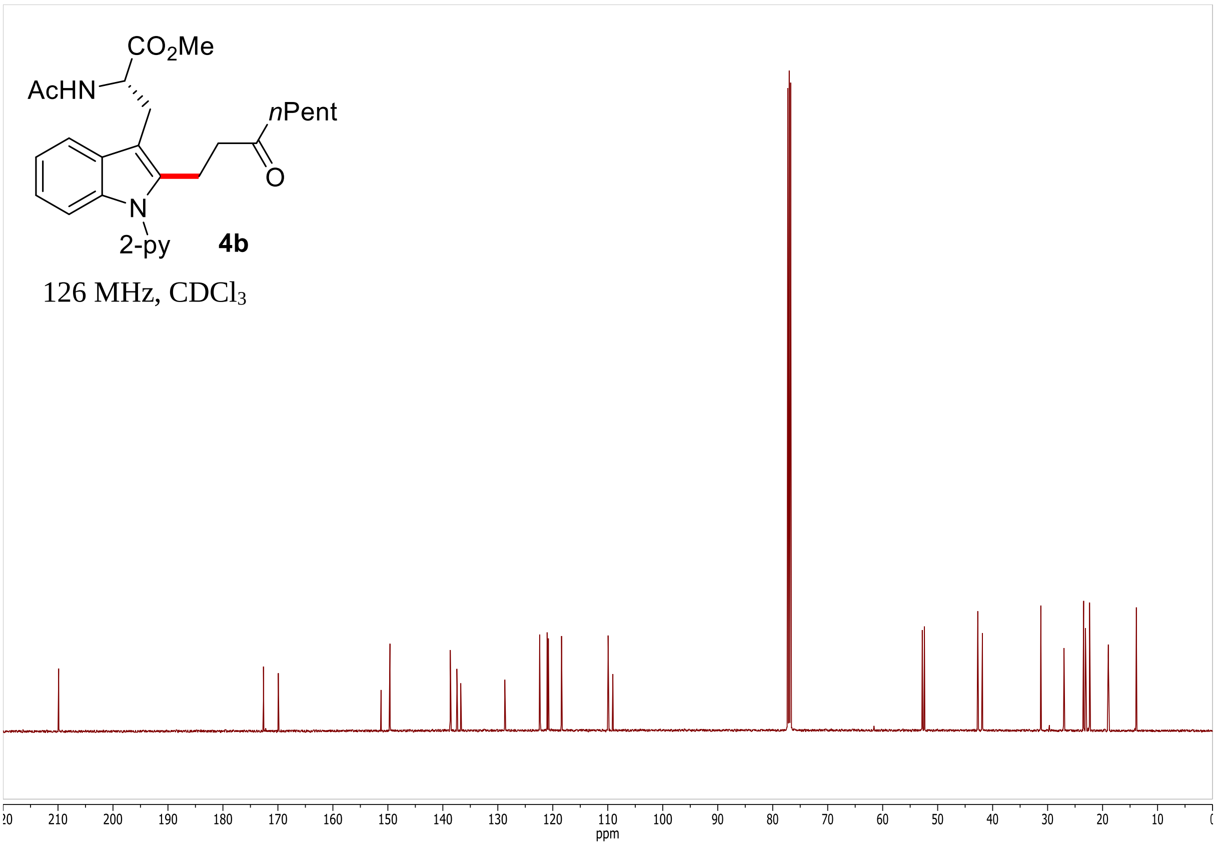

Supplementary Figure 81. ^{13}C-NMR spectrum of **4b**.

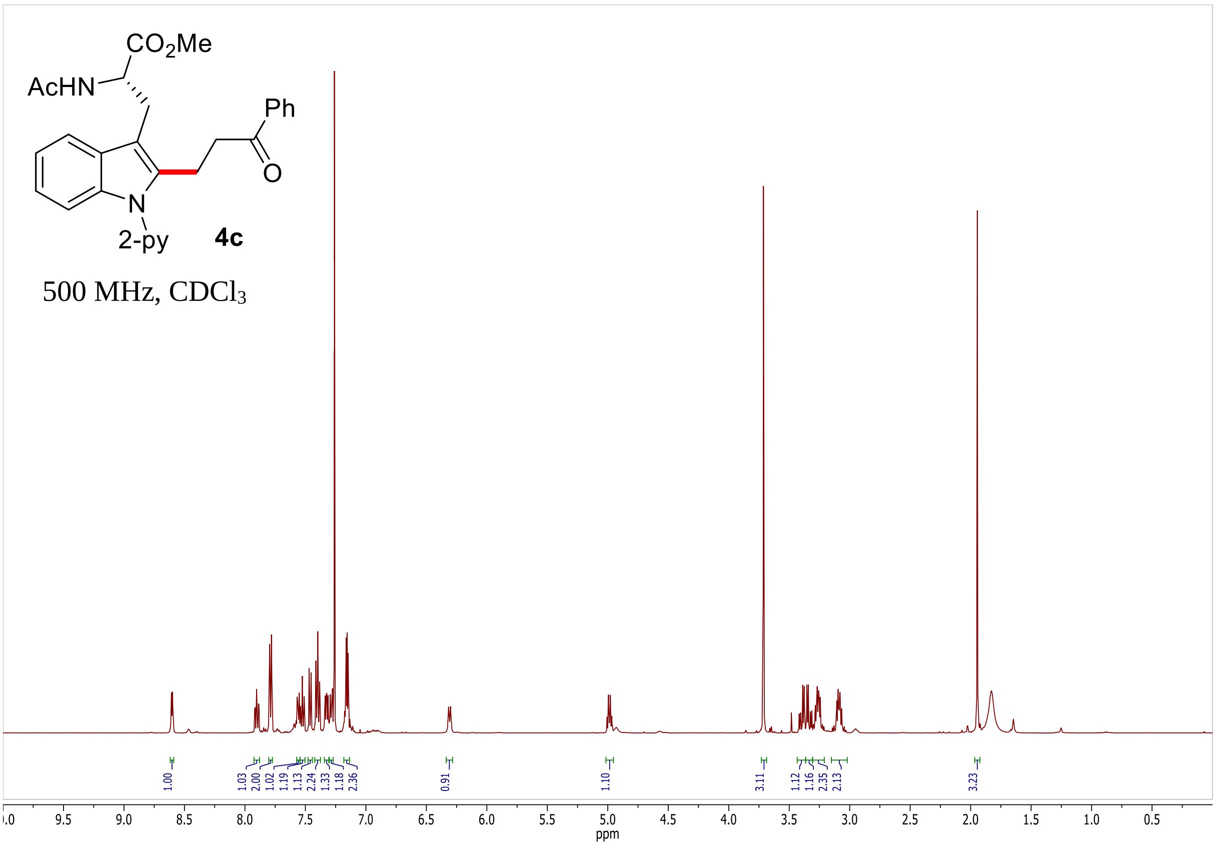

Supplementary Figure 82. ^{1}H-NMR spectrum of **4c**.

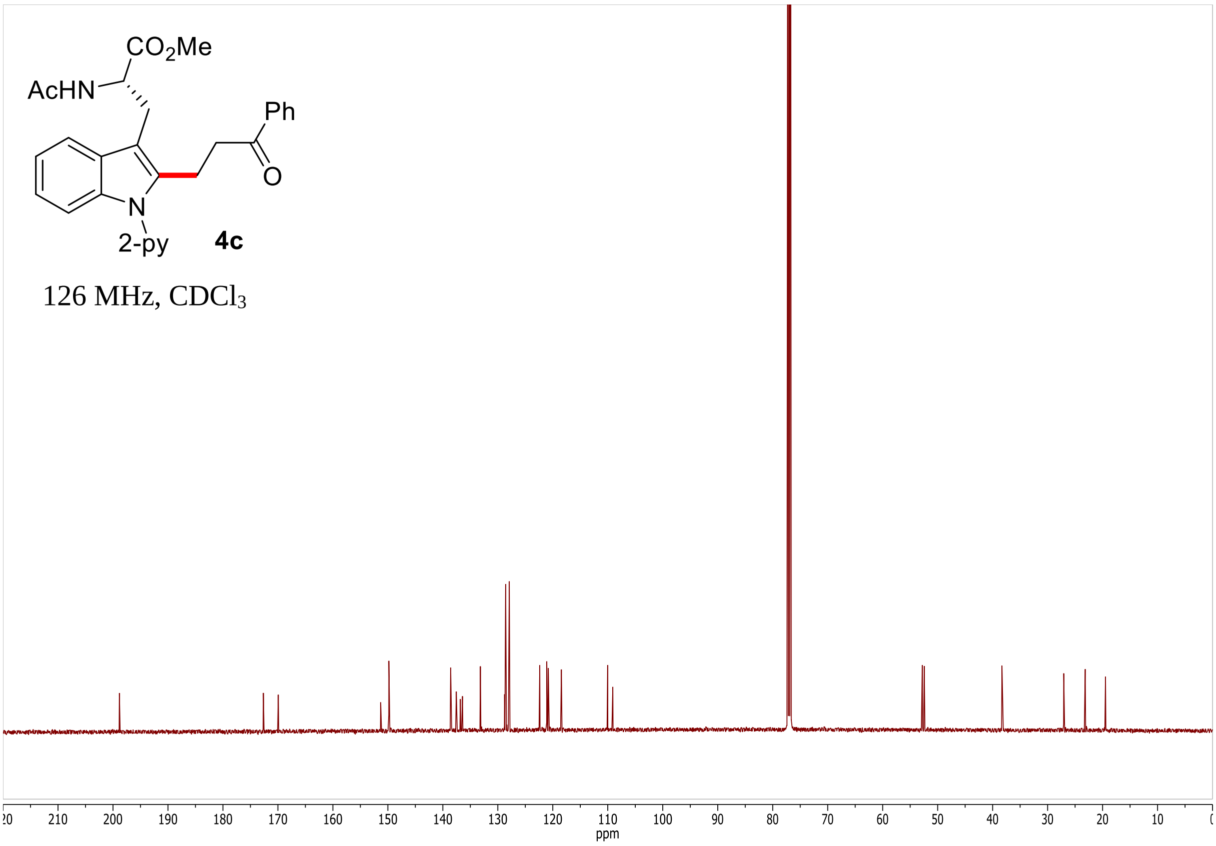

Supplementary Figure 83. ^{13}C-NMR spectrum of **4c**.

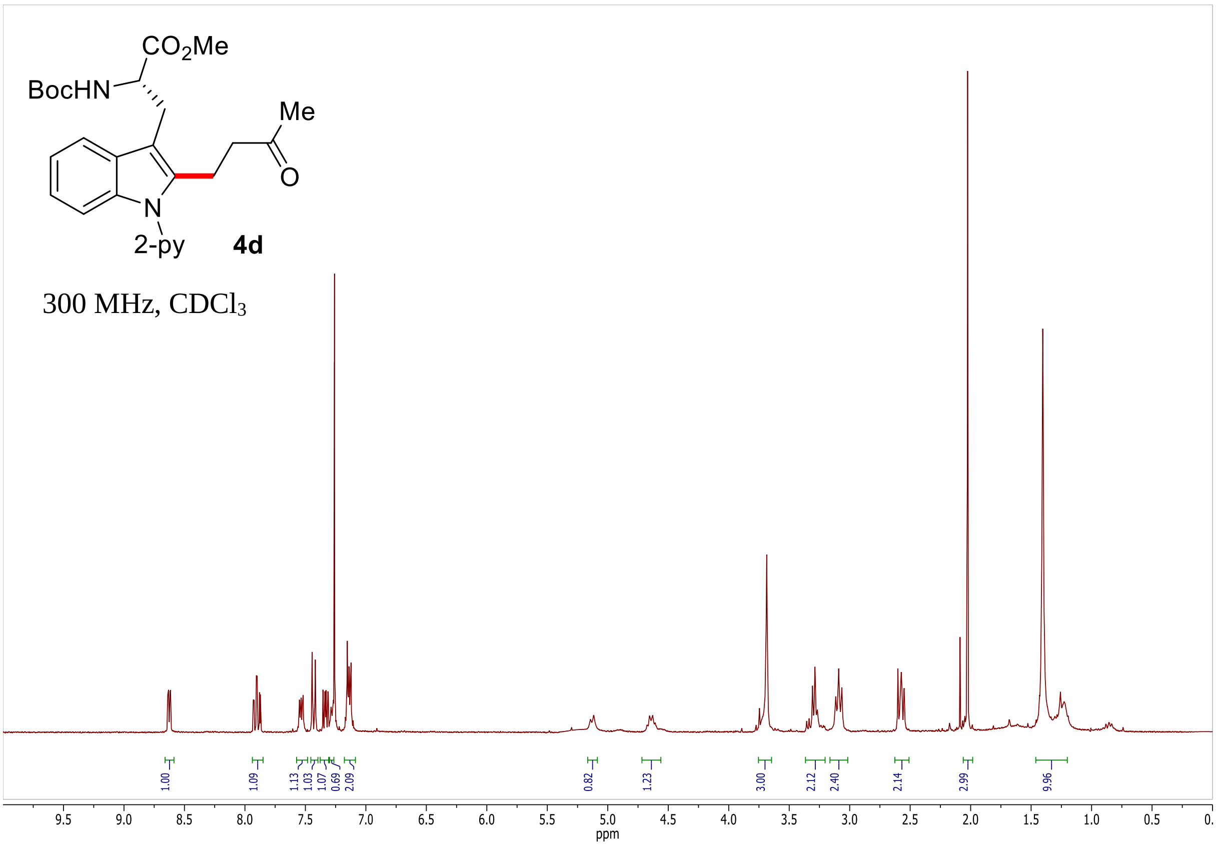

Supplementary Figure 84. ^{1}H-NMR spectrum of **4d**.

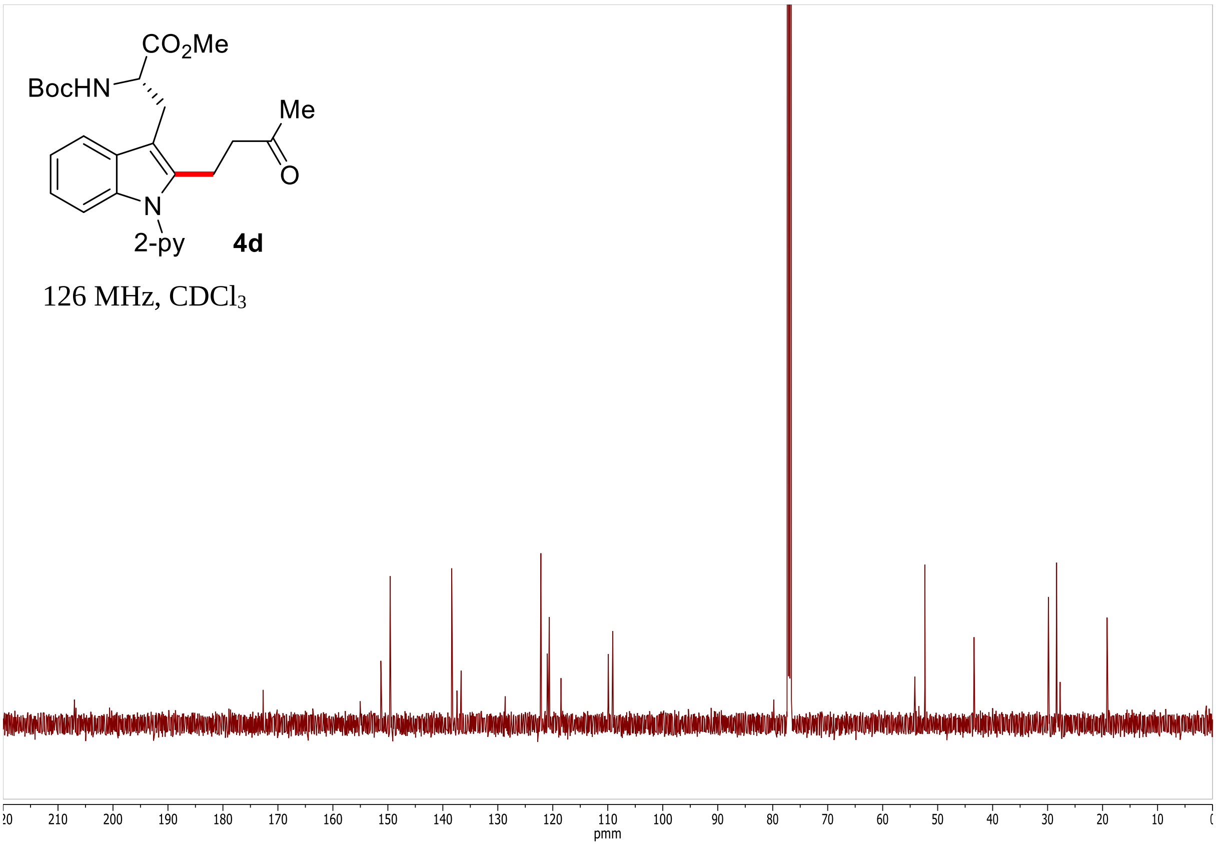

Supplementary Figure 85. ^{13}C-NMR spectrum of **4d**.

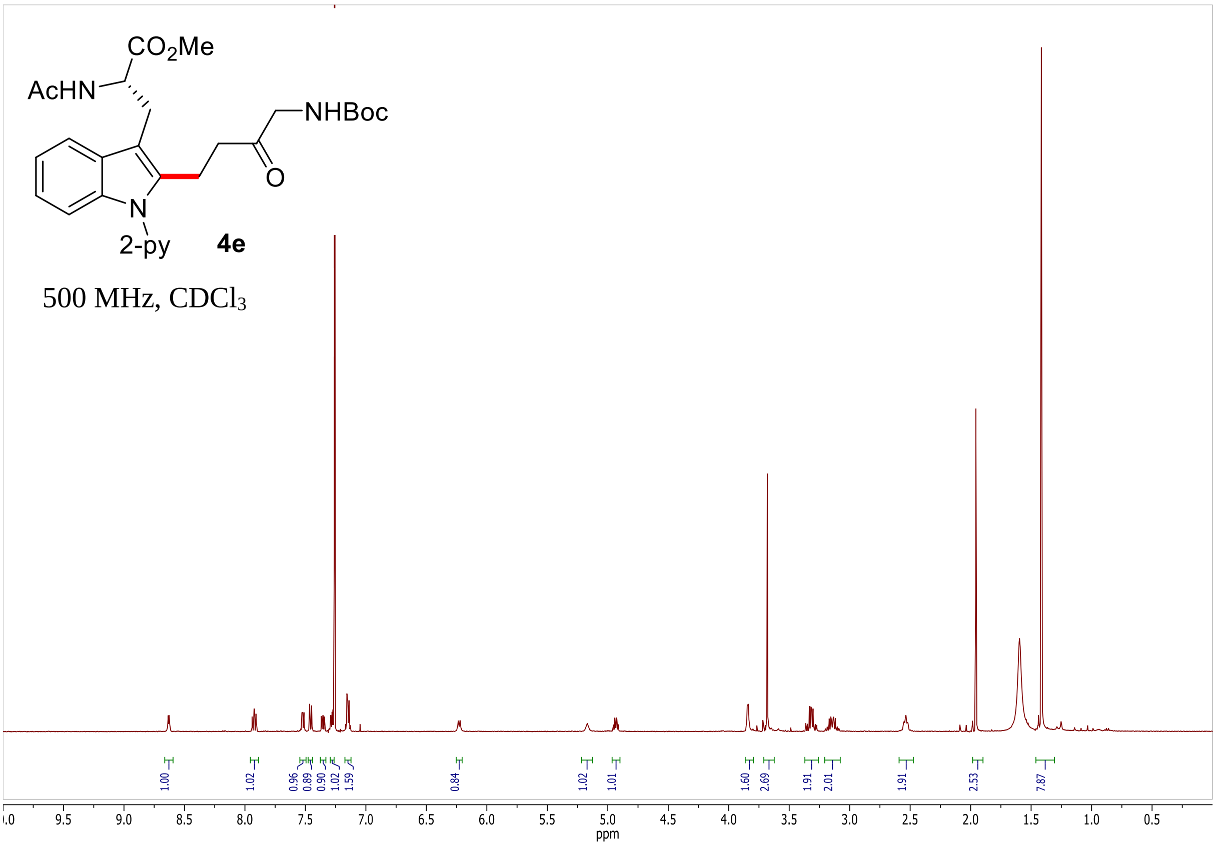

Supplementary Figure 86. ^{1}H-NMR spectrum of **4e**.

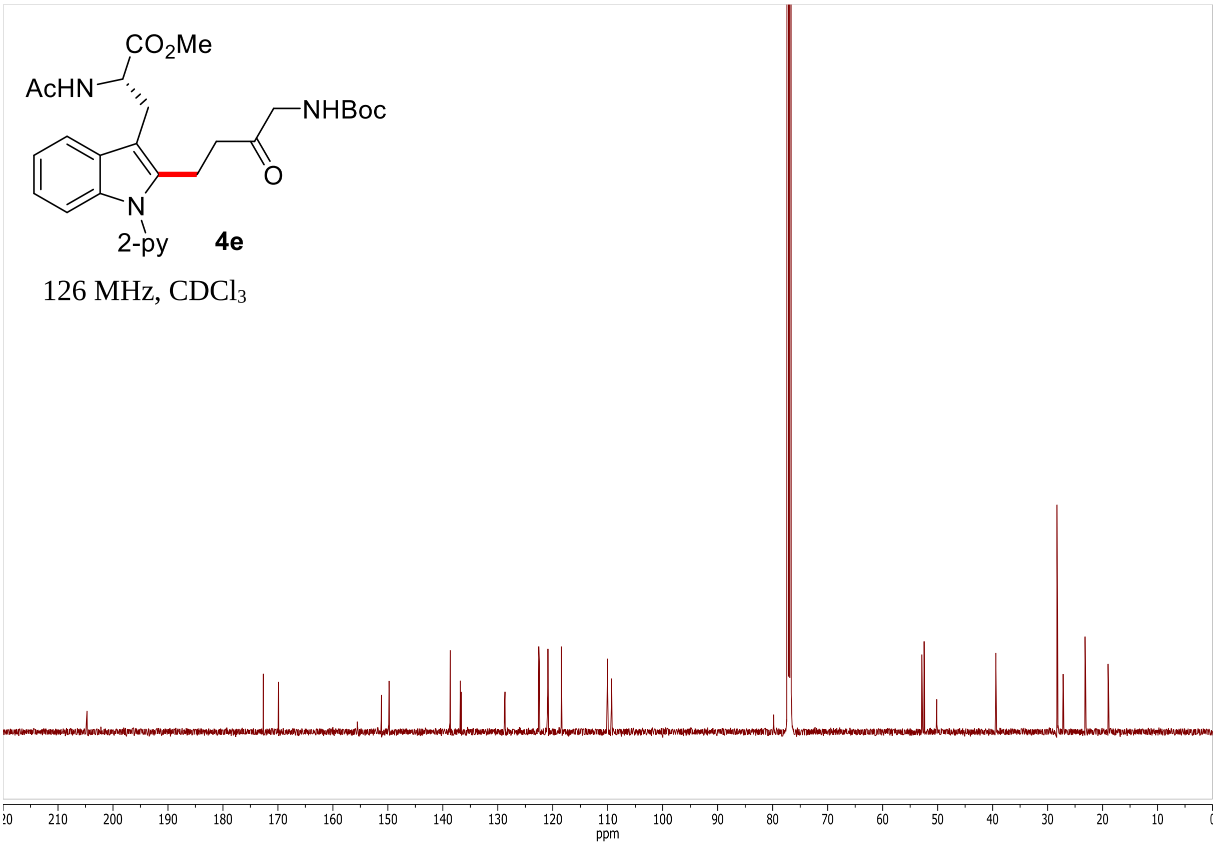

Supplementary Figure 87. ^{13}C-NMR spectrum of **4e**.

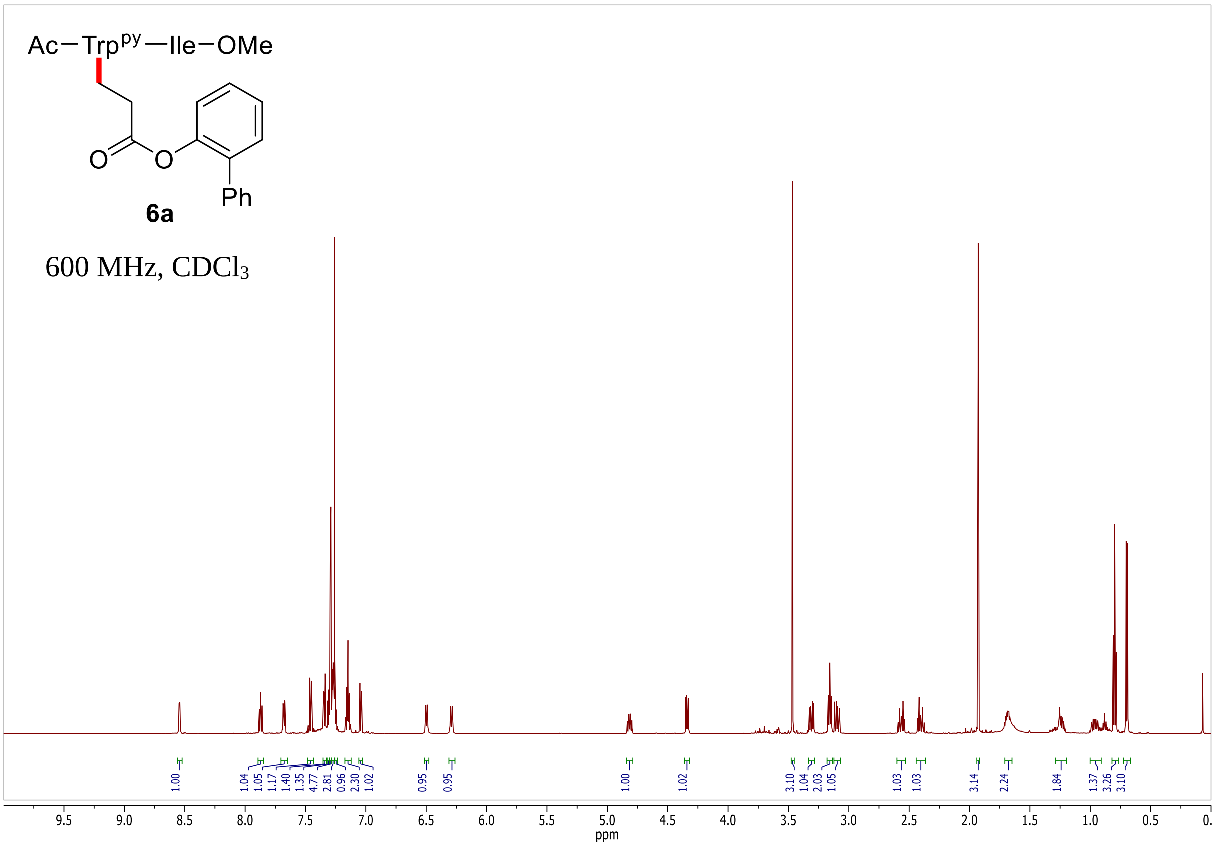

Supplementary Figure 88. ^{1}H-NMR spectrum of **6a**.

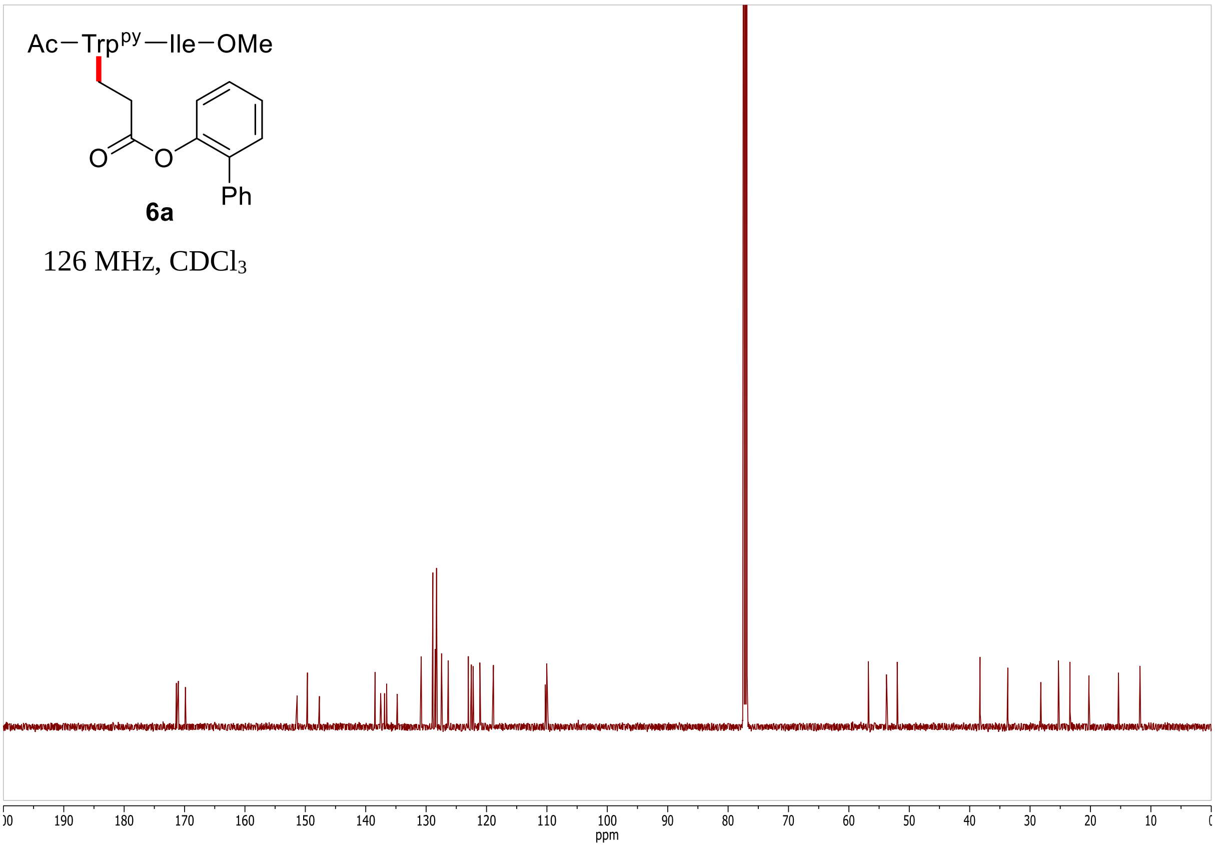

Supplementary Figure 89. ^{13}C-NMR spectrum of **6a**.

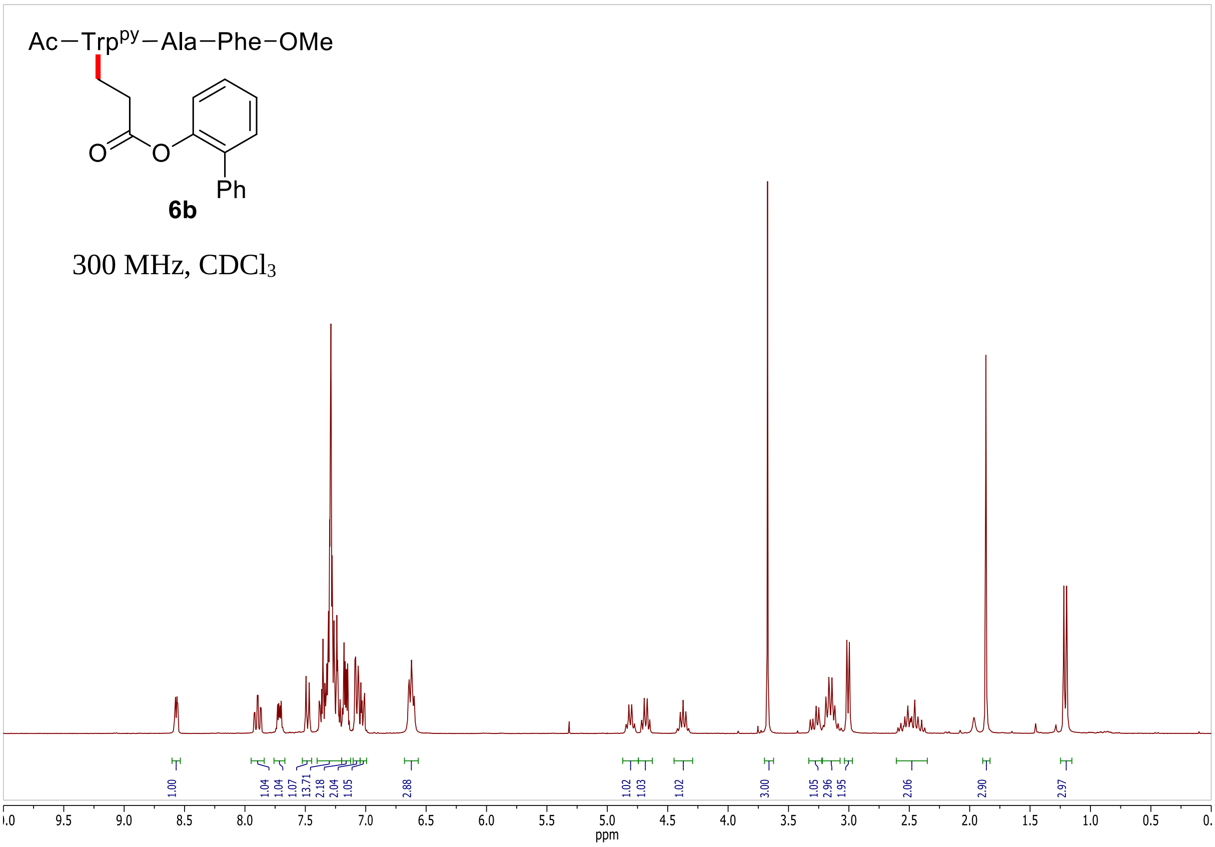

Supplementary Figure 90. ^{1}H-NMR spectrum of **6b**.

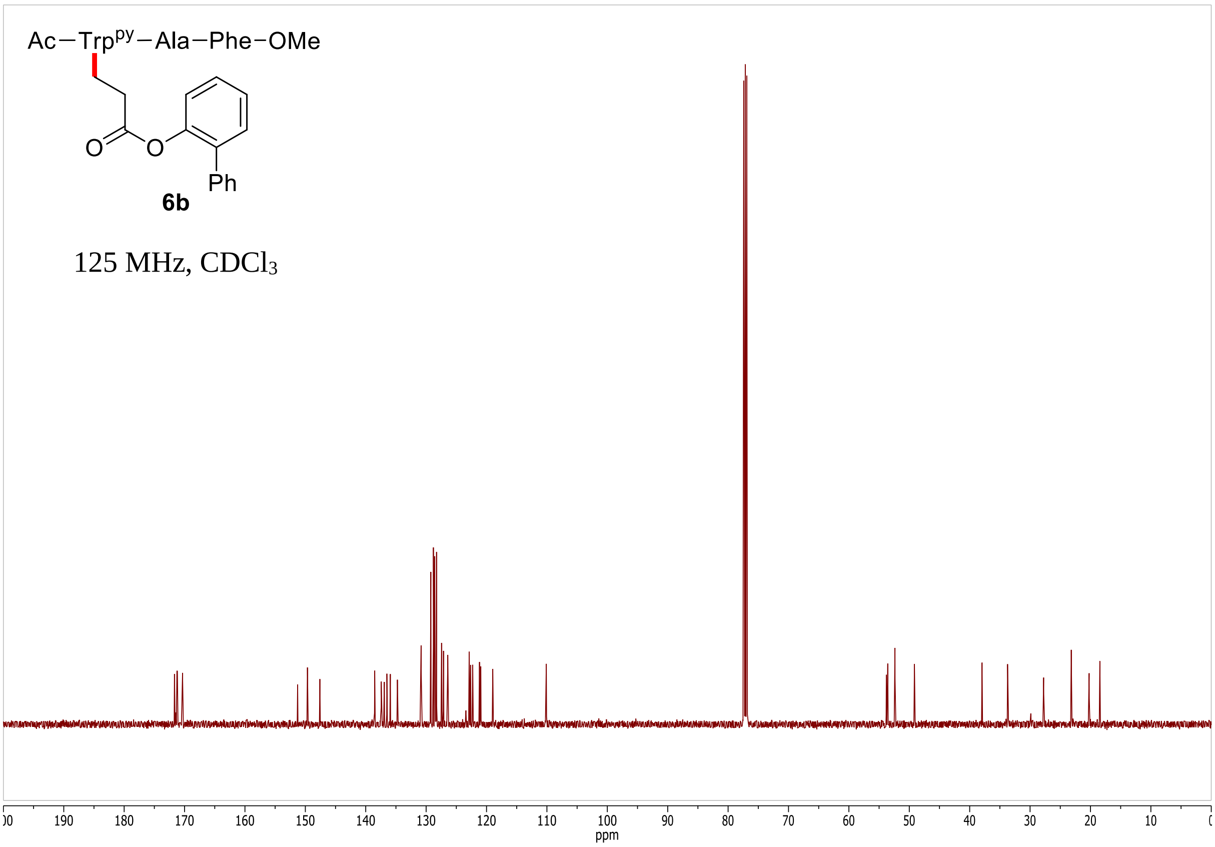

Supplementary Figure 91. ^{13}C-NMR spectrum of **6b**.

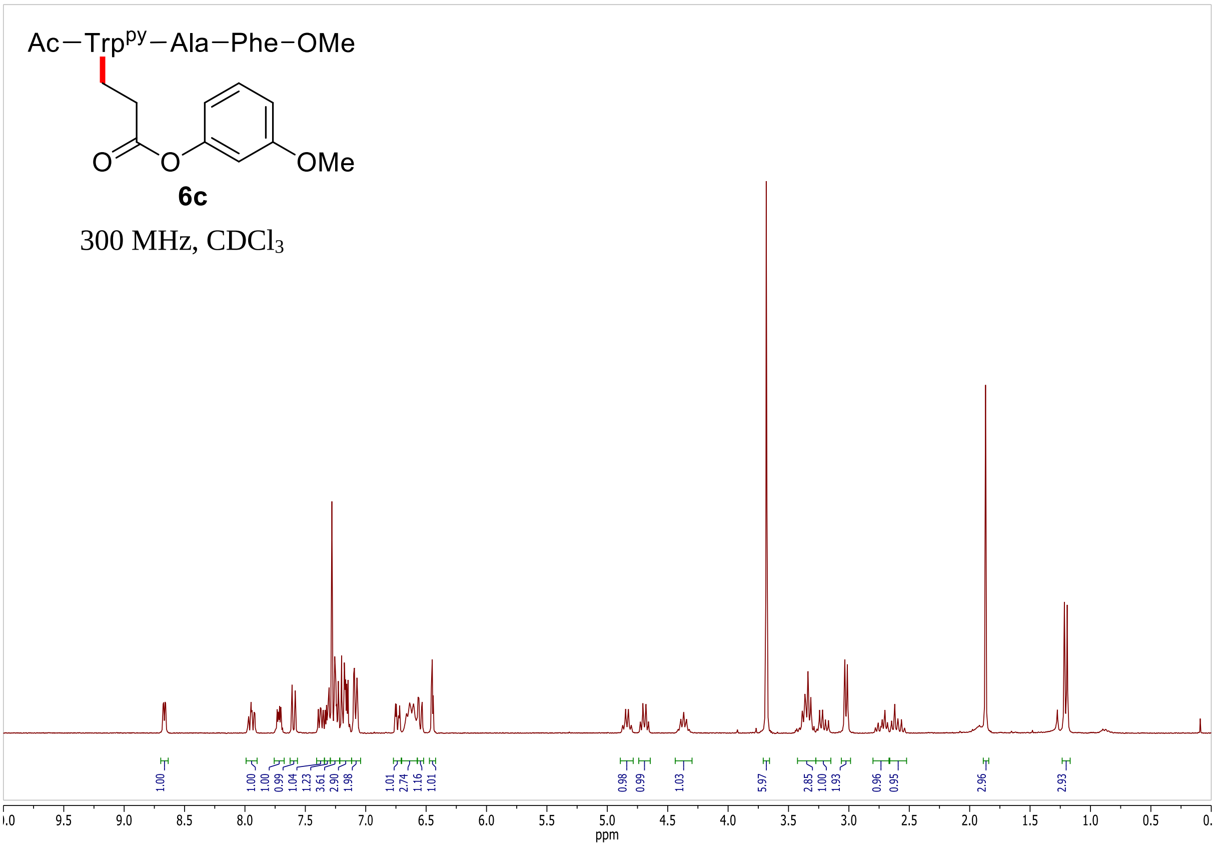

Supplementary Figure 92. ^{1}H-NMR spectrum of **6c**.

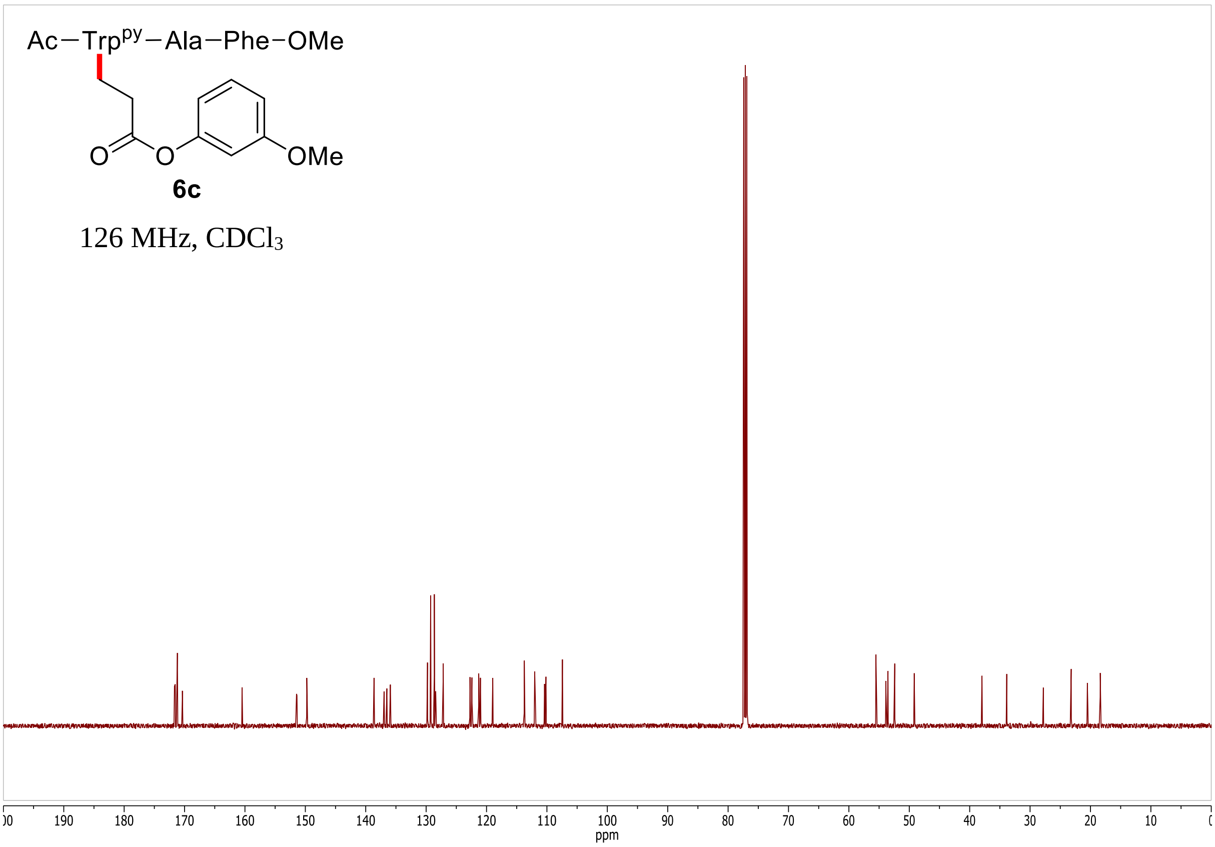

Supplementary Figure 93. ^{13}C-NMR spectrum of **6c**.

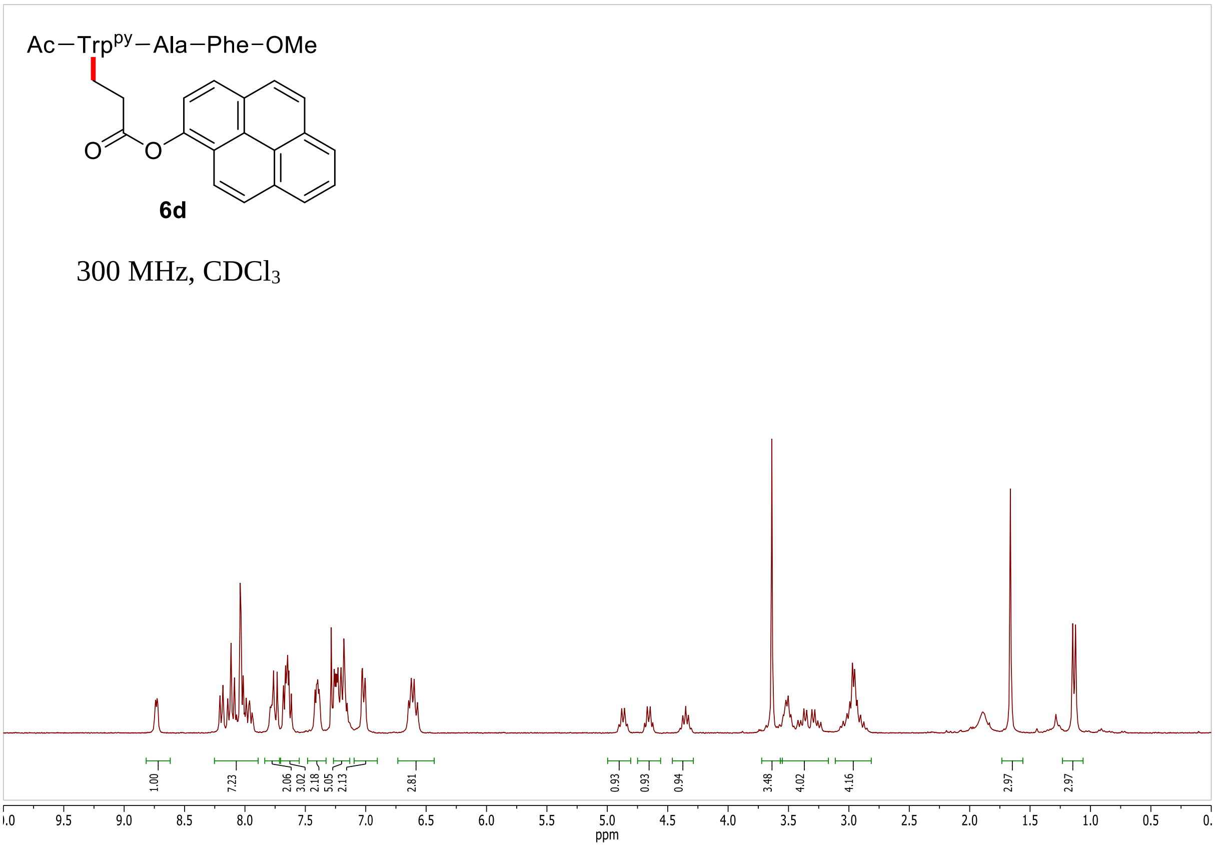

Supplementary Figure 94. ^{1}H-NMR spectrum of **6d**.

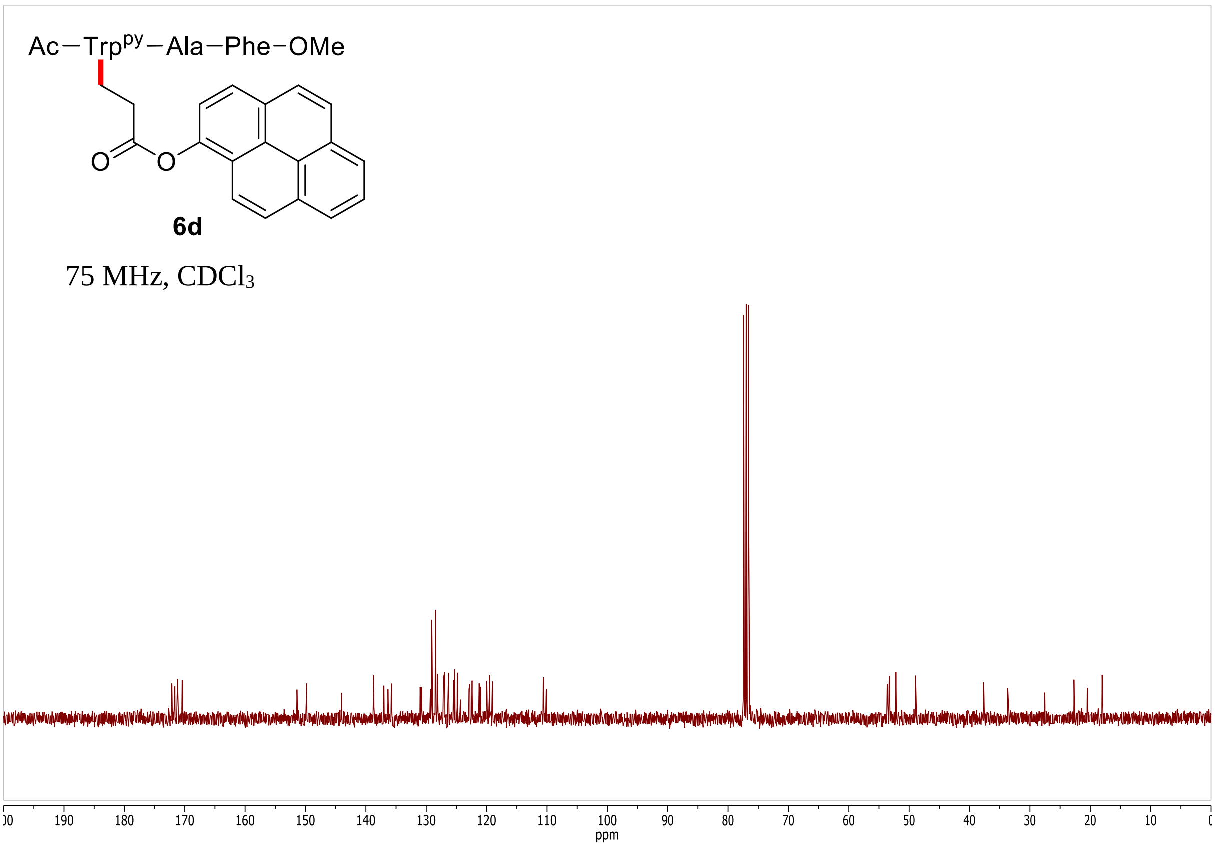

Supplementary Figure 95. ^{13}C-NMR spectrum of **6d**.

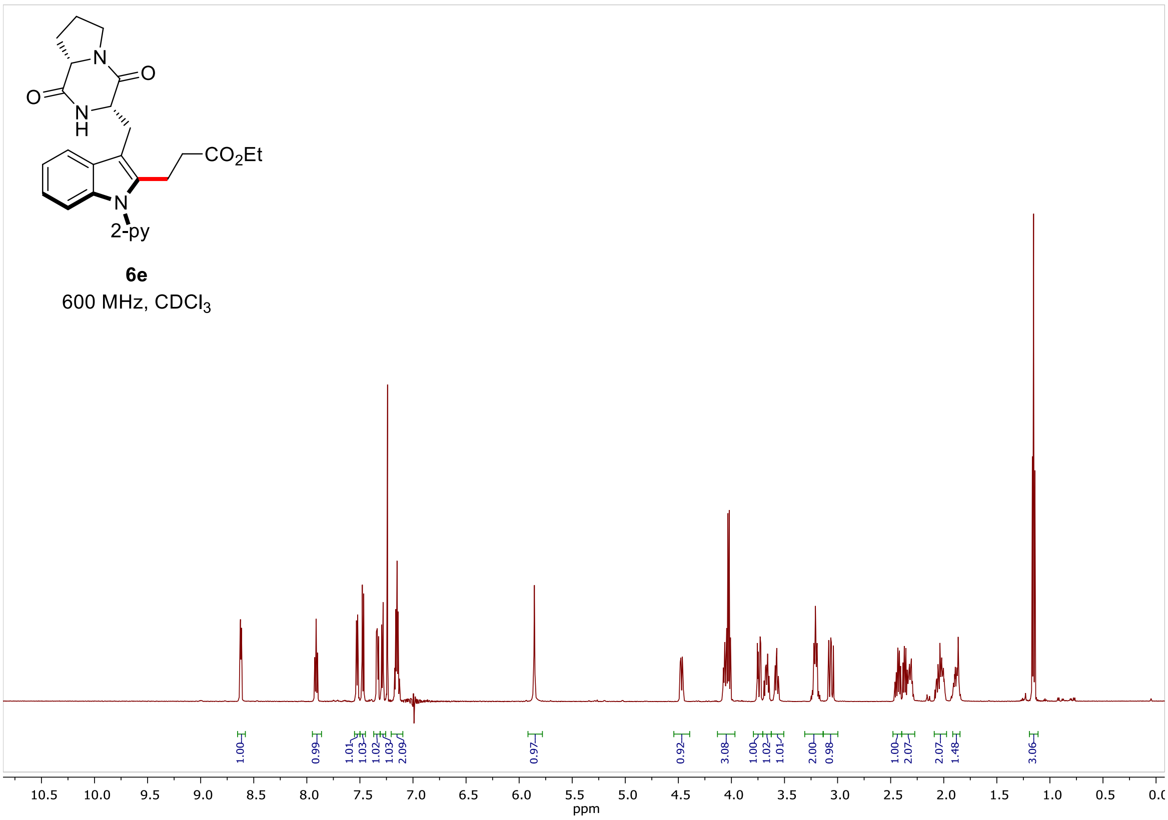

Supplementary Figure 96. ^{1}H-NMR spectrum of **6e**.

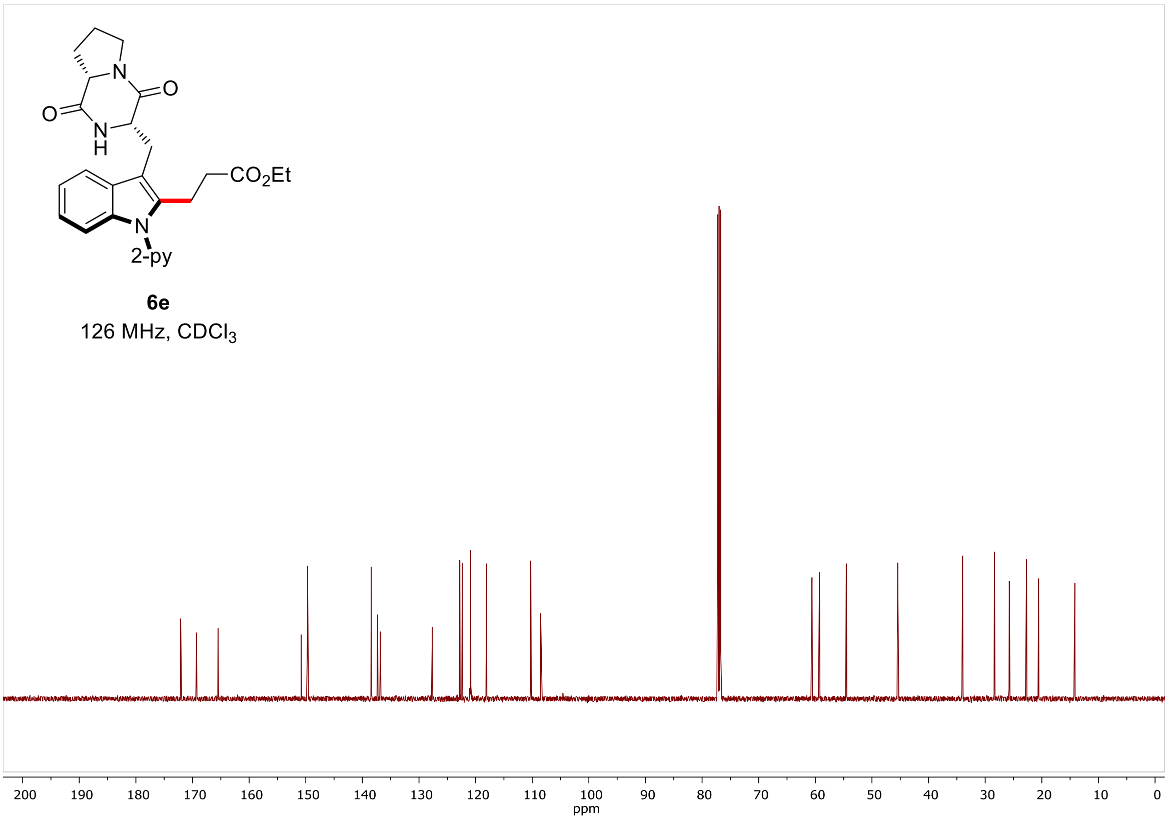

Supplementary Figure 97. ^{13}C-NMR spectrum of **6e**.

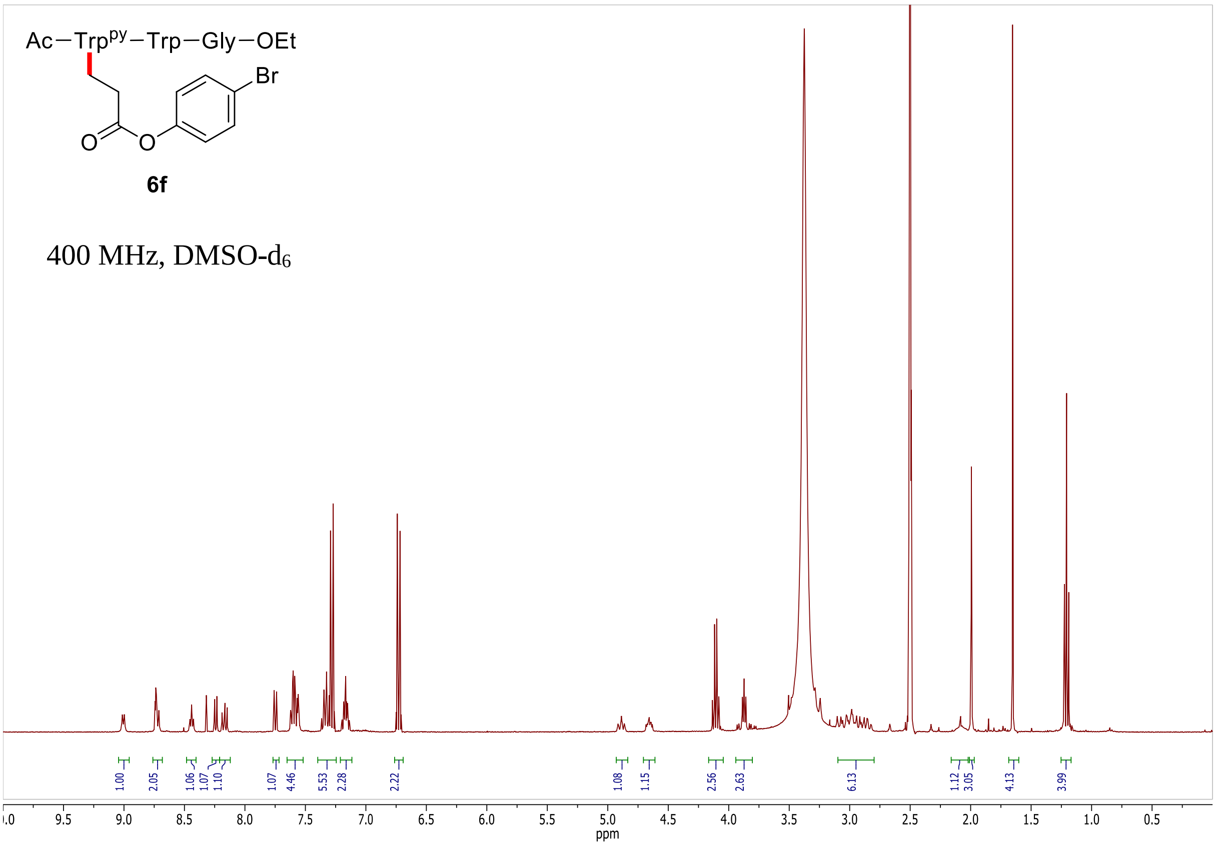

Supplementary Figure 98. ^{1}H-NMR spectrum of **6f**.

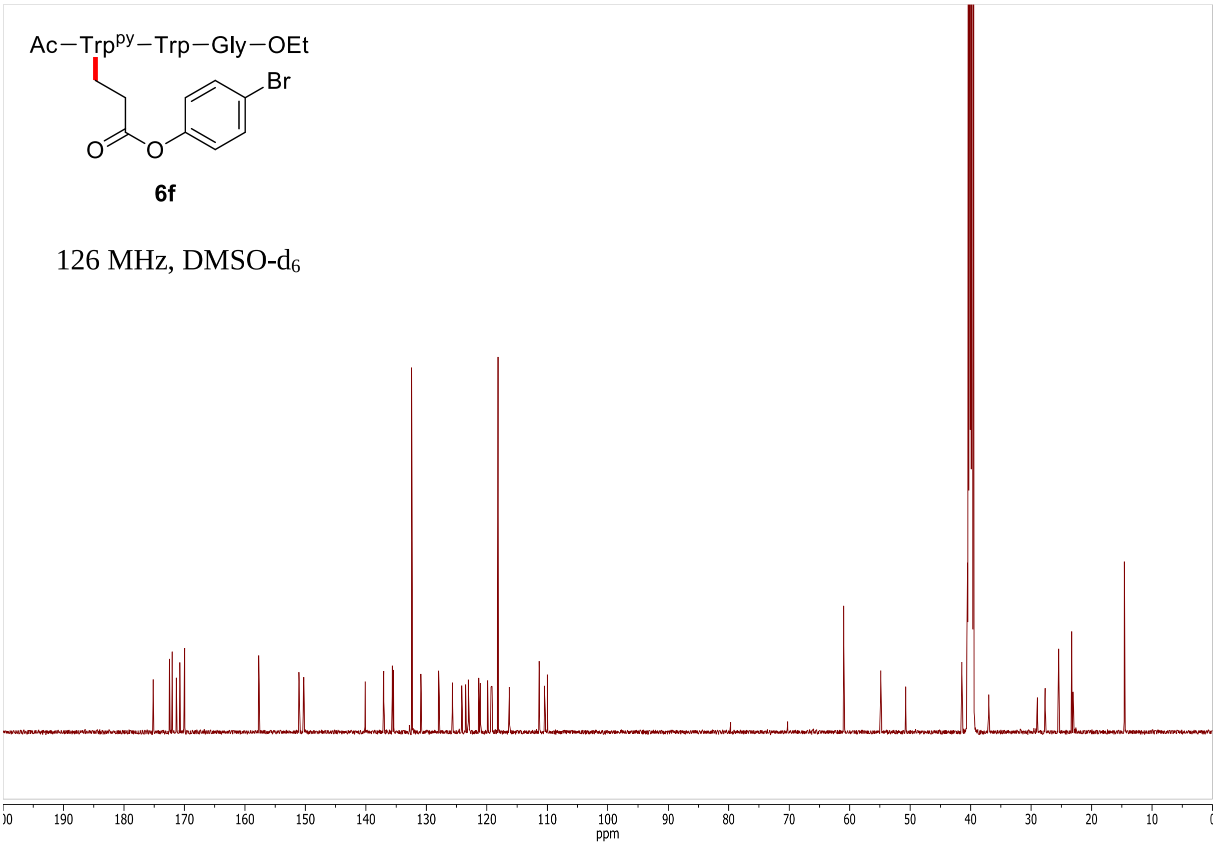

Supplementary Figure 99. ^{13}C-NMR spectrum of **6f**.

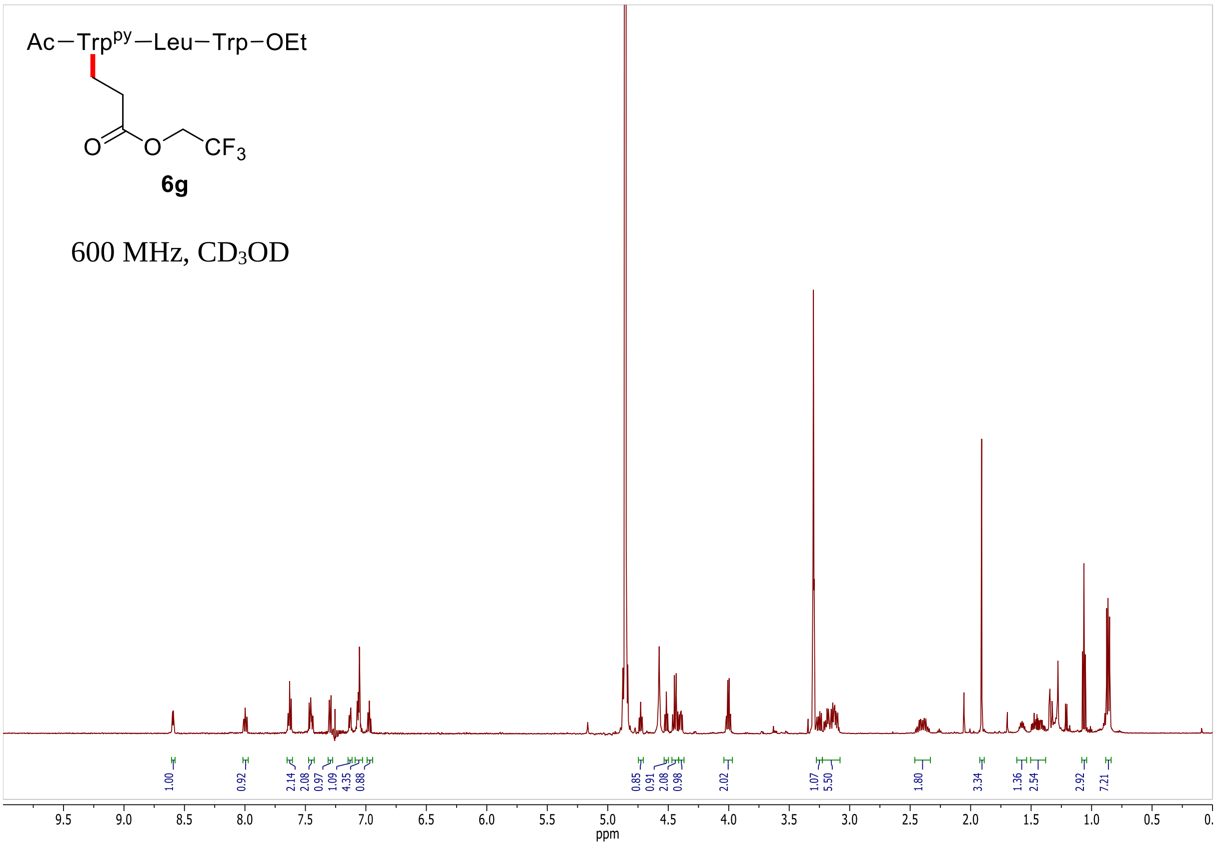

Supplementary Figure 100. ^{1}H-NMR spectrum of **6g**.

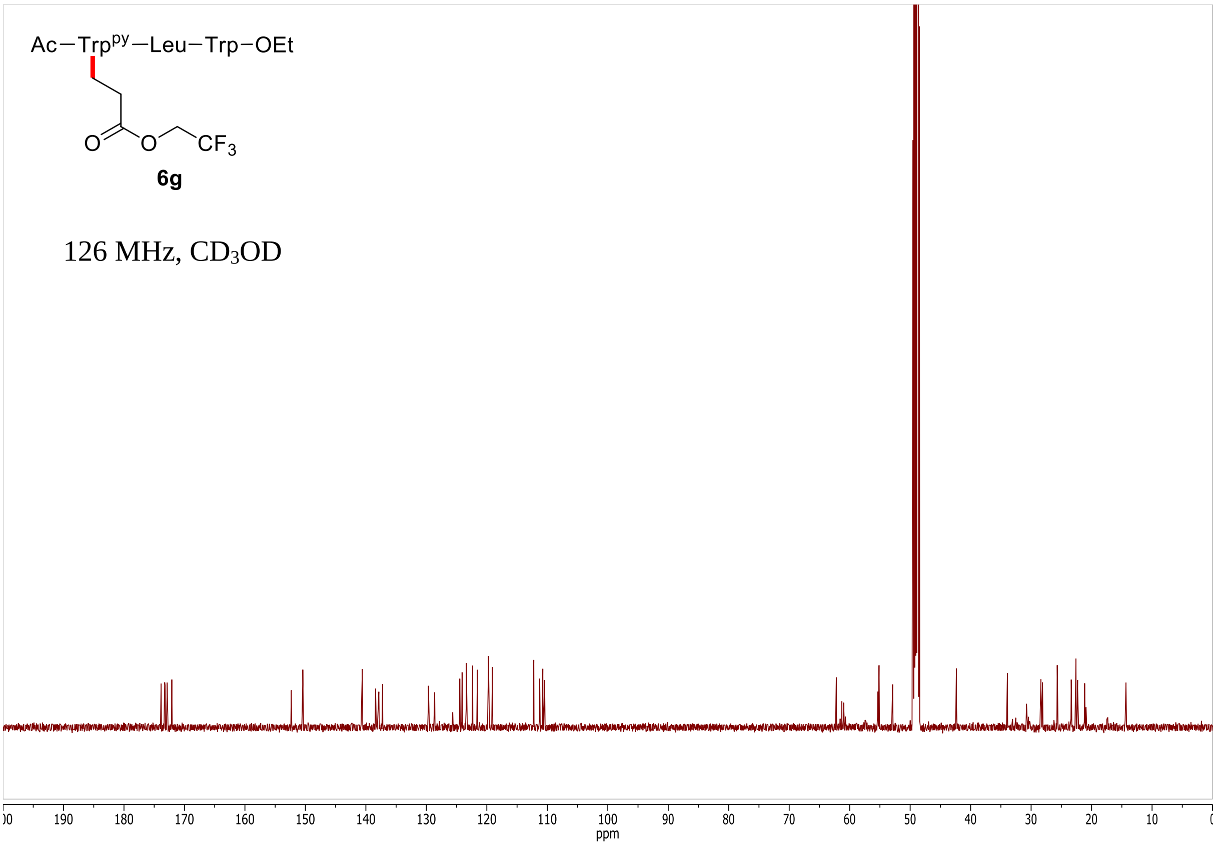

Supplementary Figure 101. ^{13}C-NMR spectrum of **6g**.

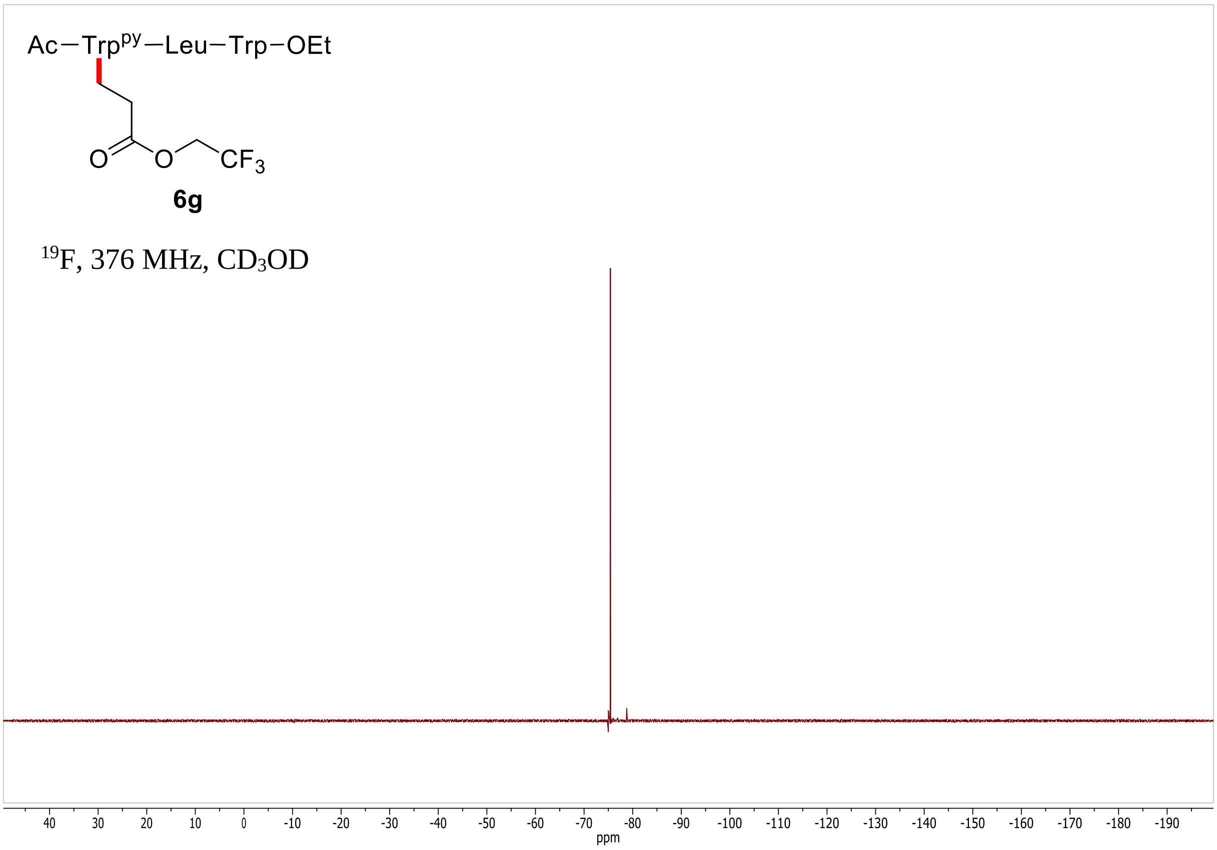

Supplementary Figure 102. ^{19}F-NMR spectrum of **6g**.

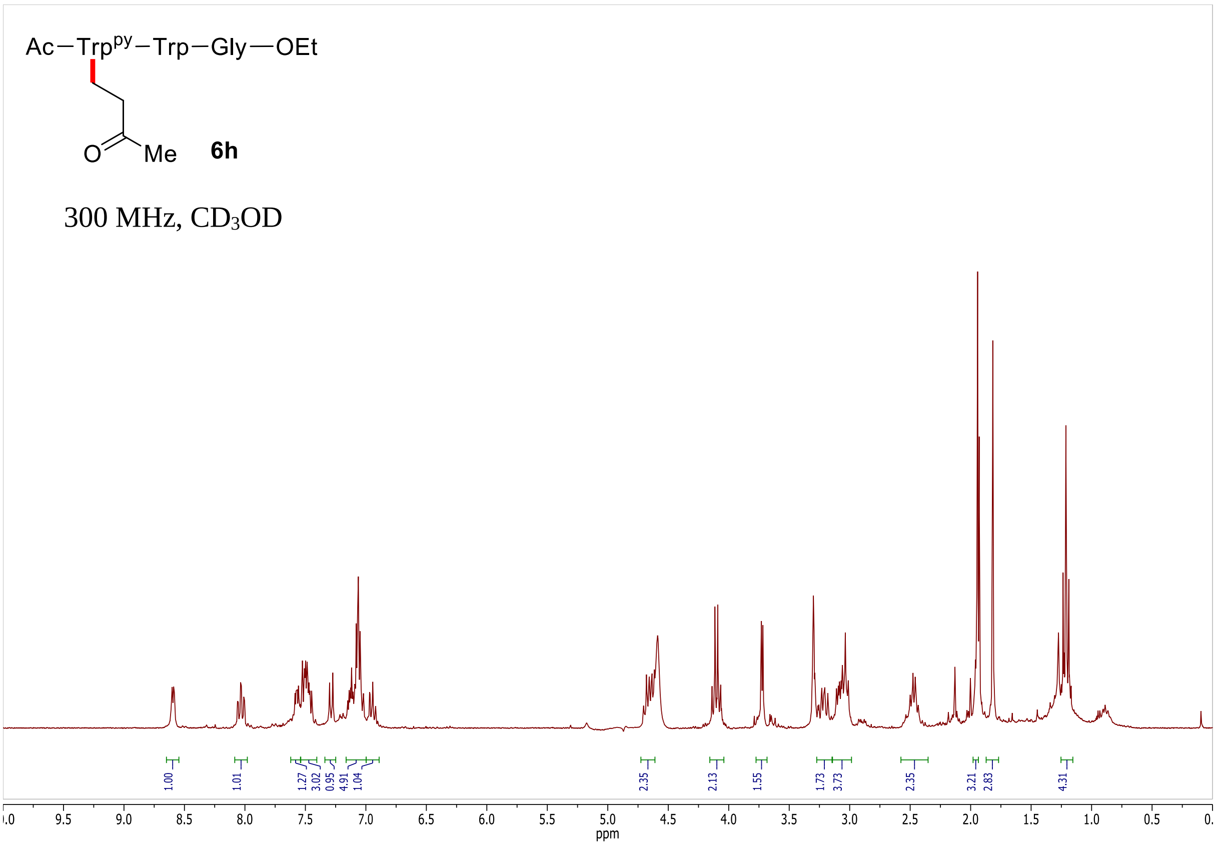

Supplementary Figure 103. ^{1}H-NMR spectrum of **6h**.

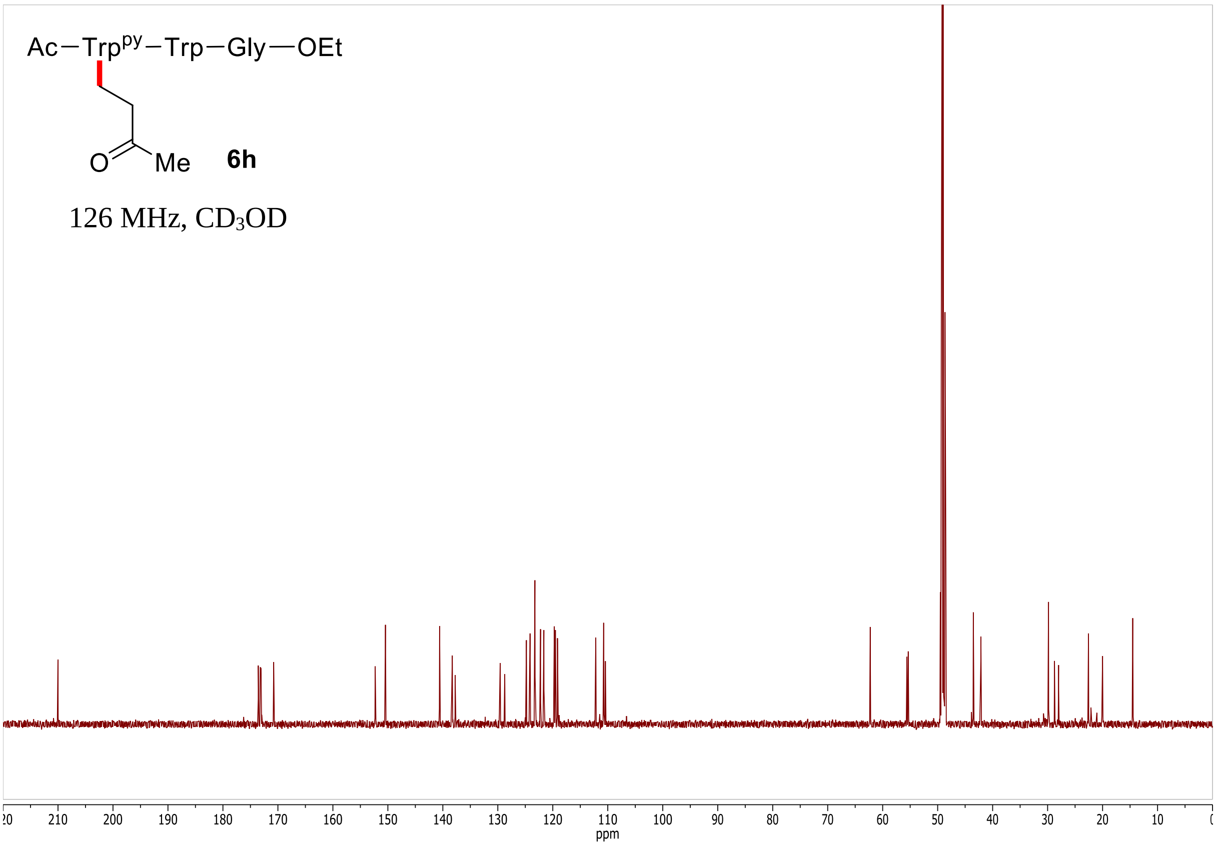

Supplementary Figure 104. ^{13}C-NMR spectrum of **6h**.

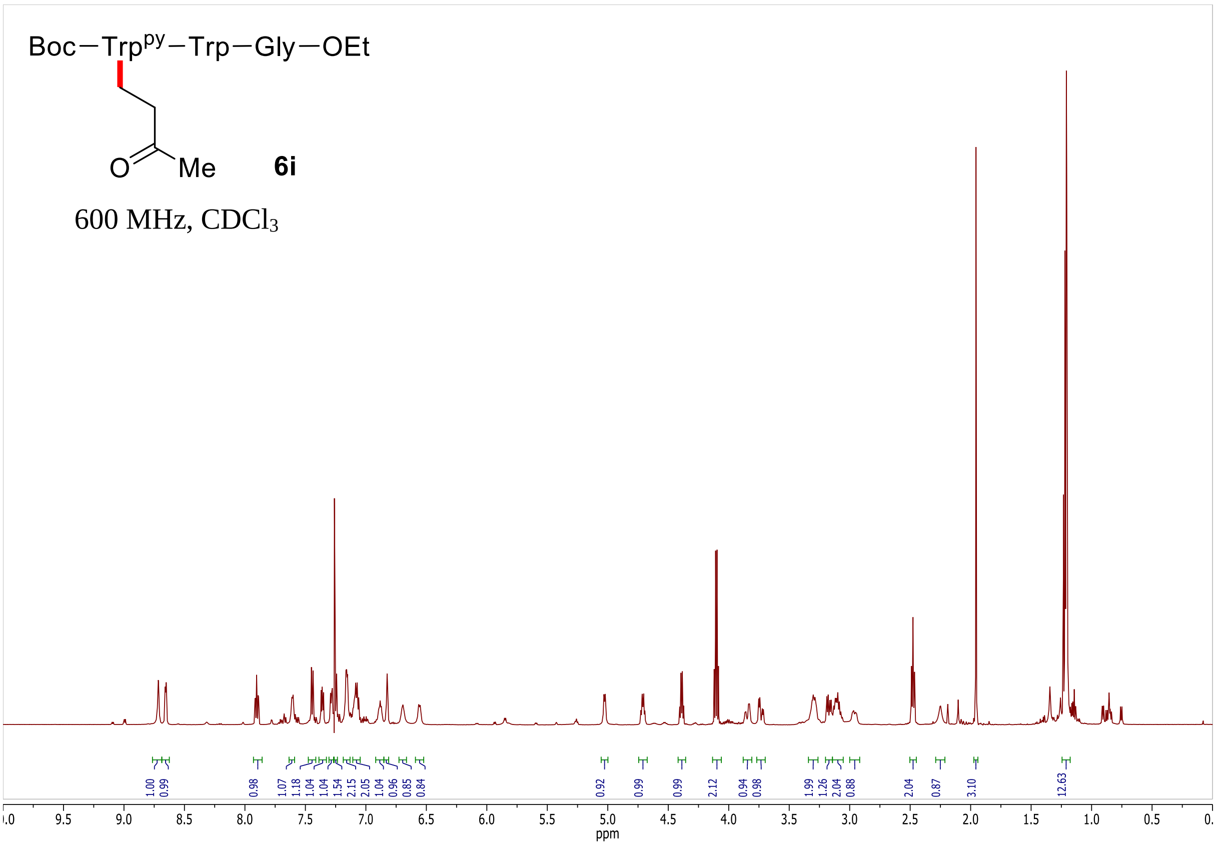

Supplementary Figure 105. ^{1}H-NMR spectrum of **6i**.

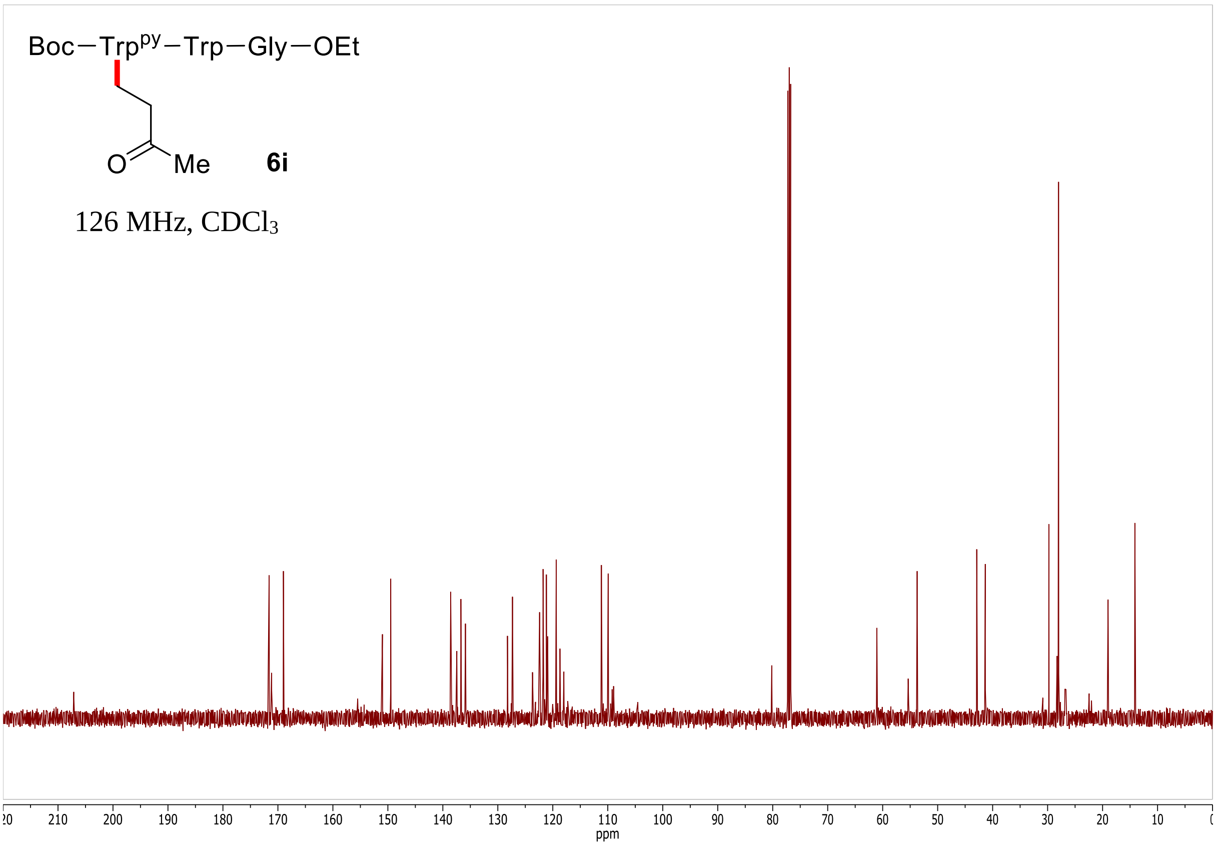

Supplementary Figure 106. ^{13}C-NMR spectrum of **6i**.

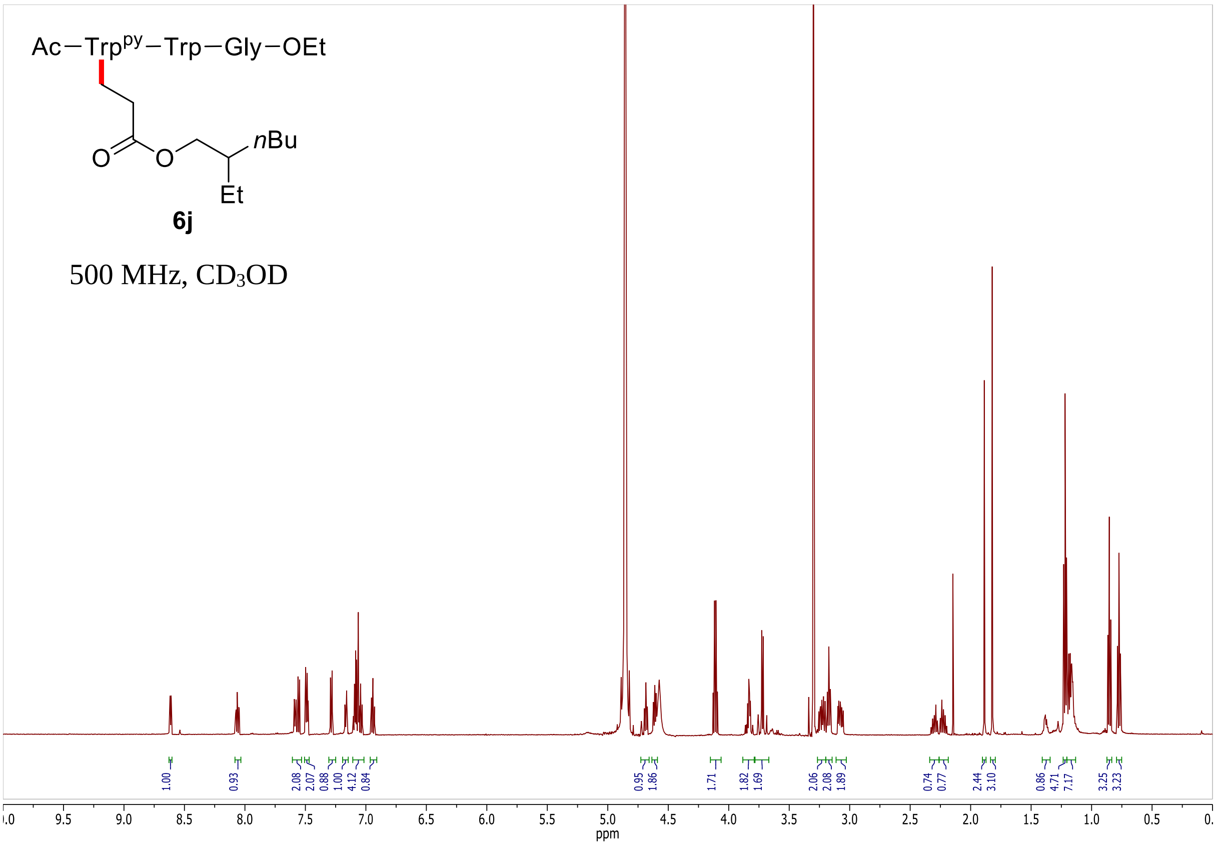

Supplementary Figure 107. ^{1}H-NMR spectrum of **6j**.

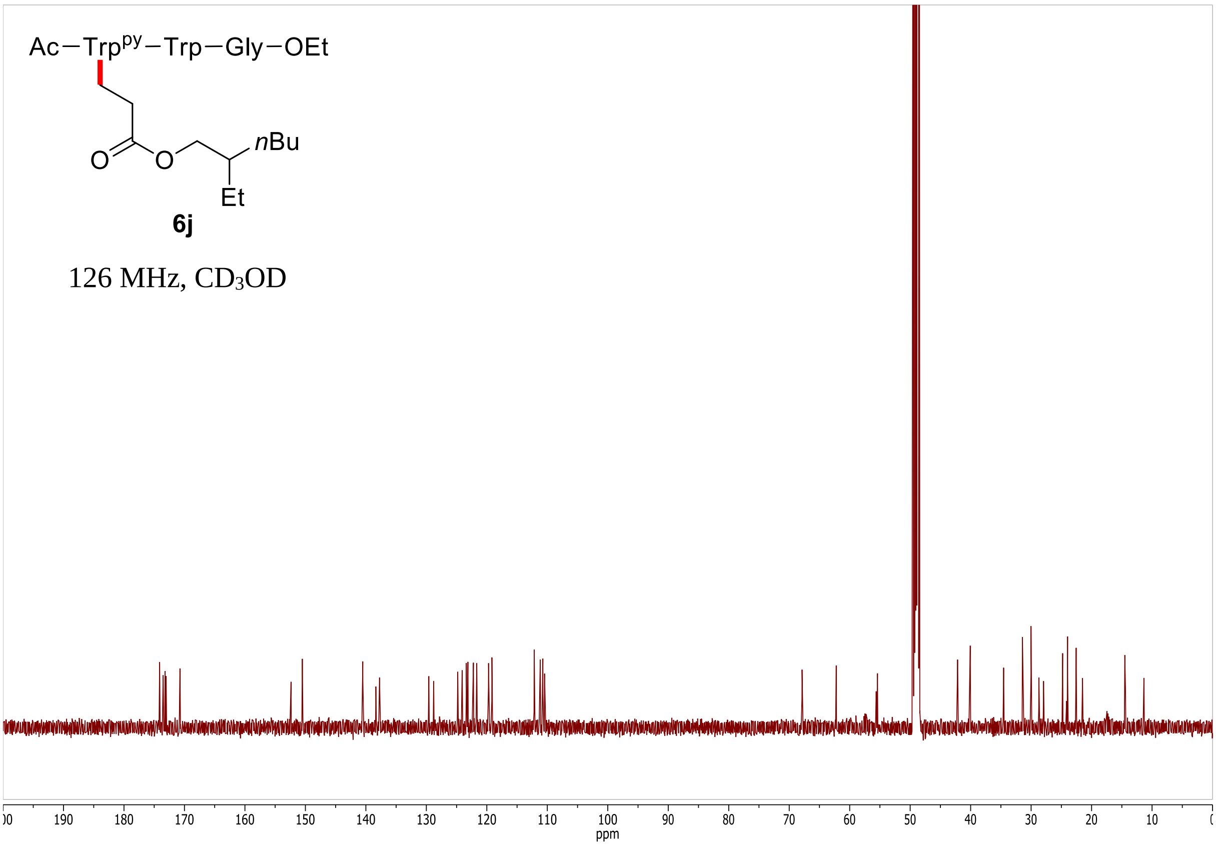

Supplementary Figure 108. ^{13}C-NMR spectrum of **6j**.

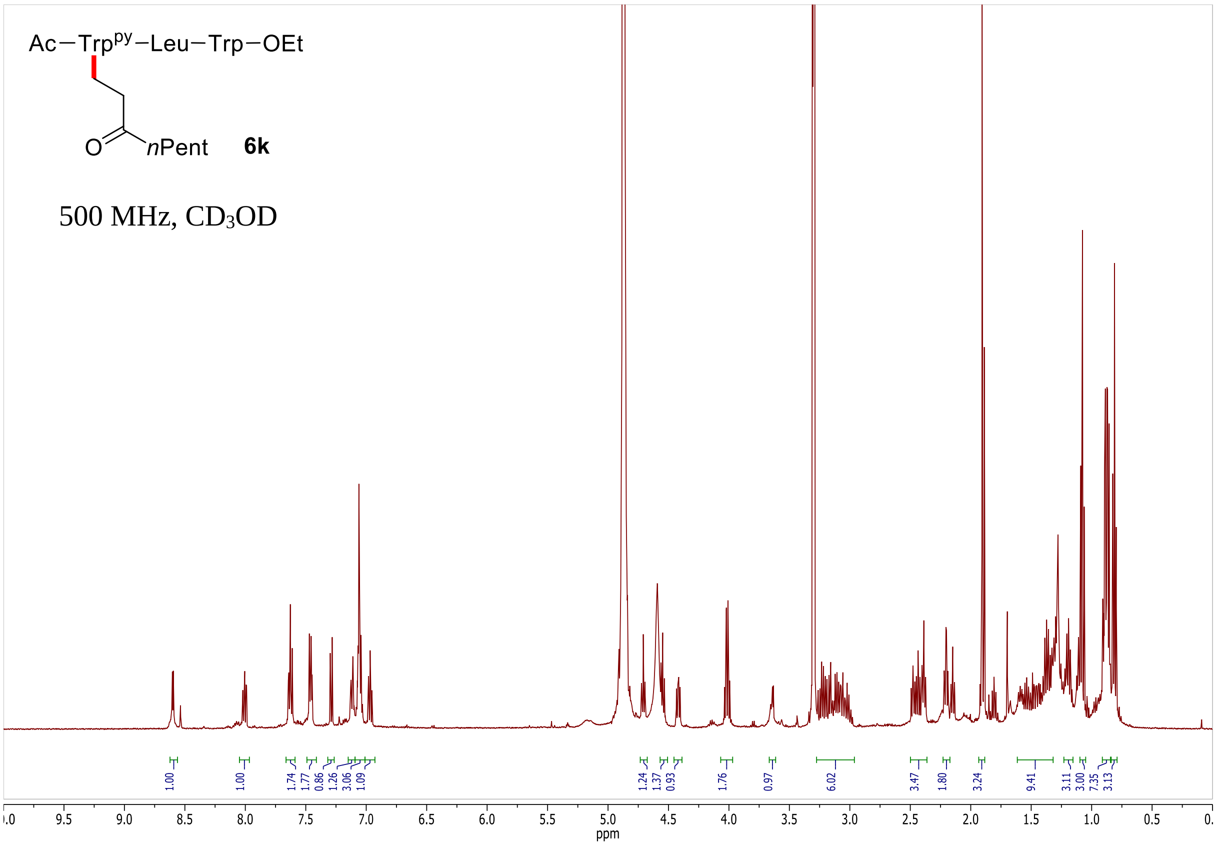

Supplementary Figure 109. ^{1}H-NMR spectrum of **6k**.

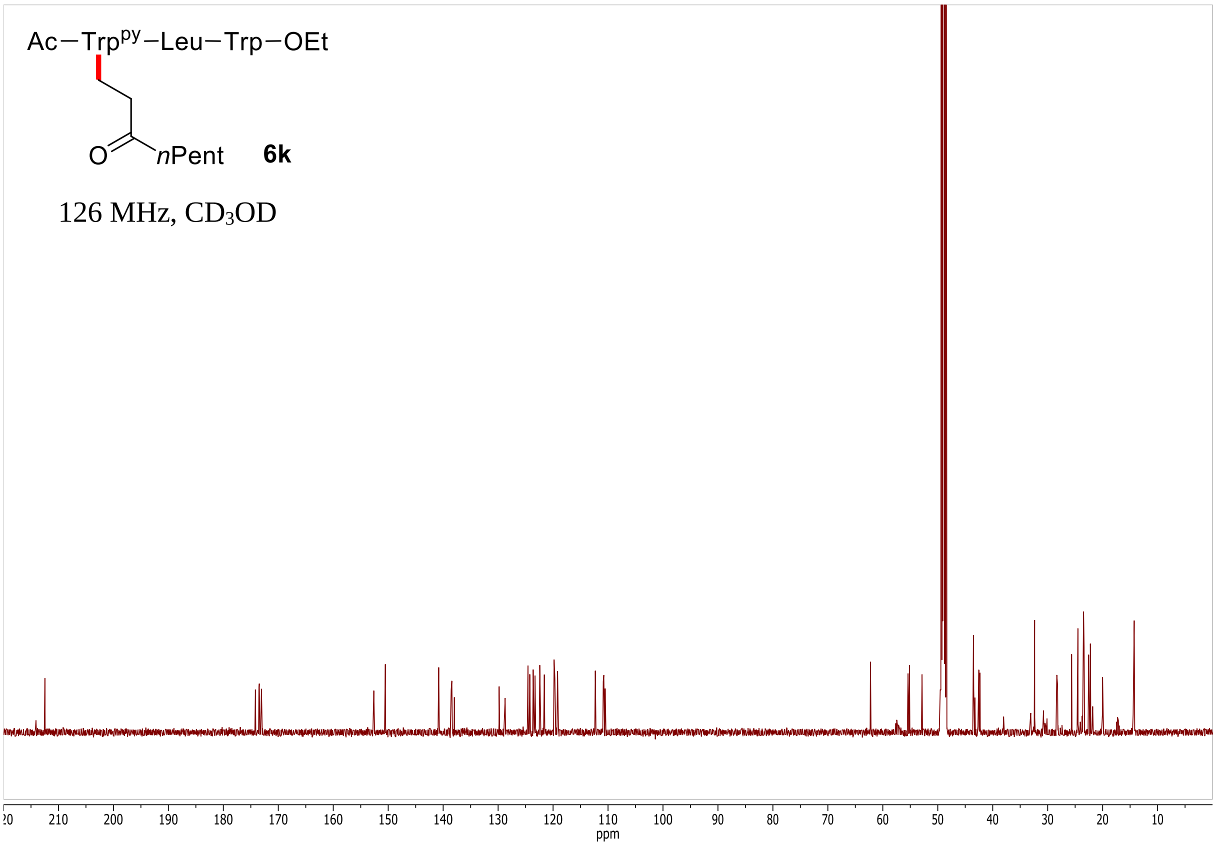

Supplementary Figure 110. ^{13}C-NMR spectrum of **6k**.

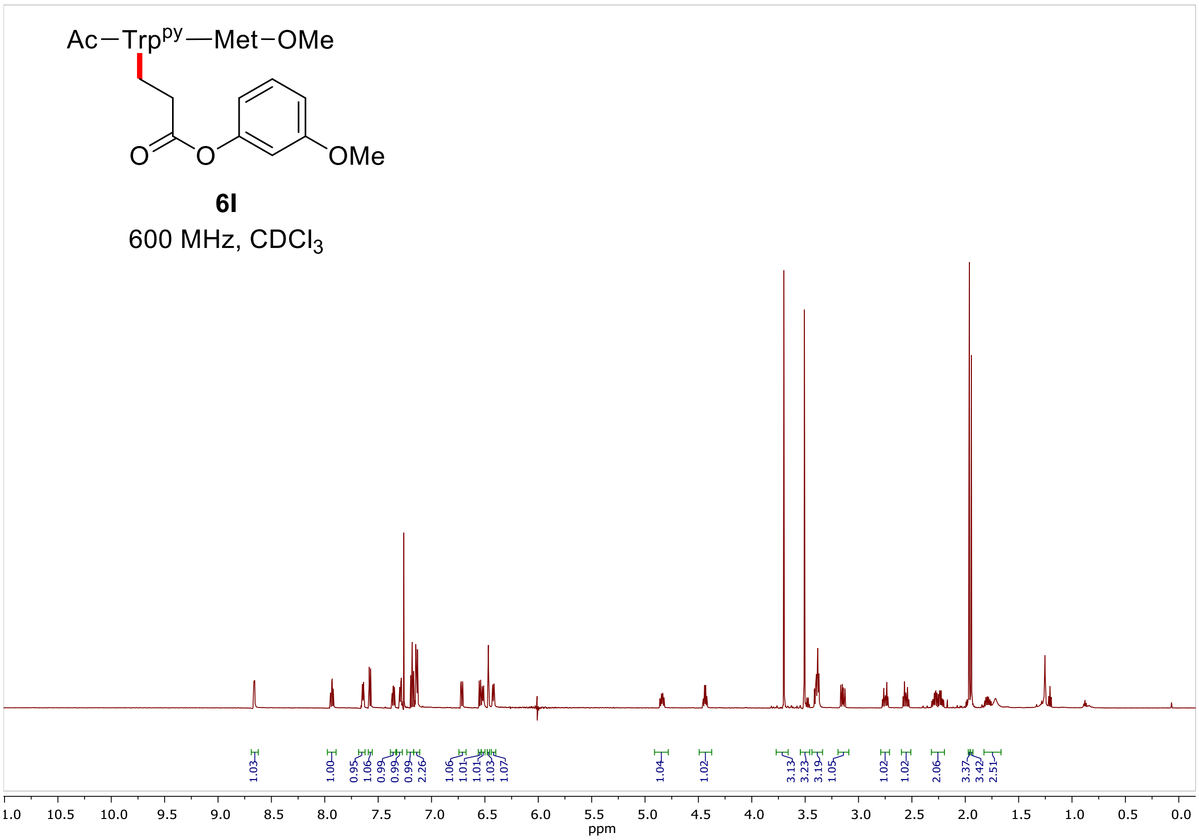

Supplementary Figure 111. ^{1}H-NMR spectrum of **6l**.

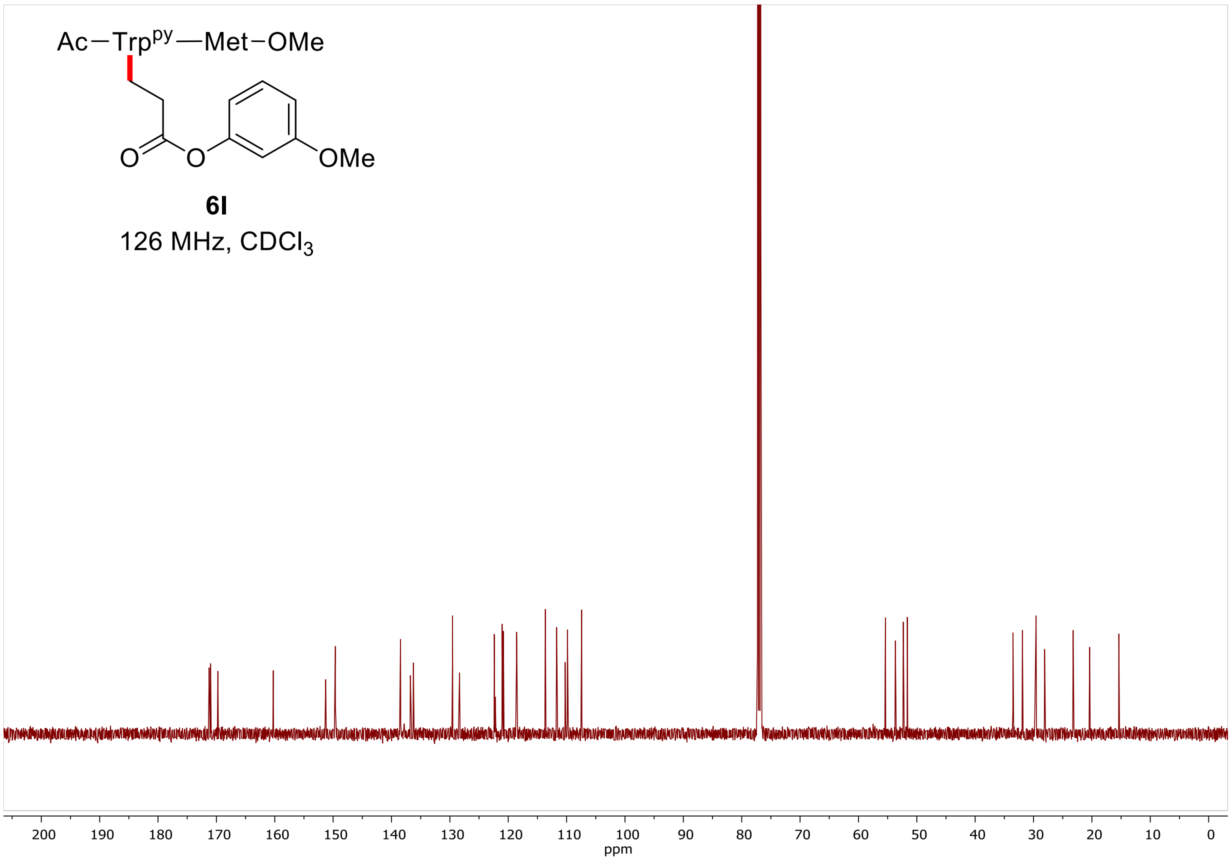

Supplementary Figure 112. ^{13}C-NMR spectrum of **6l**.

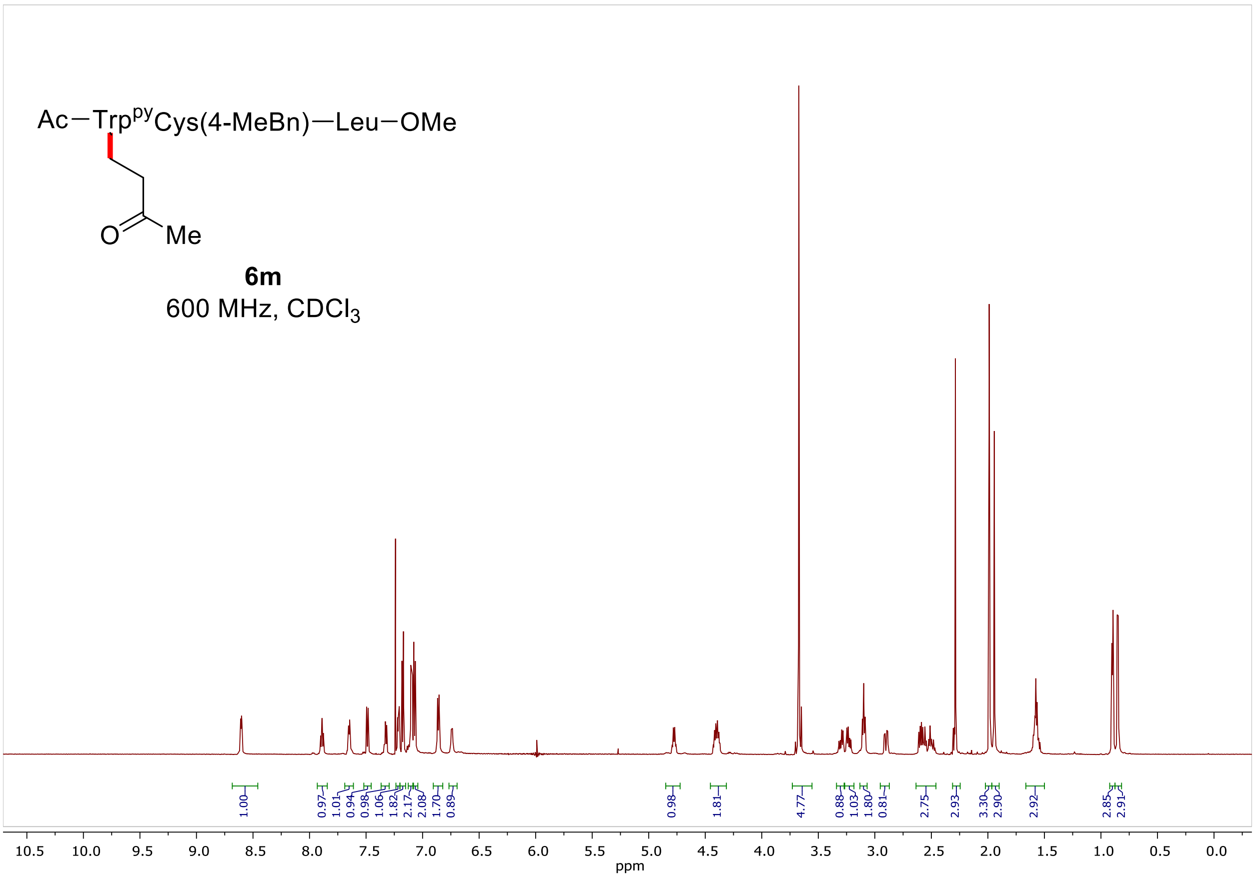

Supplementary Figure 113. ^{1}H-NMR spectrum of **6m**.

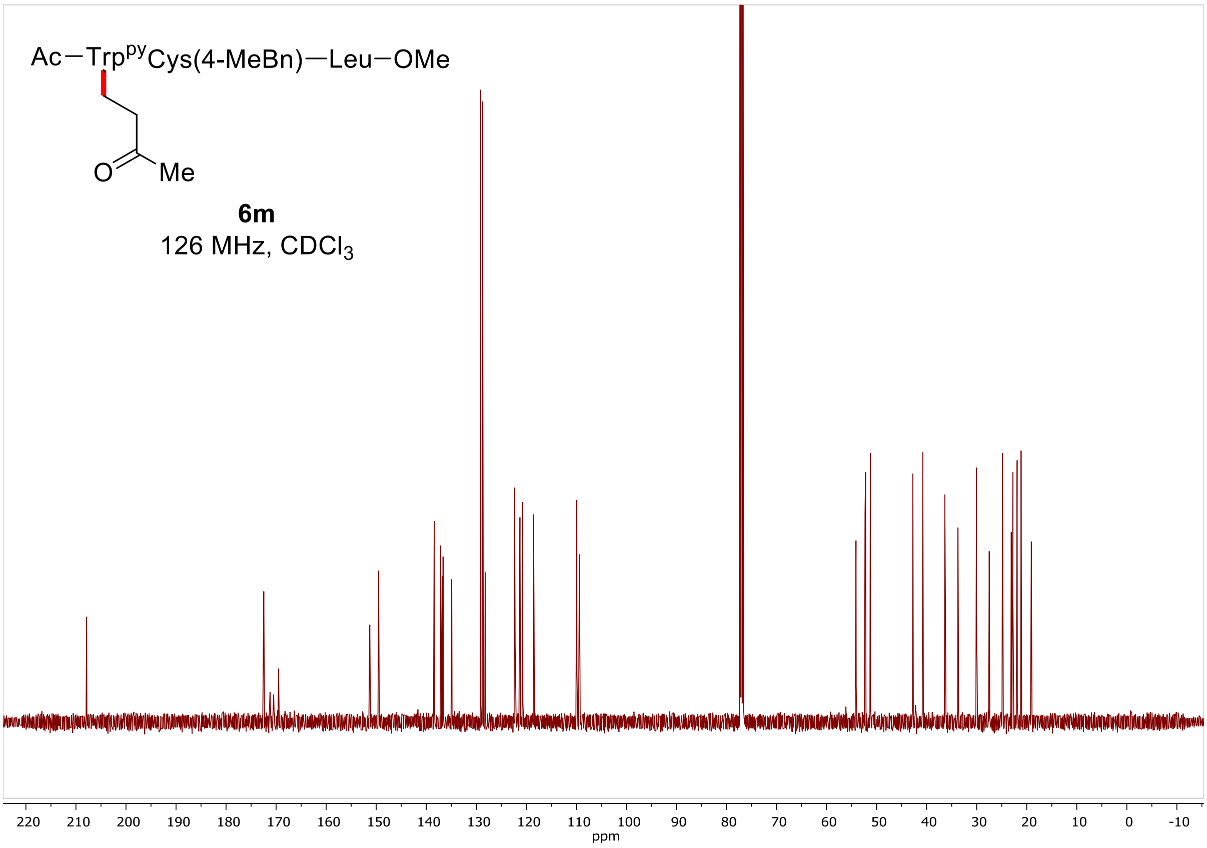

Supplementary Figure 114. ^{13}C-NMR spectrum of **6m**.

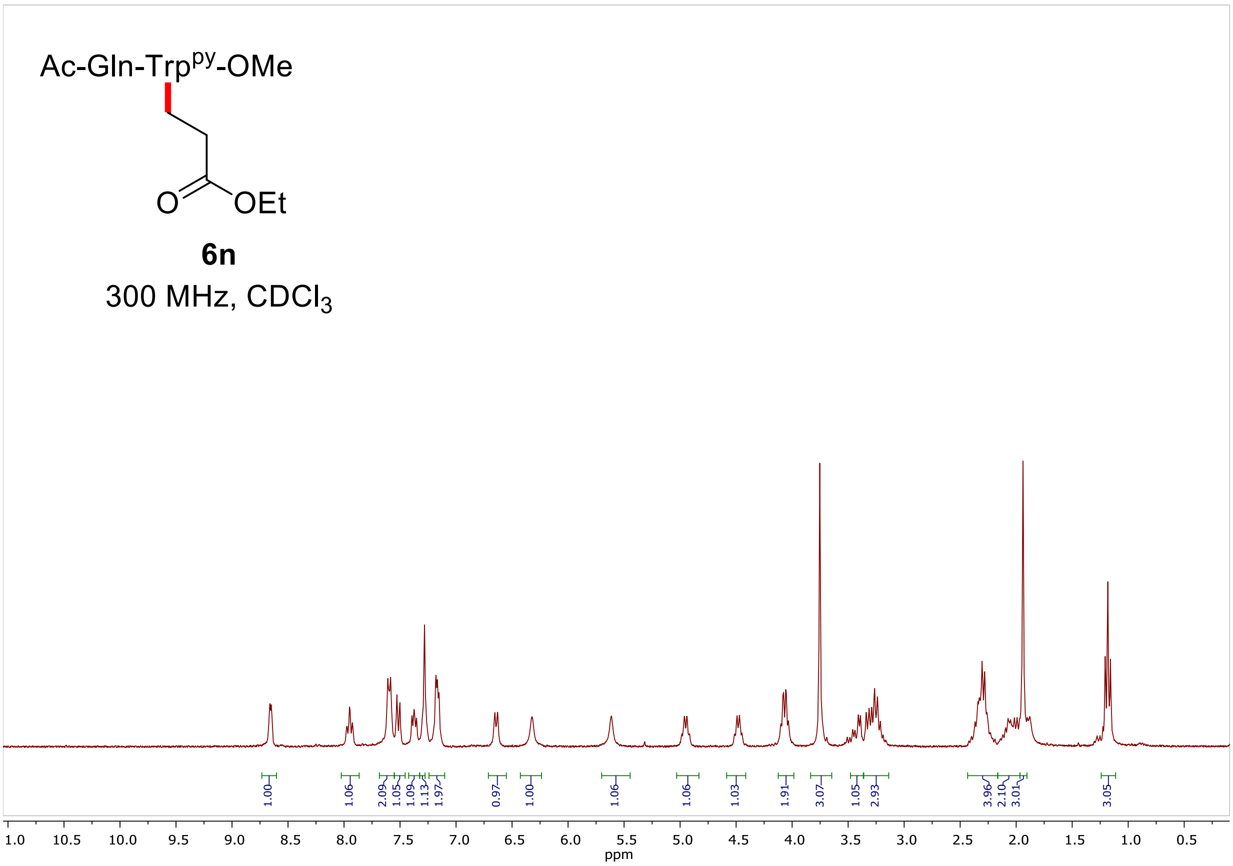

Supplementary Figure 115. ^{1}H-NMR spectrum of **6n**.

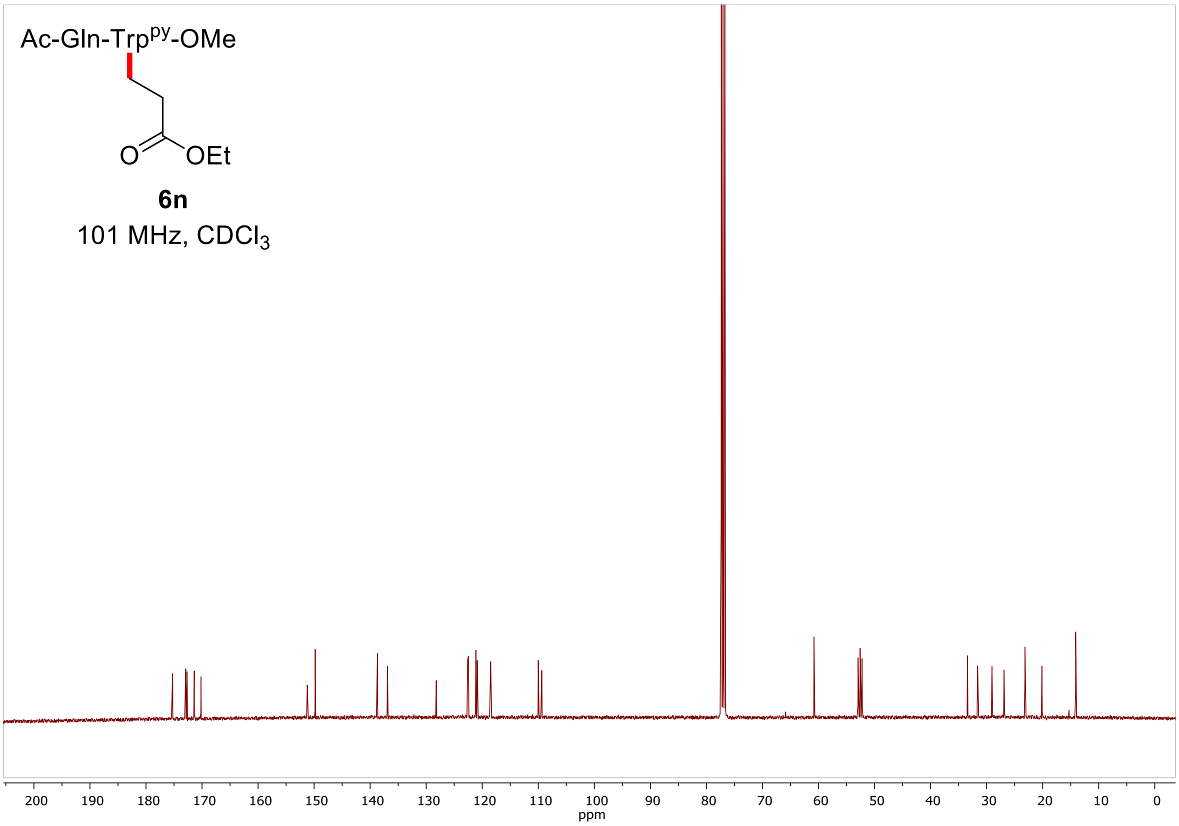

Supplementary Figure 116. ^{13}C-NMR spectrum of **6n**.

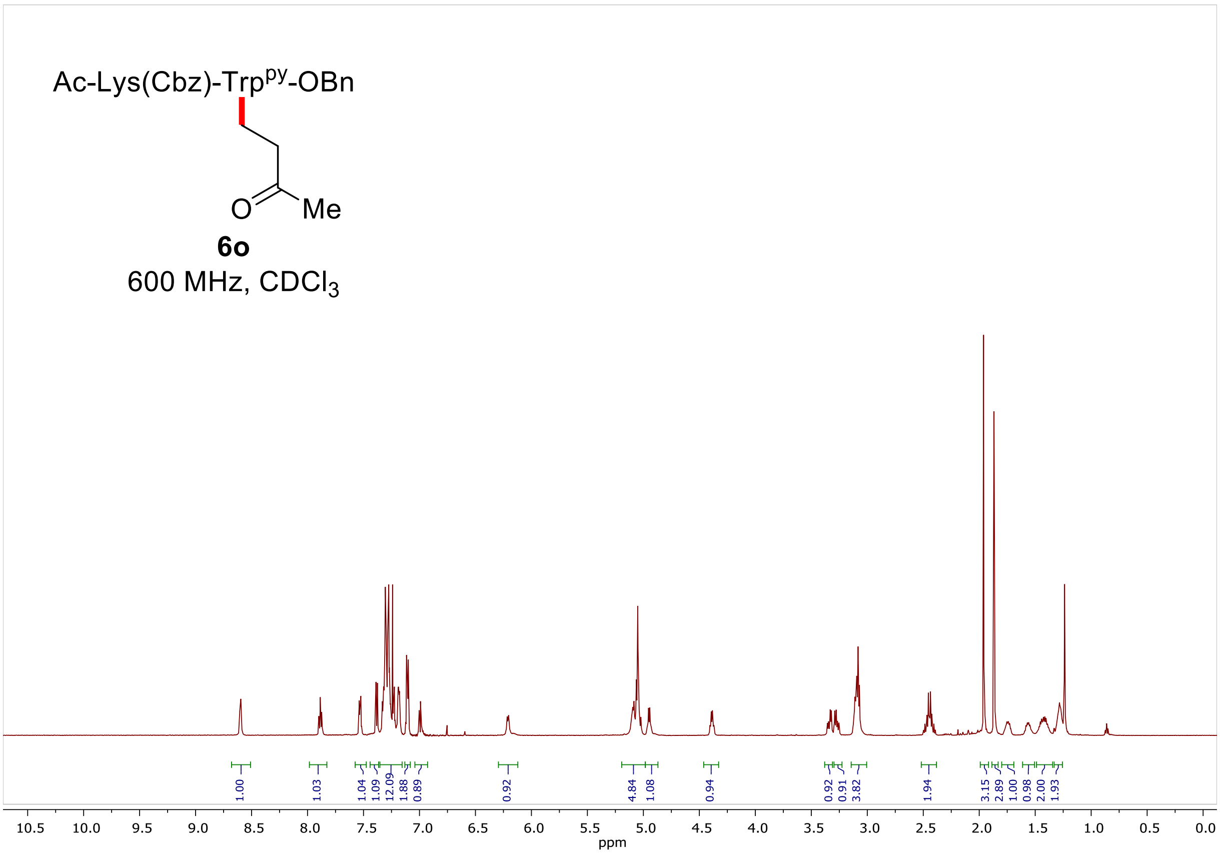

Supplementary Figure 117. ^{1}H-NMR spectrum of **6o**.

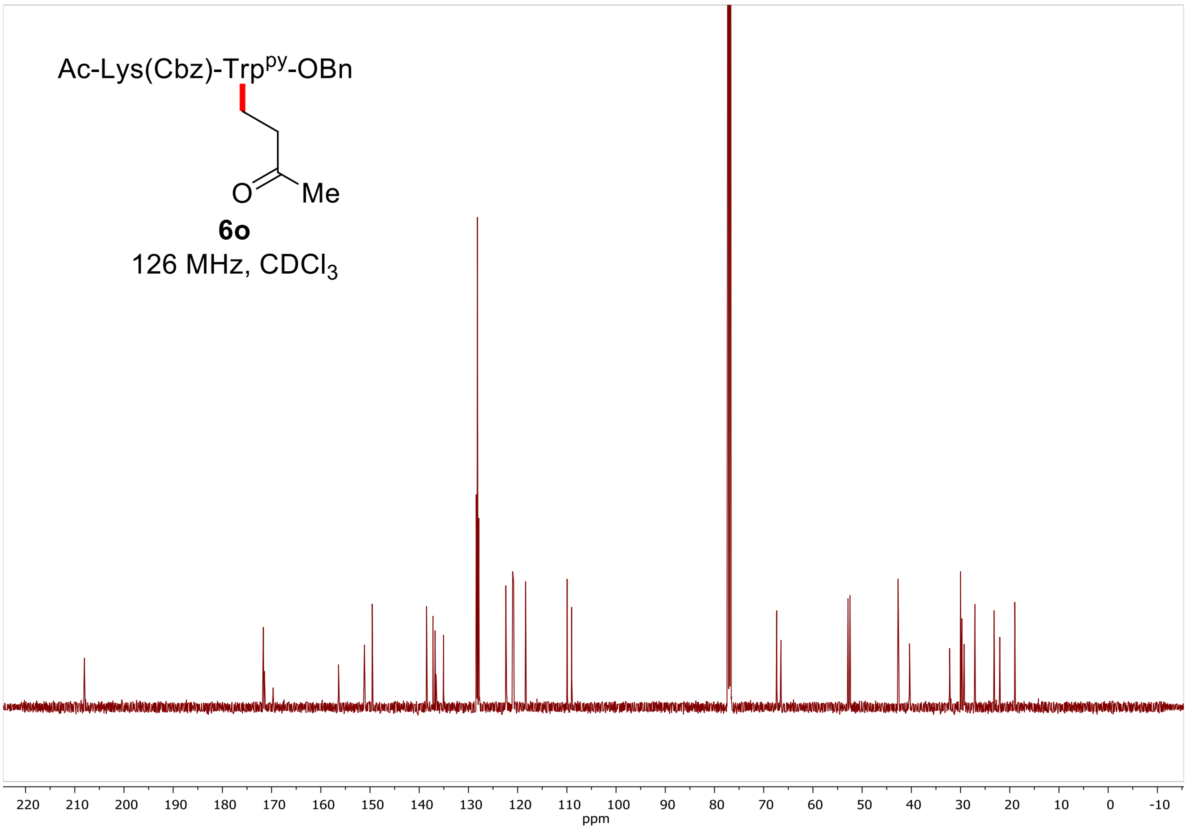

Supplementary Figure 118. ^{13}C-NMR spectrum of **6o**.

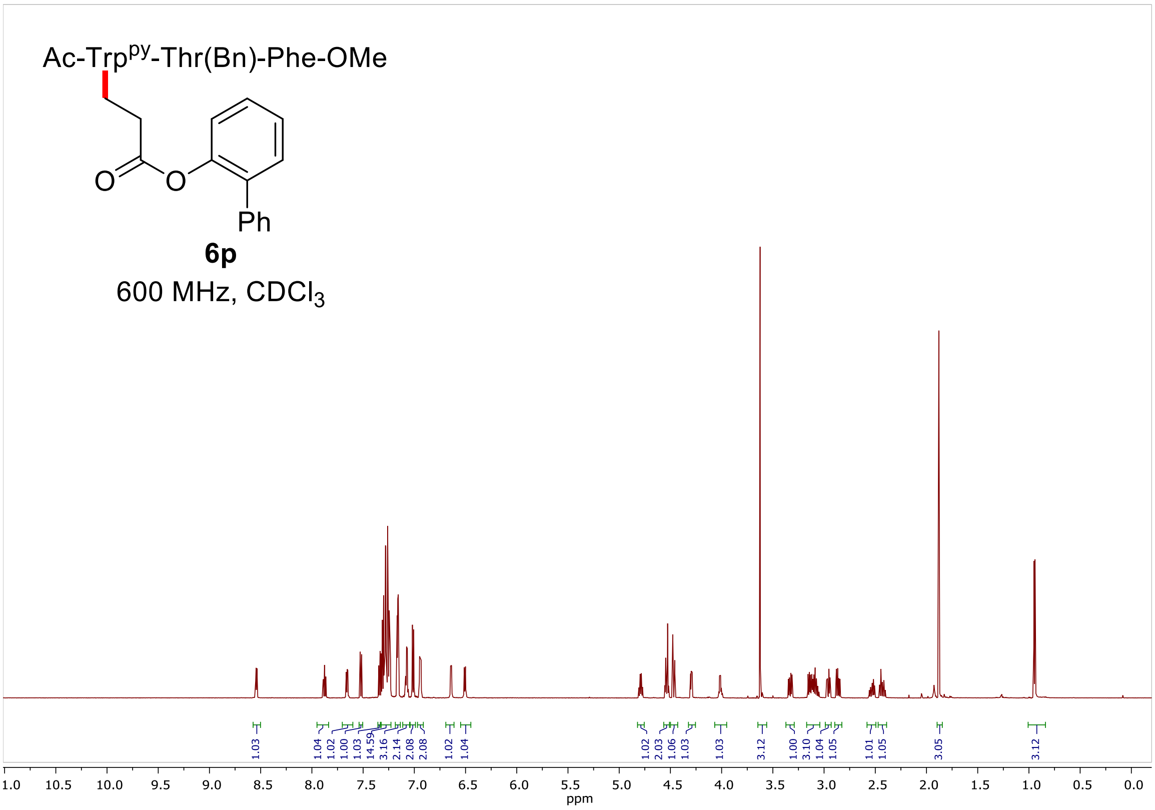

Supplementary Figure 119. ^{1}H-NMR spectrum of **6p**.

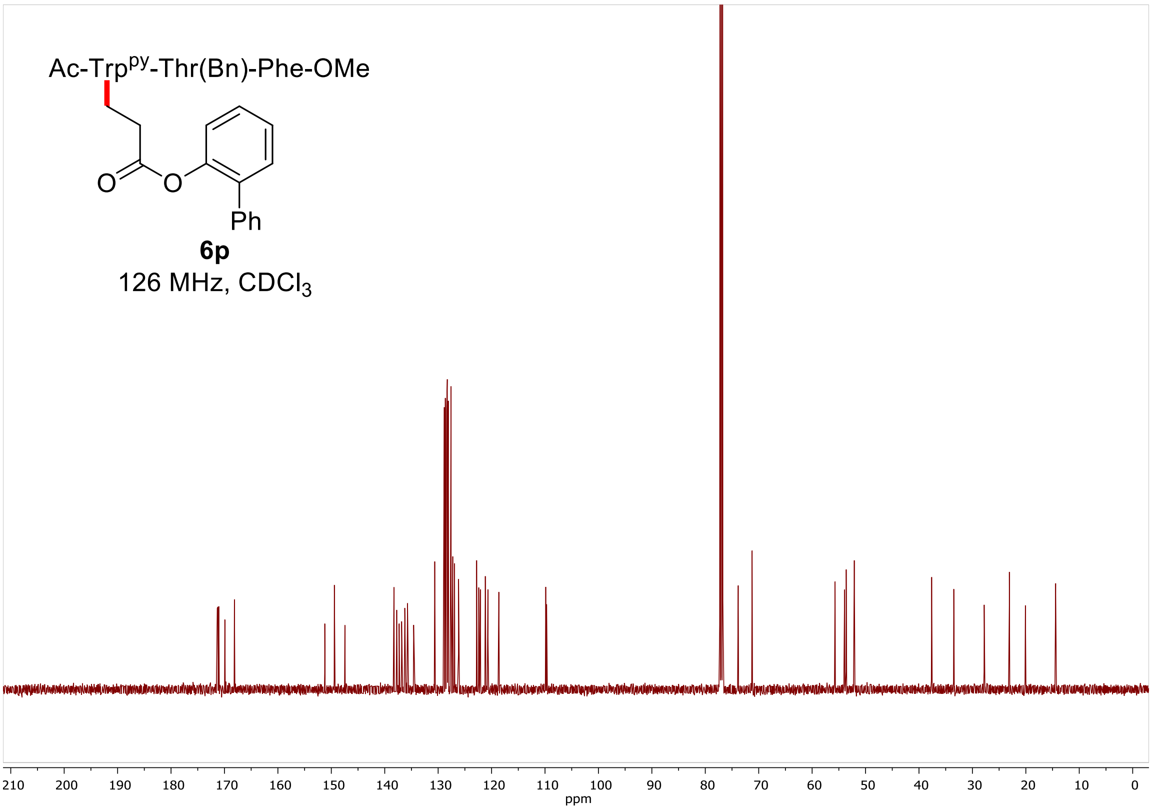

Supplementary Figure 120. ^{13}C-NMR spectrum of **6p**.

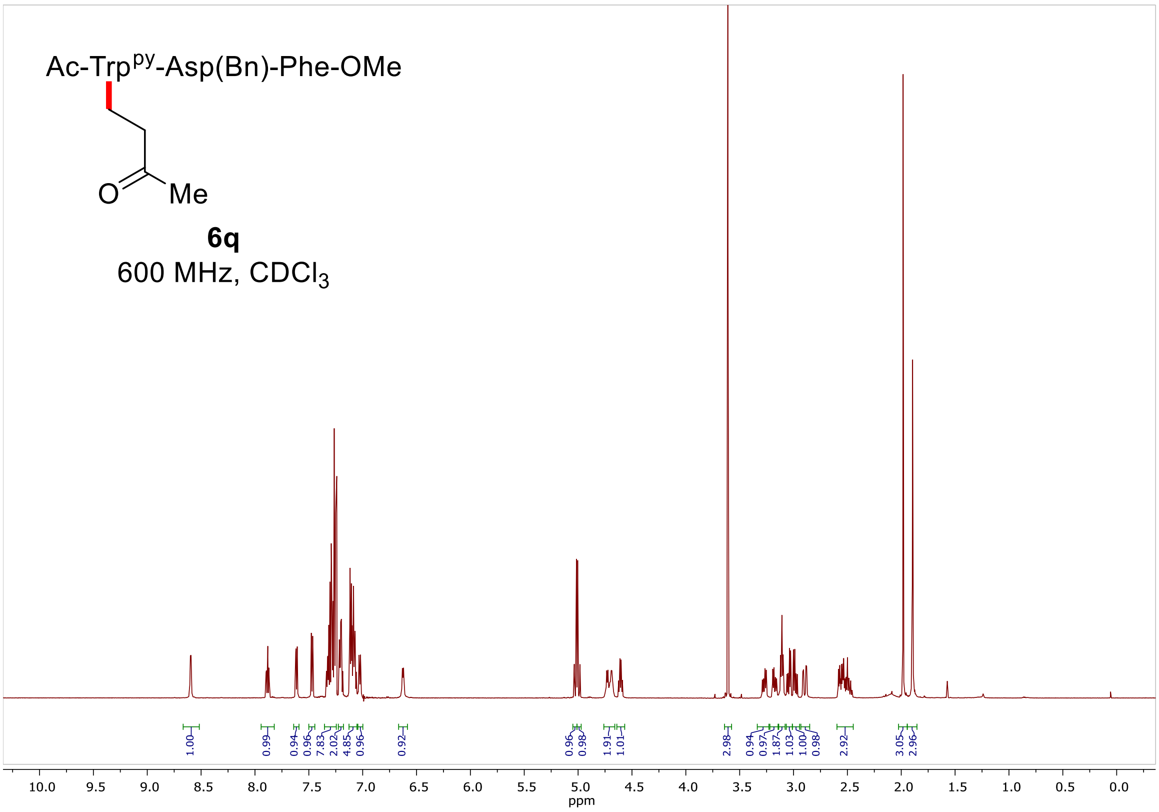

Supplementary Figure 121. ^{1}H-NMR spectrum of **6q**.

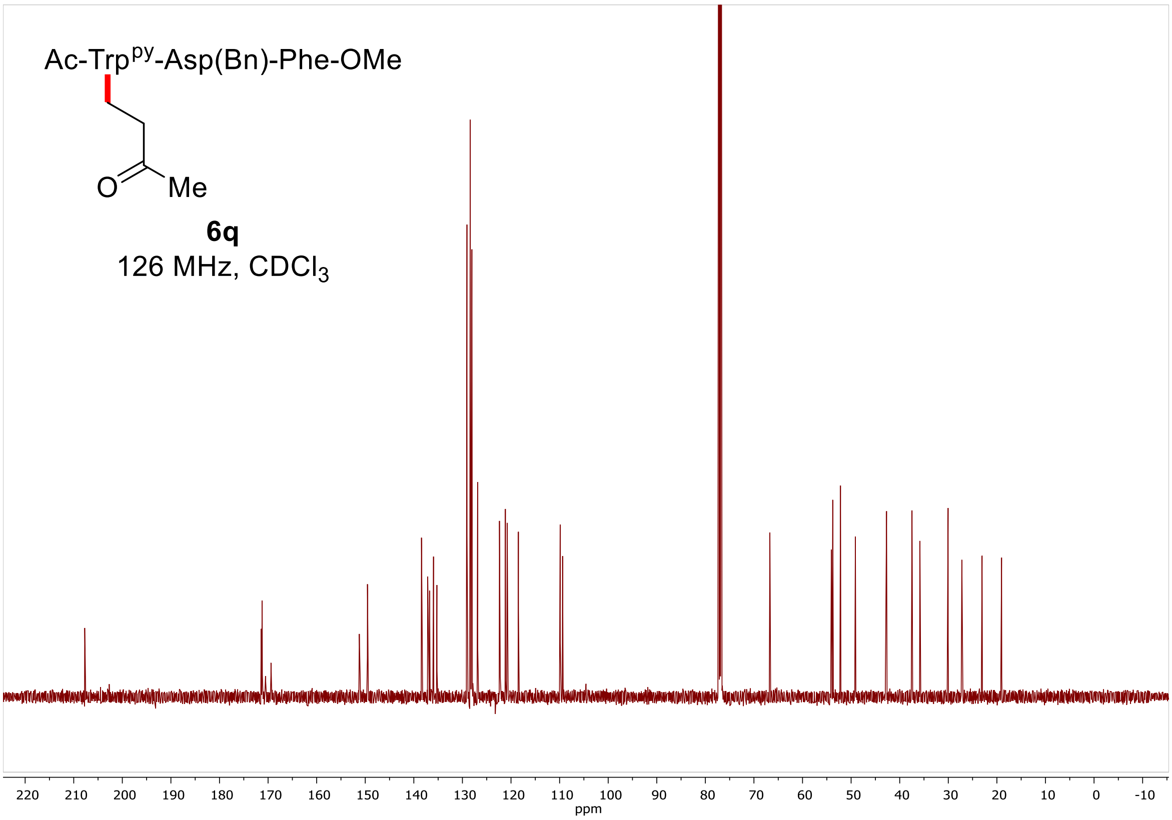

Supplementary Figure 122. ^{13}C-NMR spectrum of **6q**.

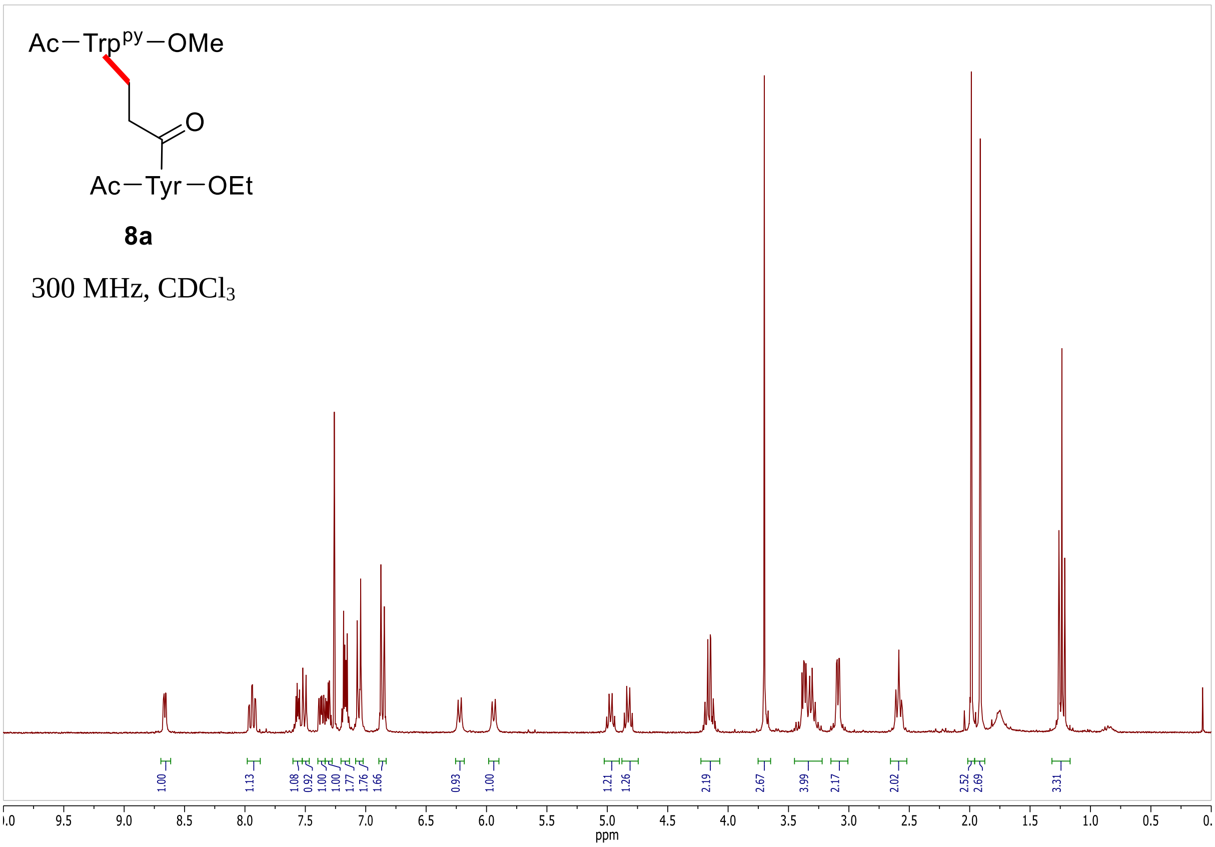

Supplementary Figure 123. ^{1}H-NMR spectrum of **8a**.

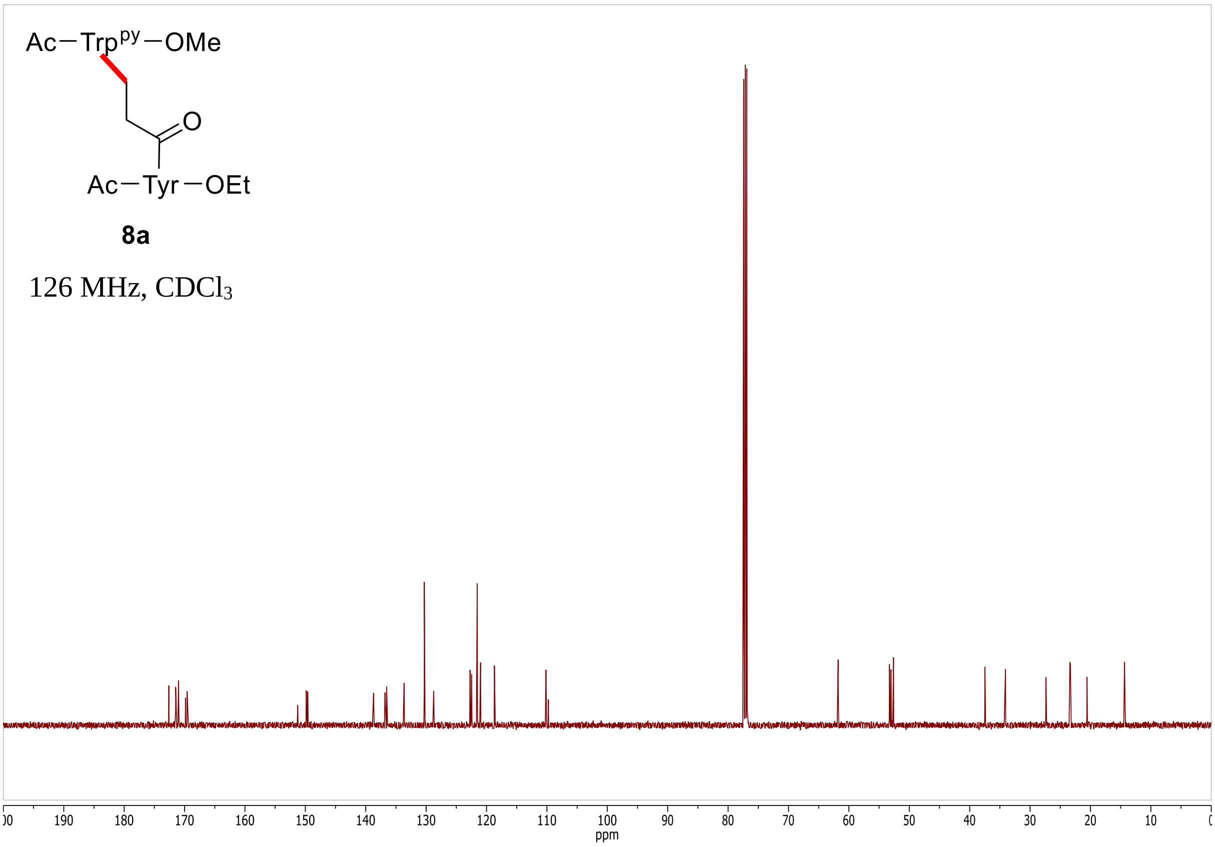

Supplementary Figure 124. ^{13}C-NMR spectrum of **8a**.

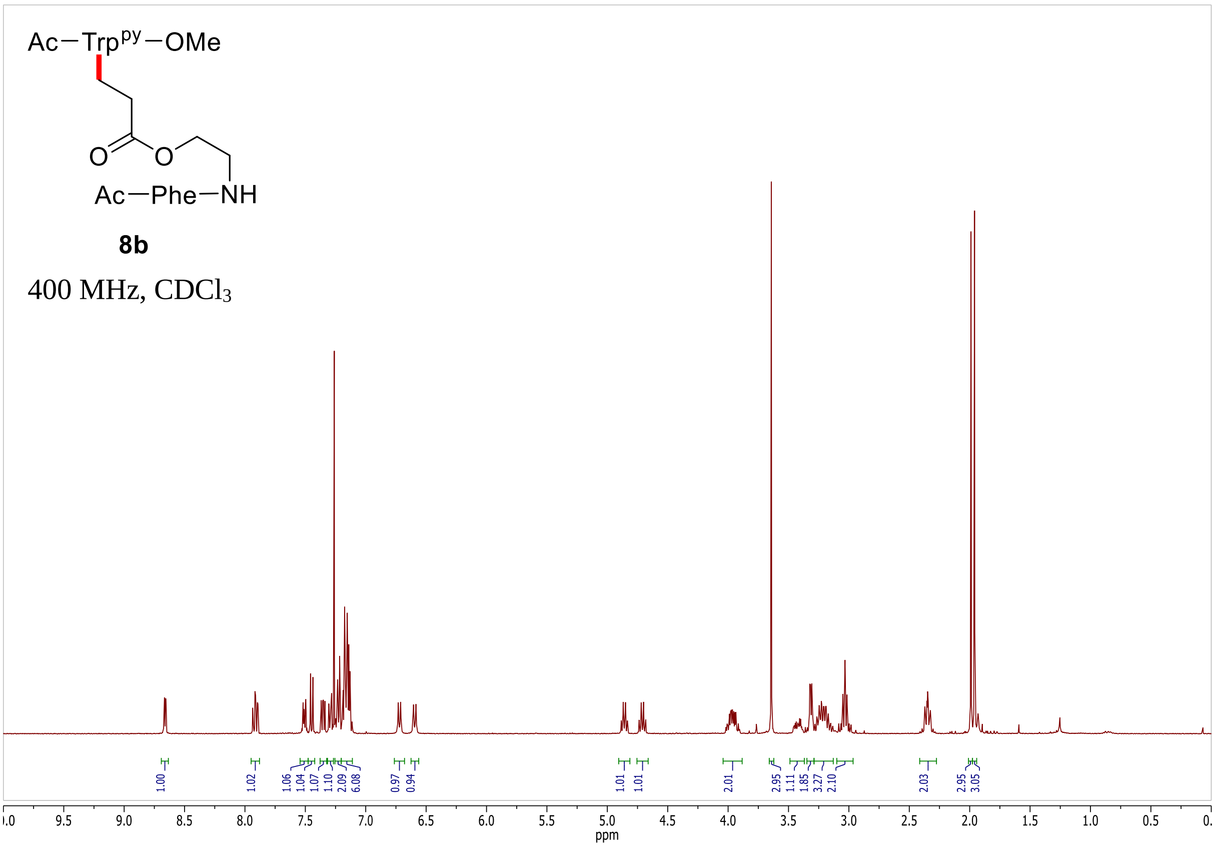

Supplementary Figure 125. ^{1}H-NMR spectrum of **8b**.

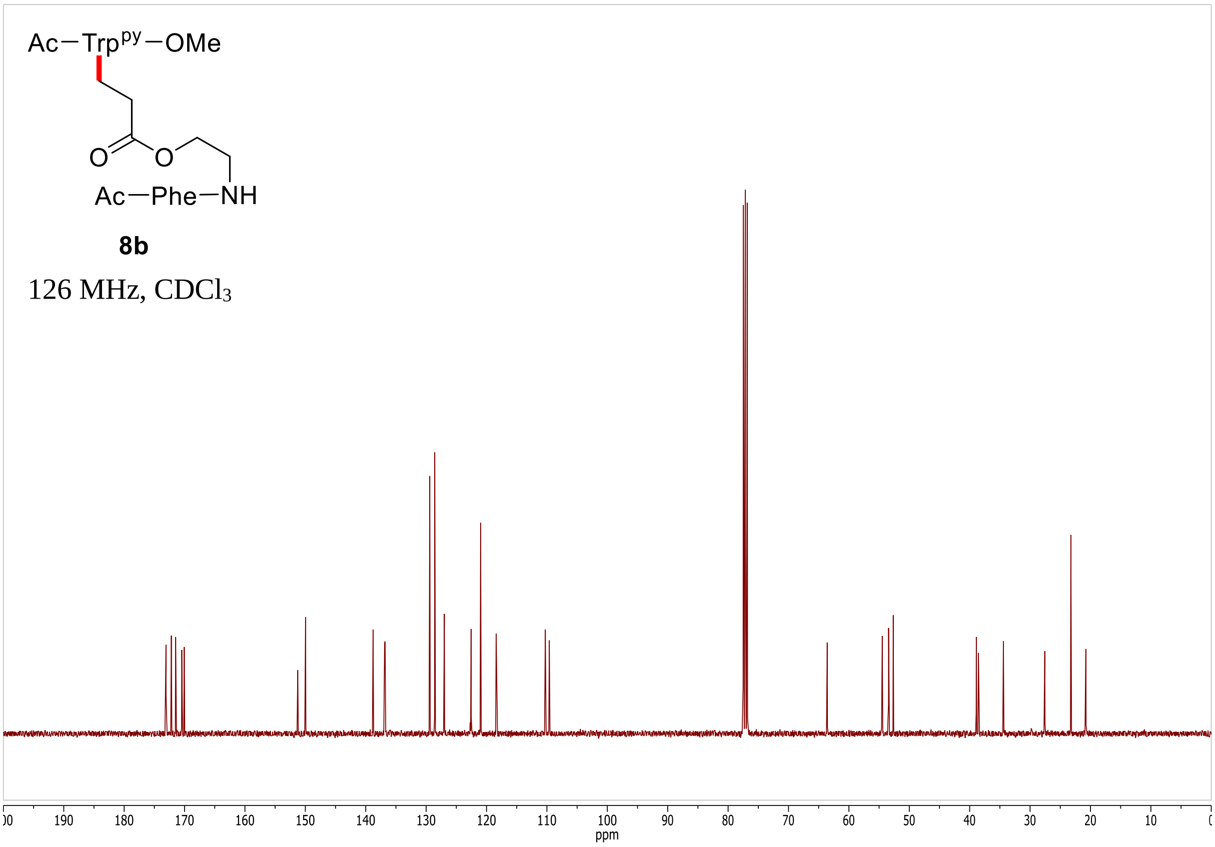

Supplementary Figure 126. ^{13}C-NMR spectrum of **8b**

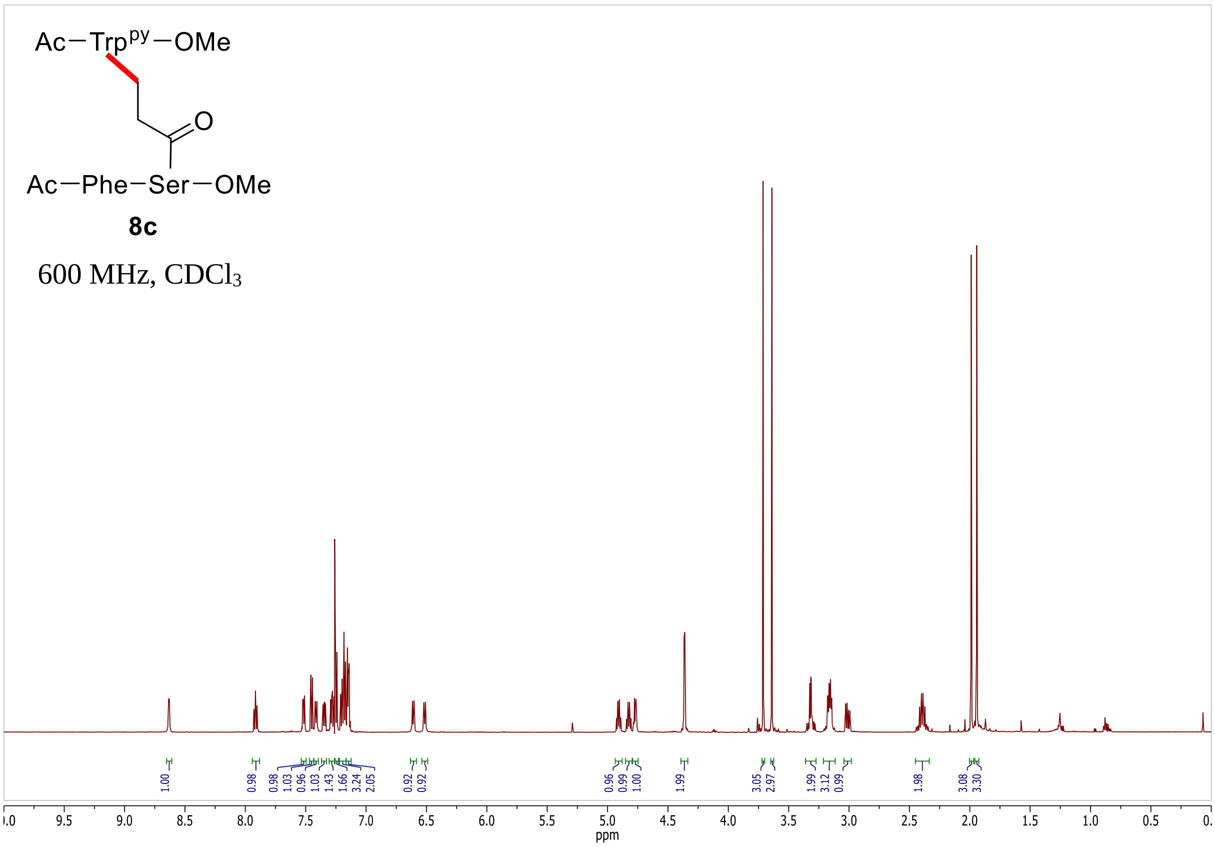

Supplementary Figure 127. ^{1}H-NMR spectrum of **8c**.

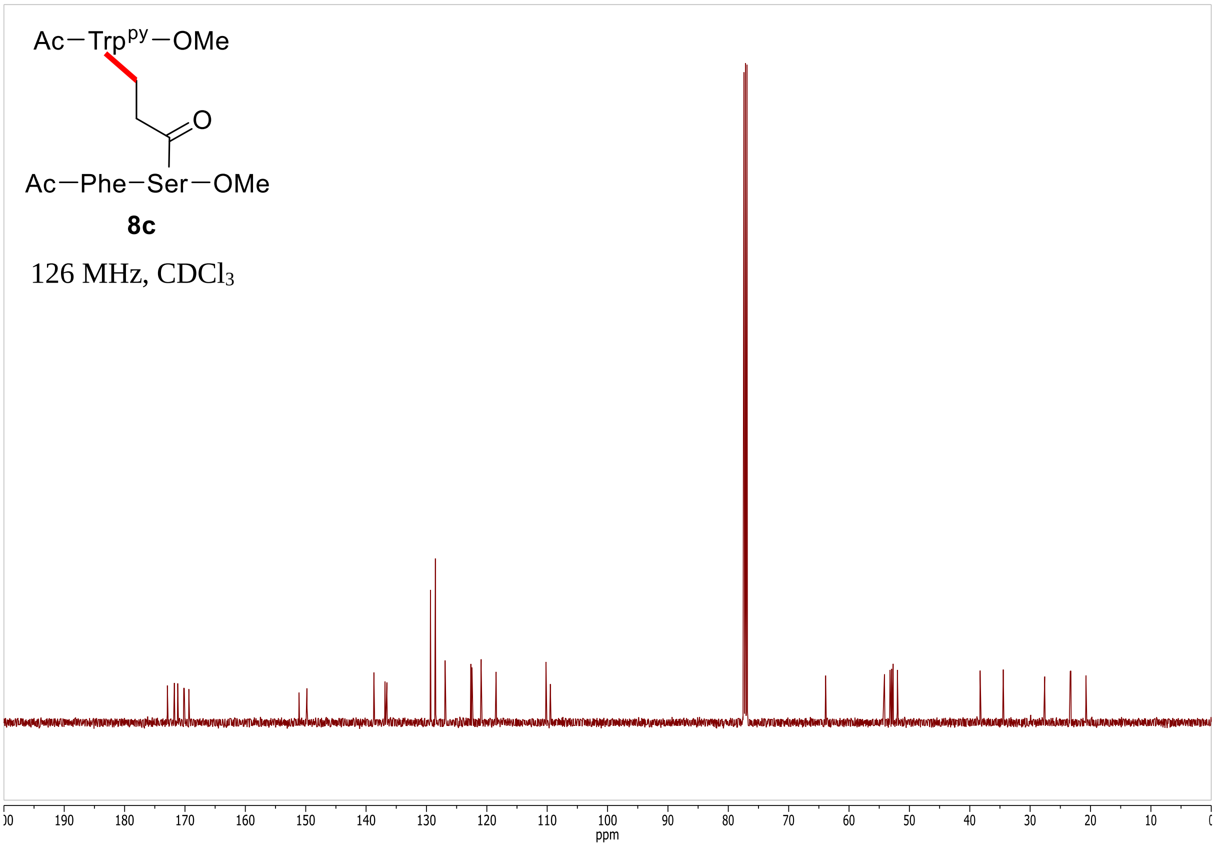

Supplementary Figure 128. ^{13}C-NMR spectrum of **8c**.

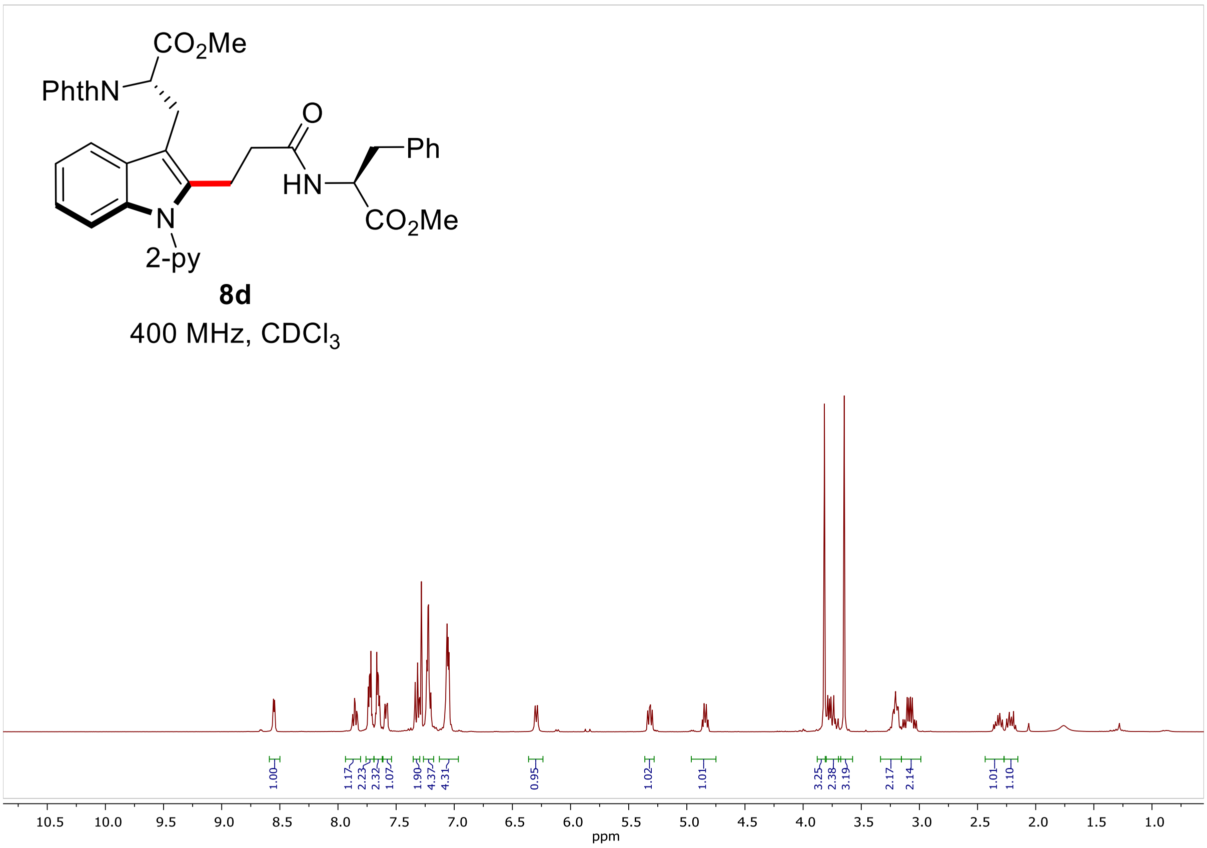

Supplementary Figure 129. ^{1}H-NMR spectrum of **8d**.

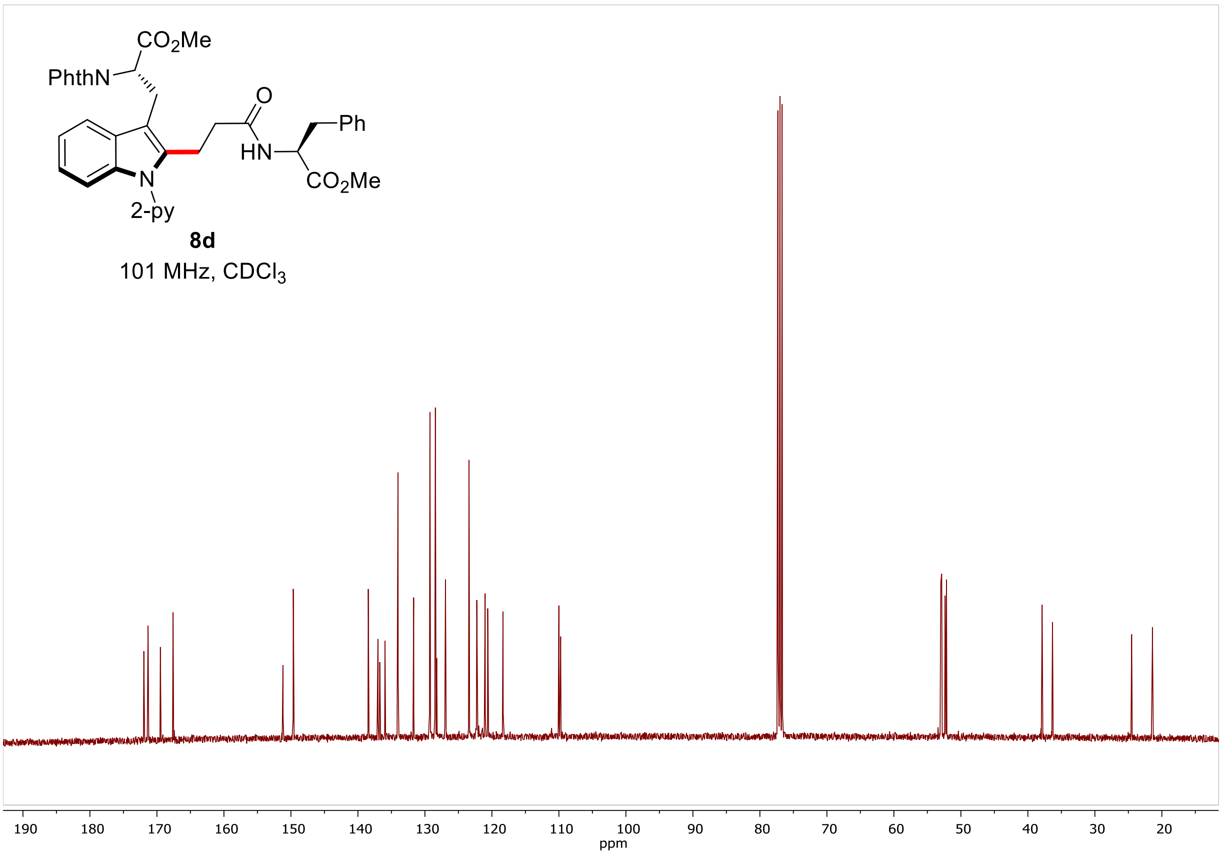

Supplementary Figure 130. ^{13}C-NMR spectrum of **8d**.

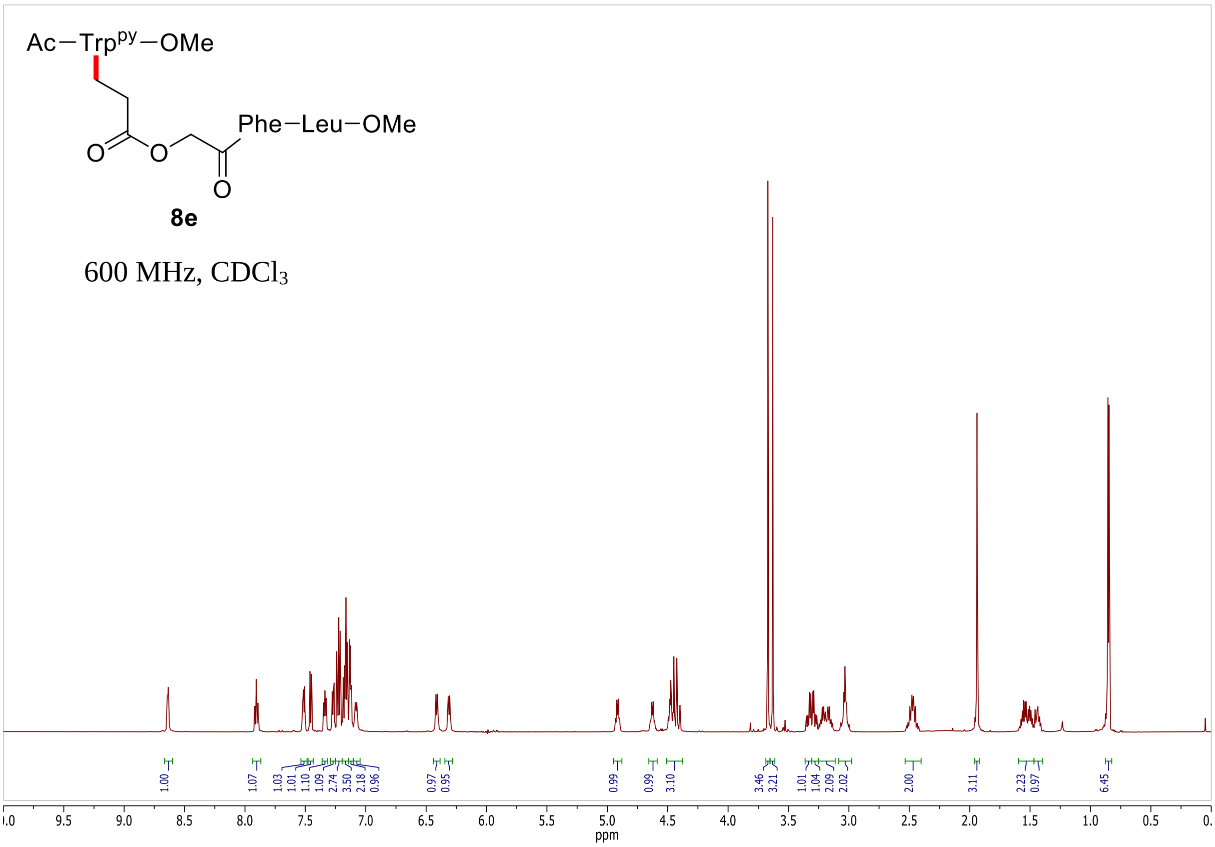

Supplementary Figure 131. ^{1}H-NMR spectrum of **8e**.

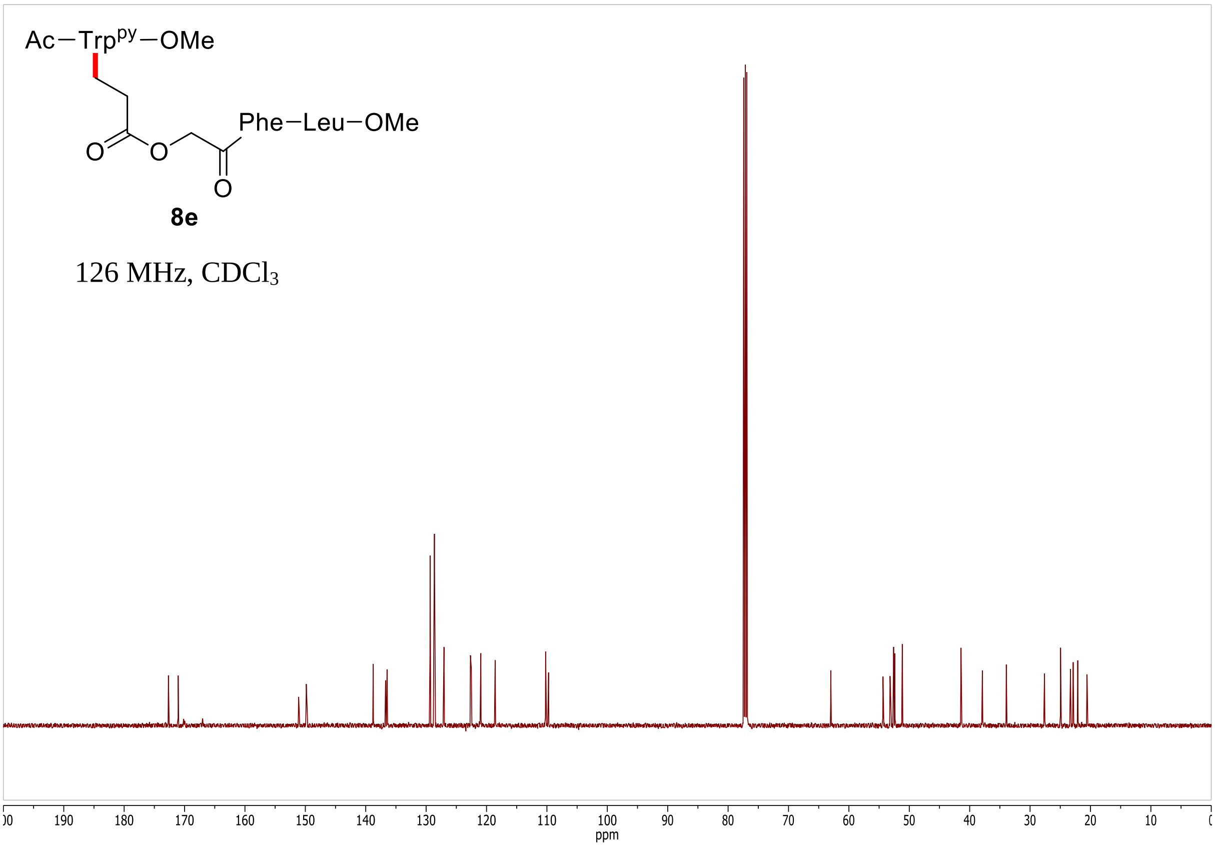

Supplementary Figure 132. ^{13}C-NMR spectrum of **8e**.

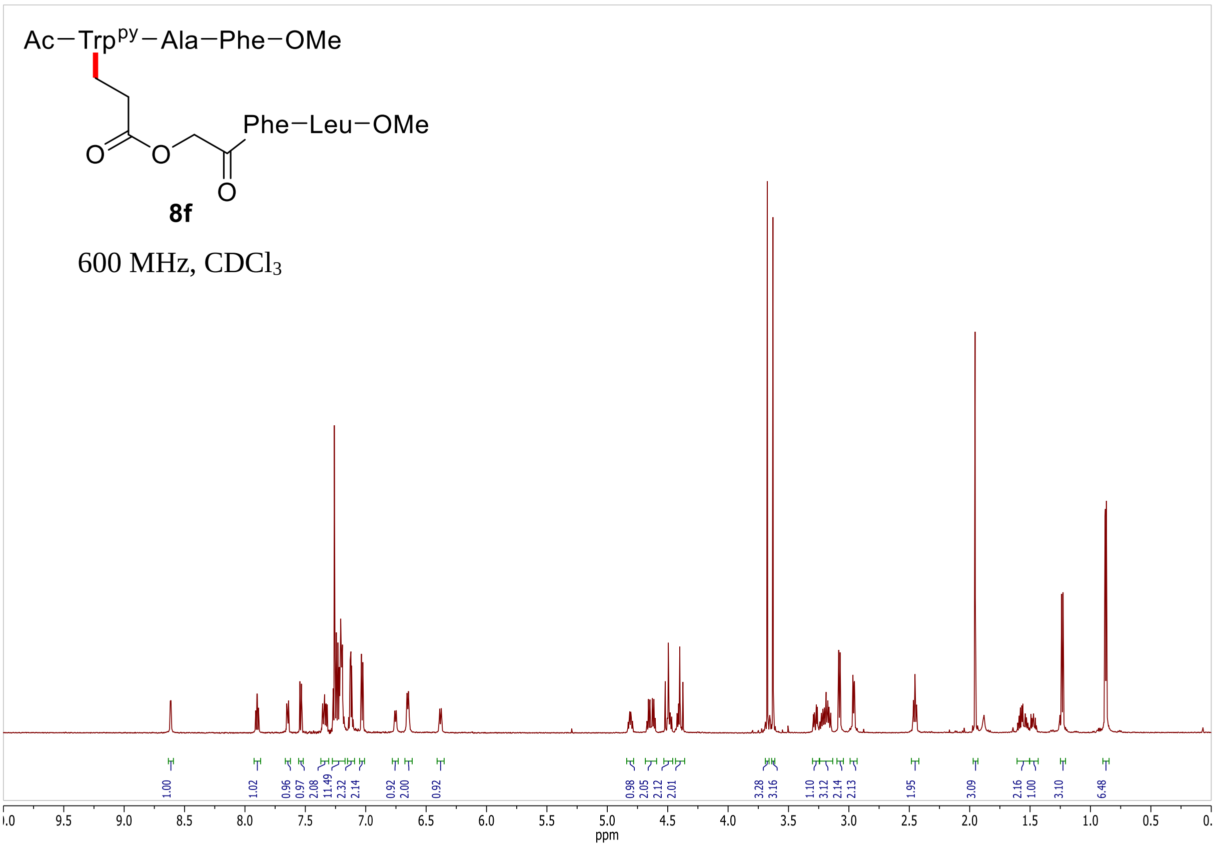

Supplementary Figure 133. ^{1}H-NMR spectrum of **8f**.

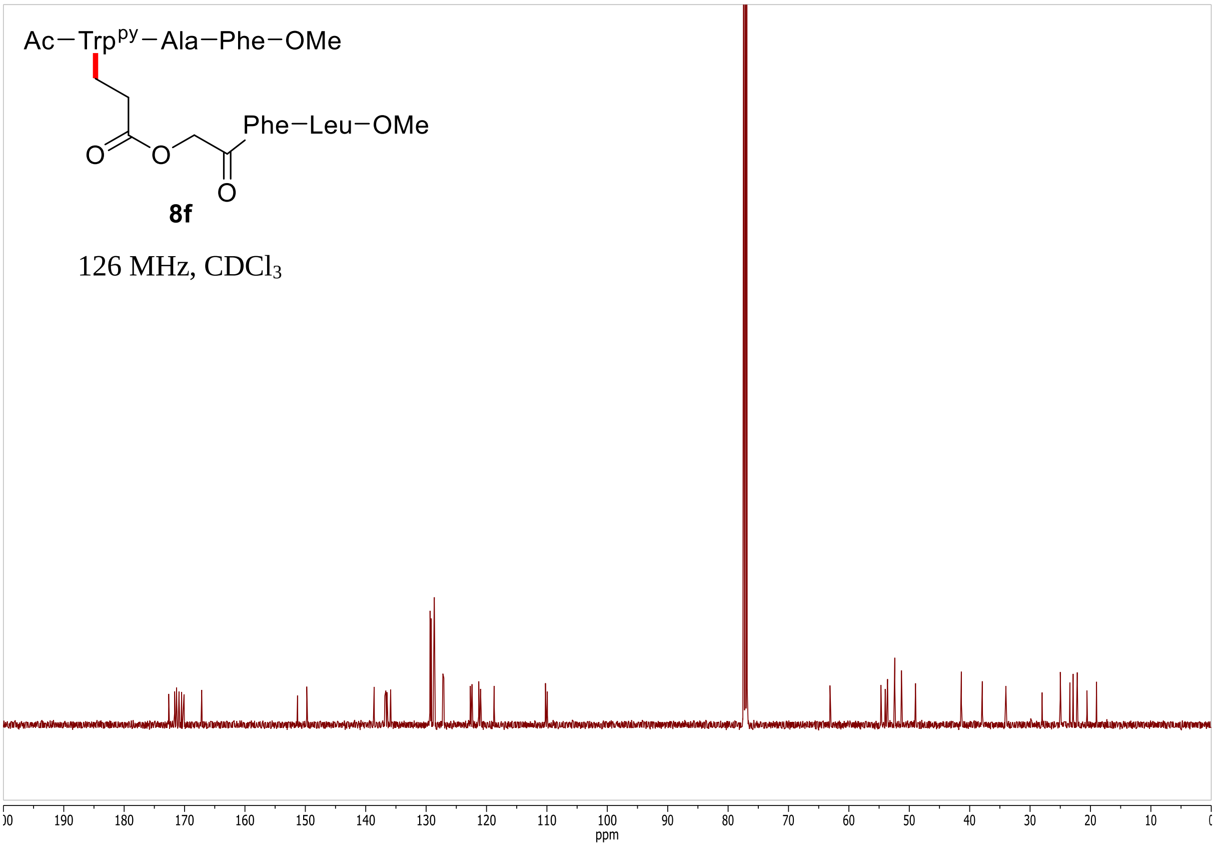

Supplementary Figure 134. ^{13}C-NMR spectrum of **8f**.

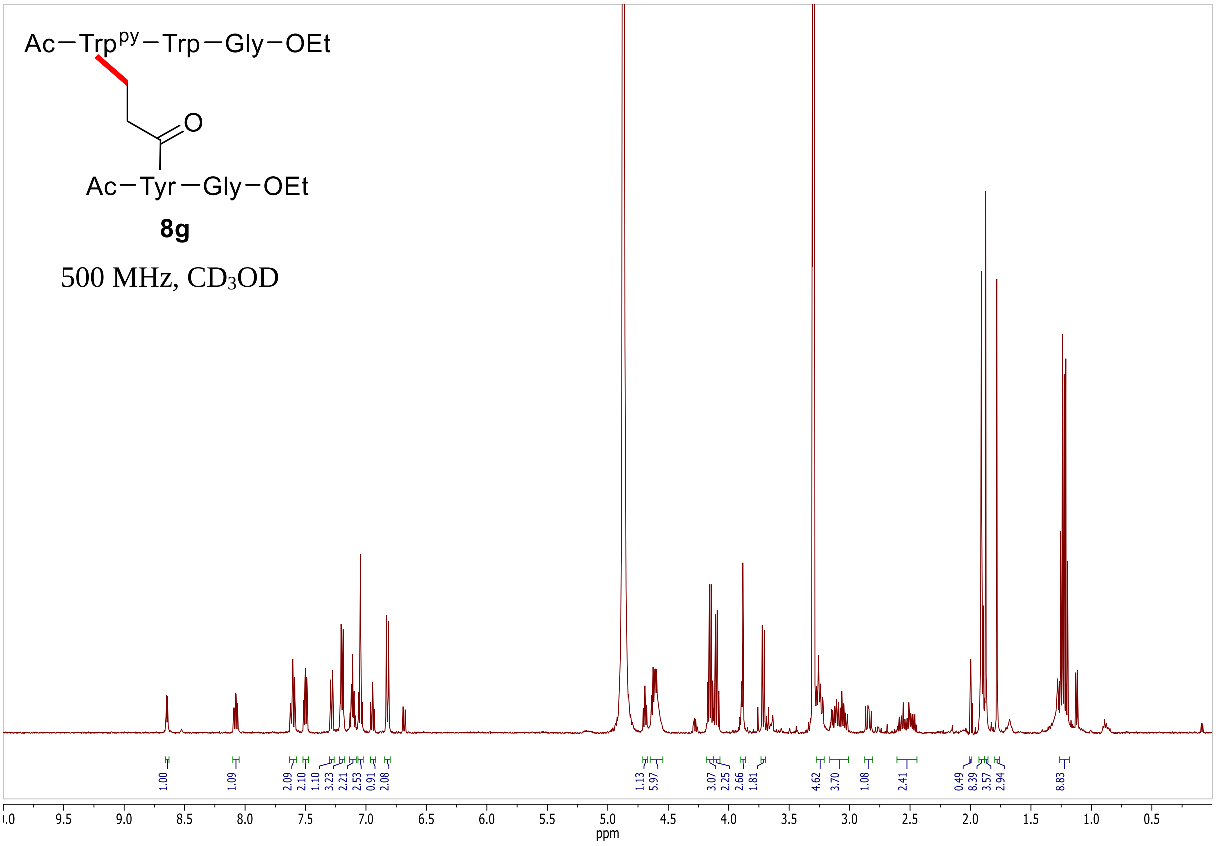

Supplementary Figure 135. ^{1}H-NMR spectrum of **8g**.

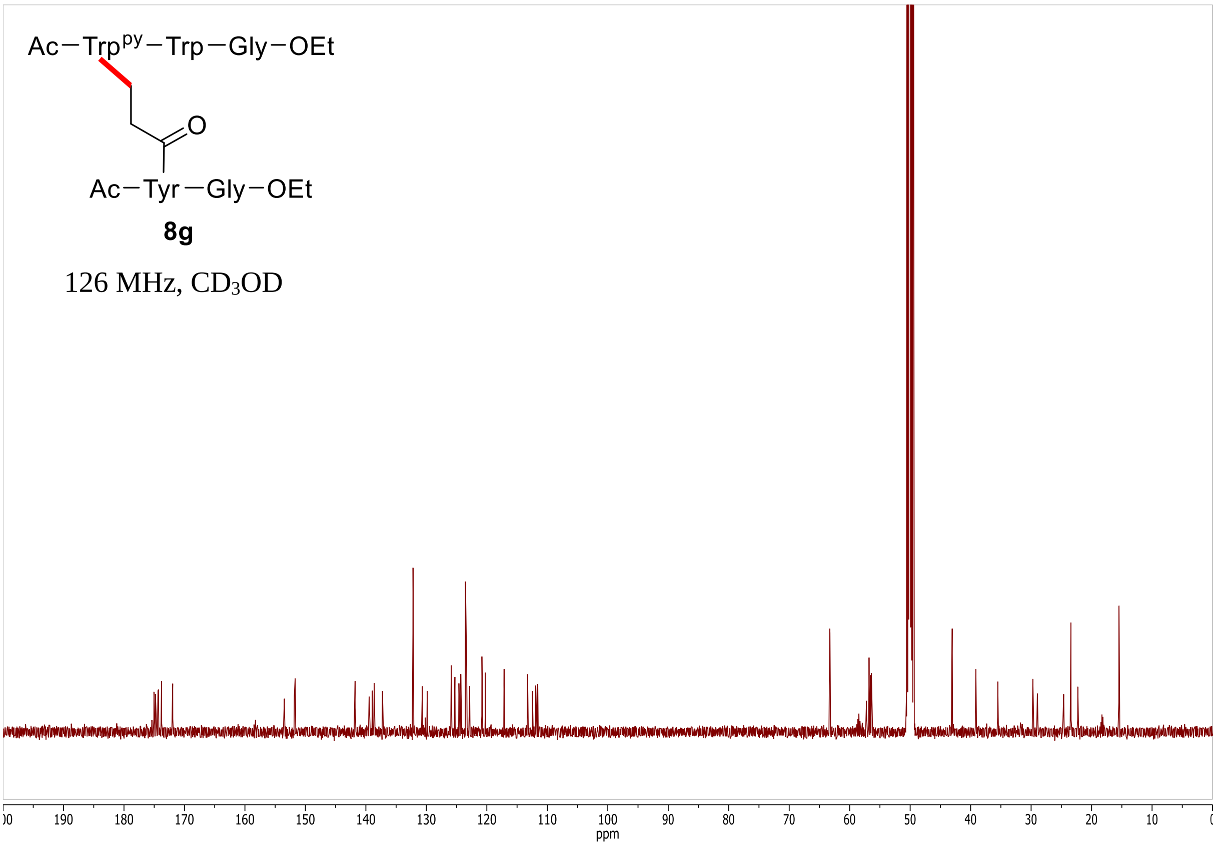

Supplementary Figure 136. ^{13}C-NMR spectrum of **8g**

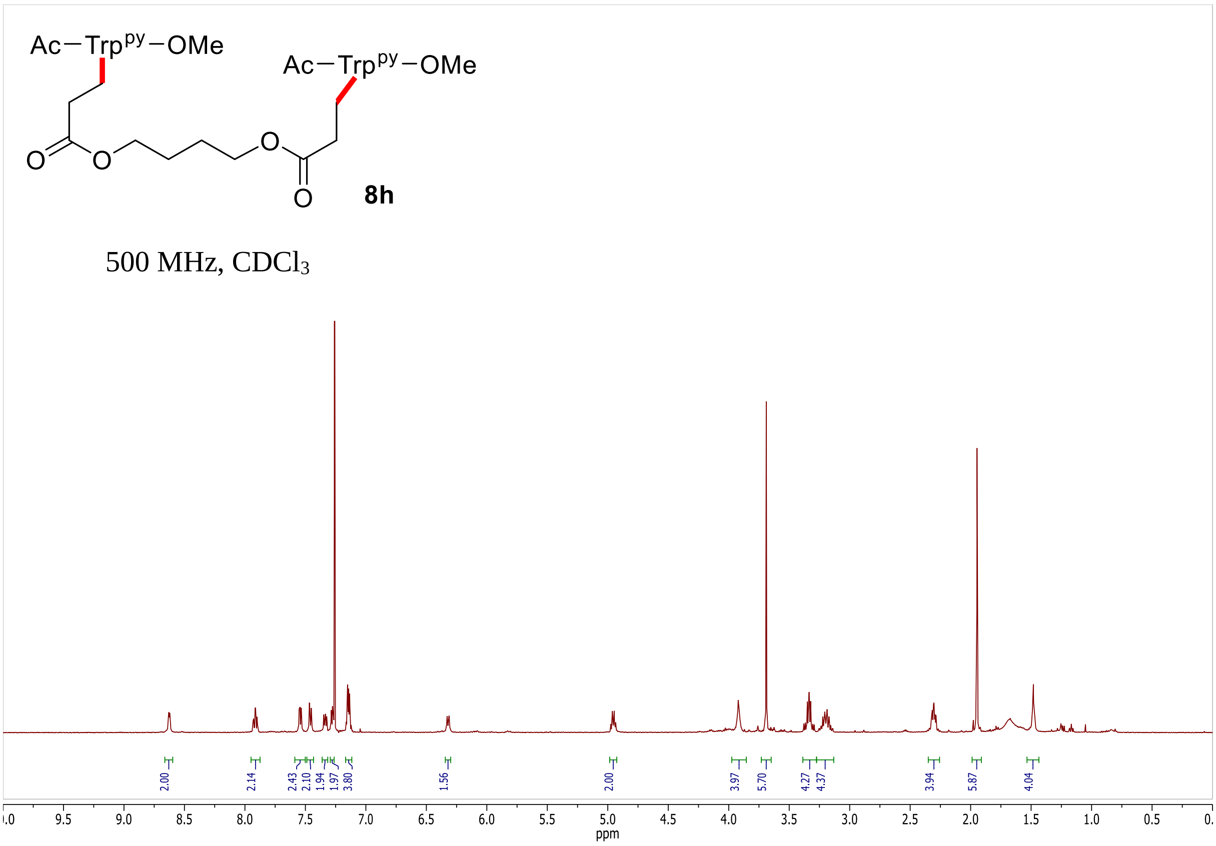

Supplementary Figure 137. ^{1}H-NMR spectrum of **8h**.

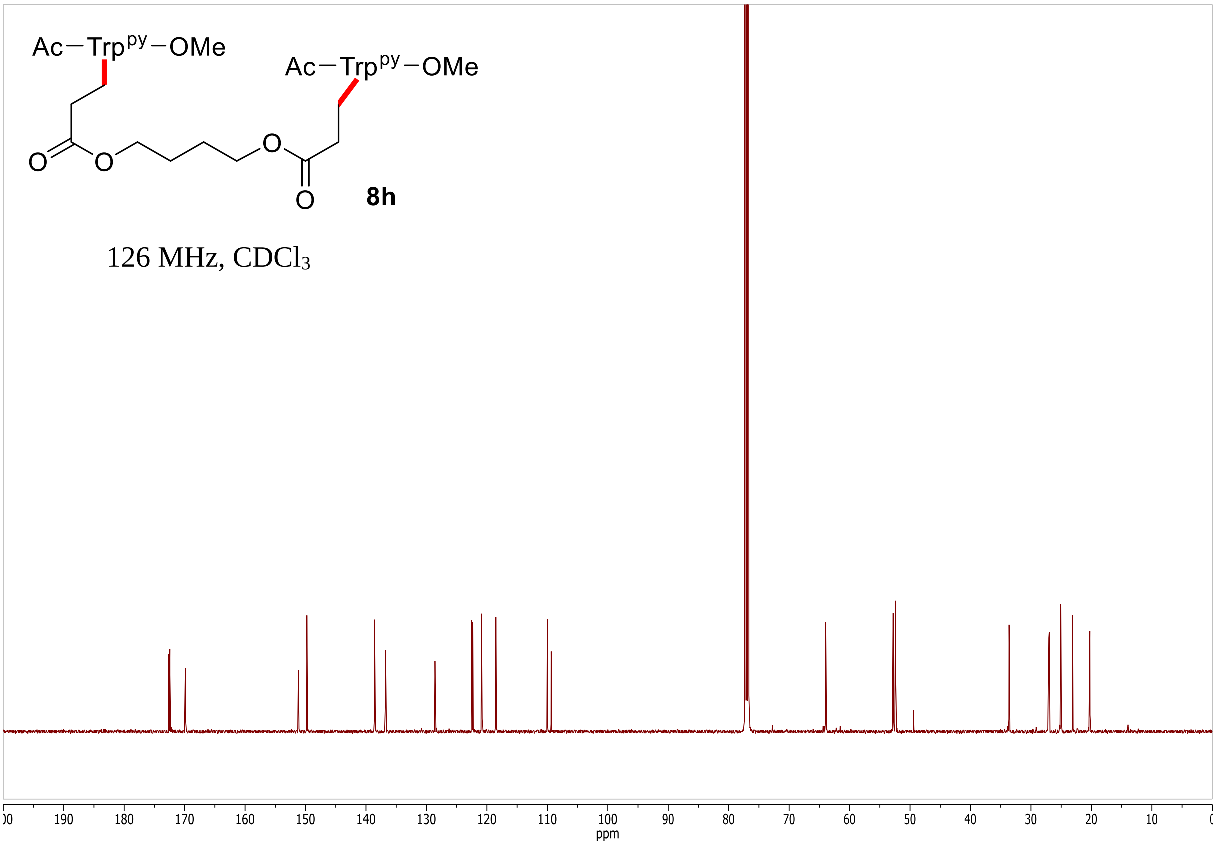

Supplementary Figure 138. ^{13}C-NMR spectrum of **8h**.

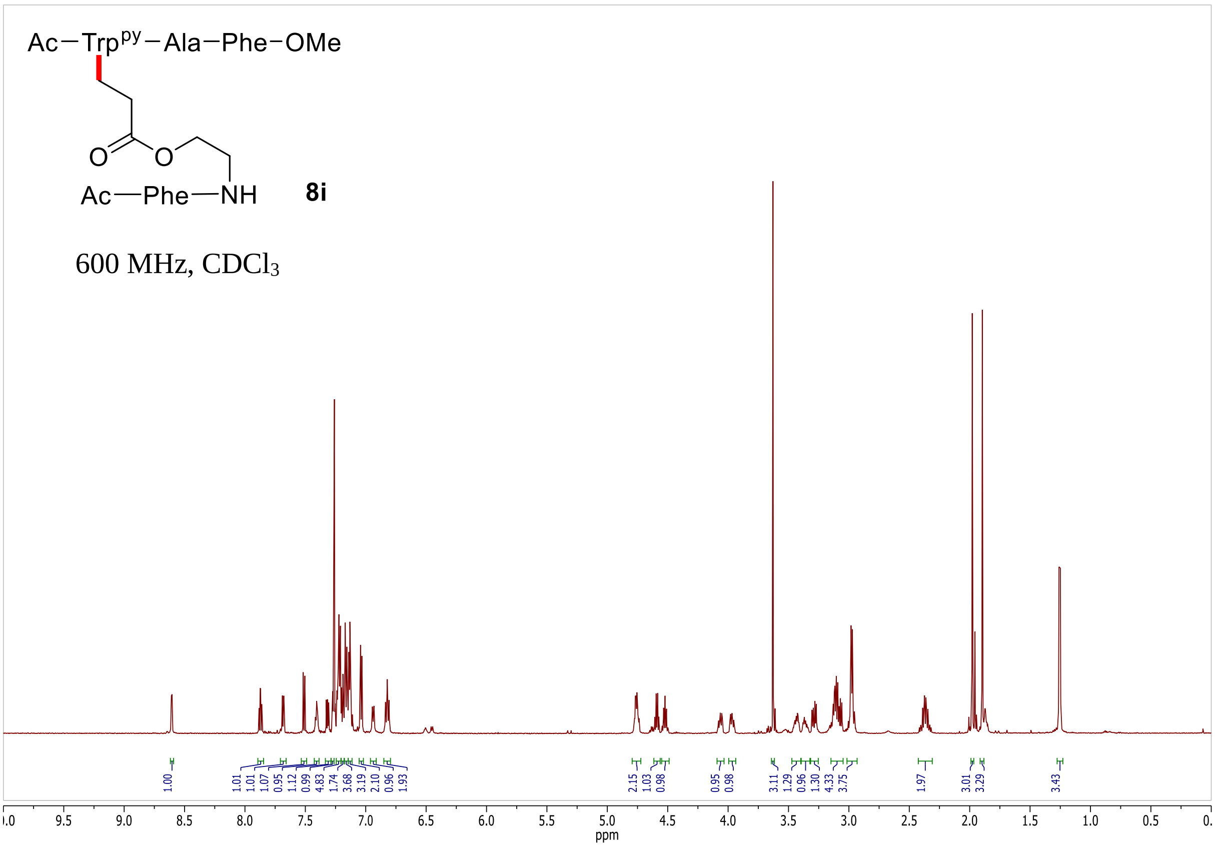

Supplementary Figure 139. ^{1}H-NMR spectrum of **8i**.

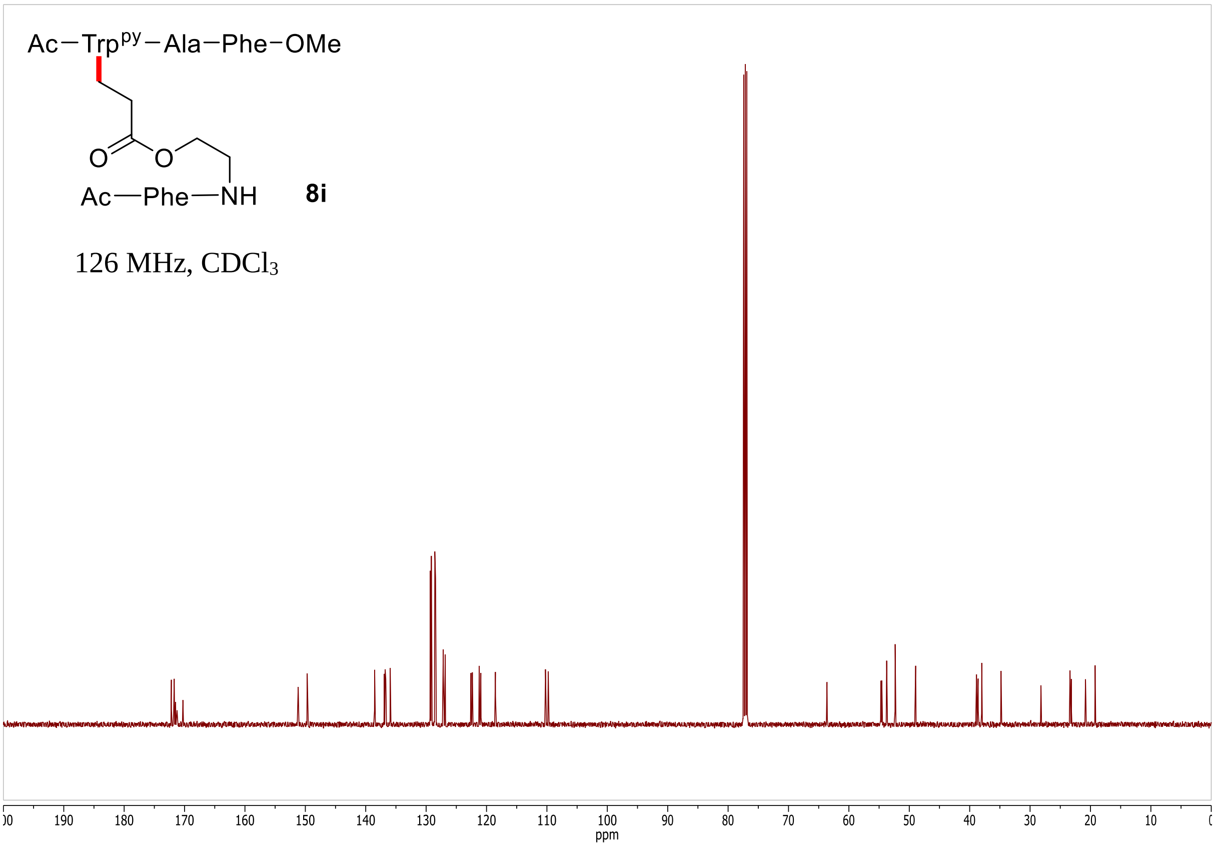

Supplementary Figure 140. ^{13}C-NMR spectrum of **8i**.

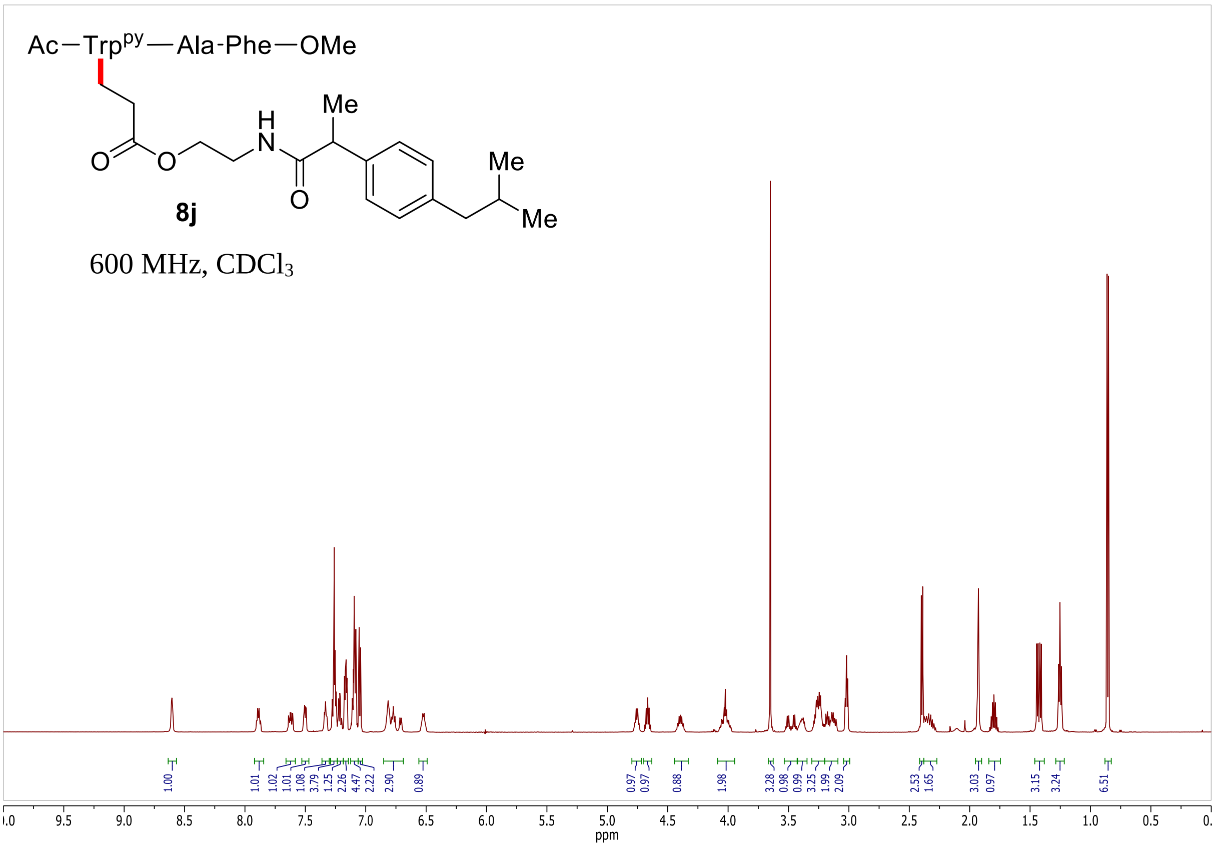

Supplementary Figure 141. ^{1}H-NMR spectrum of **8j**.

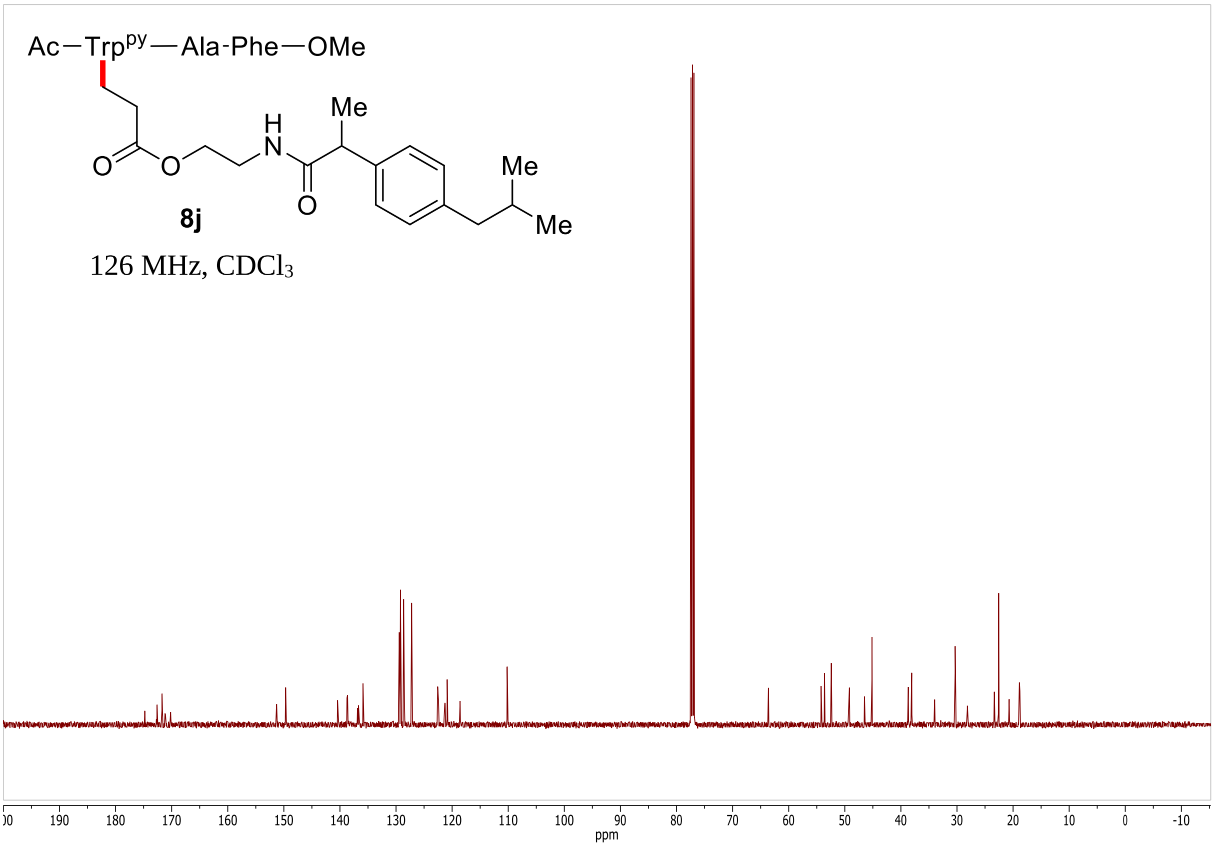

Supplementary Figure 142. ^{13}C-NMR spectrum of **8j**.

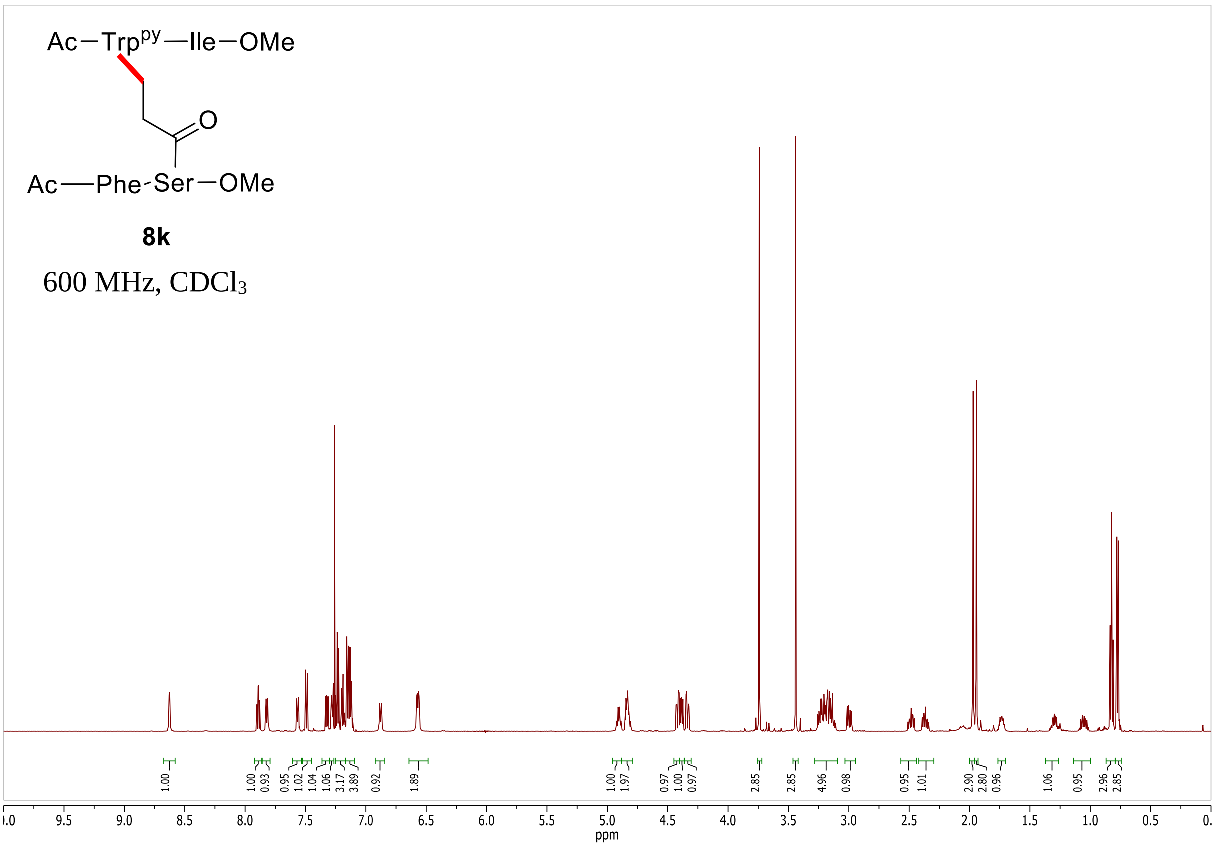

Supplementary Figure 143. ^{1}H-NMR spectrum of **8k**.

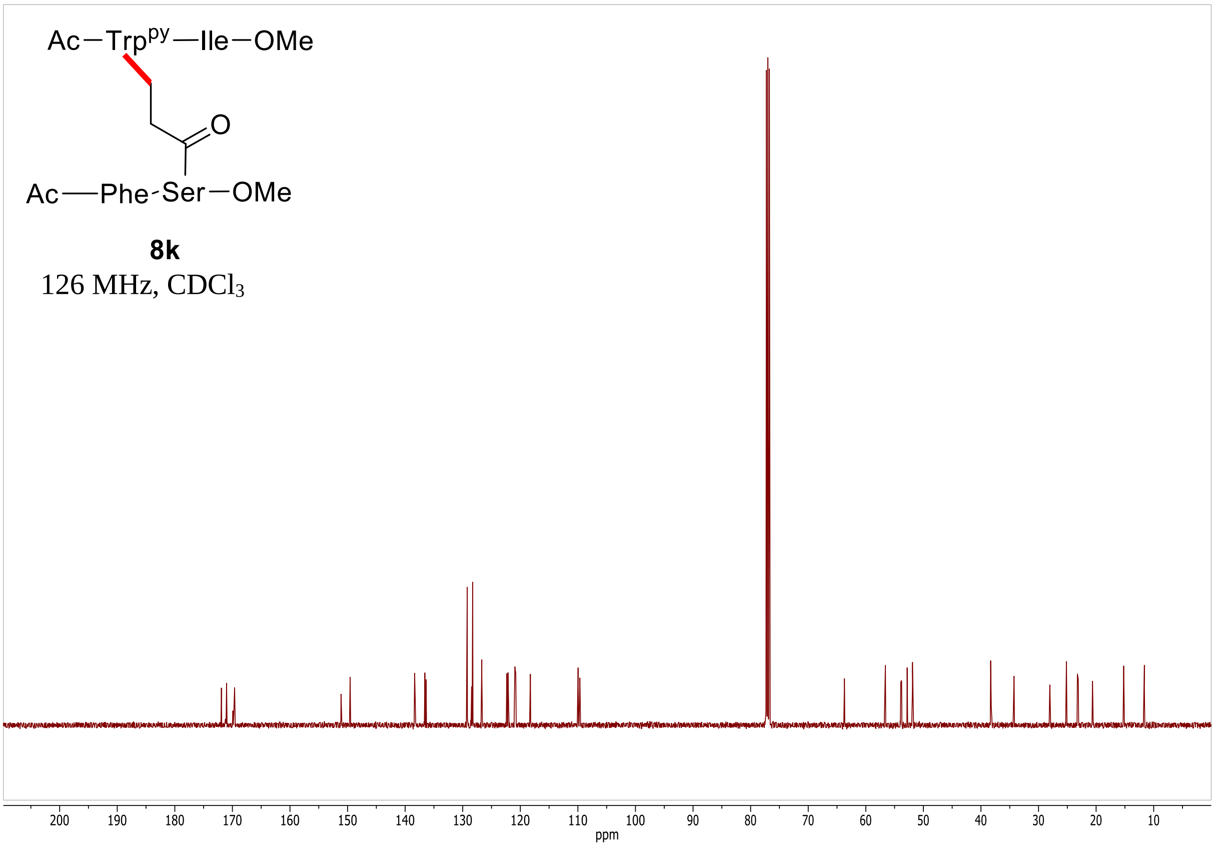

Supplementary Figure 144. ^{13}C-NMR spectrum of **8k**.

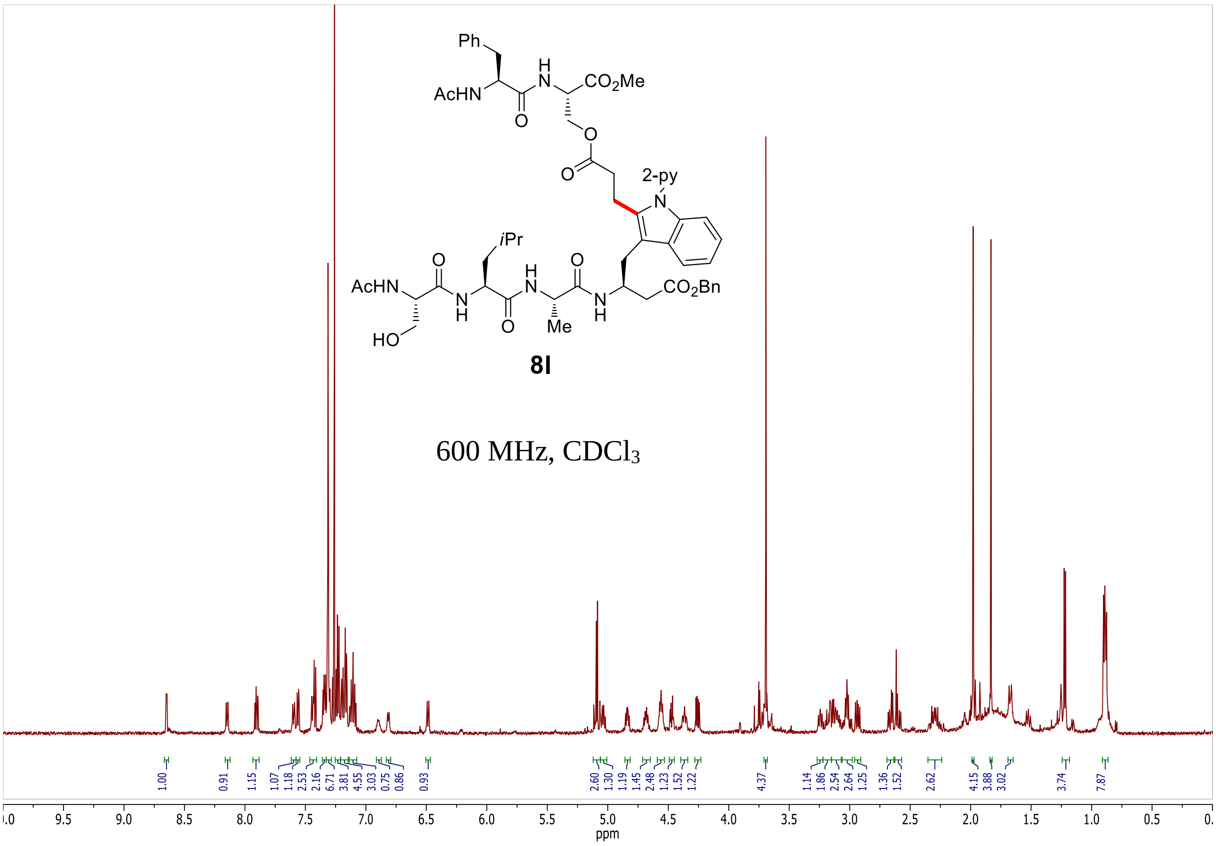

Supplementary Figure 145. ^{1}H-NMR spectrum of **8l**.

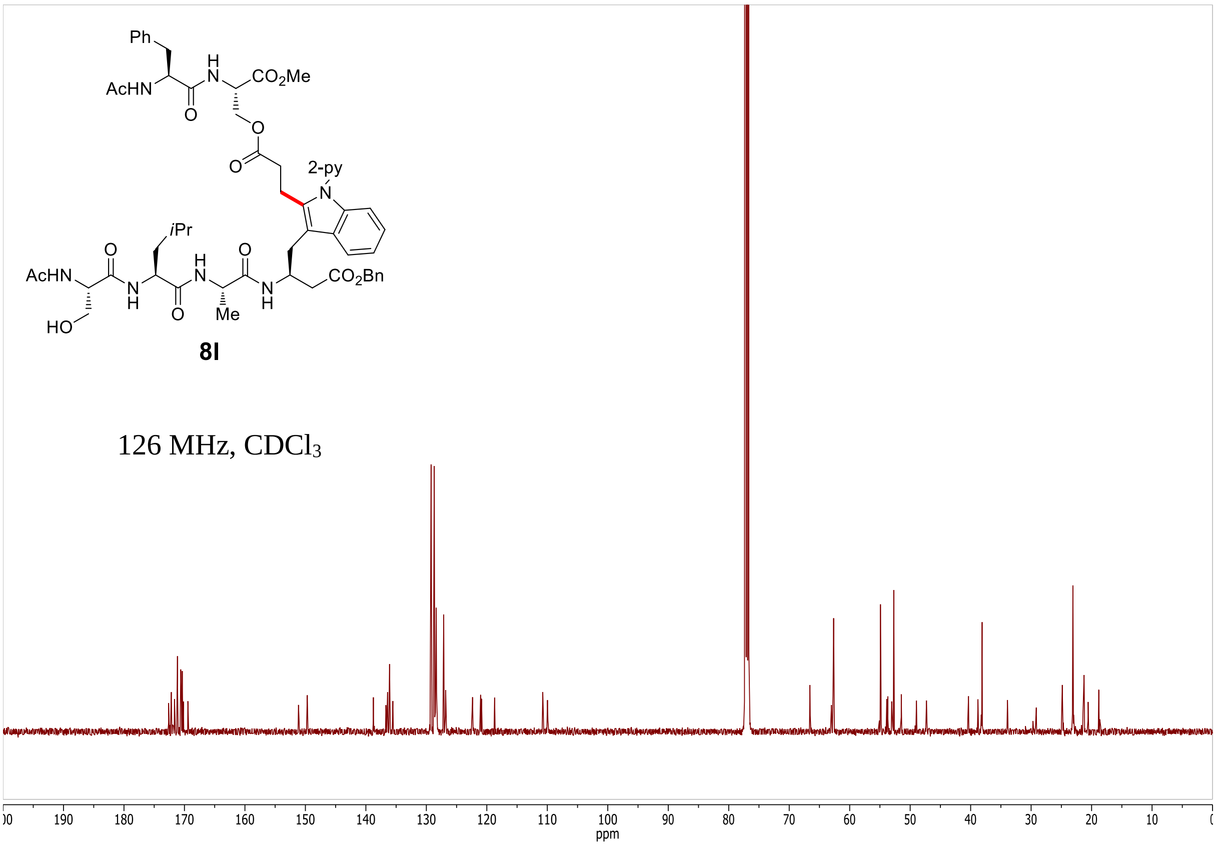

Supplementary Figure 146. ^{13}C-NMR spectrum of **8l**.

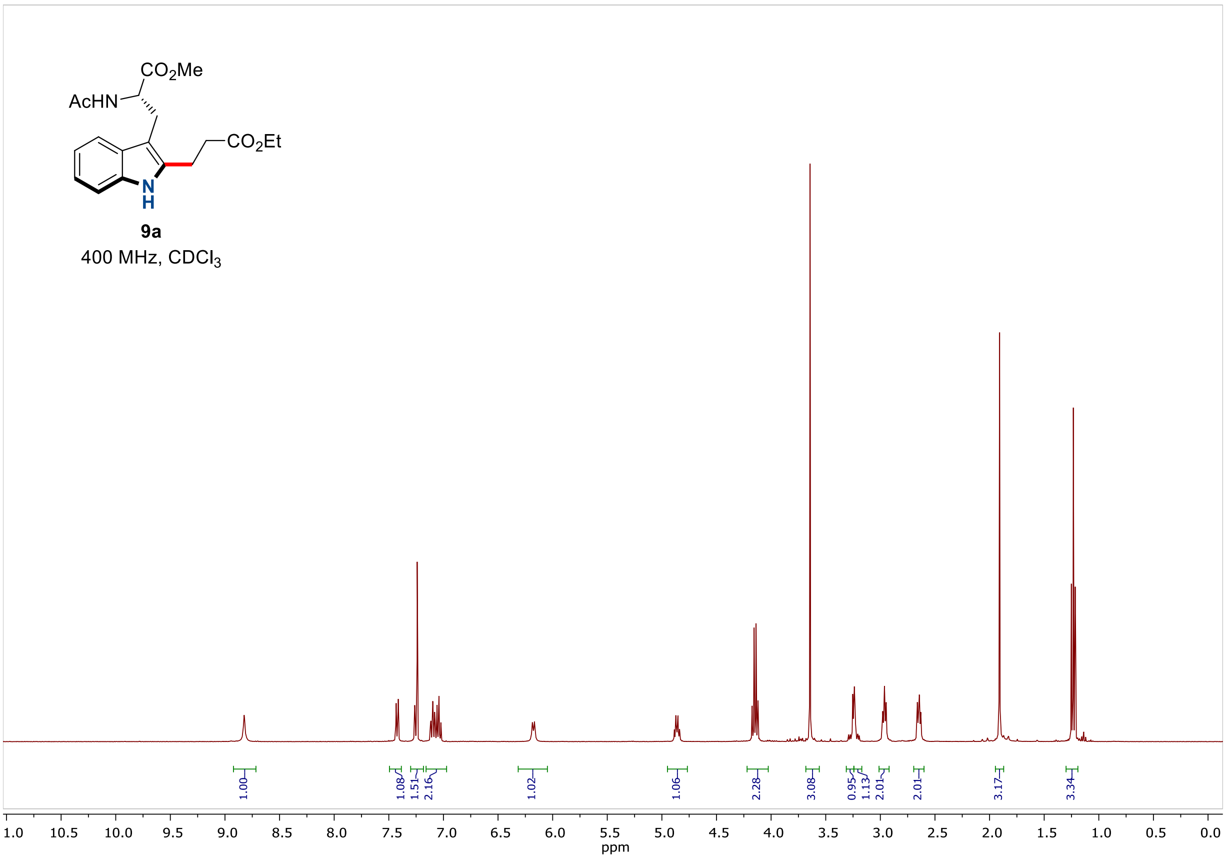

Supplementary Figure 147. ^{1}H-NMR spectrum of **9a**.

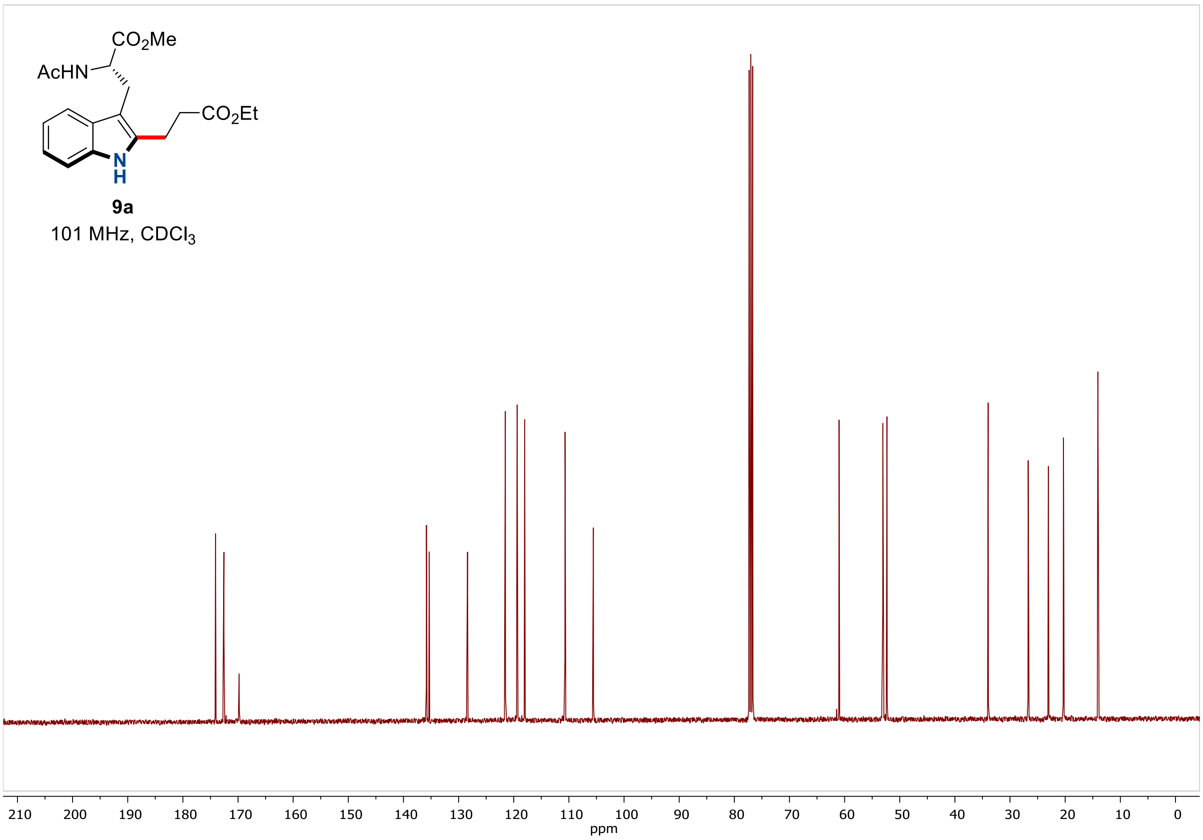

Supplementary Figure 148. ^{13}C-NMR spectrum of **9a**.

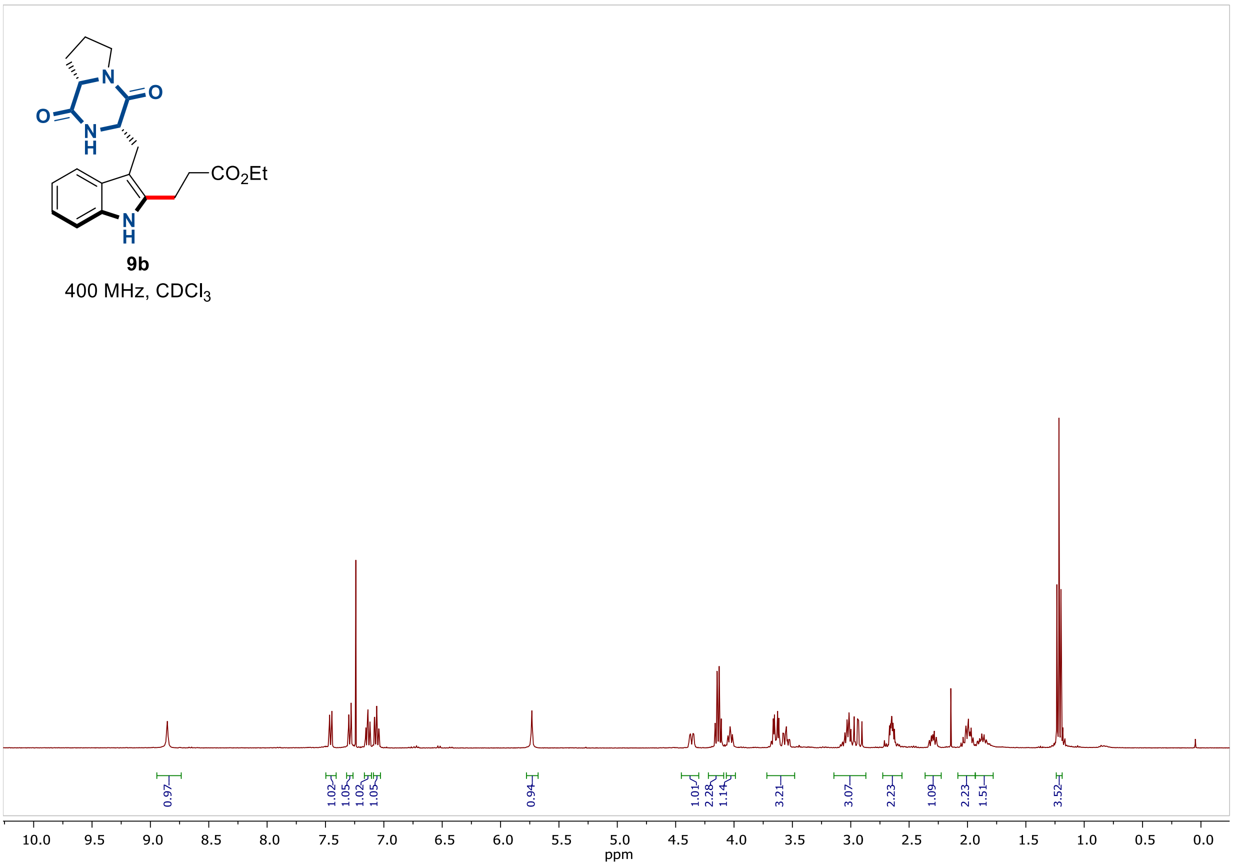

Supplementary Figure 149. ^{1}H-NMR spectrum of **9b**.

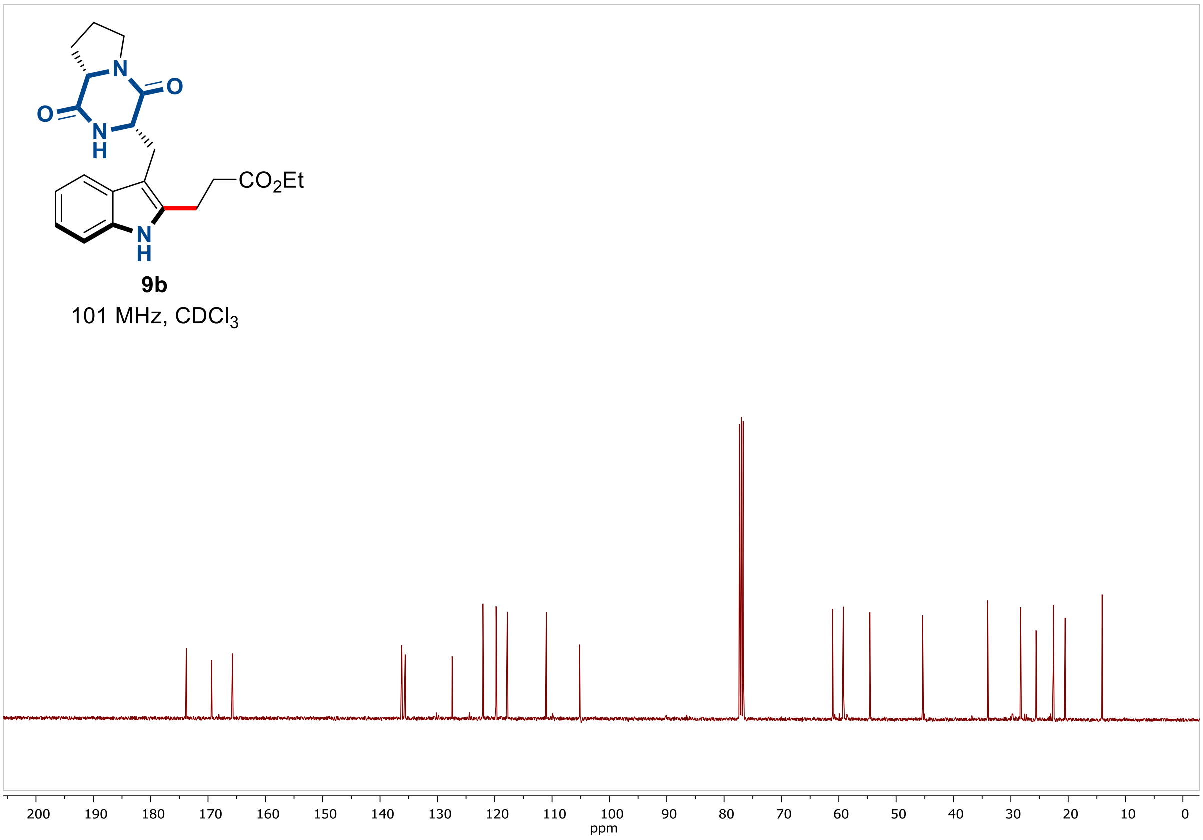

Supplementary Figure 150. ^{13}C-NMR spectrum of **9b**.

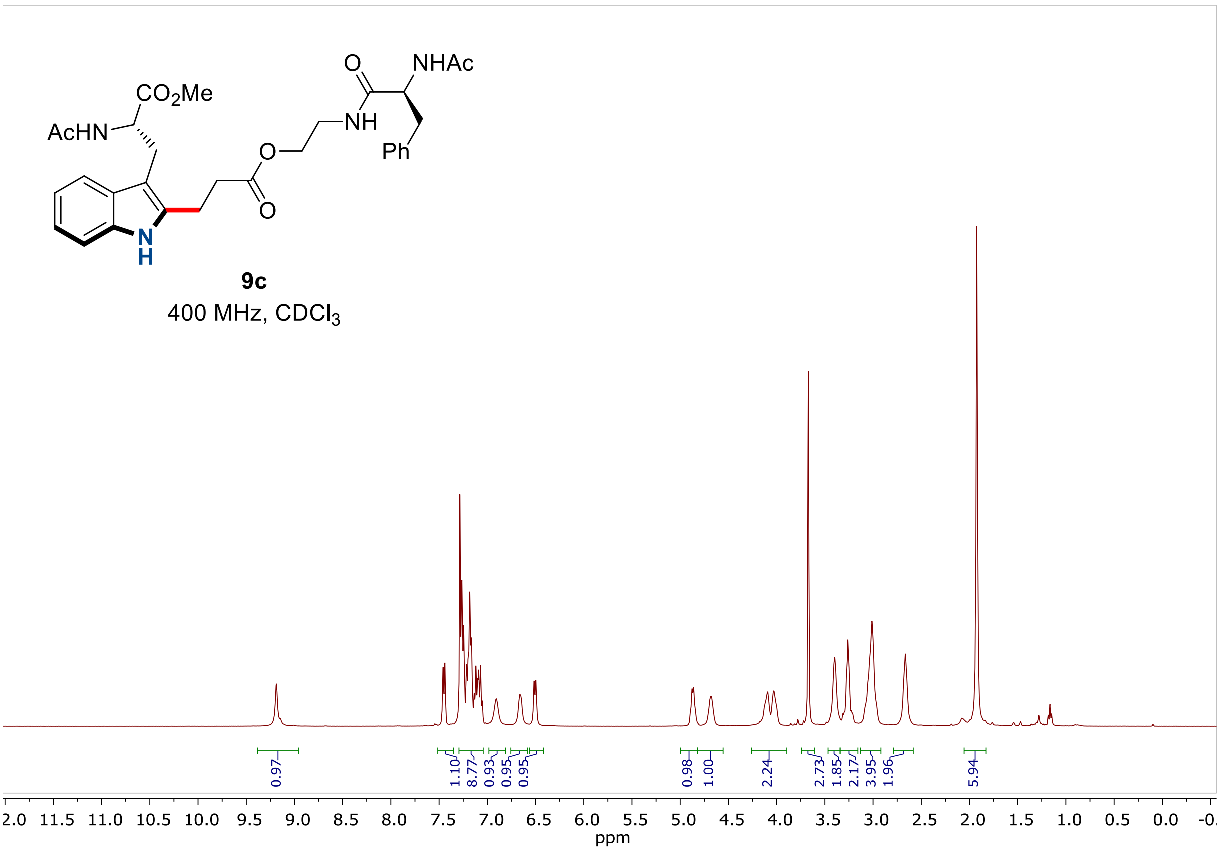

Supplementary Figure 151. ^{1}H-NMR spectrum of **9c**.

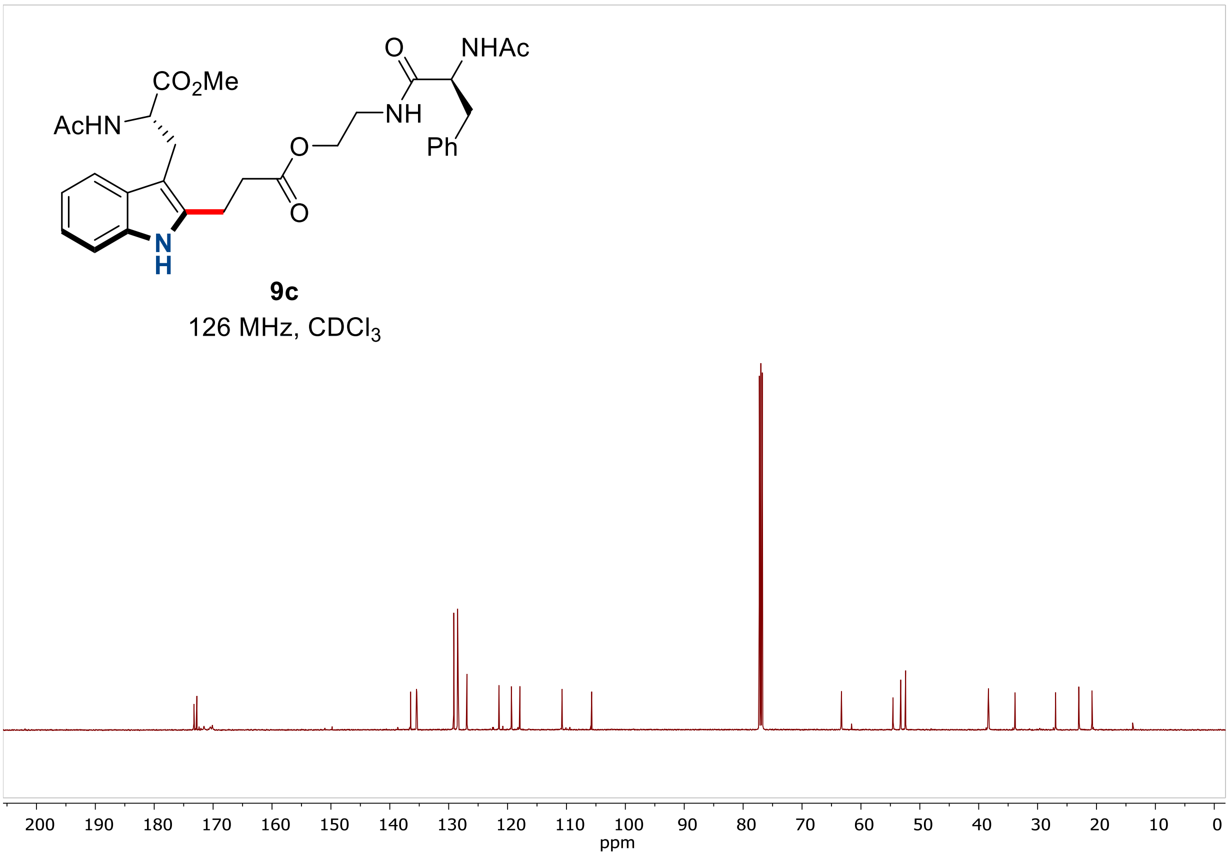

Supplementary Figure 152. ^{13}C-NMR spectrum of **9c**.

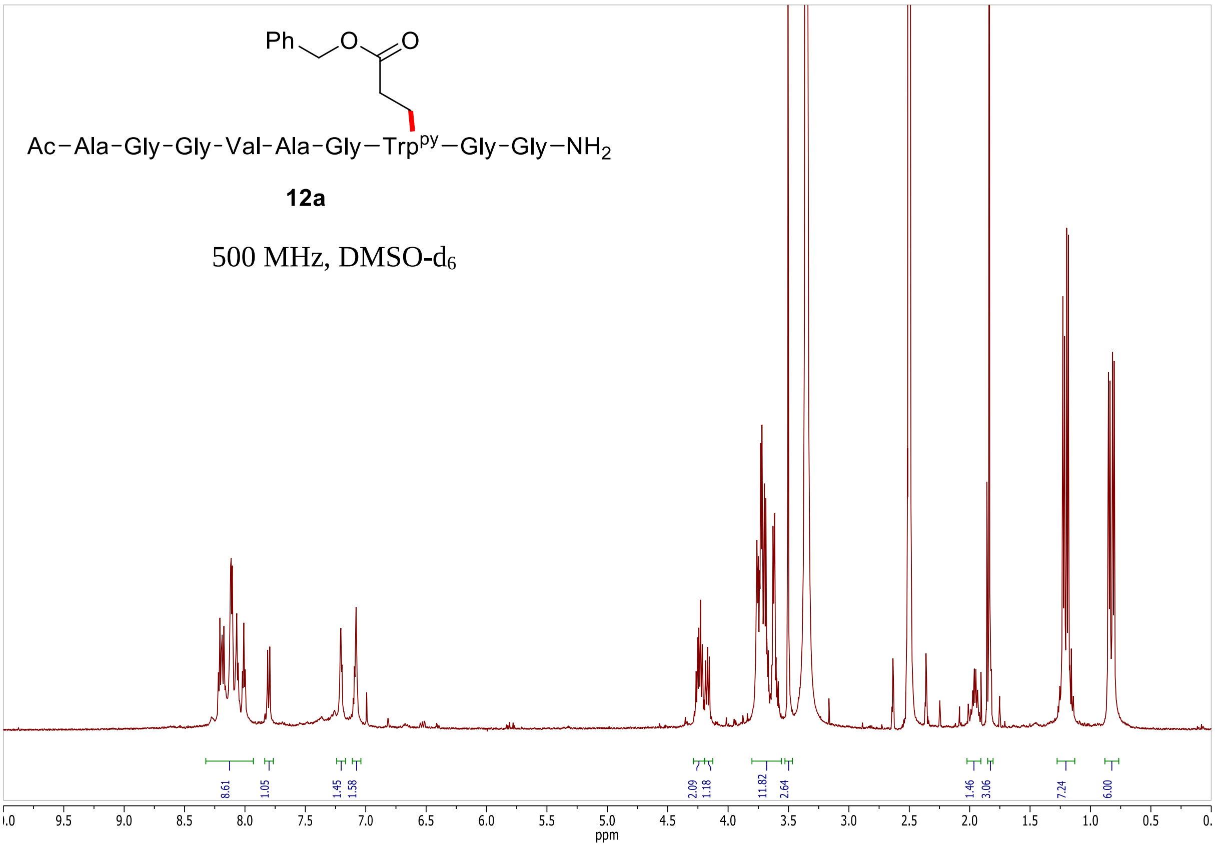

Supplementary Figure 153. ^{1}H-NMR spectrum of **12a**.

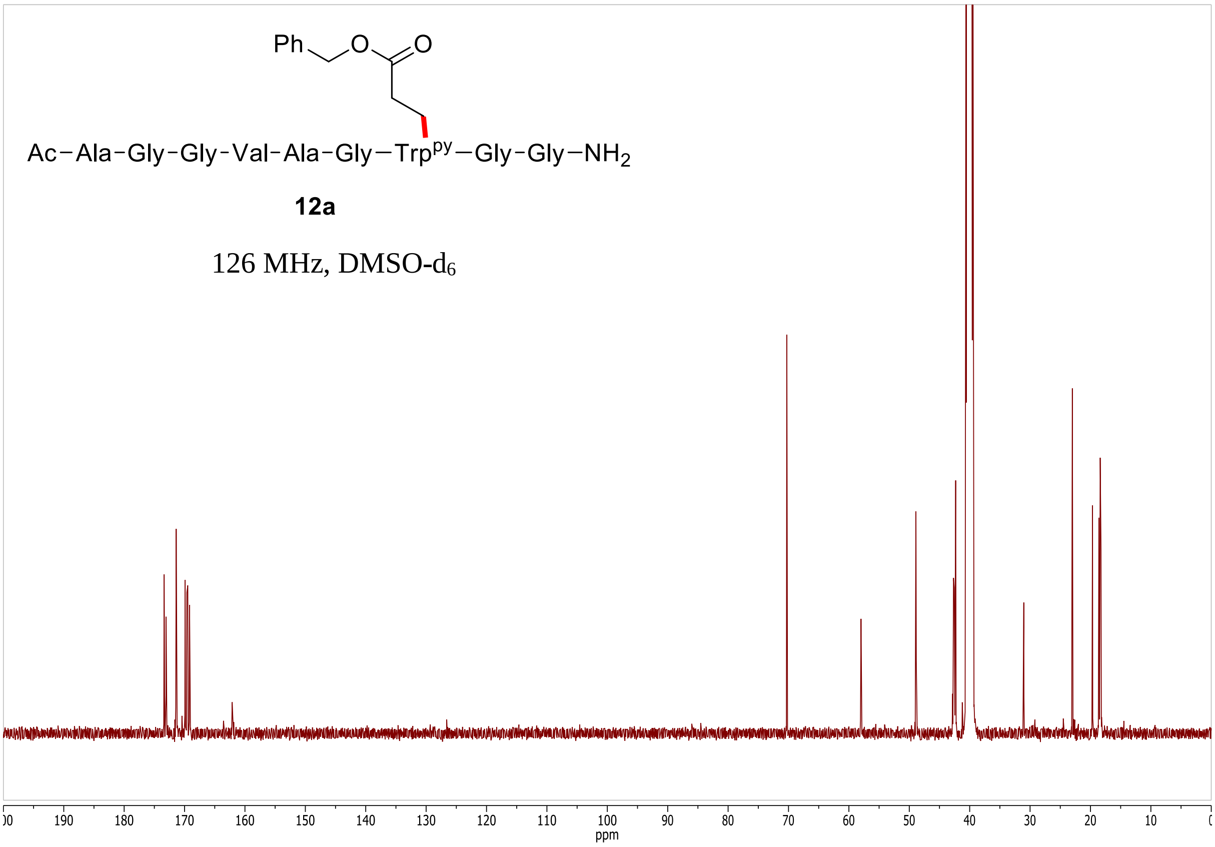

Supplementary Figure 154. ^{13}C-NMR spectrum of **12a**.

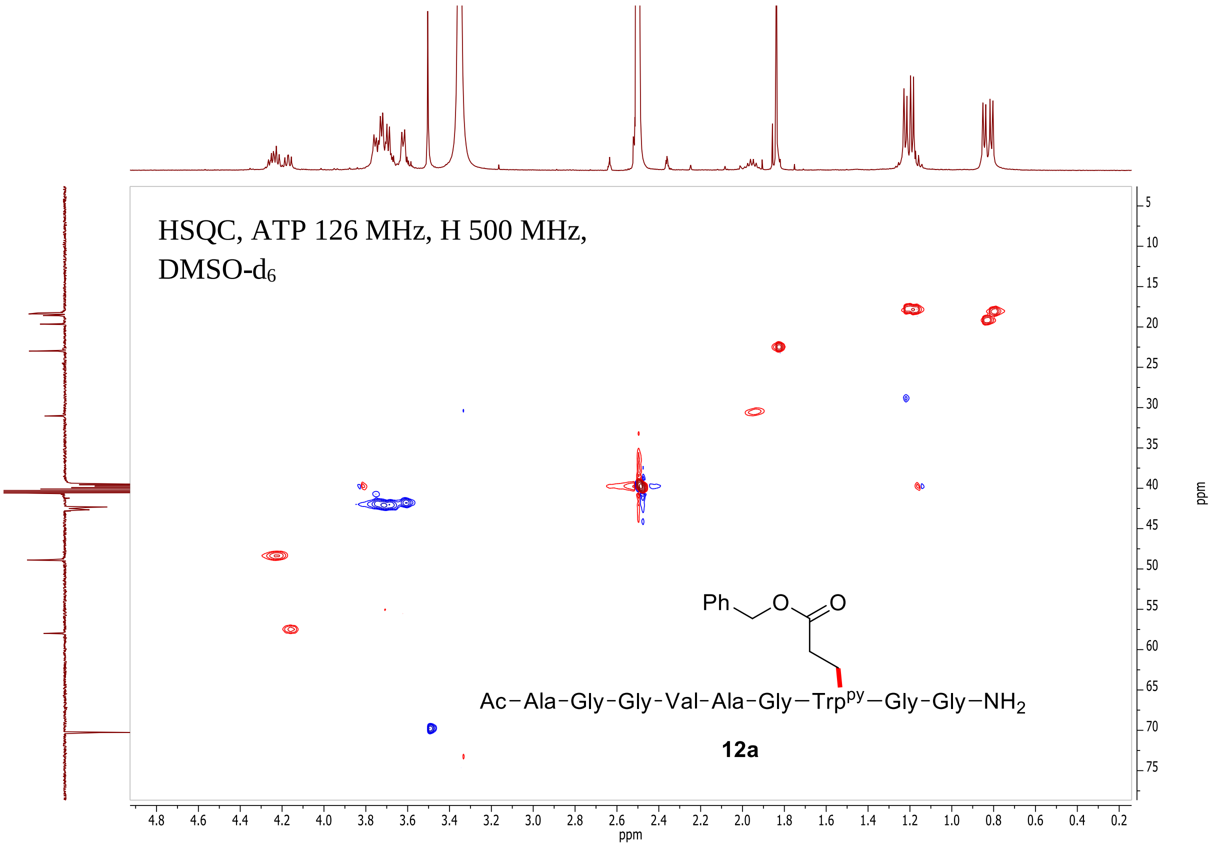

Supplementary Figure 155. HSQC: ATP, H spectrum of **12a**.

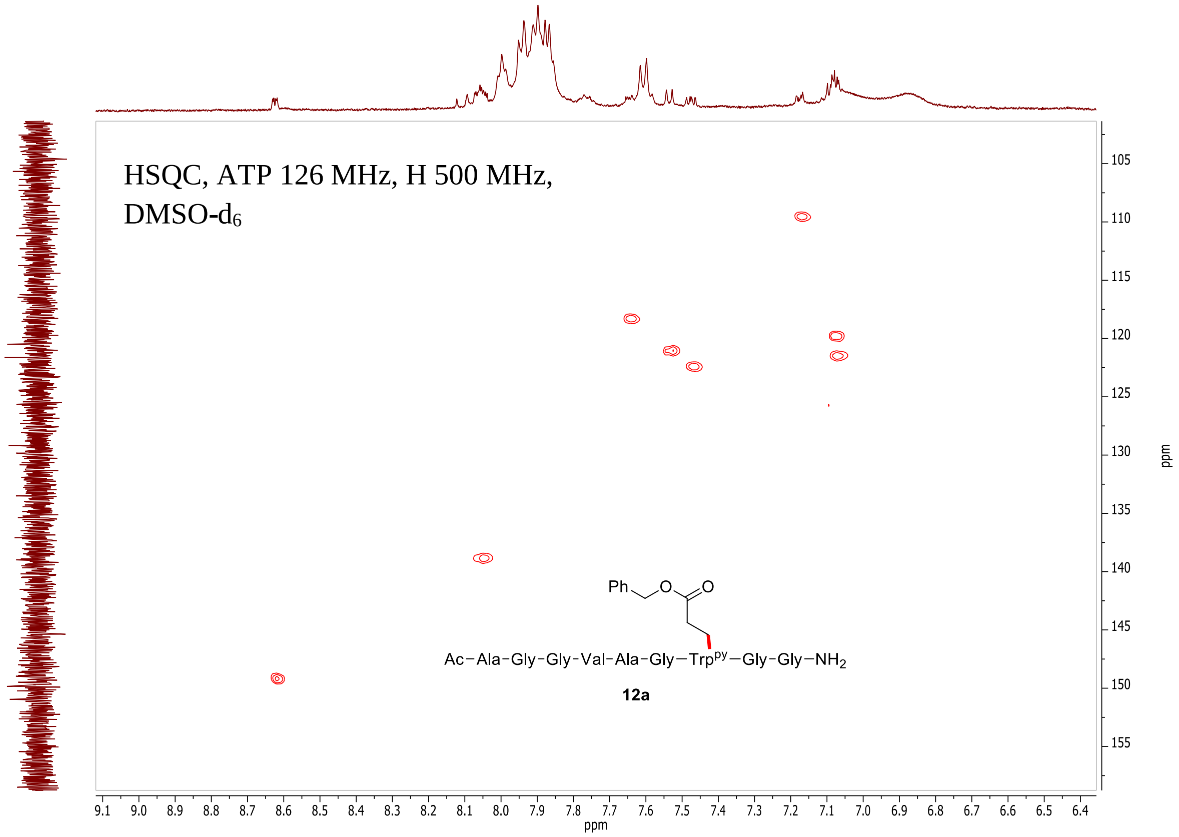

Supplementary Figure 156. HSQC: ATP, H spectrum of **12a**.

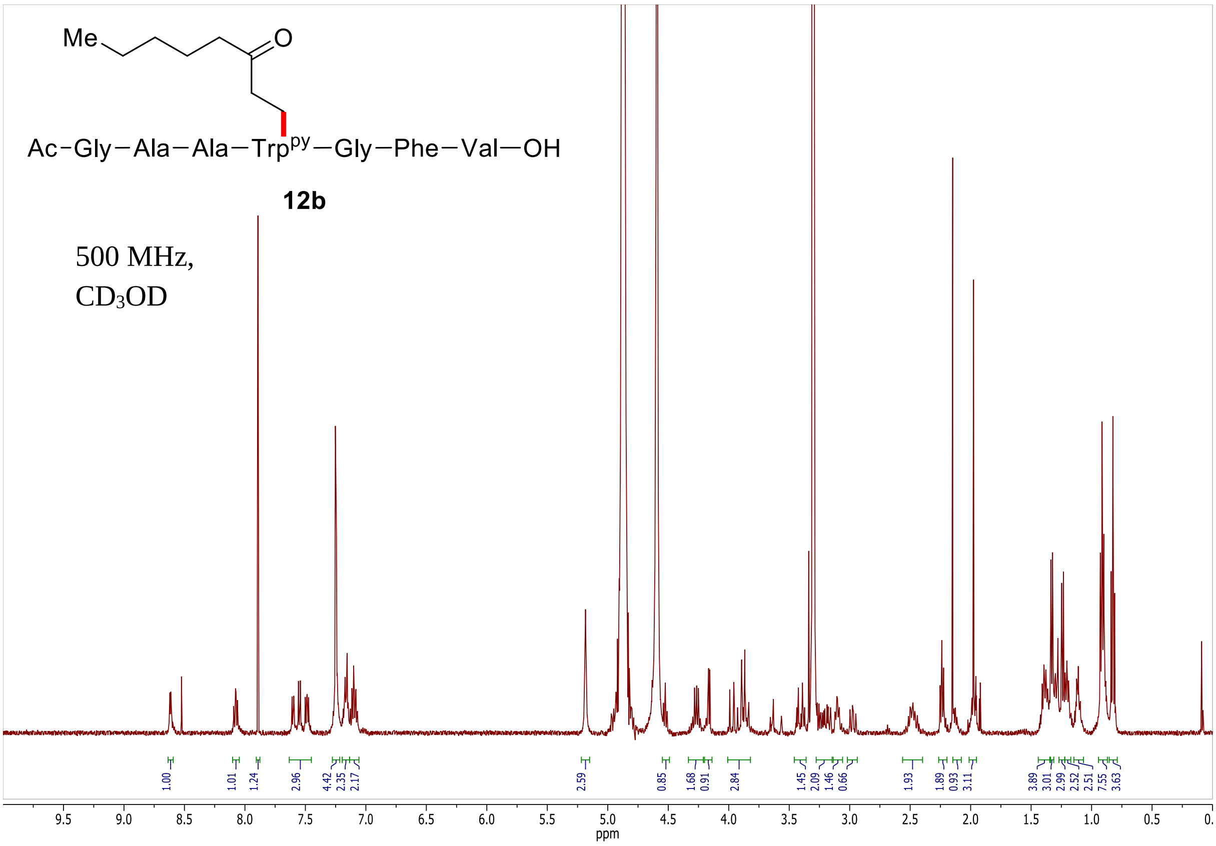

Supplementary Figure 157. ^{1}H-NMR spectrum of **12b**.

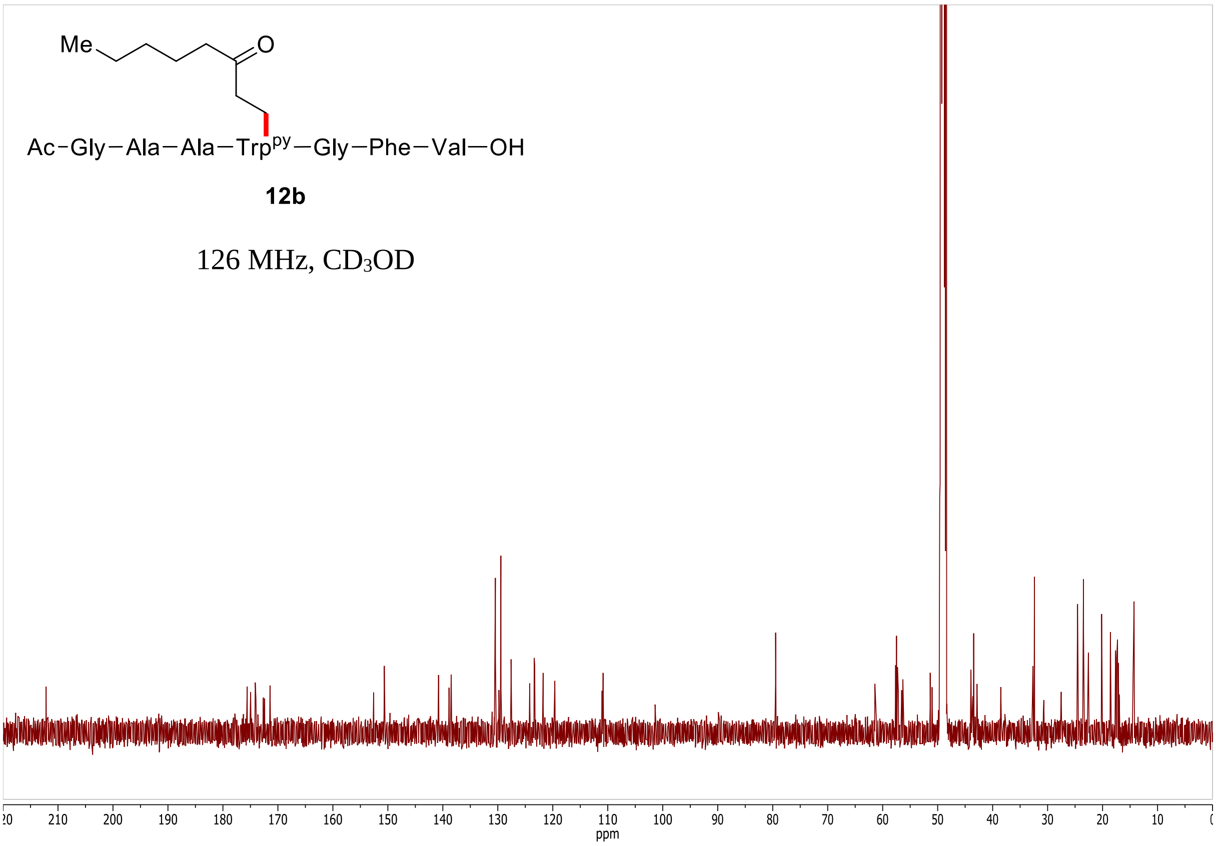

Supplementary Figure 158. ^{13}C-NMR spectrum of **12b**.

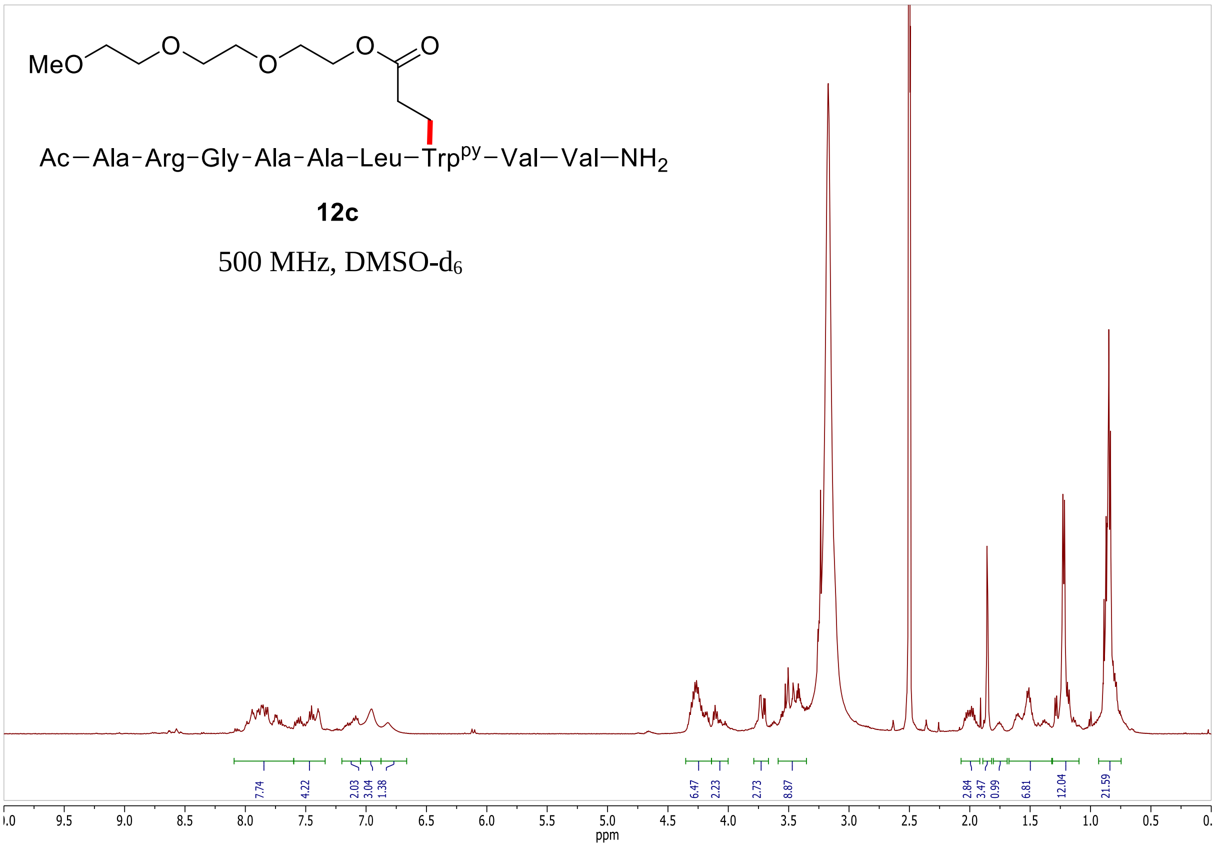

Supplementary Figure 159. ^{1}H-NMR spectrum of **12c**.

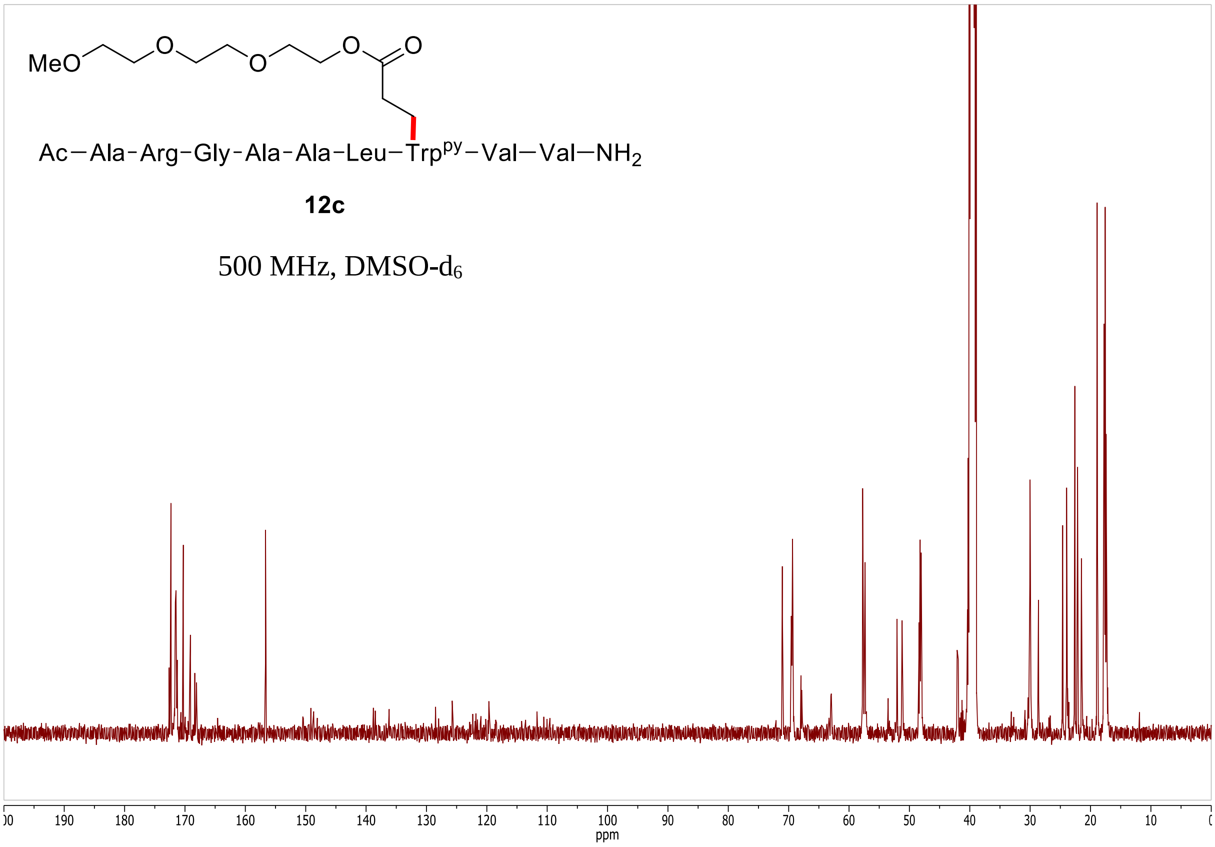

Supplementary Figure 160. ^{13}C-NMR spectrum of **12c**.

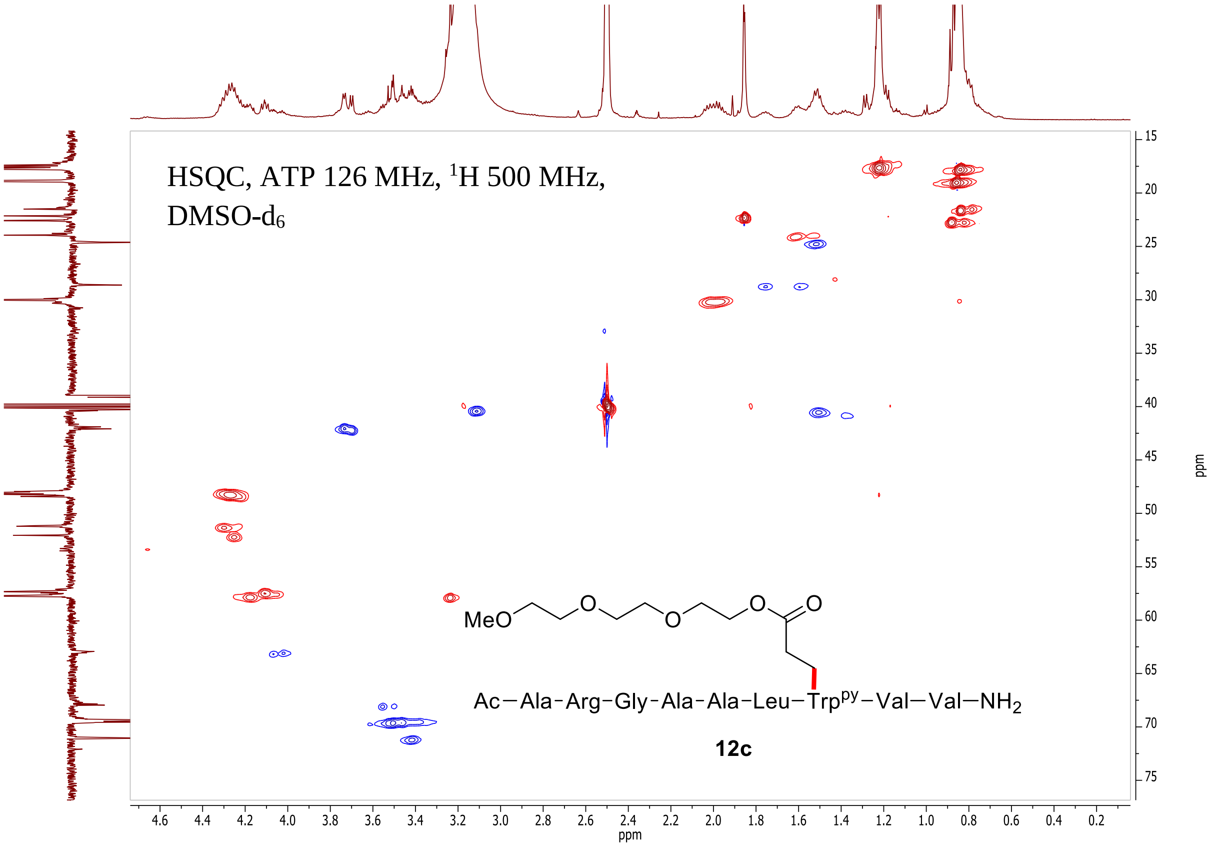

Supplementary Figure 161. HSQC: ATP, H spectrum of **12c**.

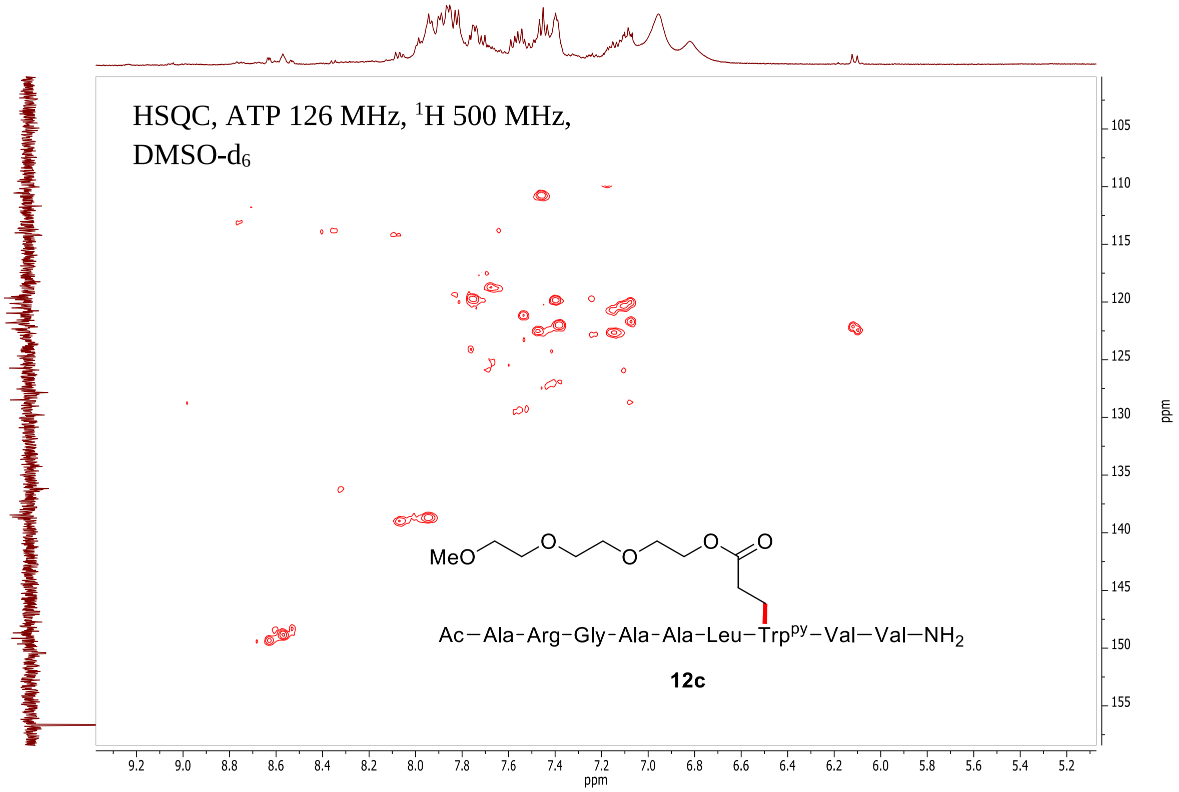

Supplementary Figure 162. HSQC: ATP, H spectrum of **12c**.

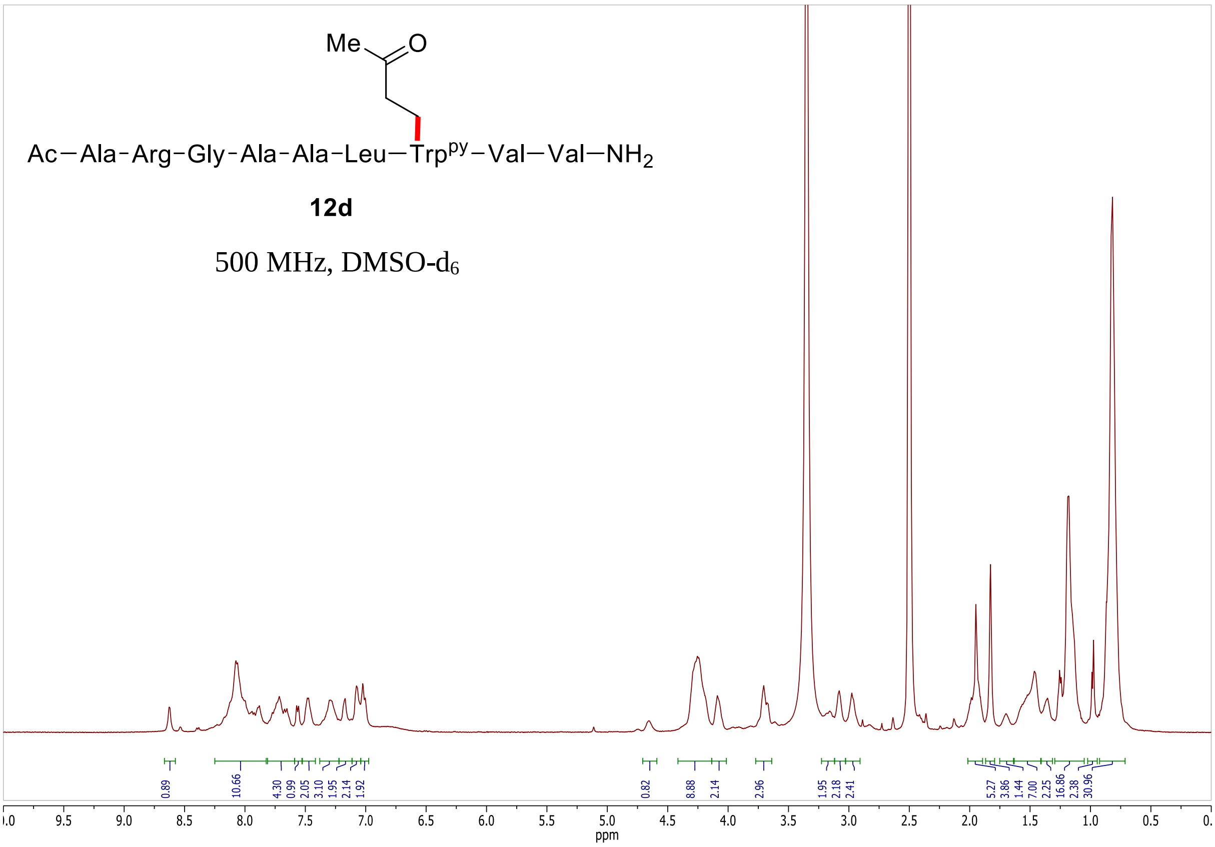

Supplementary Figure 163. ^{1}H-NMR spectrum of **12d**.

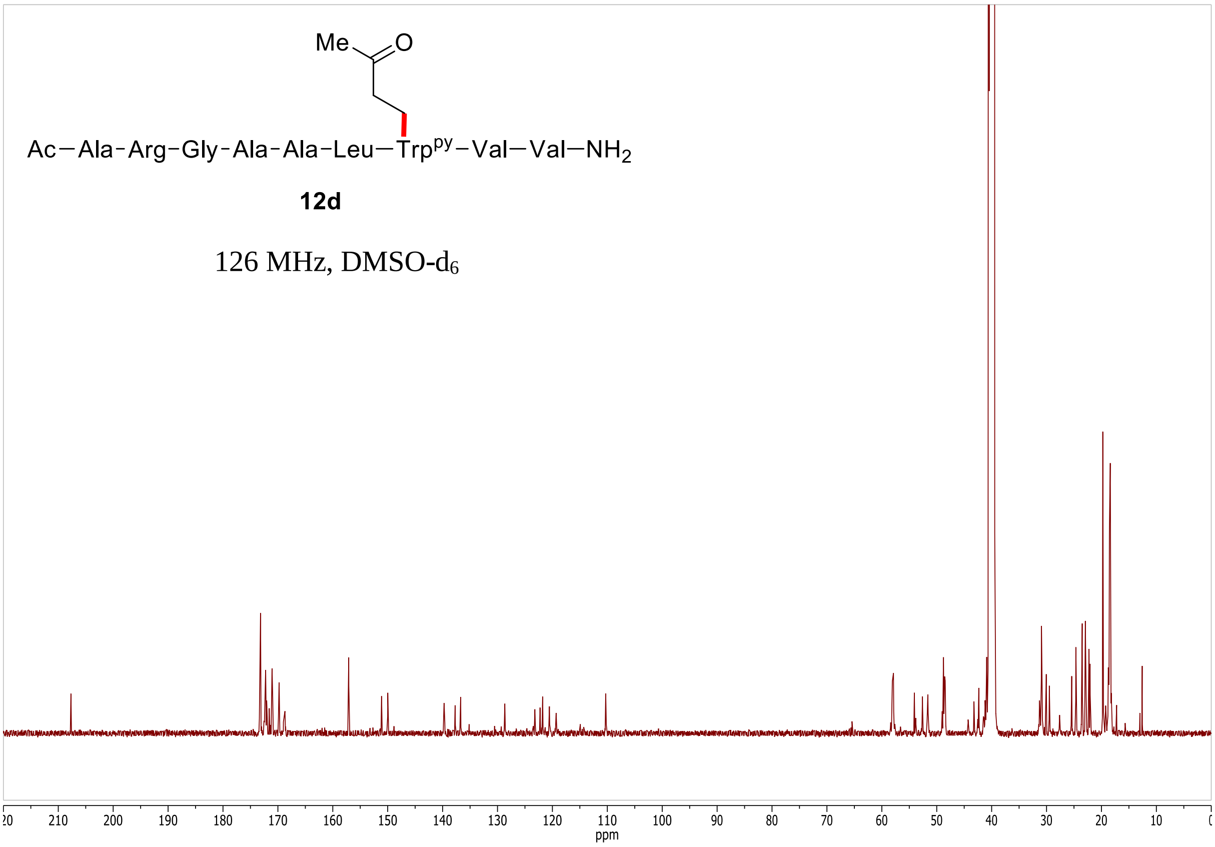

Supplementary Figure 164. ^{13}C-NMR spectrum of **12d**.

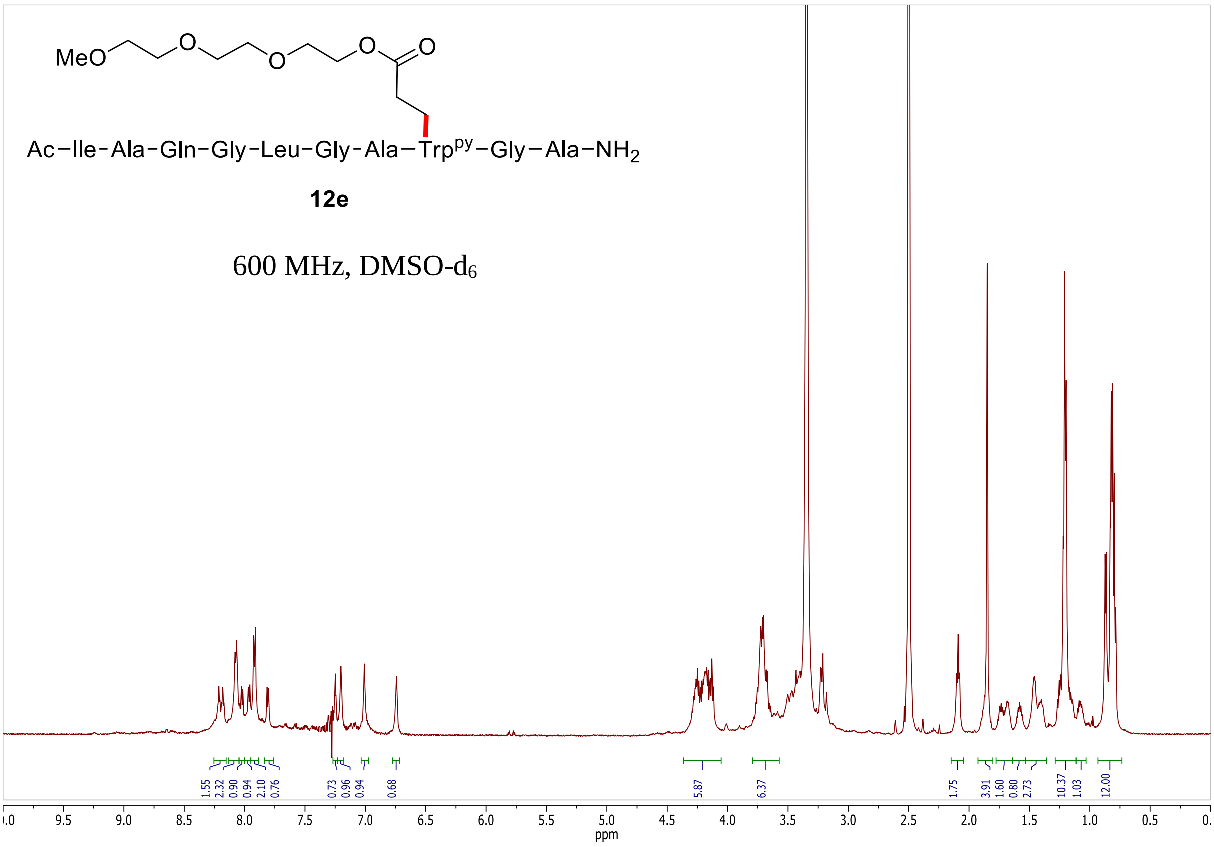

Supplementary Figure 165. ^{1}H-NMR spectrum of **12e**.

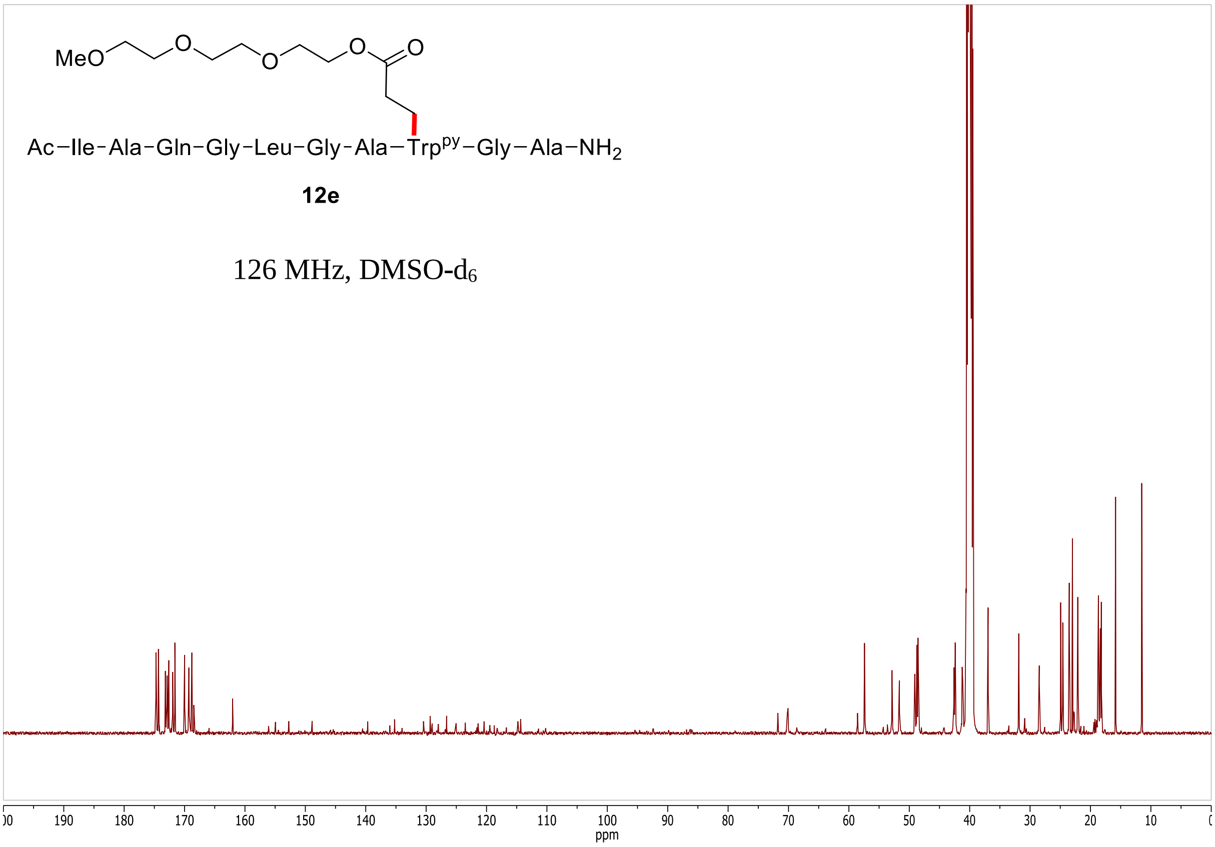

Supplementary Figure 166. ^{13}C-NMR spectrum of **12e**.

Supplementary References

1. Tiwari, V. K., Kamal, N., Kapur, M. Ruthenium-Catalyzed Heteroatom-Directed Regioselective C–H Arylation of Indoles Using a Removable Tether. *Org. Lett.* **17**, 1766–1769 (2015).
2. Ackermann, L., Lygin, A. V. Ruthenium-Catalyzed Direct C–H Bond Arylations of Heteroarenes. *Org. Lett.* **13**, 3332–3335 (2011).
3. Yang, X. *et al.* CuI/8-Hydroxyquinalidine Promoted *N*-Arylation of Indole and Azoles *Chin. J. Chem.*, **30**, 875–880 (2012).
4. Patil, P. H., Nallasivam, J. L., Fernandes, R. A. Unimolecular 4-Hydroxypiperidines: New Ligands for Copper-Catalyzed N-Arylation *Asian J. Org. Chem.* **4**, 552–559 (2015).
5. Shen, Z.-L. *et al.* Synthesis and application of a recyclable ionic liquid-supported imidazolidinone catalyst in enantioselective 1,3-dipolar cycloaddition *Chem. Commun.* **48**, 5856–5858 (2012).
6. Kumara, M. N., Gowda, D. C., Rangappa, K. S. Synthesis and kinetics of oxidation of some dipeptides with anodically generated manganese(III) sulphate: Mechanistic study *Int. J. Chem. Kinet.* **34**, 438–444 (2002).
7. Voshell, S. M., Lee, S. J., Gagné, M. R. The Discovery of an Enantioselective Receptor for (−)-Adenosine from a Racemic Dynamic Combinatorial Library *J. Am. Chem. Soc.* **128**, 12422–12423 (2006).
8. Novabiochem® *Peptide Synthesis*, Merck Millipore, **2014**/**2015**.
9. Schischko, A., Ren, H.; Kaplaneris, N., Ackermann, L. Bioorthogonal Diversification of Peptides through Selective Ruthenium(II)-Catalyzed C-H Activation *Angew. Chem. Int. Ed.* **56**, 1576–1580 (2017).
10. Frisch, M. J. *et al. Gaussian, Inc.*, Wallingford CT, **2016**.
11. Adamo, C. & Barone, V. Toward reliable density functional methods without adjustable parameters: The PBE0 model. *J. Chem. Phys.* **110**, 6158-6170 (1999).
12. Zhao, Y. & Truhlar, D. G. The M06 suite of density functionals for main group thermochemistry, thermochemical kinetics, noncovalent interactions, excited states, and transition elements: two new functionals and systematic testing of four M06-class functionals and 12 other functionals. *Theor Chem Acc* **120**, 215-241 (2008).
13. Grimme, S., Antony, J., Ehrlich, S. & Krieg, H. A consistent and accurate ab initio parametrization of density functional dispersion correction (DFT-D) for the 94 elements H-Pu. *J. Chem. Phys.* **132**, 154104 (2010).
14. Grimme, S., Ehrlich, S. & Goerigk, L. Effect of the damping function in dispersion corrected density functional theory. *J. Comput. Chem.* **32**, 1456-1465 (2011).
15. Weigend, F. & Ahlrichs, R. Balanced basis sets of split valence, triple zeta valence and quadruple zeta valence quality for H to Rn: Design and assessment of accuracy. *Phys. Chem. Chem. Phys.* **7**, 3297-3305 (2005).

16. Andrae, D., Häußermann, U., Dolg, M., Stoll, H. & Preuß, H. Energy-adjusted ab initio pseudopotentials for the second and third row transition elements. *Theor. Chim. Acta* **77**, 123-141 (1990).

17. Zhao, Y. & Truhlar, D. G. Design of Density Functionals That Are Broadly Accurate for Thermochemistry, Thermochemical Kinetics, and Nonbonded Interactions. *J. Phys. Chem. A* **109**, 5656-5667 (2005).

18. Marenich, A. V., Cramer, C. J. & Truhlar, D. G. Universal Solvation Model Based on Solute Electron Density and on a Continuum Model of the Solvent Defined by the Bulk Dielectric Constant and Atomic Surface Tensions. *J. Phys. Chem. B* **113**, 6378-6396 (2009).
