# Supplementary material for: Late-stage peptide C–H alkylation for bioorthogonal C–H activation featuring solid phase peptide synthesis
Source: Nat Commun. 2019 Aug 7;10:3553. doi: 10.1038/s41467-019-11395-3 (PMC6685959; doi:10.1038/s41467-019-11395-3)
Supplement: Supplementary file 2 — Description of Additional Supplementary Files [file 41467_2019_11395_MOESM2_ESM.pdf]

Description of Additional Supplementary Files

File Name: Supplementary Data 1

Description: Cartesian coordinates and energy values for all calculated structures.
