# Supplementary material for: Late-stage peptide C–H alkylation for bioorthogonal C–H activation featuring solid phase peptide synthesis
Source: Nat Commun. 2019 Aug 7;10:3553. doi: 10.1038/s41467-019-11395-3 (PMC6685959; doi:10.1038/s41467-019-11395-3)
Supplement: Supplementary file 3 — Supplementary Data 1 [file 41467_2019_11395_MOESM3_ESM.pdf]

Cartesian Coordinates and Energies

(PBE0-D3(BJ)/def2-QZVP*+SMD(AcOH)/PBE0-D3(BJ)/def2-SVP level of theory)

Intermediate **A**

E(SMD/def2-QZVP*) = -1322.919154 E_h

ΔΔG(353) = 0.396571 E_h

Lowest frequency = 22.96 cm^{-1}

C	0.870786	0.416361	1.779894
C	-1.166562	1.483725	2.072342
C	-0.652466	2.303816	3.063897
C	0.690470	2.172181	3.412393
C	1.455151	1.207542	2.771347
Ru	-1.268052	-0.305841	-0.319330
C	0.252213	0.574196	-1.618035
C	0.256352	-0.823239	-1.789690
C	-0.953674	-1.524016	-2.093205
C	-2.145643	-0.772976	-2.240708
C	-2.134585	0.633390	-2.022135
C	-0.942022	1.344918	-1.721047
H	1.175088	-1.382934	-1.604470
H	-3.087066	1.169690	-2.000182
H	2.498390	1.029841	3.035477
H	1.131398	2.796700	4.192389
H	-1.301712	3.033252	3.549681
H	-2.212945	1.541685	1.768767
N	-0.416716	0.576789	1.417002
C	2.832896	-0.322632	0.471541
C	1.374669	-1.925694	1.126673
C	3.320410	-1.576828	0.028247
C	3.485920	0.879667	0.185264
C	2.380633	-2.573190	0.470666
C	4.510303	-1.618962	-0.715069
C	4.660534	0.804706	-0.554259
H	3.099729	1.842016	0.528456
H	2.456111	-3.649646	0.332145
C	5.167767	-0.430051	-0.997241
H	4.909621	-2.573295	-1.065189
H	5.201604	1.723062	-0.791839
H	6.095552	-0.449268	-1.572522
N	1.632639	-0.557201	1.134385
O	-3.036059	-0.315197	0.832301
C	-2.748843	-1.437392	1.353166
O	-1.685360	-1.983354	0.936323
C	-3.587823	-2.046694	2.417450
H	-3.514274	-3.140658	2.382777
H	-3.212365	-1.710026	3.396789
H	-4.630425	-1.720771	2.318358

H	0.475844	-2.311632	1.602213
H	1.179015	1.050966	-1.294346
H	-3.095411	-1.287587	-2.396050
C	-0.957445	-3.014481	-2.157654
H	-0.646583	-3.336192	-3.164578
H	-0.248247	-3.434529	-1.431891
H	-1.955135	-3.424836	-1.956921
C	-0.980951	2.843289	-1.521410
H	-1.896656	3.056370	-0.942087
C	-1.114141	3.524871	-2.886094
H	-2.003854	3.178461	-3.432268
H	-1.196537	4.614741	-2.764171
H	-0.232260	3.320455	-3.513095
C	0.210588	3.395622	-0.749412
H	0.068613	4.469583	-0.563499
H	0.342246	2.904222	0.225365
H	1.147209	3.289869	-1.318987

Transition state **TS1**

E(SMD/def2-QZVP*) = -1322.898926 E_h

ΔΔG(353) = 0.393381 E_h

Lowest frequency = -99.57 cm^{-1}

C	-1.059558	1.015367	-1.546047
C	0.834108	2.349235	-1.411639
C	0.212565	3.369332	-2.116505
C	-1.106710	3.189083	-2.534301
C	-1.751212	1.987104	-2.266877
Ru	1.037852	-0.227572	0.196763
C	-0.236146	0.012917	1.978062
C	0.159116	-1.330534	1.856112
C	1.530851	-1.728641	1.732297
C	2.496551	-0.701547	1.712874
C	2.113905	0.669682	1.775914
C	0.742502	1.048651	1.912881
H	-0.615912	-2.094540	1.779125
H	2.874571	1.444008	1.653951
H	-2.753723	1.775403	-2.639135
H	-1.620457	3.969688	-3.099535
H	0.760327	4.285047	-2.341820
H	1.873097	2.413911	-1.083697
N	0.190336	1.217983	-1.095191
C	-2.771469	-0.577281	-0.665375
C	-0.732655	-1.365442	-1.262375
C	-2.710488	-1.959610	-0.336200
C	-3.873490	0.216307	-0.331315
C	-1.423549	-2.432469	-0.737584
C	-3.809288	-2.565851	0.300188
C	-4.935942	-0.410770	0.304412
H	-3.908803	1.282953	-0.557370
H	-1.045481	-3.451763	-0.684402

C	-4.910331	-1.786843	0.610061
H	-3.788043	-3.629868	0.545243
H	-5.816862	0.177246	0.570876
H	-5.773825	-2.238714	1.101980
N	-1.570000	-0.243721	-1.264603
O	2.569473	-0.016304	-1.158288
C	2.824602	-1.140496	-1.749856
O	2.152726	-2.148858	-1.536857
C	4.003127	-1.130981	-2.681196
H	3.937023	-1.974309	-3.378473
H	4.070405	-0.179618	-3.224498
H	4.924386	-1.239880	-2.088078
H	0.189424	-1.401901	-1.851797
H	-1.299306	0.252649	2.012559
H	3.538960	-0.952977	1.505590
C	1.905354	-3.163285	1.550841
H	2.131502	-3.616461	2.529252
H	1.084839	-3.729218	1.092084
H	2.783529	-3.261353	0.900741
C	0.389311	2.512964	2.014099
H	1.046607	3.045426	1.305160
C	0.741254	2.991752	3.427339
H	1.800563	2.821296	3.668506
H	0.541682	4.068962	3.522311
H	0.133413	2.467857	4.181038
C	-1.057108	2.841140	1.670485
H	-1.201074	3.930761	1.672073
H	-1.349135	2.466788	0.679603
H	-1.754947	2.424526	2.413078

Intermediate **B**

E(SMD/def2-QZVP*) = -1322.902812 E_h

ΔΔG(353) = 0.394631 E_h

Lowest frequency = 13.04 cm^{-1}

C	-1.137809	1.022932	-1.663702
C	0.201566	2.774323	-0.952284
C	-0.588835	3.696420	-1.627106
C	-1.703773	3.241359	-2.329103
C	-1.976471	1.876633	-2.369423
Ru	0.913533	0.023873	0.238679
C	-0.390191	-0.480110	1.942280
C	0.595291	-1.489938	1.775268
C	1.985300	-1.212927	1.742477
C	2.360068	0.158463	1.828621
C	1.399855	1.184625	1.982734
C	0.000925	0.873900	2.049392
H	0.266283	-2.519515	1.624357
H	1.724221	2.227196	2.005498
H	-2.794720	1.467539	-2.962721
H	-2.339908	3.942498	-2.873541

H	-0.323711	4.754213	-1.607023
H	1.098749	3.070634	-0.406163
N	-0.095890	1.470896	-0.937130
C	-2.247491	-1.129375	-1.076855
C	-0.035334	-1.083681	-1.574498
C	-1.679680	-2.374806	-0.688289
C	-3.582551	-0.815519	-0.809686
C	-0.295224	-2.329944	-1.027613
C	-2.484855	-3.343059	-0.059736
C	-4.348595	-1.788584	-0.182872
H	-4.015769	0.145879	-1.089162
H	0.448627	-3.121829	-0.955149
C	-3.812763	-3.040985	0.183727
H	-2.067171	-4.311730	0.223410
H	-5.400718	-1.581251	0.025049
H	-4.460290	-3.777772	0.662622
N	-1.247663	-0.368153	-1.655000
O	2.415642	0.479398	-1.068826
C	3.136378	-0.494123	-1.554132
O	2.886801	-1.677417	-1.376195
C	4.327172	-0.027643	-2.351993
H	4.718773	-0.853036	-2.957766
H	4.065338	0.829701	-2.986109
H	5.113919	0.306974	-1.658229
H	0.786122	-0.828696	-2.241934
H	-1.445237	-0.754349	1.914257
H	3.407077	0.431825	1.679522
C	3.000352	-2.298956	1.600578
H	3.359207	-2.597330	2.598790
H	2.572587	-3.179931	1.106586
H	3.855540	-1.969996	0.998764
C	-0.987214	1.998074	2.241011
H	-0.597526	2.861699	1.675172
C	-0.992862	2.379635	3.725399
H	0.010797	2.650436	4.084636
H	-1.656153	3.240602	3.893150
H	-1.358792	1.544440	4.342138
C	-2.391180	1.696152	1.739667
H	-3.017880	2.595850	1.817242
H	-2.392590	1.372547	0.689883
H	-2.879384	0.910751	2.336744

Intermediate **C**

E(SMD/def2-QZVP*) = -1322.921971 E_h

ΔΔG(353) = 0.395220 E_h

Lowest frequency = 20.75 cm^{-1}

C	1.119389	-0.908314	1.437209
C	-0.894385	-1.508153	2.449840
C	-0.247396	-2.160753	3.482779
C	1.149168	-2.185382	3.469362

C	1.841502	-1.564496	2.443049
Ru	-1.185668	0.026387	-0.143312
C	-1.397998	2.170877	0.079001
C	-1.681941	1.806751	-1.277509
C	-2.633259	0.817959	-1.582684
C	-3.438817	0.209783	-0.554866
C	-3.197492	0.583912	0.771484
C	-2.142181	1.500964	1.083061
C	-0.352212	3.215750	0.381622
H	-1.081267	2.239041	-2.080541
H	-2.758259	0.496234	-2.618710
H	-3.761825	0.111863	1.578320
H	-1.900583	1.685645	2.131289
H	0.408236	3.122782	-0.411823
H	2.925587	-1.602605	2.406318
H	1.701829	-2.700392	4.258059
H	-0.823980	-2.645417	4.270744
H	-1.982984	-1.473406	2.389575
N	-0.234664	-0.889186	1.458698
C	2.977074	-0.019406	-0.045547
C	0.755125	0.208236	-0.627881
C	2.901896	0.586603	-1.324928
C	4.207011	-0.235579	0.578260
C	1.504595	0.714739	-1.661957
C	4.073213	0.953399	-1.995372
C	5.358998	0.140367	-0.108161
H	4.302945	-0.661200	1.574783
H	1.112430	1.155853	-2.577167
C	5.297481	0.722369	-1.381164
H	4.021173	1.418368	-2.982134
H	6.330307	-0.019786	0.364206
H	6.222299	1.002905	-1.889179
N	1.655913	-0.263156	0.353111
O	-1.262956	-1.958160	-0.910354
C	-0.570869	-2.599874	-1.709216
O	0.499955	-2.138444	-2.275782
H	0.710285	-1.217435	-1.953450
C	-0.929767	-3.993373	-2.092430
H	-1.024687	-4.058973	-3.186121
H	-0.110327	-4.668036	-1.803045
H	-1.861126	-4.300007	-1.606110
C	0.348589	3.011256	1.717568
H	1.176409	3.727183	1.818561
H	-0.327375	3.182290	2.570122
H	0.769310	1.998448	1.801732
C	-0.988004	4.603718	0.278795
H	-0.227241	5.384980	0.421693
H	-1.457980	4.766652	-0.702724
H	-1.762312	4.740783	1.049994
C	-4.428968	-0.846665	-0.917793

H	-5.263679	-0.404200	-1.483703
H	-3.959292	-1.609454	-1.555726
H	-4.837730	-1.342038	-0.027924

Intermediate **D**

E(SMD/def2-QZVP*) = -1400.244897 E_h

ΔΔG(353) = 0.428333 E_h

Lowest frequency = 9.66 cm^{-1}

C	-1.083171	1.168299	0.857600
C	1.067433	1.675460	1.612911
C	0.597494	2.630697	2.491564
C	-0.781090	2.864631	2.530643
C	-1.625874	2.139261	1.712865
Ru	0.940983	-0.516685	-0.456517
C	0.664743	-2.643154	0.237902
C	1.214578	-2.617038	-1.072061
C	2.442691	-1.956920	-1.335585
C	3.104168	-1.313898	-0.245441
C	2.499900	-1.252359	1.013476
C	1.259623	-1.927496	1.291038
H	0.690469	-3.121342	-1.885661
H	2.987470	-0.694363	1.816389
H	-2.695412	2.319374	1.725135
H	-1.196274	3.621093	3.199915
H	1.294866	3.185128	3.119610
H	2.134494	1.483221	1.502827
N	0.254466	0.954797	0.820424
C	-3.159375	0.184322	-0.217065
C	-1.052528	-0.564314	-0.757053
C	-3.267569	-0.859404	-1.169945
C	-4.291690	0.820749	0.290641
C	-1.932321	-1.303085	-1.490231
C	-4.528913	-1.264724	-1.616814
C	-5.537299	0.398412	-0.169370
H	-4.248816	1.626982	1.019333
H	-1.677847	-2.075072	-2.213627
C	-5.657545	-0.630470	-1.111329
H	-4.621203	-2.066129	-2.352988
H	-6.435117	0.886591	0.214808
H	-6.649507	-0.934226	-1.451935
N	-1.785336	0.351588	0.024094
C	1.693538	1.118900	-1.713283
H	2.759397	0.948999	-1.884838
C	0.716622	0.408394	-2.420723
H	0.992790	-0.324175	-3.181276
H	-0.259776	0.877623	-2.551432
C	1.367572	2.492432	-1.234577
O	0.275191	2.996942	-1.265422
O	2.459624	3.104530	-0.770988
H	-0.299333	-3.129077	0.391145

H	4.049433	-0.793950	-0.418464
C	0.675555	-1.868313	2.681492
H	0.870246	-0.845982	3.050617
C	1.430735	-2.846500	3.586418
H	2.513303	-2.649618	3.597284
H	1.064072	-2.767584	4.620065
H	1.279097	-3.884606	3.252424
C	-0.827919	-2.106288	2.723882
H	-1.369639	-1.453682	2.023615
H	-1.083468	-3.150482	2.485796
H	-1.208313	-1.908957	3.735856
C	3.066579	-1.990286	-2.694007
H	3.503186	-1.019586	-2.968396
H	3.882473	-2.730261	-2.700903
H	2.344500	-2.284730	-3.466340
C	2.287238	4.454829	-0.358421
H	3.273711	4.806230	-0.037121
H	1.915314	5.067382	-1.191631
H	1.565151	4.516080	0.469019

Transition state **TS3**

E(SMD/def2-QZVP*) = -1400.225382 E_h

ΔΔG(353) = 0.429702 E_h

Lowest frequency = -294.19 cm^{-1}

C	-0.944450	1.445406	0.700266
C	1.144630	1.576319	1.713274
C	0.757868	2.647124	2.496555
C	-0.533469	3.151925	2.331535
C	-1.390678	2.558701	1.420552
Ru	0.857050	-0.622098	-0.366952
C	0.132678	-2.640704	0.265559
C	0.739237	-2.701226	-1.022563
C	2.066669	-2.261272	-1.246260
C	2.780541	-1.719833	-0.129413
C	2.155751	-1.597730	1.121544
C	0.812947	-2.060884	1.349498
H	0.157679	-3.080170	-1.865481
H	2.694076	-1.114256	1.940141
H	-2.379114	2.969633	1.232494
H	-0.864773	4.024276	2.898637
H	1.463340	3.093424	3.197656
H	2.160764	1.181878	1.749878
N	0.314395	0.976909	0.844796
C	-3.074423	0.615563	-0.368214
C	-1.031443	-0.141388	-1.089812
C	-3.279236	-0.353287	-1.386269
C	-4.148095	1.194727	0.309836
C	-1.992634	-0.804070	-1.820356
C	-4.584742	-0.705707	-1.758718
C	-5.432537	0.820963	-0.075036

H	-4.018105	1.904939	1.124177
H	-1.800053	-1.507014	-2.628719
C	-5.651535	-0.109647	-1.102726
H	-4.752880	-1.443179	-2.546340
H	-6.287668	1.264971	0.438628
H	-6.674610	-0.371308	-1.379885
N	-1.692799	0.748483	-0.215241
C	1.650152	0.820223	-1.722477
H	2.457361	0.322671	-2.264024
C	0.305868	0.681864	-2.210149
H	0.160497	0.082456	-3.110820
H	-0.278856	1.608705	-2.191773
C	2.036448	2.130399	-1.149062
O	1.283303	3.053432	-0.952938
O	3.341369	2.169158	-0.860069
H	-0.909780	-2.941685	0.371869
H	3.788095	-1.322815	-0.270586
C	0.192758	-1.892087	2.715378
H	0.576593	-0.934713	3.109479
C	0.687818	-3.010806	3.635889
H	1.786337	-3.043386	3.689981
H	0.304191	-2.864198	4.656039
H	0.338240	-3.992455	3.280476
C	-1.327613	-1.811182	2.689437
H	-1.686526	-1.054084	1.976301
H	-1.784205	-2.776449	2.421193
H	-1.706639	-1.545856	3.686406
C	2.706127	-2.378806	-2.593224
H	3.481810	-1.616142	-2.745625
H	3.192094	-3.363041	-2.685981
H	1.965662	-2.296148	-3.400696
C	3.831905	3.406986	-0.363258
H	4.907593	3.271317	-0.206336
H	3.650924	4.214444	-1.086782
H	3.335983	3.672610	0.582027

Intermediate **E**

E(SMD/def2-QZVP*) = -1400.248995 E_h

ΔΔG(353) = 0.432568 E_h

Lowest frequency = 19.39 cm^{-1}

C	-0.729149	2.077433	-0.628680
C	0.900035	2.247971	1.000349
C	0.589216	3.583668	1.219284
C	-0.425736	4.172267	0.471679
C	-1.085562	3.409633	-0.488850
Ru	0.480871	-0.626215	-0.097750
C	-0.927783	-1.801254	1.236212
C	-0.258166	-2.643743	0.299233
C	1.151869	-2.704275	0.182547
C	1.902132	-1.818691	1.016252

C	1.247148	-0.951890	1.914348
C	-0.184577	-0.937201	2.053807
H	-0.864432	-3.257727	-0.370594
H	1.845719	-0.257229	2.507949
H	-1.843271	3.833691	-1.148074
H	-0.680651	5.225113	0.609139
H	1.159096	4.154288	1.953321
H	1.719967	1.769056	1.533079
N	0.228215	1.489234	0.125026
C	-2.522789	0.553937	-1.296234
C	-0.389827	0.244596	-2.033593
C	-2.377693	-0.812397	-1.613292
C	-3.702137	1.066804	-0.768404
C	-1.004174	-1.000176	-2.041689
C	-3.457958	-1.684476	-1.451059
C	-4.763072	0.179444	-0.596344
H	-3.803418	2.121508	-0.507520
H	-0.604305	-1.879751	-2.544074
C	-4.648343	-1.175558	-0.940665
H	-3.372161	-2.739873	-1.720333
H	-5.709090	0.552094	-0.197970
H	-5.508169	-1.835669	-0.810659
N	-1.304449	1.206545	-1.567014
C	1.910340	-0.145059	-1.599909
H	2.291976	-1.087688	-2.011174

C	0.979659	0.603819	-2.538146
H	1.092638	0.332545	-3.599385
H	1.145885	1.686327	-2.451724
C	3.012848	0.654782	-1.023993
O	3.026852	1.860019	-0.906785
O	4.024663	-0.118711	-0.589785
H	-2.017842	-1.783234	1.250118
H	2.985005	-1.754009	0.896418
C	-0.818561	-0.003450	3.053927
H	-0.173975	0.891450	3.098309
C	-0.786127	-0.664821	4.435366
H	0.234032	-0.955406	4.727380
H	-1.172820	0.026434	5.198396
H	-1.412012	-1.570352	4.448952
C	-2.220570	0.448542	2.673064
H	-2.250325	0.874336	1.659754
H	-2.944272	-0.380166	2.710981
H	-2.573273	1.214796	3.377956
C	1.831280	-3.629748	-0.774629
H	2.773678	-3.200166	-1.141776
H	2.072569	-4.579951	-0.272417
H	1.190449	-3.862433	-1.636364
C	5.131148	0.565646	-0.021808
H	5.881167	-0.198943	0.210586
H	5.542326	1.298533	-0.730072

H	4.838008	1.100130	0.894638

Intermediate **F**

E(SMD/def2-QZVP*) = -1629.209186 E_h

ΔΔG(353) = 0.487612 E_h

Lowest frequency = 19.48 cm^{-1}

C	1.603423	0.534662	1.568561
C	-0.450842	1.381488	2.188538
C	0.068598	1.999447	3.311197
C	1.431936	1.863057	3.573204
C	2.199846	1.118663	2.695083
Ru	-0.735359	0.171651	-0.510448
C	-0.979122	2.354164	-1.088014
C	0.280953	1.788735	-1.459529
C	0.393590	0.648296	-2.298541
C	-0.829317	-0.003714	-2.659429
C	-2.063813	0.472148	-2.184608
C	-2.166471	1.690389	-1.422486
H	1.201132	2.249518	-1.090485
H	-2.961145	-0.120473	-2.375646
H	3.264619	0.954793	2.863530
H	1.885012	2.317563	4.456805
H	-0.587562	2.577695	3.962907
H	-1.506422	1.479574	1.939404
N	0.286796	0.663122	1.317530
C	3.666259	0.198961	0.265935
C	2.225327	-1.555729	0.396652
C	4.286666	-0.930312	-0.317678
C	4.276012	1.454871	0.292912
C	3.356341	-2.020511	-0.210702
C	5.563497	-0.792386	-0.880103
C	5.543277	1.562502	-0.270323
H	3.784450	2.326362	0.730528
H	3.503261	-3.040134	-0.561375
C	6.180572	0.451119	-0.847475
H	6.060502	-1.649480	-1.339499
H	6.049236	2.530194	-0.265909
H	7.175487	0.572112	-1.280807
N	2.395451	-0.194701	0.688371
C	-0.021807	-1.834903	-0.515147
C	0.921745	-2.232176	0.620157
H	1.099811	-3.318917	0.620737
H	0.512061	-1.985632	1.613927
H	-1.000836	3.239393	-0.451873
H	-0.809076	-0.952309	-3.199668
C	-3.535214	2.145441	-0.982451
H	-4.049644	1.236035	-0.626425
C	-4.300506	2.679872	-2.197411
H	-4.356346	1.938361	-3.007749
H	-5.329395	2.945212	-1.913686

H	-3.815514	3.582881	-2.599114
C	-3.525657	3.159302	0.151236
H	-2.966706	2.796004	1.026637
H	-3.083615	4.118480	-0.160482
H	-4.554467	3.371146	0.475962
C	1.721323	0.163720	-2.780623
H	1.740224	-0.927807	-2.899479
H	1.933328	0.607267	-3.766526
H	2.530721	0.452644	-2.097096
O	-2.391526	-0.371959	0.813503
C	-2.711535	-0.859846	1.899048
O	-2.207807	-1.955921	2.383264
H	-1.737747	-2.473988	1.668017
C	-3.740223	-0.223521	2.772808
H	-4.617147	-0.885745	2.837702
H	-3.344852	-0.124581	3.794125
H	-4.043272	0.750954	2.375599
C	-1.186129	-2.717811	-0.750991
O	-1.780664	-2.859443	-1.788552
O	-1.563639	-3.414996	0.365760
C	-2.649761	-4.328975	0.203185
H	-3.558288	-3.799040	-0.115409
H	-2.395639	-5.080181	-0.555416
H	-2.797492	-4.805151	1.178924
H	0.550685	-1.897519	-1.449573

Transition state **TS4**

E(SMD/def2-QZVP*) = -1629.183552 E_h

ΔΔG(353) = 0.483402 E_h

Lowest frequency = -1167.57 cm^{-1}

C	-1.473983	-0.384325	1.679431
C	0.521533	-1.297852	2.404378
C	-0.033975	-1.705112	3.605076
C	-1.376622	-1.425573	3.849538
C	-2.097659	-0.752991	2.874992
Ru	0.827355	-0.306453	-0.394946
C	0.593068	-2.371473	-1.044670
C	-0.520767	-1.610882	-1.519410
C	-0.335113	-0.458957	-2.308204
C	1.014311	-0.029322	-2.548483
C	2.107385	-0.767551	-2.074210
C	1.917851	-1.980743	-1.329330
H	-1.536464	-1.903092	-1.241827
H	3.113678	-0.362601	-2.196582
H	-3.147538	-0.491638	3.013364
H	-1.851230	-1.715979	4.789229
H	0.585066	-2.230574	4.333358
H	1.569897	-1.487547	2.174756
N	-0.176163	-0.657271	1.447513
C	-3.438173	-0.261444	0.221568

C	-2.166086	1.617332	0.356339
C	-4.118018	0.781688	-0.450395
C	-3.944592	-1.560784	0.296037
C	-3.293316	1.952868	-0.339567
C	-5.343350	0.502906	-1.073936
C	-5.163574	-1.806729	-0.327402
H	-3.413724	-2.355910	0.824805
H	-3.503689	2.940486	-0.745170
C	-5.853513	-0.786870	-1.005385
H	-5.886028	1.289124	-1.603249
H	-5.591601	-2.810605	-0.288233
H	-6.807734	-1.016098	-1.484062
N	-2.234079	0.256207	0.694287
C	0.215077	1.981122	-0.217772
C	-0.962224	2.434227	0.655939
H	-1.198946	3.485633	0.445526
H	-0.696429	2.389140	1.725737
H	0.411621	-3.229974	-0.397193
H	1.202366	0.929811	-3.035705
C	3.131825	-2.723321	-0.831199
H	3.797688	-1.951981	-0.409123
C	3.836882	-3.372142	-2.026756
H	4.102670	-2.636304	-2.799913
H	4.765250	-3.861115	-1.697878
H	3.197968	-4.137825	-2.493460

C	2.838474	-3.744981	0.256479
H	2.317120	-3.298334	1.115732
H	2.228527	-4.581342	-0.119918
H	3.779230	-4.175378	0.627762
C	-1.494158	0.294417	-2.871856
H	-1.313130	1.378150	-2.897647
H	-1.650036	-0.032117	-3.912755
H	-2.417840	0.104343	-2.311207
O	2.566385	-0.079505	0.731899
C	2.688133	0.854426	1.580531
O	1.751111	1.657817	1.804078
H	0.984911	1.491122	0.777702
C	3.987382	1.000980	2.300851
H	4.533927	0.051334	2.329413
H	4.596022	1.734887	1.749176
H	3.820596	1.395204	3.310779
C	1.261942	2.997959	-0.584207
O	2.192594	2.744089	-1.313153
O	1.064113	4.181734	-0.021864
C	2.039452	5.181764	-0.289789
H	3.024999	4.862454	0.077951
H	2.111513	5.374453	-1.369287
H	1.708061	6.081414	0.239683
H	-0.191418	1.677319	-1.185960

Intermediate **G**

E(SMD/def2-QZVP*) = -1629.201819 E_h

ΔΔG(353) = 0.485205 E_h

Lowest frequency = 17.72 cm^{-1}

C	-1.395208	0.004595	1.727009
C	0.574829	-0.900521	2.530167
C	0.076849	-0.974707	3.820490
C	-1.217008	-0.524586	4.066953
C	-1.956473	-0.024716	3.003935
Ru	0.774241	-0.462528	-0.420630
C	0.369805	-2.535618	-0.739099
C	-0.699737	-1.811113	-1.348811
C	-0.434702	-0.798851	-2.288327
C	0.940092	-0.507008	-2.590727
C	1.988451	-1.245392	-2.025882
C	1.724550	-2.291921	-1.080999
H	-1.730036	-1.991191	-1.033603
H	3.018719	-0.934300	-2.207667
H	-2.971660	0.352721	3.133471
H	-1.640964	-0.551935	5.072989
H	0.707178	-1.371902	4.617076
H	1.592010	-1.211160	2.291444
N	-0.145238	-0.434267	1.495603
C	-3.373130	-0.137324	0.297724
C	-2.148488	1.765694	0.110236
C	-4.091256	0.769747	-0.516733
C	-3.852494	-1.412585	0.605635
C	-3.294775	1.961493	-0.609025
C	-5.323243	0.369033	-1.056127
C	-5.078365	-1.780178	0.060974
H	-3.295944	-2.094151	1.253373
H	-3.535884	2.866538	-1.163209
C	-5.802542	-0.900757	-0.763295
H	-5.895842	1.047425	-1.692334
H	-5.486958	-2.768835	0.280502
H	-6.761366	-1.223650	-1.173866
N	-2.173432	0.471040	0.654214
C	0.203967	2.113712	-0.576128
C	-0.961187	2.651503	0.246137
H	-1.230746	3.652530	-0.115531
H	-0.638223	2.766759	1.293034
H	0.139475	-3.251719	0.050615
H	1.182630	0.355743	-3.215820
C	2.887741	-3.009054	-0.444241
H	3.577177	-2.211222	-0.119214
C	3.587448	-3.860560	-1.508327
H	3.914143	-3.261911	-2.371228
H	4.479193	-4.339620	-1.079161
H	2.922290	-4.655735	-1.879359
C	2.516133	-3.845137	0.770783

H	2.000287	-3.254771	1.541905
H	1.872540	-4.697370	0.500757
H	3.424988	-4.260580	1.228274
C	-1.540785	-0.045450	-2.949165
H	-1.263473	0.994789	-3.167751
H	-1.762466	-0.530156	-3.914004
H	-2.457229	-0.050203	-2.346110
O	2.516952	-0.076903	0.565696
C	2.747367	0.966851	1.316461
O	1.886879	1.761898	1.665726
H	0.717637	1.366932	0.108049
C	4.193828	1.127749	1.695654
H	4.714764	1.590235	0.842562
H	4.284543	1.789039	2.565280
H	4.670012	0.157787	1.888808
C	1.385940	3.016461	-0.854712
O	2.352154	2.633741	-1.464985
O	1.226417	4.232321	-0.367705
C	2.324725	5.126059	-0.520935
H	3.188235	4.757934	0.050902
H	2.606273	5.215542	-1.579061
H	1.990285	6.090961	-0.125926
H	-0.140686	1.736341	-1.543803

Intermediate **H**

E(SMD/def2-QZVP*) = -1629.232680 E_h

ΔΔG(353) = 0.484414 E_h

Lowest frequency = 15.80 cm^{-1}

C	-0.357013	0.878589	1.824906
C	-0.614787	-1.321201	2.493316
C	-0.801700	-0.949332	3.816870
C	-0.772404	0.401711	4.146345
C	-0.536830	1.323259	3.133369
Ru	-0.462177	-1.233126	-0.490180
C	-2.291908	-0.146200	-0.986531
C	-1.259654	0.270398	-1.855581
C	-0.502870	-0.689998	-2.588344
C	-0.833870	-2.064661	-2.445033
C	-1.850229	-2.458843	-1.535363
C	-2.617350	-1.514756	-0.794288
H	-0.988007	1.326425	-1.899276
H	-1.986543	-3.524119	-1.330157
H	-0.475082	2.394465	3.329340
H	-0.913933	0.733905	5.177022
H	-0.968097	-1.719753	4.570923
H	-0.618188	-2.370358	2.195862
N	-0.417280	-0.430653	1.502990
C	-1.099157	2.684887	0.320213
C	1.084682	2.068493	0.170745
C	-0.492430	3.451531	-0.705280

C	-2.442270	2.830705	0.675294
C	0.883302	3.042172	-0.773715
C	-1.275684	4.383599	-1.404598
C	-3.190250	3.764976	-0.033187
H	-2.887694	2.249131	1.486093
H	1.654491	3.470322	-1.410340
C	-2.613911	4.528279	-1.064324
H	-0.835478	4.989595	-2.199575
H	-4.241064	3.915559	0.223236
H	-3.230533	5.254677	-1.597531
N	-0.131701	1.819975	0.812302
C	3.443030	1.366103	-0.363279
C	2.338906	1.443427	0.671780
H	2.691433	2.018567	1.547203
H	2.144948	0.428718	1.039113
H	-2.774621	0.616285	-0.374310
H	-0.222898	-2.824696	-2.935082
C	-3.704537	-1.986667	0.143475
H	-3.313883	-2.890830	0.642860
C	-4.931927	-2.392266	-0.676808
H	-4.689807	-3.165605	-1.420772
H	-5.718501	-2.791371	-0.019863
H	-5.346970	-1.525256	-1.214138
C	-4.072162	-0.971026	1.217431
H	-3.196453	-0.643793	1.796999
H	-4.552965	-0.079589	0.784913
H	-4.791586	-1.413630	1.920758
C	0.665640	-0.257494	-3.408796
H	1.436157	-1.037827	-3.452439
H	0.329926	-0.043689	-4.436252
H	1.108537	0.658758	-2.995897
O	0.523189	-2.876646	0.399525
C	1.667630	-2.375319	0.164100
O	1.658564	-1.269227	-0.455728
H	3.068479	0.879338	-1.278964
C	2.928988	-3.034044	0.580123
H	3.591489	-2.287955	1.043614
H	2.724720	-3.872395	1.255635
H	3.441993	-3.410791	-0.318278
C	4.599096	0.530019	0.118685
O	4.544853	-0.264553	1.026347
O	5.693418	0.751208	-0.599794
C	6.840654	-0.016505	-0.261467
H	7.121754	0.147589	0.788426
H	6.648521	-1.089216	-0.409584
H	7.641887	0.320111	-0.928197
H	3.820505	2.354466	-0.663617

Intermediate **A**$^{\mathbf{benzene}}$

E(SMD/def2-QZVP*) = -1165.763414 E_h

$\Delta\Delta G(353) = 0.293233\ E_h$

Lowest frequency = 25.71 cm^{-1}

C	0.657195	1.686180	0.107044
C	-1.349097	2.282338	-0.894029
C	-0.901843	3.570292	-1.140928
C	0.387532	3.919379	-0.744510
C	1.168899	2.968055	-0.103838
Ru	-1.324716	-0.642325	-0.277173
C	0.284985	-1.312549	-1.595849
C	0.342209	-2.060555	-0.401883
C	-0.833091	-2.715887	0.063934
C	-2.038058	-2.657193	-0.671331
C	-2.081166	-1.871996	-1.857916
C	-0.923949	-1.209659	-2.336667
H	1.250906	-2.060111	0.202582
H	-3.038827	-1.707417	-2.357042
H	2.171064	3.196043	0.261543
H	0.772759	4.927369	-0.913169
H	-1.562274	4.283360	-1.636004
H	-2.356154	1.966922	-1.170619
N	-0.581226	1.345656	-0.304080
C	2.703185	0.318638	0.294798
C	1.171711	0.093133	1.947593
C	3.206166	-0.602487	1.246407
C	3.398064	0.645253	-0.873338
C	2.217688	-0.714440	2.286330
C	4.453806	-1.203649	1.016479
C	4.629659	0.032474	-1.072511
H	3.002705	1.358284	-1.600428
H	2.286276	-1.314453	3.191189
C	5.151888	-0.879829	-0.137928
H	4.865674	-1.914283	1.736283
H	5.204319	0.267991	-1.970749
H	6.124493	-1.338269	-0.327515
N	1.447880	0.725103	0.737007
O	-3.163107	0.291719	0.123453
C	-2.932690	0.313960	1.373089
O	-1.850548	-0.233876	1.737604
C	-3.850377	0.977224	2.332783
H	-3.803458	0.480929	3.310070
H	-3.526053	2.021713	2.464658
H	-4.875753	0.980543	1.943474
H	0.232709	0.287484	2.460408
H	1.161020	-0.731994	-1.895125
H	-2.949824	-3.107765	-0.276842
H	-0.982535	-0.569311	-3.217889
H	-0.823153	-3.194866	1.045499

Transition state **TS1**$^{\mathbf{benzene}}$

E(SMD/def2-QZVP*) = -1165.743738 E_h

$\Delta\Delta G(353) = 0.291709\ E_h$

Lowest frequency = -114.98 cm^{-1}

C	0.782371	1.801518	0.333130
C	-1.214413	2.601578	-0.541979
C	-0.739090	3.904568	-0.535573
C	0.559418	4.142881	-0.083843
C	1.330273	3.081660	0.376559
Ru	-1.062850	-0.437224	-0.405677
C	0.015161	-0.611034	-2.289521
C	0.381561	-1.699232	-1.481350
C	-0.634672	-2.507169	-0.881023
C	-1.998711	-2.263263	-1.126657
C	-2.365783	-1.141796	-1.927142
C	-1.366671	-0.311115	-2.500125
H	1.430373	-1.862236	-1.227486
H	-3.416152	-0.856735	-2.011660
H	2.321132	3.233107	0.805418
H	0.959132	5.158940	-0.057840
H	-1.382526	4.718041	-0.872700
H	-2.229033	2.350939	-0.856832
N	-0.450354	1.571450	-0.153619
C	2.681442	0.182578	0.520819
C	0.684258	-0.337650	1.455077
C	2.755030	-1.143982	1.030631
C	3.733453	0.759860	-0.197362
C	1.490957	-1.440318	1.624703
C	3.937414	-1.886883	0.852097
C	4.881320	-0.002519	-0.361176
H	3.667820	1.766749	-0.612018
H	1.202827	-2.344522	2.157715
C	4.987779	-1.307524	0.162630
H	4.018286	-2.900441	1.250468
H	5.726438	0.421692	-0.907638
H	5.914562	-1.865511	0.016145
N	1.422079	0.669860	0.821843
O	-2.717690	0.300686	0.569518
C	-2.880080	-0.368240	1.667111
O	-2.079887	-1.244339	1.996619
C	-4.091507	-0.015123	2.478367
H	-4.047705	-0.507768	3.456170
H	-4.168868	1.074105	2.599182
H	-4.994630	-0.347276	1.944512
H	-0.249055	-0.118308	1.979048
H	0.784124	0.065321	-2.669161
H	-2.760395	-2.838292	-0.599024
H	-1.656582	0.585325	-3.052310
H	-0.352868	-3.267967	-0.151096

Intermediate **B**$^{\mathbf{benzene}}$

E(SMD/def2-QZVP*) = -1165.748628 E_h

ΔΔG(353) = 0.291483 E_h

Lowest frequency = 27.63 cm^{-1}

C	-0.872928	1.840711	-0.482751
C	0.992469	2.805054	0.497802
C	0.454689	4.076939	0.349050
C	-0.807215	4.210067	-0.228439
C	-1.481642	3.075839	-0.671898
Ru	0.950544	-0.275492	0.464739
C	-0.245782	-1.583167	1.740486
C	0.816012	-2.341553	1.148879
C	2.146961	-1.909932	1.267026
C	2.441249	-0.698994	1.965428
C	1.391577	0.049176	2.545131
C	0.039182	-0.406114	2.451831
H	0.591067	-3.208186	0.526630
H	1.609152	1.010222	3.016416
H	-2.438547	3.140048	-1.190693
H	-1.252845	5.197882	-0.363792
H	1.024056	4.947723	0.676335
H	1.983091	2.641710	0.925920
N	0.321534	1.710320	0.123535
C	-2.576312	0.013254	-0.607758
C	-0.466530	-0.356930	-1.355652
C	-2.446270	-1.375844	-0.908330
C	-3.735889	0.532514	-0.020101
C	-1.130132	-1.579245	-1.394473
C	-3.528918	-2.247370	-0.663702
C	-4.776479	-0.353199	0.210551
H	-3.828834	1.587896	0.239208
H	-0.686145	-2.496012	-1.780082
C	-4.682551	-1.726273	-0.113368
H	-3.449327	-3.308743	-0.907961
H	-5.701835	0.022281	0.653344
H	-5.537196	-2.377189	0.079811
N	-1.388440	0.625627	-0.931819
O	2.362415	0.237924	-0.912584
C	2.755382	-0.700451	-1.735812
O	2.246433	-1.809750	-1.786420
C	3.906237	-0.287998	-2.616130
H	4.023699	-1.003446	-3.438112
H	3.757894	0.728503	-3.004393
H	4.830050	-0.276308	-2.017610
H	0.359443	-0.060576	-2.003663
H	-1.283854	-1.881977	1.581431
H	3.459430	-0.306671	1.974322
H	2.930080	-2.436442	0.720815
H	-0.774405	0.205017	2.846955

Intermediate **C**$^{\mathbf{benzene}}$

E(SMD/def2-QZVP*) = -1165.767420 E_h

ΔΔG(353) = 0.292779 E_h

Lowest frequency = 27.29 cm^{-1}

C	0.805244	1.623359	-0.289833
C	-1.281538	2.665136	-0.214792
C	-0.715260	3.924487	-0.153893
C	0.678737	4.016730	-0.154973
C	1.447692	2.866757	-0.216699
Ru	-1.374813	-0.358401	-0.285413
C	-1.605765	-1.247295	-2.227323
C	-1.781468	-2.268322	-1.245290
C	-2.723223	-2.092570	-0.214320
C	-3.579290	-0.941849	-0.204382
C	-3.441557	0.046871	-1.178688
C	-2.399513	-0.079354	-2.154448
H	-1.127469	-3.141533	-1.244921
H	-2.795598	-2.830158	0.586547
H	-4.067506	0.940595	-1.150837
H	-2.212038	0.736925	-2.855472
H	2.531069	2.925357	-0.190635
H	1.169416	4.990708	-0.097327
H	-1.350708	4.808406	-0.097520
H	-2.363008	2.524964	-0.193142
N	-0.546771	1.544475	-0.292586
C	2.771541	0.019594	-0.356631
C	0.596594	-0.741408	-0.268046
C	2.787417	-1.394918	-0.268962
C	3.957511	0.747962	-0.464741
C	1.416197	-1.841857	-0.215338
C	4.004933	-2.083148	-0.264773
C	5.156916	0.040488	-0.459809
H	3.985519	1.830136	-0.571305
H	1.091816	-2.879922	-0.160527
C	5.184994	-1.356568	-0.356260
H	4.022381	-3.172876	-0.196083
H	6.094993	0.592760	-0.544685
H	6.145139	-1.876290	-0.355393
N	1.422588	0.400701	-0.335629
O	-1.372909	-0.045994	1.814468
C	-0.636358	-0.391194	2.746253
O	0.457129	-1.070868	2.595680
H	0.650864	-1.236101	1.631205
C	-0.962554	-0.034737	4.154356
H	-0.956610	-0.942936	4.773997
H	-0.173908	0.624614	4.547231
H	-1.934196	0.465812	4.210879
H	-4.288991	-0.806293	0.614087
H	-0.821387	-1.334835	-2.980050

Intermediate $\mathbf{D}^{\mathbf{benzene}}$

E(SMD/def2-QZVP*) = -1243.089690 E_h

ΔΔG(353) = 0.325409 E_h

Lowest frequency = 13.38 cm^{-1}

C	-0.724791	1.230779	0.841234
C	1.519093	1.653520	1.344714
C	1.221608	2.875048	1.913249
C	-0.116801	3.284307	1.927458
C	-1.093278	2.469343	1.388808
Ru	1.004758	-1.003284	0.016392
C	0.494740	-2.953742	1.026249
C	1.214125	-3.216598	-0.160178
C	2.481114	-2.604601	-0.352710
C	3.083173	-1.823638	0.672798
C	2.326164	-1.515456	1.809269
C	1.013950	-2.054682	1.981612
H	0.787558	-3.854699	-0.935613
H	2.729145	-0.839763	2.567096
H	-2.131635	2.783160	1.377842
H	-0.395888	4.249256	2.355975
H	2.018919	3.494412	2.324004
H	2.547404	1.300125	1.264938
N	0.574699	0.844872	0.830888
C	-2.957437	0.276827	0.109658
C	-0.989771	-0.856477	-0.240065
C	-3.237291	-0.944399	-0.552264
C	-3.977433	1.161843	0.457359
C	-1.984132	-1.630338	-0.758731
C	-4.556974	-1.274775	-0.874704
C	-5.284247	0.810481	0.125818
H	-3.802616	2.103914	0.972050
H	-1.859281	-2.585823	-1.264935
C	-5.573376	-0.390629	-0.533217
H	-4.781042	-2.212610	-1.387441
H	-6.096408	1.491475	0.387770
H	-6.609165	-0.632654	-0.779694
N	-1.565081	0.313186	0.291675
C	1.834394	0.126941	-1.683340
H	2.898129	-0.115064	-1.759657
C	0.851321	-0.746255	-2.157531
H	1.123266	-1.691001	-2.633927
H	-0.112779	-0.327126	-2.449618
C	1.531374	1.587809	-1.664637
O	0.440591	2.069041	-1.829907
O	2.638915	2.297503	-1.450265
H	-0.509731	-3.360567	1.155912
H	4.082139	-1.405402	0.536238
C	2.488097	3.712016	-1.482735
H	3.488157	4.130771	-1.327344
H	2.083908	4.033577	-2.452653
H	1.801936	4.043273	-0.689603
H	0.424041	-1.791417	2.860874

H	3.019472	-2.772060	-1.288694

Transition state **TS3^{benzene}**

E(SMD/def2-QZVP*) = -1243.071195 E_h

ΔΔG(353) = 0.327083 E_h

Lowest frequency = -293.46 cm^{-1}

C	-0.704984	1.431119	0.583578
C	1.463492	1.656745	1.396683
C	1.233585	2.956224	1.804383
C	-0.019410	3.516416	1.545574
C	-0.994871	2.757511	0.923031
Ru	0.842363	-1.033669	0.131174
C	0.097712	-2.638989	1.517153
C	0.441376	-3.176944	0.242586
C	1.735616	-2.991539	-0.294736
C	2.702207	-2.233134	0.429195
C	2.345169	-1.641046	1.652211
C	1.034632	-1.843161	2.192526
H	-0.307786	-3.723629	-0.332496
H	3.061927	-1.010645	2.181075
H	-1.957125	3.189484	0.661528
H	-0.228566	4.554531	1.811920
H	2.027682	3.524805	2.288725
H	2.440991	1.188200	1.515579
N	0.518545	0.903763	0.809370
C	-2.974251	0.546031	-0.081126
C	-1.066152	-0.625900	-0.580027
C	-3.337793	-0.669002	-0.720389
C	-3.941679	1.431660	0.396522
C	-2.132319	-1.377921	-1.021173
C	-4.692208	-0.972569	-0.923045
C	-5.277674	1.102678	0.187281
H	-3.693487	2.346896	0.930314
H	-2.063904	-2.318019	-1.565510
C	-5.651688	-0.078825	-0.472309
H	-4.980521	-1.900870	-1.420609
H	-6.051208	1.783487	0.548394
H	-6.710899	-0.296998	-0.621848
N	-1.579356	0.563222	-0.019647
C	1.607987	-0.159162	-1.660736
H	2.336429	-0.870064	-2.059321
C	0.226846	-0.324166	-2.005691
H	-0.035104	-1.165966	-2.649153
H	-0.298867	0.607120	-2.243985
C	2.121139	1.229060	-1.550858
O	1.444384	2.225250	-1.626910
O	3.441050	1.243808	-1.348548
H	-0.914170	-2.755354	1.908227
H	3.689142	-2.046508	0.001501
C	4.049508	2.528119	-1.299256

H	5.121351	2.355338	-1.153633
H	3.875047	3.075015	-2.236554
H	3.638707	3.122275	-0.469776
H	0.750694	-1.338953	3.118577
H	1.980459	-3.399286	-1.277275

Intermediate **E**[benzene]

E(SMD/def2-QZVP*) = -1243.095101 E_h

ΔΔG(353) = 0.329739 E_h

Lowest frequency = 30.38 cm^{-1}

C	-0.610975	2.017348	-0.138733
C	1.208184	1.926952	1.282994
C	1.100824	3.282278	1.562107
C	0.091364	4.019813	0.950716
C	-0.773316	3.378567	0.067728
Ru	0.350065	-0.822535	0.141856
C	-0.835138	-1.714481	1.868602
C	-0.750909	-2.622933	0.787503
C	0.498066	-3.004441	0.223884
C	1.690722	-2.425928	0.725042
C	1.629021	-1.479813	1.777575
C	0.360825	-1.116862	2.321372
H	-1.673502	-3.018169	0.357840
H	2.543734	-1.003110	2.132916
H	-1.546497	3.915268	-0.482520
H	-0.006147	5.091468	1.136263
H	1.819032	3.750707	2.235873
H	2.010281	1.328594	1.713917
N	0.349437	1.290904	0.475141
C	-2.643841	0.733696	-0.576864
C	-0.690175	0.219195	-1.628343
C	-2.713803	-0.621151	-0.961496
C	-3.662482	1.350160	0.140784
C	-1.447222	-0.944879	-1.588034
C	-3.862793	-1.367444	-0.681162
C	-4.791871	0.585635	0.427393
H	-3.593542	2.392405	0.456446
H	-1.225507	-1.845450	-2.159661
C	-4.898291	-0.750764	0.013064
H	-3.949095	-2.407820	-1.003956
H	-5.618727	1.043079	0.974804
H	-5.808599	-1.309986	0.237686
N	-1.408425	1.254639	-1.005955
C	1.602746	-0.401911	-1.521774
H	1.849981	-1.358549	-1.998804
C	0.628557	0.451644	-2.313651
H	0.567019	0.191075	-3.381362
H	0.910971	1.511231	-2.244552
C	2.831855	0.284786	-1.064165
O	2.975979	1.482113	-0.964394

O	3.794409	-0.587641	-0.717456
H	-1.803437	-1.419639	2.274521
H	2.651227	-2.635641	0.251079
C	5.016952	-0.021064	-0.269505
H	5.699588	-0.859755	-0.092029
H	5.434944	0.655372	-1.028141
H	4.870445	0.552214	0.658617
H	0.311571	-0.329318	3.077429
H	0.533424	-3.697452	-0.617760

Intermediate **F**$^{\mathbf{benzene}}$

E(SMD/def2-QZVP*) = -1472.052792 E_h

ΔΔG(353) = 0.384520 E_h

Lowest frequency = 30.36 cm^{-1}

C	-1.308306	1.144586	-1.181972
C	0.416246	2.624197	-0.746964
C	-0.148592	3.607891	-1.538659
C	-1.350901	3.331863	-2.189779
C	-1.930039	2.086376	-2.010166
Ru	0.694338	0.208434	1.004912
C	-0.296231	1.456796	2.621219
C	-1.134648	0.400649	2.140252
C	-0.702789	-0.943763	2.167504
C	0.623745	-1.240251	2.612521
C	1.482719	-0.192233	2.987264
C	1.009191	1.160495	3.020865
H	-2.123399	0.628315	1.735440
H	2.530349	-0.409730	3.200740
H	-2.857226	1.806148	-2.511301
H	-1.822720	4.073396	-2.837979
H	0.349970	4.572798	-1.638170
H	1.344627	2.808771	-0.205513
N	-0.139649	1.408972	-0.559538
C	-3.252654	-0.249744	-0.584645
C	-1.356308	-1.338699	-1.212075
C	-3.513153	-1.640840	-0.571628
C	-4.206242	0.692931	-0.194400
C	-2.302023	-2.298160	-0.982279
C	-4.780251	-2.092560	-0.176076
C	-5.453058	0.215039	0.196524
H	-3.995080	1.764373	-0.192659
H	-2.150534	-3.368214	-1.110048
C	-5.737983	-1.160628	0.200997
H	-5.006203	-3.160945	-0.161538
H	-6.223935	0.924720	0.503936
H	-6.728851	-1.499169	0.510373
N	-1.921840	-0.083583	-0.969168
C	0.941959	-1.470395	-0.311461
C	0.076321	-1.482047	-1.575105
H	0.207578	-2.431552	-2.114309

H	0.363879	-0.700496	-2.303094
H	-0.641381	2.491696	2.584064
H	1.012748	-2.258681	2.567259
O	2.613591	0.978121	0.477699
C	3.293038	0.976963	-0.546910
O	2.801262	0.663434	-1.720001
H	1.877782	0.368499	-1.612142
C	4.738806	1.305535	-0.535457
H	5.270341	0.368078	-0.301750
H	5.076984	1.670519	-1.512213
H	4.958140	2.024209	0.262420
C	2.372325	-1.873538	-0.516469
O	3.220919	-1.874778	0.346828
O	2.624269	-2.256312	-1.772964
C	3.952255	-2.663634	-2.065813
H	4.300406	-3.413823	-1.342976
H	3.929988	-3.082626	-3.077857
H	4.633216	-1.799630	-2.036013
H	0.552338	-2.287940	0.315931
H	-1.359116	-1.728311	1.785444
H	1.698753	1.966112	3.280551

Transition state **TS4**$^{\mathbf{benzene}}$

E(SMD/def2-QZVP*) = -1472.030998 E_h

$\Delta\Delta G(353)$ = 0.379853 E_h

Lowest frequency = -1117.34 cm^{-1}

C	1.253463	-0.997443	-1.341474
C	-0.275499	-2.686942	-0.939548
C	0.277346	-3.494052	-1.920186
C	1.361537	-3.014359	-2.650645
C	1.849863	-1.749589	-2.355218
Ru	-0.686616	-0.444781	0.998428
C	0.326119	-1.668697	2.491851
C	1.179825	-0.583422	2.120589
C	0.719163	0.743103	2.202649
C	-0.619136	0.996270	2.640973
C	-1.471958	-0.062693	2.996612
C	-0.989118	-1.404112	2.919526
H	2.174306	-0.777560	1.712243
H	-2.518341	0.136207	3.230811
H	2.691555	-1.314690	-2.895688
H	1.816172	-3.613173	-3.442718
H	-0.145011	-4.482910	-2.103264
H	-1.128331	-3.020187	-0.347340
N	0.195825	-1.461467	-0.646111
C	3.110163	0.390804	-0.537021
C	1.245323	1.504595	-1.206051
C	3.356363	1.779095	-0.412687
C	4.056401	-0.574543	-0.184233
C	2.164974	2.453344	-0.849116

C	4.598624	2.206257	0.080649
C	5.277205	-0.120316	0.303807
H	3.858116	-1.643439	-0.292778
H	2.007367	3.528499	-0.909592
C	5.544483	1.253701	0.434265
H	4.815730	3.271643	0.183833
H	6.043441	-0.845559	0.585604
H	6.515281	1.573468	0.818224
N	1.806519	0.240434	-1.002112
C	-1.081305	1.424705	-0.413635
C	-0.171812	1.669982	-1.621612
H	-0.313273	2.695040	-1.990091
H	-0.433645	1.000491	-2.457488
H	0.662891	-2.699739	2.369502
H	-1.024310	2.010190	2.617260
O	-2.460897	-1.383850	0.477437
C	-3.007448	-1.169852	-0.649398
O	-2.464776	-0.434282	-1.506932
H	-1.614302	0.251481	-0.783053
C	-4.333830	-1.795522	-0.923814
H	-5.108589	-1.070144	-0.628262
H	-4.447981	-1.995025	-1.996146
H	-4.473389	-2.706256	-0.330106
C	-2.447439	2.062158	-0.392555
O	-3.200715	1.942996	0.544087
O	-2.720425	2.749770	-1.490051
C	-4.007961	3.353321	-1.556355
H	-4.154665	4.046464	-0.716484
H	-4.044291	3.890822	-2.509798
H	-4.791440	2.583093	-1.521059
H	-0.590658	1.883203	0.448849
H	1.358833	1.557099	1.853103
H	-1.678313	-2.233239	3.090591

Intermediate **G**$^{\mathbf{benzene}}$

E(SMD/def2-QZVP*) = -1472.048548 E_h

ΔΔG(353) = 0.382223 E_h

Lowest frequency = 23.86 cm^{-1}

C	-1.094181	-0.339352	1.674458
C	0.387513	-2.113315	1.811824
C	-0.005841	-2.405413	3.108478
C	-0.974341	-1.609571	3.711852
C	-1.521842	-0.561888	2.981647
Ru	0.543946	-0.822428	-0.887678
C	-0.506447	-2.478227	-1.726790
C	-1.413300	-1.371757	-1.694732
C	-1.047502	-0.146015	-2.274450
C	0.232744	-0.021996	-2.894252
C	1.122551	-1.112095	-2.972674
C	0.746849	-2.345754	-2.372486

H	-2.353505	-1.442967	-1.143583
H	2.126645	-0.975832	-3.375844
H	-2.283512	0.098232	3.398486
H	-1.297260	-1.797456	4.738044
H	0.456152	-3.242476	3.633346
H	1.160474	-2.691485	1.304147
N	-0.145405	-1.106043	1.101852
C	-3.038442	0.583254	0.501515
C	-1.204260	1.920157	0.570131
C	-3.354628	1.798201	-0.152552
C	-3.956778	-0.462172	0.631828
C	-2.180396	2.622006	-0.086260
C	-4.636851	1.959239	-0.701595
C	-5.217406	-0.273170	0.076395
H	-3.705677	-1.386335	1.157884
H	-2.072341	3.637051	-0.463927
C	-5.551933	0.922639	-0.584602
H	-4.908529	2.887248	-1.209393
H	-5.963970	-1.065548	0.162112
H	-6.552898	1.036143	-1.005628
N	-1.712840	0.663096	0.912730
C	1.129218	1.674699	-0.204959
C	0.209964	2.298360	0.835873
H	0.297753	3.392121	0.787195
H	0.538743	1.996610	1.842327
H	-0.750859	-3.403273	-1.201166
H	0.566469	0.953812	-3.254935
O	2.345363	-1.475792	-0.225840
C	3.034339	-0.924055	0.740944
O	2.586838	-0.075085	1.497892
H	1.295879	0.603958	0.139536
C	4.455375	-1.404722	0.815929
H	5.038862	-0.812295	0.093348
H	4.859289	-1.230197	1.820180
H	4.543095	-2.460914	0.532120
C	2.580167	2.106901	-0.263566
O	3.350214	1.649517	-1.068328
O	2.873388	3.013645	0.648465
C	4.238958	3.412337	0.729806
H	4.853616	2.563381	1.061033
H	4.599686	3.759884	-0.247769
H	4.277426	4.220553	1.467488
H	0.719875	1.807114	-1.210947
H	-1.697918	0.724185	-2.158726
H	1.478987	-3.152063	-2.297665

Intermediate $\mathbf{H}^{\mathbf{benzene}}$

E(SMD/def2-QZVP*) = -1472.077144 E_h

ΔΔG(353) = 0.381587 E_h

Lowest frequency = 15.97 cm^{-1}

C	-0.794504	0.462369	1.773284	C	-4.475922	2.293862	0.077915
C	-0.419628	-1.722563	2.441071	H	-3.635580	0.976806	1.580661
C	-0.594274	-1.396081	3.778370	H	0.139800	3.513134	-1.553440
C	-0.883094	-0.078573	4.116777	C	-4.221312	3.182002	-0.982556
C	-0.977426	0.859685	3.095912	H	-2.737657	4.160558	-2.209559
Ru	-0.409404	-1.613604	-0.543313	H	-5.506201	2.114735	0.392587
C	-2.510892	-1.146346	-0.938434	H	-5.060942	3.671310	-1.480301
C	-1.708234	-0.442812	-1.864459	N	-0.922712	1.414850	0.753693
C	-0.742093	-1.159554	-2.620610	C	2.553090	2.111177	-0.588355
C	-0.599504	-2.560809	-2.481861	C	1.529293	1.848051	0.498049
C	-1.396125	-3.239923	-1.519616	H	1.716929	2.521757	1.353976
C	-2.369509	-2.545532	-0.756887	H	1.685853	0.831106	0.877060
H	-1.771374	0.644772	-1.929423	H	-3.182102	-0.576176	-0.293192
H	-1.195626	-4.292226	-1.305646	H	0.189033	-3.092313	-3.016313
H	-1.185830	1.911629	3.294990	O	1.079191	-2.839331	0.289935
H	-1.022517	0.218268	5.158487	C	1.989597	-1.992642	0.018758
H	-0.499872	-2.175155	4.536057	O	1.590873	-0.952419	-0.588271
H	-0.171152	-2.740274	2.138652	H	2.311961	1.514047	-1.483667
N	-0.529572	-0.821377	1.446931	C	3.410767	-2.203985	0.376970
C	-2.136607	1.923637	0.313480	H	3.828659	-1.270200	0.781851
C	0.120265	2.030595	0.055643	H	3.511951	-3.037442	1.081415
C	-1.853619	2.828065	-0.739954	H	3.971000	-2.440485	-0.541189
C	-3.437463	1.650515	0.743081	C	3.936587	1.699306	-0.158998
C	-0.424343	2.874591	-0.877336	O	4.177246	0.907796	0.721363
C	-2.924511	3.457983	-1.394402	O	4.872597	2.292931	-0.888176

C	6.219977	1.942280	-0.600682
H	6.463768	2.166574	0.447381
H	6.389278	0.870411	-0.778971
H	6.842293	2.541409	-1.274077
H	2.577899	3.162857	-0.908283
H	-0.038723	-0.607305	-3.247573
H	-2.943463	-3.067121	0.010147

Intermediate $\mathbf{A}^{t\text{-Bubenzene}}$

E(SMD/def2-QZVP*) = -1322.908834 E_h

ΔΔG(353) = 0.398323 E_h

Lowest frequency = 22.09 cm^{-1}

C	0.834918	-0.820055	1.569199
C	-1.296882	-0.317362	2.328037
C	-0.866836	-0.236203	3.642339
C	0.483098	-0.445030	3.918904
C	1.337645	-0.755881	2.871102
Ru	-1.223673	-0.278056	-0.665643
C	0.278332	1.231108	-1.161254
C	0.316478	0.212800	-2.132239
C	-0.884600	-0.168230	-2.796802
C	-2.093980	0.486281	-2.496873
C	-2.109795	1.491031	-1.485964
C	-0.932677	1.905811	-0.809048
H	1.243059	-0.337073	-2.304467
H	-3.074447	1.892867	-1.174224
H	2.392380	-0.979814	3.035424
H	0.860771	-0.394551	4.942453
H	-1.586013	-0.011732	4.430996
H	-2.345212	-0.175763	2.062108
N	-0.461741	-0.567534	1.300758
C	2.881864	-0.491137	0.222947
C	1.557474	-2.242271	-0.332325
C	3.489312	-1.187283	-0.850838
C	3.434549	0.669449	0.771898
C	2.632726	-2.299337	-1.169383
C	4.695915	-0.702841	-1.379129
C	4.629175	1.124971	0.225817
H	2.958593	1.199632	1.599395
H	2.806484	-3.062701	-1.924765
C	5.253108	0.446136	-0.836012
H	5.185931	-1.223386	-2.204757
H	5.093799	2.025103	0.633858
H	6.192732	0.832098	-1.236315
N	1.689516	-1.145551	0.517435
O	-2.994029	-1.082561	0.148315
C	-2.608269	-2.273565	-0.069457
O	-1.493669	-2.391203	-0.658087
C	-3.396565	-3.449226	0.380337
H	-3.179671	-4.320061	-0.250106

H	-3.107157	-3.691362	1.415265
H	-4.468638	-3.216362	0.371704
H	0.686586	-2.889058	-0.252838
H	1.194797	1.437865	-0.607794
H	-3.030573	0.148477	-2.942896
C	-0.955011	3.048297	0.201740
C	-0.783024	4.339036	-0.616882
H	-1.600105	4.465455	-1.343094
H	-0.785122	5.212913	0.051985
H	0.167905	4.338392	-1.171042
C	0.192053	2.940504	1.210418
H	0.124446	3.771774	1.926659
H	0.148363	2.003368	1.784208
H	1.179674	3.013028	0.732500
C	-2.282185	3.098715	0.963365
H	-3.135549	3.332113	0.310620
H	-2.491182	2.149206	1.477887
H	-2.240734	3.892563	1.722781
H	-0.883293	-1.031111	-3.465815

Transition state **TS1**$^{t\text{-Bubenzene}}$

E(SMD/def2-QZVP*) = -1322.888185 E_h

ΔΔG(353) = 0.397227 E_h

Lowest frequency = -120.90 cm^{-1}

C	0.689749	-0.721450	1.696297
C	-1.231163	0.312804	2.492654
C	-0.746898	0.311717	3.790747
C	0.514400	-0.234375	4.033204
C	1.237041	-0.776581	2.979519
Ru	-1.242738	-0.003224	-0.517048
C	0.189705	1.091330	-1.788001
C	-0.667947	0.273765	-2.572221
C	-2.075703	0.376659	-2.491641
C	-2.621659	1.261514	-1.532438
C	-1.772062	2.044909	-0.704799
C	-0.345771	2.009563	-0.851524
H	-0.228206	-0.503888	-3.198683
H	-2.224821	2.660437	0.071863
H	2.190579	-1.279690	3.137780
H	0.918827	-0.269148	5.047116
H	-1.356761	0.720248	4.597155
H	-2.224694	0.688576	2.244381
N	-0.509656	-0.156104	1.462902
C	2.636386	-1.165568	0.187368
C	0.568658	-1.849934	-0.445536
C	2.724945	-1.719121	-1.118391
C	3.745131	-0.600584	0.825080
C	1.412336	-2.153636	-1.485180
C	3.965714	-1.736223	-1.779753
C	4.953742	-0.625872	0.142831

H	3.679414	-0.149494	1.815534
H	1.125004	-2.684464	-2.390954
C	5.067285	-1.191962	-1.142112
H	4.053020	-2.170555	-2.777859
H	5.839574	-0.198808	0.617770
H	6.039809	-1.197835	-1.638089
N	1.310835	-1.268118	0.585989
O	-2.897428	-0.793022	0.426176
C	-3.087997	-2.001340	0.006916
O	-2.313550	-2.523717	-0.797101
C	-4.308586	-2.698933	0.530229
H	-4.210558	-3.781873	0.391933
H	-4.479571	-2.453578	1.586389
H	-5.185805	-2.351281	-0.037034
H	-0.449724	-2.239333	-0.282412
H	1.266925	0.942233	-1.860907
H	-3.695689	1.255380	-1.337057
C	0.553727	2.972613	-0.084343
C	0.852875	4.100766	-1.090763
H	-0.071972	4.574157	-1.453553
H	1.462135	4.877140	-0.604104
H	1.410148	3.726166	-1.962185
C	1.865815	2.326385	0.359521
H	2.514126	3.093480	0.806838
H	1.692513	1.555883	1.122062
H	2.431036	1.872453	-0.465676
H	-2.714466	-0.306840	-3.051105
C	-0.139128	3.569833	1.142057
H	-1.011458	4.184246	0.875839
H	-0.457142	2.793457	1.853380
H	0.564222	4.230574	1.668545

Intermediate **B**$^{\boldsymbol{t}\textbf{-Bubenzene}}$

E(SMD/def2-QZVP*) = -1322.893220 E_h

ΔΔG(353) = 0.395954 E_h

Lowest frequency = 20.33 cm^{-1}

C	0.640233	-0.657360	1.863584
C	-1.030843	0.768277	2.600602
C	-0.526076	0.753973	3.893378
C	0.616549	0.000074	4.158109
C	1.203161	-0.736093	3.134197
Ru	-1.126695	0.117366	-0.384505
C	0.091789	1.038218	-1.951129
C	-0.978042	0.295881	-2.542884
C	-2.309477	0.530334	-2.168197
C	-2.577304	1.499672	-1.158414
C	-1.516034	2.225263	-0.572005
C	-0.152183	2.034796	-0.982126
H	-0.756334	-0.519212	-3.232624
H	-1.744547	2.913697	0.241441

H	2.052960	-1.394323	3.315024
H	1.031559	-0.042038	5.167367
H	-1.030819	1.317287	4.679008
H	-1.936437	1.318229	2.340242
N	-0.433847	0.109216	1.601149
C	2.362716	-1.442293	0.233867
C	0.164584	-1.785011	-0.217425
C	2.253324	-1.953285	-1.093622
C	3.597651	-1.055448	0.770186
C	0.878520	-2.184513	-1.341332
C	3.415151	-2.117732	-1.878340
C	4.715164	-1.225026	-0.029771
H	3.688377	-0.637674	1.773074
H	0.426586	-2.646377	-2.217879
C	4.631622	-1.755232	-1.338267
H	3.344656	-2.524250	-2.889332
H	5.694420	-0.944201	0.364458
H	5.546033	-1.876411	-1.921891
N	1.094470	-1.376887	0.762317
O	-2.622853	-0.943368	0.530311
C	-3.156890	-1.940627	-0.123913
O	-2.720736	-2.364672	-1.184150
C	-4.370091	-2.525875	0.551721
H	-4.649297	-3.469541	0.069429
H	-4.180019	-2.680959	1.622434

H	-5.207434	-1.816247	0.470442
H	-0.787882	-2.231193	0.084312
H	1.117465	0.790555	-2.226649
H	-3.590402	1.617501	-0.770152
C	0.987542	2.890283	-0.432753
C	1.359640	3.869785	-1.559138
H	0.493270	4.474604	-1.866919
H	2.146879	4.557070	-1.214459
H	1.736129	3.339526	-2.446599
C	2.203369	2.038787	-0.064321
H	3.033655	2.692142	0.240754
H	1.974728	1.374264	0.779981
H	2.569037	1.420898	-0.896321
H	-3.102056	-0.114148	-2.548139
C	0.564295	3.689713	0.800967
H	0.232350	3.034826	1.620815
H	1.421944	4.269815	1.170705
H	-0.236106	4.410723	0.578319

Intermediate $\mathbf{C}^{t\text{-Bubenzene}}$

E(SMD/def2-QZVP*) = -1322.912188 E_h

ΔΔG(353) = 0.397145 E_h

Lowest frequency = 20.44 cm^{-1}

C	1.002512	-0.530456	1.587812
C	-0.971458	-1.351815	2.519016

C	-0.350308	-1.648899	3.717896	C	3.949513	1.285621	-1.877914
C	1.014314	-1.374600	3.834266	C	5.128572	1.087304	0.230619
C	1.701022	-0.819173	2.767342	H	4.049547	0.378575	1.939876
Ru	-1.217507	-0.427039	-0.349723	H	1.094367	0.772054	-2.733333
C	-2.053944	1.593226	-0.287275	C	5.112021	1.421039	-1.130156
C	-1.862718	1.183220	-1.642258	H	3.929873	1.555210	-2.935958
C	-2.412879	-0.017853	-2.145076	H	6.049021	1.212849	0.804393
C	-3.214216	-0.832928	-1.294246	H	6.021676	1.797294	-1.602375
C	-3.460387	-0.454736	0.033427	N	1.538771	0.010940	0.447296
C	-2.819958	0.714012	0.533412	O	-0.732466	-2.472975	-0.684348
C	-1.524418	2.912734	0.271982	C	0.160417	-3.062369	-1.304015
H	-1.212487	1.770736	-2.289476	O	1.146425	-2.467314	-1.899838
H	-2.200844	-0.330934	-3.168050	H	1.116565	-1.482075	-1.747573
H	-4.055949	-1.089051	0.691378	C	0.169101	-4.547352	-1.412259
H	-2.916780	0.945952	1.596373	H	0.221419	-4.836796	-2.471789
H	2.766801	-0.626640	2.837191	H	1.078484	-4.936300	-0.930162
H	1.548626	-1.603752	4.758759	H	-0.720970	-4.974084	-0.939489
H	-0.919746	-2.093214	4.534342	C	-0.761876	2.687295	1.581702
H	-2.028910	-1.564952	2.356699	H	-0.464872	3.656863	2.007113
N	-0.320321	-0.798483	1.484790	H	-1.364076	2.171457	2.344223
C	2.829218	0.452003	0.122686	H	0.151274	2.101619	1.405871
C	0.692712	0.110781	-0.677584	C	-2.748256	3.807252	0.526072
C	2.796525	0.802186	-1.250795	H	-2.424866	4.793090	0.892288
C	3.994179	0.602797	0.876908	H	-3.327842	3.962953	-0.396562
C	1.451642	0.577989	-1.722861	H	-3.421963	3.373997	1.281158

C	-0.590689	3.610869	-0.717584
H	-0.219051	4.544568	-0.271578
H	0.282227	2.987458	-0.962150
H	-1.106522	3.883931	-1.650705
H	-3.579362	-1.794866	-1.660469

Intermediate **D**$^{t\text{-Bubenzene}}$

E(SMD/def2-QZVP*) = -1400.235611 E_h

ΔΔG(353) = 0.430170 E_h

Lowest frequency = 16.98 cm^{-1}

C	-0.671920	1.094069	1.071594
C	1.577472	1.136848	1.707510
C	1.306452	1.895409	2.828092
C	-0.021790	2.268631	3.062644
C	-1.014612	1.876270	2.185248
Ru	1.001388	-0.474749	-0.767413
C	0.435500	-2.649376	-0.937905
C	1.209147	-2.282247	-2.057615
C	2.485476	-1.697006	-1.860205
C	3.025849	-1.581599	-0.552740
C	2.211991	-1.900867	0.541994
C	0.886220	-2.427386	0.389919
H	0.810612	-2.410517	-3.065165
H	2.593730	-1.720966	1.548047
H	-2.044870	2.175375	2.346081
H	-0.280482	2.876393	3.932328
H	2.115780	2.193168	3.494594
H	2.595730	0.842692	1.452059
N	0.616626	0.732676	0.858389
C	-2.922029	0.698361	-0.028140
C	-0.976678	-0.136666	-0.926302
C	-3.226240	-0.009439	-1.217933
C	-3.923437	1.300349	0.733062
C	-1.987342	-0.515165	-1.758101
C	-4.553322	-0.108100	-1.647185
C	-5.237862	1.187149	0.285115
H	-3.727933	1.848572	1.651901
H	-1.881164	-1.086114	-2.678602
C	-5.551577	0.493473	-0.890096
H	-4.797014	-0.648708	-2.564263
H	-6.035889	1.652975	0.866605
H	-6.592466	0.426850	-1.213152
N	-1.529300	0.612803	0.131427
C	1.900162	1.327583	-1.661983
H	2.961619	1.128793	-1.833727
C	0.916347	0.834177	-2.525027
H	1.188726	0.248690	-3.406199
H	-0.031868	1.370303	-2.587116
C	1.620227	2.591128	-0.921871
O	0.541792	3.119407	-0.836459

O	2.733648	3.071212	-0.366406
H	-0.571684	-3.032361	-1.101952
H	4.027995	-1.176924	-0.399967
C	0.068977	-2.903090	1.590129
C	0.450498	-4.379368	1.797934
H	1.525985	-4.494317	2.003200
H	-0.103372	-4.792583	2.654222
H	0.205943	-4.986145	0.912820
C	-1.436536	-2.794994	1.329544
H	-1.741245	-1.763570	1.103768
H	-1.773836	-3.438917	0.504880
H	-1.982390	-3.122390	2.225744
C	2.604154	4.314169	0.312938
H	3.607442	4.577608	0.664763
H	2.221022	5.087143	-0.367692
H	1.909907	4.218388	1.160657
C	0.399100	-2.114874	2.860585
H	1.448284	-2.224825	3.172381
H	0.174971	-1.044743	2.750088
H	-0.213384	-2.497310	3.689300
H	3.068623	-1.365560	-2.722467

Transition state **TS3**$^{t\text{-Bubenzene}}$

E(SMD/def2-QZVP*) = -1400.216070 E_h

ΔΔG(353) = 0.428204 E_h

Lowest frequency = -293.65 cm^{-1}

C	0.395343	-1.444878	0.940635
C	-1.667475	-0.848428	1.837984
C	-1.555037	-1.777428	2.853980
C	-0.422795	-2.595085	2.877244
C	0.556008	-2.440419	1.910907
Ru	-0.861871	0.695423	-0.636380
C	0.236342	2.589192	-0.953491
C	-0.750245	2.458577	-1.968916
C	-2.097176	2.245083	-1.609776
C	-2.469747	2.187568	-0.240450
C	-1.459885	2.296958	0.740633
C	-0.084968	2.537345	0.424203
H	-0.458354	2.476564	-3.020273
H	-1.740839	2.156938	1.785184
H	1.416086	-3.103632	1.875349
H	-0.314060	-3.369935	3.638909
H	-2.349123	-1.875610	3.594372
H	-2.554080	-0.221506	1.733744
N	-0.712403	-0.672489	0.910358
C	2.659876	-1.414974	-0.175124
C	0.867683	-0.345667	-1.129853
C	3.093902	-0.761967	-1.359538
C	3.559792	-2.082980	0.656670
C	1.959142	-0.108727	-1.935057

C	4.444424	-0.823378	-1.733441
C	4.894631	-2.122966	0.264502
H	3.262654	-2.553198	1.591952
H	1.944237	0.441168	-2.874475
C	5.333055	-1.509887	-0.919748
H	4.786180	-0.330745	-2.646114
H	5.615243	-2.644493	0.897709
H	6.387820	-1.569023	-1.195287
N	1.290046	-1.166808	-0.061788
C	-1.961108	-0.808485	-1.695397
H	-2.644252	-0.278660	-2.362635
C	-0.625383	-1.071925	-2.143463
H	-0.345137	-0.714319	-3.135769
H	-0.255868	-2.075234	-1.904459
C	-2.616417	-1.852402	-0.871148
O	-2.075108	-2.847819	-0.454247
O	-3.896806	-1.554067	-0.635090
H	1.280437	2.674232	-1.254970
H	-3.504728	1.997269	0.046417
C	0.971077	2.758909	1.502943
C	1.082897	4.280253	1.696882
H	0.121934	4.717789	2.007946
H	1.826839	4.507860	2.475270
H	1.399697	4.780241	0.769052
C	2.328818	2.191440	1.078723
H	2.270530	1.114031	0.868612
H	2.740783	2.690994	0.190312
H	3.056875	2.339848	1.888946
C	-4.638361	-2.525062	0.091293
H	-5.658941	-2.135388	0.172479
H	-4.638855	-3.488151	-0.438470
H	-4.204600	-2.679869	1.090230
C	0.568657	2.107870	2.829046
H	-0.338988	2.554146	3.261843
H	0.411037	1.024511	2.719558
H	1.371992	2.255295	3.564745
H	-2.848688	2.089192	-2.386224

Intermediate **E**$^{t\text{-Bubenzene}}$

E(SMD/def2-QZVP*) = -1400.238856 E_h

ΔΔG(353) = 0.434368 E_h

Lowest frequency = 21.49 cm^{-1}

C	0.265024	-1.879907	1.091226
C	-1.307260	-0.519306	2.096745
C	-1.212940	-1.230051	3.285697
C	-0.334864	-2.306611	3.360760
C	0.403685	-2.654402	2.232657
Ru	-0.486214	0.373273	-0.755275
C	1.140754	1.964923	-0.898003
C	0.423457	1.822086	-2.124725

C	-0.976389	1.950542	-2.180475	C	-2.044148	-0.910848	-1.434993
C	-1.689209	2.149708	-0.964091	H	-2.336107	-0.578117	-2.438402
C	-0.973445	2.242689	0.250782	C	-1.261405	-2.212350	-1.418110
C	0.464759	2.209944	0.306311	H	-1.374737	-2.816074	-2.331707
H	0.977664	1.601142	-3.038666	H	-1.577225	-2.832710	-0.568086
H	-1.540795	2.352237	1.174511	C	-3.209218	-0.868486	-0.522392
H	1.054453	-3.528767	2.210835	O	-3.373642	-1.577671	0.445299
H	-0.250485	-2.894576	4.277005	O	-4.086911	0.090741	-0.864057
H	-1.841686	-0.948589	4.131035	H	2.225003	1.844773	-0.905889
H	-2.014500	0.301479	1.990092	H	-2.779727	2.156531	-0.961655
N	-0.550110	-0.801814	1.029064	C	1.252129	2.453939	1.593317
C	2.253896	-1.706159	-0.328675	C	2.041608	3.758217	1.385217
C	0.167236	-1.778482	-1.240397	H	1.371311	4.599204	1.151360
C	2.320690	-1.084538	-1.592886	H	2.594004	4.010582	2.302799
C	3.360720	-1.797610	0.507553	H	2.771521	3.667077	0.567538
C	0.979546	-1.107576	-2.143047	C	2.222343	1.299420	1.860847
C	3.538693	-0.578627	-2.057960	H	1.680739	0.363552	2.058561
C	4.562458	-1.275973	0.033770	H	2.906435	1.119395	1.019719
H	3.299326	-2.263802	1.492098	H	2.837821	1.527388	2.743608
H	0.705875	-0.894062	-3.175109	C	-5.247811	0.189479	-0.052532
C	4.654728	-0.680248	-1.233675	H	-5.875646	0.965434	-0.505030
H	3.613992	-0.118734	-3.046128	H	-5.787137	-0.767733	-0.025315
H	5.455330	-1.344074	0.658829	H	-4.986980	0.466536	0.980196
H	5.618592	-0.299681	-1.577333	C	0.339771	2.631944	2.807289
N	0.931934	-2.138551	-0.115532	H	-0.345372	3.485111	2.690247

H	-0.251011	1.729213	3.018886
H	0.954321	2.831205	3.696905
H	-1.508750	1.829725	-3.124706

Intermediate $\mathbf{F}^{\textit{t}\text{-Bubenzene}}$

E(SMD/def2-QZVP*) = -1629.196825 E_h

ΔΔG(353) = 0.489014 E_h

Lowest frequency = 23.99 cm^{-1}

C	-1.742208	0.146503	1.635824
C	0.271487	-0.493546	2.574460
C	-0.300203	-0.681019	3.820158
C	-1.663282	-0.428810	3.973444
C	-2.385850	-0.007065	2.869240
Ru	0.567549	-0.263140	-0.403151
C	0.384851	-2.528209	-0.458067
C	-0.785345	-1.855974	-0.924706
C	-0.730106	-0.927820	-1.987300
C	0.548157	-0.611592	-2.535298
C	1.710635	-1.194040	-1.999612
C	1.651352	-2.199005	-0.970119
H	-1.748995	-2.054731	-0.449904
H	2.674068	-0.832614	-2.354882
H	-3.448999	0.227225	2.933542
H	-2.152208	-0.547282	4.942735
H	0.318301	-1.020701	4.651751
H	1.328186	-0.703117	2.410196
N	-0.420901	-0.089236	1.489841
C	-3.671867	-0.088451	0.118169
C	-2.246231	1.637371	-0.285832
C	-4.170476	0.653159	-0.979533
C	-4.298376	-1.246544	0.583687
C	-3.255511	1.739635	-1.201681
C	-5.346363	0.225424	-1.612420
C	-5.461270	-1.648283	-0.065726
H	-3.902425	-1.821787	1.423326
H	-3.341005	2.522805	-1.952361
C	-5.981293	-0.918629	-1.147858
H	-5.751984	0.782968	-2.459389
H	-5.978885	-2.547524	0.274658
H	-6.898217	-1.261773	-1.631254
N	-2.485927	0.523504	0.523978
C	0.143160	1.785457	-0.895930
C	-1.011469	2.448437	-0.137465
H	-1.202666	3.453573	-0.540793
H	-0.790751	2.610874	0.934364
H	0.292385	-3.243426	0.359869
H	0.650244	0.157383	-3.302799
C	2.909425	-2.898084	-0.459767
C	2.885040	-4.316243	-1.055751
H	2.846902	-4.286717	-2.155177

H	3.795665	-4.860908	-0.763596
H	2.016248	-4.890717	-0.700811
C	2.919701	-2.980535	1.070004
H	2.967559	-1.974074	1.509943
H	2.040407	-3.501516	1.475556
H	3.806448	-3.539235	1.403303
O	2.377851	0.309641	0.569017
C	2.736975	1.281291	1.230154
O	1.893986	2.165342	1.702451
H	0.998590	1.960215	1.373238
C	4.165973	1.547404	1.525882
H	4.572497	2.085025	0.653307
H	4.283780	2.175112	2.416692
H	4.714980	0.603437	1.623747
C	1.366935	2.635891	-1.060280
O	2.397798	2.274791	-1.582647
O	1.204995	3.872308	-0.573648
C	2.309503	4.755776	-0.687735
H	2.685242	4.782818	-1.719586
H	1.947187	5.743608	-0.382238
H	3.126261	4.437480	-0.022208
H	-0.214507	1.680449	-1.932491
C	4.183611	-2.184497	-0.917138
H	4.304548	-2.212435	-2.009982
H	4.196489	-1.135086	-0.589476
H	5.059423	-2.690330	-0.485462
H	-1.637917	-0.425351	-2.326127

Transition state **TS4**$^{t\text{-Bubenzene}}$

E(SMD/def2-QZVP*) = -1629.175555 E_h

ΔΔG(353) = 0.484630 E_h

Lowest frequency = -1175.27 cm^{-1}

C	-1.711688	0.028889	1.668362
C	0.240309	-0.779396	2.607638
C	-0.355786	-0.932883	3.848614
C	-1.691616	-0.570372	3.999695
C	-2.372128	-0.082175	2.893442
Ru	0.628750	-0.299450	-0.324155
C	0.517534	-2.461518	-0.624057
C	-0.695124	-1.850712	-1.063351
C	-0.674506	-0.815216	-2.013959
C	0.589726	-0.368253	-2.499881
C	1.788638	-0.952698	-2.053997
C	1.774848	-2.037514	-1.117154
H	-1.648533	-2.147948	-0.620838
H	2.731593	-0.514926	-2.375983
H	-3.417390	0.224657	2.947605
H	-2.194453	-0.660689	4.964886
H	0.228453	-1.329645	4.679837
H	1.283890	-1.048799	2.447038

N	-0.414130	-0.309902	1.530571
C	-3.535985	-0.251419	0.051976
C	-2.303164	1.646676	-0.153021
C	-4.081585	0.517693	-1.003773
C	-4.056163	-1.494067	0.422097
C	-3.287033	1.710510	-1.102519
C	-5.187928	0.016731	-1.706821
C	-5.151394	-1.964776	-0.294411
H	-3.632488	-2.074554	1.244991
H	-3.434174	2.538700	-1.793038
C	-5.710084	-1.217891	-1.346307
H	-5.629562	0.591011	-2.524140
H	-5.587559	-2.930590	-0.030807
H	-6.571527	-1.617869	-1.884911
N	-2.436081	0.443838	0.546795
C	0.096844	2.003858	-0.565129
C	-1.165734	2.570530	0.092779
H	-1.406922	3.548604	-0.345134
H	-1.007729	2.743453	1.170228
H	0.470919	-3.231882	0.145683
H	0.649990	0.496496	-3.164401
C	3.060485	-2.728526	-0.676289
C	3.119725	-4.030709	-1.495935
H	3.107272	-3.826662	-2.577331
H	4.050971	-4.569610	-1.265164
H	2.274814	-4.696640	-1.263712
C	3.048043	-3.060409	0.818820
H	3.006946	-2.139419	1.416245
H	2.212960	-3.715739	1.106171
H	3.973057	-3.594964	1.078597
O	2.344548	0.110695	0.773972
C	2.441302	1.160197	1.479253
O	1.484553	1.965369	1.583283
H	0.763737	1.652551	0.555648
C	3.736703	1.439591	2.165017
H	4.344896	2.055293	1.483198
H	3.568972	2.014899	3.083426
H	4.285101	0.512557	2.369518
C	1.199487	2.956259	-0.946709
O	2.213914	2.589935	-1.491346
O	0.940654	4.214509	-0.622080
C	1.954539	5.166994	-0.920511
H	2.180589	5.169156	-1.995865
H	1.560944	6.140196	-0.608393
H	2.874304	4.931057	-0.366476
H	-0.207432	1.590247	-1.530074
C	4.297450	-1.880733	-0.982338
H	4.448164	-1.741580	-2.062951
H	4.233980	-0.895050	-0.500741
H	5.191695	-2.394194	-0.600927

H	-1.602629	-0.315883	-2.300729

Intermediate **G**$^{\boldsymbol{t}\text{-Bubenzene}}$

E(SMD/def2-QZVP*) = -1629.194335 E_h

ΔΔG(353) = 0.486437 E_h

Lowest frequency = 12.91 cm^{-1}

C	-1.678340	0.491743	1.589780
C	0.231603	-0.209261	2.690887
C	-0.326150	0.055805	3.931859
C	-1.612277	0.582412	3.991025
C	-2.293575	0.803333	2.800401
Ru	0.570470	-0.497016	-0.273342
C	0.481178	-2.616283	-0.099524
C	-0.790113	-2.160848	-0.566809
C	-0.878453	-1.354731	-1.712279
C	0.327434	-0.993845	-2.371926
C	1.585503	-1.473236	-1.945171
C	1.686215	-2.317639	-0.801004
H	-1.691667	-2.363104	0.015211
H	2.483664	-1.113771	-2.443961
H	-3.304090	1.213209	2.779178
H	-2.078928	0.821600	4.948941
H	0.254190	-0.141418	4.833977
H	1.244584	-0.599728	2.593548
N	-0.426251	-0.002325	1.539390
C	-3.491409	-0.180297	0.085454
C	-2.287213	1.628195	-0.573787
C	-4.040272	0.294556	-1.130273
C	-4.005067	-1.292044	0.758836
C	-3.262993	1.438086	-1.516477
C	-5.136842	-0.381400	-1.689303
C	-5.090220	-1.940138	0.179970
H	-3.585213	-1.633882	1.707940
H	-3.420079	2.070512	-2.388186
C	-5.647347	-1.491240	-1.031329
H	-5.581099	-0.035442	-2.625158
H	-5.522542	-2.809799	0.679340
H	-6.501173	-2.024212	-1.454455
N	-2.406315	0.629792	0.398045
C	0.105404	2.043098	-1.060339
C	-1.195083	2.638374	-0.540436
H	-1.483239	3.488749	-1.173107
H	-1.032109	3.036208	0.473067
H	0.527895	-3.178562	0.832936
H	0.294930	-0.278838	-3.197331
C	3.023345	-2.870786	-0.329763
C	3.083822	-4.297604	-0.907294
H	3.013482	-4.292910	-2.005536
H	4.042795	-4.761960	-0.632428
H	2.274736	-4.931732	-0.513719

C	3.114013	-2.928772	1.198184
H	3.025090	-1.920602	1.625278
H	2.355134	-3.586099	1.647484
H	4.092942	-3.338963	1.484423
O	2.274283	0.119868	0.636898
C	2.473628	1.297154	1.173991
O	1.588677	2.112425	1.384412
H	0.521093	1.423627	-0.208985
C	3.919141	1.579044	1.474243
H	4.383269	1.929392	0.538582
H	4.000952	2.372855	2.226138
H	4.452736	0.675666	1.796530
C	1.292121	2.958117	-1.278316
O	2.353815	2.549636	-1.673852
O	1.019132	4.216273	-0.986731
C	2.103337	5.135913	-1.072072
H	2.854387	4.895991	-0.306053
H	2.572615	5.090859	-2.064247
H	1.677524	6.127964	-0.888993
H	-0.056578	1.500014	-1.996599
C	4.197801	-2.048642	-0.865876
H	4.108360	-0.995153	-0.566488
H	5.136146	-2.448433	-0.455786
H	4.281435	-2.107454	-1.961132
H	-1.836335	-0.928143	-2.017106

Intermediate $\mathbf{H}^{t\text{-Bubenzene}}$

E(SMD/def2-QZVP*) = -1629.222437 E_h

ΔΔG(353) = 0.486591 E_h

Lowest frequency = 15.77 cm^{-1}

C	-0.213619	0.905275	1.741566
C	-0.499456	-1.304956	2.359413
C	-0.696597	-0.961223	3.688758
C	-0.662859	0.382169	4.047679
C	-0.404218	1.322117	3.058088
Ru	-0.366631	-1.142933	-0.632159
C	-2.093471	0.101178	-1.130152
C	-1.018452	0.451808	-1.976757
C	-0.353737	-0.567487	-2.706026
C	-0.783011	-1.910108	-2.608436
C	-1.844845	-2.231794	-1.720216
C	-2.544509	-1.240354	-0.973630
H	-0.653608	1.479983	-1.997386
H	-2.067565	-3.285411	-1.546793
H	-0.328172	2.387528	3.279217
H	-0.813265	0.693551	5.083585
H	-0.874121	-1.747339	4.423826
H	-0.502553	-2.347570	2.040816
N	-0.290375	-0.395958	1.387999
C	-0.873318	2.804344	0.312661

C	1.276546	2.084506	0.125071
C	-0.231324	3.581780	-0.682421
C	-2.205261	3.006185	0.682676
C	1.121932	3.107217	-0.774506
C	-0.967219	4.582283	-1.337160
C	-2.905739	4.007965	0.019182
H	-2.677340	2.415712	1.471305
H	1.911306	3.521130	-1.397995
C	-2.294422	4.782742	-0.982723
H	-0.498667	5.197346	-2.108598
H	-3.945744	4.203033	0.289379
H	-2.873960	5.562489	-1.481019
N	0.049121	1.867861	0.759829
C	3.587350	1.270286	-0.457515
C	2.500074	1.377922	0.592685
H	2.892638	1.907250	1.479860
H	2.256432	0.364814	0.933325
H	-2.520601	0.890178	-0.511729
H	-0.230814	-2.705980	-3.110076
C	-3.730436	-1.601444	-0.085466
C	-4.960817	-1.651609	-1.005837
H	-4.842865	-2.412336	-1.792412
H	-5.859481	-1.904275	-0.423012
H	-5.134494	-0.680993	-1.494746
C	-3.958741	-0.548644	1.002447
H	-3.073023	-0.424700	1.643841
H	-4.228810	0.432175	0.584741
H	-4.794050	-0.862397	1.644541
O	0.502943	-2.871839	0.212635
C	1.679033	-2.428343	0.015052
O	1.741688	-1.304022	-0.566694
H	3.174916	0.844632	-1.387239
C	2.894200	-3.169087	0.428787
H	3.608372	-2.466877	0.883480
H	2.636477	-3.988851	1.109016
H	3.372723	-3.587214	-0.470565
C	4.694000	0.347722	-0.018765
O	4.606038	-0.460849	0.873791
O	5.785468	0.511802	-0.756680
C	6.886129	-0.336564	-0.459669
H	7.196029	-0.218300	0.588288
H	6.622328	-1.390501	-0.630807
H	7.695565	-0.035365	-1.133410
H	4.023263	2.243775	-0.726049
C	-3.532132	-2.966108	0.580871
H	-3.501265	-3.791197	-0.144979
H	-2.606063	-2.996427	1.173560
H	-4.374703	-3.172336	1.256314
H	0.552065	-0.327208	-3.266402

***p*-cymene**

E(SMD/def2-QZVP*) = -389.215198 E_h

ΔΔG(353) = 0.166011 E_h

Lowest frequency = 16.58 cm^{-1}

C	-1.600312	-1.198595	0.000014
C	-2.200051	0.062573	0.000039
C	-1.356630	1.181970	0.000060
C	0.026381	1.042771	0.000040
C	0.628296	-0.222973	0.000005
C	-0.212377	-1.337511	-0.000007
H	-2.230566	-2.092538	0.000018
H	-1.795719	2.184133	0.000100
H	0.650550	1.940901	0.000063
H	0.228153	-2.338955	-0.000021
C	2.134365	-0.379194	-0.000024
H	2.340459	-1.463515	-0.000059
C	2.762952	0.209817	-1.262510
H	3.850355	0.035097	-1.278393
H	2.329689	-0.237467	-2.169545
H	2.600785	1.298244	-1.317518
C	2.762997	0.209744	1.262474
H	2.329774	-0.237600	2.169498
H	3.850403	0.035036	1.278305
H	2.600821	1.298166	1.317556
C	-3.692672	0.224603	-0.000047
H	-4.034532	0.783068	-0.886198
H	-4.203394	-0.748599	0.000895
H	-4.034476	0.784795	0.885025

benzene

E(SMD/def2-QZVP*) = -232.072172 E_h

ΔΔG(353) = 0.067643 E_h

Lowest frequency = 416.11 cm^{-1}

C	0.018893	-1.394571	-0.000000
C	1.217192	-0.680925	-0.000003
C	1.198298	0.713649	0.000002
C	-0.018892	1.394571	-0.000001
C	-1.217192	0.680926	-0.000002
C	-1.198298	-0.713648	0.000003
H	0.033690	-2.487177	-0.000001
H	2.170810	-1.214419	0.000002
H	2.137118	1.272770	0.000006
H	-0.033688	2.487174	0.000004
H	-2.170807	1.214417	-0.000006
H	-2.137122	-1.272770	0.000005

***t*-Bubenzene**

E(SMD/def2-QZVP*) = -389.210794 E_h

ΔΔG(353) = 0.170727 E_h

Lowest frequency = 49.22 cm^{-1}

C	-2.207105	1.201807	0.000021
C	-0.811318	1.217179	0.000047
C	-0.071178	0.029813	0.000053
C	-0.786734	-1.177639	0.000038
C	-2.177786	-1.199472	0.000009
C	-2.898430	-0.005321	0.000001
H	-2.755331	2.147332	0.000021
H	-0.300304	2.180549	0.000071
H	-0.246178	-2.127529	0.000039
H	-2.703861	-2.157462	-0.000005
H	-3.990894	-0.018238	-0.000020
C	1.459174	0.005706	0.000013
C	1.952917	-0.729681	1.254479
H	3.054094	-0.761141	1.273383
H	1.608476	-0.221395	2.168125
H	1.588017	-1.766749	1.290519
C	1.952693	-0.729267	-1.254788
H	1.608137	-0.220620	-2.168191
H	3.053866	-0.760802	-1.273881
H	1.587679	-1.766286	-1.291142
C	2.057973	1.412672	0.000189
H	1.759077	1.984535	-0.891561
H	1.759182	1.984263	0.892150
H	3.156800	1.348758	0.000109

Alkene **3a**

E(SMD/def2-QZVP*) = -306.263685 E_h

ΔΔG(353) = 0.058388 E_h

Lowest frequency = 98.57 cm^{-1}

C	-0.039118	0.113460	-0.000001
O	0.060969	1.314493	0.000005
O	1.014277	-0.714085	0.000034
C	2.284595	-0.090371	0.000079
H	3.026256	-0.897672	0.000105
H	2.410819	0.543173	-0.890819
H	2.410756	0.543173	0.890986
C	-1.311072	-0.647737	-0.000047
C	-2.481310	-0.007576	-0.000086
H	-1.236107	-1.738033	-0.000046
H	-3.431251	-0.546877	-0.000120
H	-2.501012	1.086327	-0.000084

HOAc

E(SMD/def2-QZVP*) = -228.952262 E_h

ΔΔG(353) = 0.029151 E_h

Lowest frequency = 79.73 cm^{-1}

C	-0.092792	0.121673	-0.000005
O	-0.637637	1.192942	-0.000033
O	-0.776657	-1.033953	-0.000017
H	-1.715791	-0.792741	-0.000050

C	1.388081	-0.110577	0.000048
H	1.673345	-0.698450	0.884681
H	1.673400	-0.698526	-0.884515
H	1.911661	0.851229	0.000025

HCl

E(SMD/def2-QZVP*) = -460.684264 E_h

ΔΔG(353) = -0.014961 E_h

Lowest frequency = 3018.19 cm^{-1}

Cl	0.000000	0.000000	0.071392
H	0.000000	0.000000	-1.213660

[RuCl$_2$(*p*-cymene)]$_2$

E(SMD/def2-QZVP*) = -2808.883869 E_h

ΔΔG(353) = 0.357479 E_h

Lowest frequency = 9.69 cm^{-1}

C	-3.196188	-0.881133	-1.724727
C	-2.371988	-1.963468	-1.260467
C	-2.269199	-2.159877	0.134134
C	-2.946720	-1.292237	1.044799
C	-3.760441	-0.219736	0.599562
C	-3.875624	-0.038364	-0.819662
Ru	-1.811333	-0.089646	-0.293817
Cl	0.001572	0.128449	-1.916935
Ru	1.809584	0.132269	-0.273456
Cl	-0.004440	-0.066608	1.344309
C	-1.560623	-2.786344	-2.207202
C	-4.409728	0.767555	1.538076
C	-5.859333	0.356010	1.795703
C	3.763883	0.127707	0.618783
C	3.871526	0.130798	-0.812916
C	3.195636	1.089471	-1.597373
C	2.382693	2.109581	-0.992661
C	2.289094	2.124351	0.416242
C	2.963022	1.140788	1.204020
C	1.572043	3.053204	-1.819882
C	4.407444	-0.979552	1.416745
C	5.867609	-0.627189	1.701042
Cl	1.733421	-2.245157	-0.408586
Cl	-1.759048	2.286406	-0.143414
C	3.640625	-1.331765	2.683813
C	-3.629077	0.972261	2.828988
H	3.195710	0.984858	-2.684214
H	4.373563	-0.703630	-1.307028
H	2.745271	1.096065	2.271915
H	1.573127	2.796338	0.892809
H	2.087522	4.024532	-1.891154
H	0.580044	3.198613	-1.367041
H	1.422809	2.659663	-2.833339
H	4.373735	-1.866664	0.762902

H	6.423475	-0.414491	0.774940
H	6.372464	-1.459410	2.214608
H	5.939667	0.262356	2.347470
H	3.683510	-0.524377	3.432535
H	4.079562	-2.226550	3.149109
H	2.587783	-1.548344	2.450087
H	-1.544442	-2.881035	0.516197
H	-2.723097	-1.383268	2.108609
H	-4.387192	0.848501	-1.199421
H	-3.204023	-0.638008	-2.789120
H	-0.566714	-2.986507	-1.780037
H	-1.417225	-2.262506	-3.160795
H	-2.072935	-3.741979	-2.403382
H	-4.401041	1.729943	1.000161
H	-3.646673	0.076588	3.470675
H	-4.075563	1.793313	3.408956
H	-2.583653	1.235456	2.610407
H	-5.906636	-0.609163	2.325395
H	-6.426357	0.251912	0.857858
H	-6.369247	1.107450	2.417440

[Ru(OAc)$_2$(*p*-cymene)]

E(SMD/def2-QZVP*) = -940.928367 E_h

$\Delta\Delta G(353)$ = 0.254300 E_h

Lowest frequency = 19.80 cm^{-1}

C	1.948116	0.134342	0.046793
C	1.733976	-1.254545	0.339047
H	2.096856	-2.011710	-0.357625
C	0.932686	-1.661312	1.424001
H	0.698693	-2.719857	1.552988
C	0.274448	-0.688223	2.244652
C	0.513839	0.684570	1.989350
H	-0.052312	1.452960	2.514923
C	1.344993	1.083413	0.902232
H	1.373534	2.143201	0.647368
C	2.744866	0.588941	-1.153850
H	2.358278	1.589742	-1.409287
C	4.216354	0.732049	-0.762631
H	4.344863	1.423145	0.083907
H	4.806856	1.114565	-1.609114
H	4.640670	-0.242036	-0.469803
C	2.566628	-0.304916	-2.376032
H	3.048622	-1.286051	-2.236428
H	3.040052	0.160676	-3.253223
H	1.503065	-0.473675	-2.598974
C	-0.715403	-1.123843	3.277534
H	-1.316700	-1.965832	2.907200
H	-1.395654	-0.302600	3.538442
H	-0.192136	-1.445779	4.192271
C	-1.793125	-1.555271	-1.199943

C	-1.543438	2.359108	-0.138215
Ru	-0.169599	-0.273890	0.189433
O	-0.699321	-1.150383	-1.686672
O	-1.958619	-1.426229	0.046955
O	-1.434034	1.127062	-0.533233
O	-0.879858	2.900389	0.732549
C	-2.625426	3.106886	-0.890170
H	-3.592036	2.602419	-0.744958
H	-2.686081	4.143242	-0.537347
H	-2.410705	3.086849	-1.968746
C	-2.866062	-2.119944	-2.069395
H	-3.483799	-2.831178	-1.507062
H	-3.507634	-1.287688	-2.397780
H	-2.432121	-2.593490	-2.958806

Acetate

E(SMD/def2-QZVP*) = -228.467675 E_h

ΔΔG(353) = 0.014592 E_h

Lowest frequency = 40.43 cm^{-1}

C	0.220014	0.001633	-0.000009
O	0.802620	-1.099484	-0.000016
O	0.692779	1.155456	-0.000035
C	-1.346982	-0.054735	0.000046
H	-1.737753	0.480214	0.883510
H	-1.737817	0.480304	-0.883334
H	-1.725813	-1.089684	0.000013

Pyridyl indole

E(SMD/def2-QZVP*) = -610.478575 E_h

ΔΔG(353) = 0.154933 E_h

Lowest frequency = 56.61 cm^{-1}

C	1.116100	-0.091876	0.012371
C	2.186608	0.835163	-0.106455
C	3.509840	0.375742	-0.057932
C	3.747838	-0.978842	0.126283
C	2.680891	-1.878725	0.281726
C	1.358517	-1.452177	0.232279
C	0.249841	1.981677	-0.178029
C	1.601870	2.141166	-0.228519
H	4.338883	1.080918	-0.153375
H	4.774280	-1.350352	0.166538
H	2.890060	-2.937108	0.453332
H	0.550751	-2.165992	0.389938
H	-0.559897	2.704483	-0.224131
H	2.130227	3.085459	-0.339015
N	-0.067497	0.636142	-0.052808
C	-1.389186	0.173129	-0.028504
C	-1.731098	-1.108442	-0.486307
N	-2.294466	1.042187	0.410039
C	-3.065099	-1.489573	-0.422054

H	-0.979707	-1.768399	-0.916311
C	-3.568702	0.670774	0.448794
C	-4.013671	-0.591983	0.065073
H	-3.363486	-2.481210	-0.770769
H	-4.279138	1.422572	0.810986
H	-5.070723	-0.854871	0.129829

Cartesian Coordinates and Energies

(PW6B95-D3(BJ)/def2-QZVP*+SMD(AcOH)/M06-D3/def2-SVP level of theory

Intermediate **A**

E(SMD/def2-QZVP*) = -1325.968745 E_h

$\Delta\Delta$G(353) = 0.389382 E_h

Lowest frequency = 24.16 cm^{-1}

C	0.902682	0.359349	1.805232
C	-1.108515	1.462816	2.141668
C	-0.573185	2.235614	3.159156
C	0.765833	2.057592	3.500197
C	1.507027	1.100317	2.822941
Ru	-1.281489	-0.281513	-0.330966
C	0.242927	0.582488	-1.656670
C	0.197937	-0.811612	-1.853116
C	-1.037459	-1.472607	-2.141422
C	-2.211388	-0.684721	-2.245051
C	-2.153308	0.717050	-2.008841
C	-0.932742	1.385577	-1.716480
H	1.102276	-1.408120	-1.694092
H	-3.088777	1.285578	-1.952097
H	2.548626	0.889069	3.076555
H	1.222606	2.643303	4.301951
H	-1.203389	2.964088	3.672509
H	-2.157161	1.560332	1.845551
N	-0.383033	0.558450	1.453862
C	2.844475	-0.361818	0.448324
C	1.381064	-1.974175	1.073172
C	3.316735	-1.604828	-0.034748
C	3.502071	0.843558	0.187843
C	2.374604	-2.607099	0.387968
C	4.498398	-1.633398	-0.790989
C	4.667881	0.782351	-0.565555
H	3.123827	1.800035	0.563914
H	2.442450	-3.680797	0.217892
C	5.161529	-0.442594	-1.046887
H	4.887737	-2.581365	-1.172562
H	5.213272	1.704506	-0.783408
H	6.084268	-0.452546	-1.632752
N	1.645783	-0.603488	1.117803
O	-3.045454	-0.290544	0.867659
C	-2.763629	-1.425459	1.364343
O	-1.720470	-1.989659	0.921249
C	-3.589975	-2.028778	2.438547
H	-3.496384	-3.122269	2.431719
H	-3.226928	-1.661236	3.411900
H	-4.639756	-1.724689	2.334891

H 0.485076 -2.366763 1.554421
H 1.193267 1.025370 -1.339945
H -3.180593 -1.173080 -2.381005
C -1.088705 -2.959176 -2.208240
H -0.851900 -3.297623 -3.230540
H -0.348298 -3.403448 -1.525943
H -2.084988 -3.341115 -1.943868
C -0.936046 2.875328 -1.469987
H -1.838598 3.085978 -0.861030
C -1.082299 3.597159 -2.808376
H -1.992258 3.291293 -3.348220
H -1.129625 4.686778 -2.659120
H -0.217389 3.384474 -3.459673
C 0.283047 3.381589 -0.717102
H 0.170937 4.453468 -0.495262
H 0.439180 2.858865 0.241197
H 1.202442 3.276233 -1.318969

Transition state **TS1**

E(SMD/def2-QZVP*) = -1325.948371 E_h

ΔΔG(353) = 0.389554 E_h

Lowest frequency = -111.19 cm^{-1}

C -1.086594 1.090844 -1.611057
C 0.558484 2.634767 -1.082391
C -0.148045 3.656747 -1.700204
C -1.380864 3.363237 -2.281947
C -1.852829 2.056078 -2.258335
Ru 1.030605 -0.128723 0.220437
C -0.392077 -0.345103 1.902916
C 0.280698 -1.563591 1.687914
C 1.706951 -1.667277 1.658000
C 2.441547 -0.470857 1.818959
C 1.783052 0.779633 1.984207
C 0.354387 0.860690 2.036912
H -0.311337 -2.456392 1.463878
H 2.372520 1.702927 2.011417
H -2.777742 1.762183 -2.759824
H -1.957642 4.144869 -2.782820
H 0.270291 4.664369 -1.731874
H 1.541437 2.801762 -0.630455
N 0.075768 1.387010 -1.002667
C -2.551327 -0.808666 -0.932255
C -0.449406 -1.241675 -1.654467
C -2.270351 -2.179561 -0.695333
C -3.733391 -0.206614 -0.494363
C -0.947396 -2.428973 -1.177522
C -3.223276 -2.972182 -0.031332
C -4.648831 -1.013450 0.166946
H -3.938507 0.854103 -0.664466
H -0.415269 -3.380510 -1.204554

C	-4.401009	-2.381563	0.392696
H	-3.032287	-4.034078	0.147616
H	-5.589067	-0.577204	0.514777
H	-5.154173	-2.981471	0.909589
N	-1.442357	-0.256314	-1.560890
O	2.619241	0.416627	-1.010148
C	3.042991	-0.638166	-1.629977
O	2.484226	-1.728119	-1.495747
C	4.253322	-0.444612	-2.491859
H	4.389399	-1.305263	-3.158290
H	4.176026	0.485353	-3.072308
H	5.142544	-0.349875	-1.849299
H	0.443647	-1.079559	-2.260371
H	-1.485571	-0.326618	1.854126
H	3.526695	-0.489563	1.673640
C	2.373957	-2.969722	1.376379
H	2.526952	-3.527512	2.315195
H	1.761621	-3.588845	0.705155
H	3.349585	-2.822228	0.893684
C	-0.291368	2.204060	2.260634
H	0.311918	2.943529	1.697142
C	-0.174066	2.536075	3.749113
H	0.871266	2.536119	4.095397
H	-0.598735	3.530495	3.955097
H	-0.730293	1.801030	4.355208
C	-1.734787	2.302448	1.798974
H	-2.097660	3.334599	1.918483
H	-1.862243	2.023823	0.740896
H	-2.400251	1.659416	2.400111

Intermediate **B**

E(SMD/def2-QZVP*) = -1325.951899 E_h

ΔΔG(353) = 0.388463 E_h

Lowest frequency = 31.67 cm^{-1}

C	-1.185912	1.010742	-1.674563
C	0.117517	2.788510	-0.964760
C	-0.706729	3.697718	-1.615176
C	-1.818653	3.220236	-2.306653
C	-2.054778	1.850042	-2.361228
Ru	0.916205	0.016344	0.232307
C	-0.371457	-0.459745	1.971139
C	0.572987	-1.499483	1.775378
C	1.973351	-1.268492	1.714445
C	2.399461	0.084969	1.807109
C	1.477332	1.143935	1.985109
C	0.070391	0.881662	2.074024
H	0.207233	-2.519645	1.620123
H	1.835073	2.178818	2.005013
H	-2.870016	1.423067	-2.949674
H	-2.481804	3.911230	-2.833158

H	-0.469939	4.762843	-1.586807
H	1.017562	3.105313	-0.428284
N	-0.146634	1.477046	-0.957498
C	-2.262291	-1.150949	-1.074099
C	-0.058089	-1.098062	-1.611035
C	-1.686573	-2.395194	-0.702807
C	-3.584585	-0.829680	-0.758481
C	-0.310155	-2.346547	-1.069476
C	-2.472395	-3.357465	-0.041836
C	-4.330856	-1.795065	-0.098210
H	-4.020902	0.137095	-1.023755
H	0.438342	-3.137668	-1.002465
C	-3.787616	-3.048241	0.252011
H	-2.046730	-4.327016	0.232343
H	-5.373662	-1.580652	0.151141
H	-4.419611	-3.780254	0.760555
N	-1.277472	-0.384259	-1.677426
O	2.406786	0.527787	-1.099852
C	3.145531	-0.429274	-1.591090
O	2.918086	-1.618617	-1.420264
C	4.321866	0.066079	-2.387187
H	4.762240	-0.755955	-2.965033
H	4.028224	0.891026	-3.051429
H	5.083185	0.464645	-1.698360
H	0.755123	-0.834038	-2.289011
H	-1.439770	-0.697756	1.963714
H	3.454935	0.318841	1.632255
C	2.942781	-2.383880	1.520910
H	3.315595	-2.729919	2.499383
H	2.473442	-3.237104	1.011040
H	3.797208	-2.067934	0.907456
C	-0.873903	2.036151	2.292146
H	-0.441179	2.906685	1.760682
C	-0.880401	2.355673	3.787659
H	0.129156	2.573469	4.170097
H	-1.516134	3.230830	3.991791
H	-1.282433	1.504999	4.363646
C	-2.284705	1.813647	1.776363
H	-2.880563	2.730208	1.903933
H	-2.304987	1.547070	0.707157
H	-2.805559	1.016086	2.333370

Transition state **TS2**

E(SMD/def2-QZVP*) = -1325.941160 E_h

ΔΔG(353) = 0.385173 E_h

Lowest frequency = -985.57 cm^{-1}

C	0.748172	-1.666426	0.855463
C	-1.279880	-1.852430	1.981049
C	-0.810677	-2.901750	2.752404
C	0.498384	-3.340850	2.545938

C	1.287165	-2.731102	1.582343
Ru	-1.171928	0.411726	-0.100130
C	-0.154914	2.255844	0.572043
C	-0.709884	2.436281	-0.732191
C	-2.070994	2.187645	-1.014567
C	-2.956177	1.749246	0.018422
C	-2.422925	1.550964	1.312959
C	-1.038459	1.782115	1.572503
C	1.301817	2.559707	0.821280
H	-0.037577	2.707217	-1.553415
H	-2.440305	2.272150	-2.040344
H	-3.066225	1.150457	2.102643
H	-0.639842	1.523498	2.558986
H	1.847376	2.237231	-0.087245
H	2.287722	-3.098899	1.352129
H	0.896930	-4.180484	3.121055
H	-1.463000	-3.373333	3.489053
H	-2.305871	-1.482667	2.074071
N	-0.510920	-1.231165	1.073852
C	2.742006	-0.778303	-0.399924
C	0.608567	-0.329095	-1.141644
C	2.832375	0.009027	-1.580810
C	3.884175	-1.133792	0.326444
C	1.503397	0.269148	-2.016759
C	4.093577	0.402310	-2.064540

C	5.111465	-0.718543	-0.169314
H	3.830956	-1.697149	1.260236
H	1.219954	0.806970	-2.923149
C	5.221075	0.032341	-1.356228
H	4.172386	0.997656	-2.978150
H	6.019610	-0.981600	0.379807
H	6.210970	0.329078	-1.711352
N	1.398598	-0.997660	-0.159298
O	-2.499284	-1.005884	-0.887798
C	-2.183671	-1.956908	-1.676948
O	-1.029881	-2.152023	-2.106624
H	-0.222893	-1.102308	-1.659320
C	-3.292967	-2.858342	-2.124703
H	-3.660607	-2.499554	-3.099066
H	-2.913892	-3.877789	-2.273785
H	-4.128773	-2.847846	-1.414108
C	1.893366	1.826750	2.013654
H	2.982625	1.983322	2.049074
H	1.486053	2.201473	2.968500
H	1.719571	0.738235	1.967656
C	1.472136	4.070263	0.967995
H	2.533588	4.330373	1.101036
H	1.099533	4.616406	0.086670
H	0.921691	4.440815	1.849732
C	-4.368347	1.402205	-0.297371

H	-4.992328	2.310862	-0.268802
H	-4.449183	0.965366	-1.302933
H	-4.776776	0.681762	0.425252

Intermediate **C**

E(SMD/def2-QZVP*) = -1325.963398 E_h

ΔΔG(353) = 0.385144 E_h

Lowest frequency = 11.67 cm^{-1}

C	1.160627	-0.871788	1.433279
C	-0.825426	-1.538405	2.458868
C	-0.151850	-2.188255	3.475349
C	1.244369	-2.176818	3.445175
C	1.909683	-1.524208	2.421747
Ru	-1.197418	-0.004669	-0.163616
C	-1.577869	2.117264	0.057611
C	-1.806863	1.744529	-1.305631
C	-2.671928	0.684390	-1.632953
C	-3.455592	0.017198	-0.626373
C	-3.274363	0.402509	0.706768
C	-2.296236	1.390484	1.045699
C	-0.635675	3.246103	0.391426
H	-1.218676	2.225463	-2.094103
H	-2.748677	0.351026	-2.672719
H	-3.818204	-0.121384	1.498967
H	-2.088968	1.587253	2.103157
H	0.083874	3.307139	-0.447171
H	2.996063	-1.532660	2.374594
H	1.819173	-2.688677	4.221231
H	-0.707015	-2.698228	4.263957
H	-1.918352	-1.532790	2.414081
N	-0.192900	-0.888431	1.468276
C	2.990434	0.117171	-0.032167
C	0.760072	0.298867	-0.606897
C	2.892124	0.781176	-1.279051
C	4.229319	-0.092123	0.575978
C	1.491498	0.878189	-1.611298
C	4.048972	1.216834	-1.932634
C	5.365990	0.352642	-0.092937
H	4.344542	-0.568131	1.549619
H	1.084446	1.348590	-2.508314
C	5.281610	0.995100	-1.334336
H	3.977333	1.727893	-2.896754
H	6.344609	0.197851	0.368863
H	6.196150	1.329653	-1.830668
N	1.675653	-0.190812	0.358154
O	-1.084052	-2.036949	-0.939049
C	-0.319087	-2.659369	-1.679522
O	0.733846	-2.141737	-2.244301
H	0.850877	-1.190664	-1.988821
C	-0.538299	-4.092792	-2.004150

H	-0.591362	-4.218124	-3.095917
H	0.330216	-4.677046	-1.664142
H	-1.452808	-4.465207	-1.530122
C	0.158932	3.033477	1.668724
H	0.865461	3.864603	1.813987
H	-0.488095	3.010892	2.562505
H	0.745781	2.100917	1.637804
C	-1.435974	4.545577	0.438549
H	-0.771132	5.406937	0.605805
H	-1.989470	4.722591	-0.497606
H	-2.169989	4.522512	1.262730
C	-4.347174	-1.111833	-1.013320
H	-5.246108	-0.732066	-1.526309
H	-3.832908	-1.790279	-1.711670
H	-4.671745	-1.693950	-0.139542

Intermediate **D**

E(SMD/def2-QZVP*) = -1403.482404 E_h

ΔΔG(353) = 0.423396 E_h

Lowest frequency = 24.43 cm^{-1}

C	-1.108261	1.061839	0.929375
C	0.987226	1.689251	1.757131
C	0.429553	2.533502	2.694818
C	-0.965243	2.644845	2.728728
C	-1.738953	1.917656	1.845537
Ru	1.062380	-0.421968	-0.441454
C	0.842029	-2.641139	-0.001977
C	1.592656	-2.457226	-1.187229
C	2.798030	-1.698916	-1.179904
C	3.239595	-1.141425	0.055307
C	2.431260	-1.237394	1.189729
C	1.202117	-1.986596	1.192793
H	1.249296	-2.908727	-2.123401
H	2.732651	-0.728638	2.112776
H	-2.821756	2.013079	1.847860
H	-1.449507	3.312022	3.446376
H	1.071006	3.100504	3.371143
H	2.072106	1.595297	1.652533
N	0.243909	0.957678	0.904393
C	-3.089564	0.029777	-0.283390
C	-0.922807	-0.490456	-0.856682
C	-3.100712	-0.885167	-1.363765
C	-4.275224	0.499556	0.280286
C	-1.730478	-1.187484	-1.701586
C	-4.319500	-1.323769	-1.888696
C	-5.476534	0.046068	-0.258839
H	-4.305566	1.196470	1.117711
H	-1.402183	-1.850006	-2.504279
C	-5.501277	-0.852377	-1.331409
H	-4.336975	-2.027247	-2.725799

H	-6.416514	0.404034	0.168992
H	-6.462106	-1.185498	-1.732336
N	-1.734284	0.270293	0.015127
C	1.732245	1.349987	-1.588462
H	2.829375	1.368739	-1.569029
C	1.030330	0.498347	-2.447503
H	1.560956	-0.157639	-3.143426
H	0.017339	0.781622	-2.746837
C	1.085599	2.619205	-1.154256
O	-0.071341	2.904933	-1.321959
O	1.969349	3.413548	-0.540534
H	-0.098369	-3.196549	-0.054414
H	4.170307	-0.565553	0.087041
C	0.405790	-2.092820	2.471108
H	0.427712	-1.083746	2.932162
C	1.113342	-3.062232	3.417091
H	2.154842	-2.766633	3.621534
H	0.584557	-3.115739	4.380942
H	1.130501	-4.078298	2.987377
C	-1.046022	-2.492949	2.265962
H	-1.573602	-1.841127	1.550109
H	-1.133313	-3.533312	1.908865
H	-1.586428	-2.444602	3.223122
C	3.635896	-1.565717	-2.408111
H	4.030395	-0.544821	-2.529424
H	4.505264	-2.239445	-2.332508
H	3.085599	-1.844994	-3.317809
C	1.480121	4.682122	-0.124836
H	2.316756	5.199605	0.358159
H	1.122428	5.262612	-0.988079
H	0.641810	4.562572	0.580610

Transition state **TS3**

E(SMD/def2-QZVP*) = -1403.462204 E_h

ΔΔG(353) = 0.423437 E_h

Lowest frequency = -262.37 cm^{-1}

C	-0.930400	1.481992	0.698631
C	1.135607	1.594761	1.760407
C	0.744914	2.675961	2.526150
C	-0.535506	3.195562	2.325187
C	-1.379801	2.605392	1.401226
Ru	0.873907	-0.629788	-0.351212
C	0.112972	-2.685190	0.145198
C	0.832396	-2.697464	-1.083635
C	2.173741	-2.244203	-1.163439
C	2.783818	-1.750679	0.033191
C	2.040101	-1.668163	1.220762
C	0.683314	-2.139317	1.307845
H	0.335559	-3.047949	-1.994613
H	2.492187	-1.205325	2.105421

H	-2.360359	3.027136	1.184825
H	-0.868783	4.077290	2.878093
H	1.438670	3.118336	3.242866
H	2.148030	1.184416	1.832098
N	0.319344	0.999290	0.873778
C	-3.057183	0.677854	-0.411504
C	-1.013029	-0.113084	-1.096282
C	-3.257070	-0.283414	-1.434693
C	-4.132609	1.264545	0.255813
C	-1.967894	-0.752849	-1.848114
C	-4.560193	-0.617639	-1.827289
C	-5.415594	0.908389	-0.148643
H	-4.004288	1.968764	1.078756
H	-1.769032	-1.462899	-2.652692
C	-5.629535	-0.012862	-1.184170
H	-4.725322	-1.350962	-2.621373
H	-6.273660	1.358911	0.356946
H	-6.652298	-0.262168	-1.478065
N	-1.671824	0.793542	-0.232363
C	1.723449	0.828308	-1.697477
H	2.546590	0.312526	-2.203369
C	0.397819	0.696496	-2.215595
H	0.258863	0.077209	-3.108077
H	-0.195812	1.620402	-2.214566
C	2.110998	2.119367	-1.086035
O	1.374384	3.057256	-0.903589
O	3.404635	2.112787	-0.736385
H	-0.938457	-2.986010	0.143369
H	3.800404	-1.345840	-0.004557
C	-0.064909	-2.013184	2.611114
H	0.278486	-1.065293	3.072376
C	0.338809	-3.162371	3.532516
H	1.427793	-3.200693	3.696102
H	-0.146351	-3.061775	4.515521
H	0.029799	-4.130093	3.102021
C	-1.573874	-1.939025	2.444008
H	-1.881688	-1.157566	1.727851
H	-1.993527	-2.899212	2.098872
H	-2.051889	-1.716751	3.409808
C	2.922350	-2.293638	-2.454194
H	3.759038	-1.579568	-2.471499
H	3.348812	-3.298826	-2.605151
H	2.262076	-2.085948	-3.311124
C	3.912274	3.325436	-0.197709
H	4.975990	3.159096	0.006779
H	3.789390	4.152163	-0.913161
H	3.383295	3.595228	0.730131

Intermediate **E**

E(SMD/def2-QZVP*) = -1403.486541 E_h

$\Delta\Delta G(353) = 0.426426\ E_h$

Lowest frequency = 28.13 cm^{-1}

C	-0.648696	2.112464	-0.583715
C	1.005826	2.184309	1.025346
C	0.763189	3.529320	1.267285
C	-0.228808	4.175303	0.536702
C	-0.936001	3.459256	-0.424820
Ru	0.452831	-0.681574	-0.141373
C	-0.927707	-1.813767	1.286699
C	-0.419074	-2.655978	0.259339
C	0.969151	-2.823145	0.009089
C	1.861935	-2.044169	0.801973
C	1.367378	-1.157347	1.783605
C	-0.040658	-1.034478	2.049335
H	-1.131428	-3.187811	-0.381320
H	2.074440	-0.534394	2.343396
H	-1.683193	3.926149	-1.070086
H	-0.430732	5.238113	0.690854
H	1.366653	4.062562	2.003699
H	1.810255	1.657811	1.546501
N	0.287453	1.469814	0.148841
C	-2.536976	0.690625	-1.196246
C	-0.464319	0.280778	-2.049079
C	-2.492546	-0.668411	-1.566018
C	-3.644631	1.247177	-0.567638
C	-1.158895	-0.918623	-2.070876
C	-3.610405	-1.482797	-1.361754
C	-4.740955	0.415407	-0.351987
H	-3.662255	2.296740	-0.260888
H	-0.823764	-1.812311	-2.602293
C	-4.730666	-0.928603	-0.752788
H	-3.602772	-2.532720	-1.670620
H	-5.634222	0.824493	0.127400
H	-5.617956	-1.544435	-0.585726
N	-1.299040	1.285658	-1.516930
C	1.858107	-0.154199	-1.683500
H	2.259531	-1.097154	-2.085258
C	0.900036	0.567936	-2.612316
H	0.961477	0.230075	-3.660057
H	1.095464	1.651578	-2.608037
C	2.936929	0.671929	-1.104332
O	2.954956	1.880940	-1.052259
O	3.918222	-0.084283	-0.571320
H	-2.010092	-1.712312	1.410167
H	2.933698	-2.059748	0.581628
C	-0.514758	-0.100755	3.133754
H	0.262320	0.680790	3.244364
C	-0.575009	-0.884931	4.444529
H	0.389029	-1.361222	4.684100
H	-0.843290	-0.221805	5.281317

H	-1.337440	-1.680148	4.385279
C	-1.836280	0.587703	2.834648
H	-1.808022	1.150182	1.886219
H	-2.673507	-0.128600	2.775535
H	-2.082760	1.296244	3.640193
C	1.472195	-3.746386	-1.050492
H	2.432707	-3.400188	-1.461672
H	1.637367	-4.755506	-0.638589
H	0.752357	-3.844489	-1.877817
C	4.990856	0.622526	0.029909
H	5.722961	-0.126842	0.353367
H	5.455455	1.318863	-0.683884
H	4.643553	1.206932	0.897971

Intermediate **F**

E(SMD/def2-QZVP*) = -1632.962863 E_h

$\Delta\Delta G(353)$ = 0.480600 E_h

Lowest frequency = 26.52 cm^{-1}

C	1.608013	0.283791	1.654366
C	-0.451367	1.029455	2.380724
C	0.057541	1.459691	3.591875
C	1.417995	1.279526	3.840184
C	2.194673	0.682938	2.863285
Ru	-0.717904	0.234704	-0.520904
C	-0.966865	2.484190	-0.793608
C	0.297159	1.981695	-1.230913
C	0.421010	0.970137	-2.224252
C	-0.794091	0.367045	-2.680197
C	-2.034485	0.767588	-2.149575
C	-2.148392	1.867836	-1.227500
H	1.215363	2.389727	-0.791613
H	-2.932370	0.199939	-2.418455
H	3.259520	0.492076	3.014779
H	1.862940	1.587704	4.789583
H	-0.603852	1.928802	4.322659
H	-1.509320	1.173549	2.145089
N	0.293486	0.453610	1.414432
C	3.677923	0.149423	0.310954
C	2.245055	-1.612584	0.181136
C	4.299520	-0.876394	-0.435299
C	4.279030	1.392542	0.515921
C	3.375570	-1.975544	-0.488502
C	5.568551	-0.646184	-0.982924
C	5.538392	1.593546	-0.037791
H	3.782606	2.187745	1.081265
H	3.523303	-2.933206	-0.987805
C	6.176669	0.584343	-0.776680
H	6.067092	-1.423506	-1.568521
H	6.037427	2.556021	0.102922
H	7.166563	0.776146	-1.198958

N	2.406485	-0.306166	0.676536
C	-0.004369	-1.787370	-0.769831
C	0.947506	-2.323628	0.294864
H	1.136680	-3.401029	0.140833
H	0.533153	-2.234182	1.317036
H	-0.996077	3.267002	-0.030890
H	-0.760574	-0.497216	-3.350941
C	-3.521198	2.242996	-0.735600
H	-4.035426	1.284259	-0.524834
C	-4.271904	2.953279	-1.861899
H	-4.322563	2.343977	-2.777737
H	-5.303635	3.182871	-1.554061
H	-3.776712	3.905088	-2.118279
C	-3.527222	3.080469	0.530014
H	-2.971258	2.603842	1.355109
H	-3.090366	4.079529	0.360979
H	-4.560229	3.240747	0.875290
C	1.753476	0.566697	-2.758087
H	1.771145	-0.489287	-3.066645
H	1.990993	1.170617	-3.649527
H	2.557193	0.731015	-2.022414
O	-2.438633	-0.522266	0.698494
C	-2.759212	-1.072719	1.750304
O	-2.189763	-2.155264	2.208308
H	-1.658875	-2.588334	1.490062
C	-3.843197	-0.551492	2.626579
H	-4.682024	-1.264968	2.633065
H	-3.480604	-0.490487	3.664153
H	-4.195780	0.427019	2.278194
C	-1.204995	-2.597108	-1.062382
O	-1.855083	-2.574594	-2.076167
O	-1.555934	-3.411738	-0.020339
C	-2.739545	-4.186489	-0.202066
H	-2.644797	-4.834028	-1.084392
H	-2.859871	-4.793019	0.703960
H	-3.612306	-3.528876	-0.346155
H	0.548511	-1.743769	-1.721725

Transition state **TS4**

E(SMD/def2-QZVP*) = -1632.936304 E_h

ΔΔG(353) = 0.475273 E_h

Lowest frequency = -1522.07 cm^{-1}

C	-1.508011	-0.221414	1.718284
C	0.459131	-1.121753	2.532328
C	-0.104527	-1.382176	3.769449
C	-1.434120	-1.029677	3.984892
C	-2.137367	-0.439082	2.946812
Ru	0.816166	-0.373884	-0.383670
C	0.708843	-2.497173	-0.853089
C	-0.462533	-1.856224	-1.361744

C	-0.374865	-0.768662	-2.256406
C	0.935170	-0.272779	-2.562321
C	2.090831	-0.891677	-2.058732
C	1.999202	-2.044299	-1.211347
H	-1.453194	-2.182614	-1.024977
H	3.067908	-0.430573	-2.236441
H	-3.179403	-0.129392	3.057569
H	-1.913390	-1.203146	4.951572
H	0.499371	-1.849464	4.549426
H	1.506226	-1.363829	2.330780
N	-0.220454	-0.559544	1.514373
C	-3.453103	-0.232737	0.225495
C	-2.204932	1.666540	0.220164
C	-4.138256	0.743589	-0.531439
C	-3.935450	-1.532600	0.387761
C	-3.328479	1.929610	-0.508535
C	-5.346107	0.394455	-1.152174
C	-5.135638	-1.850479	-0.238254
H	-3.394137	-2.276449	0.982535
H	-3.542211	2.880520	-0.996758
C	-5.831355	-0.897214	-1.000383
H	-5.894178	1.128683	-1.749129
H	-5.544536	-2.859062	-0.134279
H	-6.771965	-1.180802	-1.479764
N	-2.255444	0.332616	0.668232

C	0.179482	1.984003	-0.370359
C	-1.008329	2.517051	0.438604
H	-1.255207	3.540309	0.115143
H	-0.746282	2.596396	1.510813
H	0.600404	-3.303369	-0.122103
H	1.041448	0.653667	-3.136504
C	3.270477	-2.658355	-0.690892
H	3.929729	-1.809646	-0.429207
C	3.917656	-3.447643	-1.829540
H	4.105406	-2.822598	-2.716752
H	4.882343	-3.866103	-1.504225
H	3.273695	-4.288298	-2.139090
C	3.092511	-3.525079	0.541301
H	2.602587	-2.983081	1.366015
H	2.503786	-4.432946	0.324158
H	4.073072	-3.863852	0.907920
C	-1.591351	-0.145018	-2.850689
H	-1.495282	0.947365	-2.951707
H	-1.739059	-0.548322	-3.866430
H	-2.498597	-0.366938	-2.268209
O	2.550408	-0.006480	0.757055
C	2.648394	1.002134	1.522695
O	1.694964	1.798096	1.689091
H	0.910586	1.549017	0.646202
C	3.950768	1.244910	2.207129

H	4.512807	0.312345	2.341587
H	4.547152	1.912404	1.564090
H	3.793035	1.756910	3.164942
C	1.258364	2.944719	-0.780117
O	2.202469	2.613674	-1.458541
O	1.085363	4.165123	-0.289274
C	2.117330	5.107067	-0.552767
H	3.069536	4.760865	-0.122579
H	2.253140	5.247170	-1.635174
H	1.810161	6.047829	-0.082729
H	-0.202997	1.582879	-1.316873

Intermediate **G**

E(SMD/def2-QZVP*) = -1632.965014 E_h

ΔΔG(353) = 0.479009 E_h

Lowest frequency = 27.00 cm^{-1}

C	-1.358773	0.404525	1.639119
C	0.472337	-0.468308	2.743913
C	0.032102	0.019061	3.963759
C	-1.147636	0.755852	4.003858
C	-1.856318	0.938415	2.824151
Ru	0.803465	-0.679445	-0.266550
C	0.776972	-2.794468	-0.265071
C	-0.437326	-2.408794	-0.905684
C	-0.418903	-1.512133	-1.988078
C	0.854924	-1.005287	-2.412288
C	2.063720	-1.455711	-1.853445
C	2.051291	-2.362031	-0.752059
H	-1.402222	-2.720776	-0.492205
H	3.007180	-0.989745	-2.155389
H	-2.804033	1.482950	2.790397
H	-1.517273	1.172587	4.943986
H	0.620343	-0.168791	4.863767
H	1.420367	-1.006914	2.663257
N	-0.199238	-0.272284	1.597402
C	-3.232008	-0.308065	0.270749
C	-2.345490	1.715094	-0.260318
C	-4.115521	0.337581	-0.621363
C	-3.498823	-1.561678	0.821096
C	-3.527609	1.611108	-0.935488
C	-5.283807	-0.329192	-1.015840
C	-4.666498	-2.199808	0.414562
H	-2.820039	-2.024503	1.546402
H	-3.925247	2.366546	-1.613687
C	-5.542542	-1.592968	-0.501008
H	-5.982157	0.140936	-1.713781
H	-4.910674	-3.185377	0.820159
H	-6.451139	-2.122094	-0.800166
N	-2.121466	0.520115	0.456468
C	-0.228699	2.300670	-1.360468

C	-1.280785	2.750006	-0.354205
H	-1.724273	3.706478	-0.670301
H	-0.793734	2.922570	0.619237
H	0.729182	-3.404622	0.642086
H	0.882637	-0.197704	-3.152612
C	3.357278	-2.749477	-0.114510
H	3.970775	-1.829090	-0.105942
C	4.032711	-3.785246	-1.014592
H	4.185442	-3.411446	-2.039247
H	5.017809	-4.058013	-0.606450
H	3.428580	-4.706381	-1.076777
C	3.235804	-3.256838	1.310035
H	2.726571	-2.533539	1.966637
H	2.698402	-4.219270	1.362243
H	4.236785	-3.429762	1.732329
C	-1.669960	-1.051945	-2.653311
H	-1.692094	0.042592	-2.773671
H	-1.723484	-1.491782	-3.663027
H	-2.568193	-1.359239	-2.099324
O	2.412023	0.188909	0.607590
C	2.344853	1.417050	1.051967
O	1.304869	2.056916	1.110672
H	0.034780	1.234646	-1.182046
C	3.674019	1.992391	1.434182
H	4.162663	2.325347	0.503006
H	3.540430	2.860561	2.092702
H	4.328503	1.243221	1.899782
C	1.085681	3.029518	-1.393602
O	2.115167	2.520485	-1.765877
O	0.961427	4.293149	-1.014492
C	2.154370	5.061401	-0.954692
H	2.818309	4.667132	-0.169667
H	2.687919	5.036109	-1.916022
H	1.856622	6.086987	-0.709858
H	-0.646355	2.311120	-2.386078

Intermediate **H**

E(SMD/def2-QZVP*) = -1632.988615 E_h

ΔΔG(353) = 0.478226 E_h

Lowest frequency = 23.25 cm^{-1}

C	-0.308437	0.877812	1.860823
C	-0.614725	-1.328533	2.488132
C	-0.788116	-0.977479	3.819242
C	-0.733815	0.366674	4.171261
C	-0.479326	1.302871	3.176690
Ru	-0.534243	-1.182521	-0.537079
C	-2.401380	-0.093225	-0.944055
C	-1.410284	0.341014	-1.851092
C	-0.676581	-0.601793	-2.629267
C	-0.990289	-1.981971	-2.490318

C	-1.966520	-2.397684	-1.546734
C	-2.700858	-1.467368	-0.753870
H	-1.147133	1.403145	-1.892583
H	-2.085129	-3.468494	-1.343637
H	-0.394502	2.370975	3.391542
H	-0.869187	0.682419	5.208723
H	-0.965781	-1.757515	4.561895
H	-0.644343	-2.375599	2.173190
N	-0.403166	-0.423278	1.513790
C	-0.976428	2.719233	0.348288
C	1.190516	2.038677	0.232801
C	-0.332273	3.462284	-0.669106
C	-2.322579	2.895583	0.673669
C	1.031977	3.013806	-0.715222
C	-1.082002	4.401979	-1.393096
C	-3.038885	3.833060	-0.062047
H	-2.796233	2.327064	1.482222
H	1.826209	3.413877	-1.345417
C	-2.425127	4.573388	-1.087211
H	-0.612619	4.992086	-2.185287
H	-4.093735	4.005015	0.168518
H	-3.016642	5.304881	-1.643991
N	-0.041985	1.827698	0.864183
C	3.540087	1.319071	-0.271126
C	2.410576	1.355949	0.733824
H	2.749103	1.857898	1.662095
H	2.181043	0.321673	1.032122
H	-2.856884	0.659036	-0.293298
H	-0.388081	-2.729357	-3.015120
C	-3.714374	-1.965504	0.247776
H	-3.256860	-2.849797	0.736582
C	-4.966715	-2.424764	-0.496129
H	-4.744084	-3.203722	-1.242363
H	-5.708480	-2.834725	0.206373
H	-5.435991	-1.577444	-1.024570
C	-4.066691	-0.948316	1.321128
H	-3.179907	-0.567693	1.856286
H	-4.606445	-0.084009	0.896314
H	-4.734562	-1.402566	2.068344
C	0.468063	-0.149033	-3.466780
H	1.229277	-0.935978	-3.565719
H	0.114383	0.118101	-4.476297
H	0.937068	0.743486	-3.024438
O	0.488359	-2.855052	0.296150
C	1.628577	-2.359801	0.026924
O	1.622045	-1.248558	-0.583863
H	3.175393	0.946017	-1.246182
C	2.886805	-3.024733	0.431077
H	3.356503	-2.428433	1.229600
H	2.699048	-4.048945	0.775212

H	3.597973	-3.019363	-0.407740
C	4.640141	0.389540	0.157142
O	4.551547	-0.427469	1.040363
O	5.731212	0.561016	-0.584191
C	6.833513	-0.288232	-0.300871
H	7.158205	-0.173040	0.744085
H	6.568013	-1.343787	-0.466901
H	7.640266	0.006637	-0.981338
H	3.972479	2.313614	-0.471349

Alkene **3a**

E(SMD/def2-QZVP*) = -306.978933 E_h

ΔΔG(353) = 0.057218 E_h

Lowest frequency = 98.10 cm^{-1}

C	-0.041115	0.115962	-0.000001
O	0.056877	1.316312	0.000005
O	1.014428	-0.712454	0.000034
C	2.285179	-0.092297	0.000079
H	3.031048	-0.896465	0.000102
H	2.415626	0.542922	-0.890398
H	2.415565	0.542917	0.890568
C	-1.309380	-0.648391	-0.000046
C	-2.479582	-0.011171	-0.000087
H	-1.230126	-1.740092	-0.000044
H	-3.431146	-0.548690	-0.000120
H	-2.502018	1.083925	-0.000087

HOAc

E(SMD/def2-QZVP*) = -229.469374 E_h

ΔΔG(353) = 0.028749 E_h

Lowest frequency = 91.72 cm^{-1}

C	-0.092892	0.124244	-0.000006
O	-0.646051	1.190449	-0.000050
O	-0.766277	-1.039274	-0.000003
H	-1.709529	-0.809639	-0.000040
C	1.384835	-0.103073	0.000051
H	1.672718	-0.692564	0.883434
H	1.672778	-0.692617	-0.883278
H	1.911001	0.858393	0.000041
